# Supplementary material for: TNFSF14+ natural killer cells prevent spontaneous abortion by restricting leucine-mediated decidual stromal cell senescence
Source: EMBO J. 2024 Sep 11;43(21):5018–36. doi: 10.1038/s44318-024-00220-3 (PMC11535022; doi:10.1038/s44318-024-00220-3)
Supplement: Supplementary file 1 — Appendix [file 44318_2024_220_MOESM1_ESM.pdf]

Appendix

The Appendix file include 2 tables and 7 figures.

Table of contents

Appendix Table S1. Primer sequences of related genes……………………….…….……….....2

Appendix Table S2. Human antibodies for flow cytometry assays…………………...…….3

Appendix Figure S1. Expression of senescence-related molecules in aging mice……….…4-5

Appendix Figure S2. Leucine accumulation induces oxidative stress and activates MAP2K3/p38 signaling pathway during decidualization………………………………..6-7

Appendix Figure S3. Decidual NK cells inhibited excessive senescence of DSCs………….8

Appendix Figure S4. The distribution of TNFSF14 at the maternal-fetal interface………9-10

Appendix Figure S5. Decidual NK cells inhibited excessive senescence of DSCs through TNFSF14/TNFRSF14/MAP2K3/p38 pathway…………………………………….……..11

Appendix Figure S6. Verification of NK cell depletion in pregnant mice……………….12-13

Appendix Figure S7. Metformin reduced the degree of senescence in the uterus of aging pregnant mice……………………………………………………….……...……….14-15

Appendix Table S1. Primer sequences of related genes.

ACTB	Forward GCCGACAGGATGCAGAAGGAGATCA<br>Reverse AAGCATTTGCGGTGGACGATGGA
CDKN2A	Forward CTACTGAGGAGCCAGCGTCT<br>Reverse CTGCCCATCATCATGACCT
CDKN1A	Forward ACAGCAGAGGAAGACCATGTGGACC<br>Reverse CGTTTTCGACCCTGAGAGTCTCCAG
TP53	Forward GCCCATCCTCACCATCATCACAC<br>Reverse GCACAAACACGCACCTCAAAGC
SLC3A2	Forward TGAATGAGTTAGAGCCCGAGA<br>Reverse GTCTTCCGCCACCTTGATCTT
HSF1	Forward CCATGAAGCATGAGAATGAGGC<br>Reverse CTTGTTGACGACTTTCTGTTGC
TNFRSF14	Forward CGTCTTGAGGCTGGTGCTGTATC<br>Reverse CCTTCACACGATAACCTGGACTGC

Appendix Table S2. Human antibodies for flow cytometry assays.

Allophycocyanin (APC)-Cy7 anti-human PTPRC antibody (304014; Biolegend, CA, USA)
FITC anti-human CD3 antibody (317306; Biolegend, CA, USA)
Brilliant Violet® 421 (BV421) anti-human NCAM1 antibody (562751; BD Pharmingen, USA)
PE/Cyanine7 anti-human TNFSF14 Antibody (318708; Biolegend, CA, USA)
PE/Cyanine7 anti-mouse Ptprc Antibody (103114; Biolegend, CA, USA)
APC-Cy7 anti-mouse NK1.1 Antibody (108724; Biolegend, CA, USA)
BV605 anti-mouse CD3 Antibody (101236; Biolegend, CA, USA)
APC anti-human/mouse vimentin Antibody (IC2105A; R&D Systems, USA)

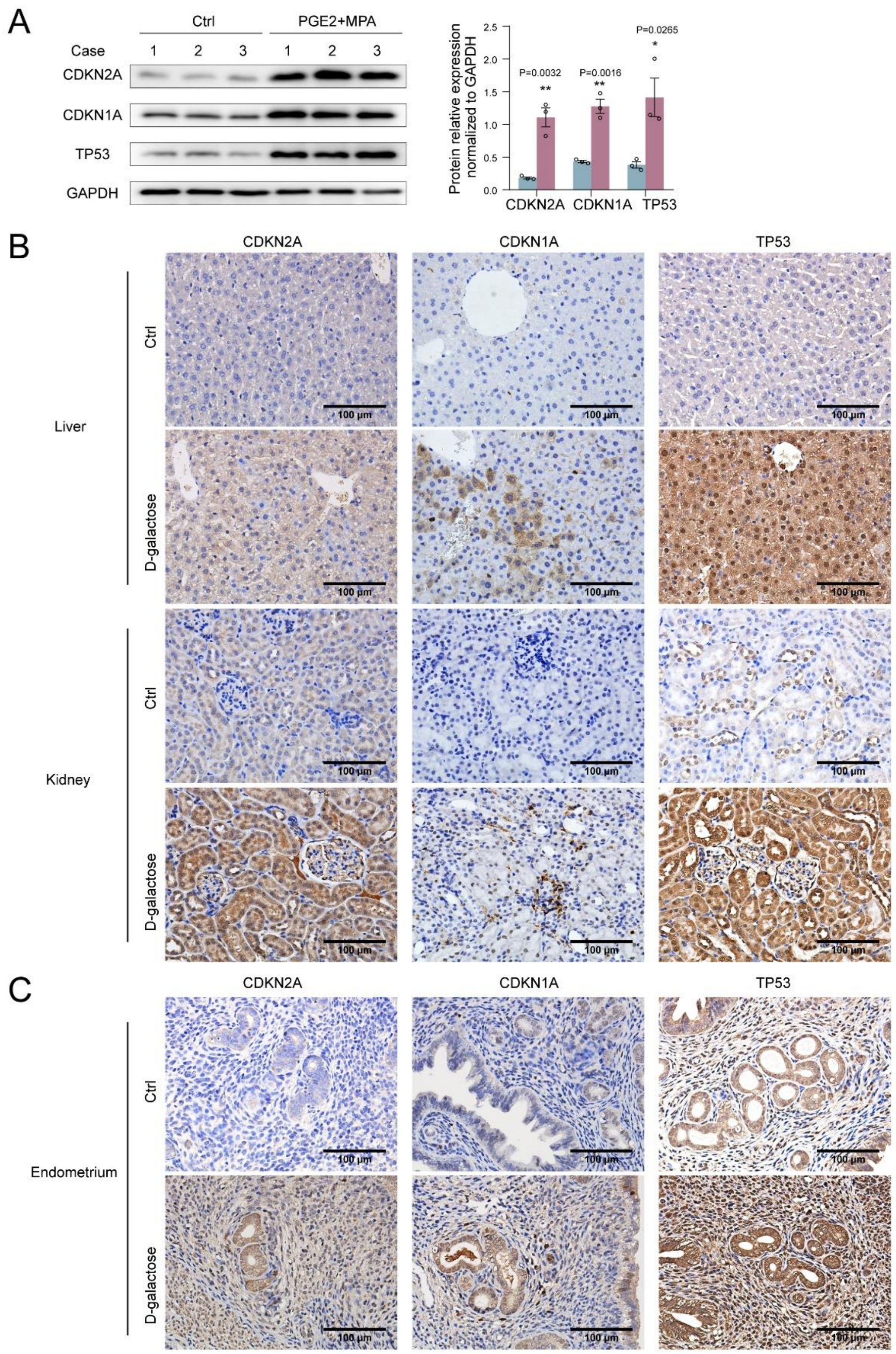

Appendix Figure S1. Expression of senescence-related molecules in aging mice

A. Decidualized hESCs were induced with PGE_2 (1 μM) plus MPA (1μM) for 48h, and then expression of CDKN2A, CDKN1A and TP53 was measured by western blot (n=3 biological

replicates per group), relative expression levels of proteins were standardized using internal reference GAPDH.

B, C. CDKN2A, CDKN1A and TP53 expression in the liver, kidney and uterus of control mice and D-galactose-treated mice (n=4 biological replicates for each group). Scale bar, 100 μm. Data were presented as mean ± SEM. * $P < 0.05$, ** $P < 0.01$, using a two-tailed, unpaired Student' s t test.

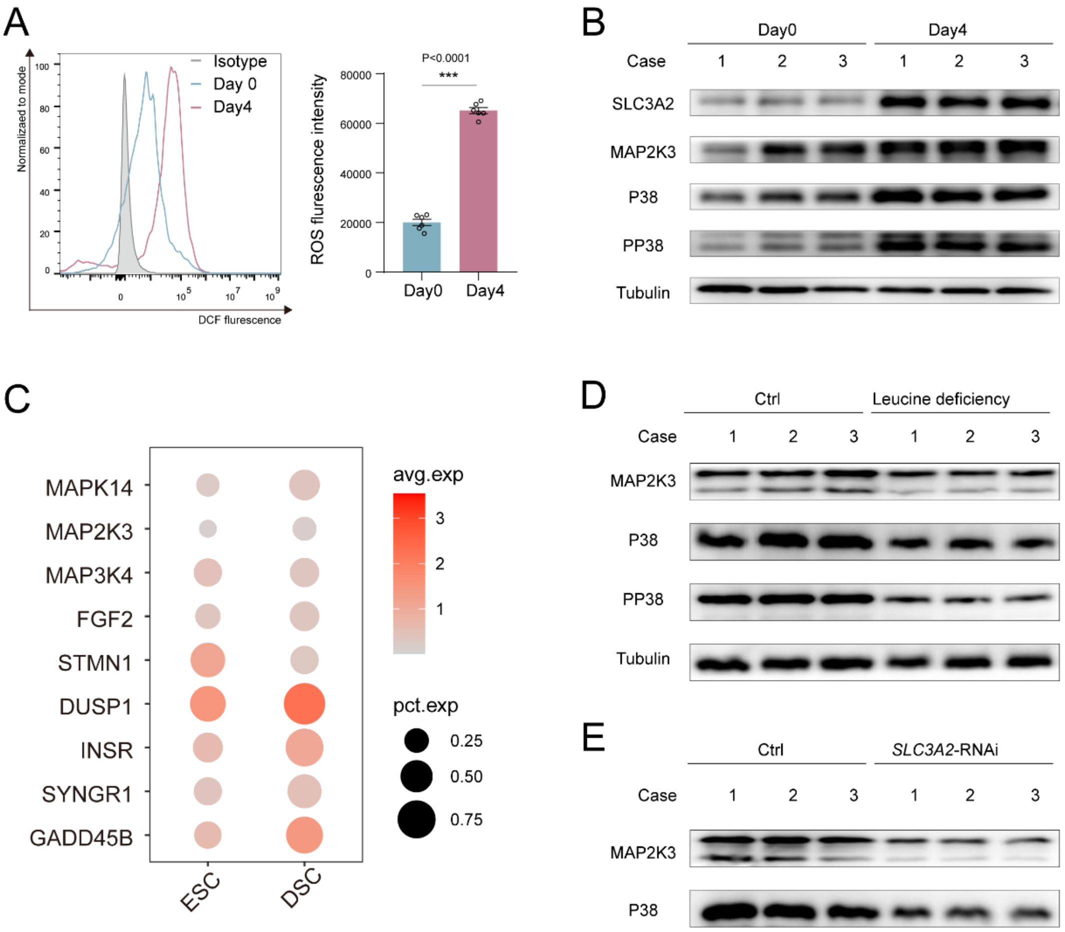

Appendix Figure S2. Leucine accumulation induces oxidative stress and activates MAP2K3/p38 signaling pathway during decidualization.

A. ROS levels in hESCs were detected by flow cytometry during the decidualization (n = 6 biological replicates per group).

B. MAP2K3/p38 pathway of hESCs were measured by western blotting (n=3 biological replicates per group) during decidualization.

C. Bubble diagram showing the average expression of MAPK pathway-related genes in ESCs and DSCs.

D. hESCs were cultured under the medium of leucine deficiency during decidualization, and MAP2K3/p38 pathway were detected by western blotting (n=3 biological replicates per group).

E. During decidualization, the expression of MAP2K3/p38 pathway in control and si*SLC3A2* hESCs were measured by western blotting (n=3 biological replicates per group).

P38: total p38; PP38: phosphorylated p38.

Data were presented as mean ± SEM. *** $P < 0.001$, using a two-tailed, unpaired Student's t test.

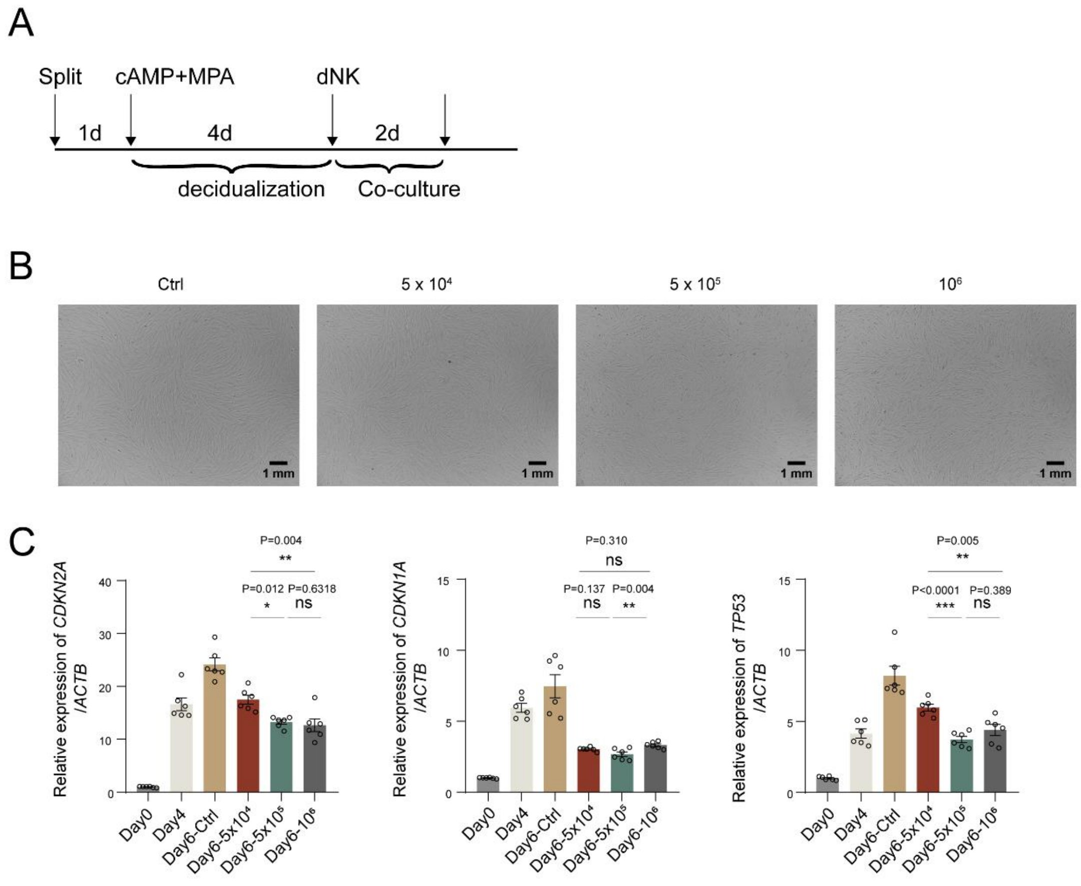

Appendix Figure S3. Decidual NK cells inhibited excessive senescence of DSCs.

A, B, C. Decidualized hESCs were co-cultured with different quantities of dNK cells, transcription levels of *CDKN2A, CDKN1A* and *TP53* were detected by qRT-PCR (n=6 biological replicates per group).

Data were presented as mean ± SEM. * P < 0.05, ** P < 0.01, *** P < 0.001, ns, no significance, using one-way ANOVA with Bonferroni multiple comparisons test.

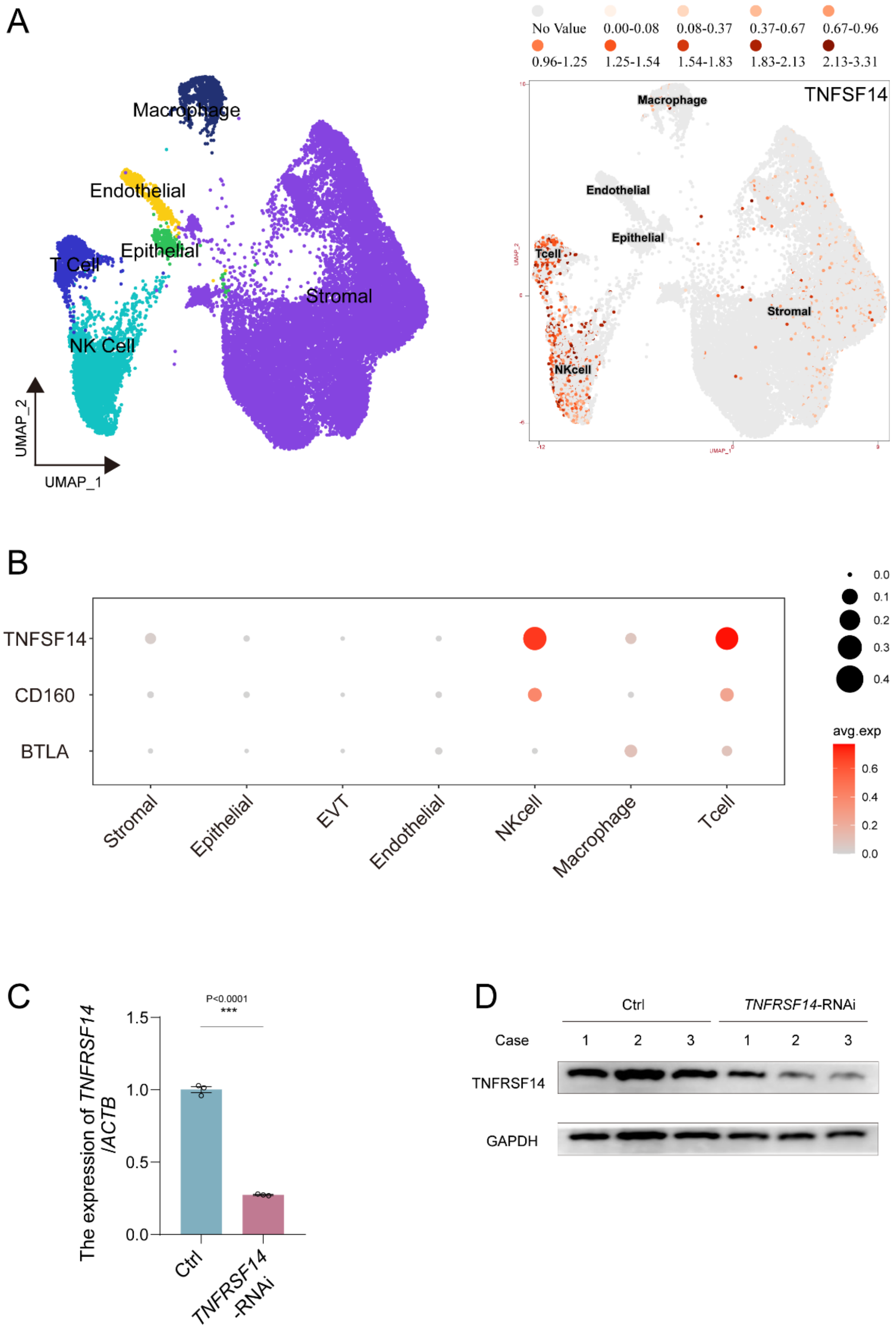

Appendix Figure S4. The distribution of TNFSF14 at the maternal-fetal interface.

A, B. UMAP map and Bubble diagram showing the expression of TNFSF14 in the decidua

C, D. After treatment of si*TNFRSF14*, expression of TNFRSF14 were verified by qRT-PCR

and western blotting (n = 3 biological replicates per group). Data were presented as mean ± SEM. *** $P < 0.001$, using a two-tailed, unpaired Student's t test.

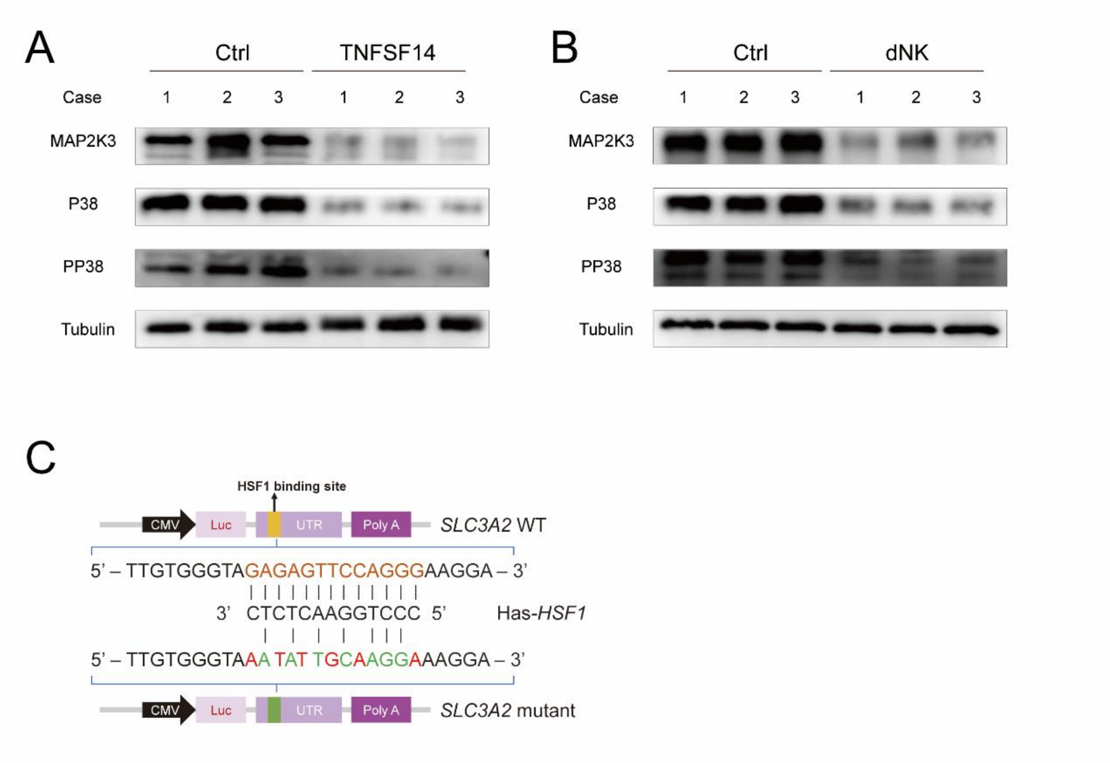

Appendix Figure S5. Decidual NK cells inhibited excessive senescence of DSCs through TNFSF14/TNFRSF14/MAP2K3/p38 pathway.

A, B. Decidualized hESCs were treated with TNFSF14 (250ng/mL), or co-cultured with dNK cells (5×10^5) for 48h, MAP2K3/p38 pathway were measured by western blotting (n=3 biological replicates per group).

C. Schematic diagram of the prediction of the binding region of the transcription factor HSF1 and WT or mutated *SLC3A2* promoter region.

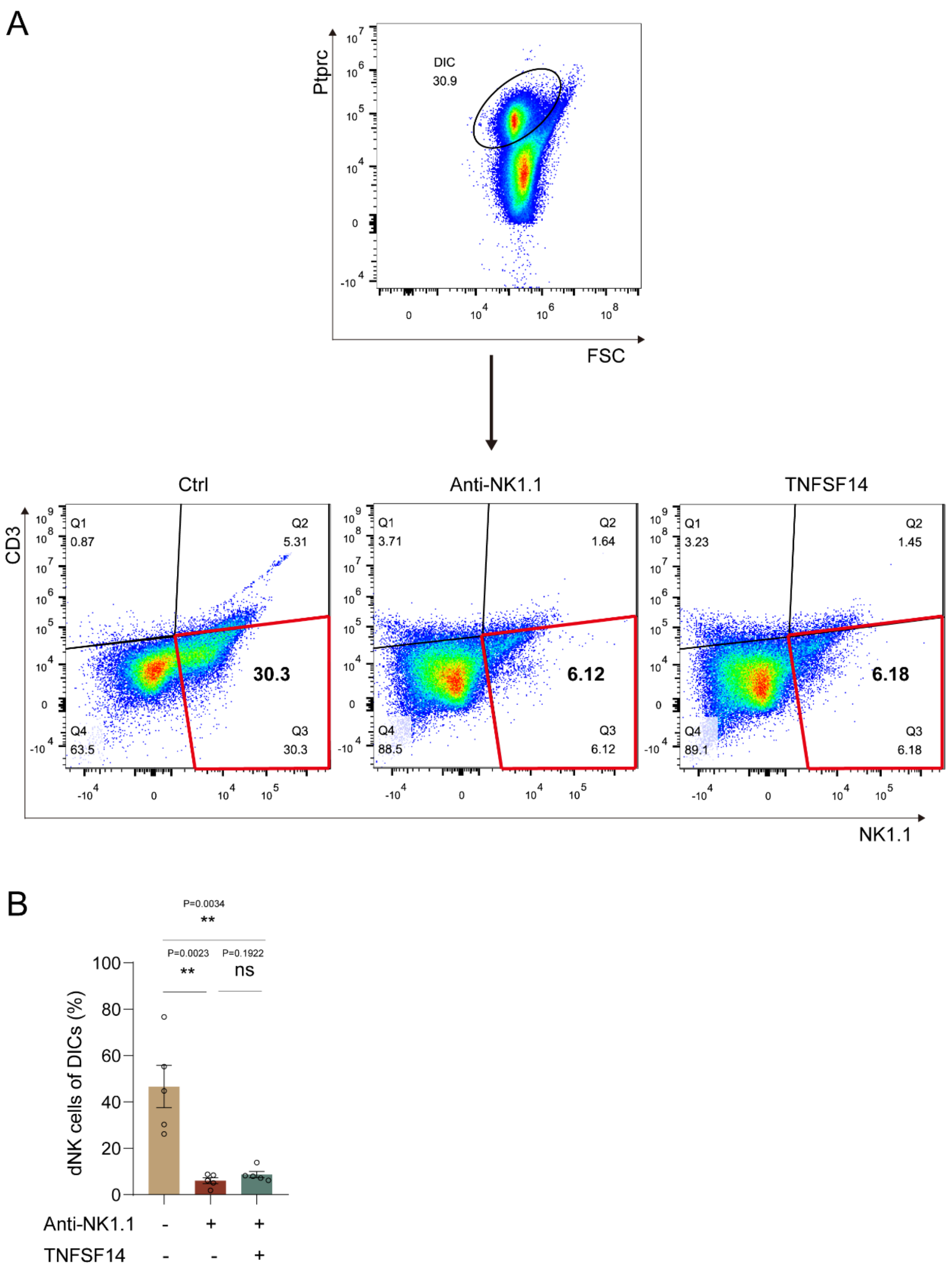

Appendix Figure S6. Verification of NK cell depletion in pregnant mice.

A,B. C57BL/6 pregnant mice were treated with NK1.1 neutralizing antibody (n = 5 biological replicates per group) or isotype control IgG antibody (n = 5 biological replicates per group) by

intraperitoneal injection, and at the same time, mice were injected with recombinant TNFSF14 protein intraperitoneally (n = 5 biological replicates per group), the depletion degree of uterine NK cells in pregnant mice at the gestation of day 13.5 was verified by flow cytometry.

Data were presented as mean ± SEM. ** $P < 0.01$, ns, no significance, using a two-tailed, unpaired Student's t test.

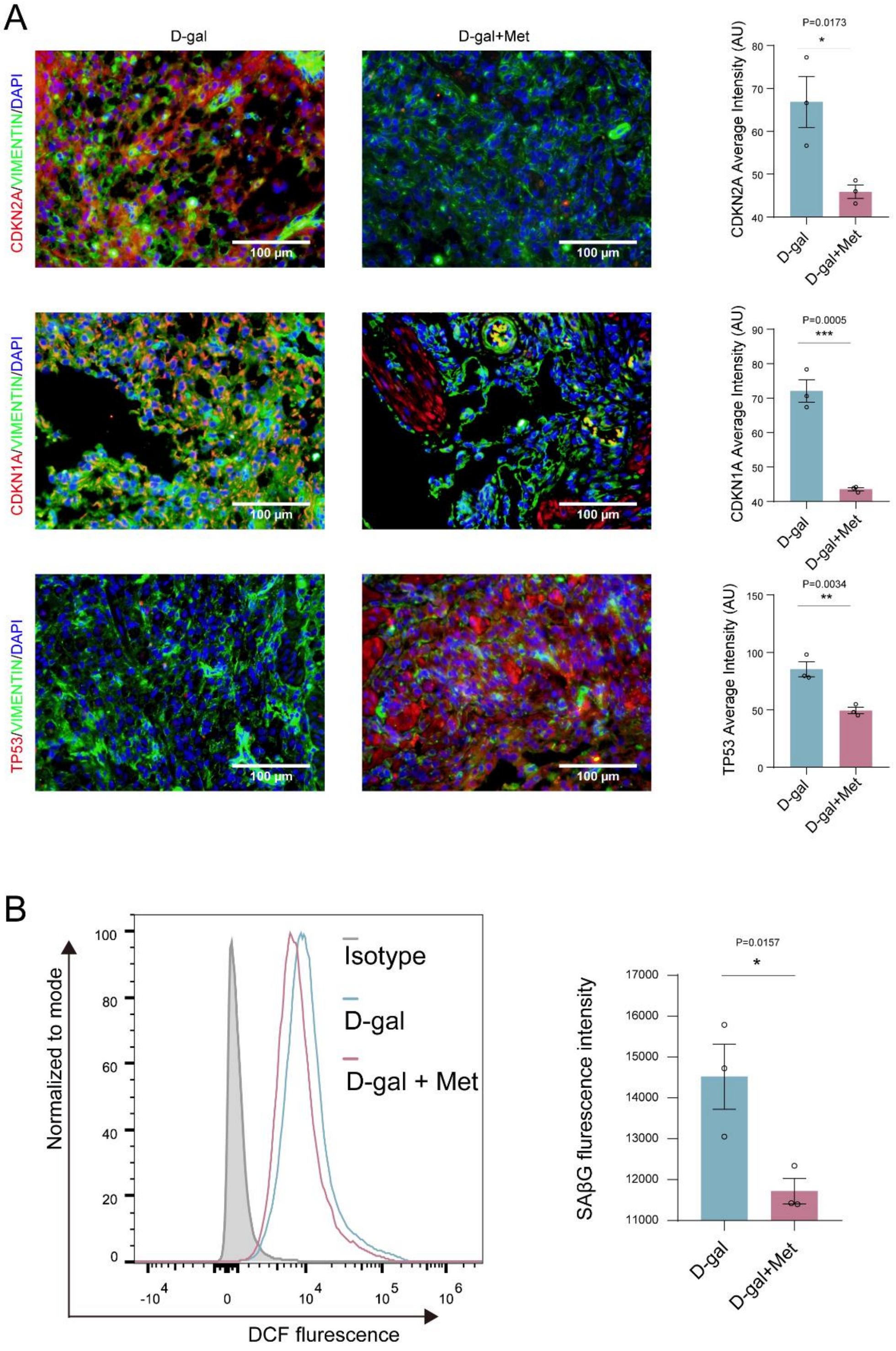

Appendix Figure S7. Metformin reduced the degree of senescence in the uterus of aging pregnant mice.

A, B. After treatment with metformin (200mg/kg, every other day) in aging pregnant mice, CDKN2A, CDKN1A and TP53 expression of DSCs were verified by immunofluorescence, and

SAβG activity of DSCs were measured by flow cytometry (n=3 biological replicates per group). Data were presented as mean ± SEM. * $P < 0.05$, ** $P < 0.01$, using a one-tailed, unpaired Student's t test. Scale bar, 100 μm.
