# Supplementary figures and images for: TNFSF14+ natural killer cells prevent spontaneous abortion by restricting leucine-mediated decidual stromal cell senescence (part 1 of 4)
Source: EMBO J. 2024 Sep 11;43(21):5018–36. doi: 10.1038/s44318-024-00220-3 (PMC11535022; doi:10.1038/s44318-024-00220-3)

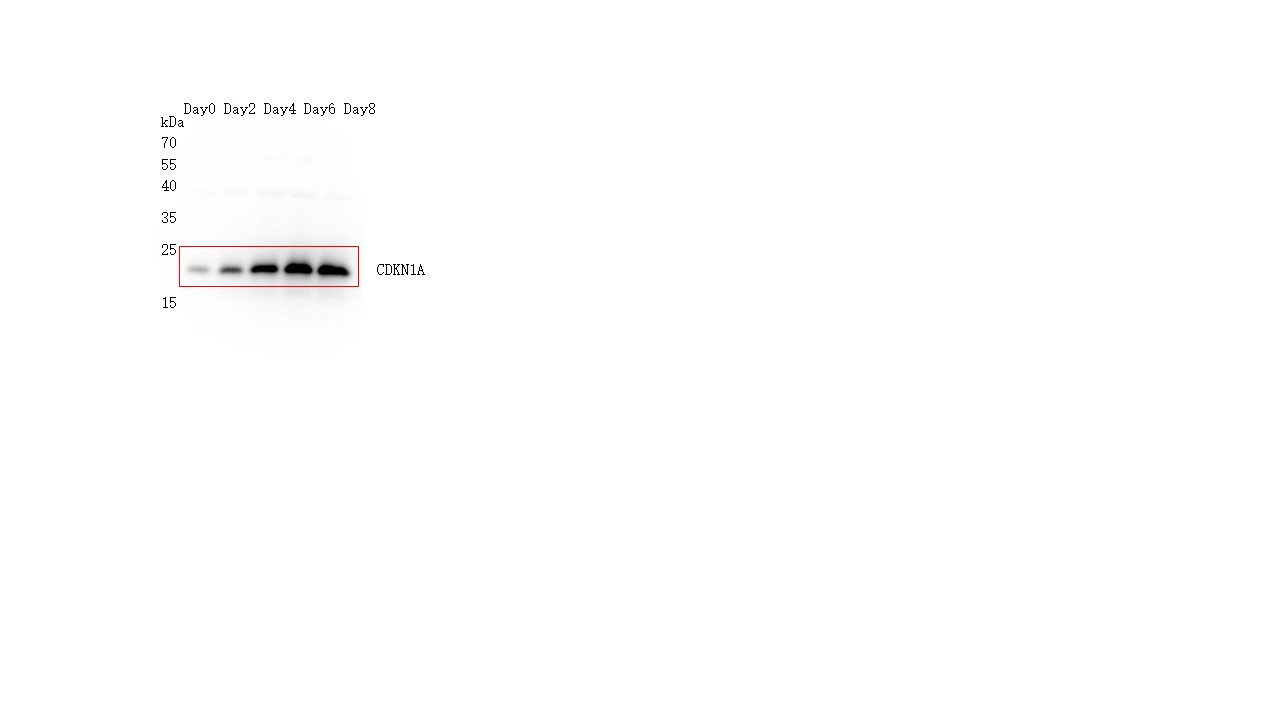

Supplement: Supplementary file 3 — Source data Fig. 1 [file 44318_2024_220_MOESM3_ESM.zip › Figure1/1D/western CDKN1A.jpg]

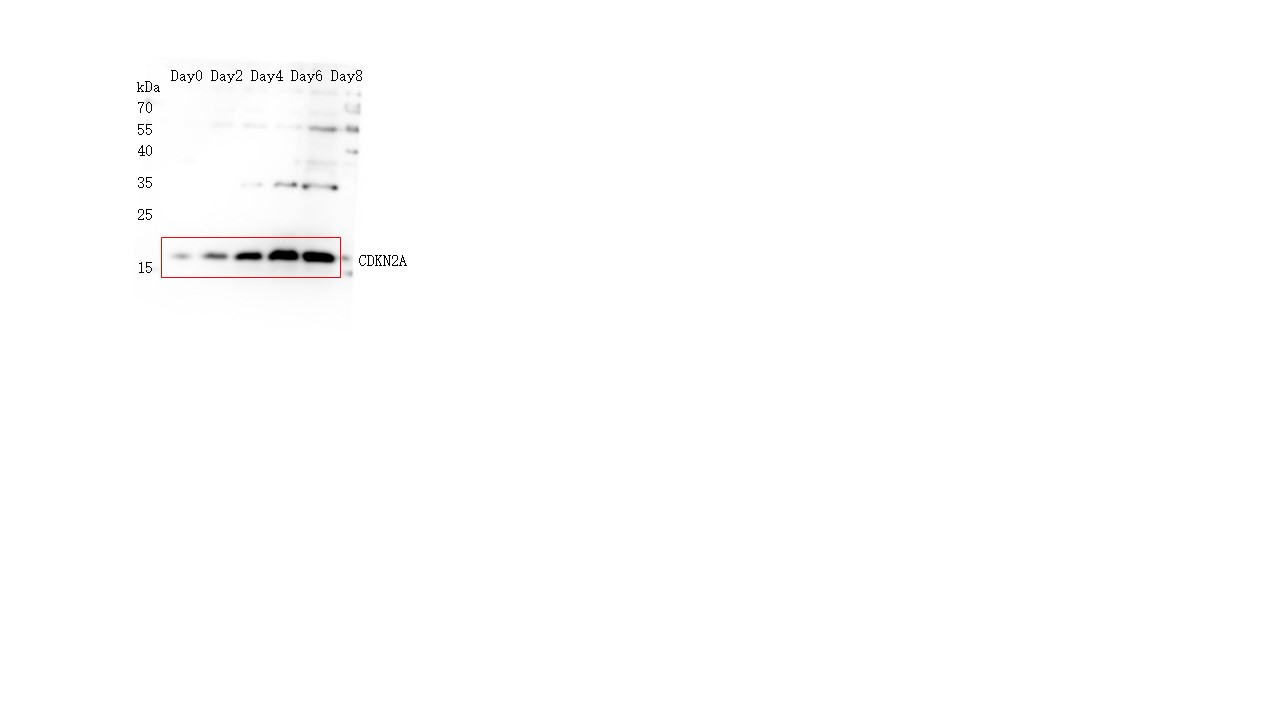

Supplement: Supplementary file 3 — Source data Fig. 1 [file 44318_2024_220_MOESM3_ESM.zip › Figure1/1D/western CDKN2A.jpg]

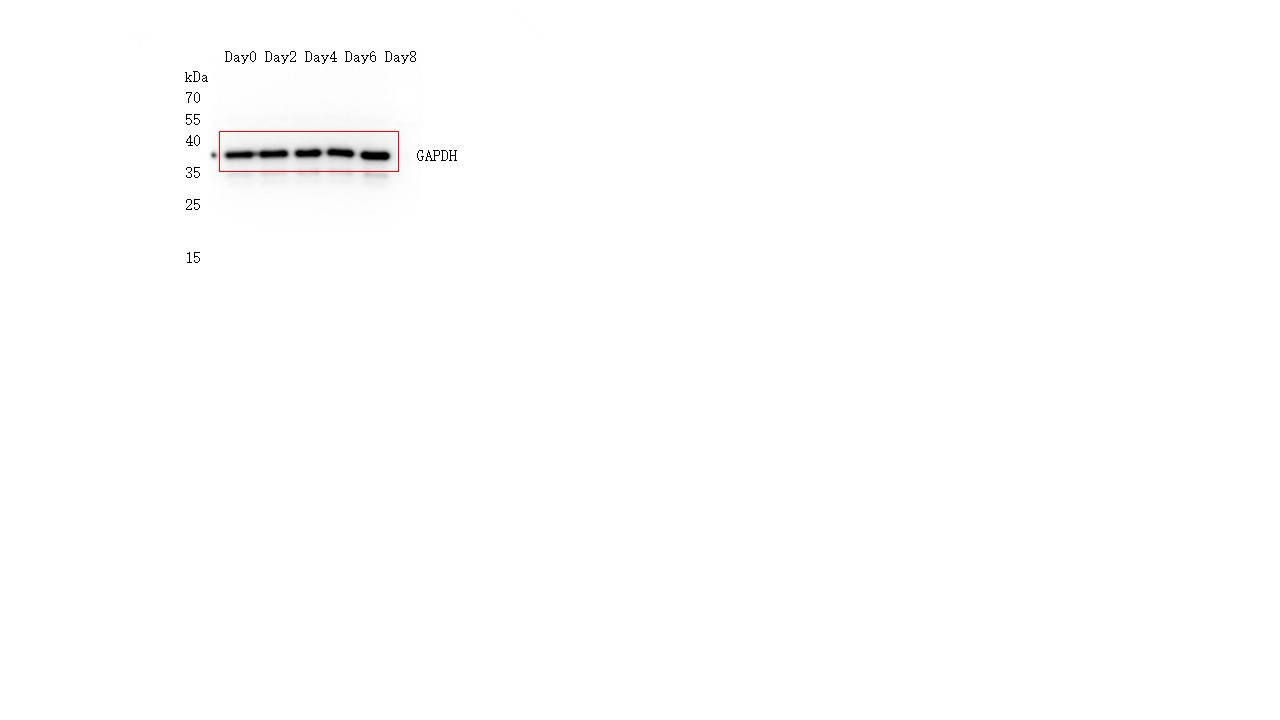

Supplement: Supplementary file 3 — Source data Fig. 1 [file 44318_2024_220_MOESM3_ESM.zip › Figure1/1D/western GAPDH.jpg]

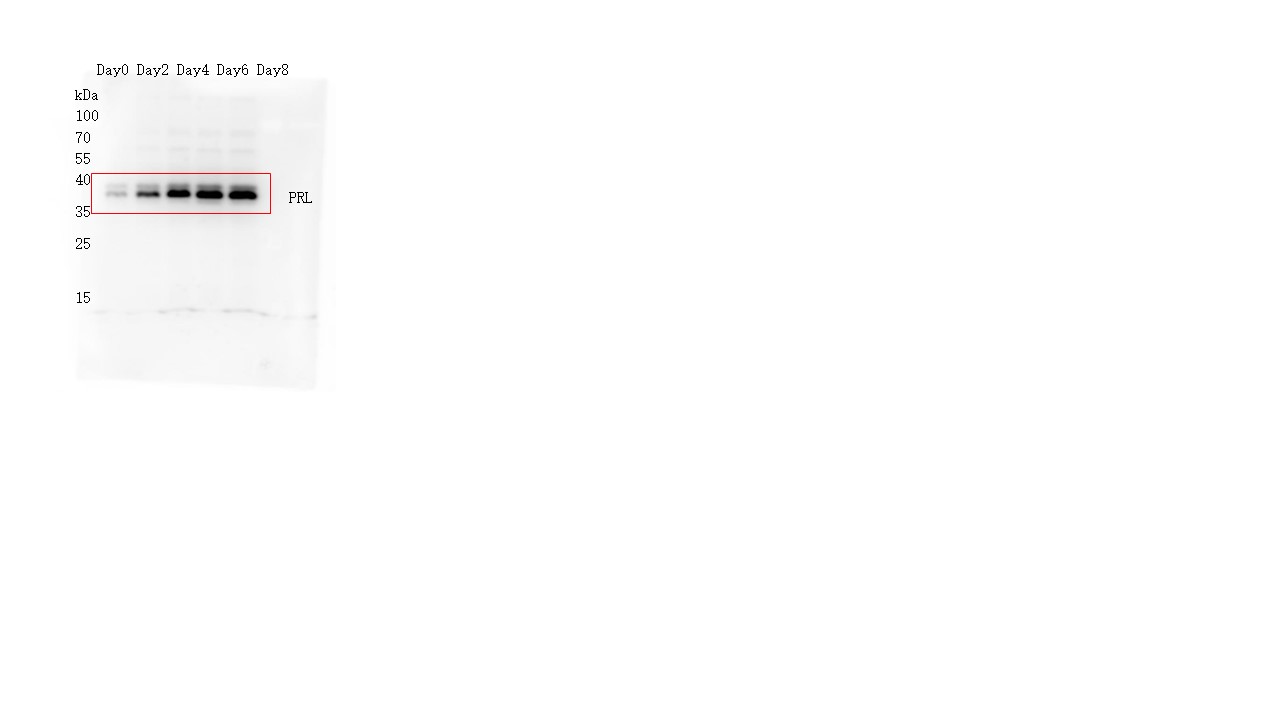

Supplement: Supplementary file 3 — Source data Fig. 1 [file 44318_2024_220_MOESM3_ESM.zip › Figure1/1D/western PRL.jpg]

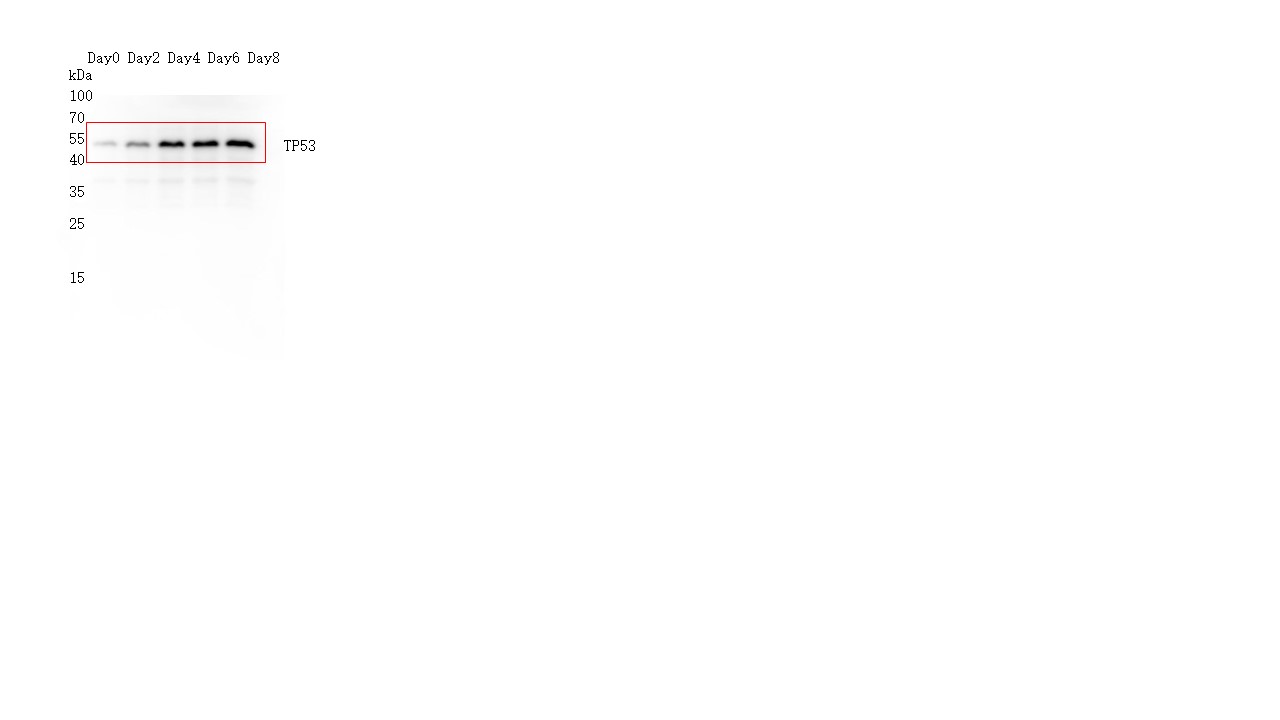

Supplement: Supplementary file 3 — Source data Fig. 1 [file 44318_2024_220_MOESM3_ESM.zip › Figure1/1D/western TP53.jpg]

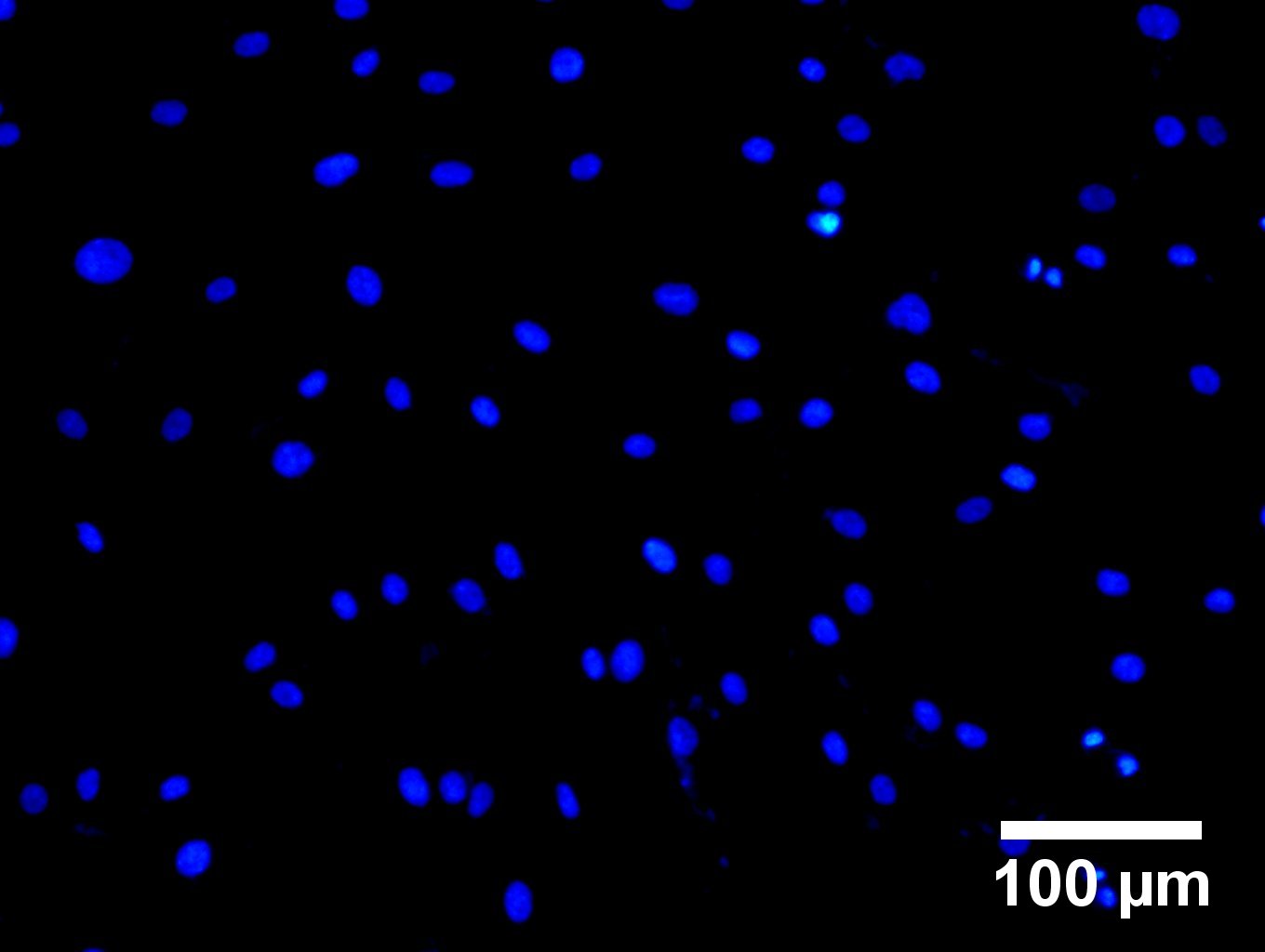

Supplement: Supplementary file 3 — Source data Fig. 1 [file 44318_2024_220_MOESM3_ESM.zip › Figure1/1E/Image/Day0-1 (1).jpg]

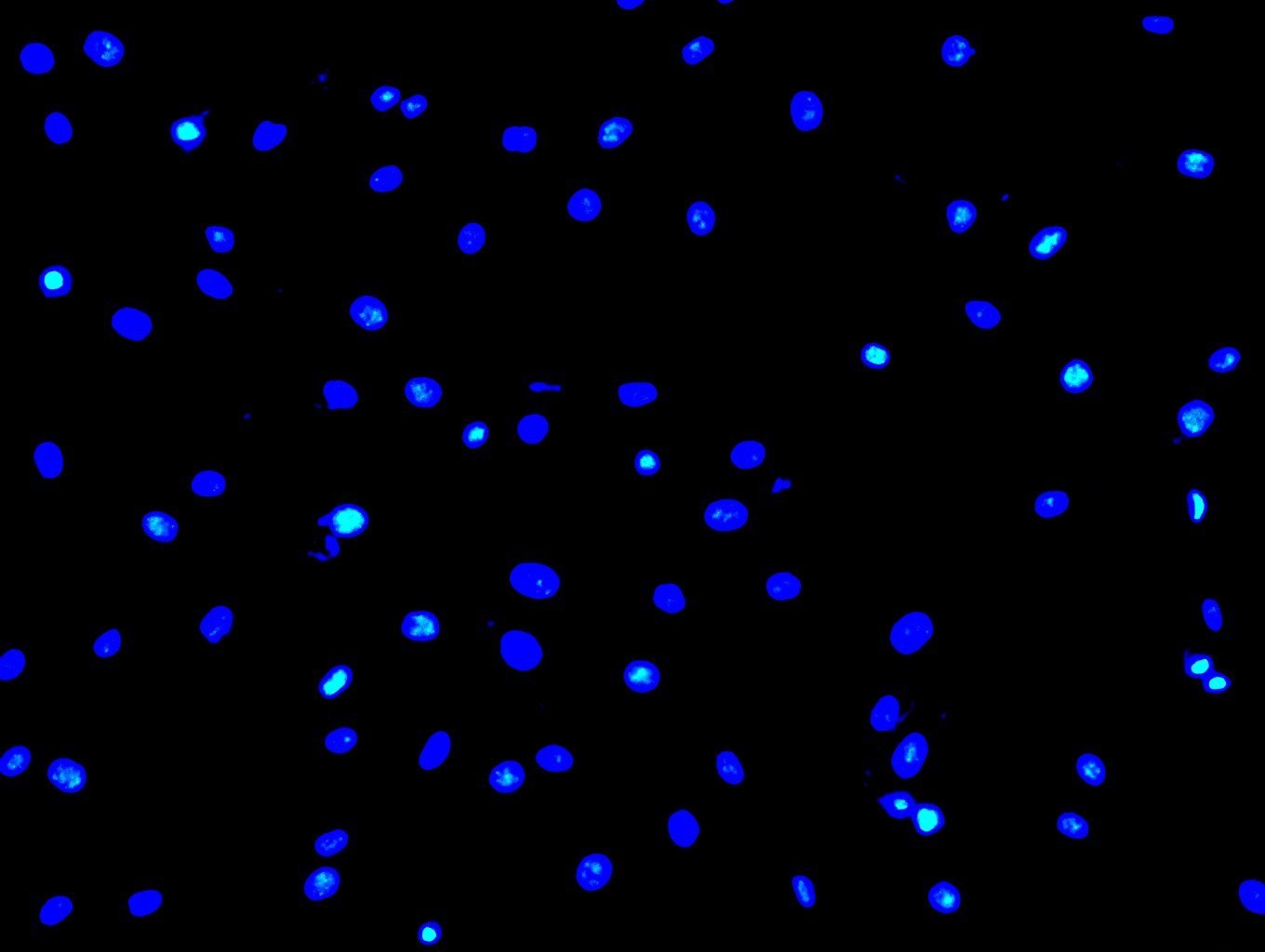

Supplement: Supplementary file 3 — Source data Fig. 1 [file 44318_2024_220_MOESM3_ESM.zip › Figure1/1E/Image/Day0-1 (2).jpg]

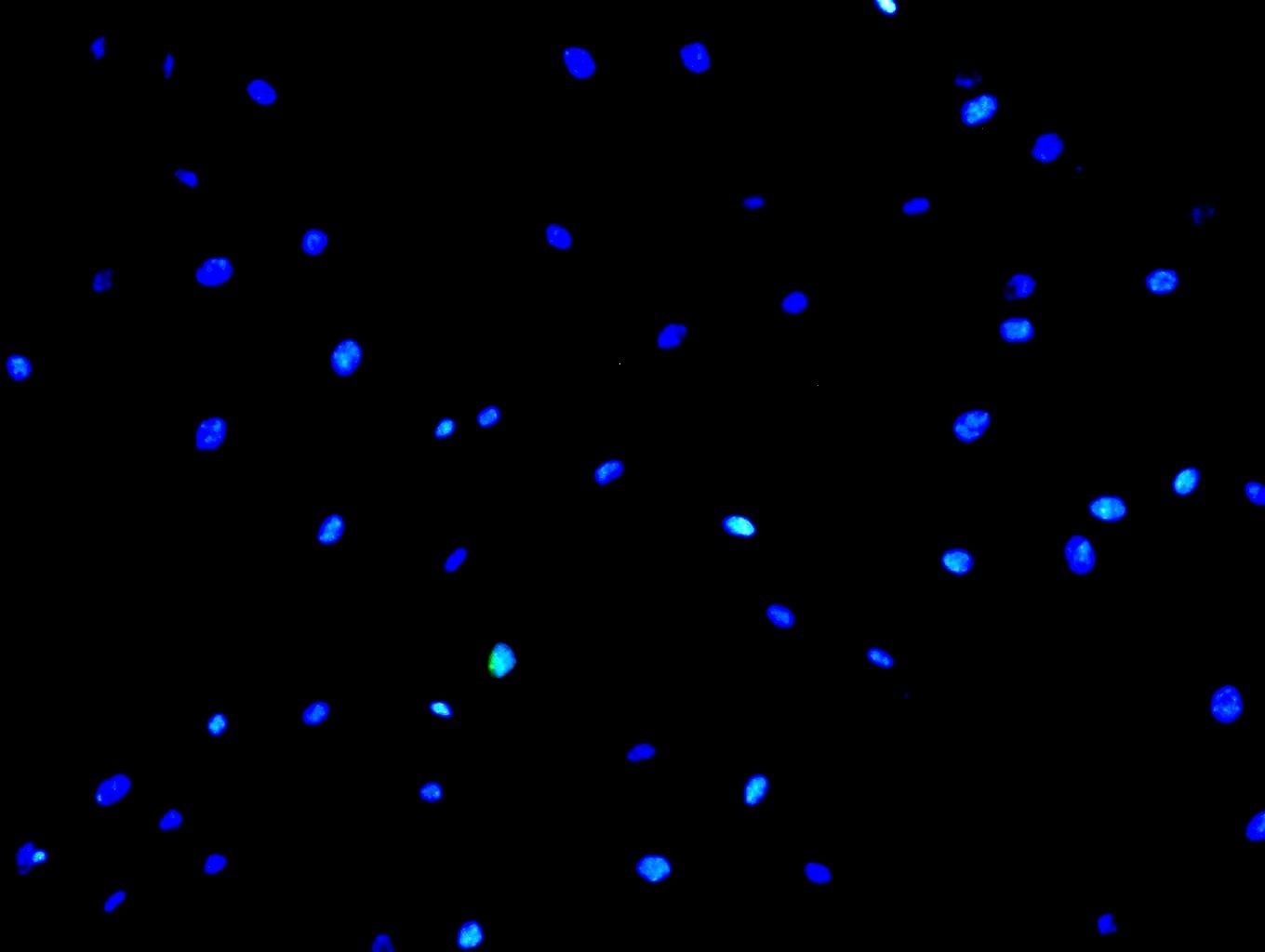

Supplement: Supplementary file 3 — Source data Fig. 1 [file 44318_2024_220_MOESM3_ESM.zip › Figure1/1E/Image/Day0-1 (3).jpg]

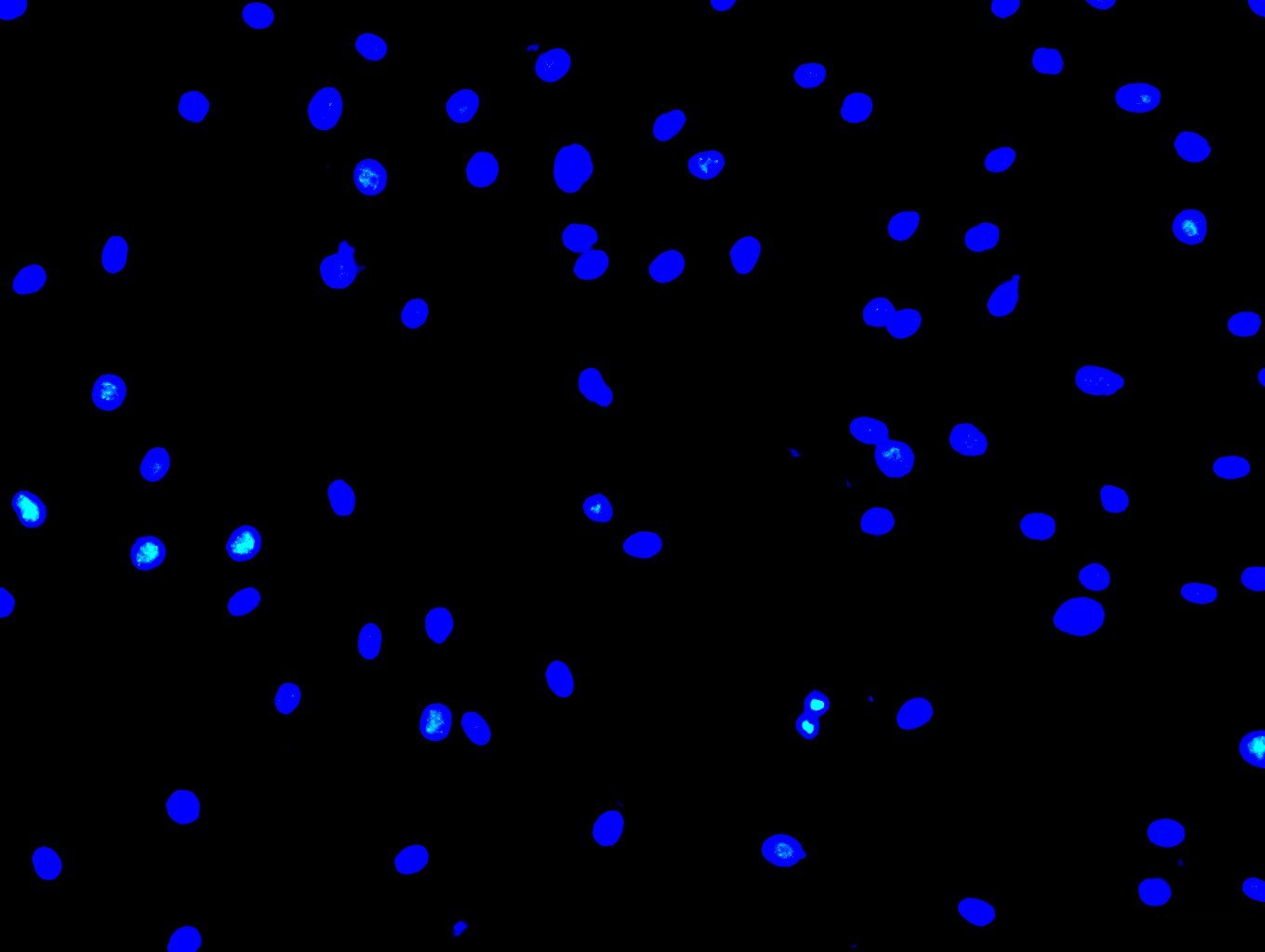

Supplement: Supplementary file 3 — Source data Fig. 1 [file 44318_2024_220_MOESM3_ESM.zip › Figure1/1E/Image/Day0-2 (1).jpg]

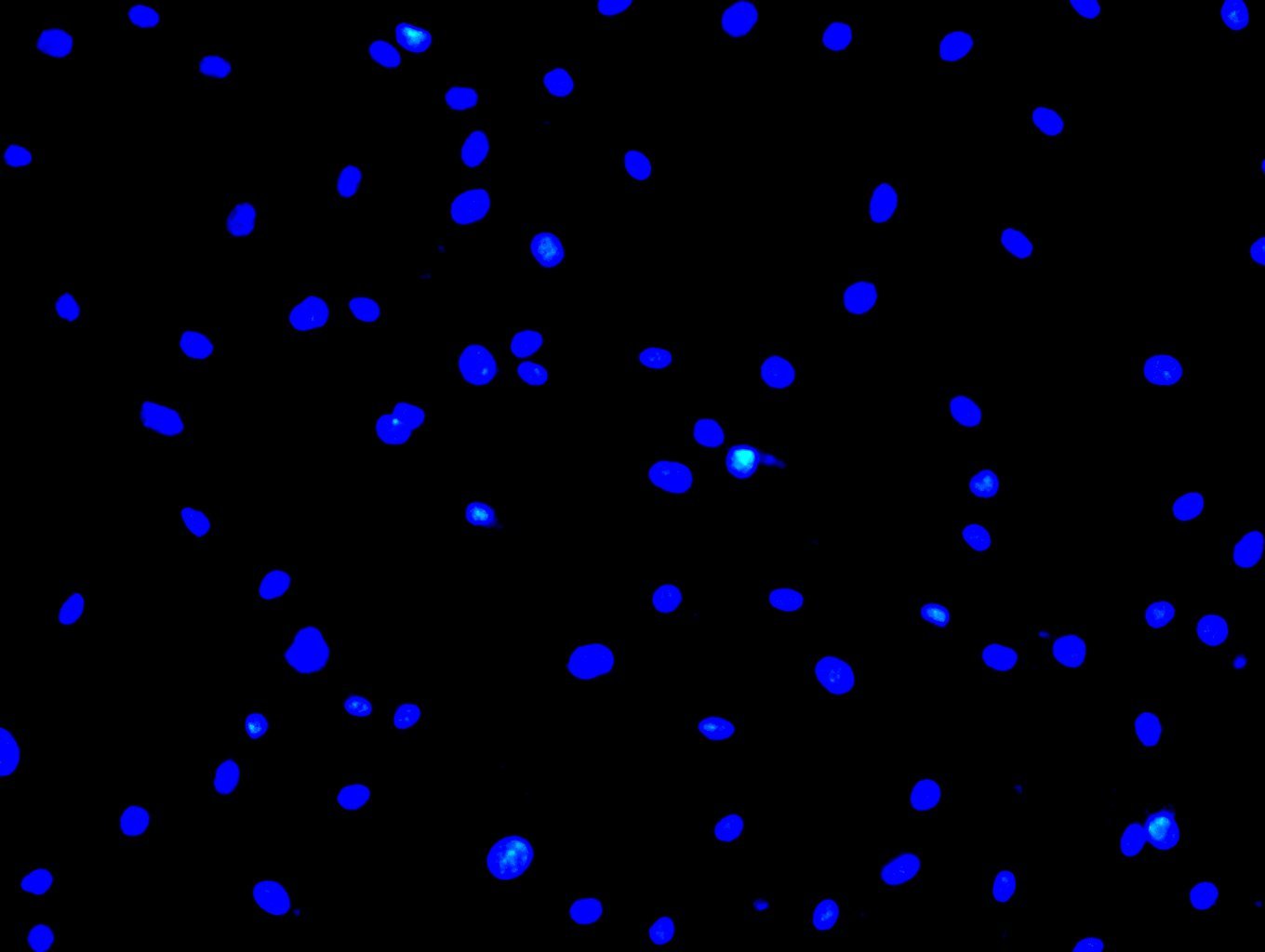

Supplement: Supplementary file 3 — Source data Fig. 1 [file 44318_2024_220_MOESM3_ESM.zip › Figure1/1E/Image/Day0-2 (2).jpg]

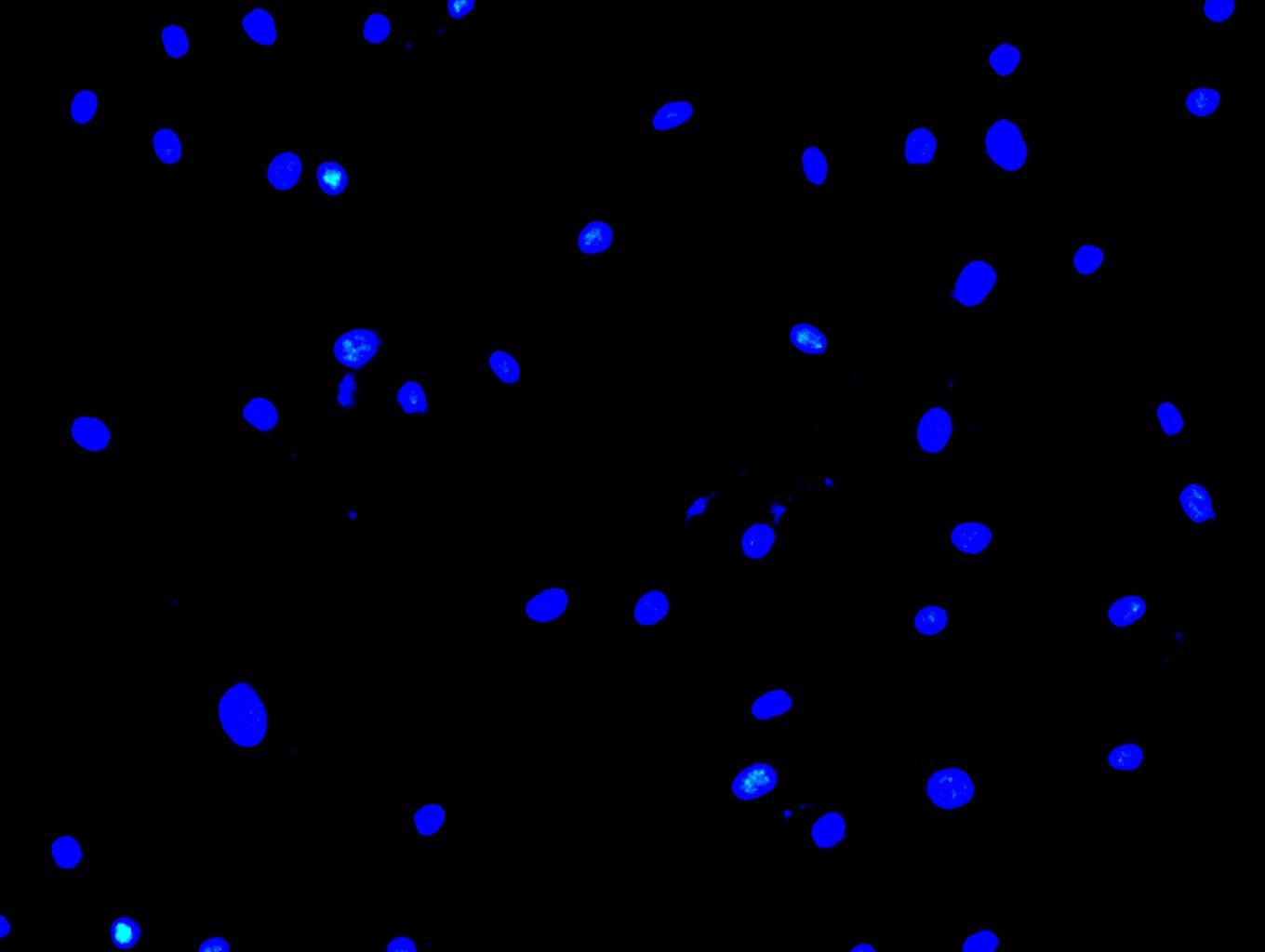

Supplement: Supplementary file 3 — Source data Fig. 1 [file 44318_2024_220_MOESM3_ESM.zip › Figure1/1E/Image/Day0-2 (3).jpg]

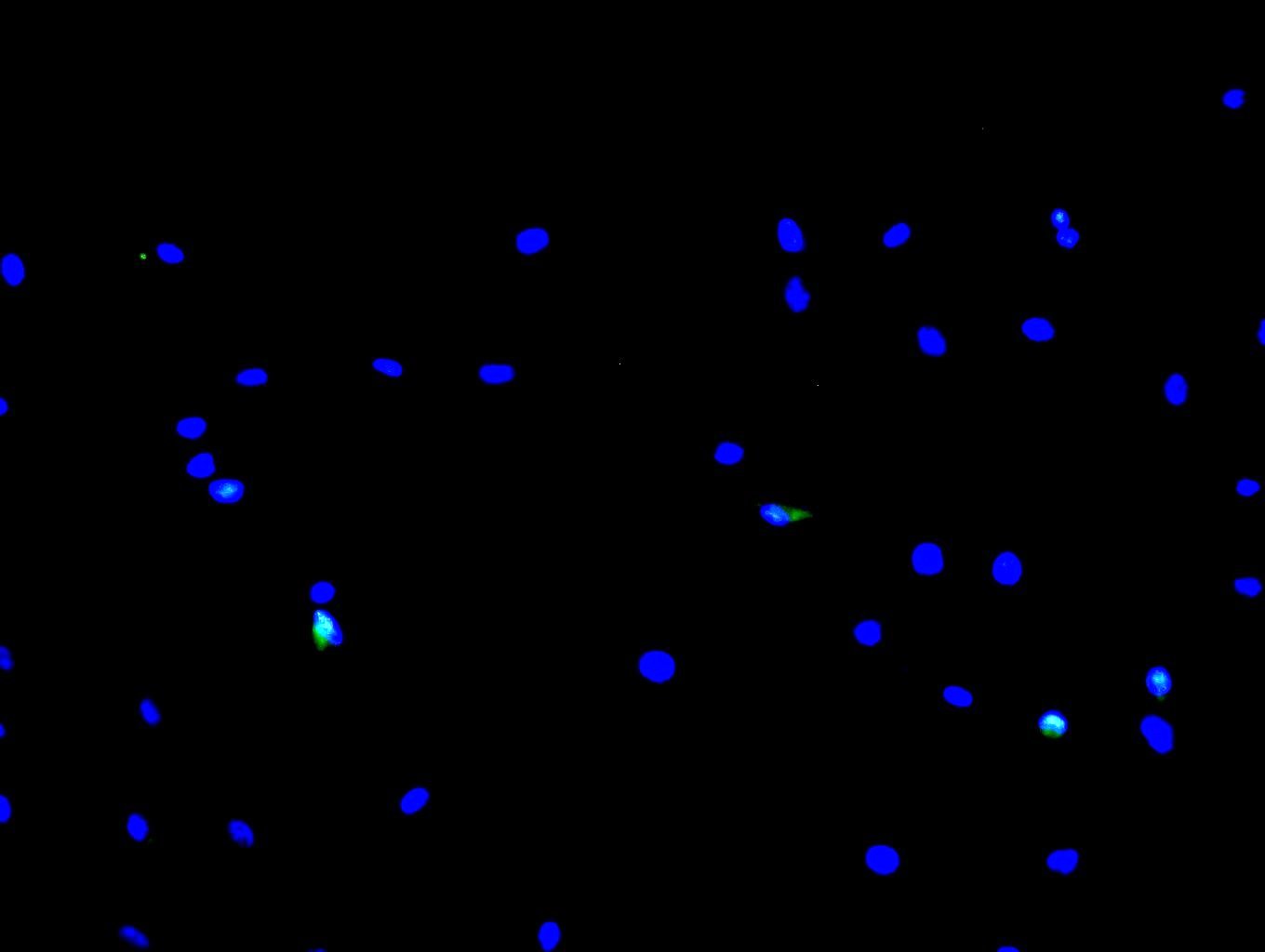

Supplement: Supplementary file 3 — Source data Fig. 1 [file 44318_2024_220_MOESM3_ESM.zip › Figure1/1E/Image/Day0-3 (1).jpg]

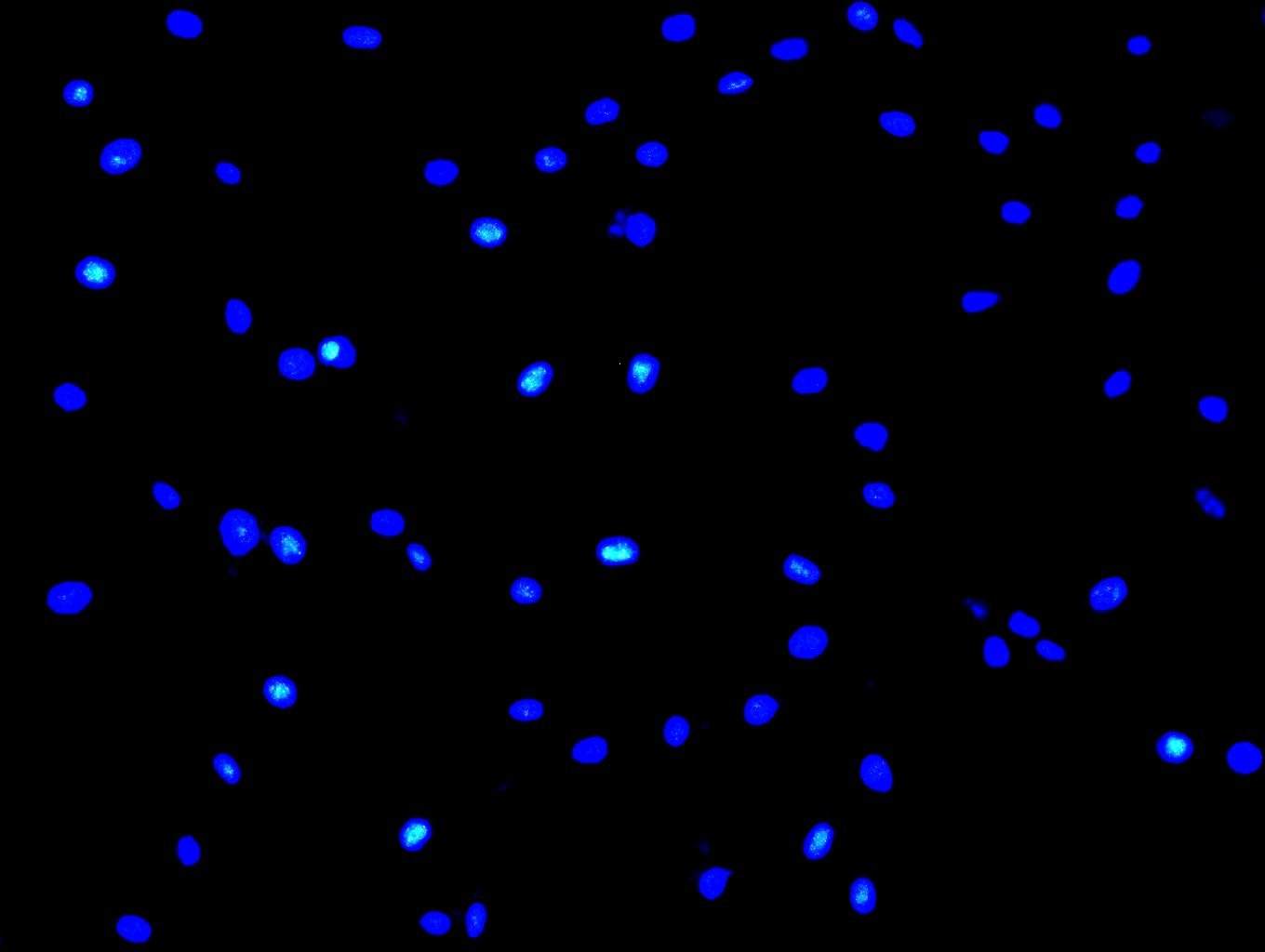

Supplement: Supplementary file 3 — Source data Fig. 1 [file 44318_2024_220_MOESM3_ESM.zip › Figure1/1E/Image/Day0-3 (2).jpg]

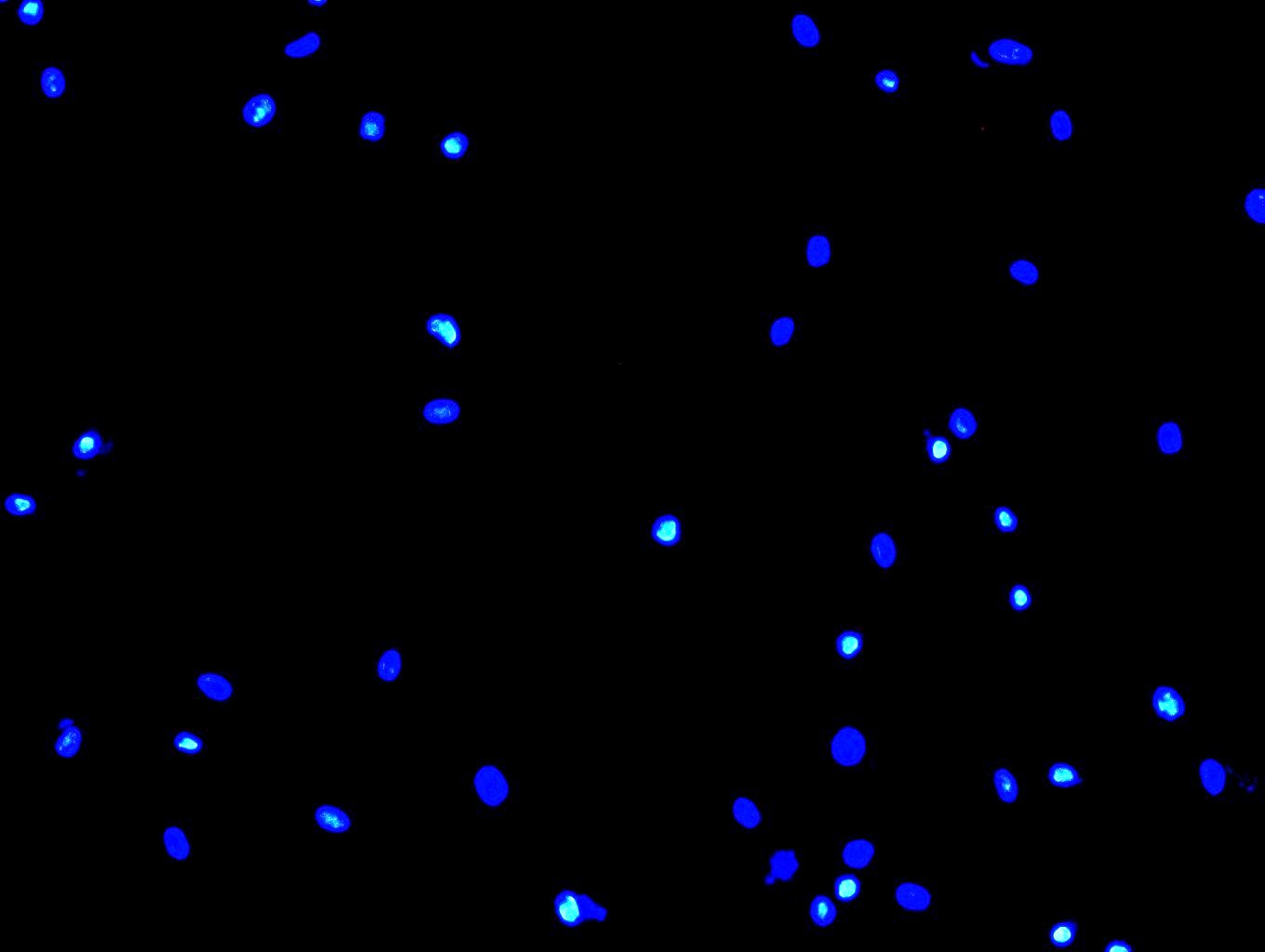

Supplement: Supplementary file 3 — Source data Fig. 1 [file 44318_2024_220_MOESM3_ESM.zip › Figure1/1E/Image/Day0-3 (3).jpg]

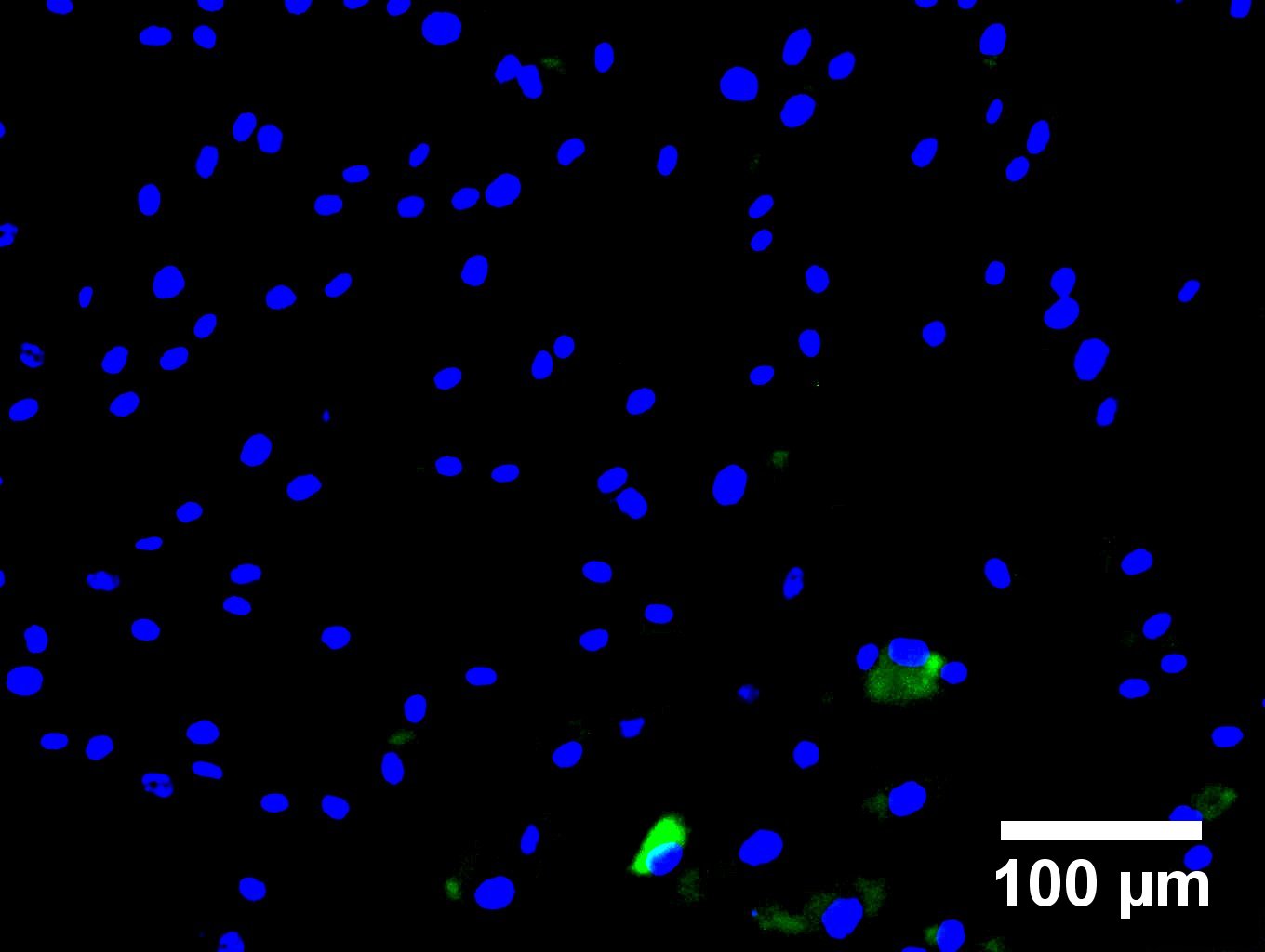

Supplement: Supplementary file 3 — Source data Fig. 1 [file 44318_2024_220_MOESM3_ESM.zip › Figure1/1E/Image/Day2-1(1).jpg]

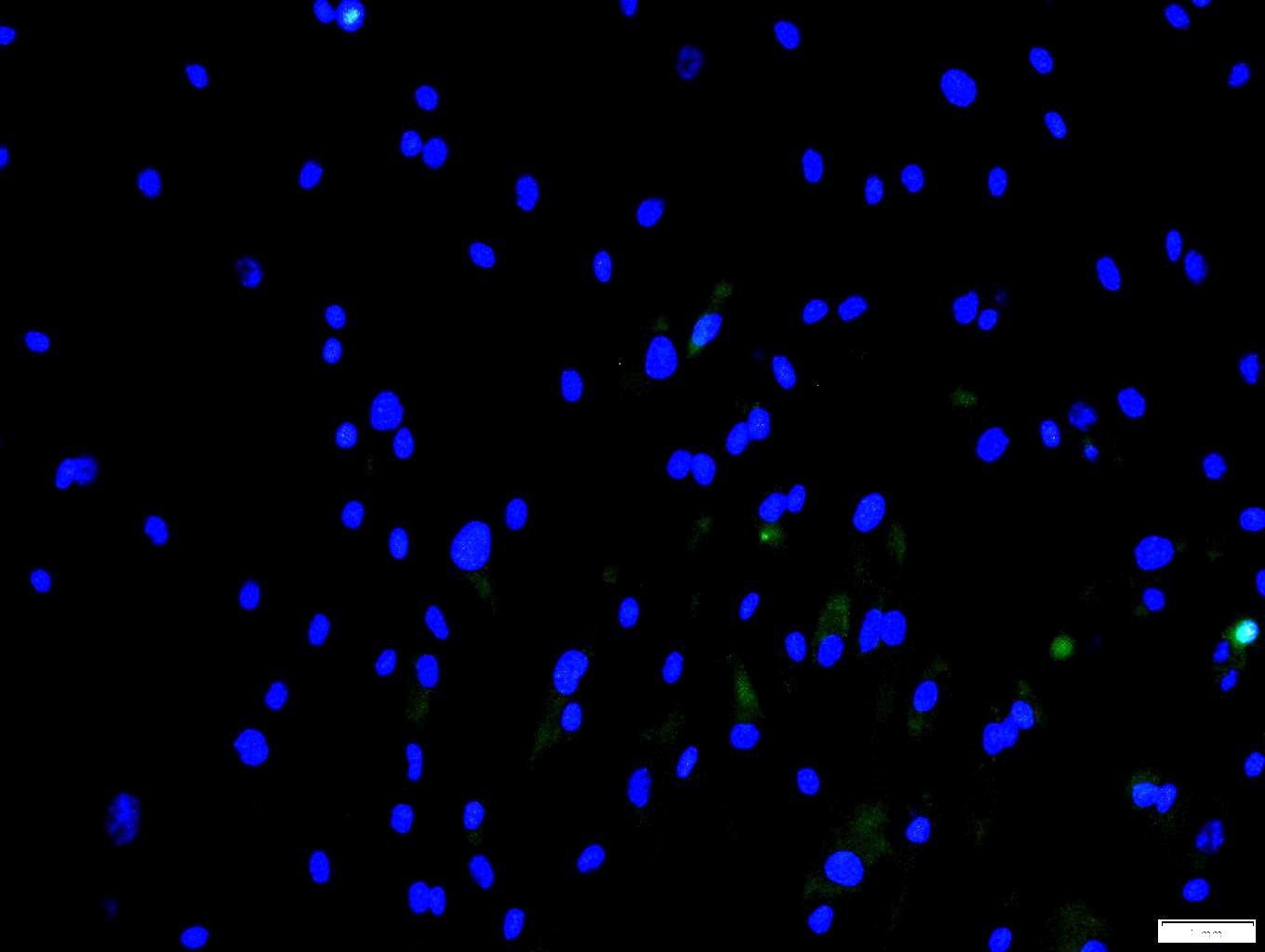

Supplement: Supplementary file 3 — Source data Fig. 1 [file 44318_2024_220_MOESM3_ESM.zip › Figure1/1E/Image/Day2-1(2).jpg]

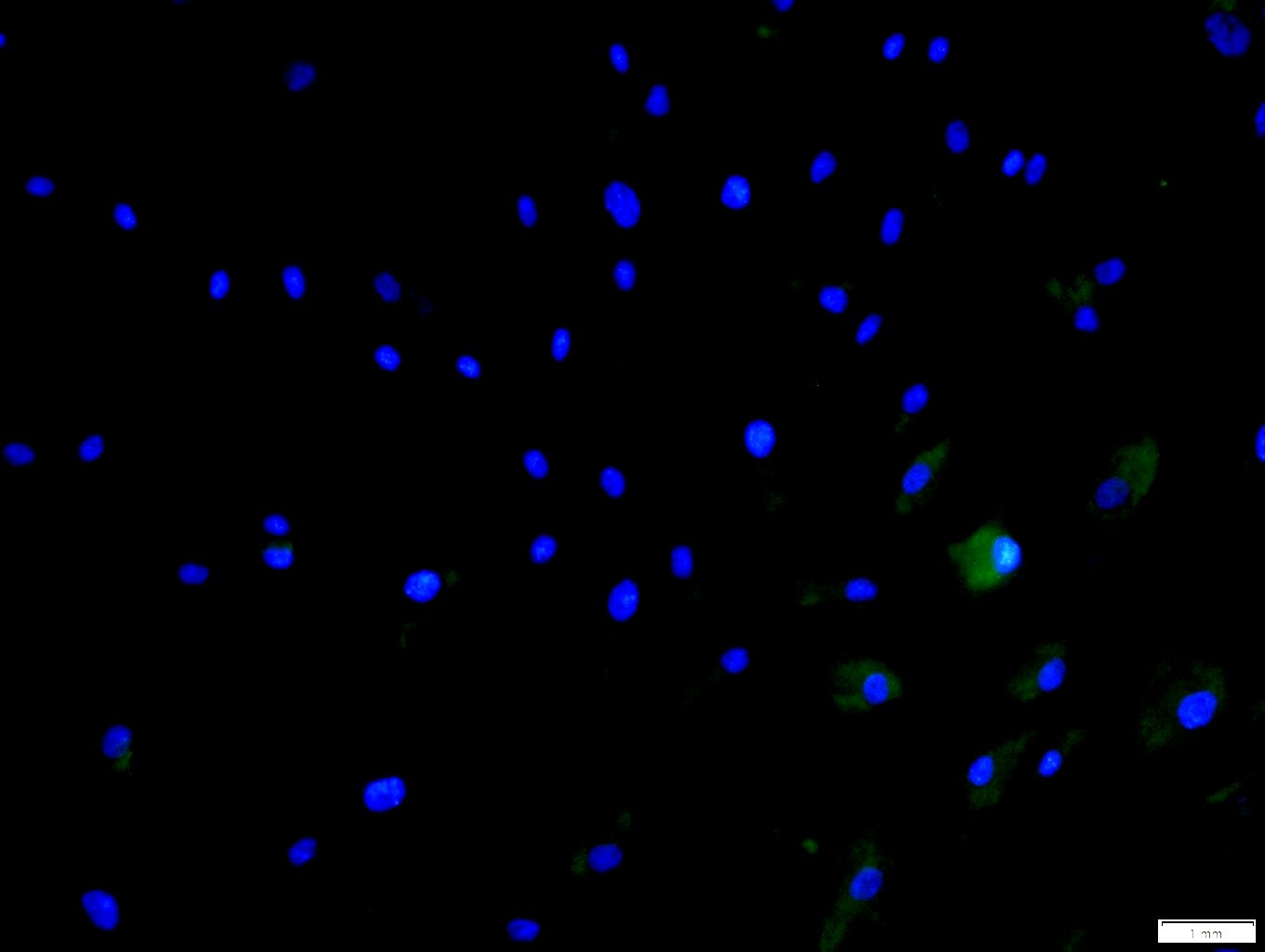

Supplement: Supplementary file 3 — Source data Fig. 1 [file 44318_2024_220_MOESM3_ESM.zip › Figure1/1E/Image/Day2-1(3).jpg]

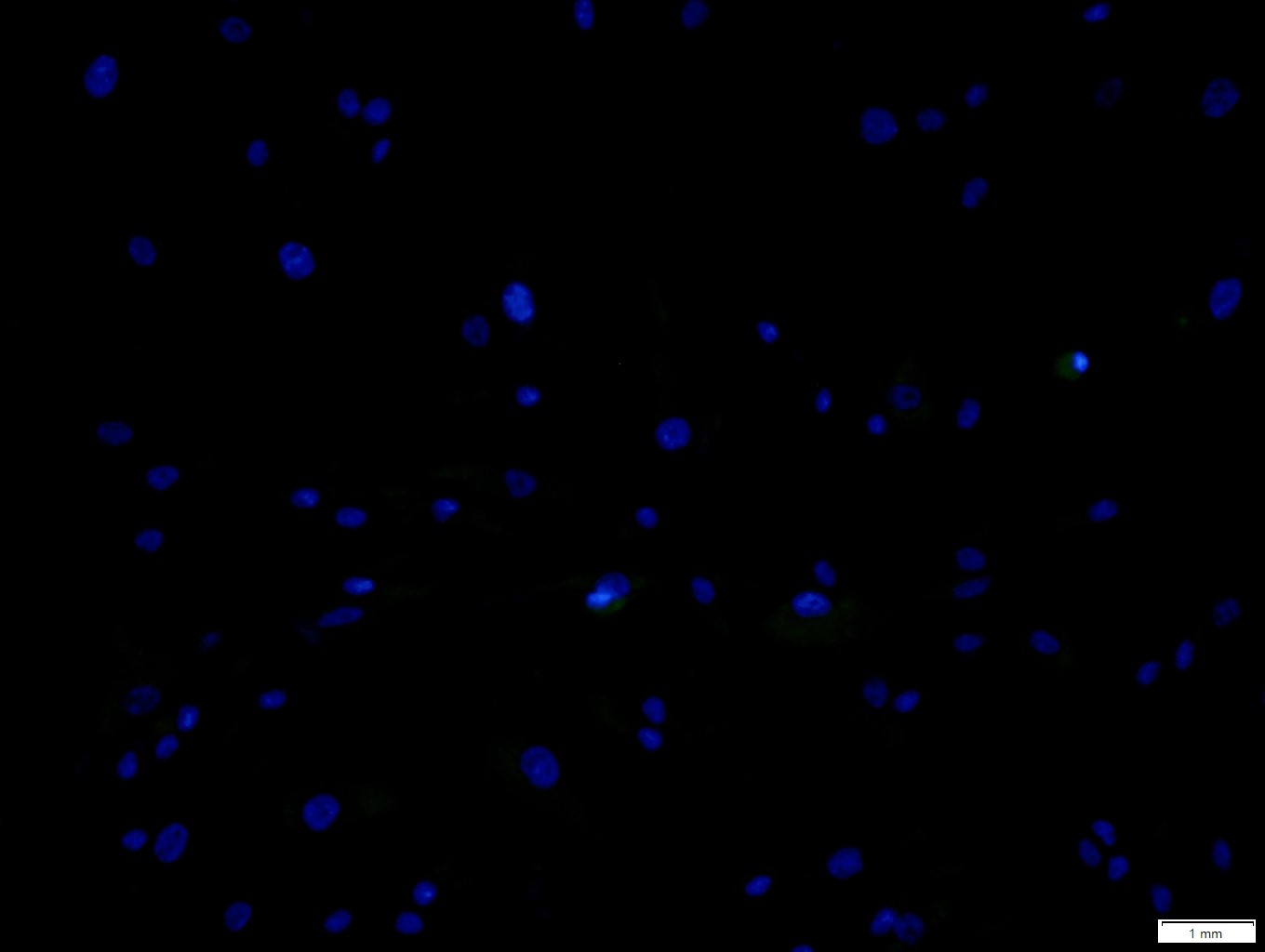

Supplement: Supplementary file 3 — Source data Fig. 1 [file 44318_2024_220_MOESM3_ESM.zip › Figure1/1E/Image/Day2-2 (1).jpg]

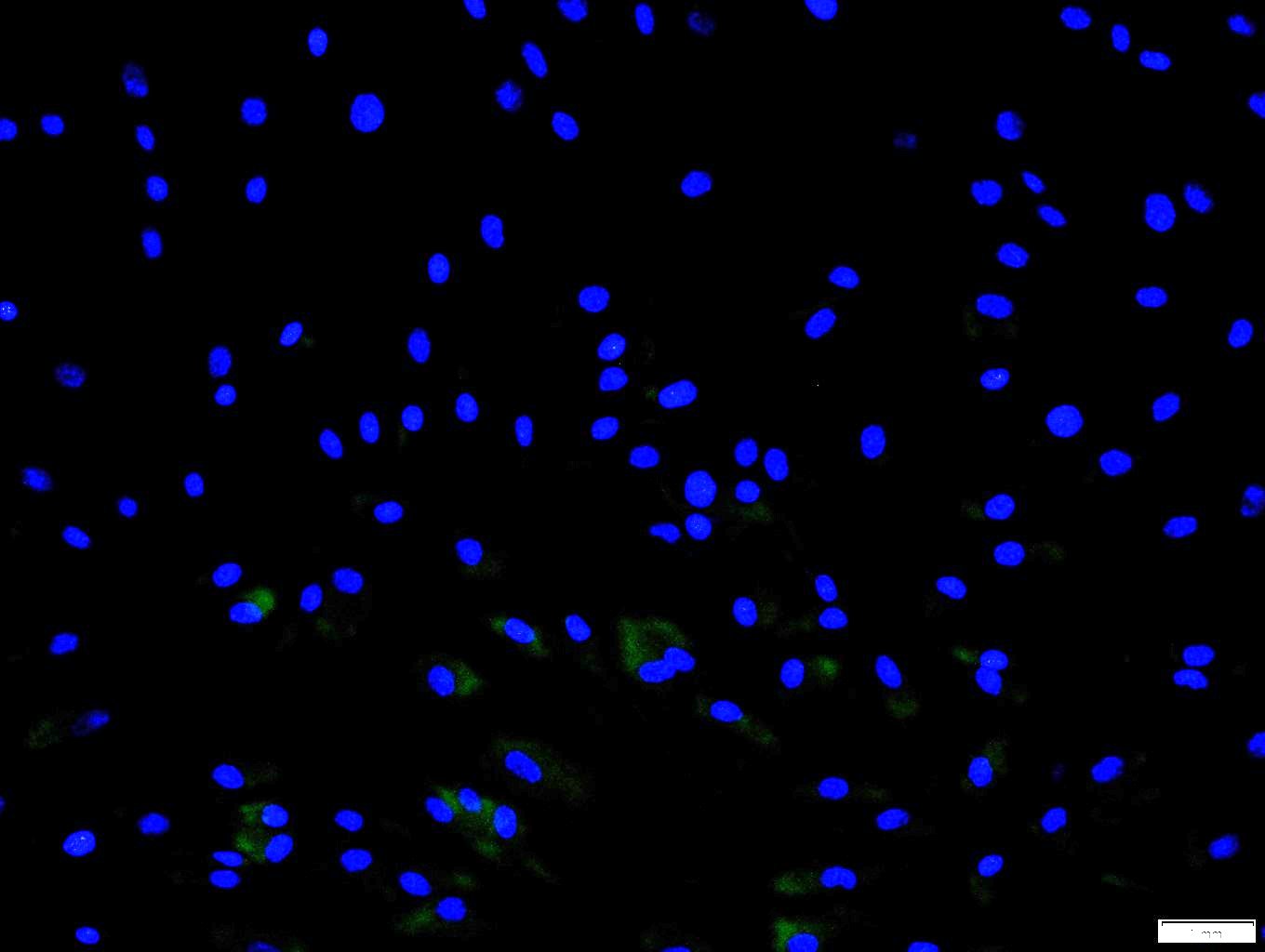

Supplement: Supplementary file 3 — Source data Fig. 1 [file 44318_2024_220_MOESM3_ESM.zip › Figure1/1E/Image/Day2-2 (2).jpg]

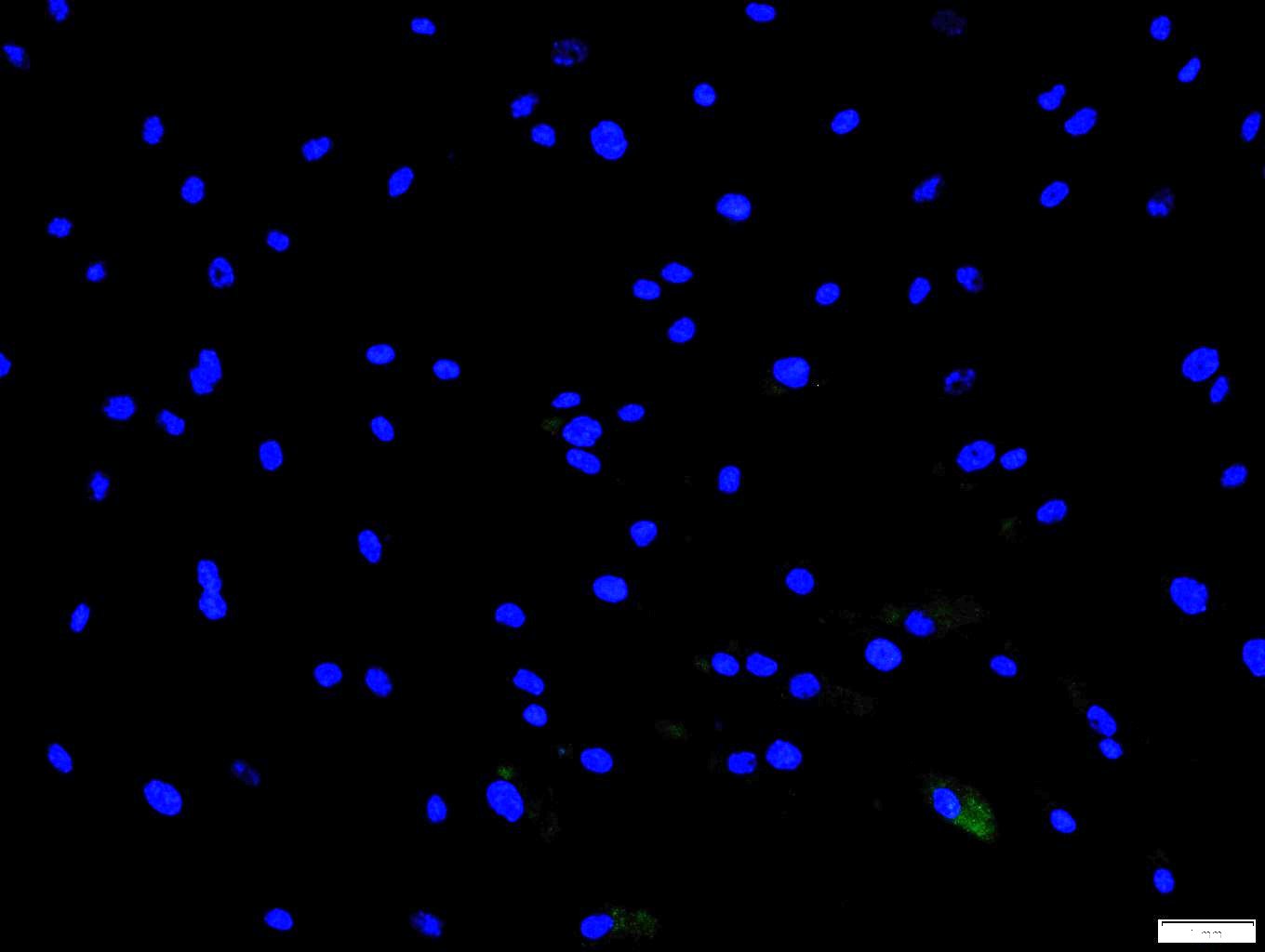

Supplement: Supplementary file 3 — Source data Fig. 1 [file 44318_2024_220_MOESM3_ESM.zip › Figure1/1E/Image/Day2-2 (3).jpg]

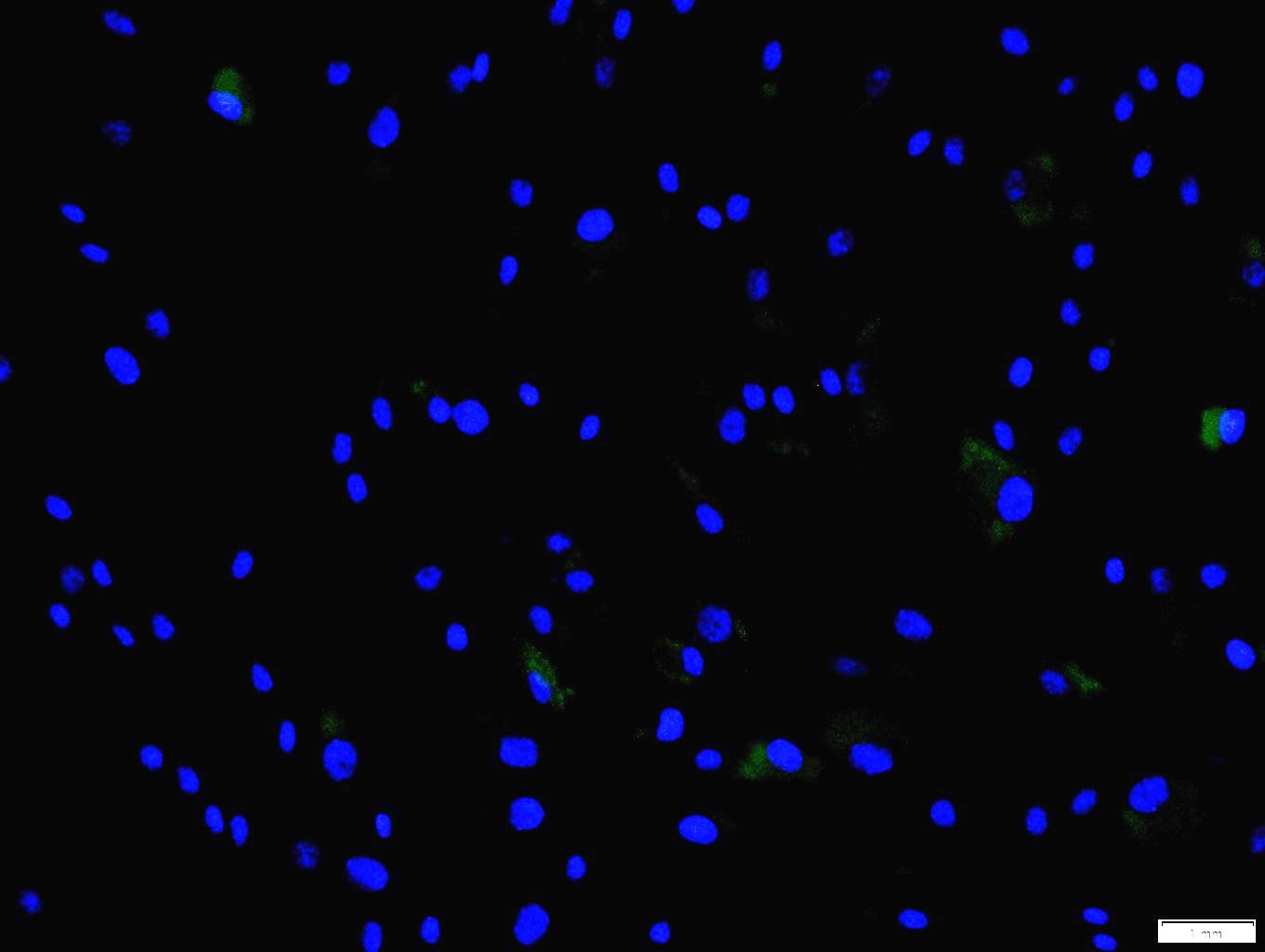

Supplement: Supplementary file 3 — Source data Fig. 1 [file 44318_2024_220_MOESM3_ESM.zip › Figure1/1E/Image/Day2-3 (1).jpg]

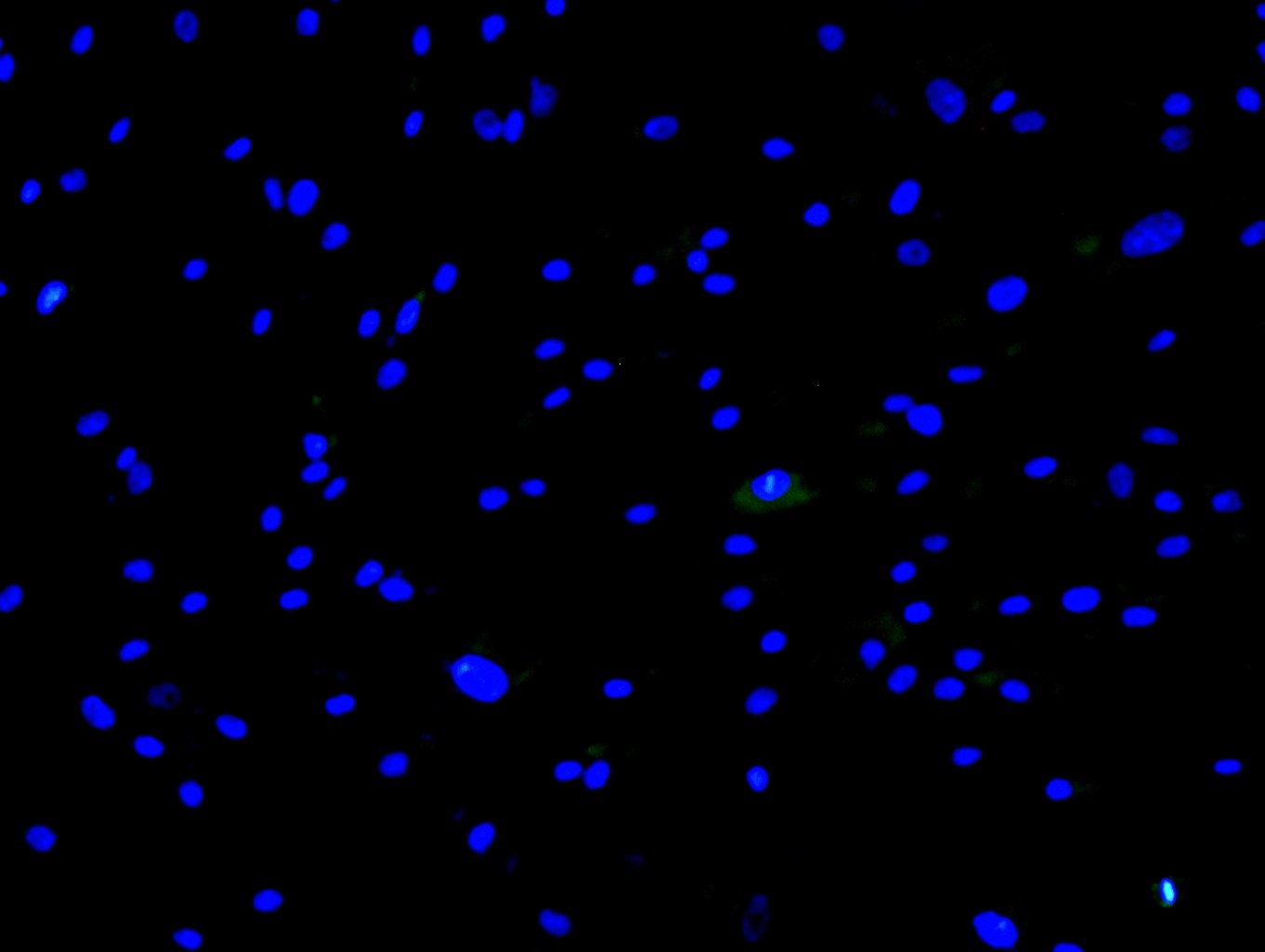

Supplement: Supplementary file 3 — Source data Fig. 1 [file 44318_2024_220_MOESM3_ESM.zip › Figure1/1E/Image/Day2-3 (2).jpg]

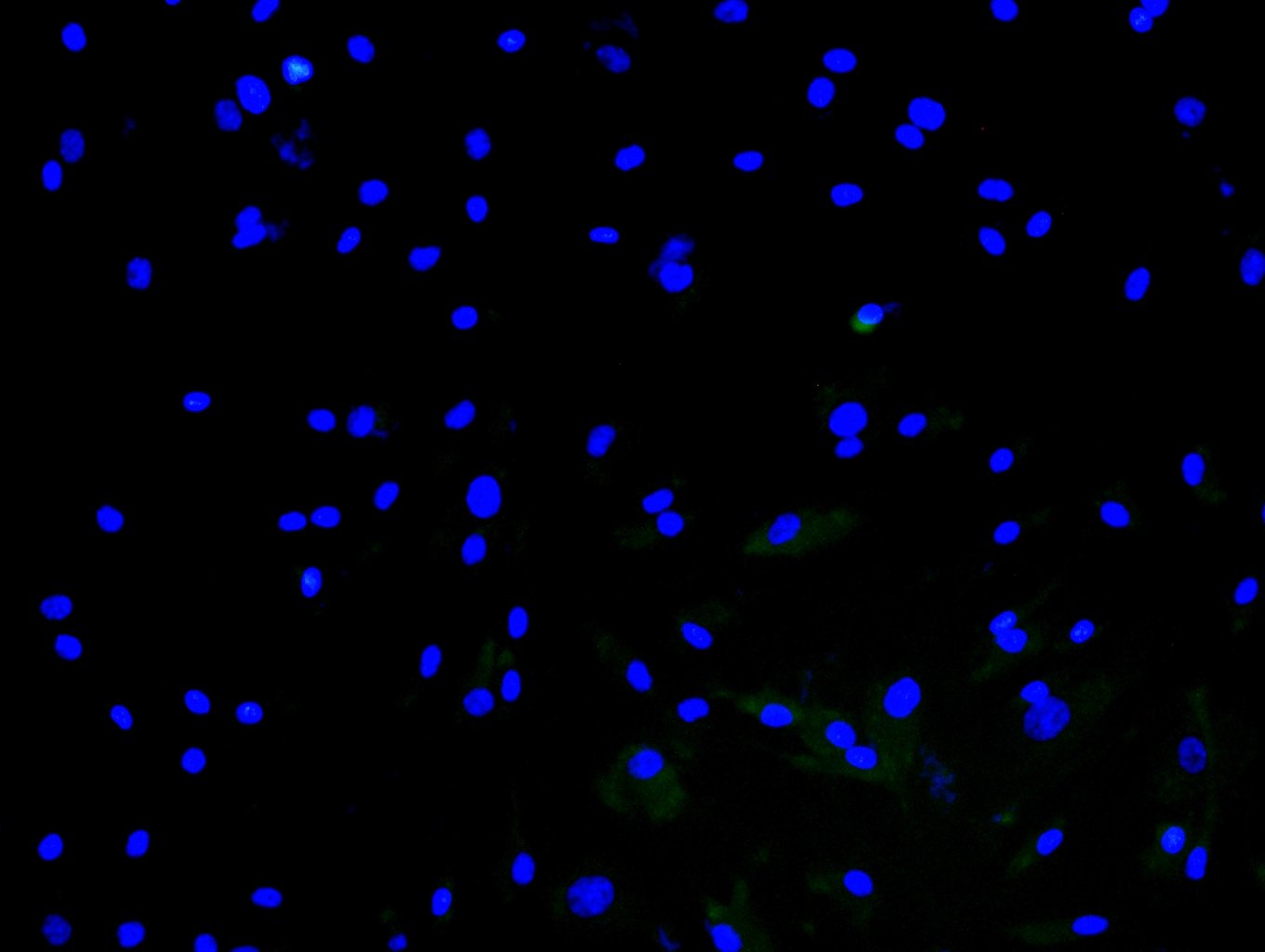

Supplement: Supplementary file 3 — Source data Fig. 1 [file 44318_2024_220_MOESM3_ESM.zip › Figure1/1E/Image/Day2-3 (3).jpg]

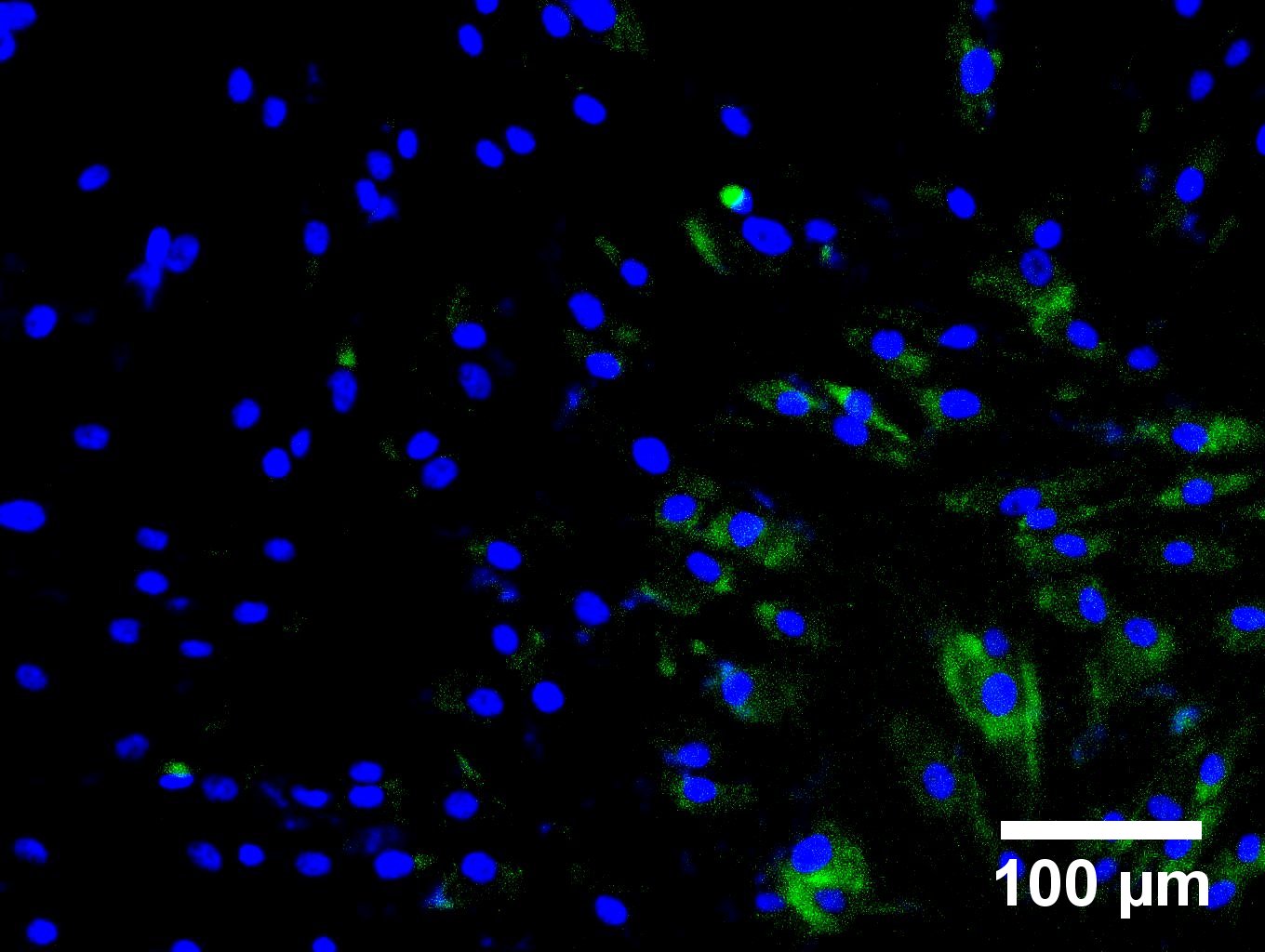

Supplement: Supplementary file 3 — Source data Fig. 1 [file 44318_2024_220_MOESM3_ESM.zip › Figure1/1E/Image/Day4-1(1).jpg]

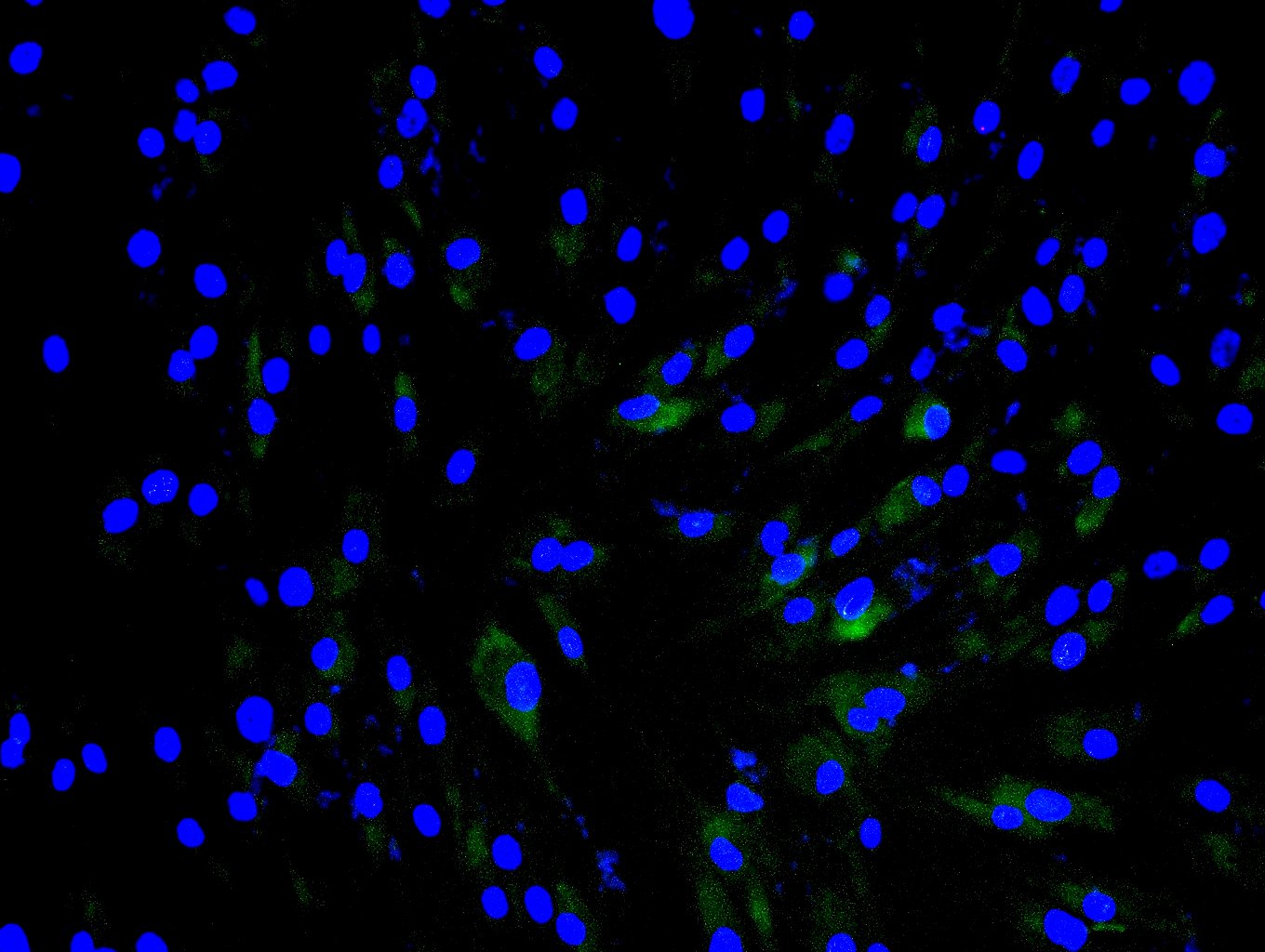

Supplement: Supplementary file 3 — Source data Fig. 1 [file 44318_2024_220_MOESM3_ESM.zip › Figure1/1E/Image/Day4-1(2).jpg]

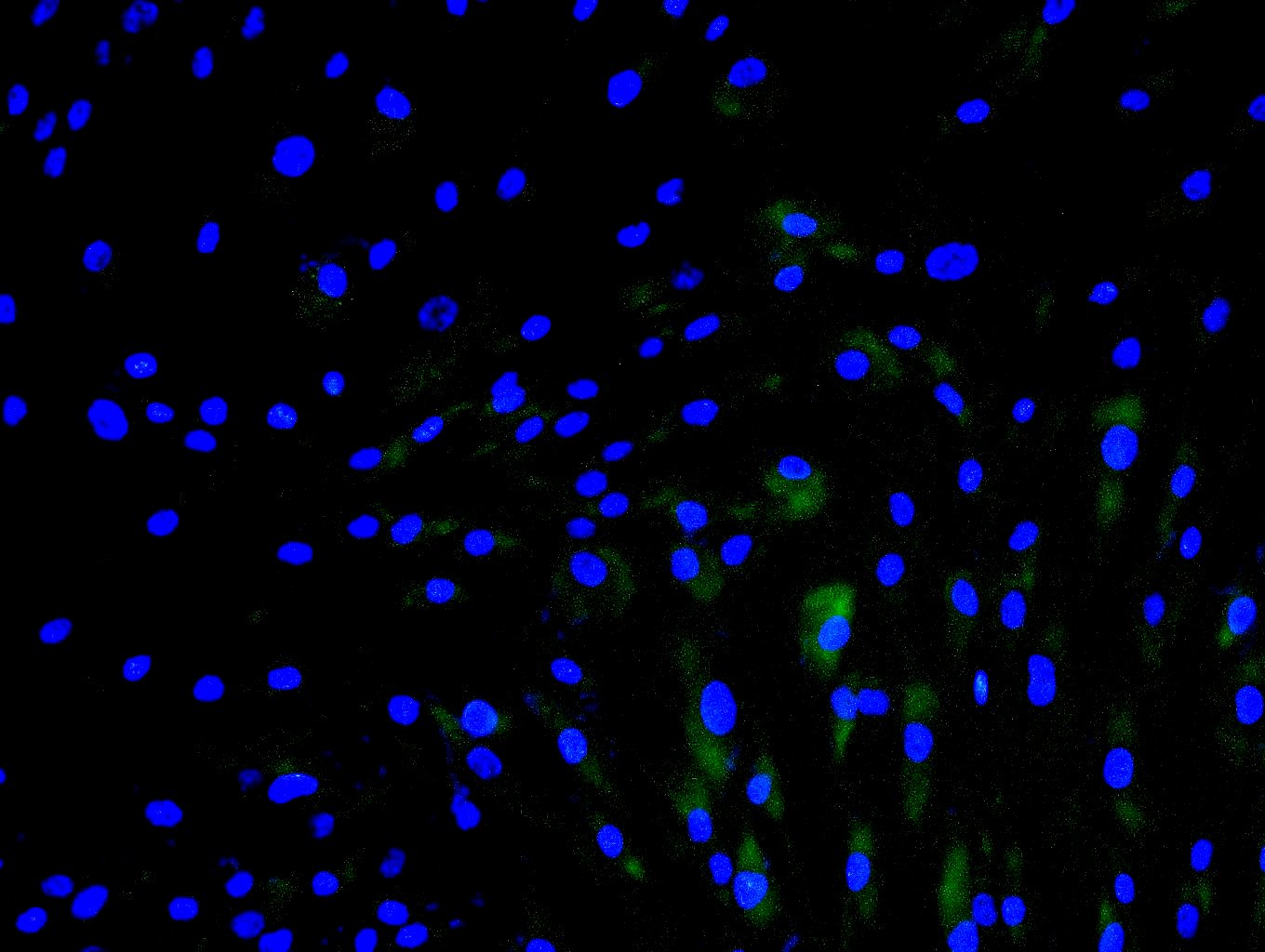

Supplement: Supplementary file 3 — Source data Fig. 1 [file 44318_2024_220_MOESM3_ESM.zip › Figure1/1E/Image/Day4-1(3).jpg]

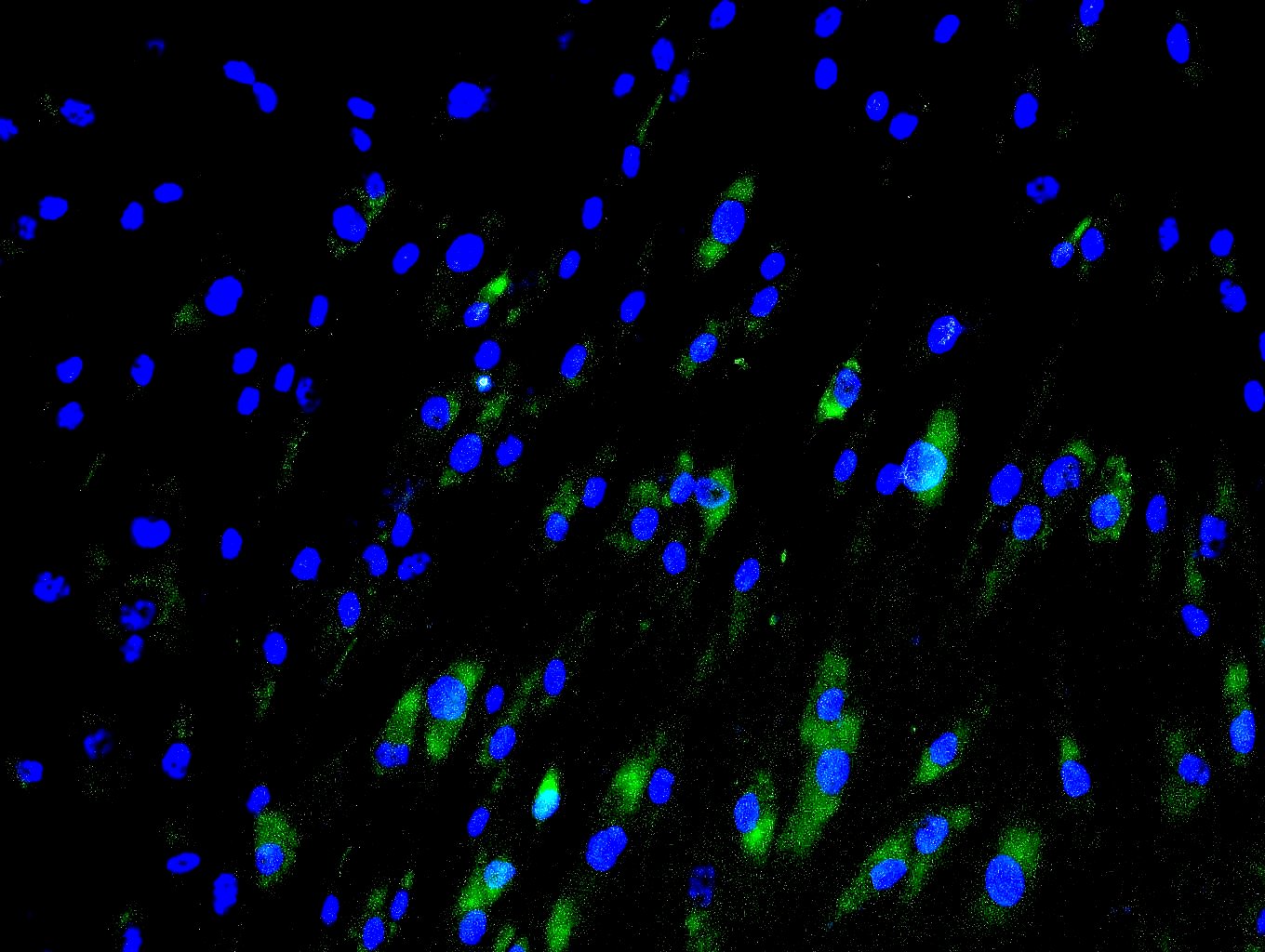

Supplement: Supplementary file 3 — Source data Fig. 1 [file 44318_2024_220_MOESM3_ESM.zip › Figure1/1E/Image/Day4-2(1).jpg]

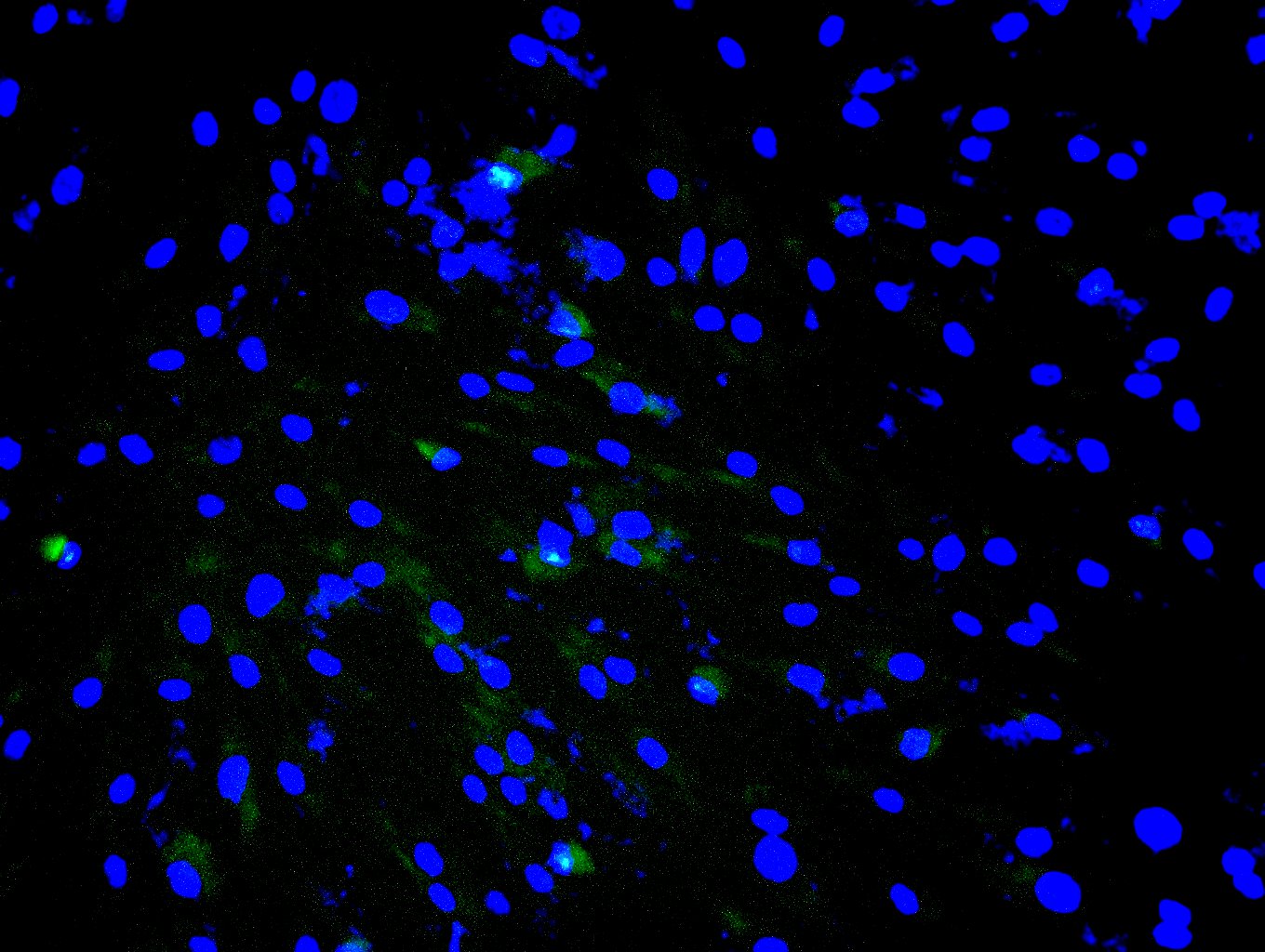

Supplement: Supplementary file 3 — Source data Fig. 1 [file 44318_2024_220_MOESM3_ESM.zip › Figure1/1E/Image/Day4-2(2).jpg]

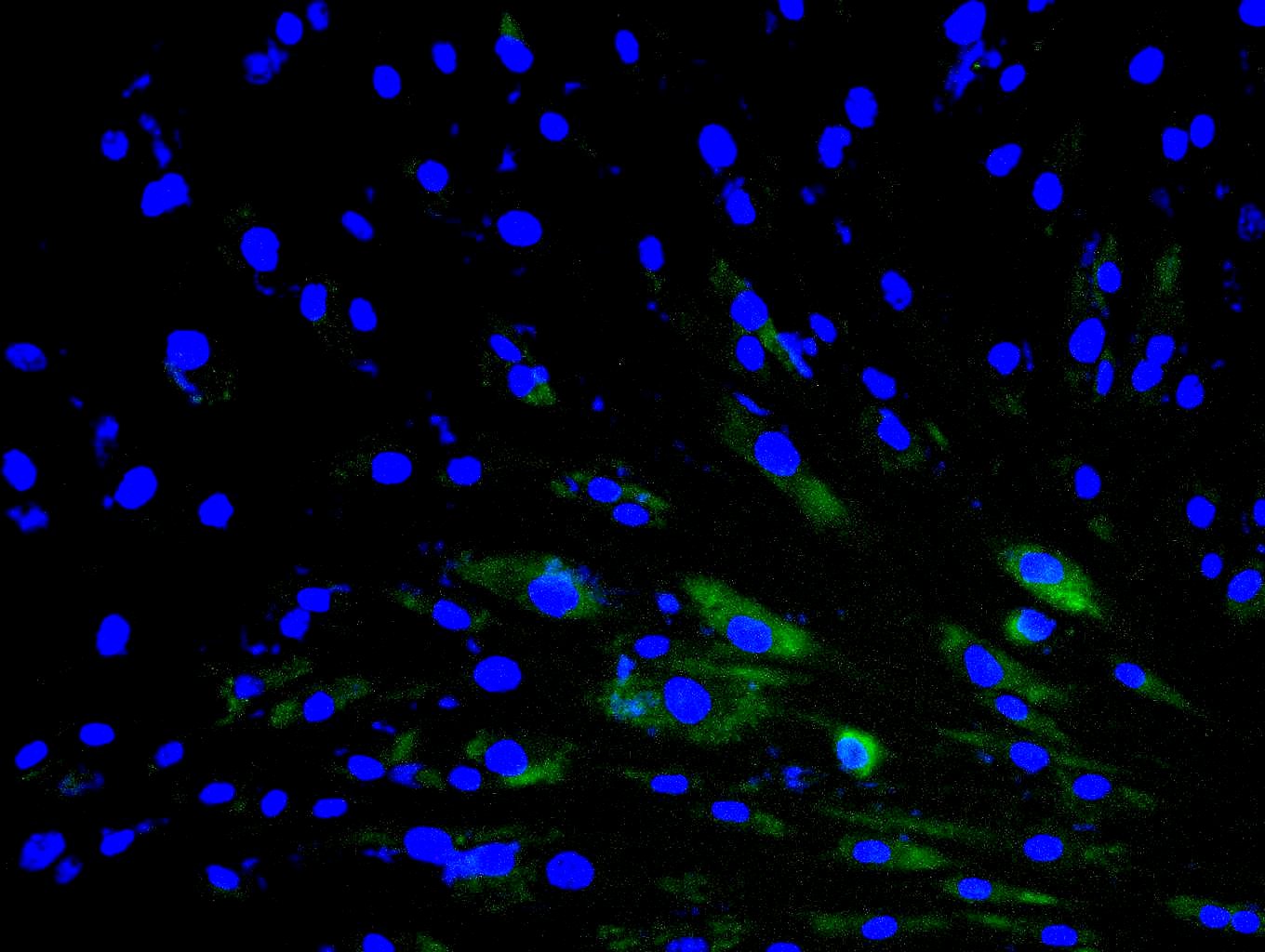

Supplement: Supplementary file 3 — Source data Fig. 1 [file 44318_2024_220_MOESM3_ESM.zip › Figure1/1E/Image/Day4-2(3).jpg]

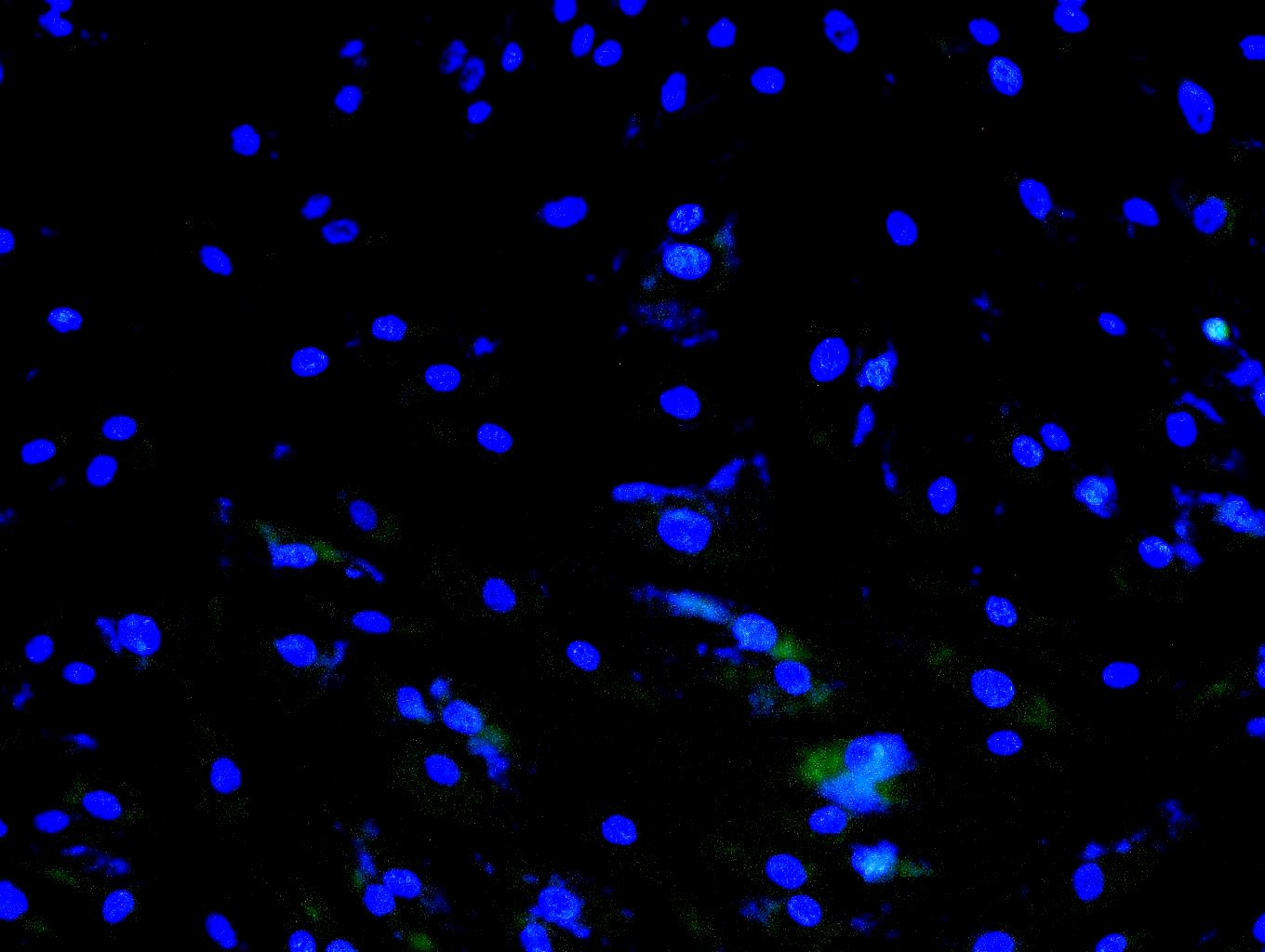

Supplement: Supplementary file 3 — Source data Fig. 1 [file 44318_2024_220_MOESM3_ESM.zip › Figure1/1E/Image/Day4-3 (1).jpg]

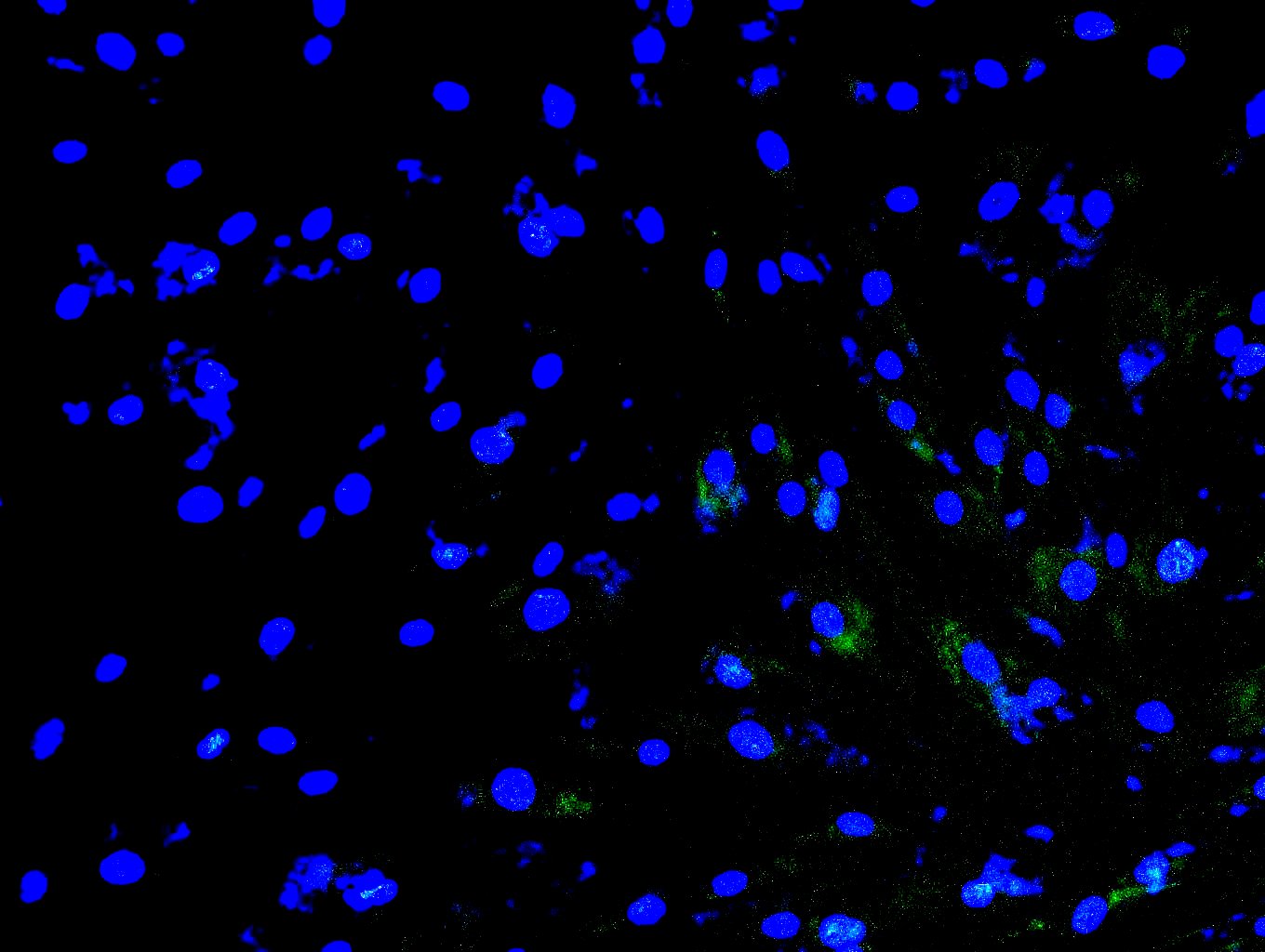

Supplement: Supplementary file 3 — Source data Fig. 1 [file 44318_2024_220_MOESM3_ESM.zip › Figure1/1E/Image/Day4-3 (2).jpg]

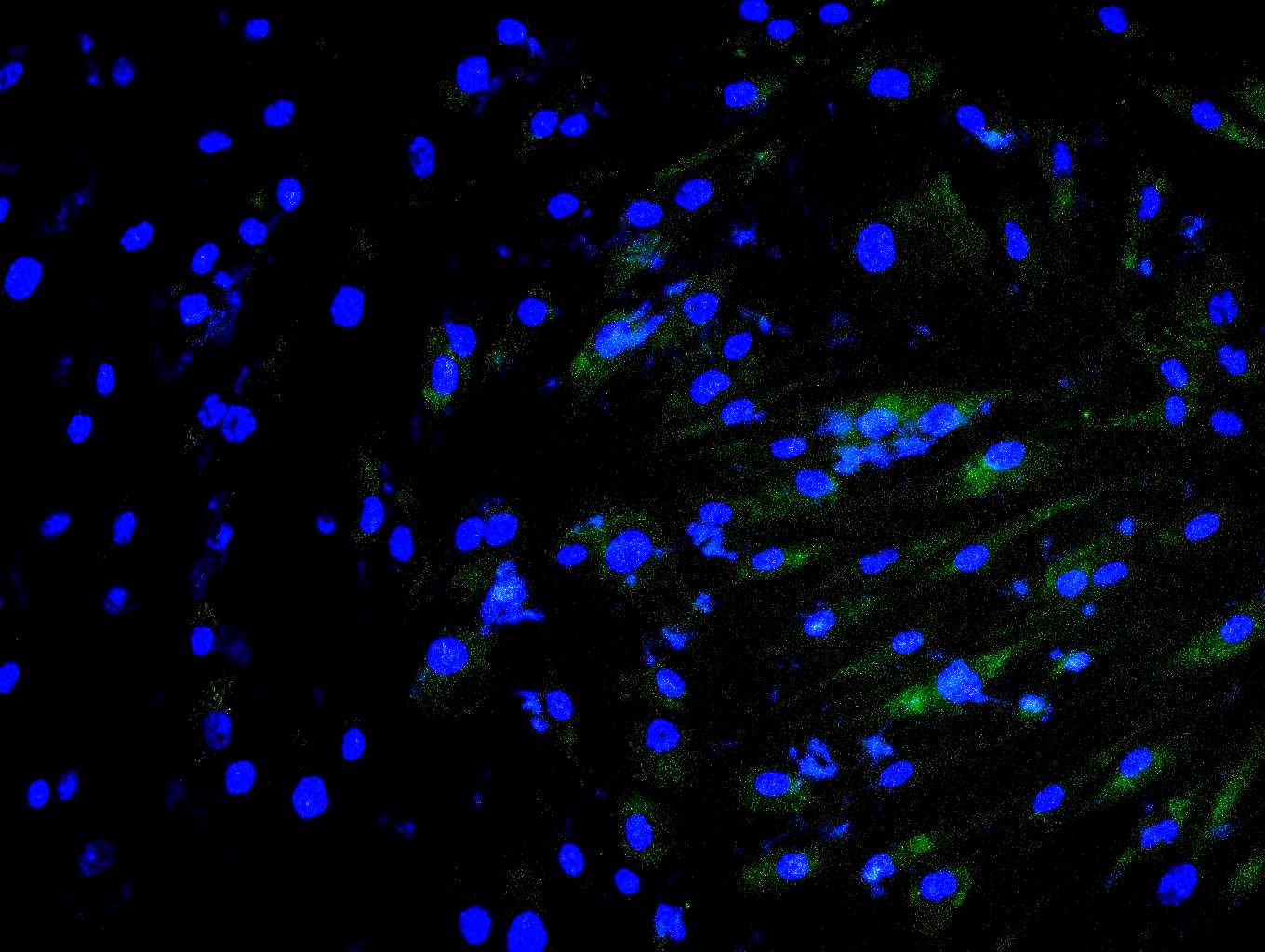

Supplement: Supplementary file 3 — Source data Fig. 1 [file 44318_2024_220_MOESM3_ESM.zip › Figure1/1E/Image/Day4-3 (3).jpg]

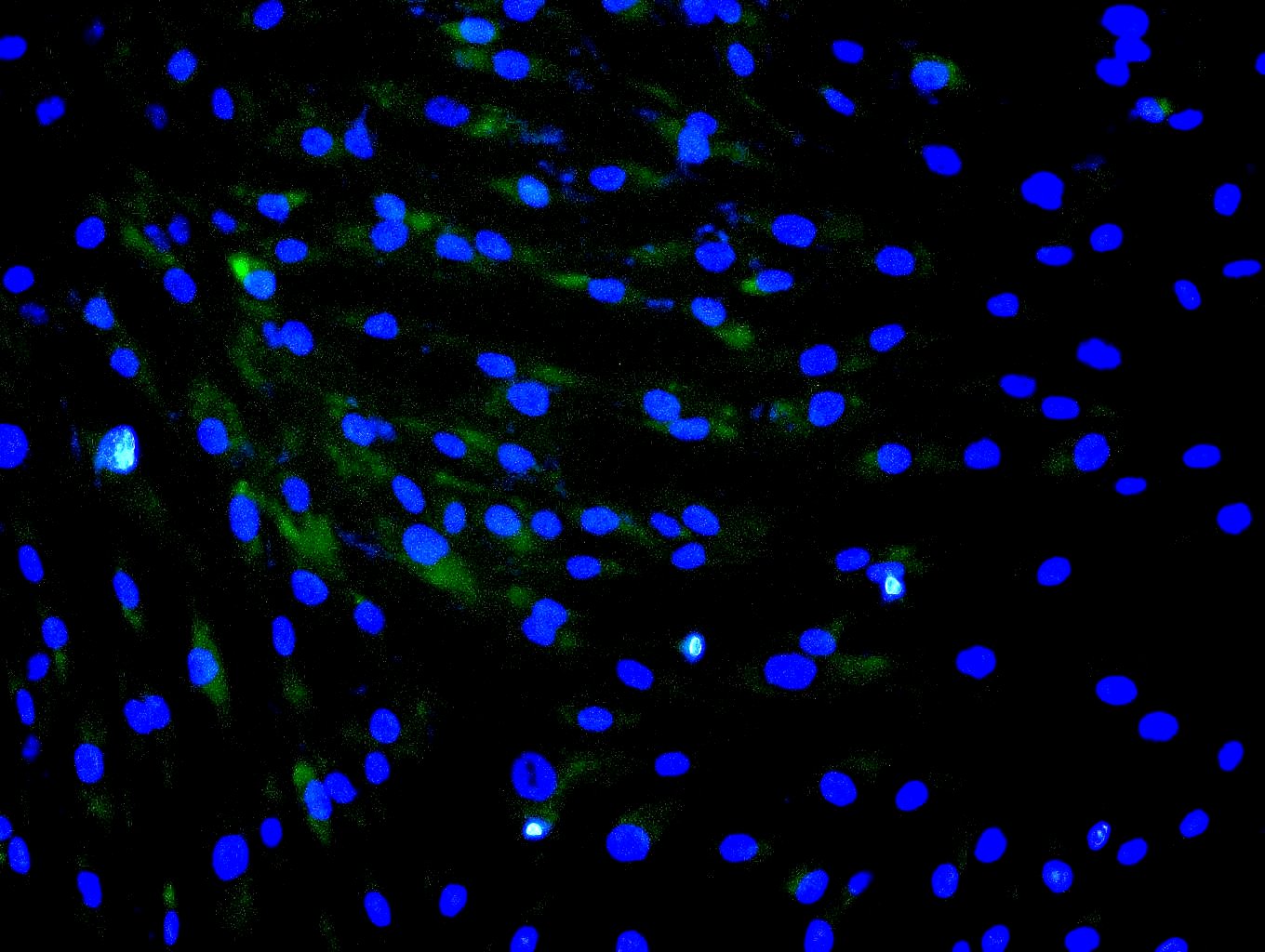

Supplement: Supplementary file 3 — Source data Fig. 1 [file 44318_2024_220_MOESM3_ESM.zip › Figure1/1E/Image/Day6-1(1).jpg]

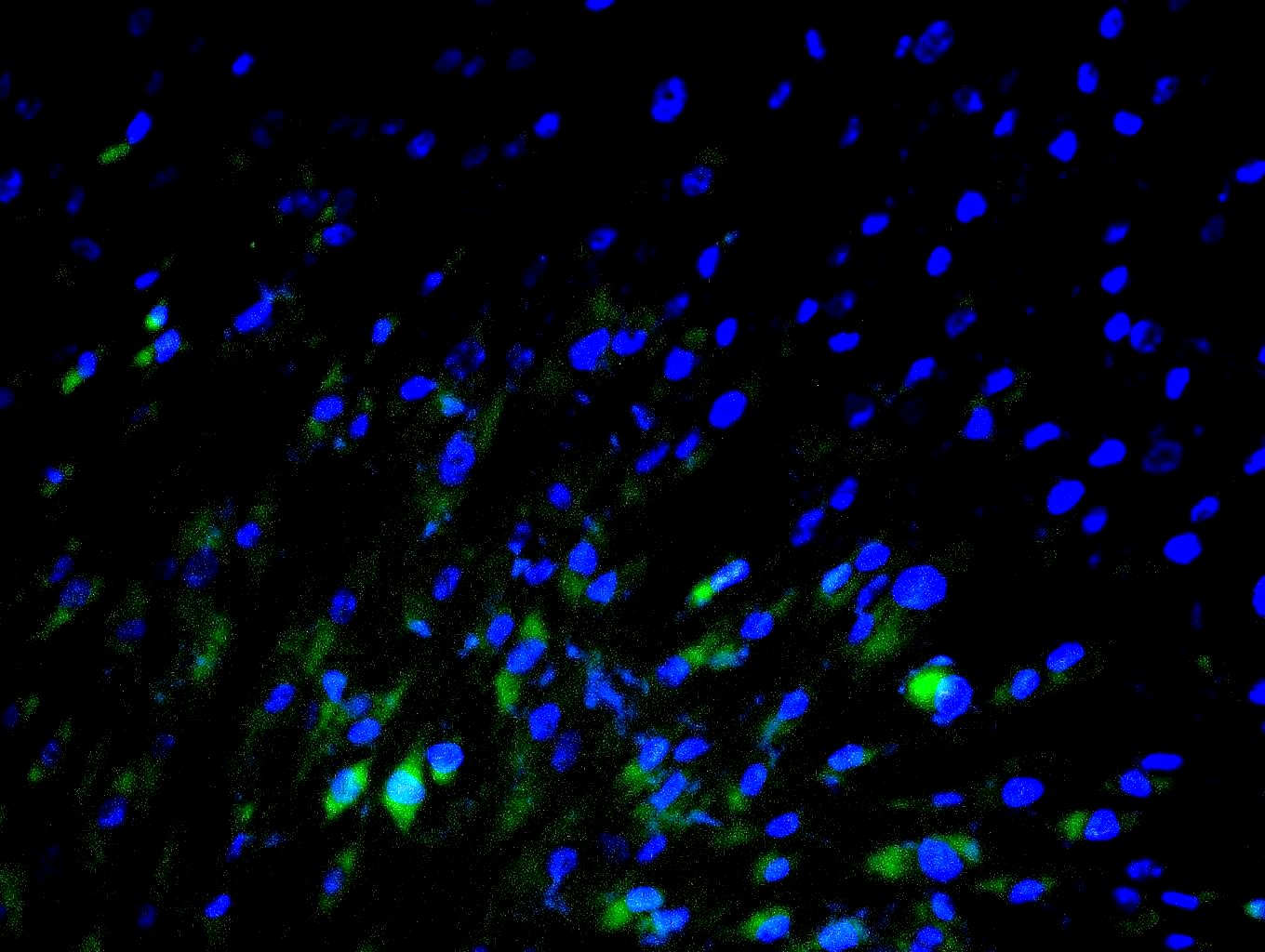

Supplement: Supplementary file 3 — Source data Fig. 1 [file 44318_2024_220_MOESM3_ESM.zip › Figure1/1E/Image/Day6-1(2).jpg]

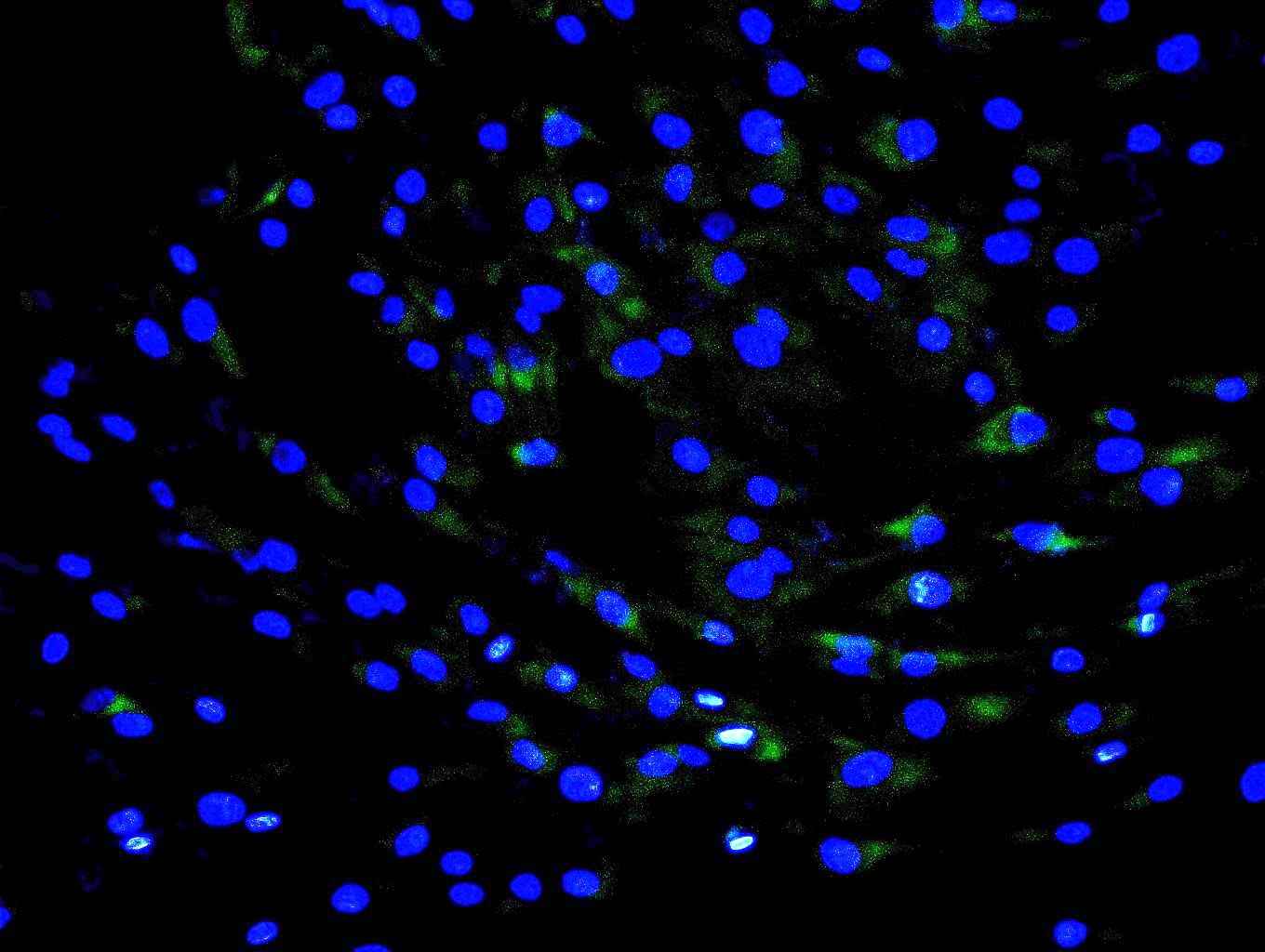

Supplement: Supplementary file 3 — Source data Fig. 1 [file 44318_2024_220_MOESM3_ESM.zip › Figure1/1E/Image/Day6-1(3).jpg]

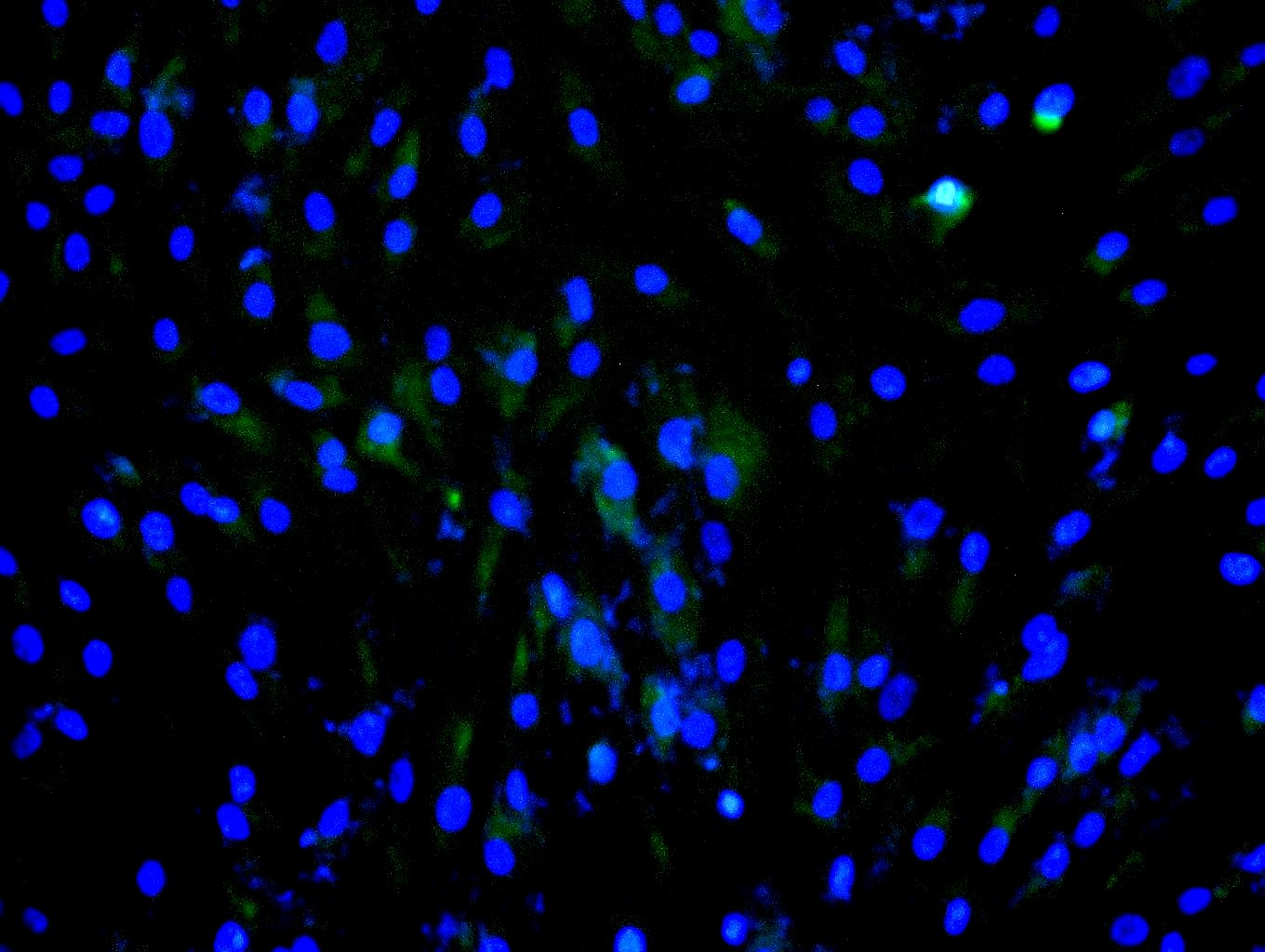

Supplement: Supplementary file 3 — Source data Fig. 1 [file 44318_2024_220_MOESM3_ESM.zip › Figure1/1E/Image/Day6-2(1).jpg]

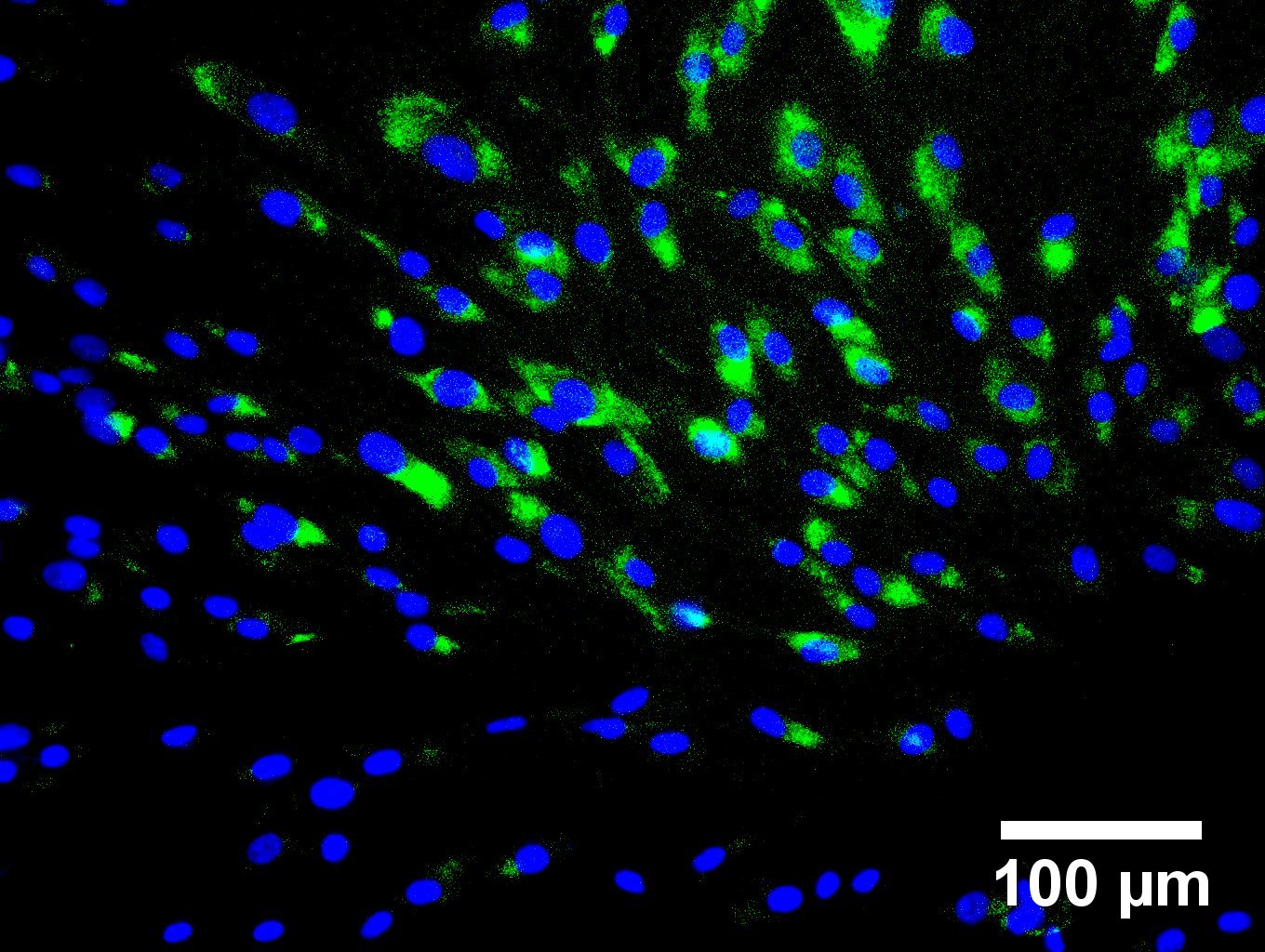

Supplement: Supplementary file 3 — Source data Fig. 1 [file 44318_2024_220_MOESM3_ESM.zip › Figure1/1E/Image/Day6-2(2).jpg]

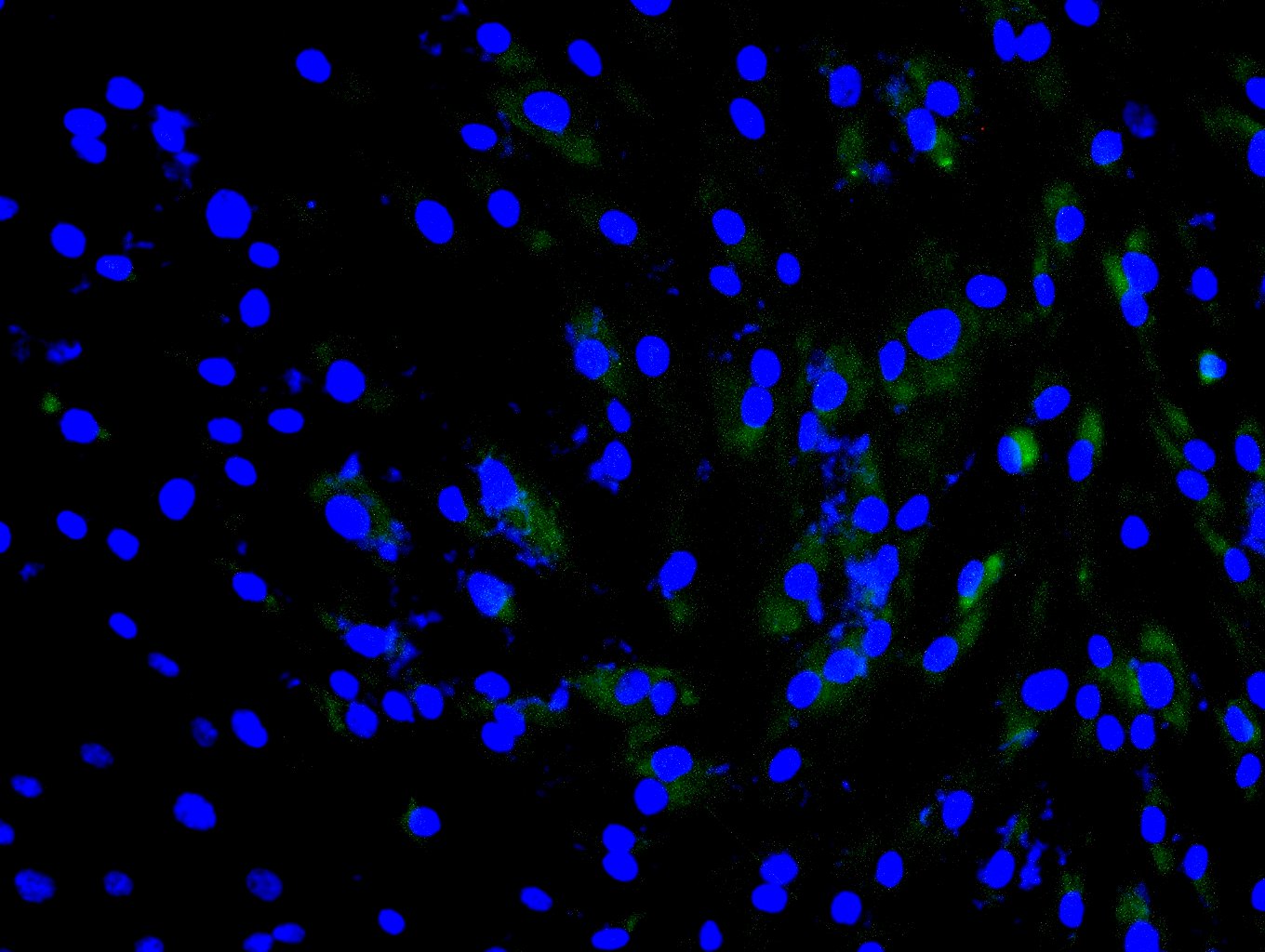

Supplement: Supplementary file 3 — Source data Fig. 1 [file 44318_2024_220_MOESM3_ESM.zip › Figure1/1E/Image/Day6-2(3).jpg]

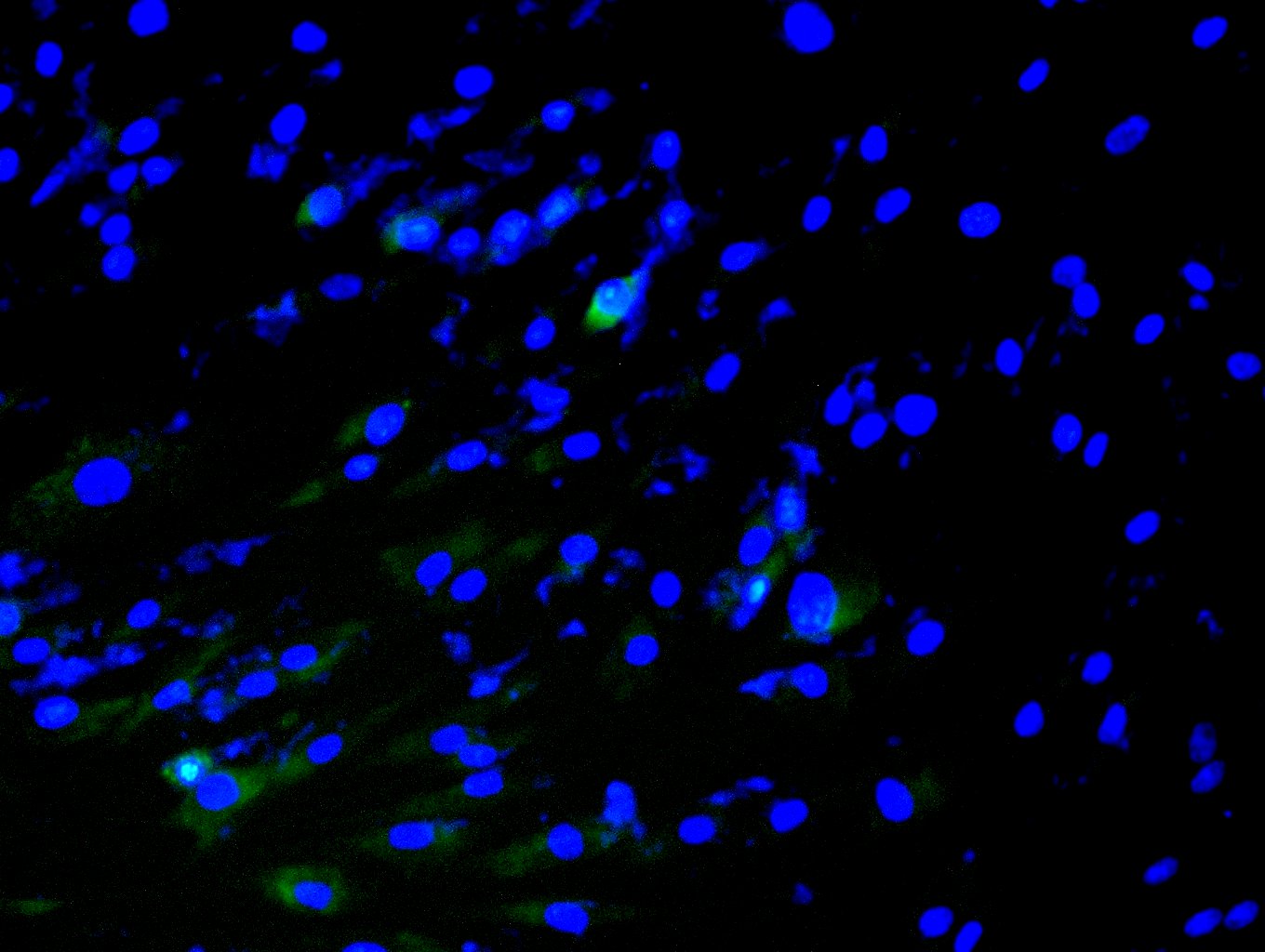

Supplement: Supplementary file 3 — Source data Fig. 1 [file 44318_2024_220_MOESM3_ESM.zip › Figure1/1E/Image/Day6-3(1).jpg]

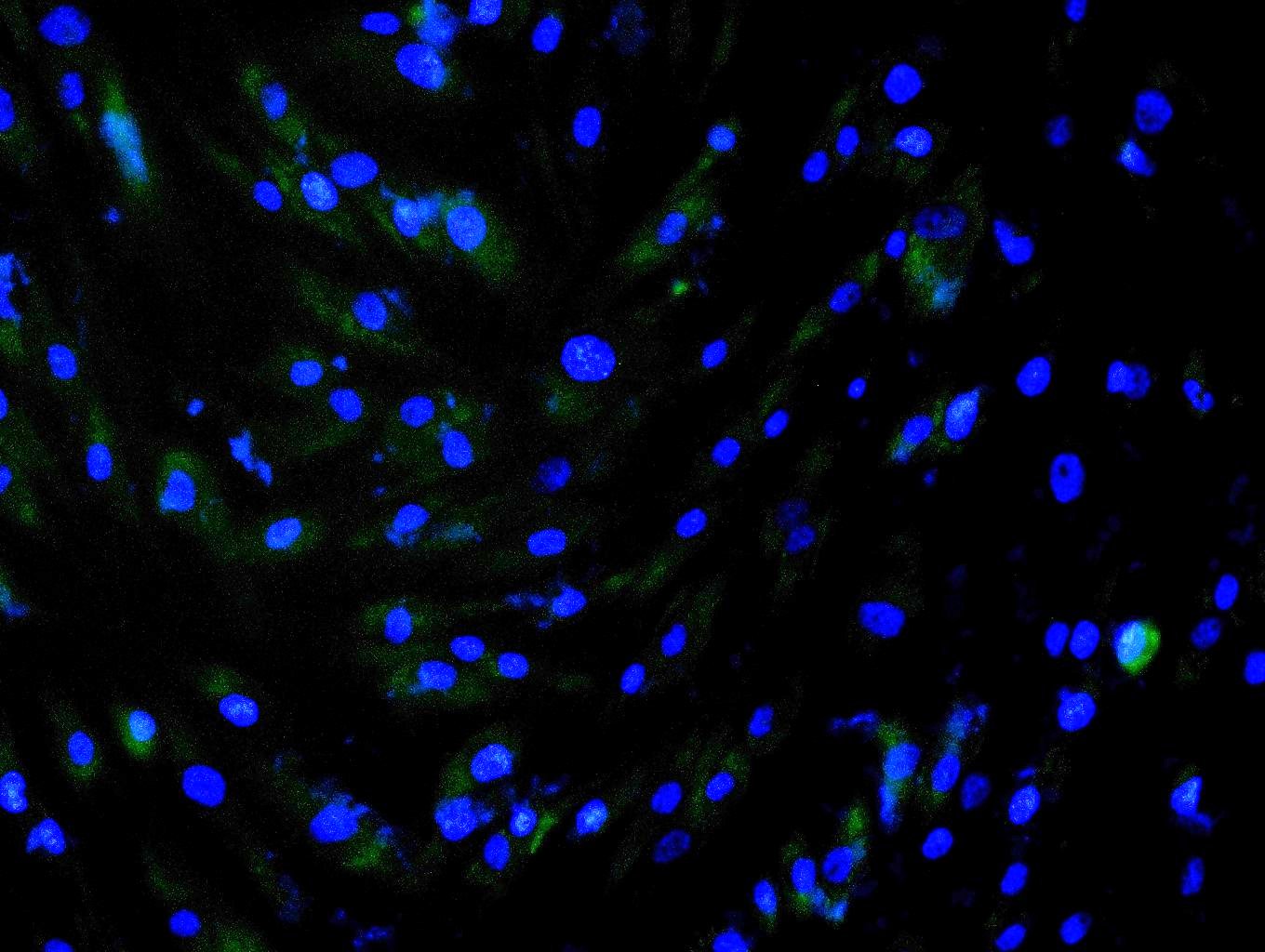

Supplement: Supplementary file 3 — Source data Fig. 1 [file 44318_2024_220_MOESM3_ESM.zip › Figure1/1E/Image/Day6-3(2).jpg]

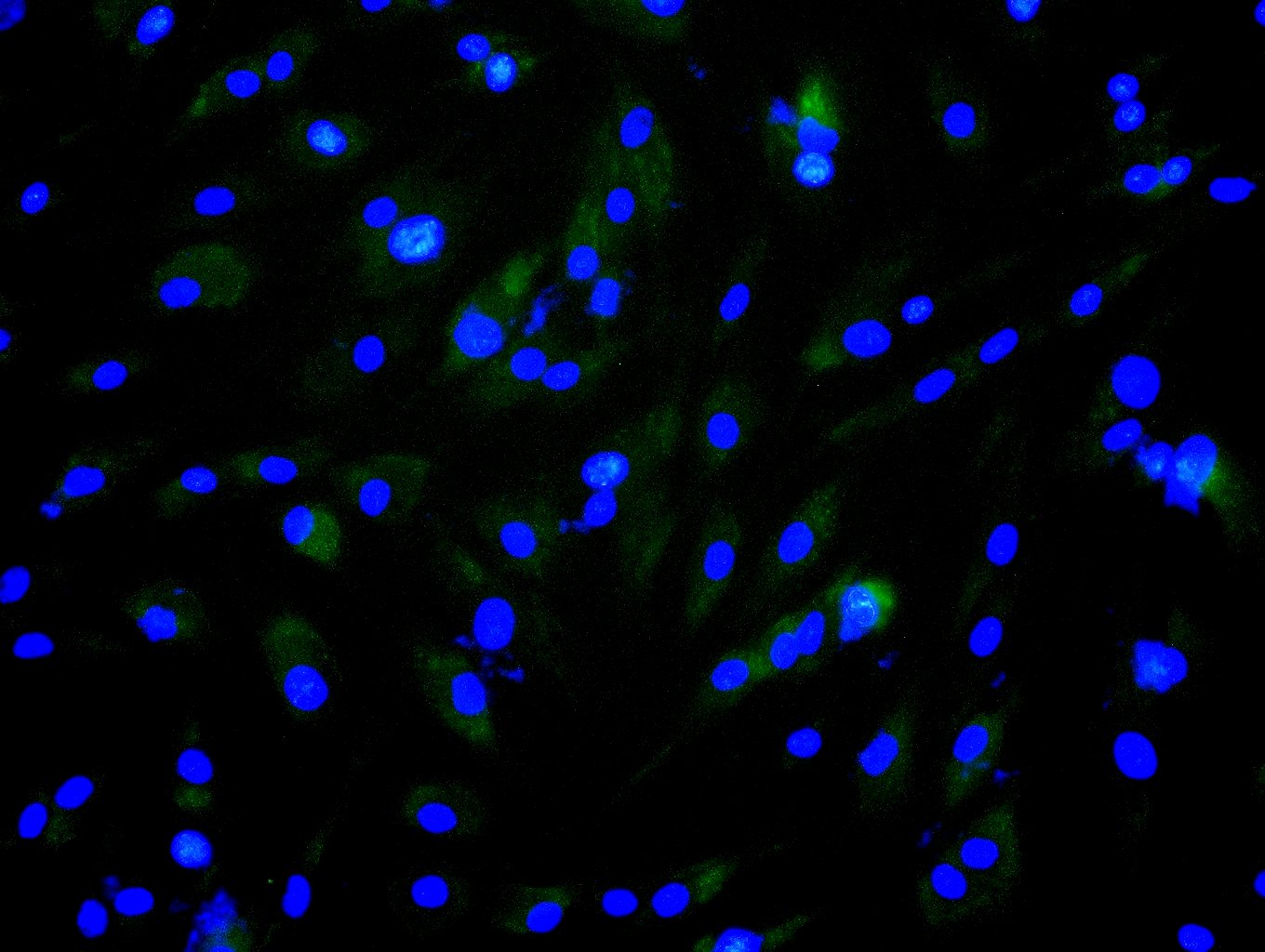

Supplement: Supplementary file 3 — Source data Fig. 1 [file 44318_2024_220_MOESM3_ESM.zip › Figure1/1E/Image/Day6-3(3).jpg]

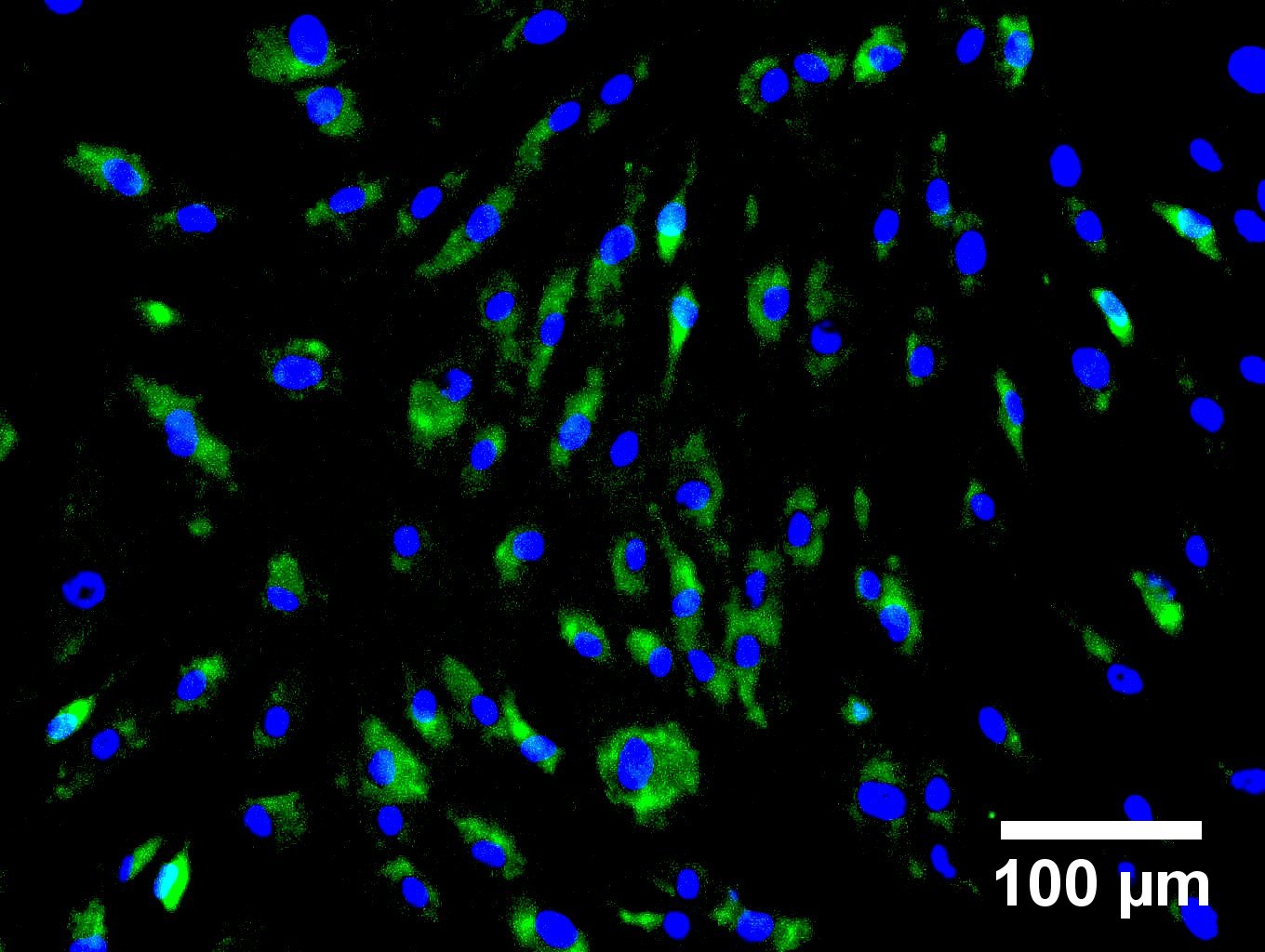

Supplement: Supplementary file 3 — Source data Fig. 1 [file 44318_2024_220_MOESM3_ESM.zip › Figure1/1E/Image/Day8-1(1).jpg]

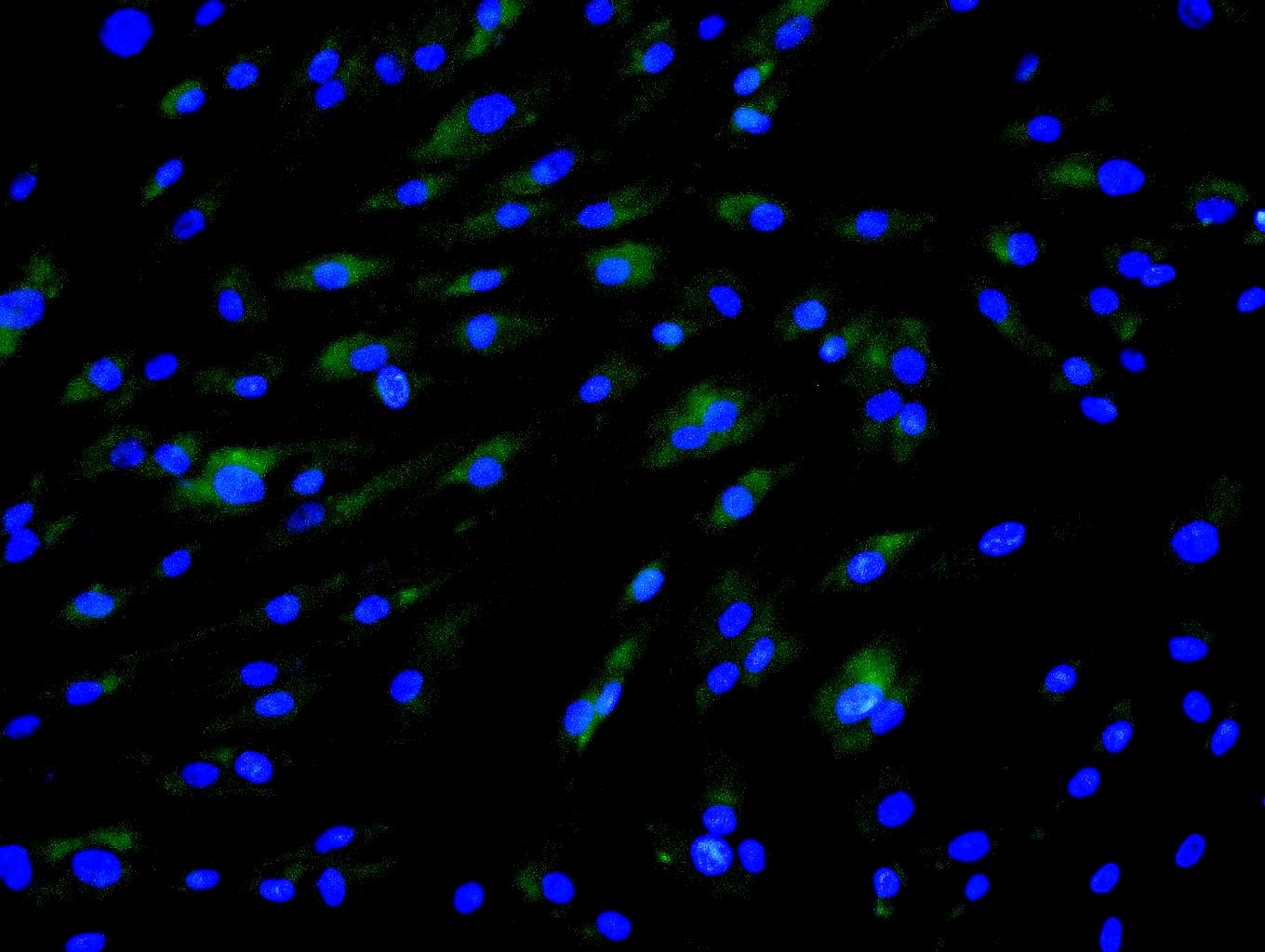

Supplement: Supplementary file 3 — Source data Fig. 1 [file 44318_2024_220_MOESM3_ESM.zip › Figure1/1E/Image/Day8-1(2).jpg]

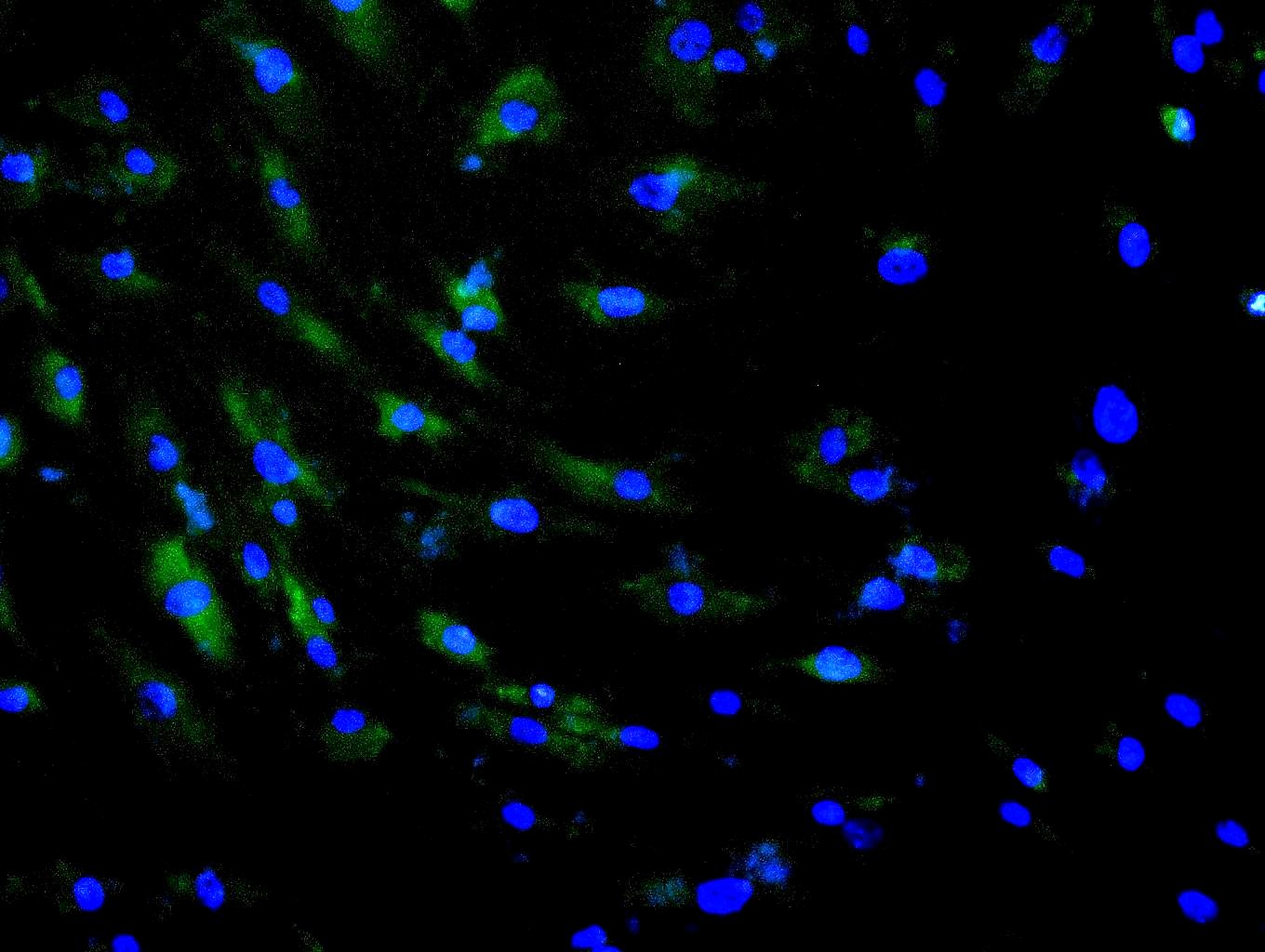

Supplement: Supplementary file 3 — Source data Fig. 1 [file 44318_2024_220_MOESM3_ESM.zip › Figure1/1E/Image/Day8-1(3).jpg]

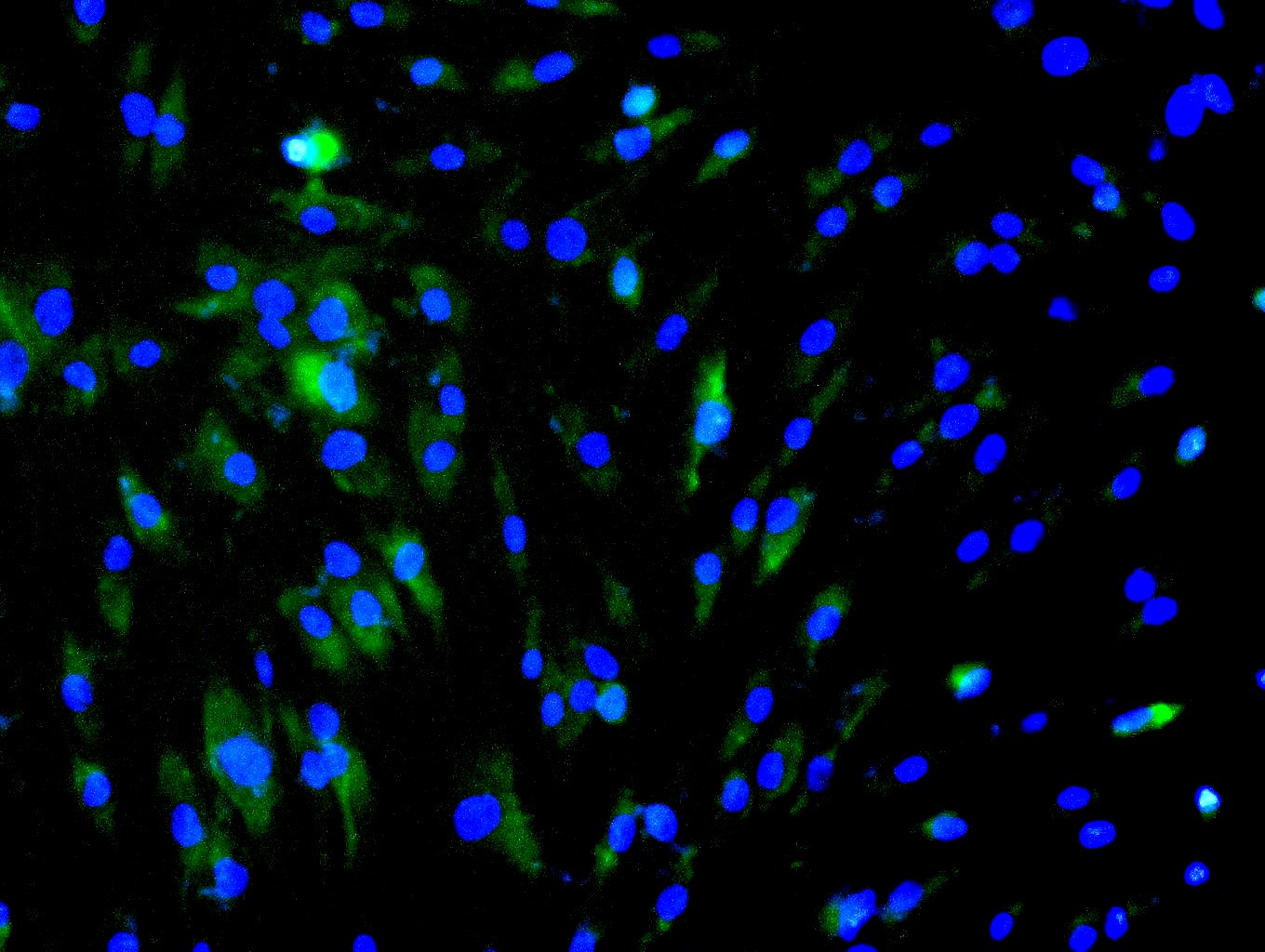

Supplement: Supplementary file 3 — Source data Fig. 1 [file 44318_2024_220_MOESM3_ESM.zip › Figure1/1E/Image/Day8-2(1).jpg]

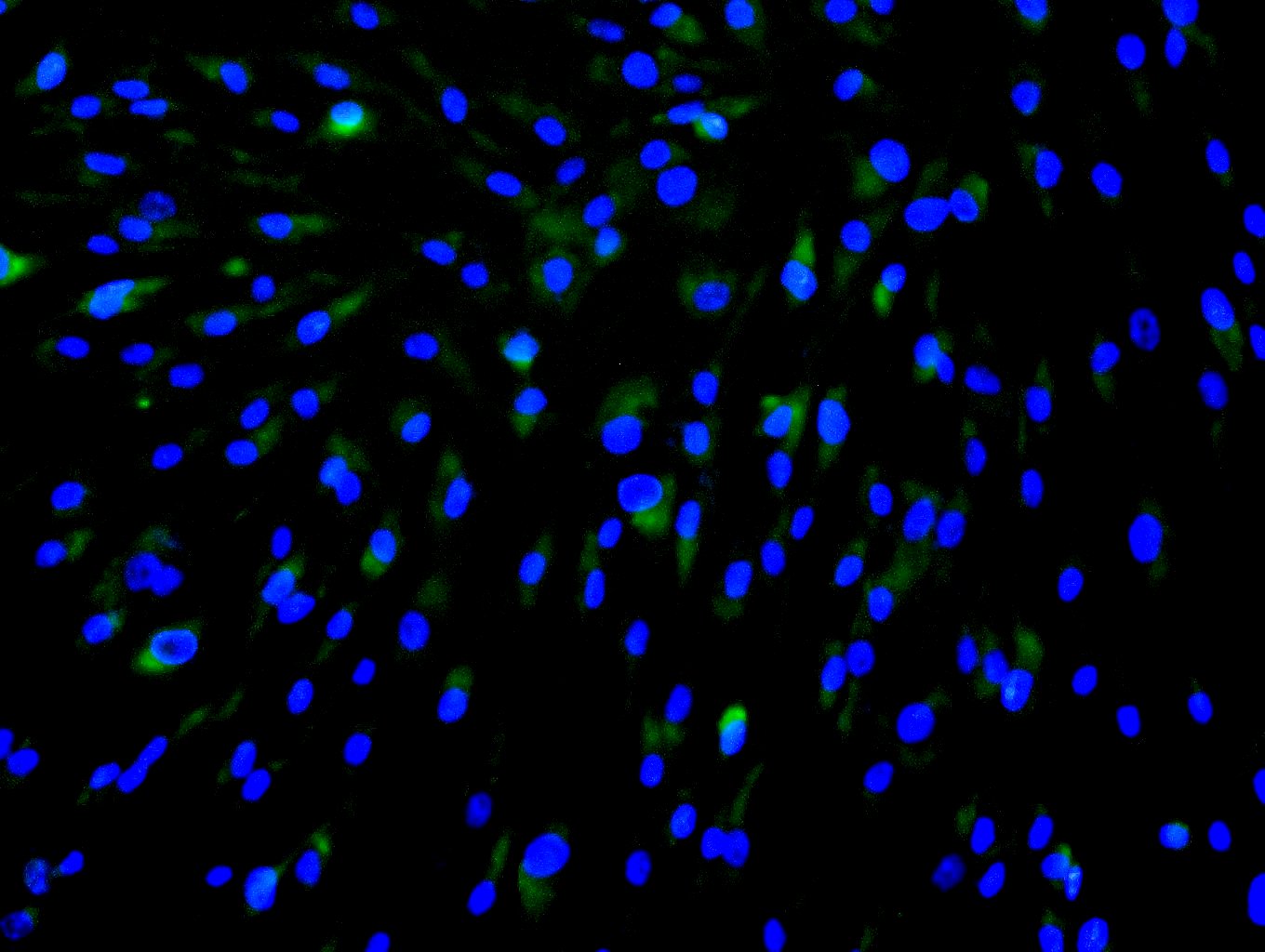

Supplement: Supplementary file 3 — Source data Fig. 1 [file 44318_2024_220_MOESM3_ESM.zip › Figure1/1E/Image/Day8-2(2).jpg]

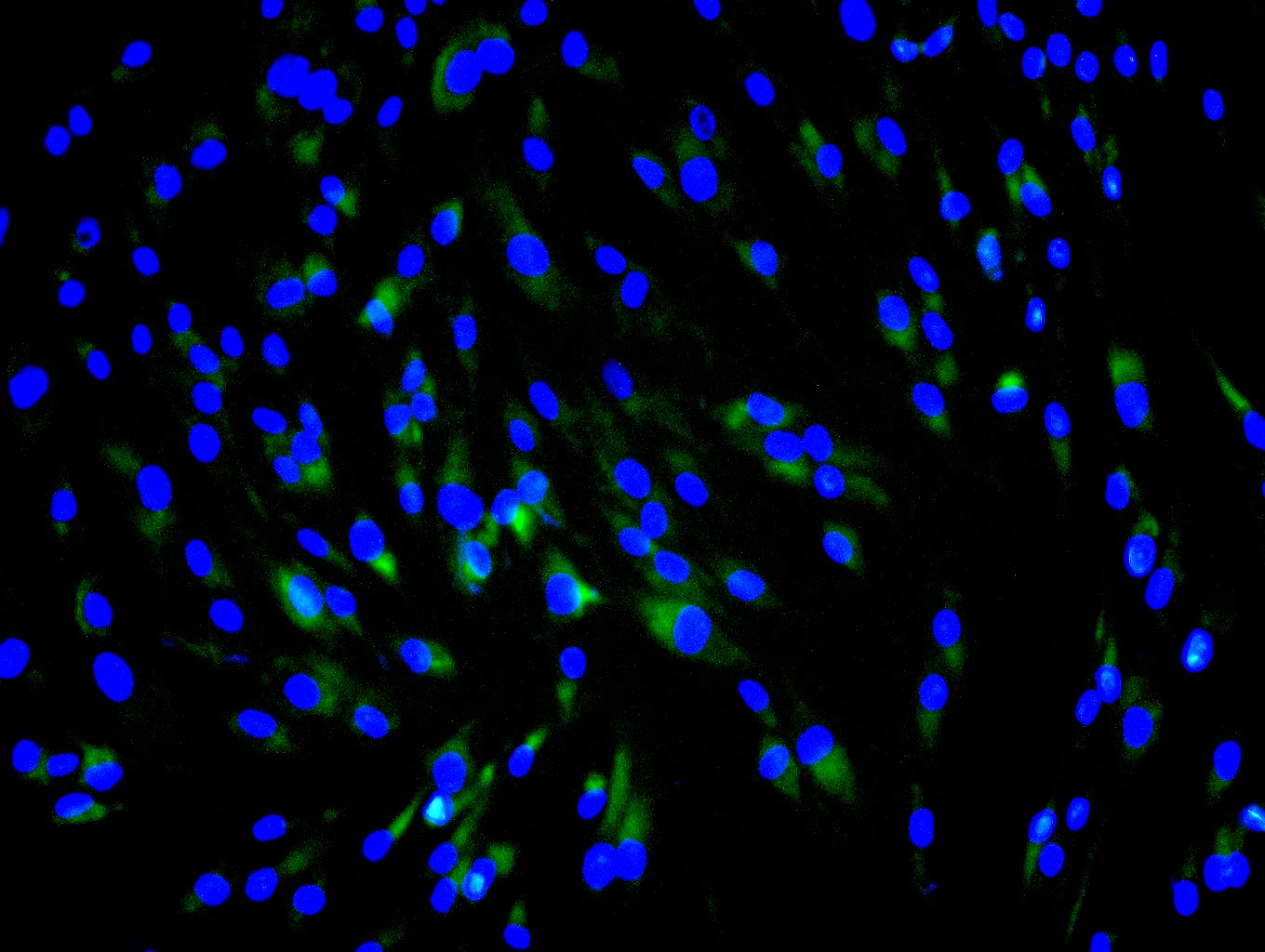

Supplement: Supplementary file 3 — Source data Fig. 1 [file 44318_2024_220_MOESM3_ESM.zip › Figure1/1E/Image/Day8-2(3).jpg]

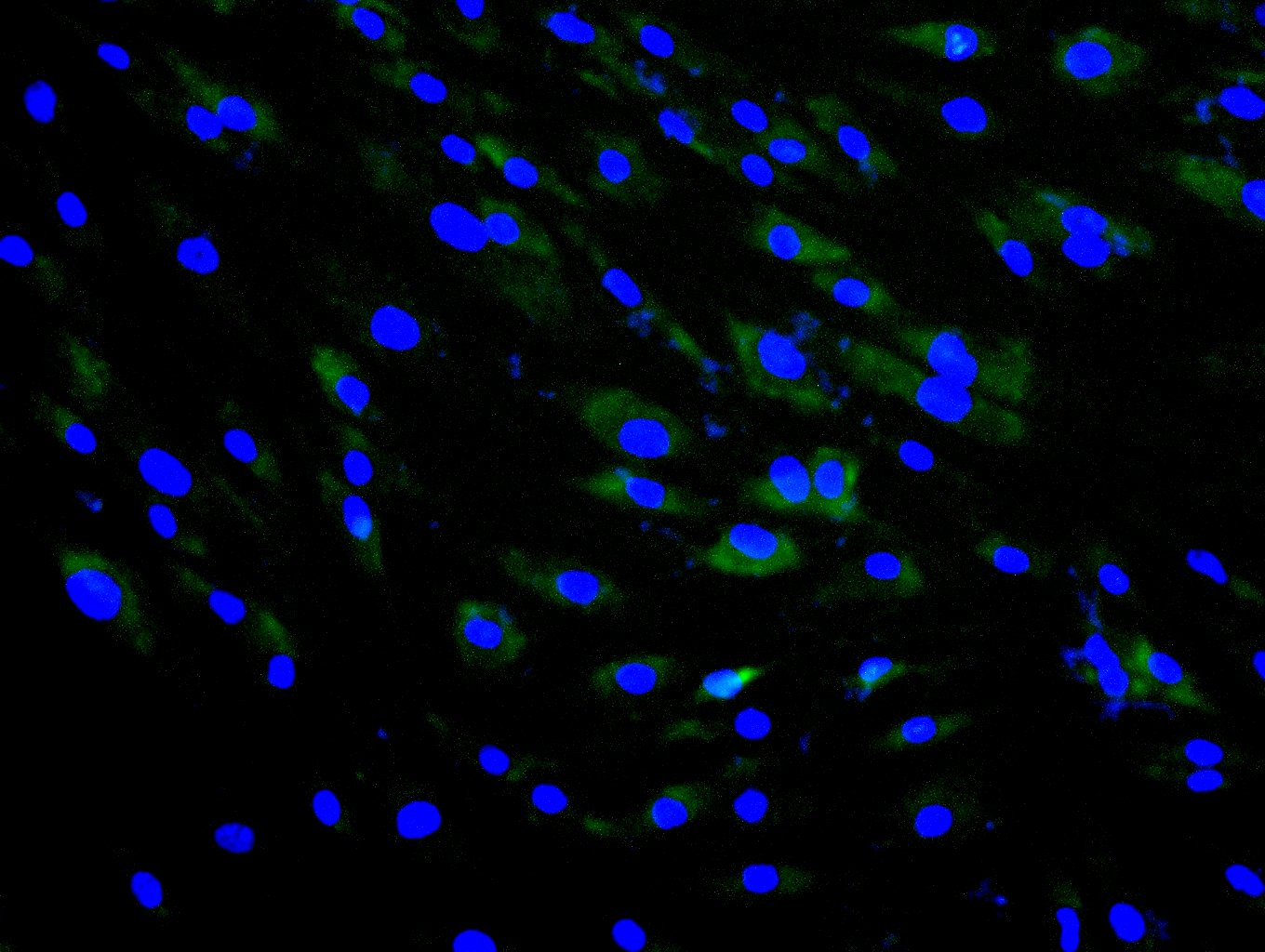

Supplement: Supplementary file 3 — Source data Fig. 1 [file 44318_2024_220_MOESM3_ESM.zip › Figure1/1E/Image/Day8-3 (1).jpg]

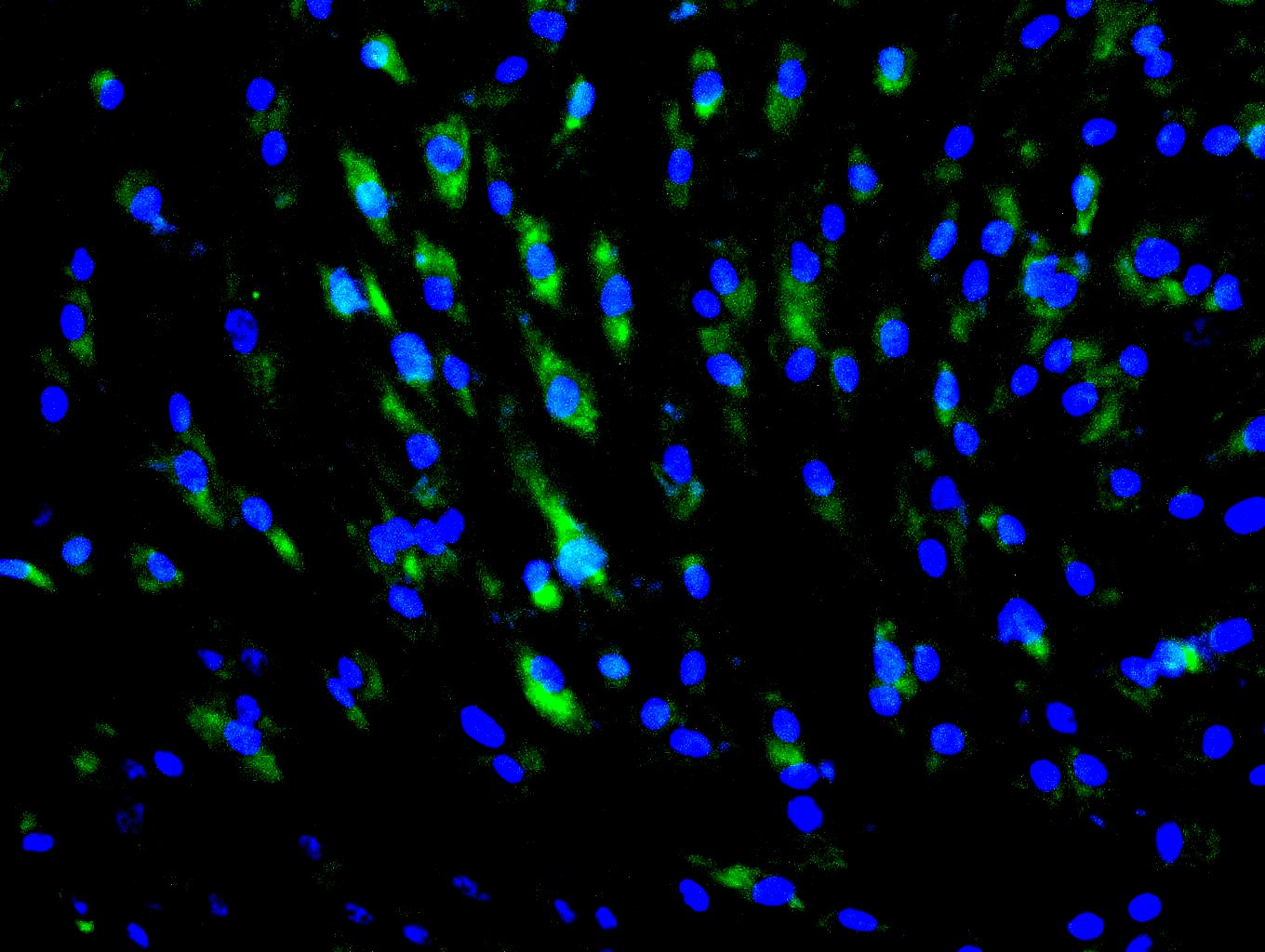

Supplement: Supplementary file 3 — Source data Fig. 1 [file 44318_2024_220_MOESM3_ESM.zip › Figure1/1E/Image/Day8-3 (2).jpg]

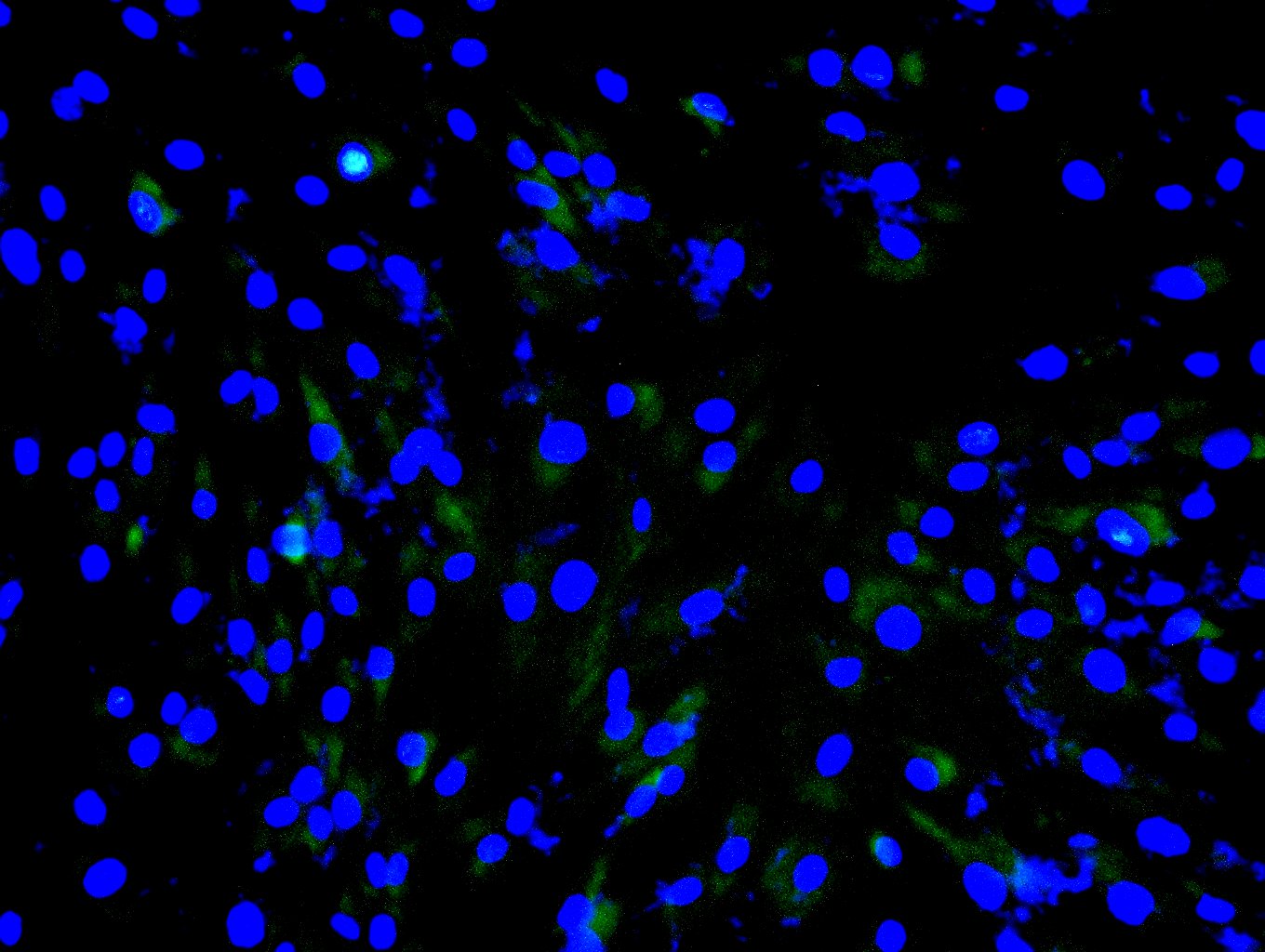

Supplement: Supplementary file 3 — Source data Fig. 1 [file 44318_2024_220_MOESM3_ESM.zip › Figure1/1E/Image/Day8-3 (3).jpg]

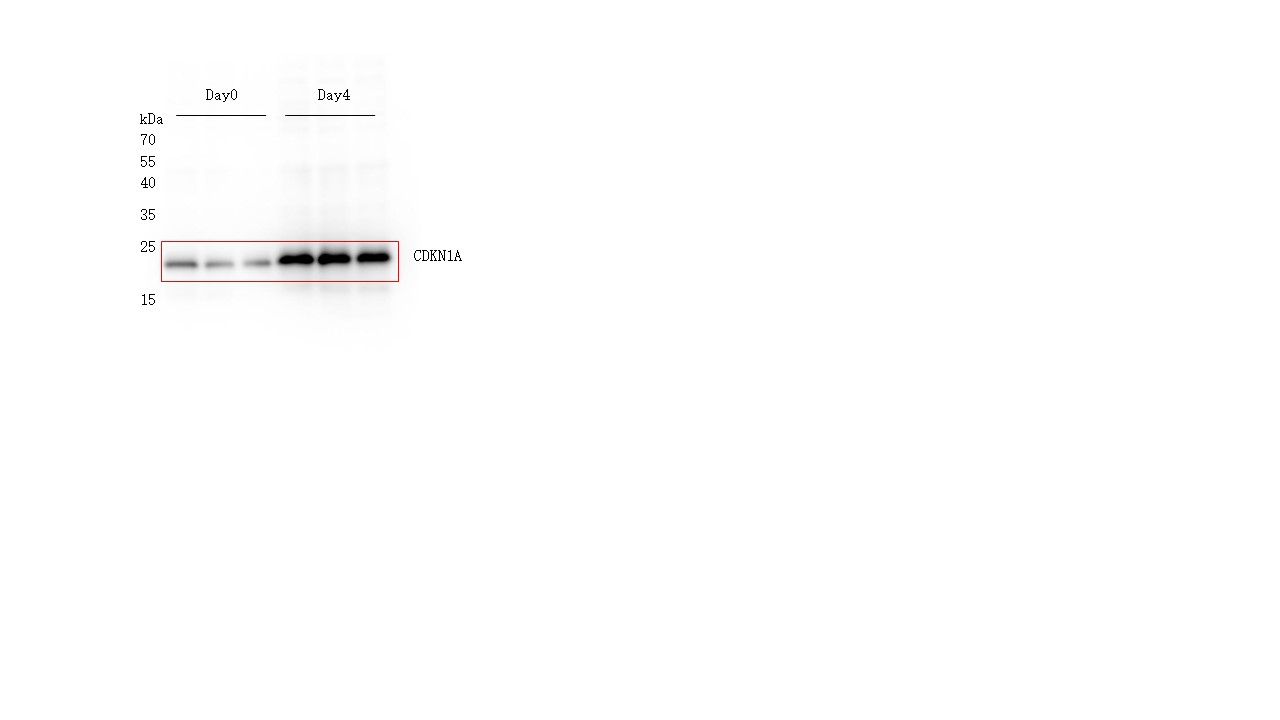

Supplement: Supplementary file 3 — Source data Fig. 1 [file 44318_2024_220_MOESM3_ESM.zip › Figure1/1F/western CDKN1A.jpg]

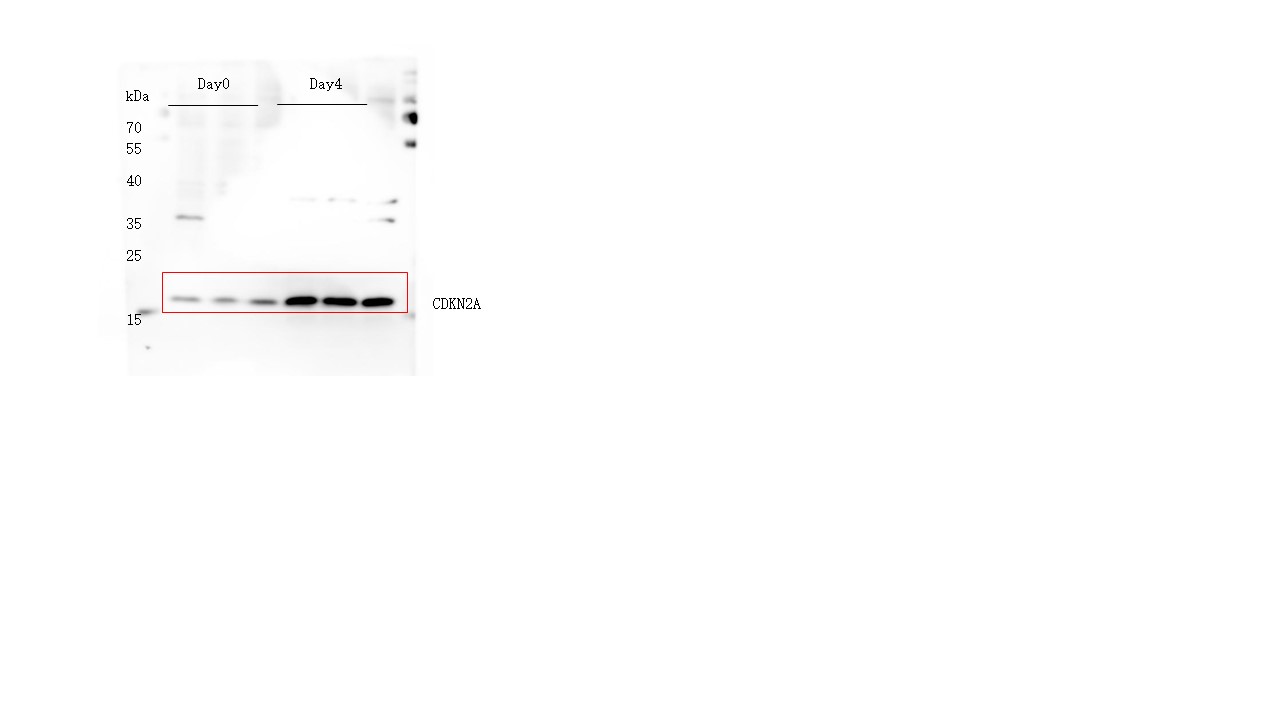

Supplement: Supplementary file 3 — Source data Fig. 1 [file 44318_2024_220_MOESM3_ESM.zip › Figure1/1F/western CDKN2A.jpg]

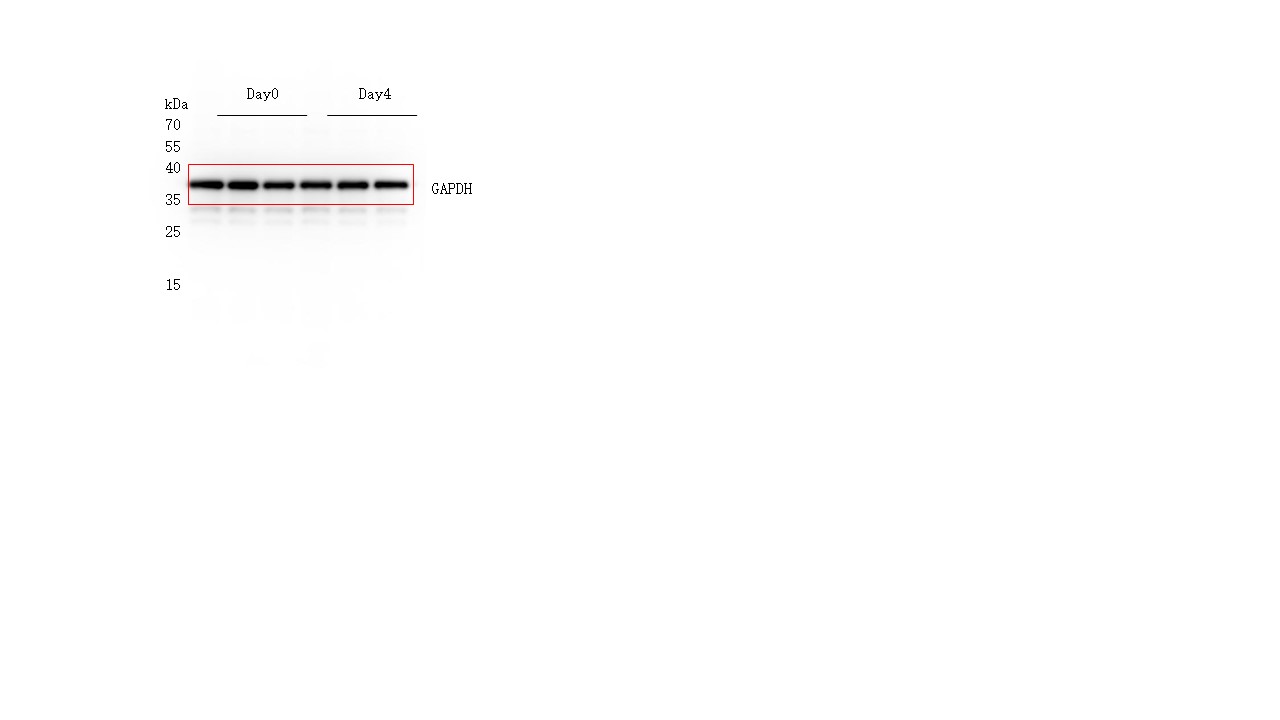

Supplement: Supplementary file 3 — Source data Fig. 1 [file 44318_2024_220_MOESM3_ESM.zip › Figure1/1F/western GAPDH.jpg]

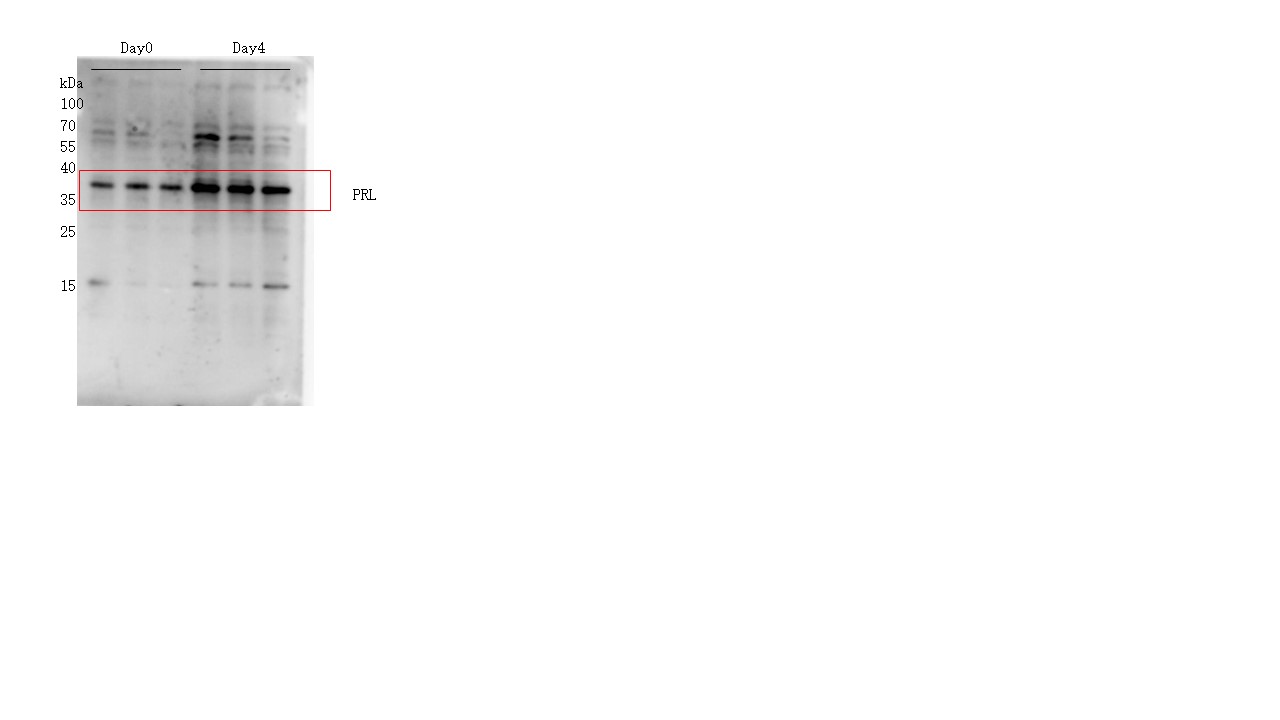

Supplement: Supplementary file 3 — Source data Fig. 1 [file 44318_2024_220_MOESM3_ESM.zip › Figure1/1F/western PRL.jpg]

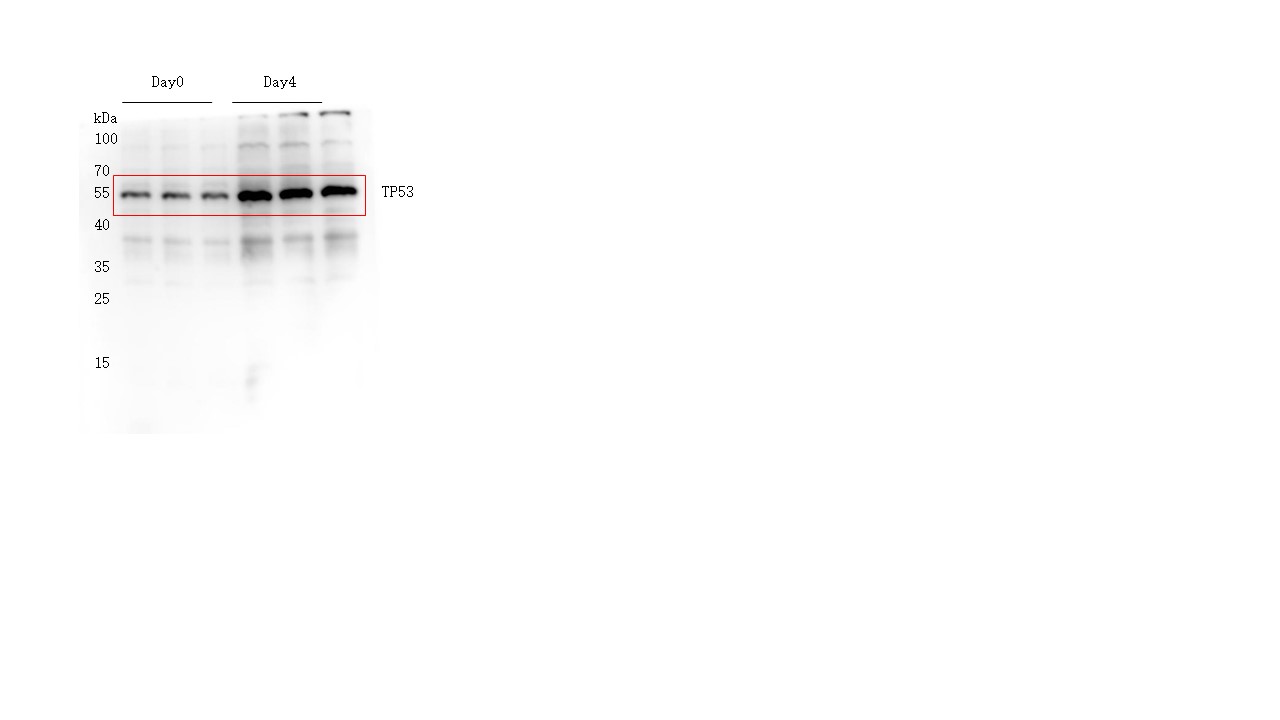

Supplement: Supplementary file 3 — Source data Fig. 1 [file 44318_2024_220_MOESM3_ESM.zip › Figure1/1F/western TP53.jpg]

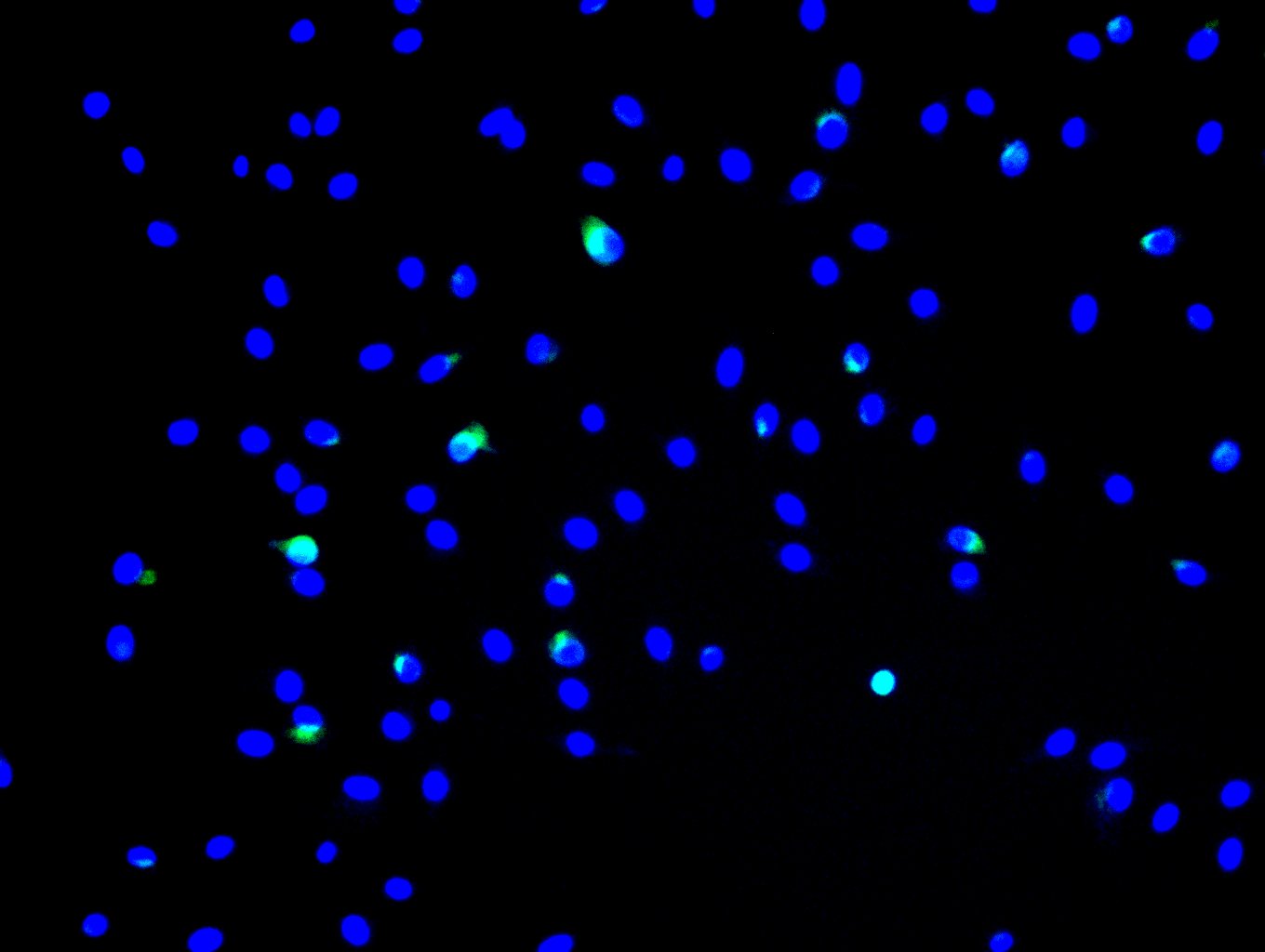

Supplement: Supplementary file 3 — Source data Fig. 1 [file 44318_2024_220_MOESM3_ESM.zip › Figure1/1G/DSC1 (1).jpg]

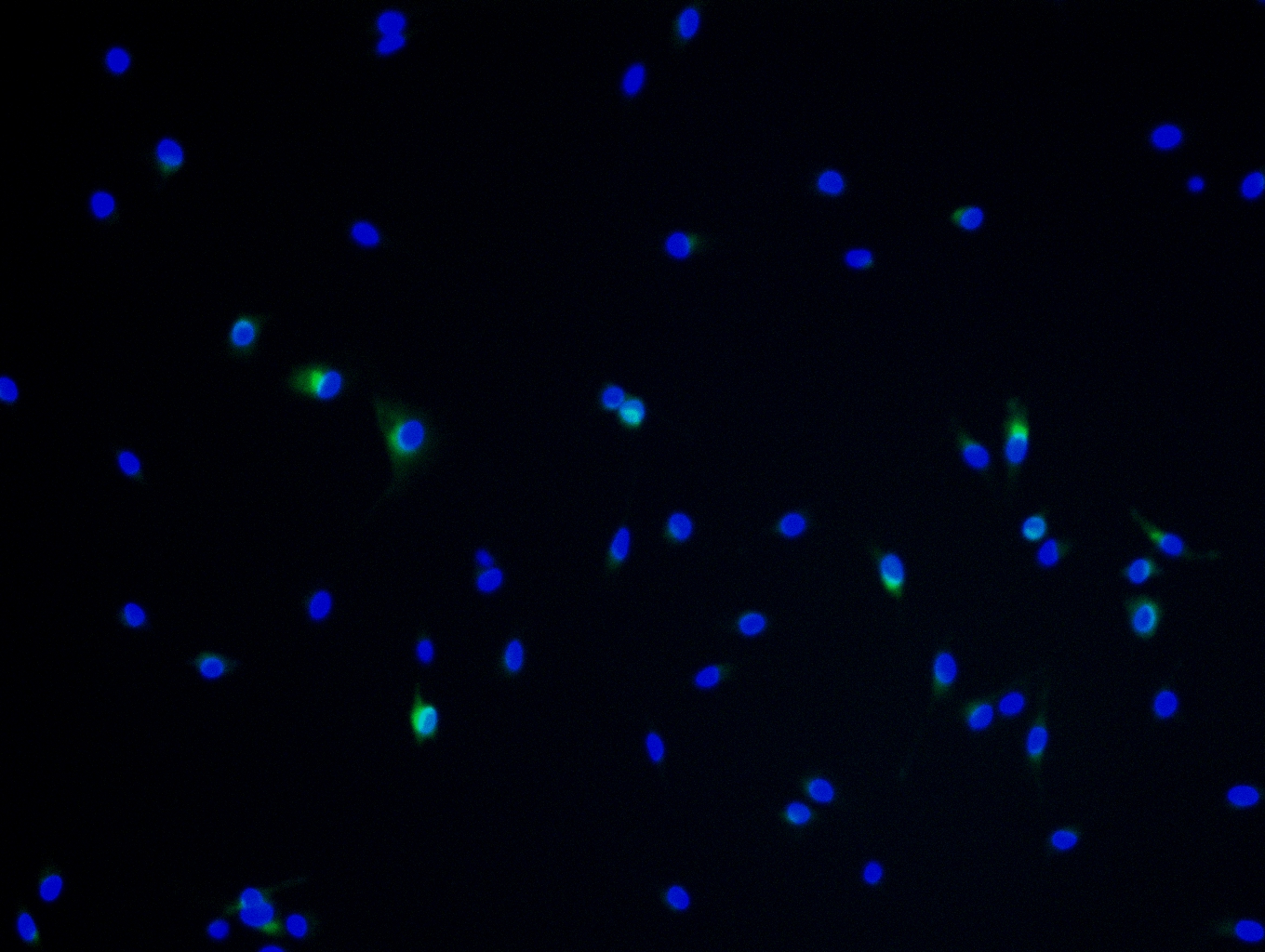

Supplement: Supplementary file 3 — Source data Fig. 1 [file 44318_2024_220_MOESM3_ESM.zip › Figure1/1G/DSC1 (2).jpg]

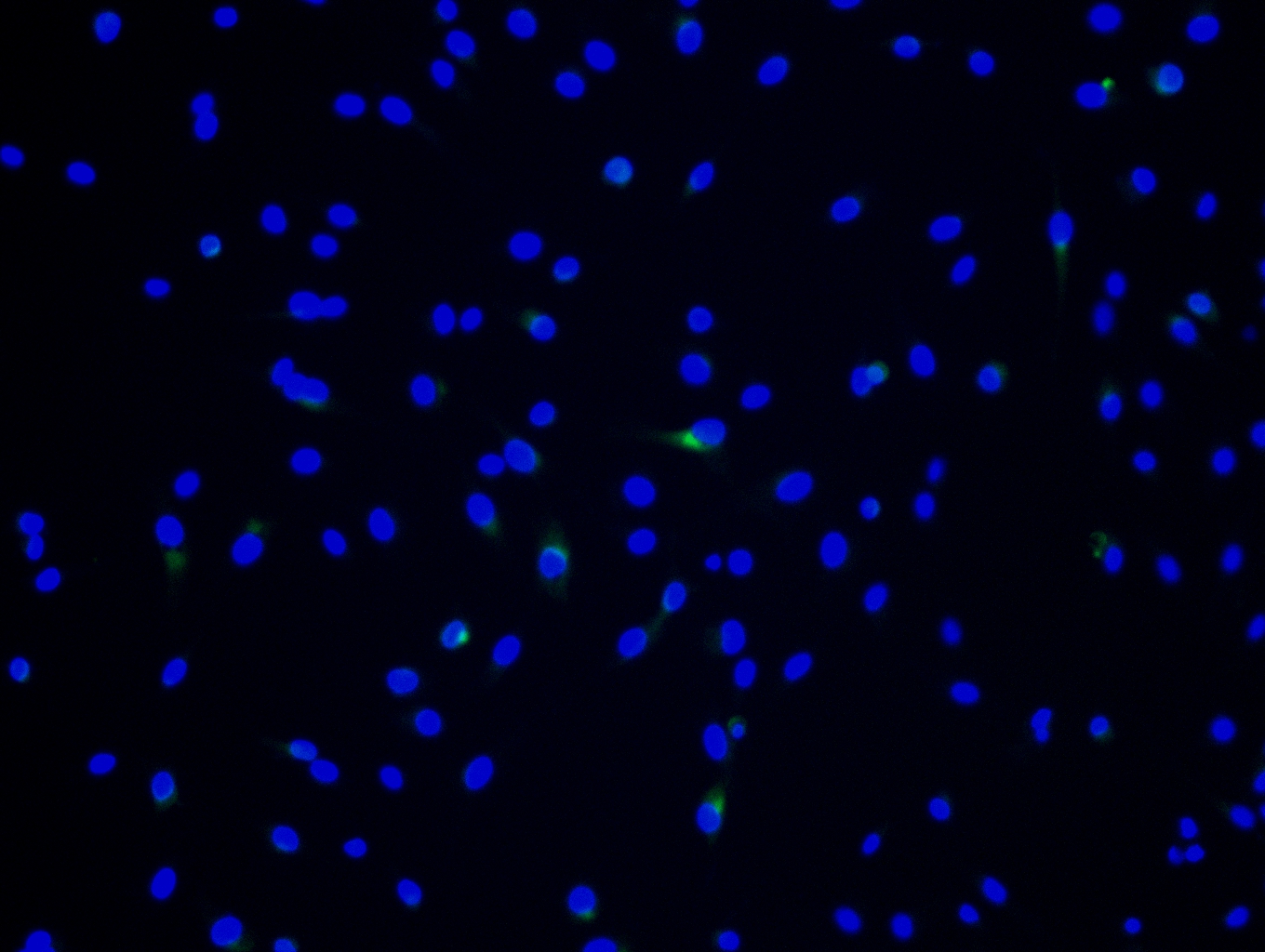

Supplement: Supplementary file 3 — Source data Fig. 1 [file 44318_2024_220_MOESM3_ESM.zip › Figure1/1G/DSC1 (3).jpg]

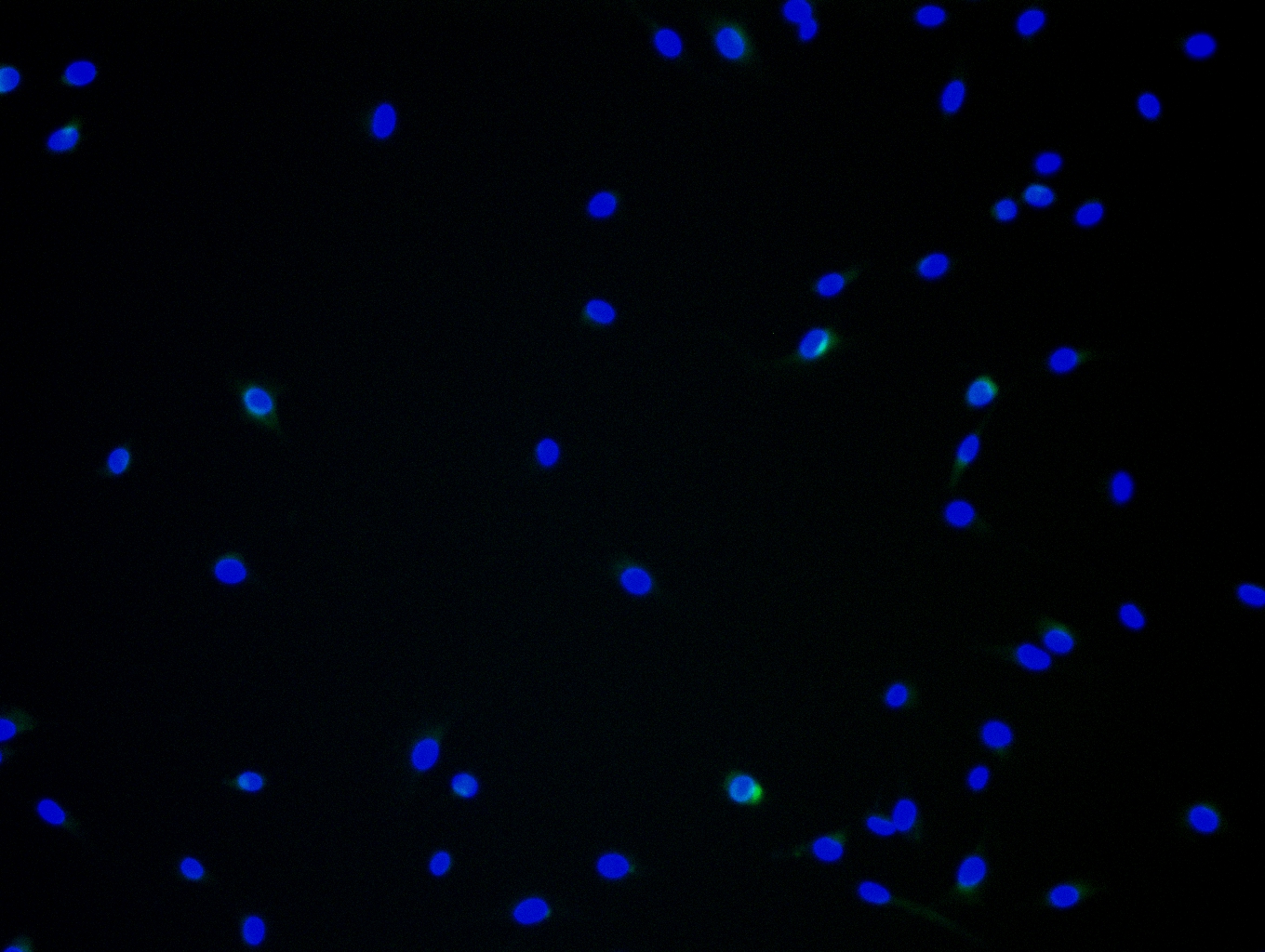

Supplement: Supplementary file 3 — Source data Fig. 1 [file 44318_2024_220_MOESM3_ESM.zip › Figure1/1G/DSC2 (1).jpg]

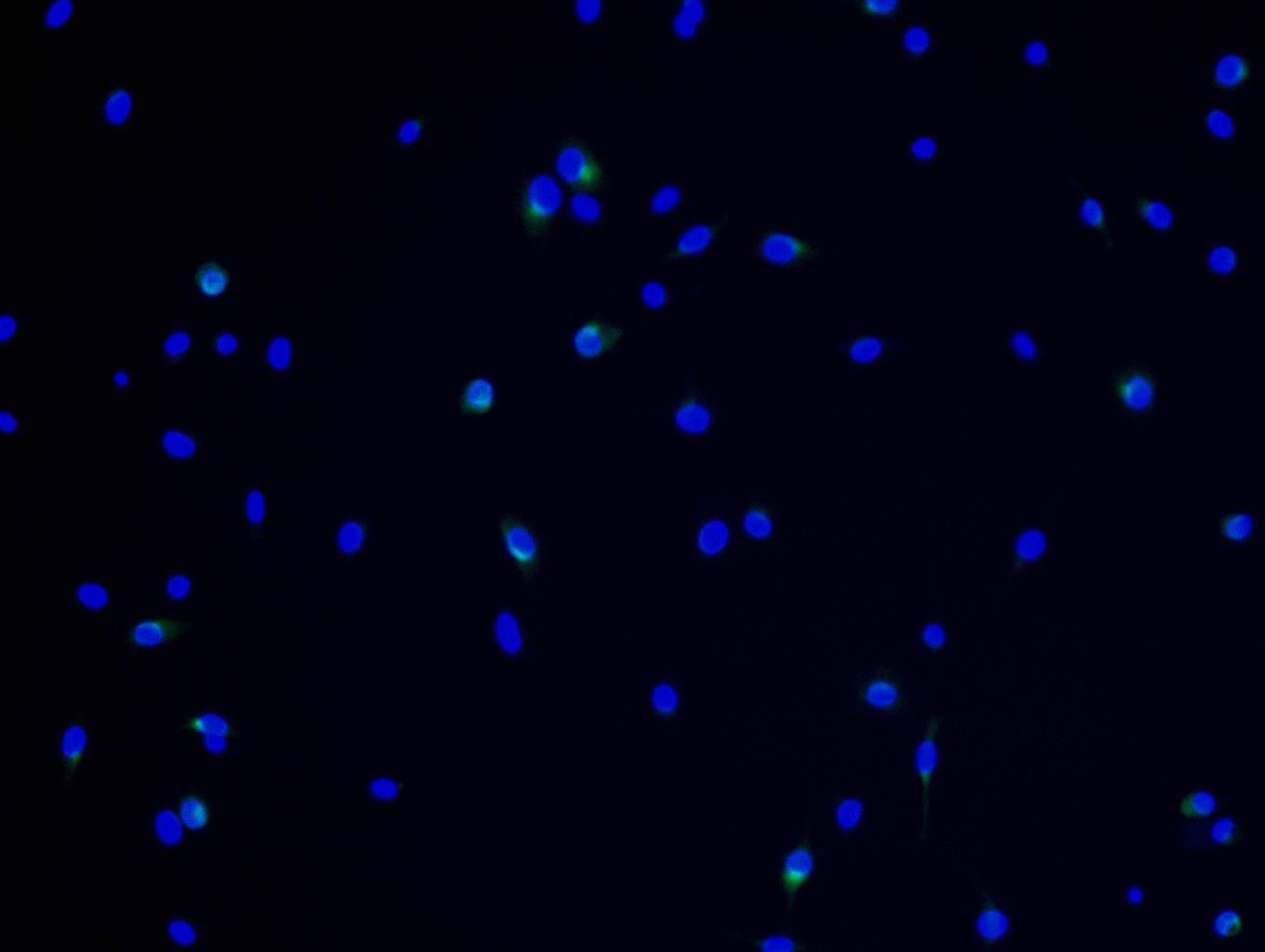

Supplement: Supplementary file 3 — Source data Fig. 1 [file 44318_2024_220_MOESM3_ESM.zip › Figure1/1G/DSC2 (2).jpg]

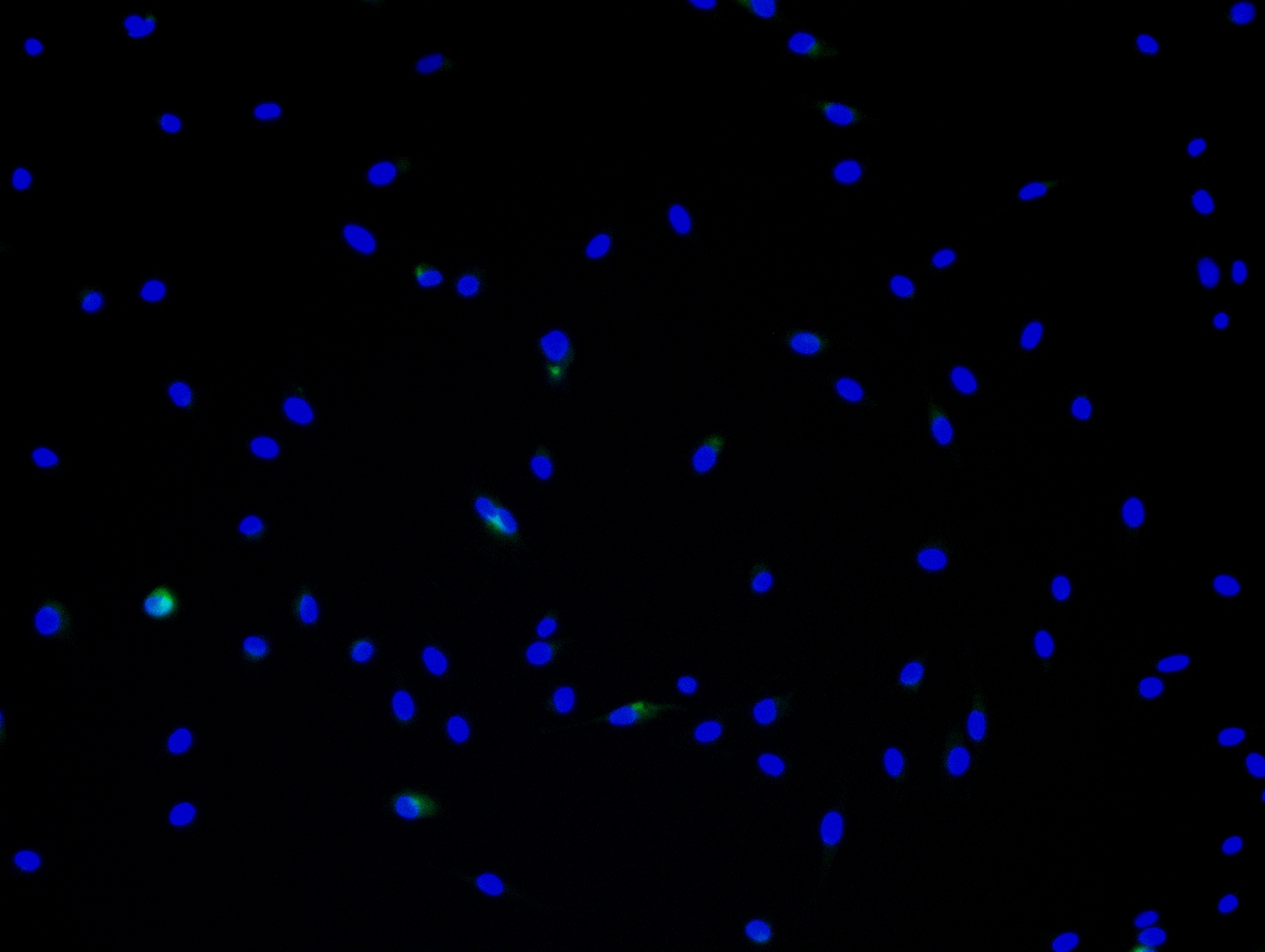

Supplement: Supplementary file 3 — Source data Fig. 1 [file 44318_2024_220_MOESM3_ESM.zip › Figure1/1G/DSC2 (3).jpg]

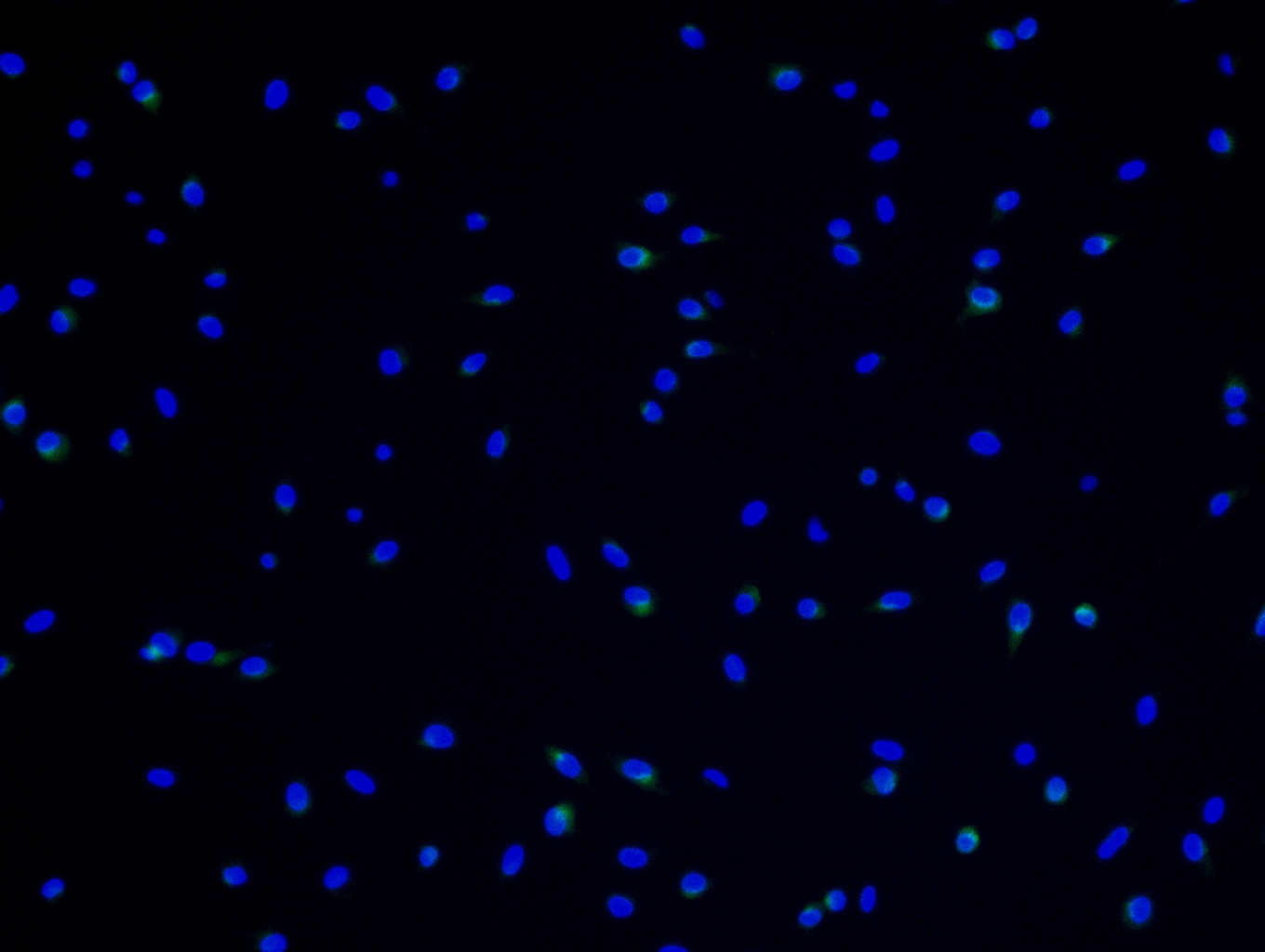

Supplement: Supplementary file 3 — Source data Fig. 1 [file 44318_2024_220_MOESM3_ESM.zip › Figure1/1G/DSC3 (1).jpg]

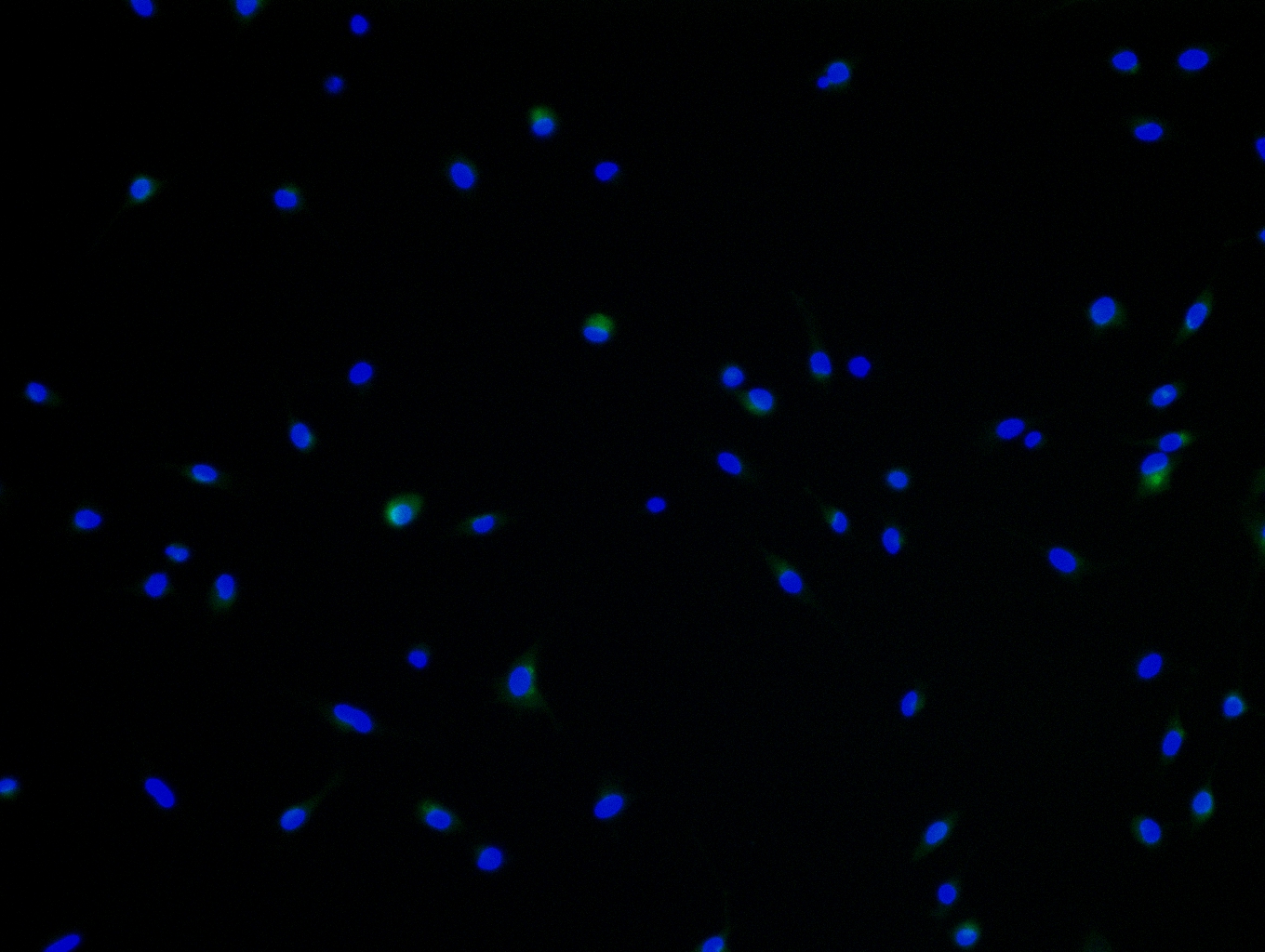

Supplement: Supplementary file 3 — Source data Fig. 1 [file 44318_2024_220_MOESM3_ESM.zip › Figure1/1G/DSC3 (2).jpg]

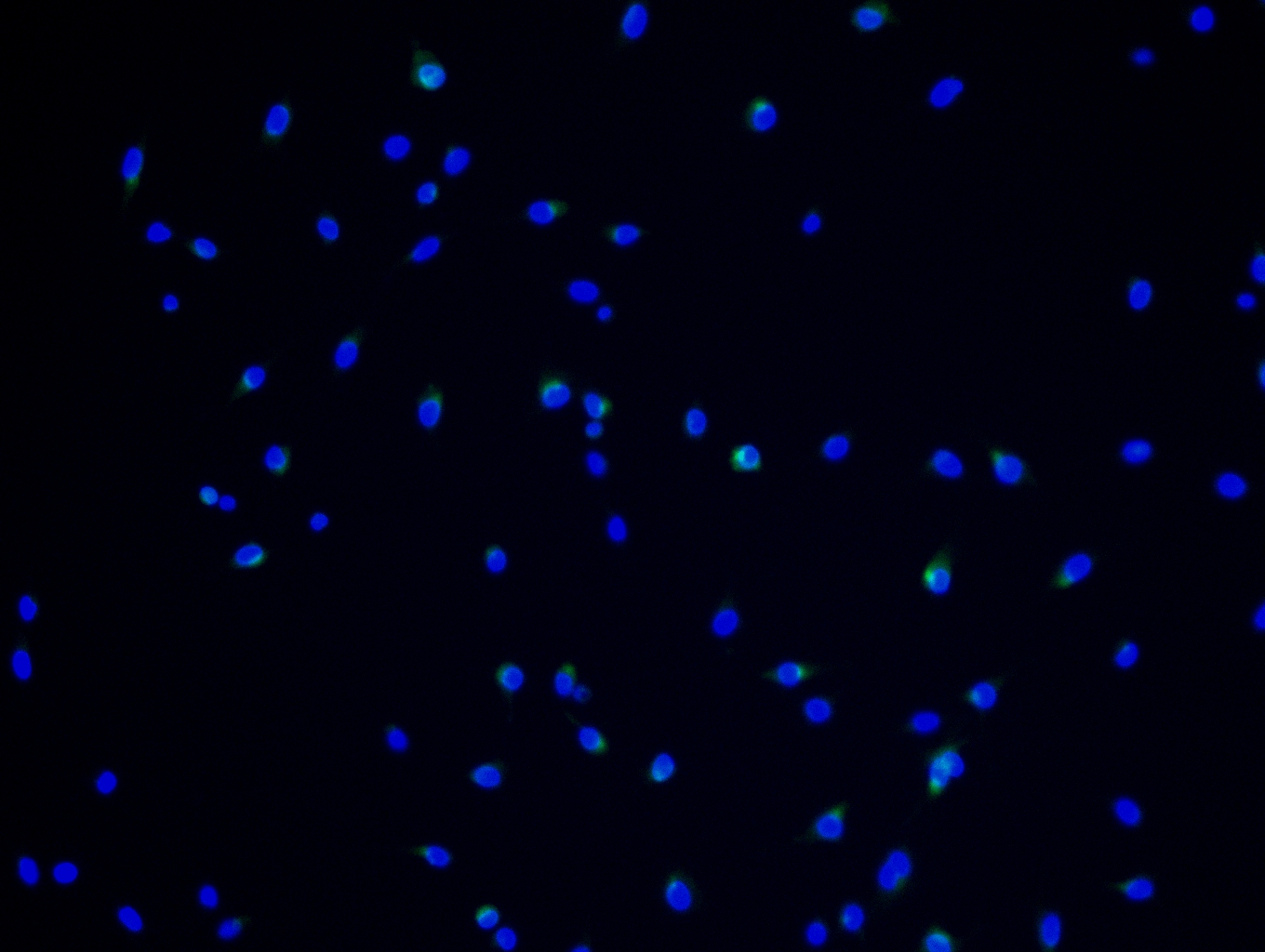

Supplement: Supplementary file 3 — Source data Fig. 1 [file 44318_2024_220_MOESM3_ESM.zip › Figure1/1G/DSC3 (3).jpg]

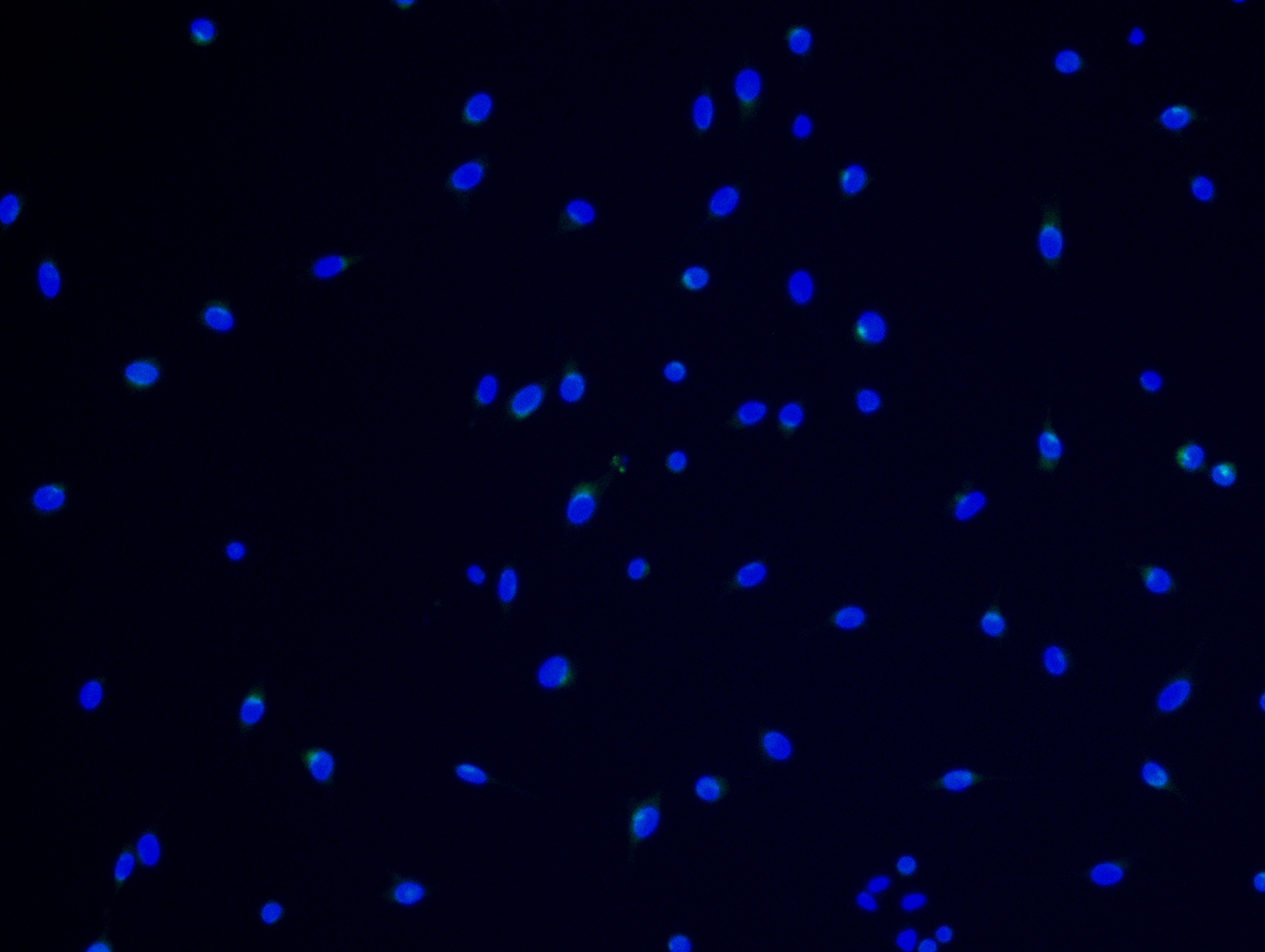

Supplement: Supplementary file 3 — Source data Fig. 1 [file 44318_2024_220_MOESM3_ESM.zip › Figure1/1G/DSC4 (1).jpg]

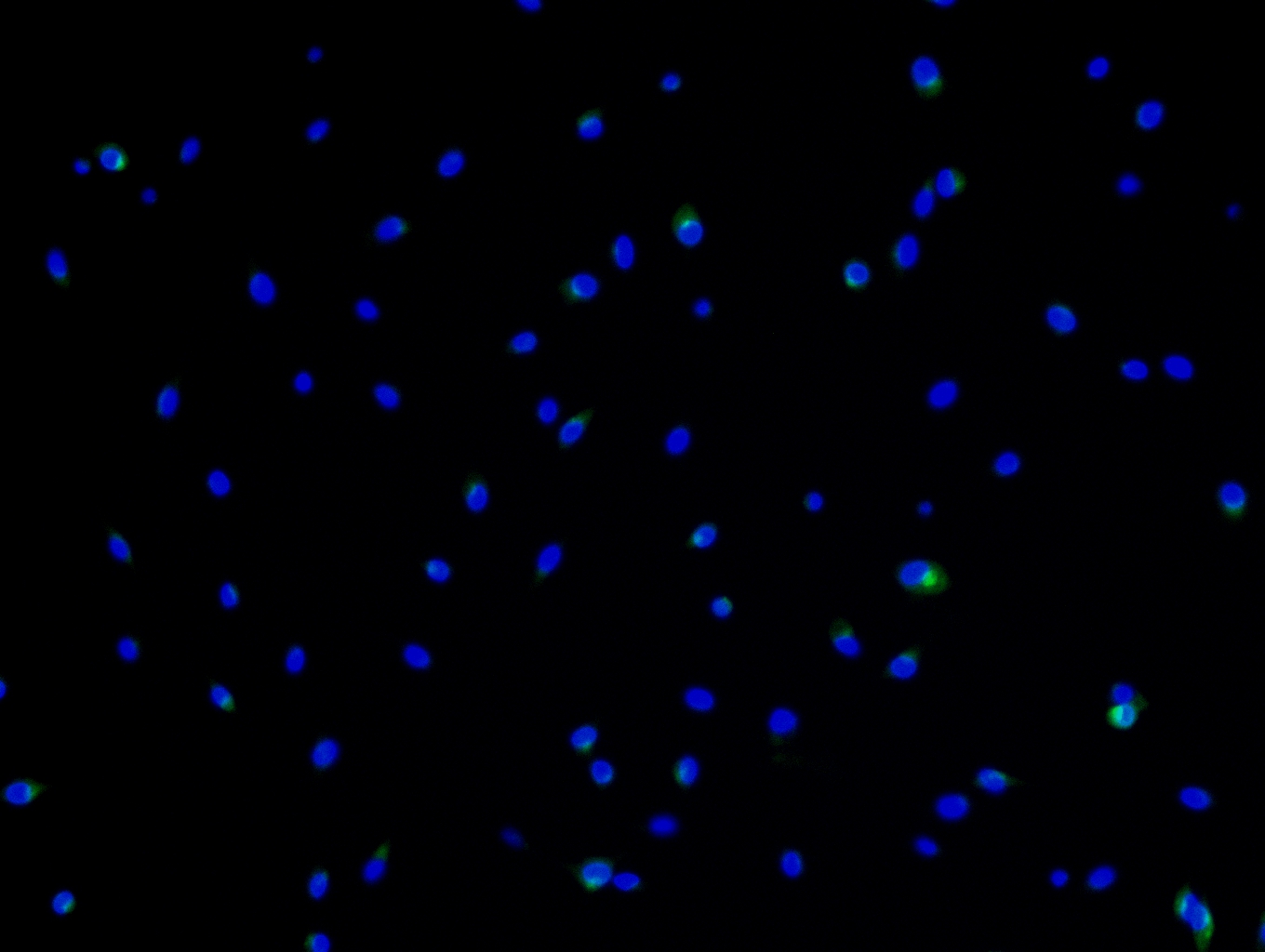

Supplement: Supplementary file 3 — Source data Fig. 1 [file 44318_2024_220_MOESM3_ESM.zip › Figure1/1G/DSC4 (2).jpg]

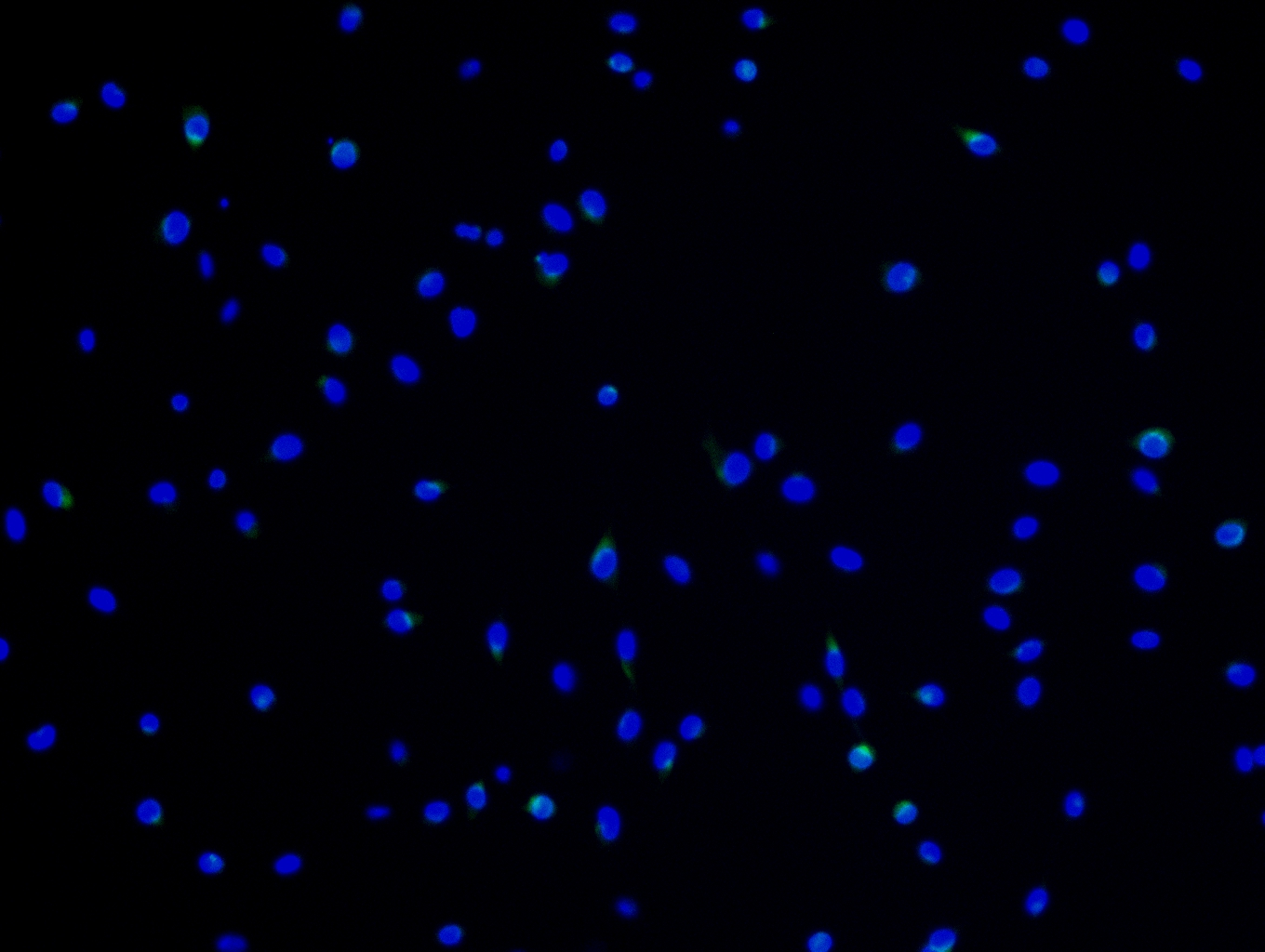

Supplement: Supplementary file 3 — Source data Fig. 1 [file 44318_2024_220_MOESM3_ESM.zip › Figure1/1G/DSC4 (3).jpg]

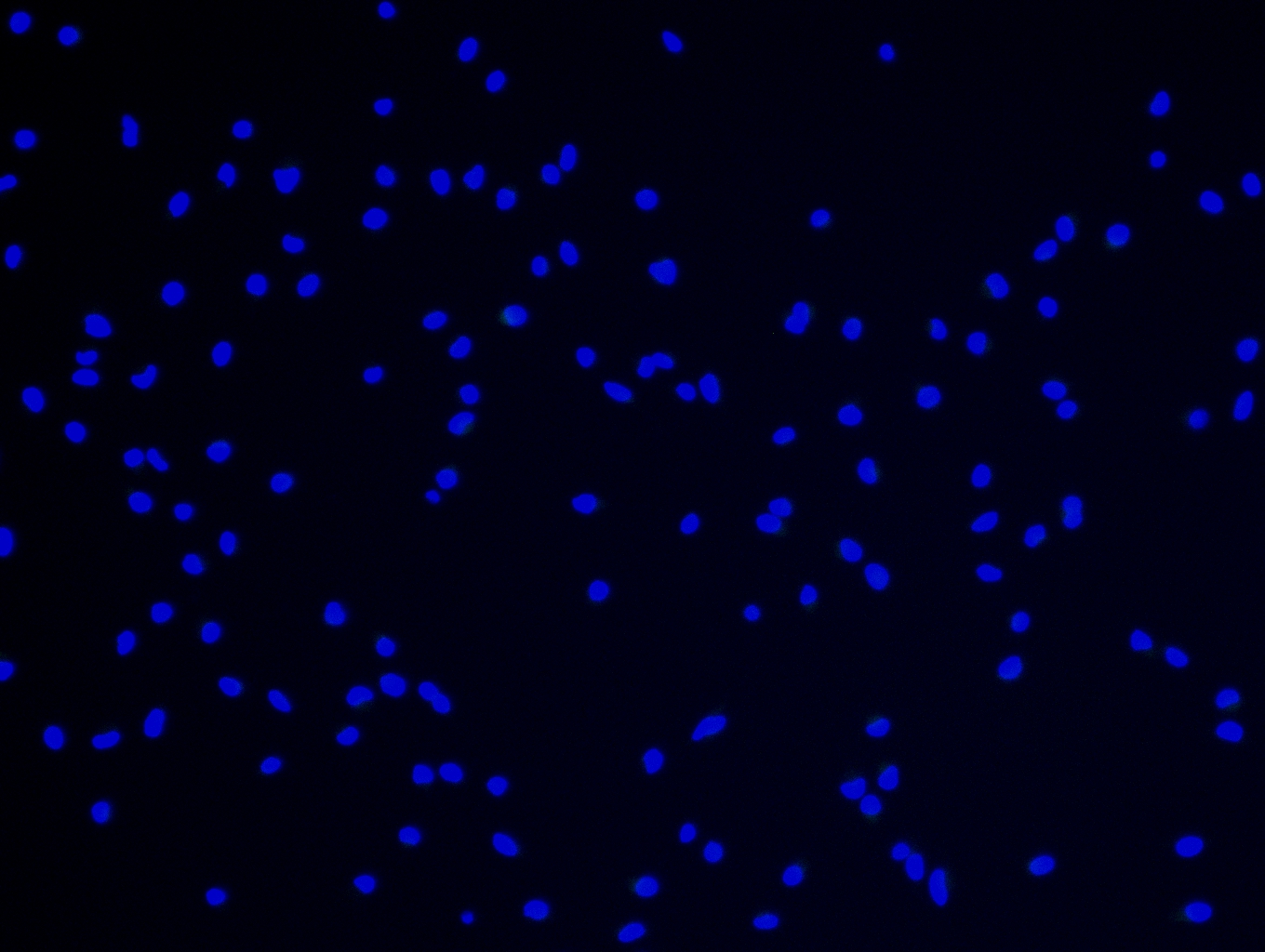

Supplement: Supplementary file 3 — Source data Fig. 1 [file 44318_2024_220_MOESM3_ESM.zip › Figure1/1G/ESC1 (1).jpg]

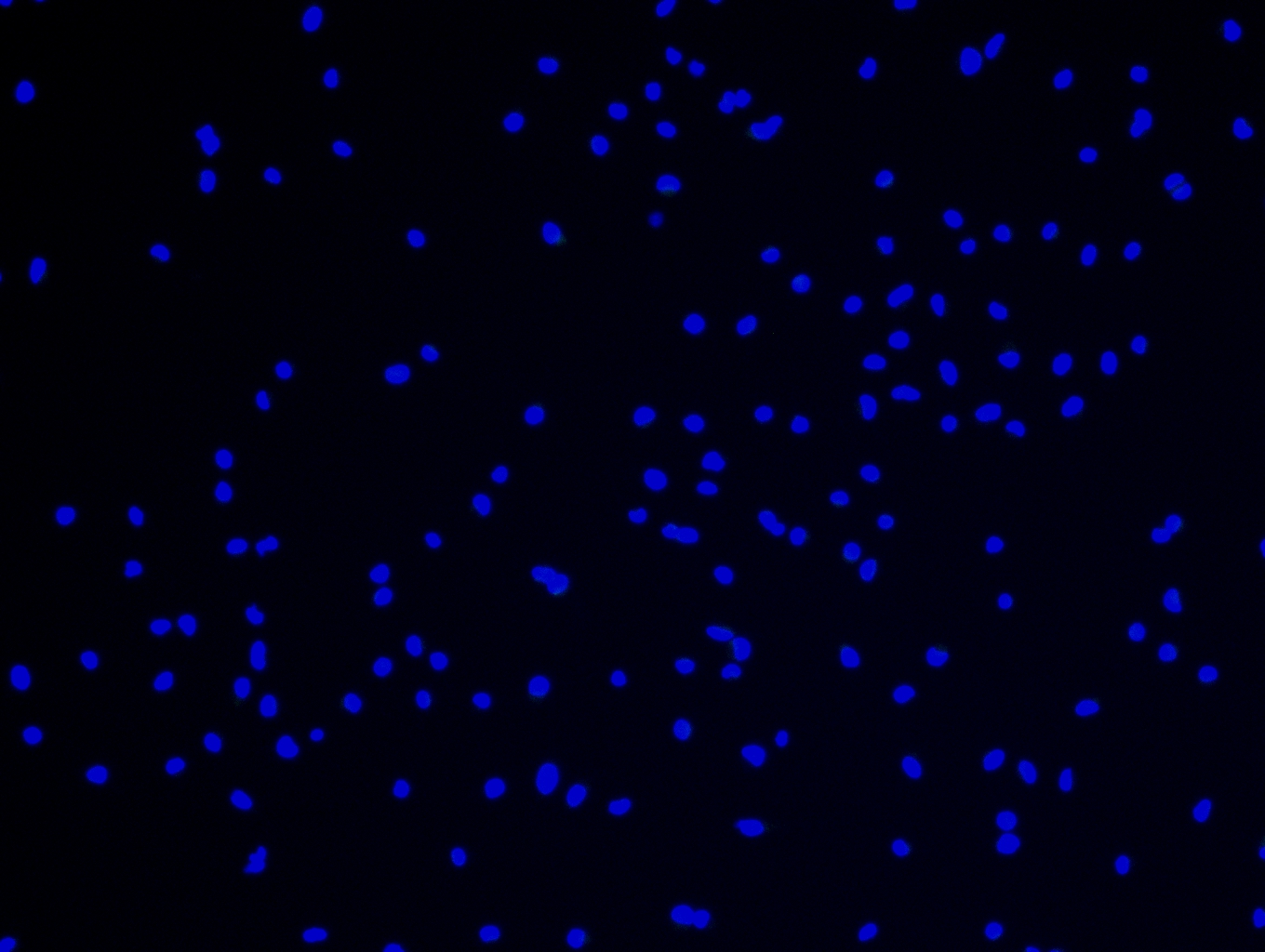

Supplement: Supplementary file 3 — Source data Fig. 1 [file 44318_2024_220_MOESM3_ESM.zip › Figure1/1G/ESC1 (2).jpg]

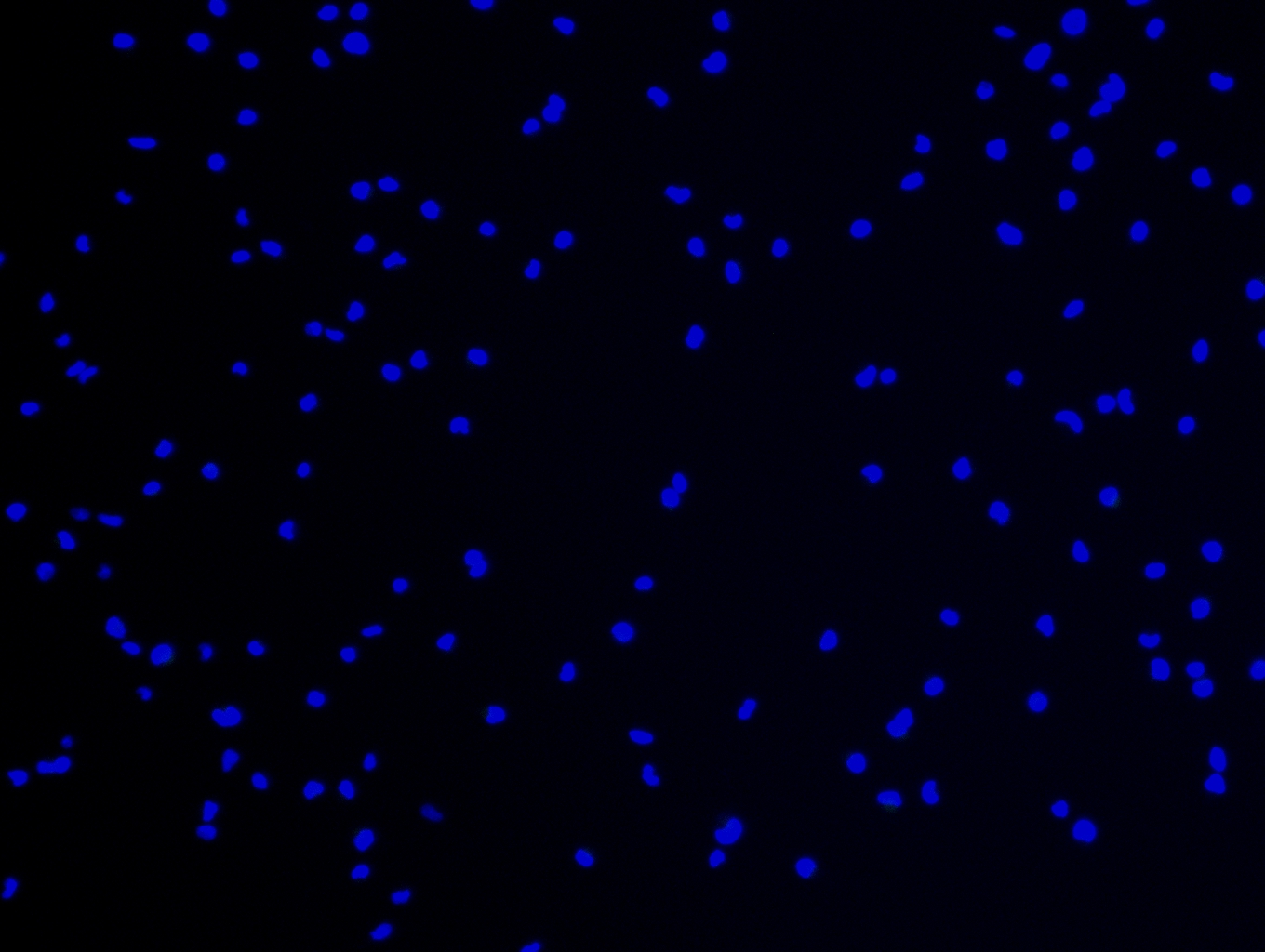

Supplement: Supplementary file 3 — Source data Fig. 1 [file 44318_2024_220_MOESM3_ESM.zip › Figure1/1G/ESC1 (3).jpg]

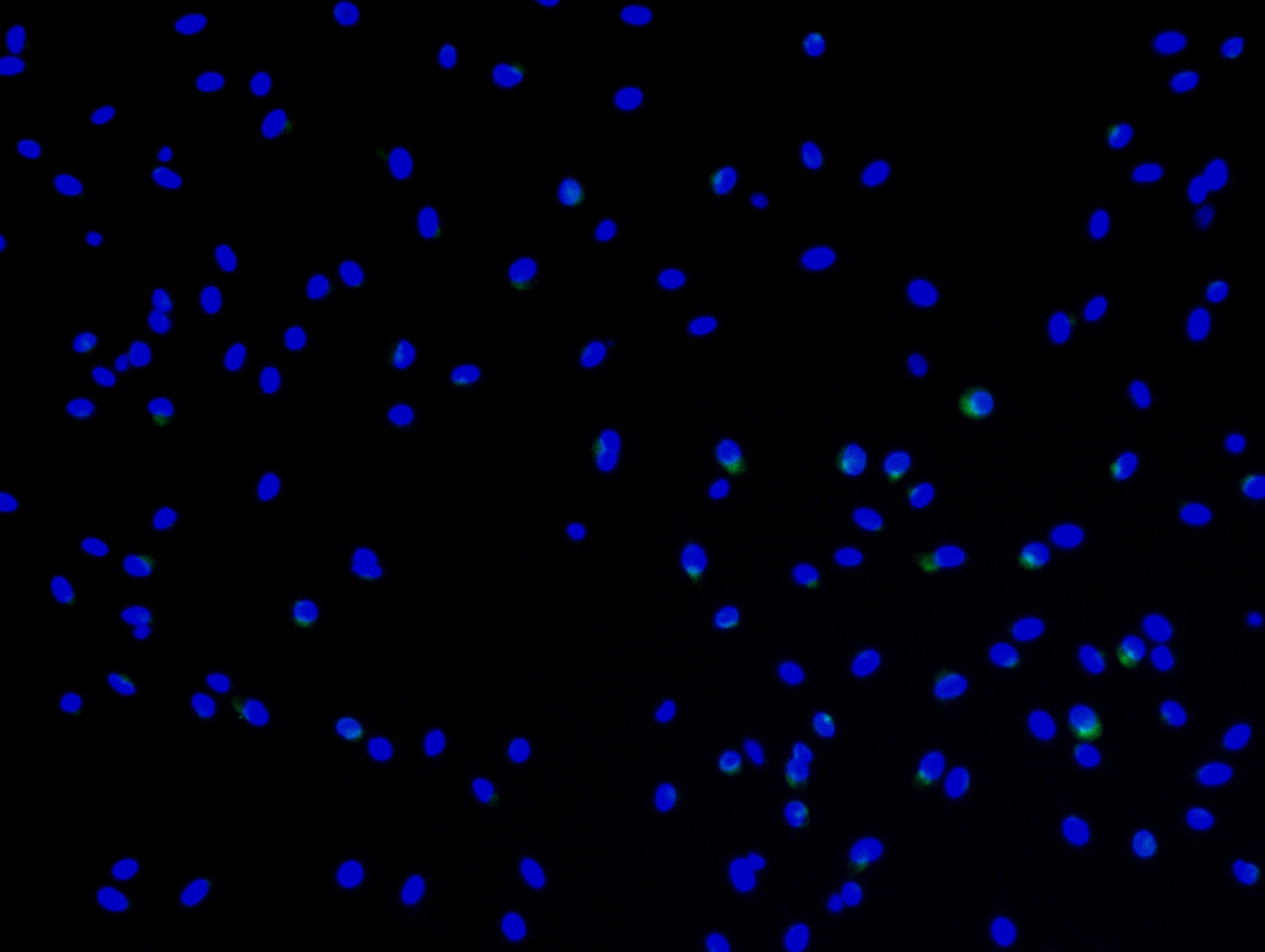

Supplement: Supplementary file 3 — Source data Fig. 1 [file 44318_2024_220_MOESM3_ESM.zip › Figure1/1G/ESC2(1).jpg]

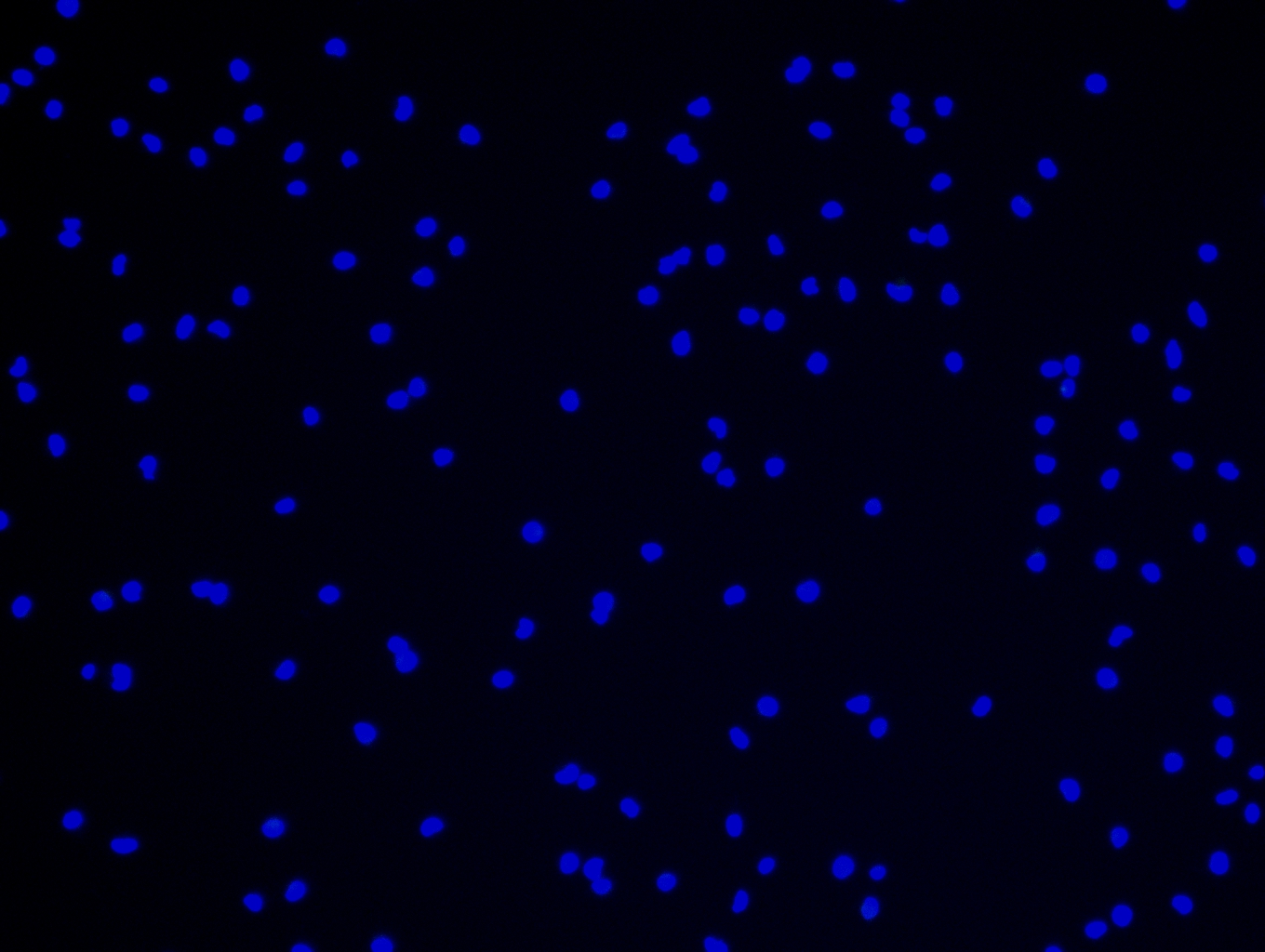

Supplement: Supplementary file 3 — Source data Fig. 1 [file 44318_2024_220_MOESM3_ESM.zip › Figure1/1G/ESC2(2).jpg]

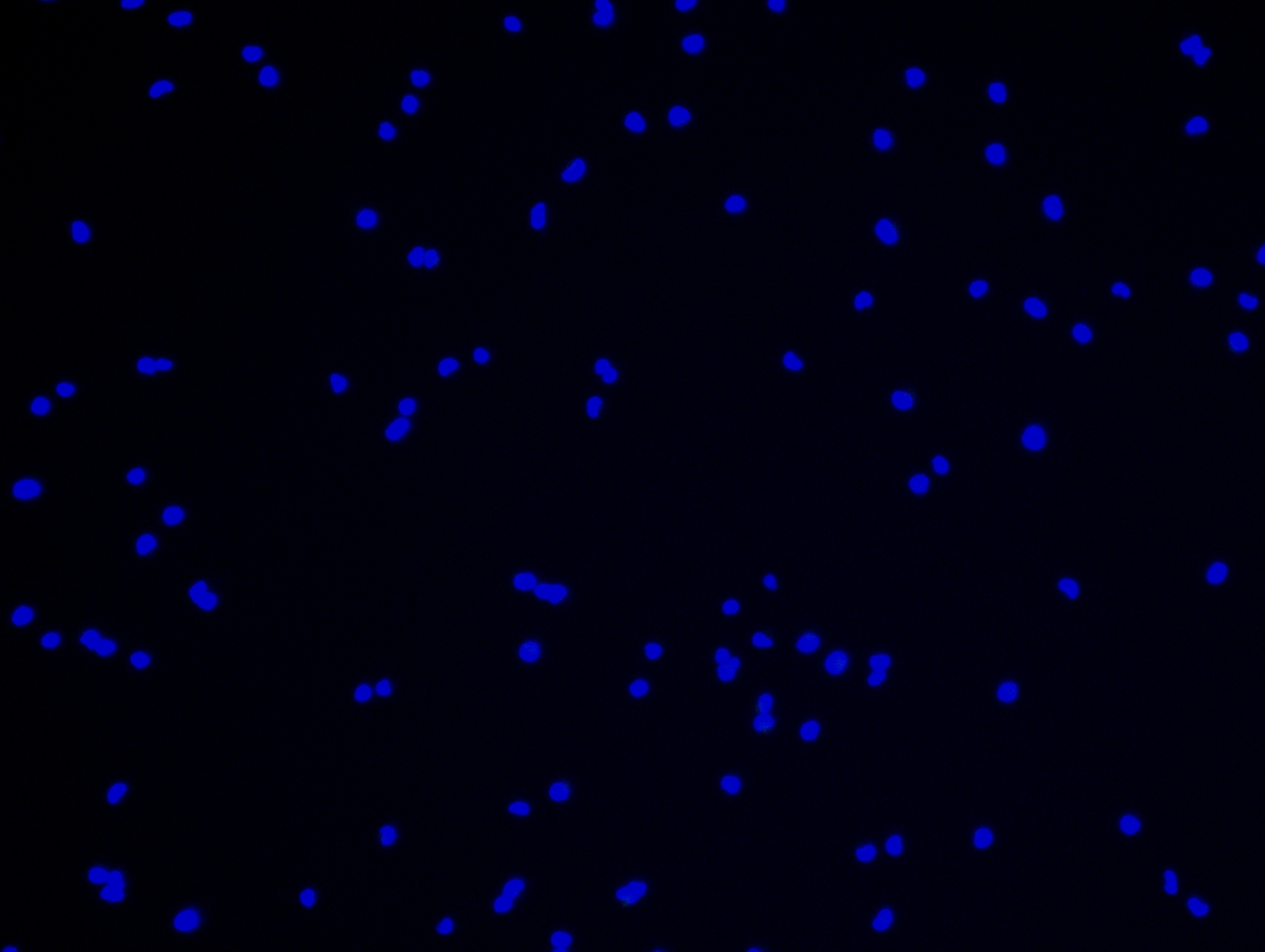

Supplement: Supplementary file 3 — Source data Fig. 1 [file 44318_2024_220_MOESM3_ESM.zip › Figure1/1G/ESC2(3).jpg]

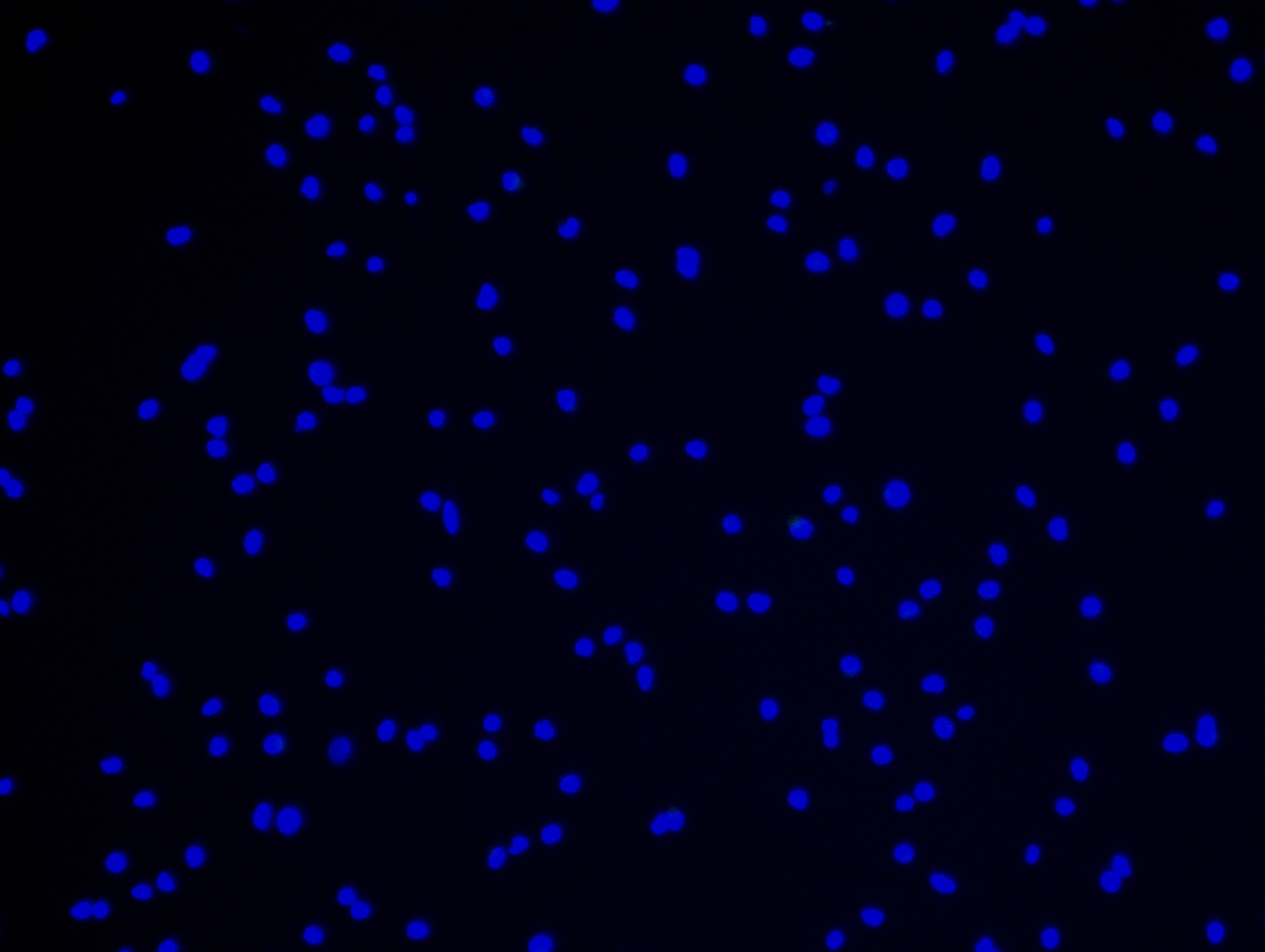

Supplement: Supplementary file 3 — Source data Fig. 1 [file 44318_2024_220_MOESM3_ESM.zip › Figure1/1G/ESC3 (1).jpg]

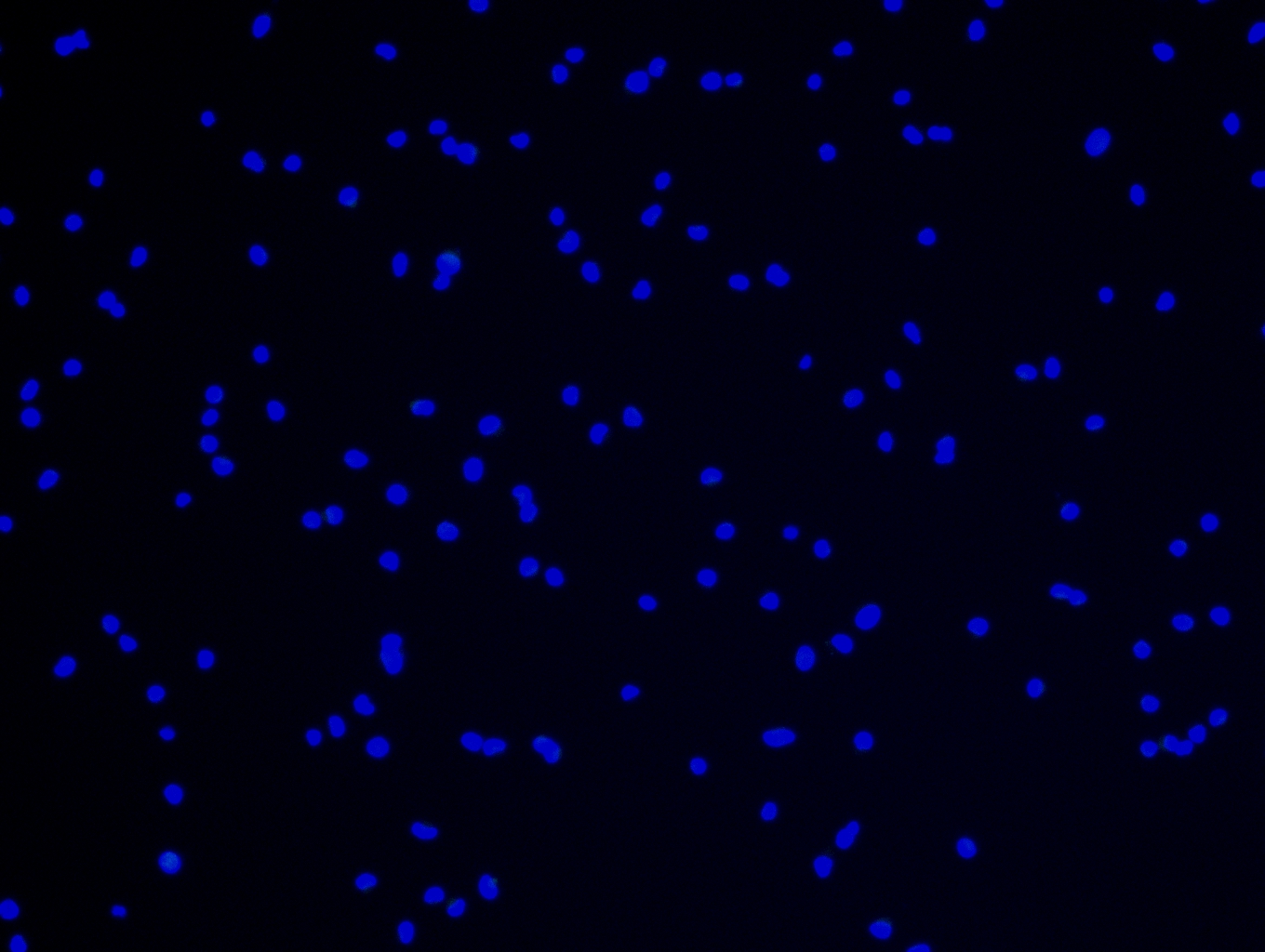

Supplement: Supplementary file 3 — Source data Fig. 1 [file 44318_2024_220_MOESM3_ESM.zip › Figure1/1G/ESC3 (2).jpg]

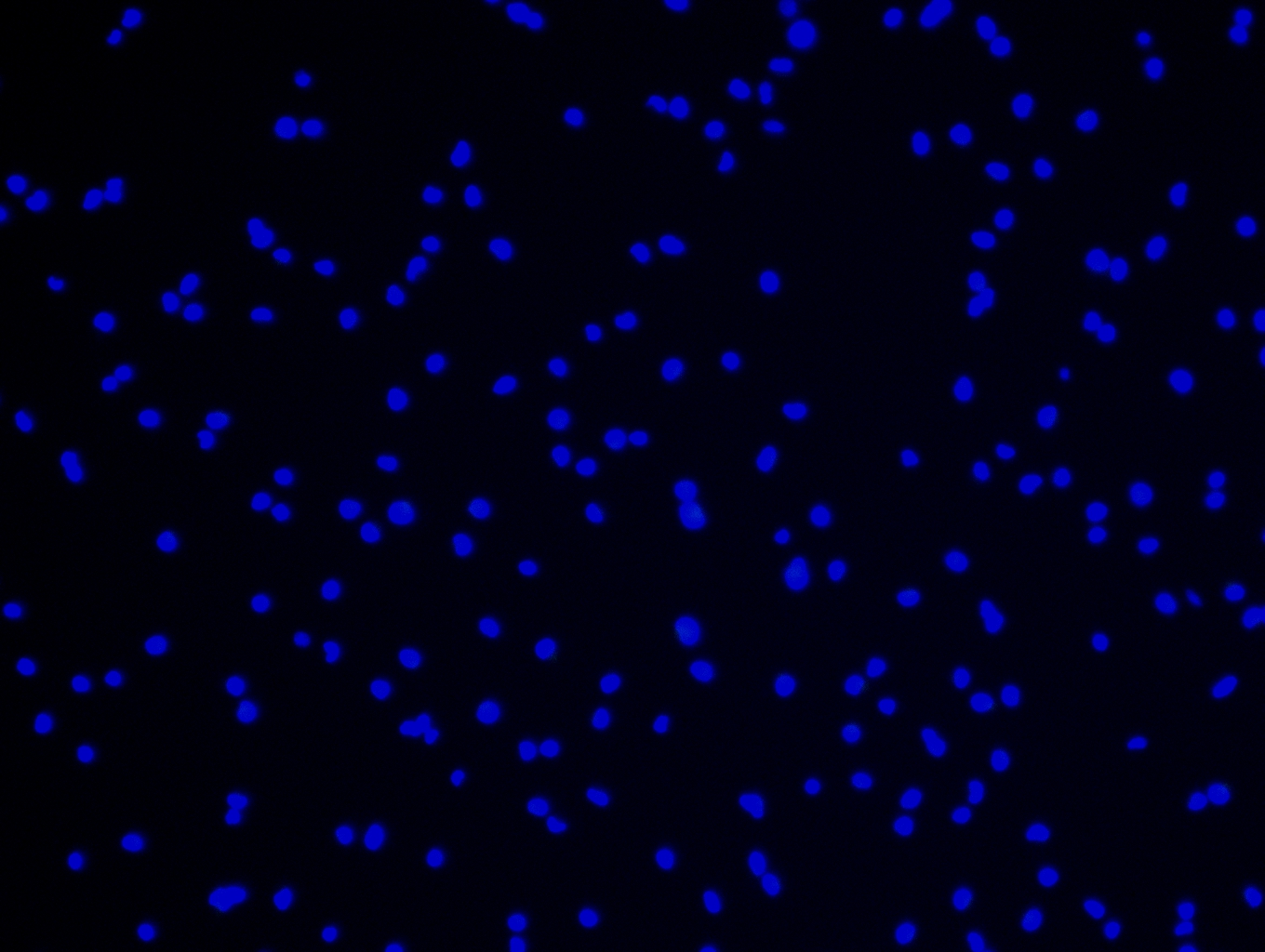

Supplement: Supplementary file 3 — Source data Fig. 1 [file 44318_2024_220_MOESM3_ESM.zip › Figure1/1G/ESC3 (3).jpg]

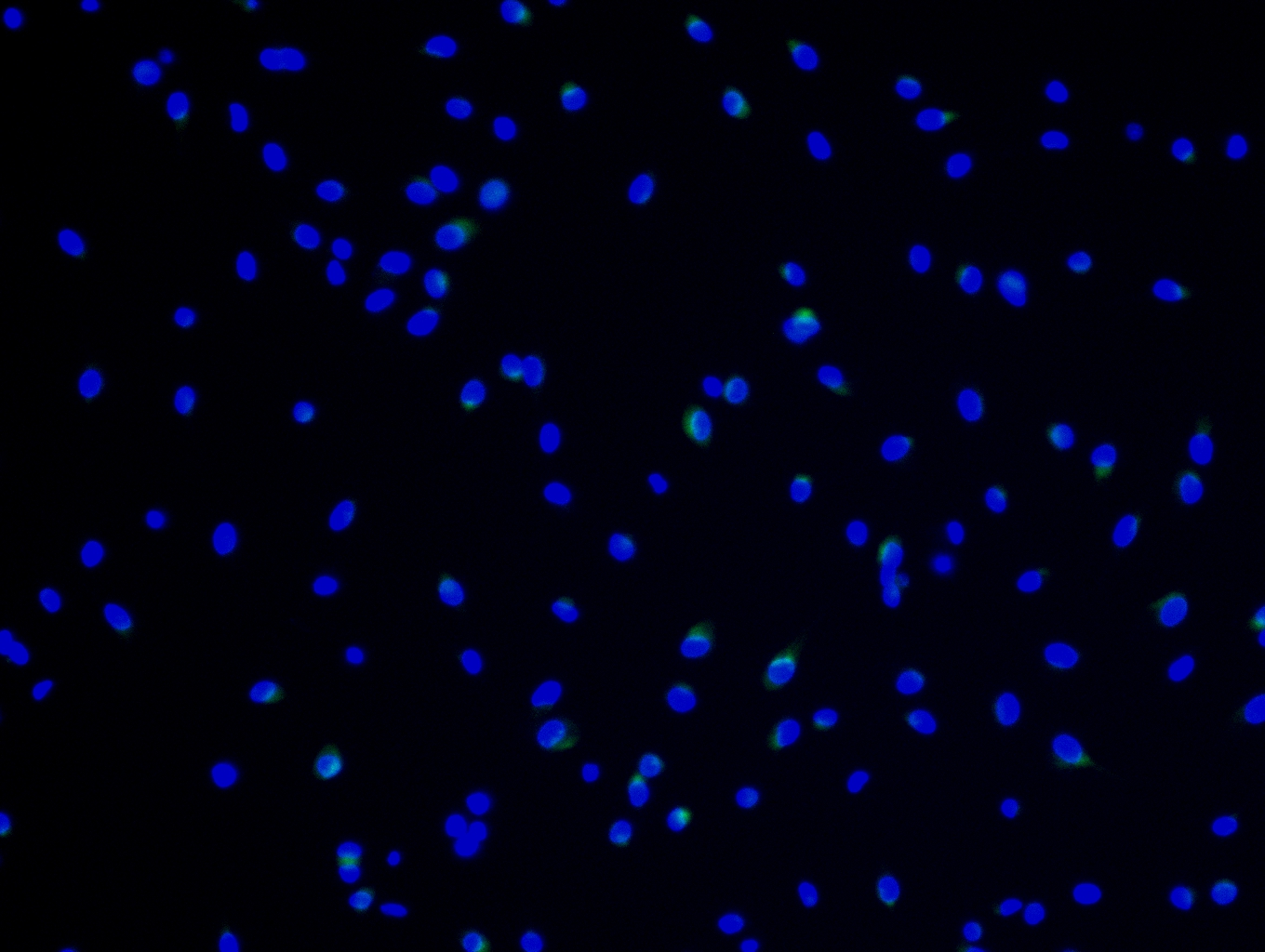

Supplement: Supplementary file 3 — Source data Fig. 1 [file 44318_2024_220_MOESM3_ESM.zip › Figure1/1G/ESC4 (1).jpg]

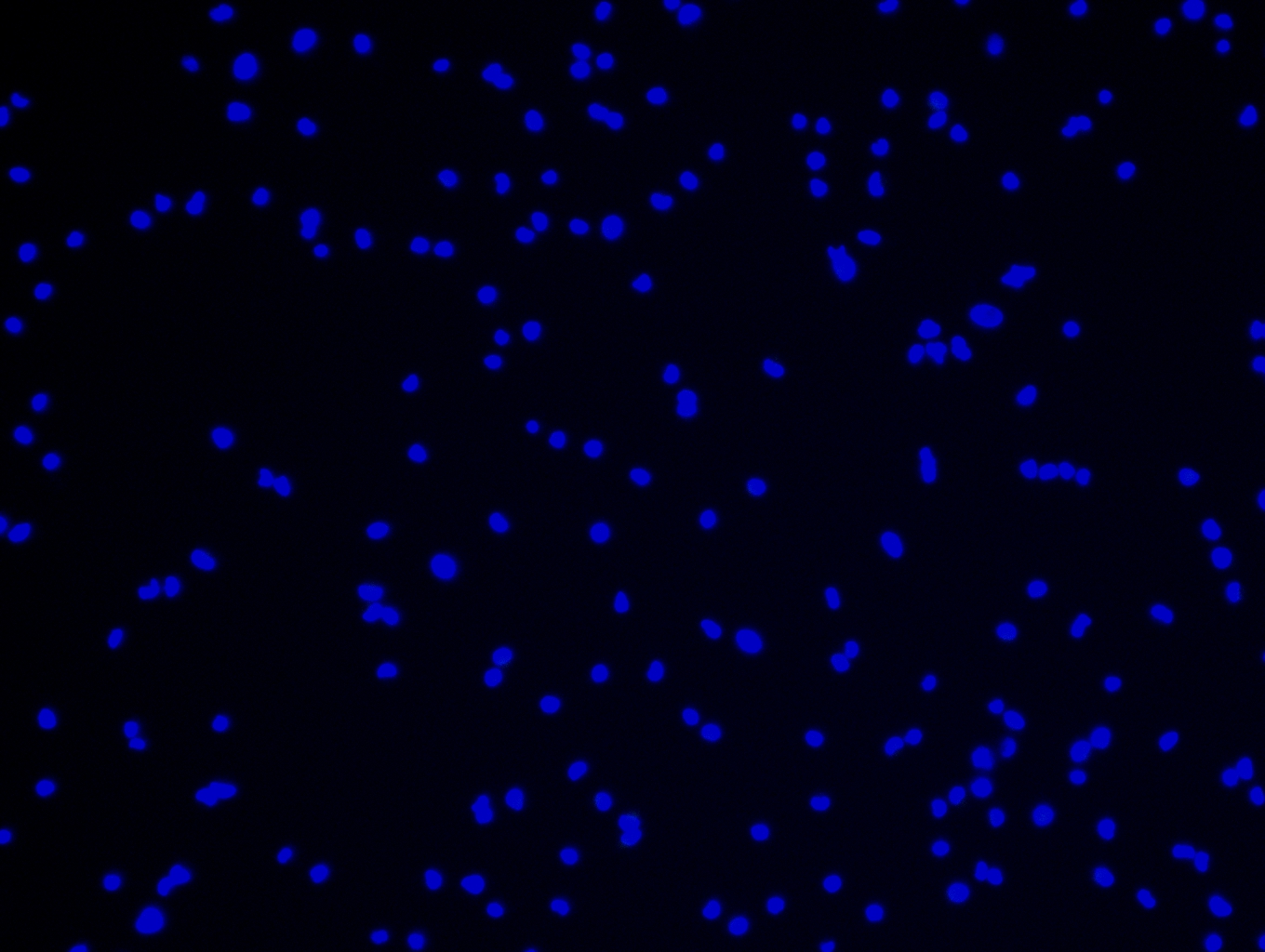

Supplement: Supplementary file 3 — Source data Fig. 1 [file 44318_2024_220_MOESM3_ESM.zip › Figure1/1G/ESC4 (2).jpg]

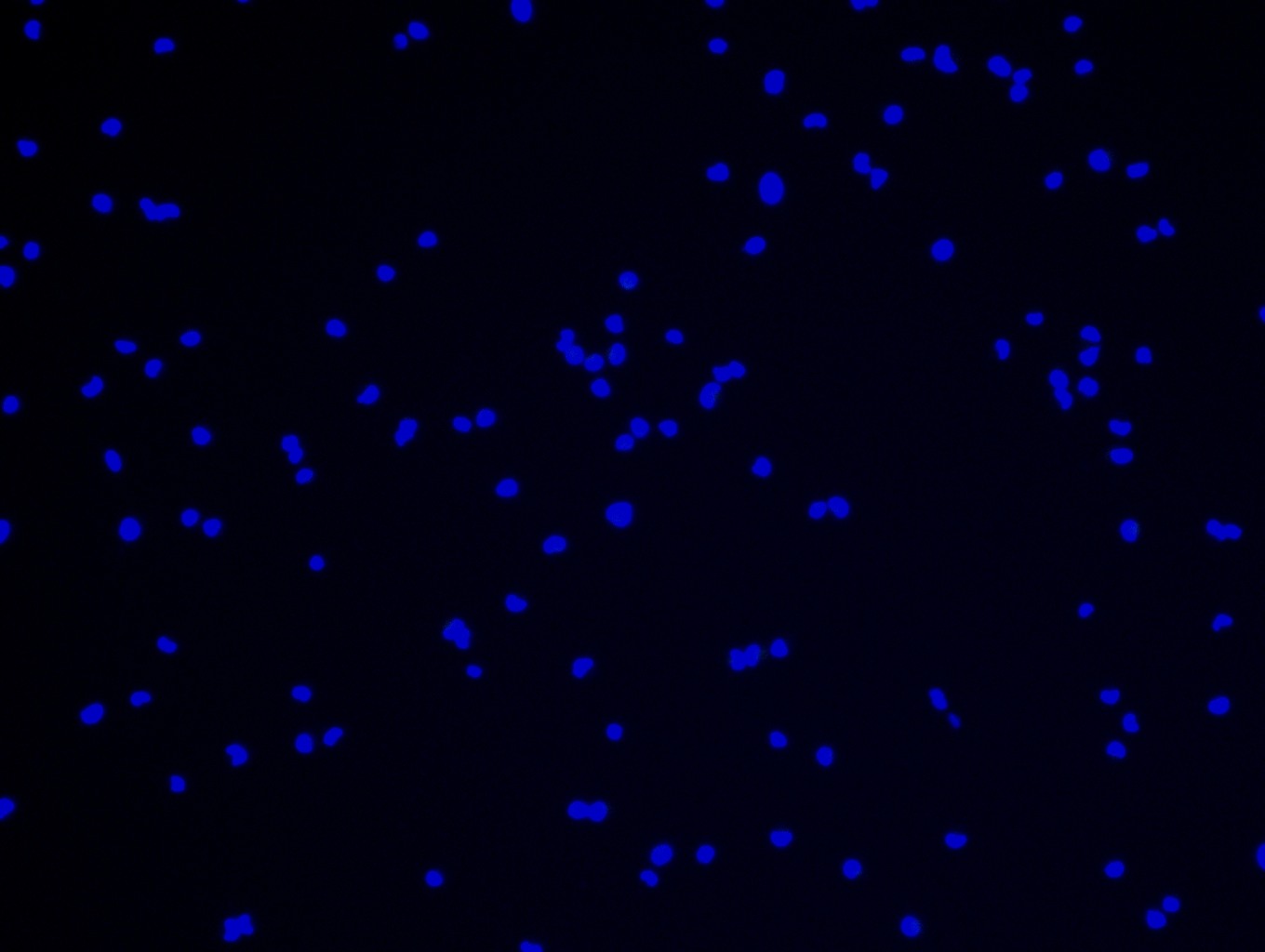

Supplement: Supplementary file 3 — Source data Fig. 1 [file 44318_2024_220_MOESM3_ESM.zip › Figure1/1G/ESC4 (3).jpg]

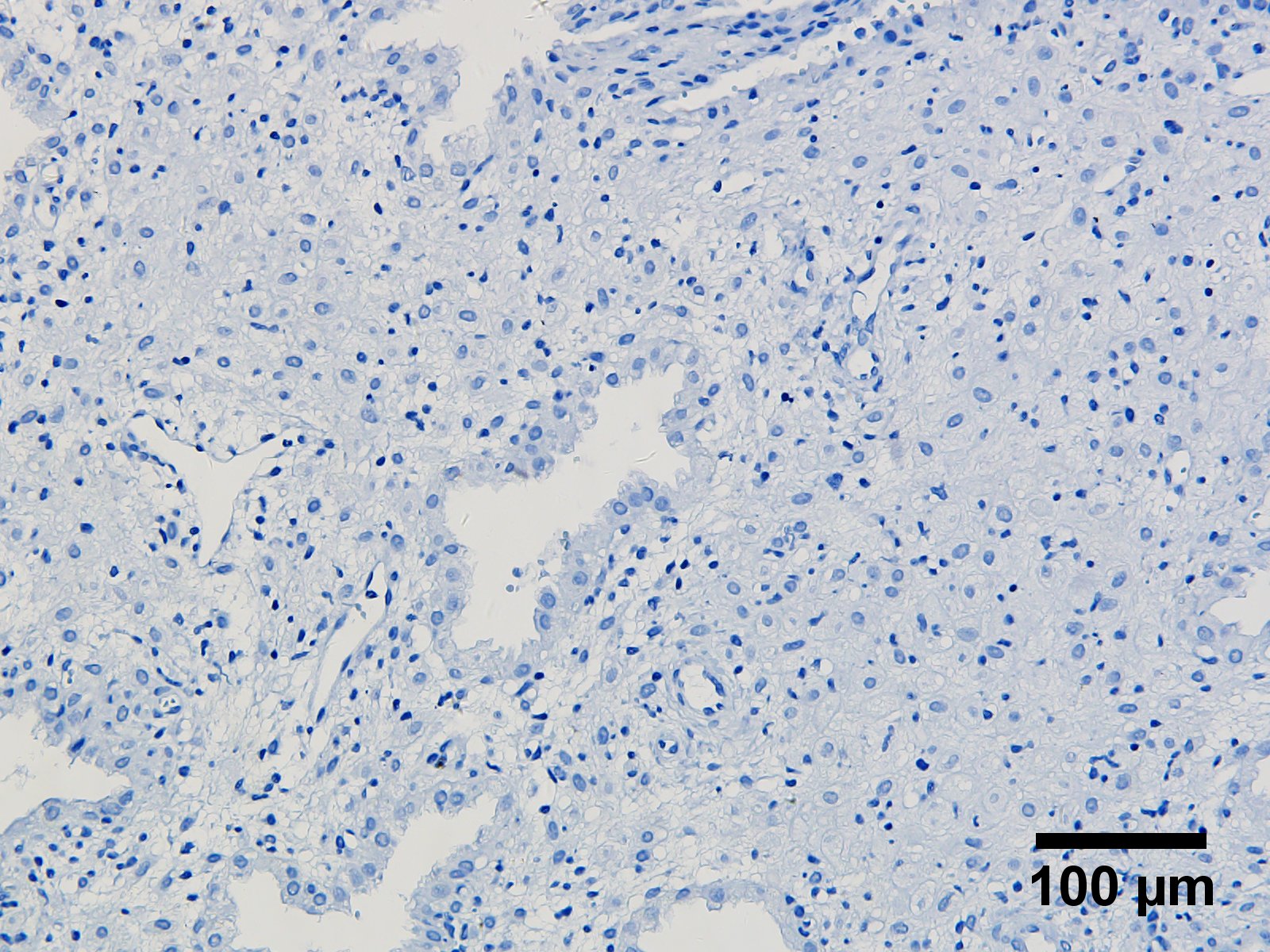

Supplement: Supplementary file 4 — Source data Fig. 2 [file 44318_2024_220_MOESM4_ESM.zip › Figure2/2A/CDKN1A-BLANK-200.jpg]

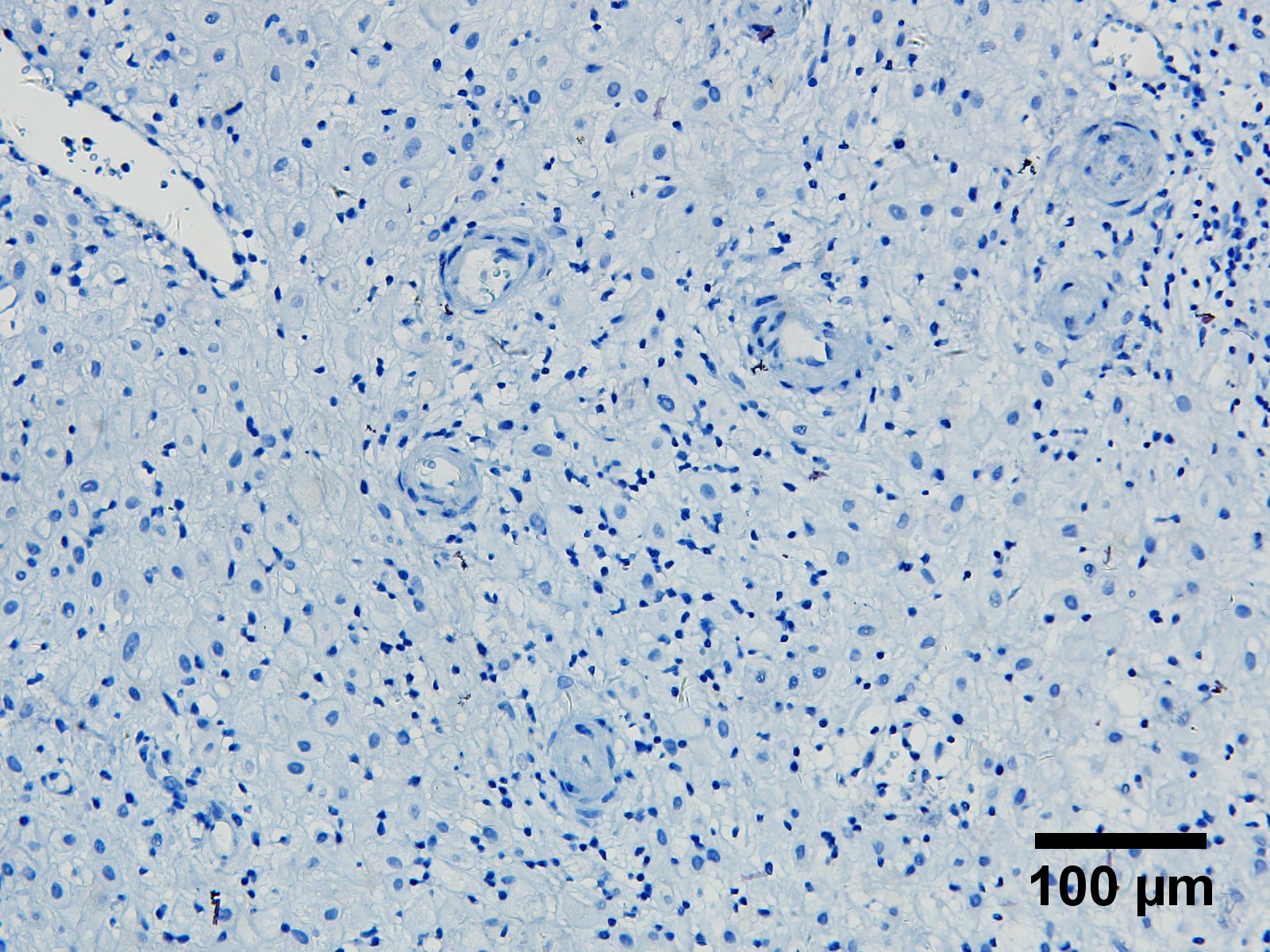

Supplement: Supplementary file 4 — Source data Fig. 2 [file 44318_2024_220_MOESM4_ESM.zip › Figure2/2A/CDKN2A-BLANK-200.jpg]

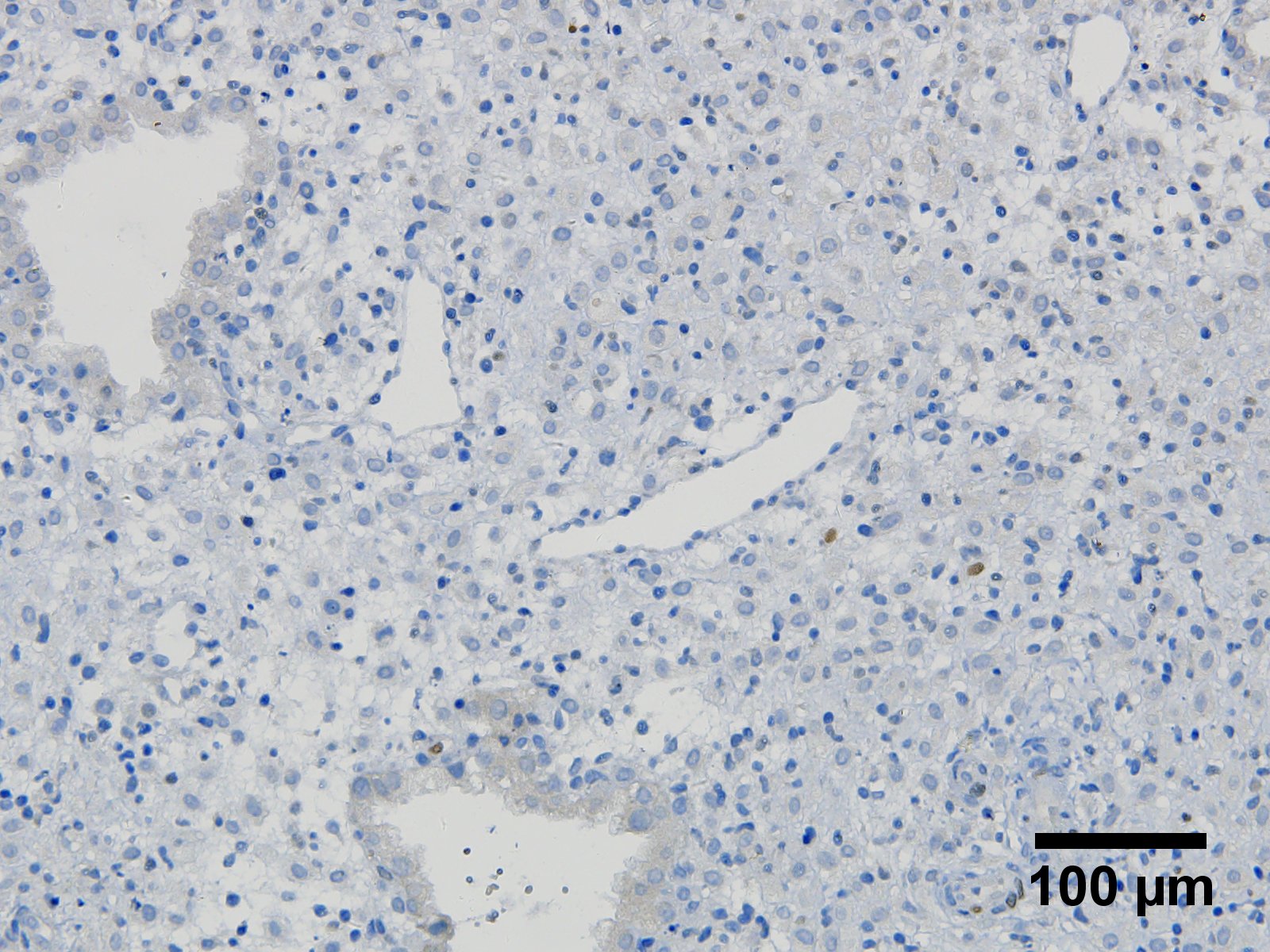

Supplement: Supplementary file 4 — Source data Fig. 2 [file 44318_2024_220_MOESM4_ESM.zip › Figure2/2A/NP-CDKN1A-200-1.jpg]

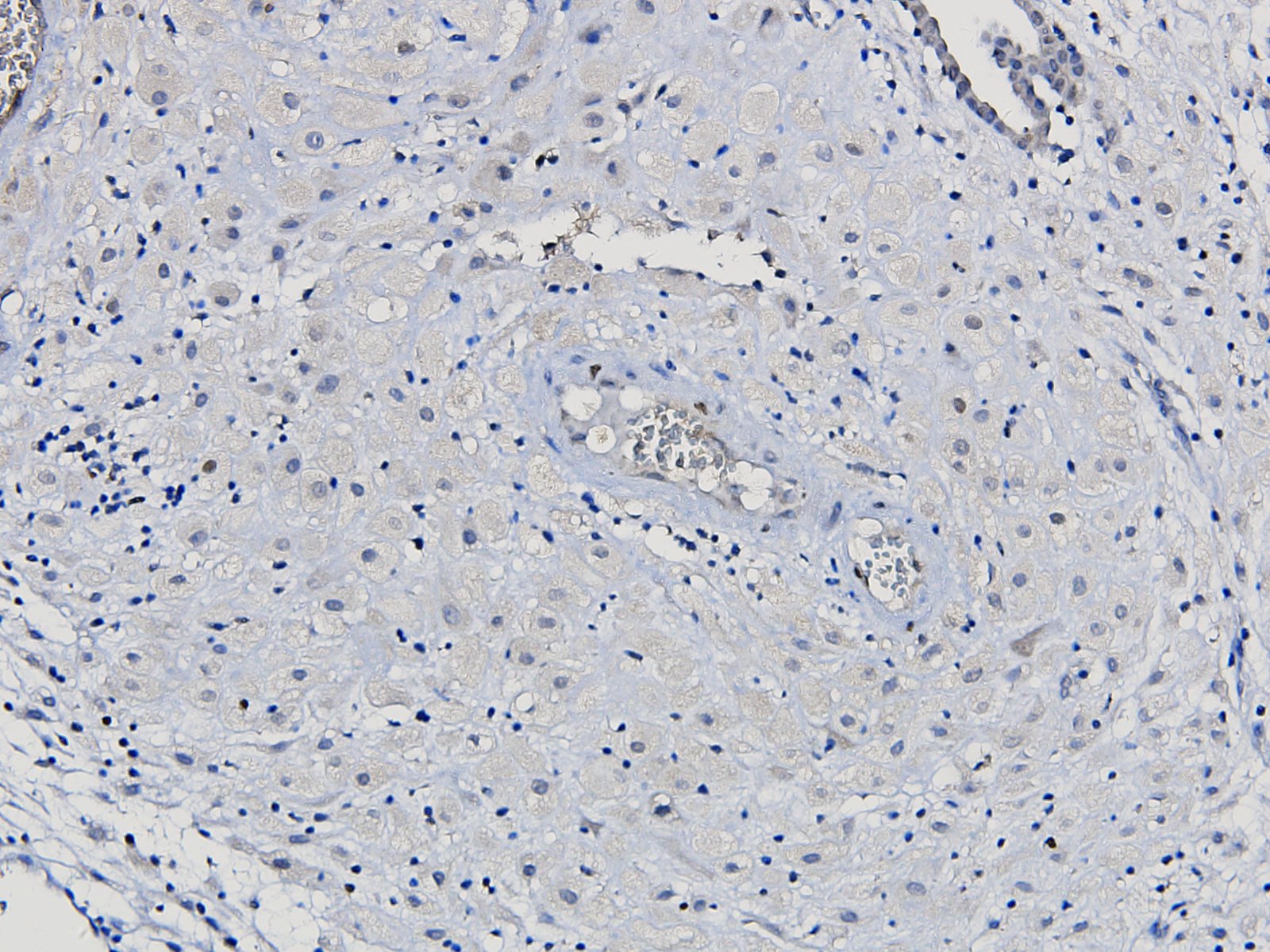

Supplement: Supplementary file 4 — Source data Fig. 2 [file 44318_2024_220_MOESM4_ESM.zip › Figure2/2A/NP-CDKN1A-200-2.jpg]

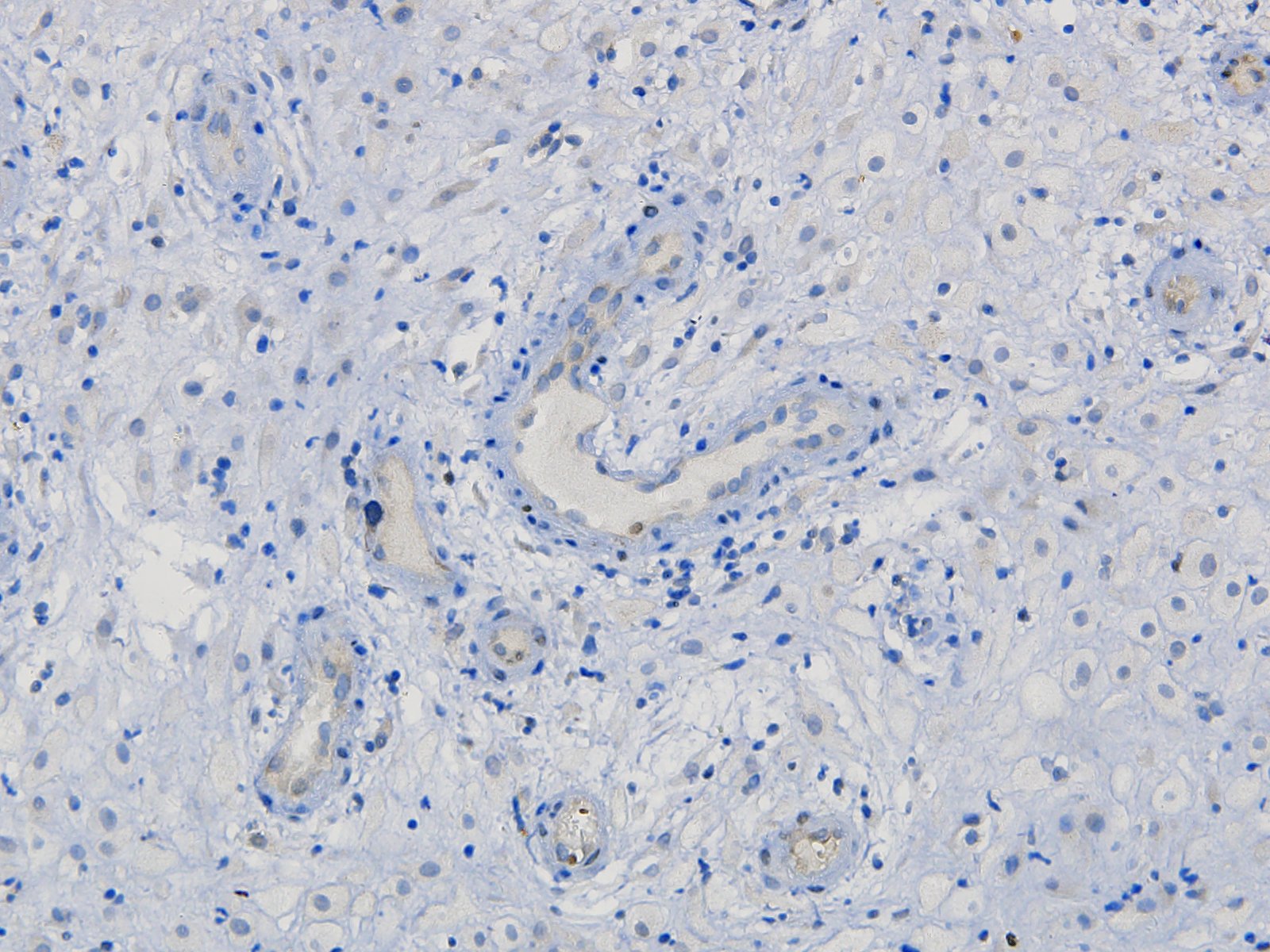

Supplement: Supplementary file 4 — Source data Fig. 2 [file 44318_2024_220_MOESM4_ESM.zip › Figure2/2A/NP-CDKN1A-200-3.jpg]

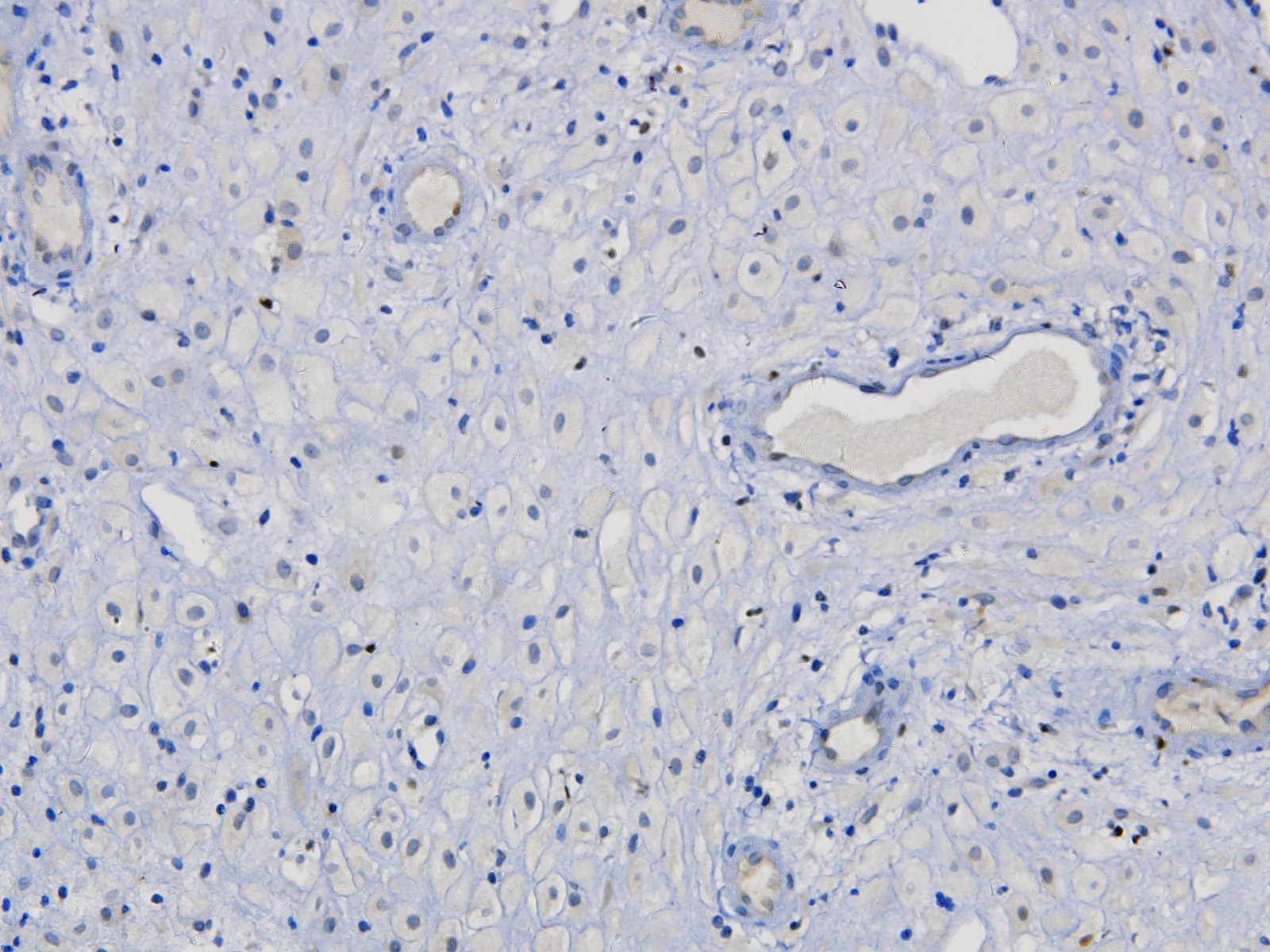

Supplement: Supplementary file 4 — Source data Fig. 2 [file 44318_2024_220_MOESM4_ESM.zip › Figure2/2A/NP-CDKN1A-200-4.jpg]

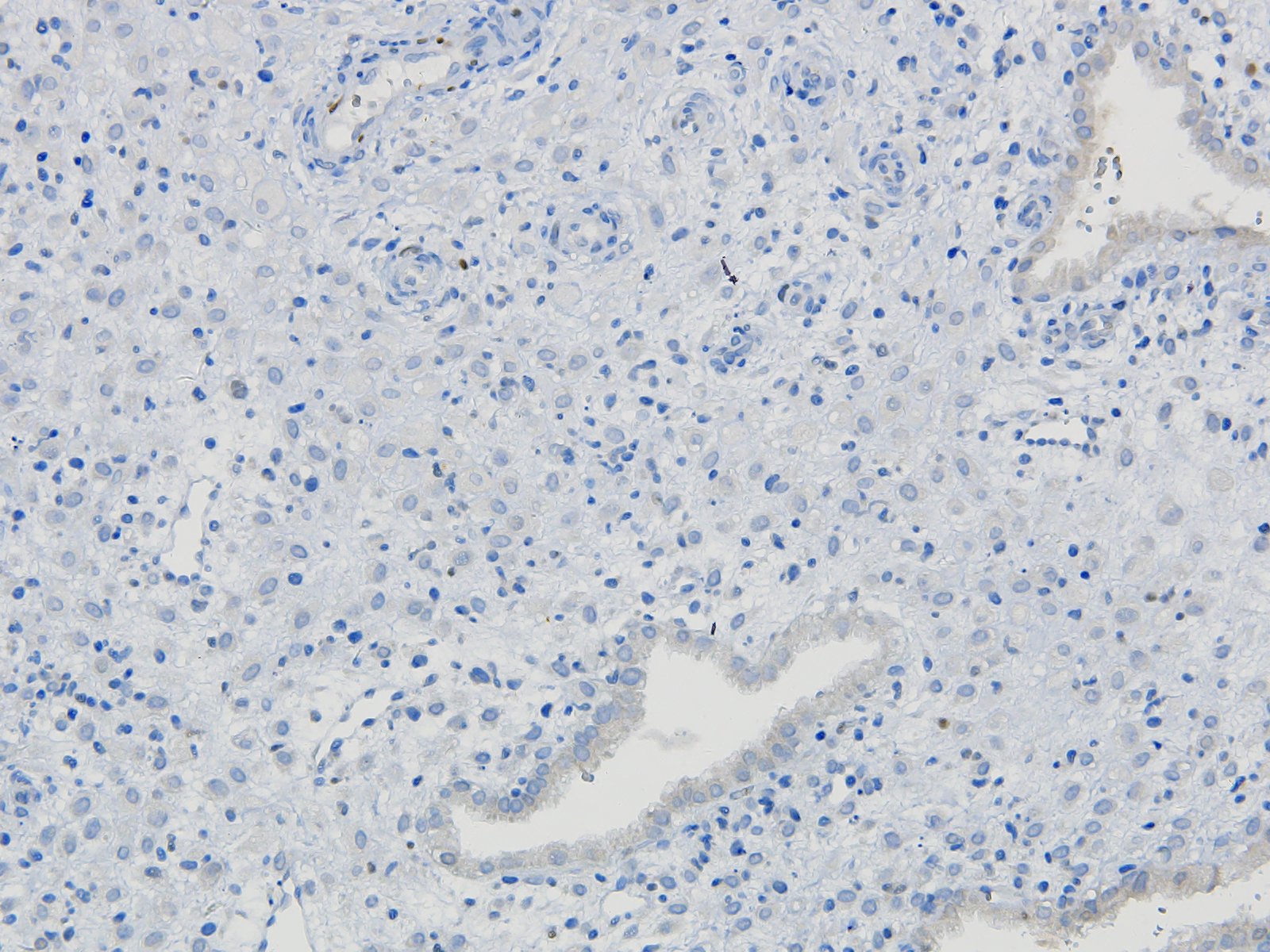

Supplement: Supplementary file 4 — Source data Fig. 2 [file 44318_2024_220_MOESM4_ESM.zip › Figure2/2A/NP-CDKN1A-200-5.jpg]

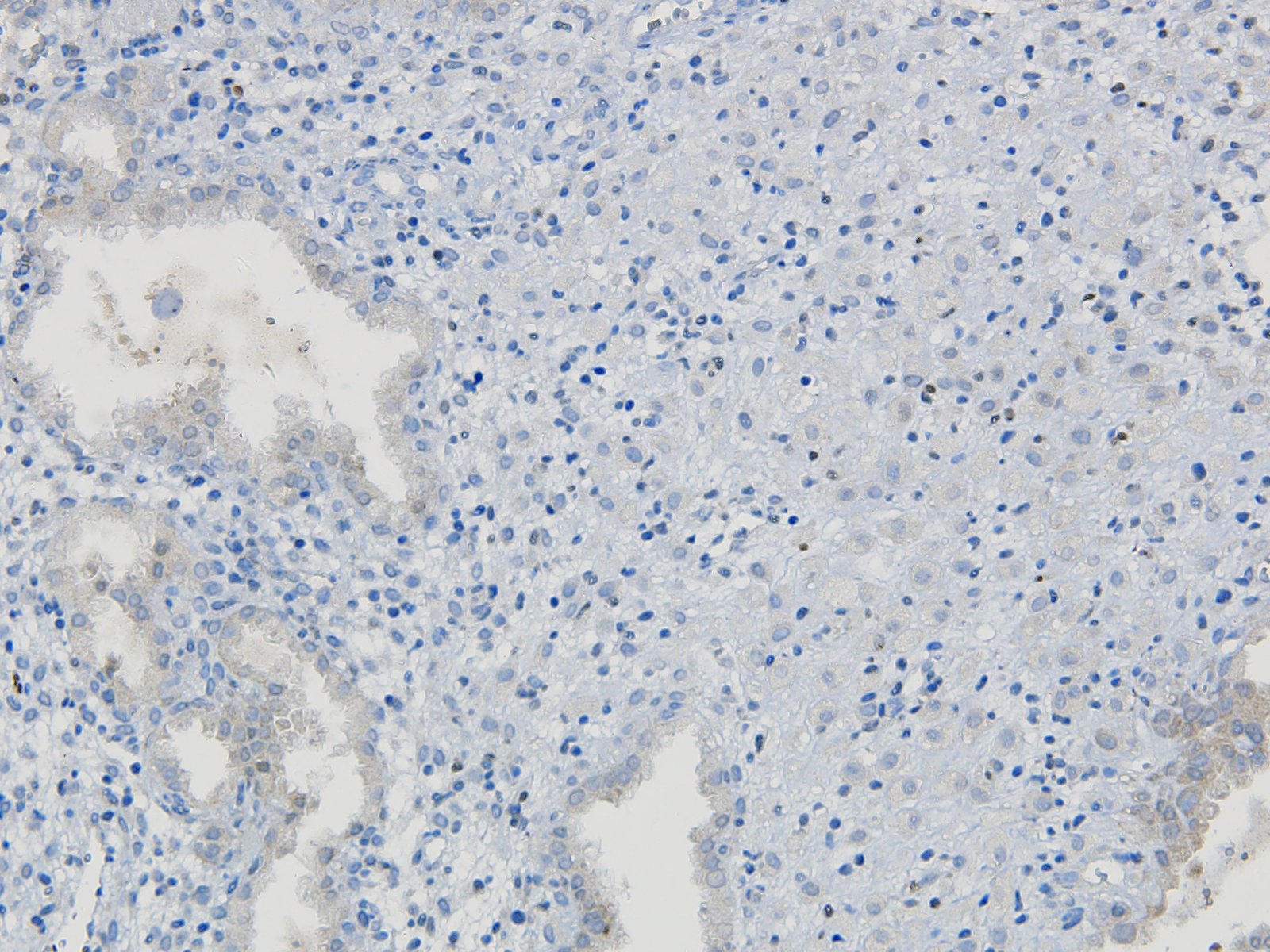

Supplement: Supplementary file 4 — Source data Fig. 2 [file 44318_2024_220_MOESM4_ESM.zip › Figure2/2A/NP-CDKN1A-200-6.jpg]

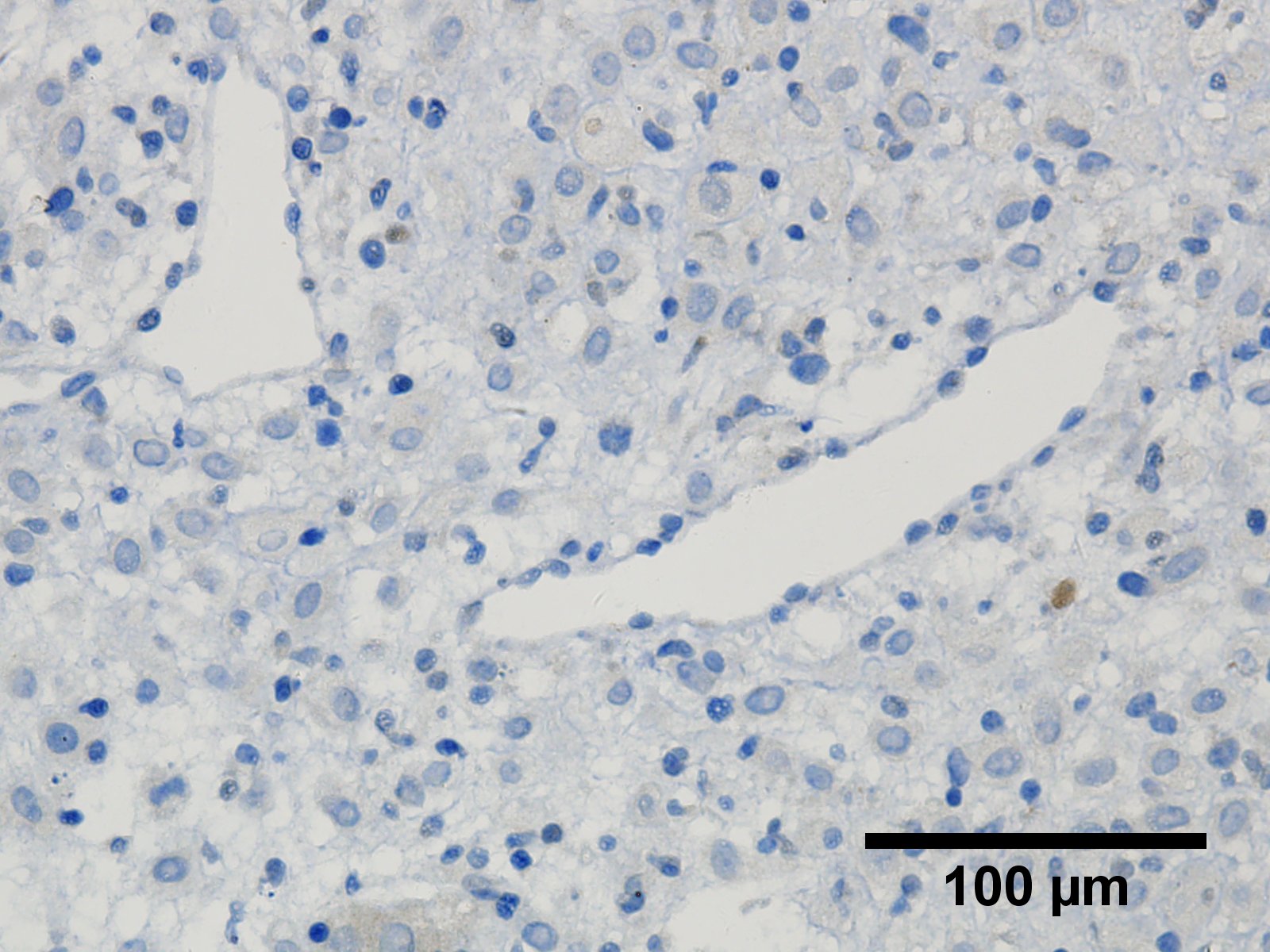

Supplement: Supplementary file 4 — Source data Fig. 2 [file 44318_2024_220_MOESM4_ESM.zip › Figure2/2A/NP-CDKN1A-400-1.jpg]

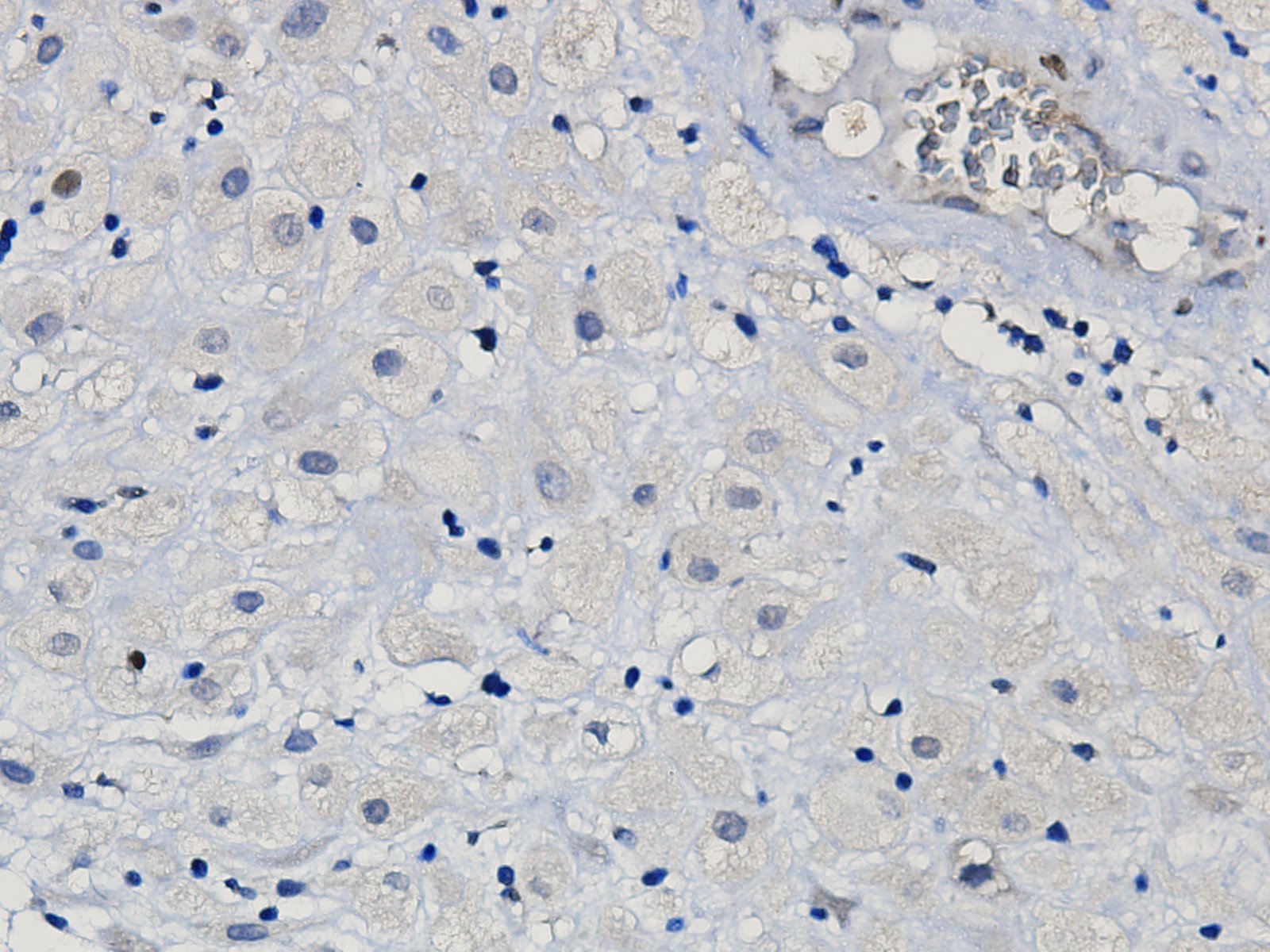

Supplement: Supplementary file 4 — Source data Fig. 2 [file 44318_2024_220_MOESM4_ESM.zip › Figure2/2A/NP-CDKN1A-400-2.jpg]

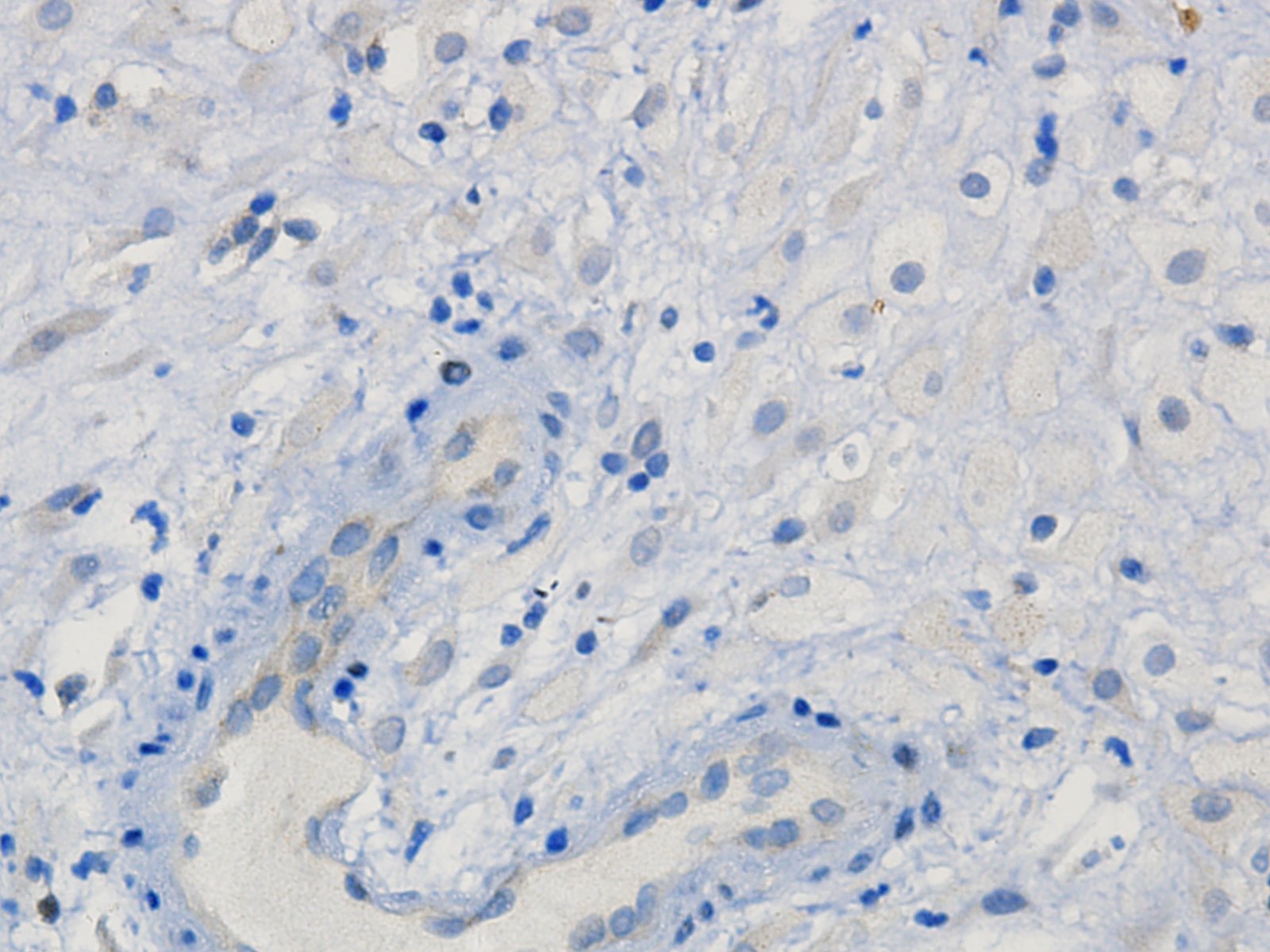

Supplement: Supplementary file 4 — Source data Fig. 2 [file 44318_2024_220_MOESM4_ESM.zip › Figure2/2A/NP-CDKN1A-400-3.jpg]

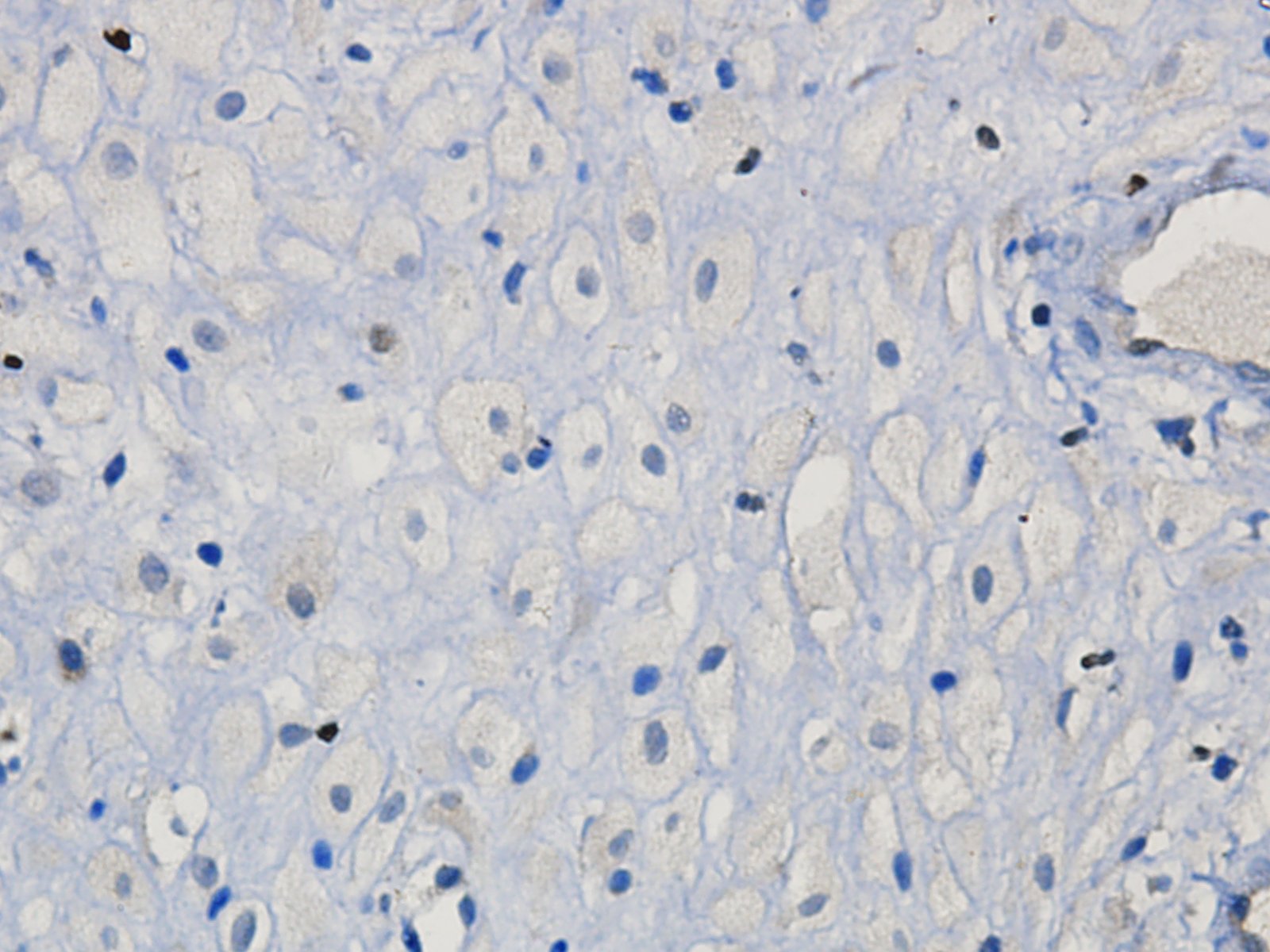

Supplement: Supplementary file 4 — Source data Fig. 2 [file 44318_2024_220_MOESM4_ESM.zip › Figure2/2A/NP-CDKN1A-400-4.jpg]

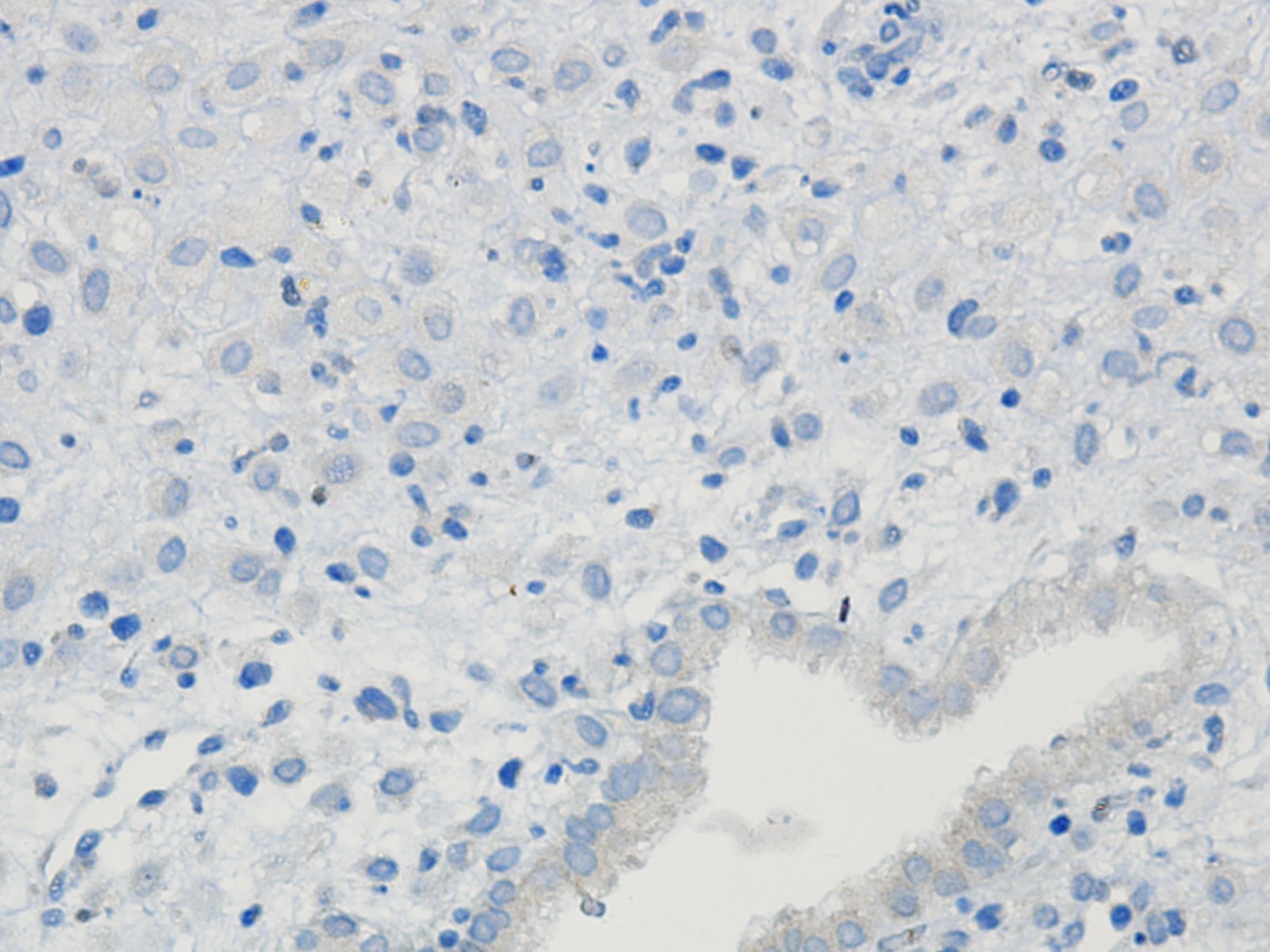

Supplement: Supplementary file 4 — Source data Fig. 2 [file 44318_2024_220_MOESM4_ESM.zip › Figure2/2A/NP-CDKN1A-400-5.jpg]

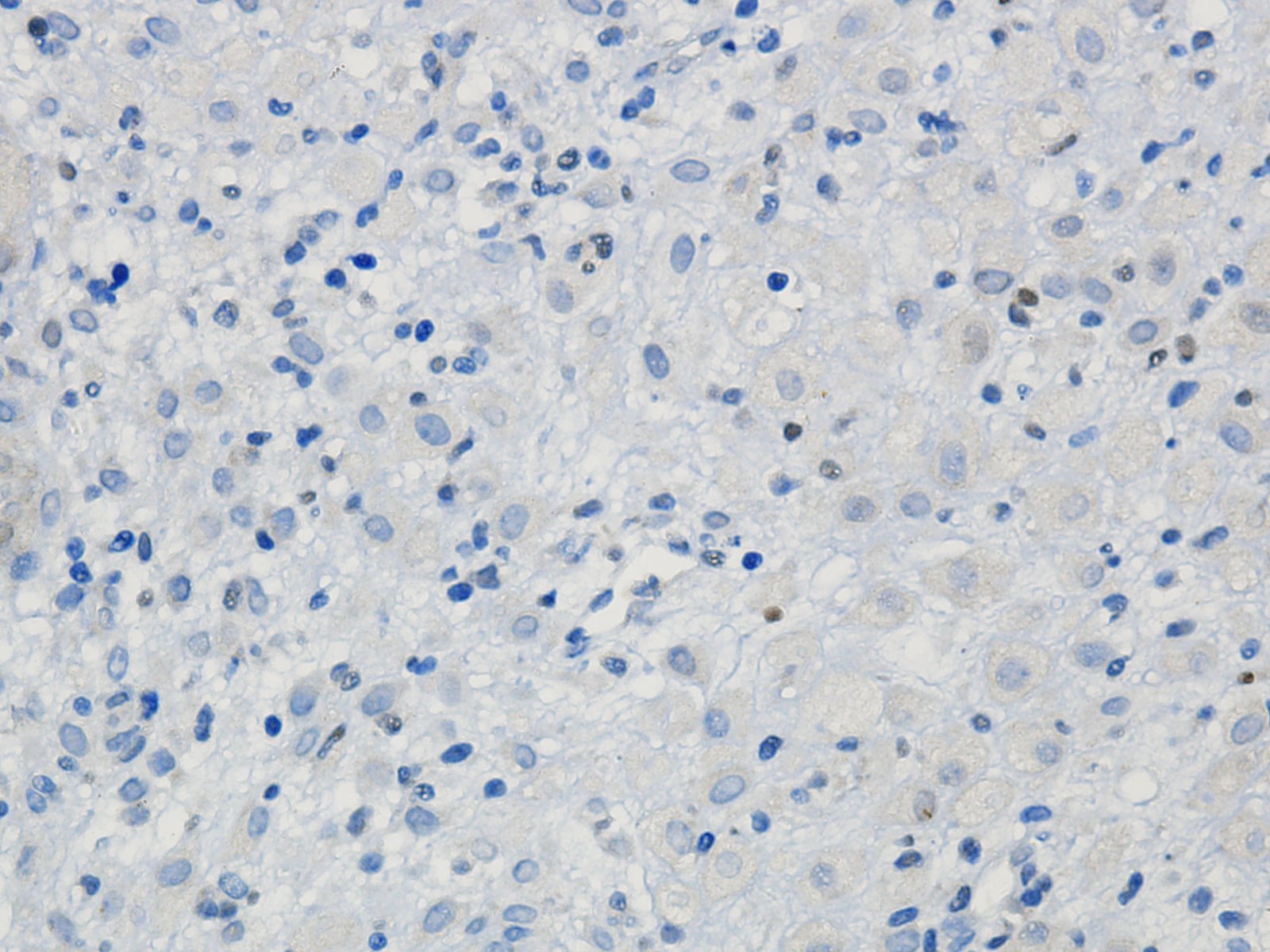

Supplement: Supplementary file 4 — Source data Fig. 2 [file 44318_2024_220_MOESM4_ESM.zip › Figure2/2A/NP-CDKN1A-400-6.jpg]

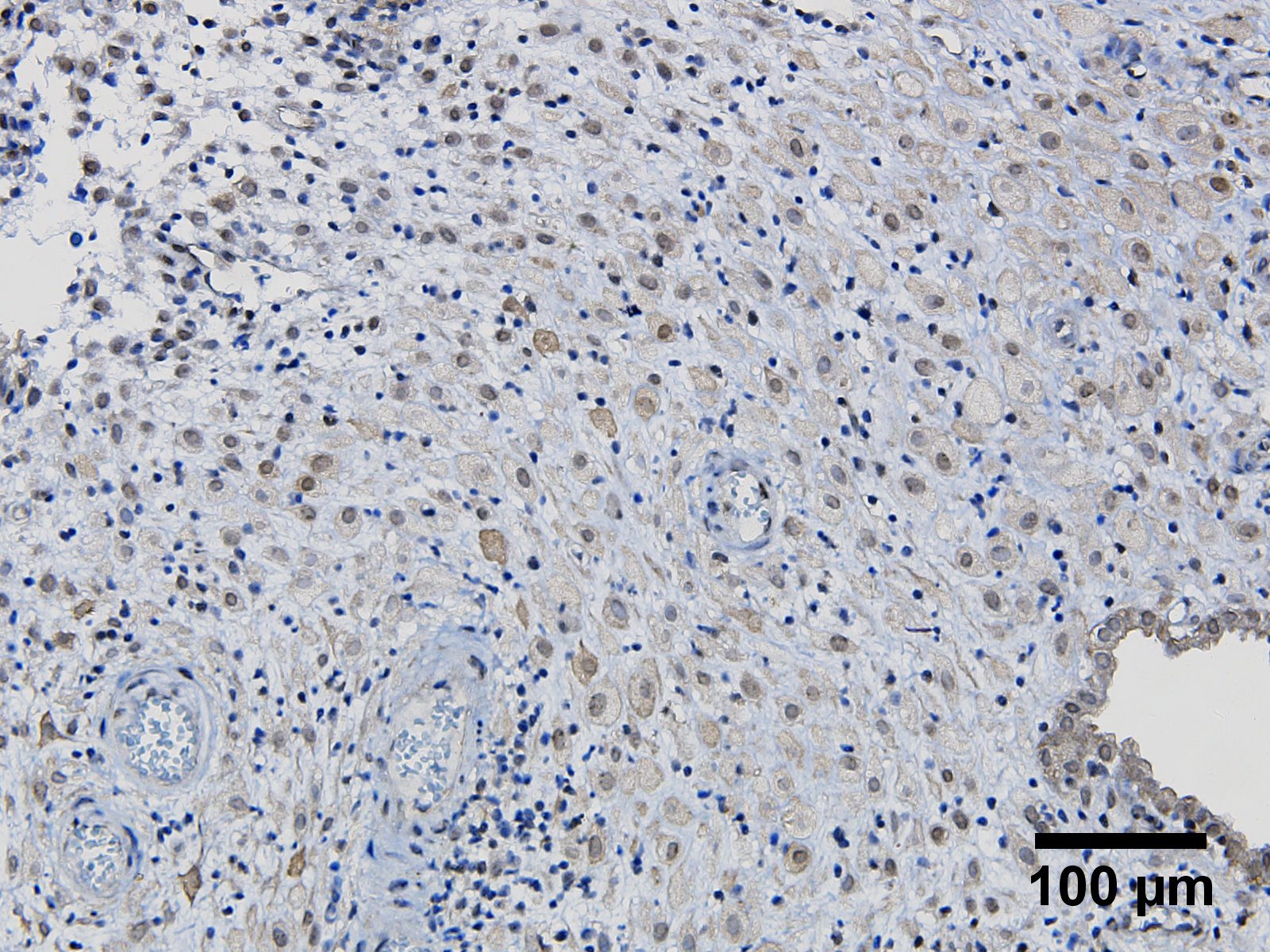

Supplement: Supplementary file 4 — Source data Fig. 2 [file 44318_2024_220_MOESM4_ESM.zip › Figure2/2A/NP-CDKN2A-200-1.jpg]

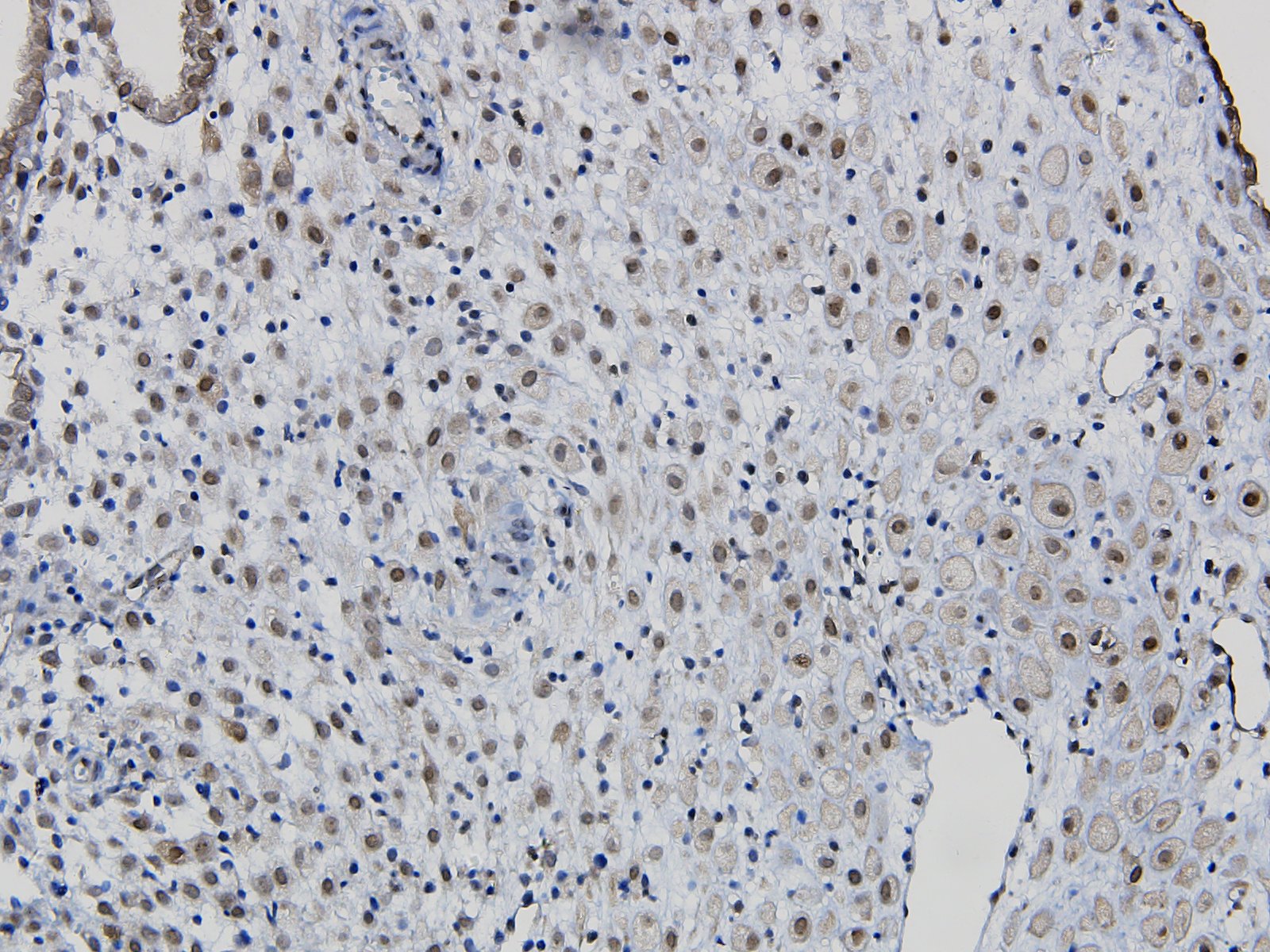

Supplement: Supplementary file 4 — Source data Fig. 2 [file 44318_2024_220_MOESM4_ESM.zip › Figure2/2A/NP-CDKN2A-200-2.jpg]

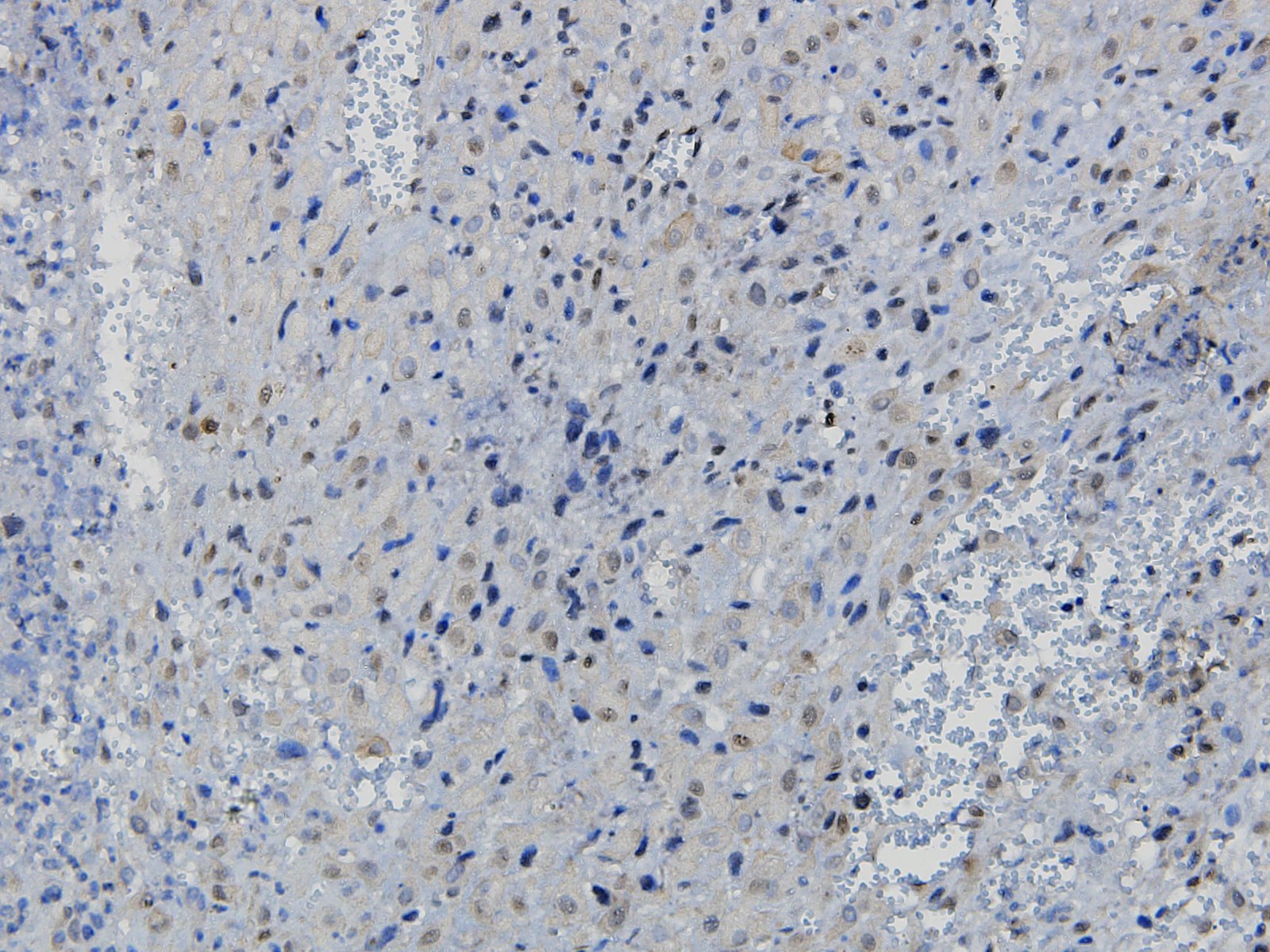

Supplement: Supplementary file 4 — Source data Fig. 2 [file 44318_2024_220_MOESM4_ESM.zip › Figure2/2A/NP-CDKN2A-200-3.jpg]

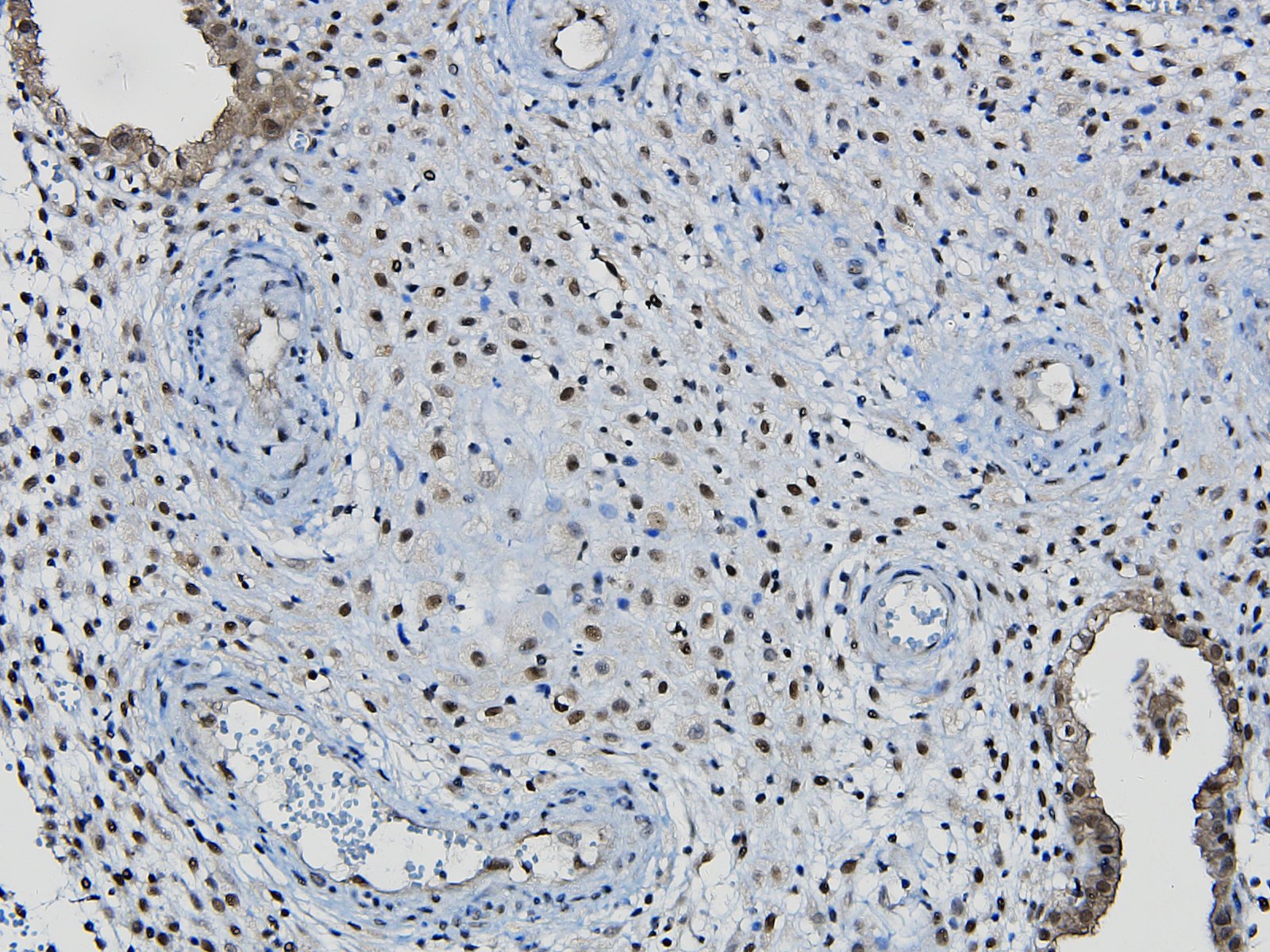

Supplement: Supplementary file 4 — Source data Fig. 2 [file 44318_2024_220_MOESM4_ESM.zip › Figure2/2A/NP-CDKN2A-200-4.jpg]

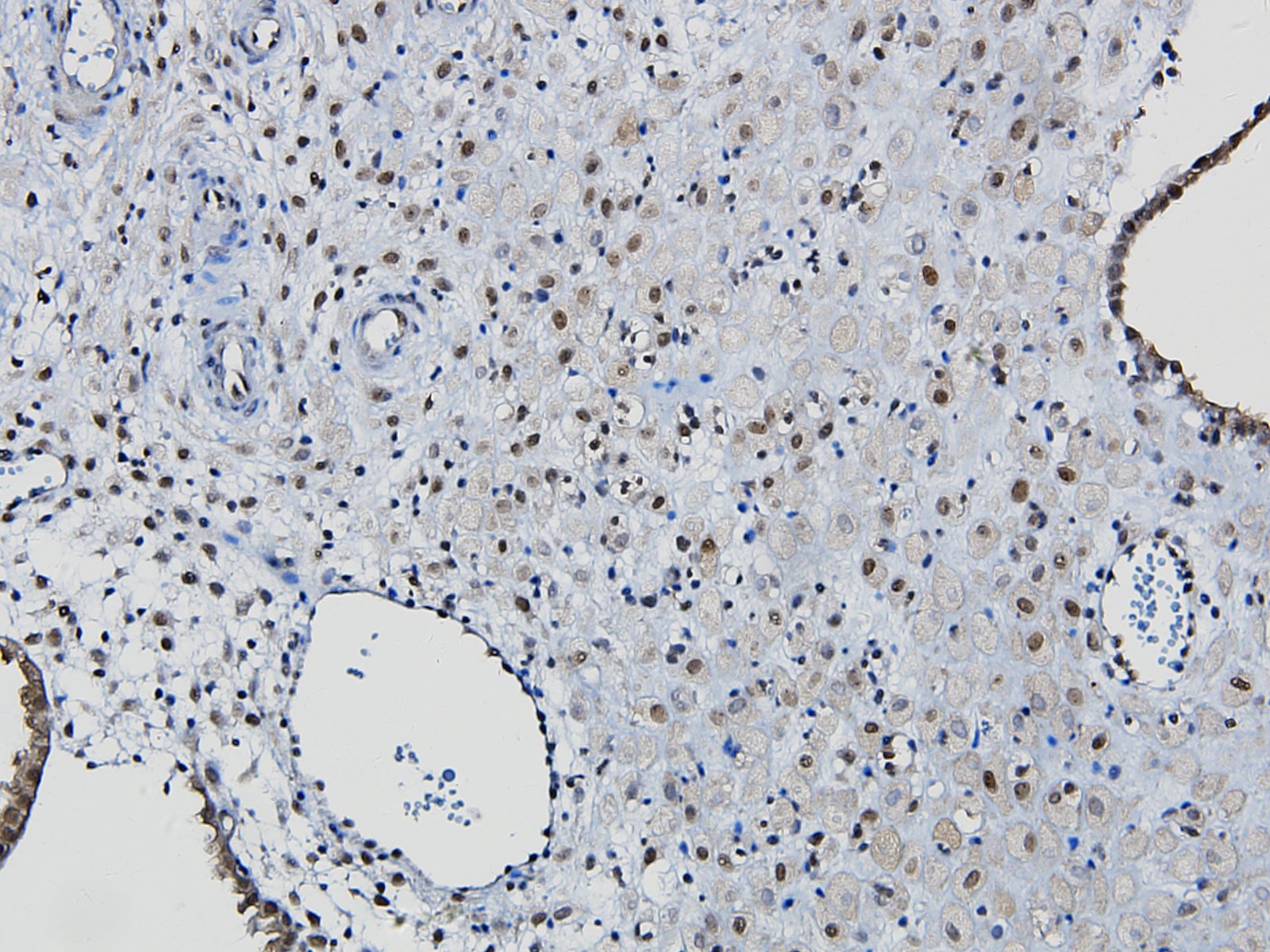

Supplement: Supplementary file 4 — Source data Fig. 2 [file 44318_2024_220_MOESM4_ESM.zip › Figure2/2A/NP-CDKN2A-200-5.jpg]

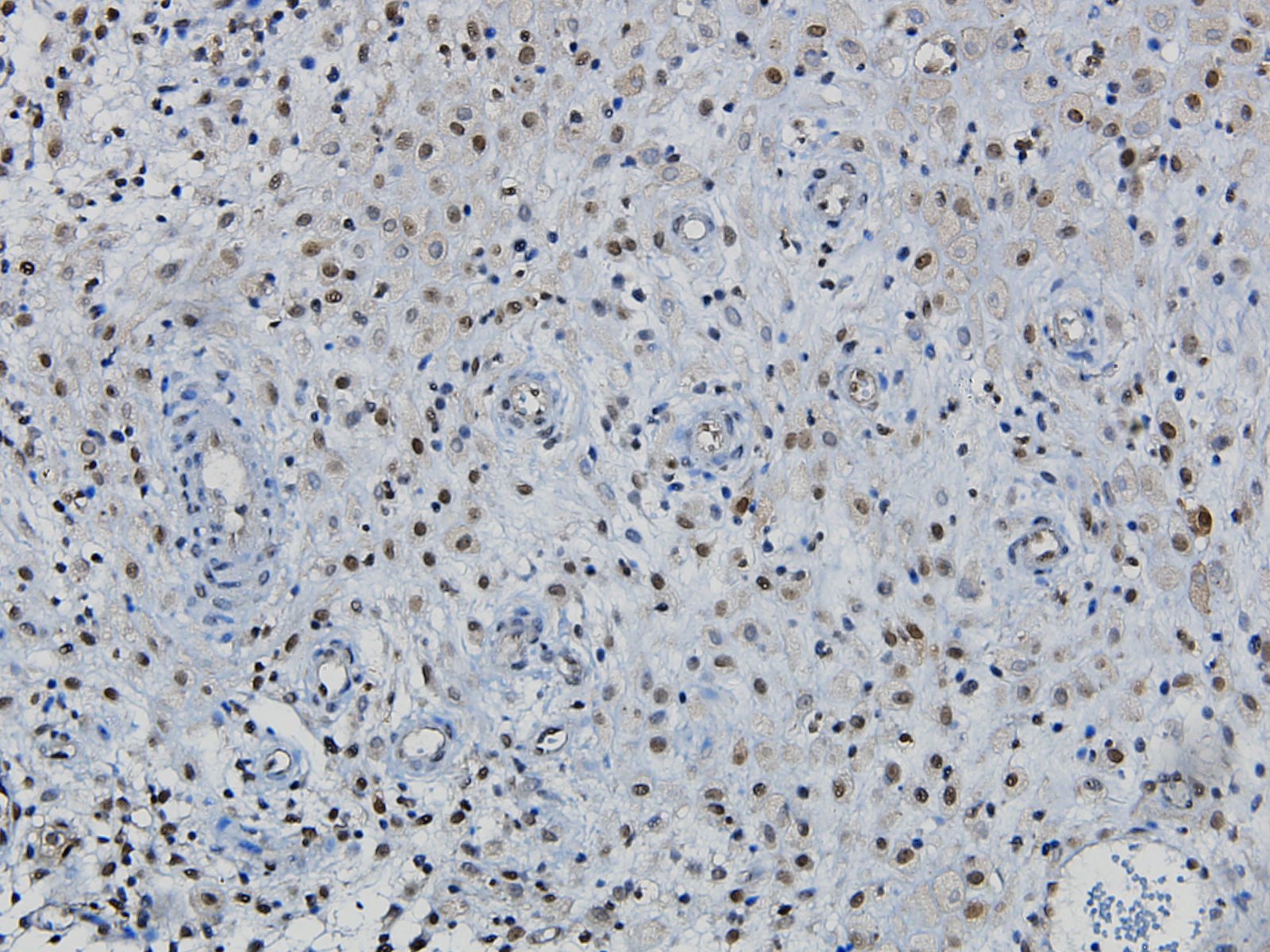

Supplement: Supplementary file 4 — Source data Fig. 2 [file 44318_2024_220_MOESM4_ESM.zip › Figure2/2A/NP-CDKN2A-200-6.jpg]

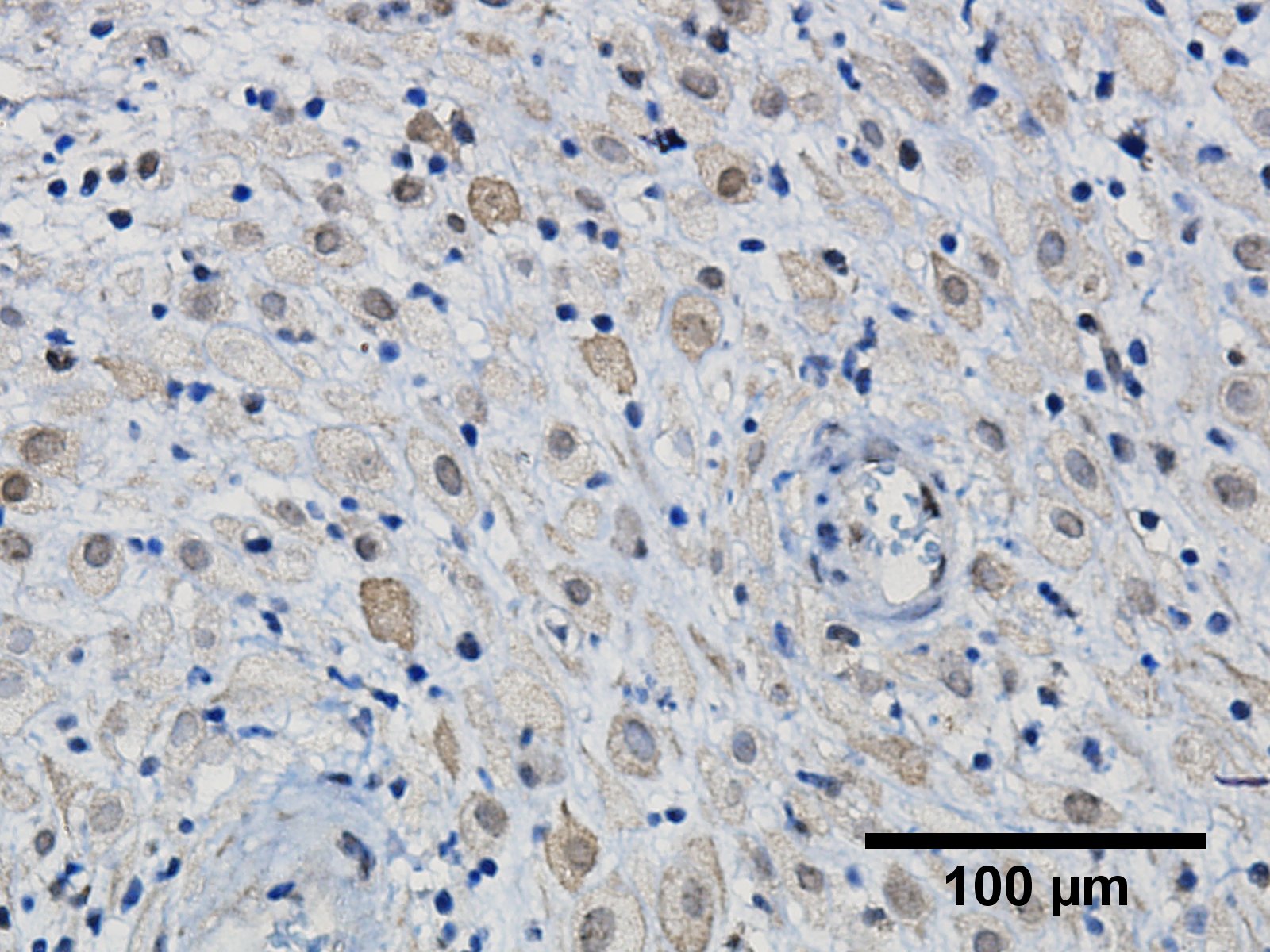

Supplement: Supplementary file 4 — Source data Fig. 2 [file 44318_2024_220_MOESM4_ESM.zip › Figure2/2A/NP-CDKN2A-400-1.jpg]
