# Supplementary figures and images for: TNFSF14+ natural killer cells prevent spontaneous abortion by restricting leucine-mediated decidual stromal cell senescence (part 2 of 4)
Source: EMBO J. 2024 Sep 11;43(21):5018–36. doi: 10.1038/s44318-024-00220-3 (PMC11535022; doi:10.1038/s44318-024-00220-3)

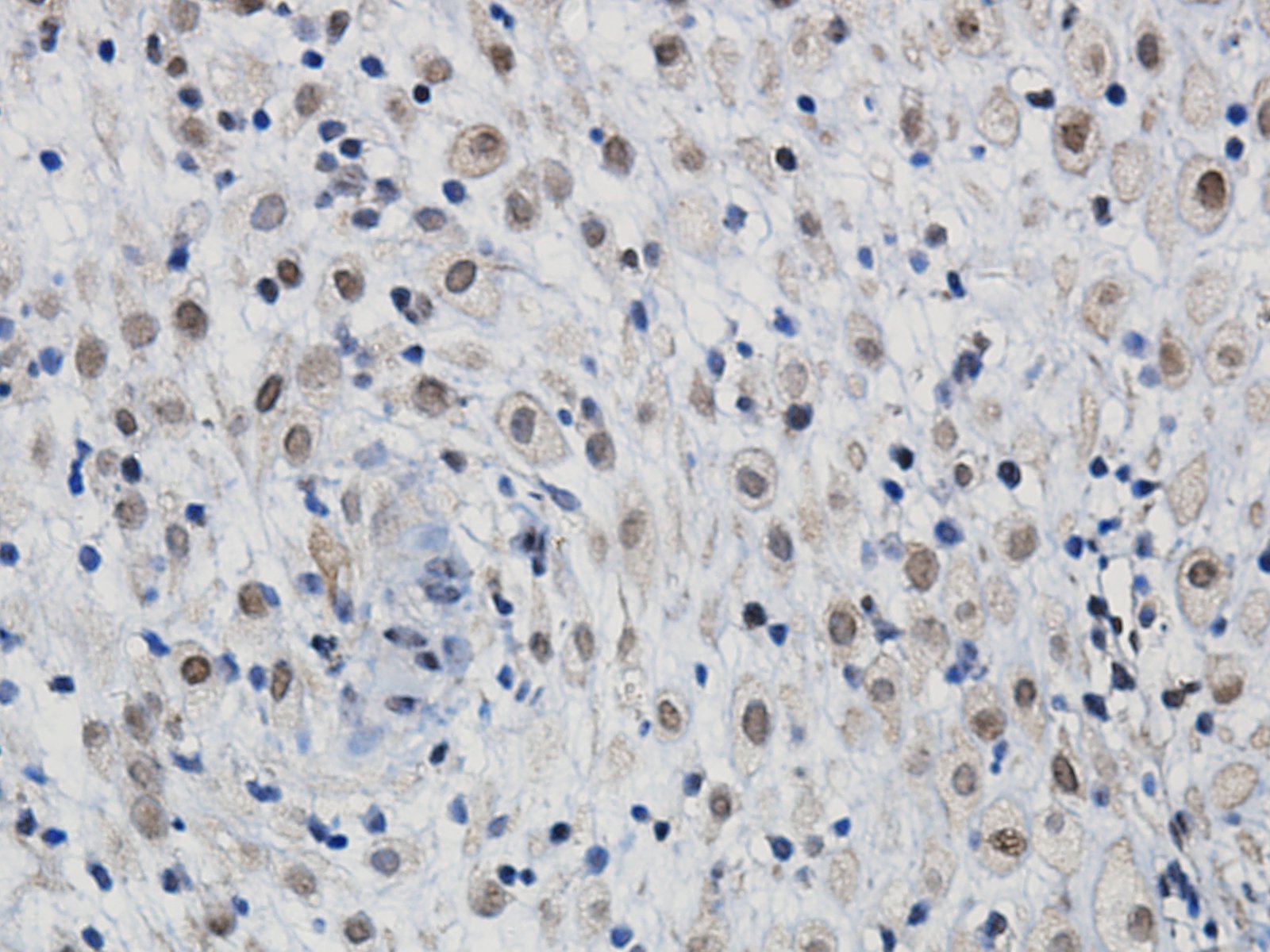

Supplement: Supplementary file 4 — Source data Fig. 2 [file 44318_2024_220_MOESM4_ESM.zip › Figure2/2A/NP-CDKN2A-400-2.jpg]

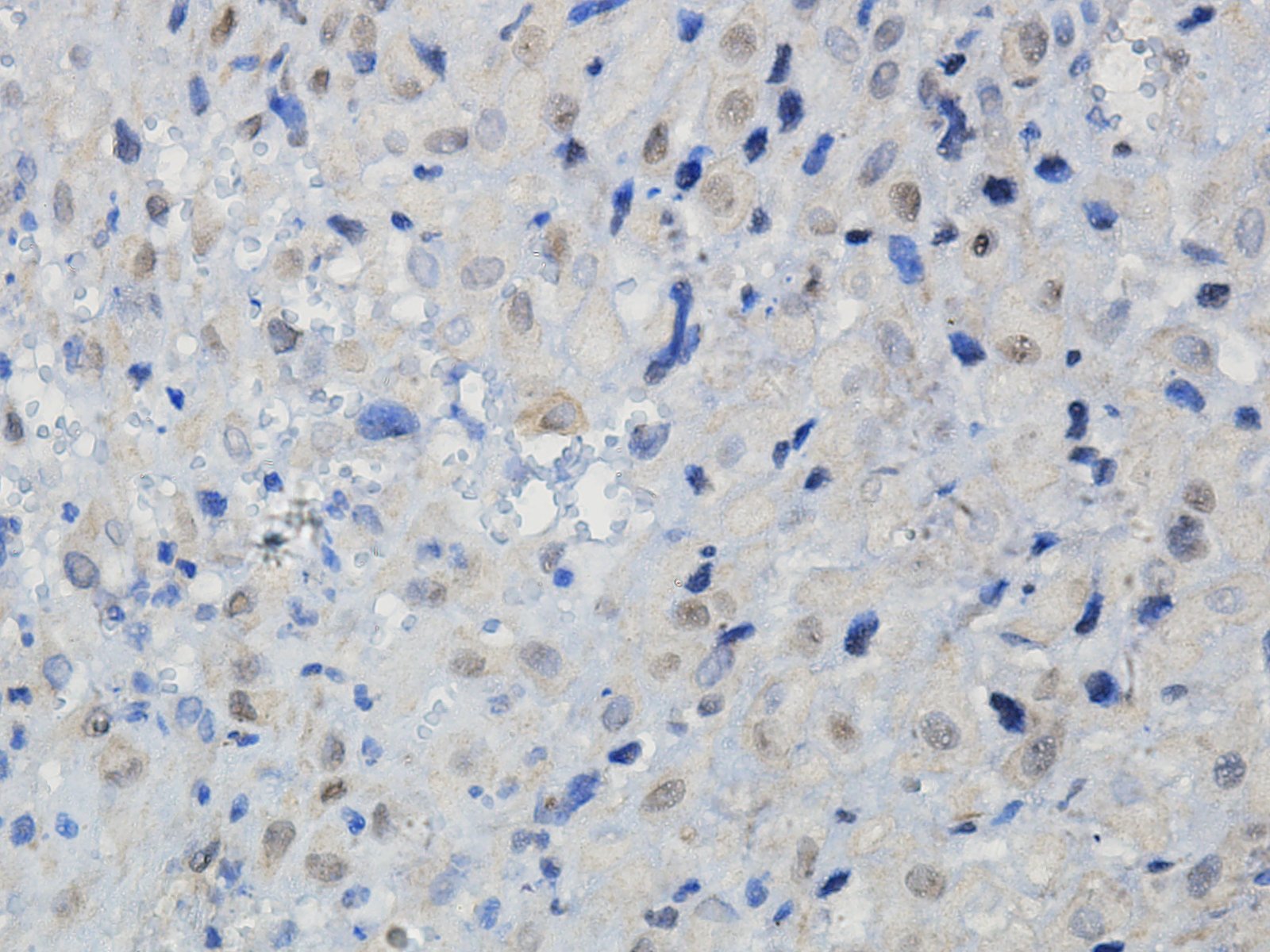

Supplement: Supplementary file 4 — Source data Fig. 2 [file 44318_2024_220_MOESM4_ESM.zip › Figure2/2A/NP-CDKN2A-400-3.jpg]

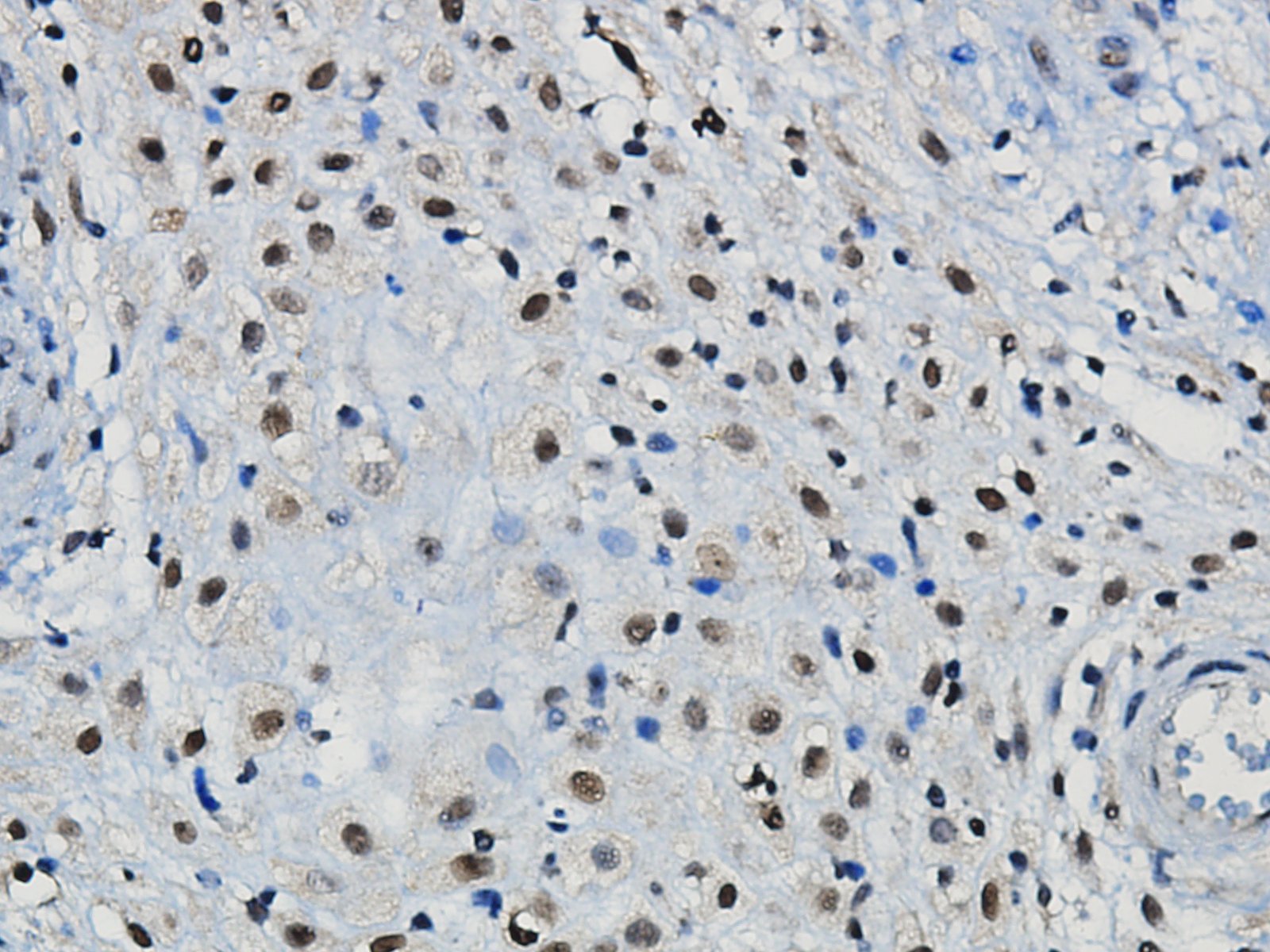

Supplement: Supplementary file 4 — Source data Fig. 2 [file 44318_2024_220_MOESM4_ESM.zip › Figure2/2A/NP-CDKN2A-400-4.jpg]

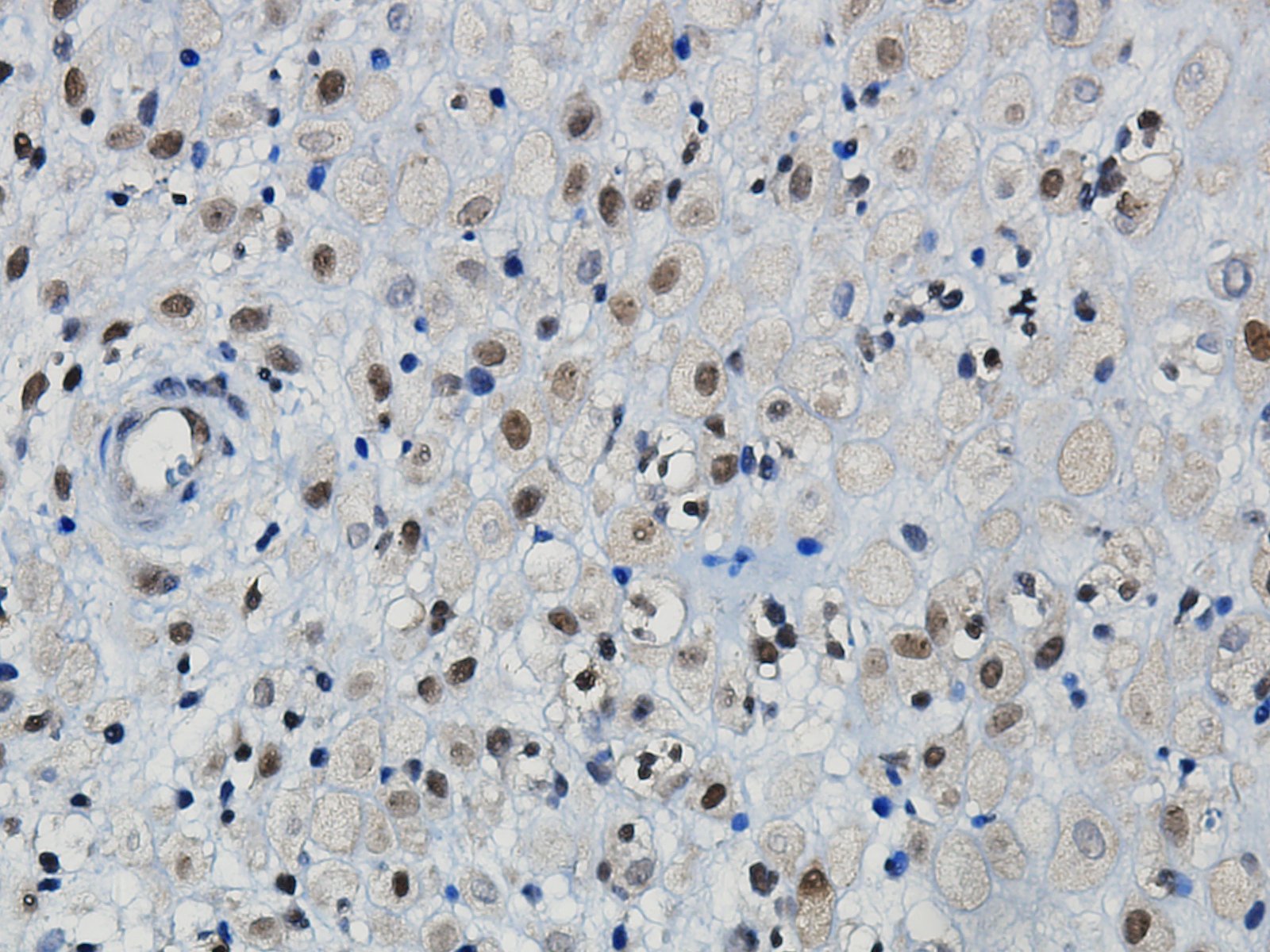

Supplement: Supplementary file 4 — Source data Fig. 2 [file 44318_2024_220_MOESM4_ESM.zip › Figure2/2A/NP-CDKN2A-400-5.jpg]

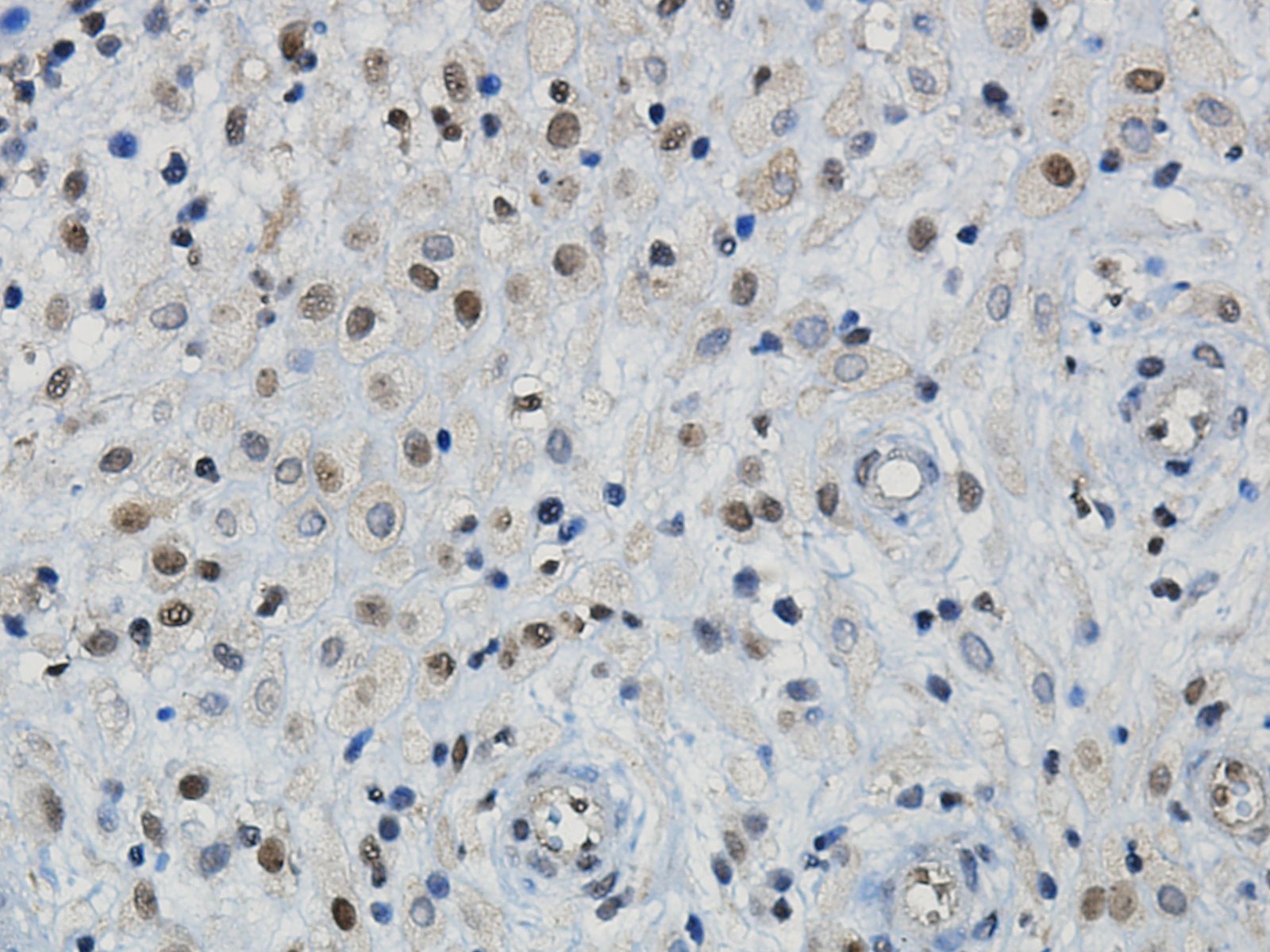

Supplement: Supplementary file 4 — Source data Fig. 2 [file 44318_2024_220_MOESM4_ESM.zip › Figure2/2A/NP-CDKN2A-400-6.jpg]

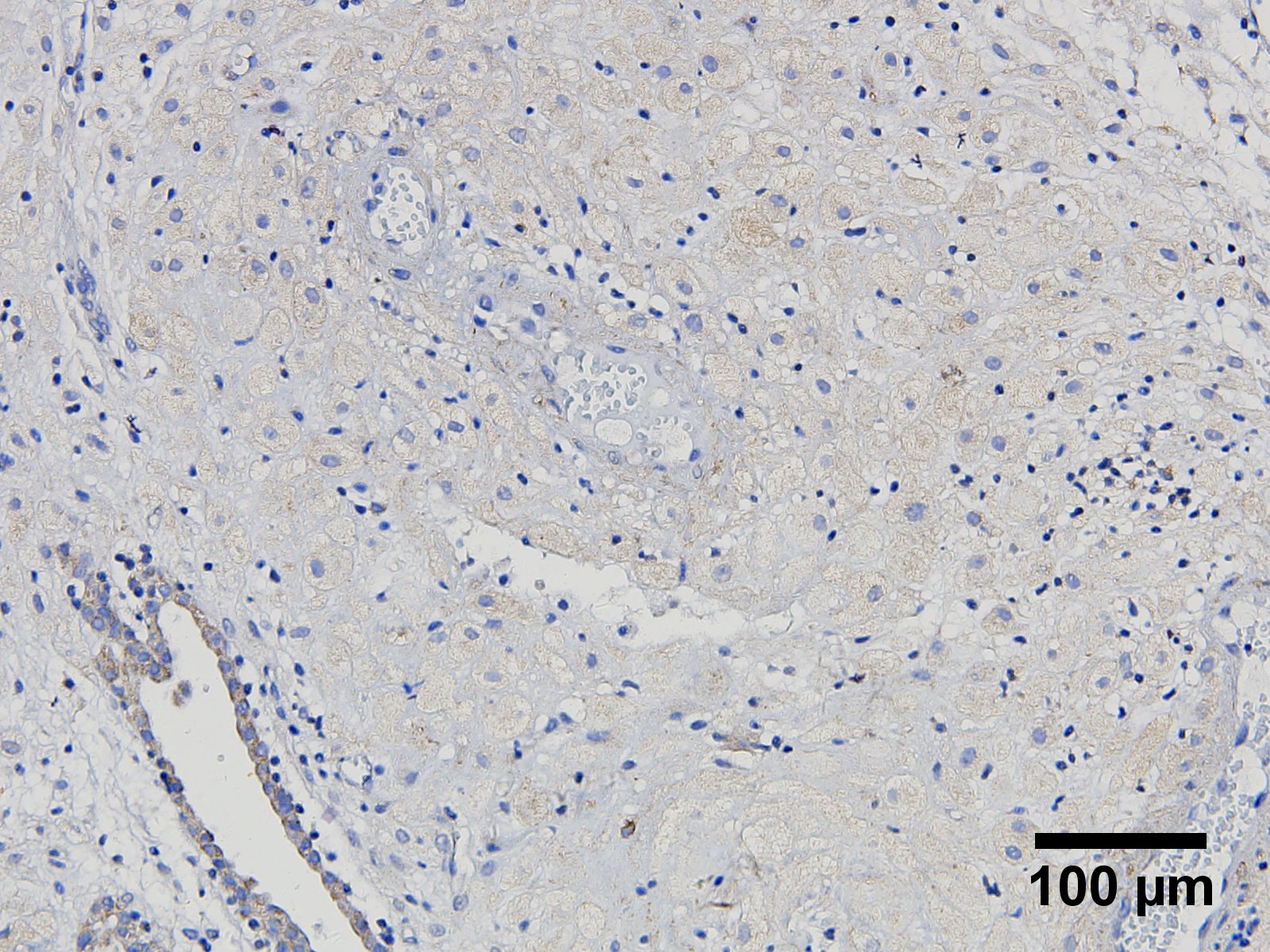

Supplement: Supplementary file 4 — Source data Fig. 2 [file 44318_2024_220_MOESM4_ESM.zip › Figure2/2A/NP-TP53-200-1.jpg]

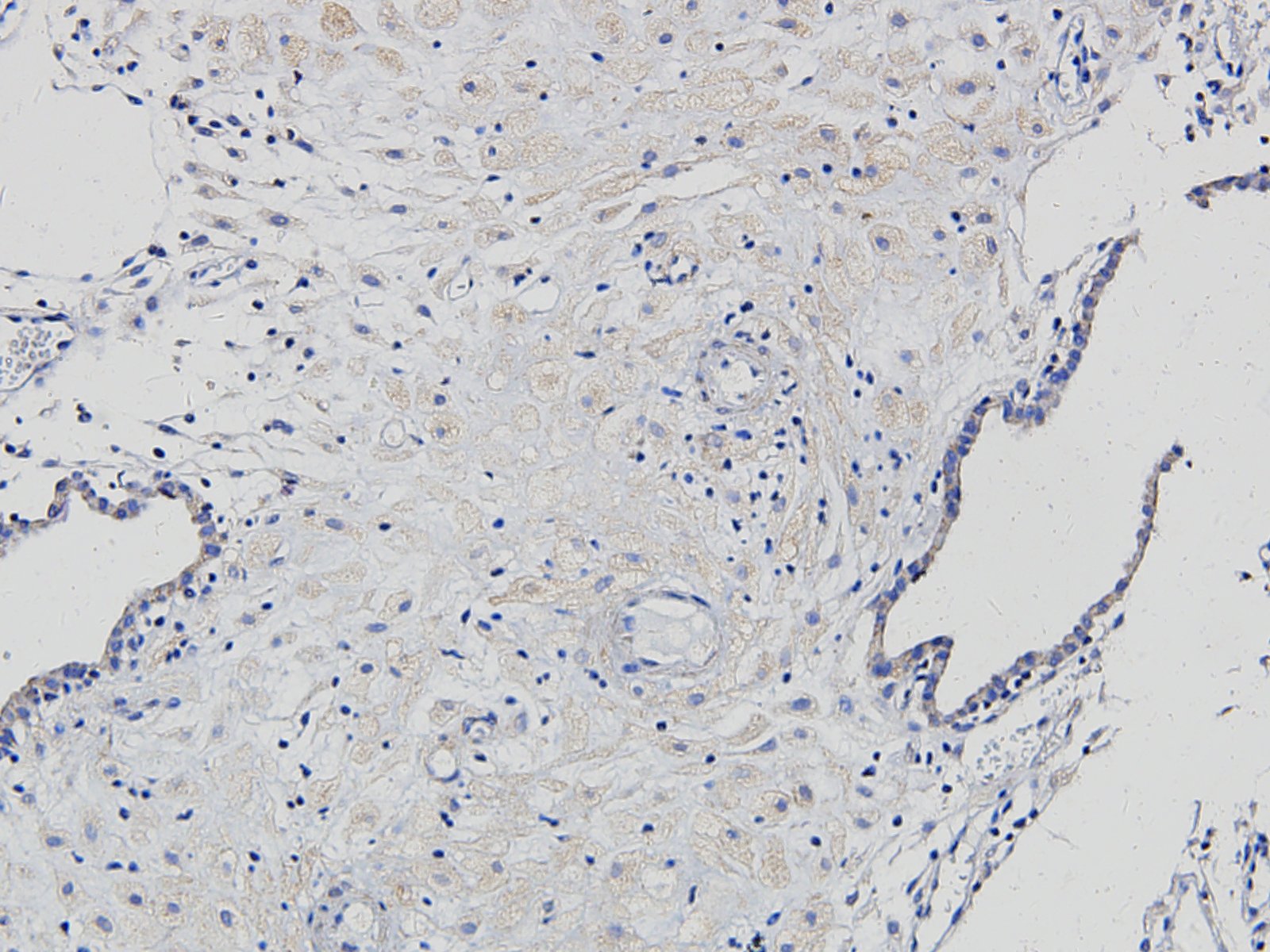

Supplement: Supplementary file 4 — Source data Fig. 2 [file 44318_2024_220_MOESM4_ESM.zip › Figure2/2A/NP-TP53-200-2.jpg]

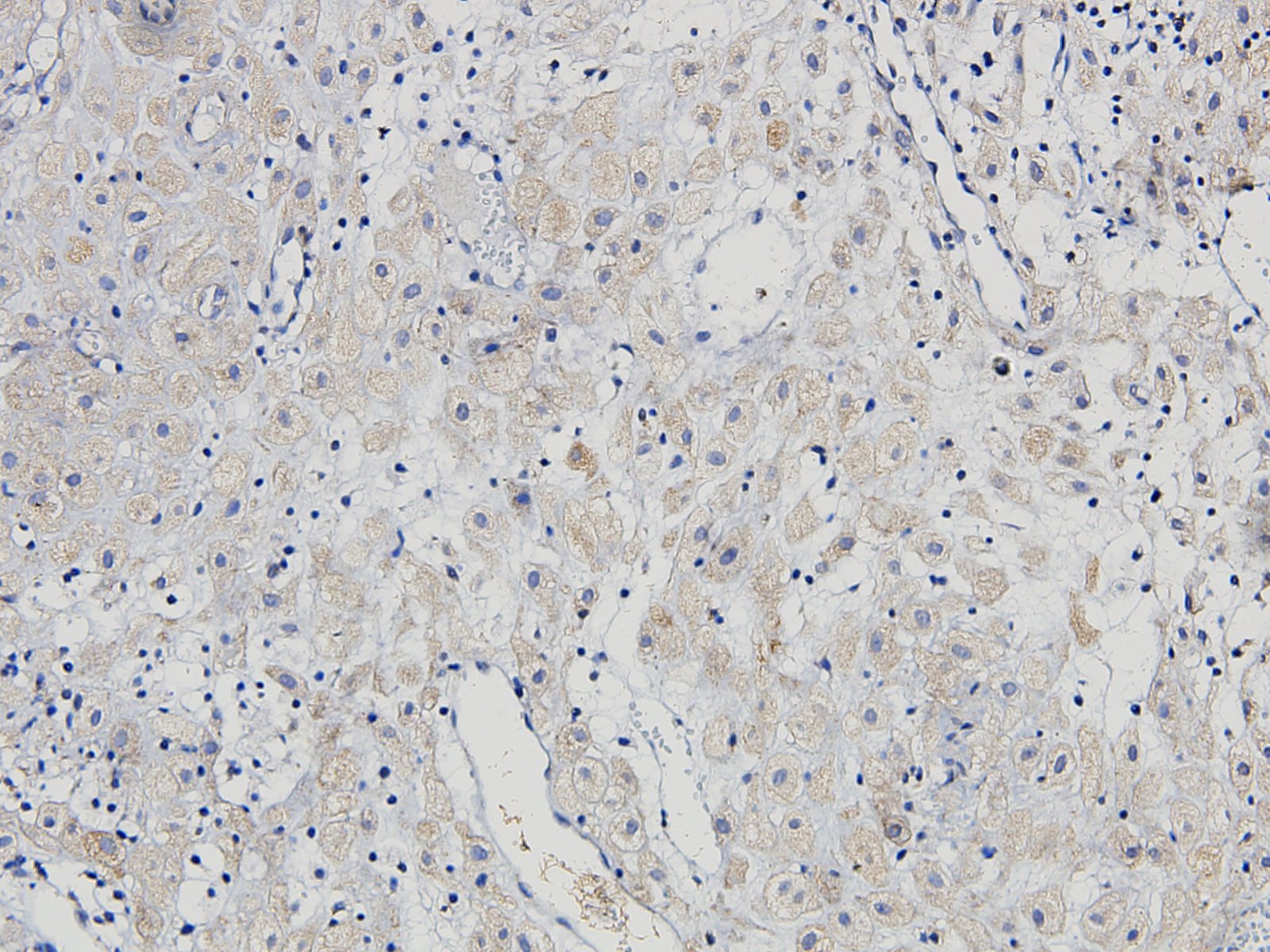

Supplement: Supplementary file 4 — Source data Fig. 2 [file 44318_2024_220_MOESM4_ESM.zip › Figure2/2A/NP-TP53-200-3.jpg]

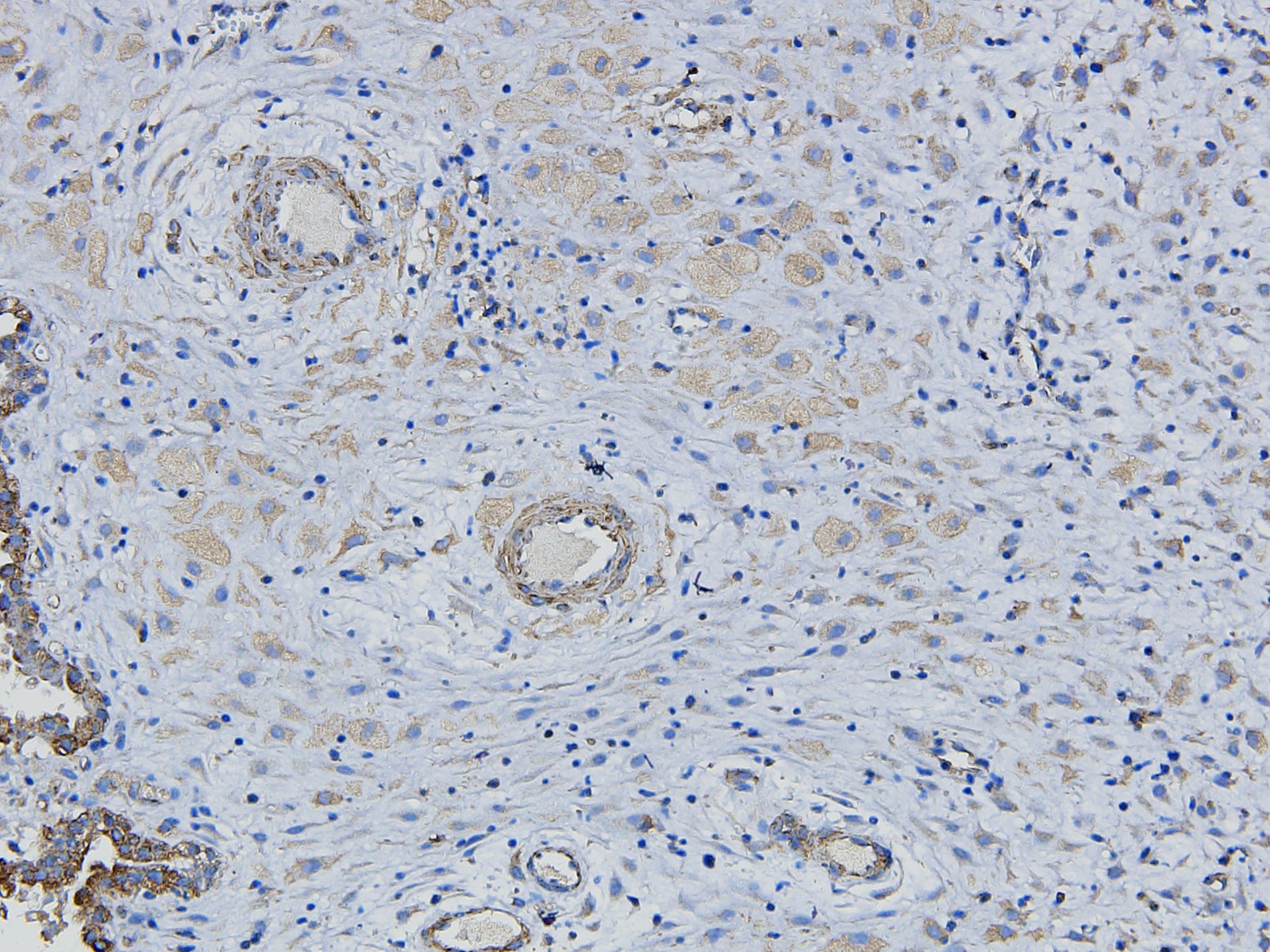

Supplement: Supplementary file 4 — Source data Fig. 2 [file 44318_2024_220_MOESM4_ESM.zip › Figure2/2A/NP-TP53-200-4.jpg]

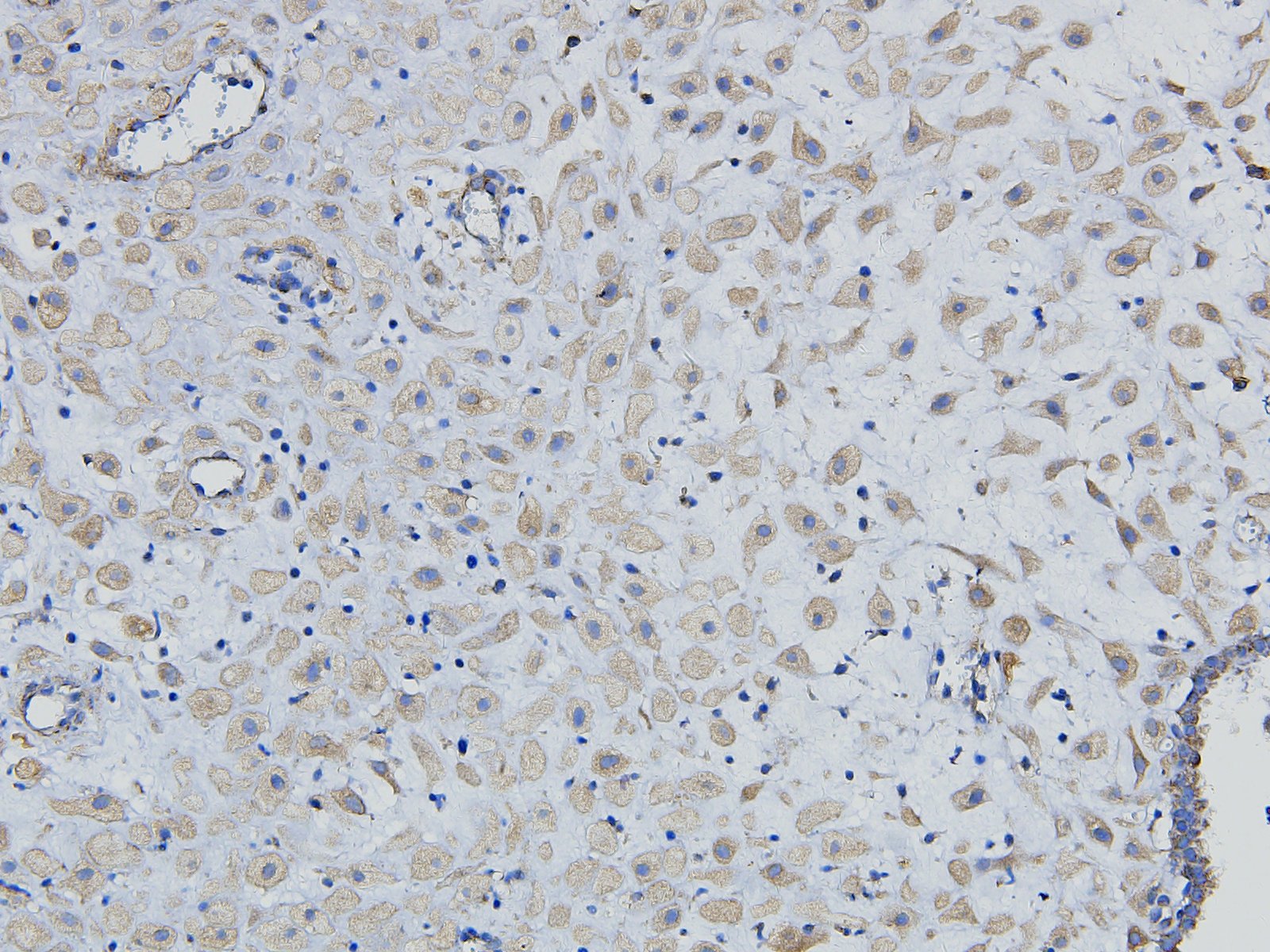

Supplement: Supplementary file 4 — Source data Fig. 2 [file 44318_2024_220_MOESM4_ESM.zip › Figure2/2A/NP-TP53-200-5.jpg]

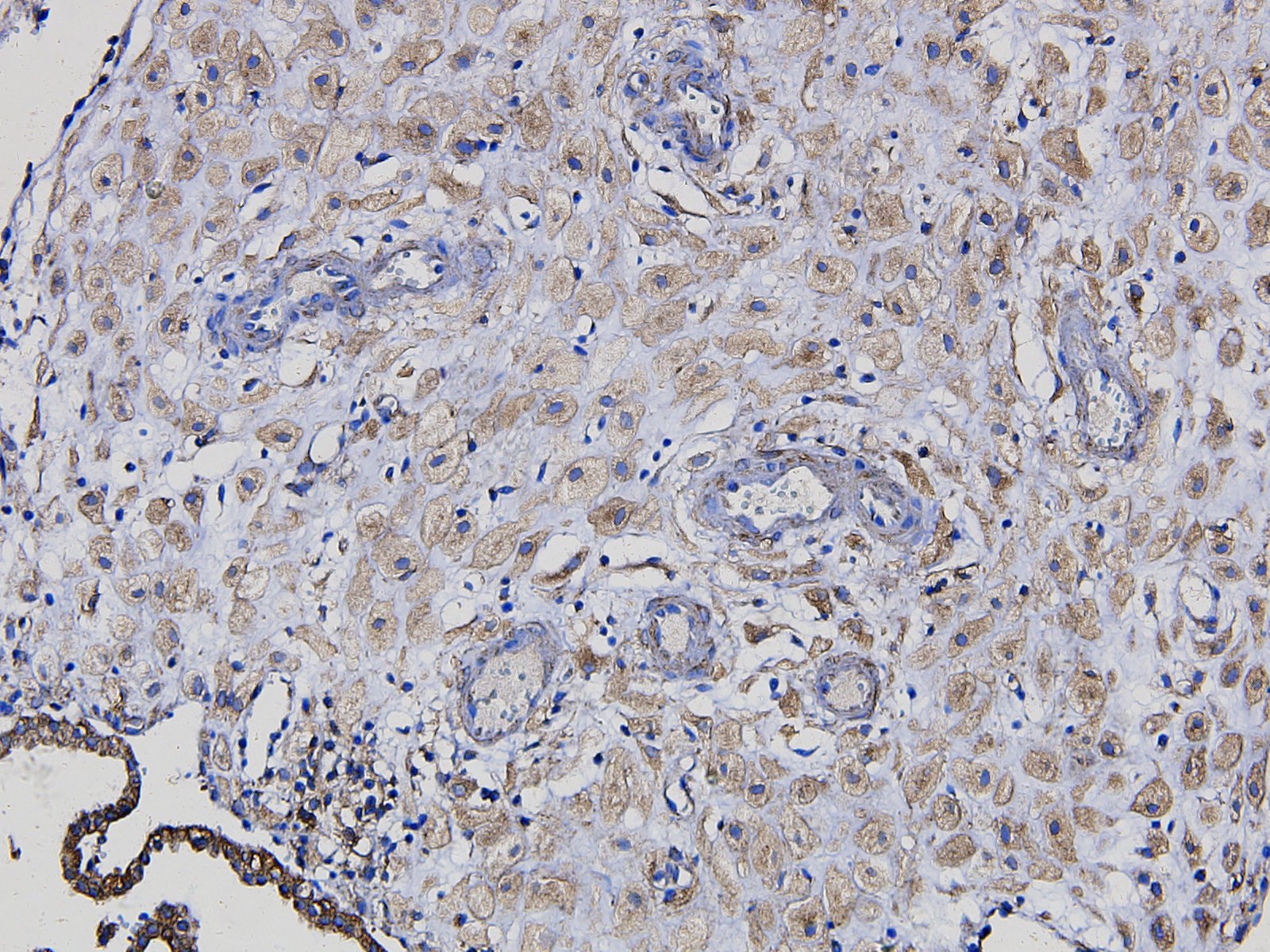

Supplement: Supplementary file 4 — Source data Fig. 2 [file 44318_2024_220_MOESM4_ESM.zip › Figure2/2A/NP-TP53-200-6.jpg]

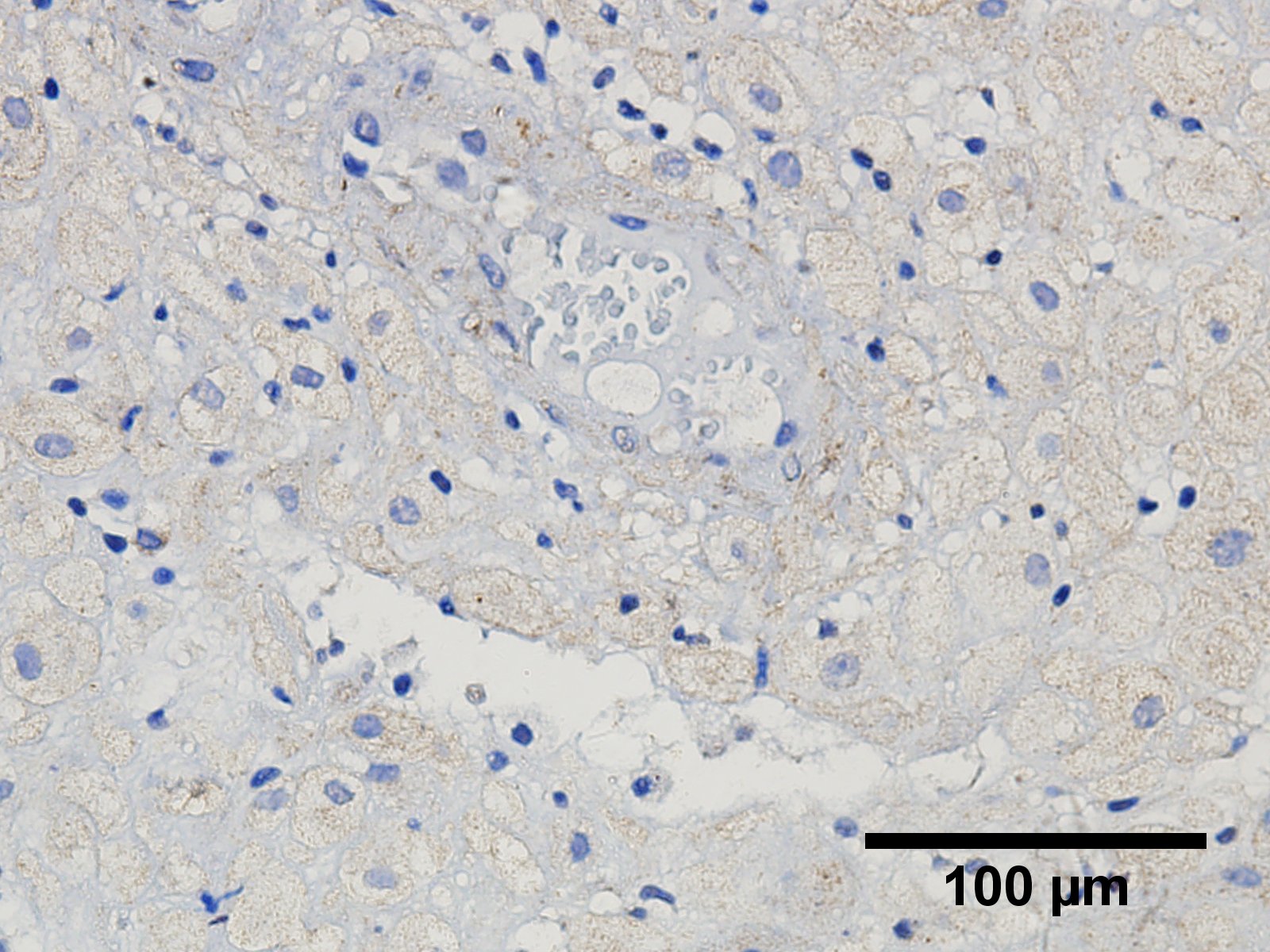

Supplement: Supplementary file 4 — Source data Fig. 2 [file 44318_2024_220_MOESM4_ESM.zip › Figure2/2A/NP-TP53-400-1.jpg]

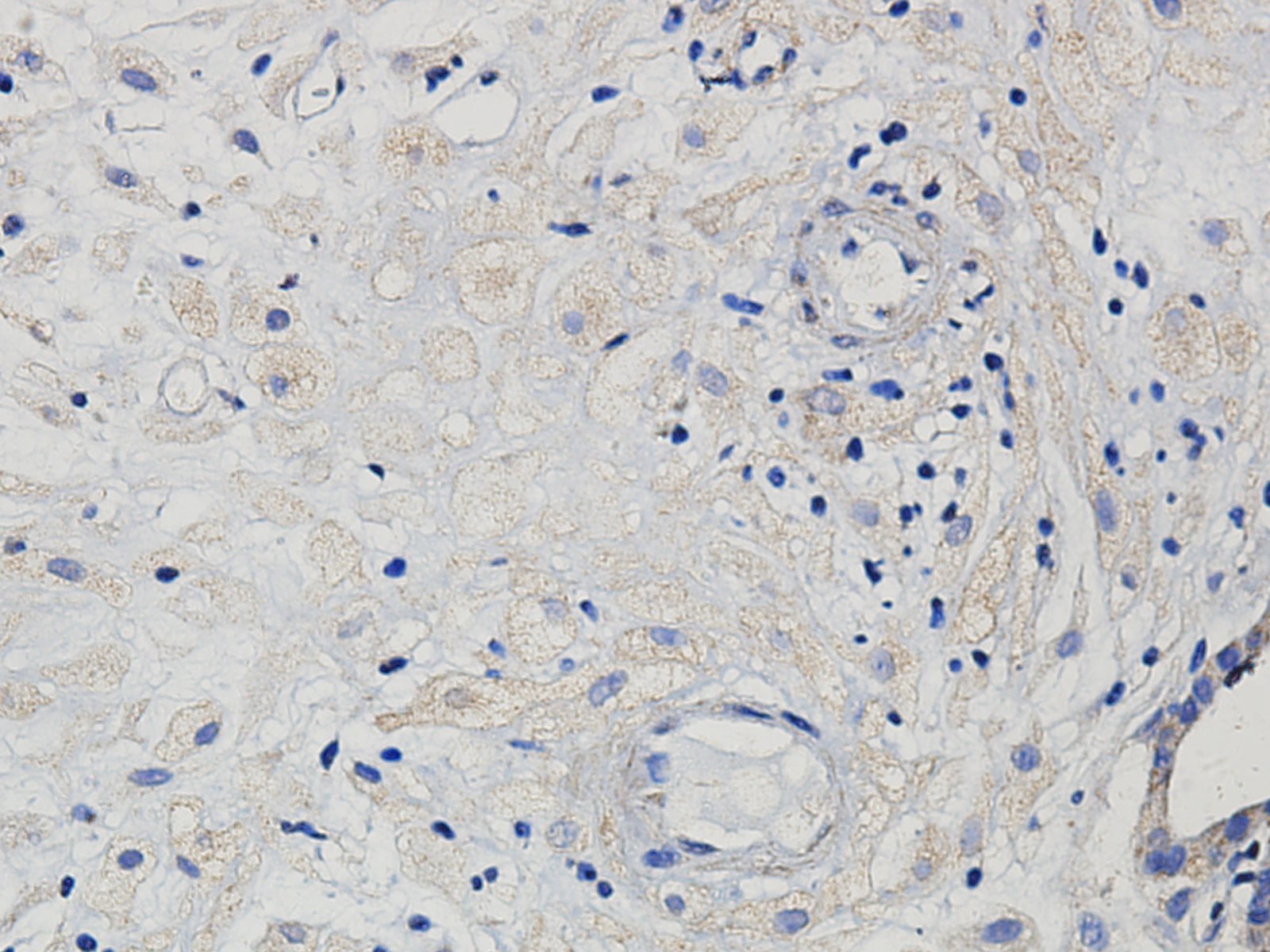

Supplement: Supplementary file 4 — Source data Fig. 2 [file 44318_2024_220_MOESM4_ESM.zip › Figure2/2A/NP-TP53-400-2.jpg]

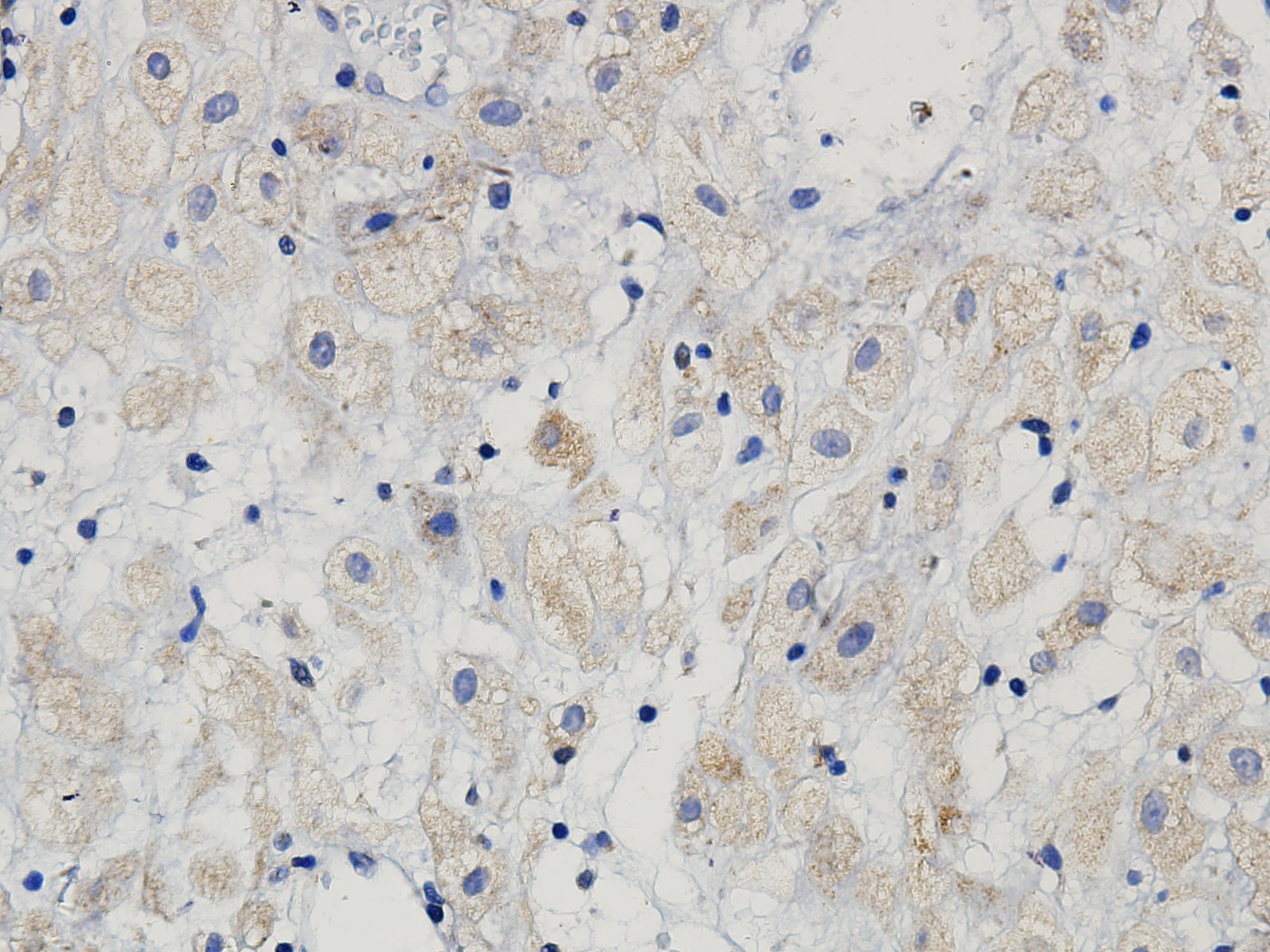

Supplement: Supplementary file 4 — Source data Fig. 2 [file 44318_2024_220_MOESM4_ESM.zip › Figure2/2A/NP-TP53-400-3.jpg]

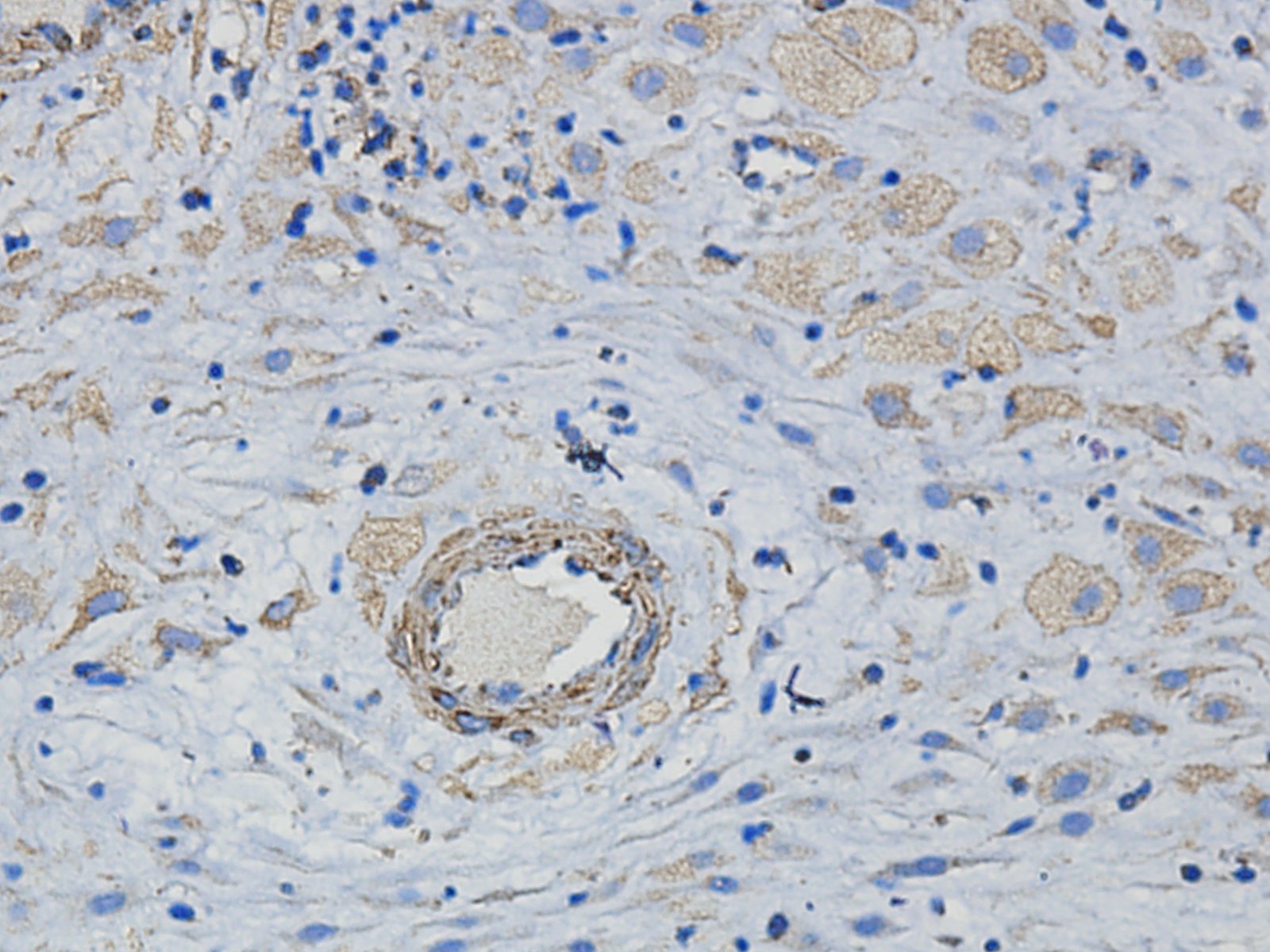

Supplement: Supplementary file 4 — Source data Fig. 2 [file 44318_2024_220_MOESM4_ESM.zip › Figure2/2A/NP-TP53-400-4.jpg]

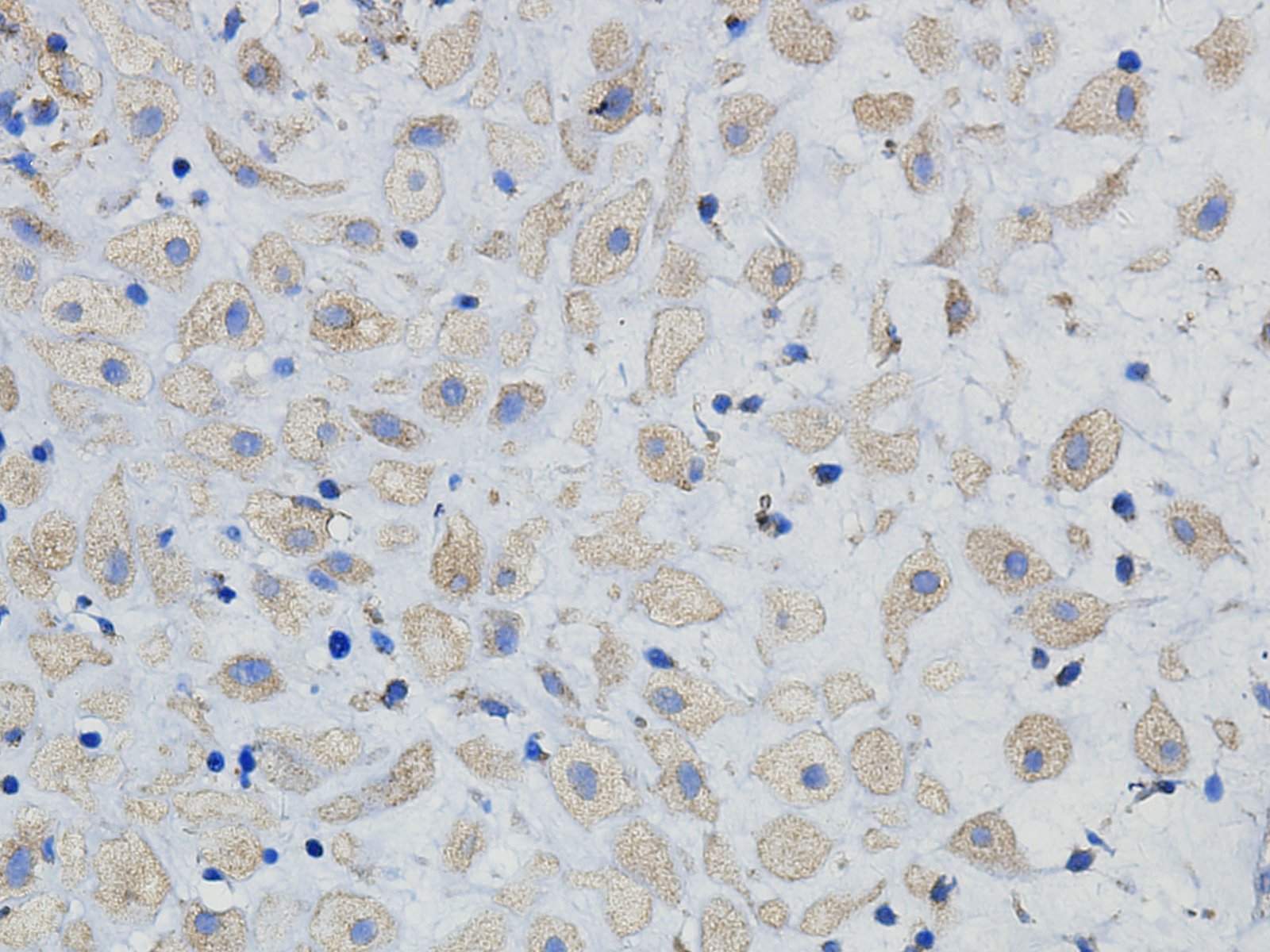

Supplement: Supplementary file 4 — Source data Fig. 2 [file 44318_2024_220_MOESM4_ESM.zip › Figure2/2A/NP-TP53-400-5.jpg]

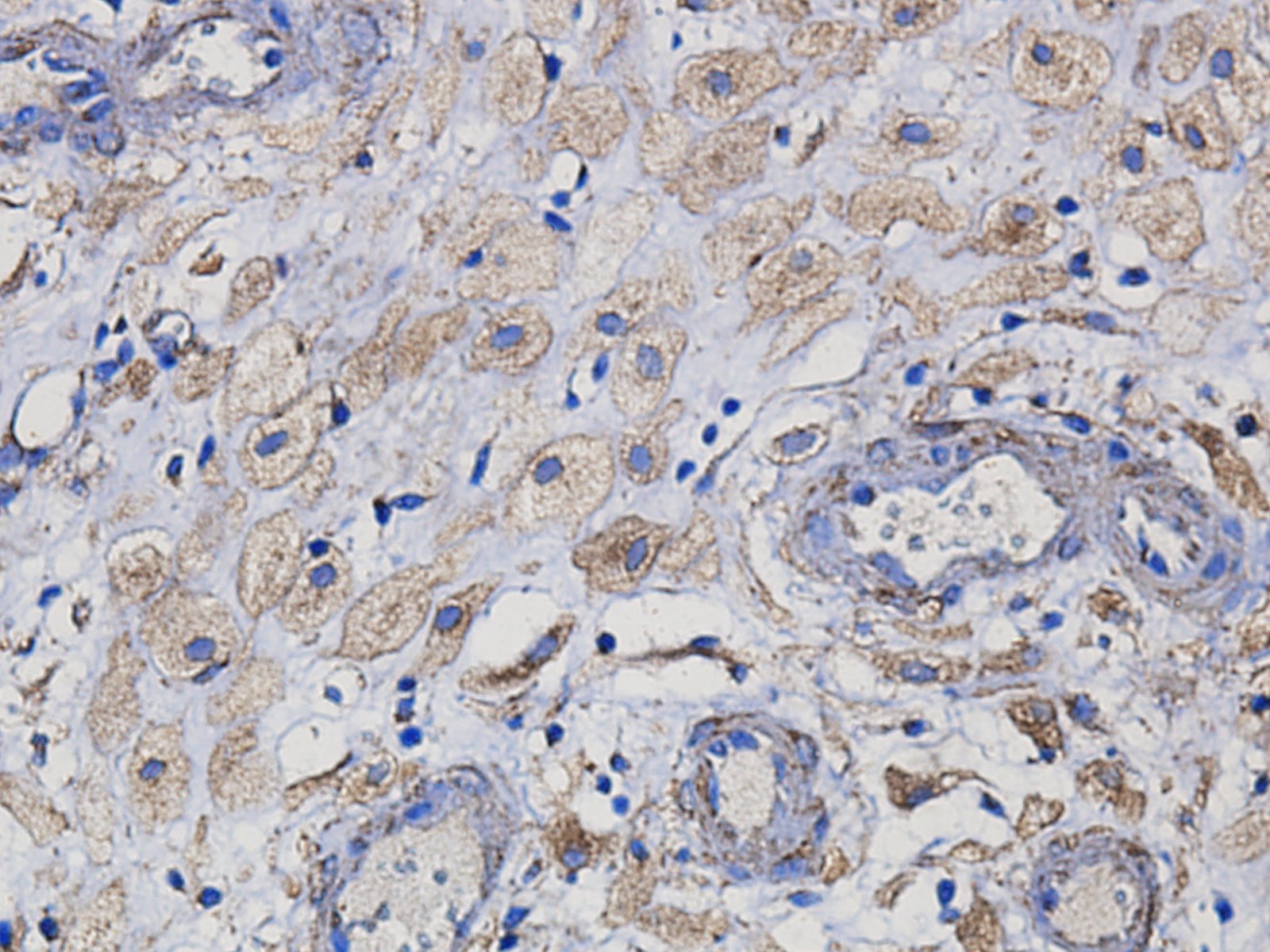

Supplement: Supplementary file 4 — Source data Fig. 2 [file 44318_2024_220_MOESM4_ESM.zip › Figure2/2A/NP-TP53-400-6.jpg]

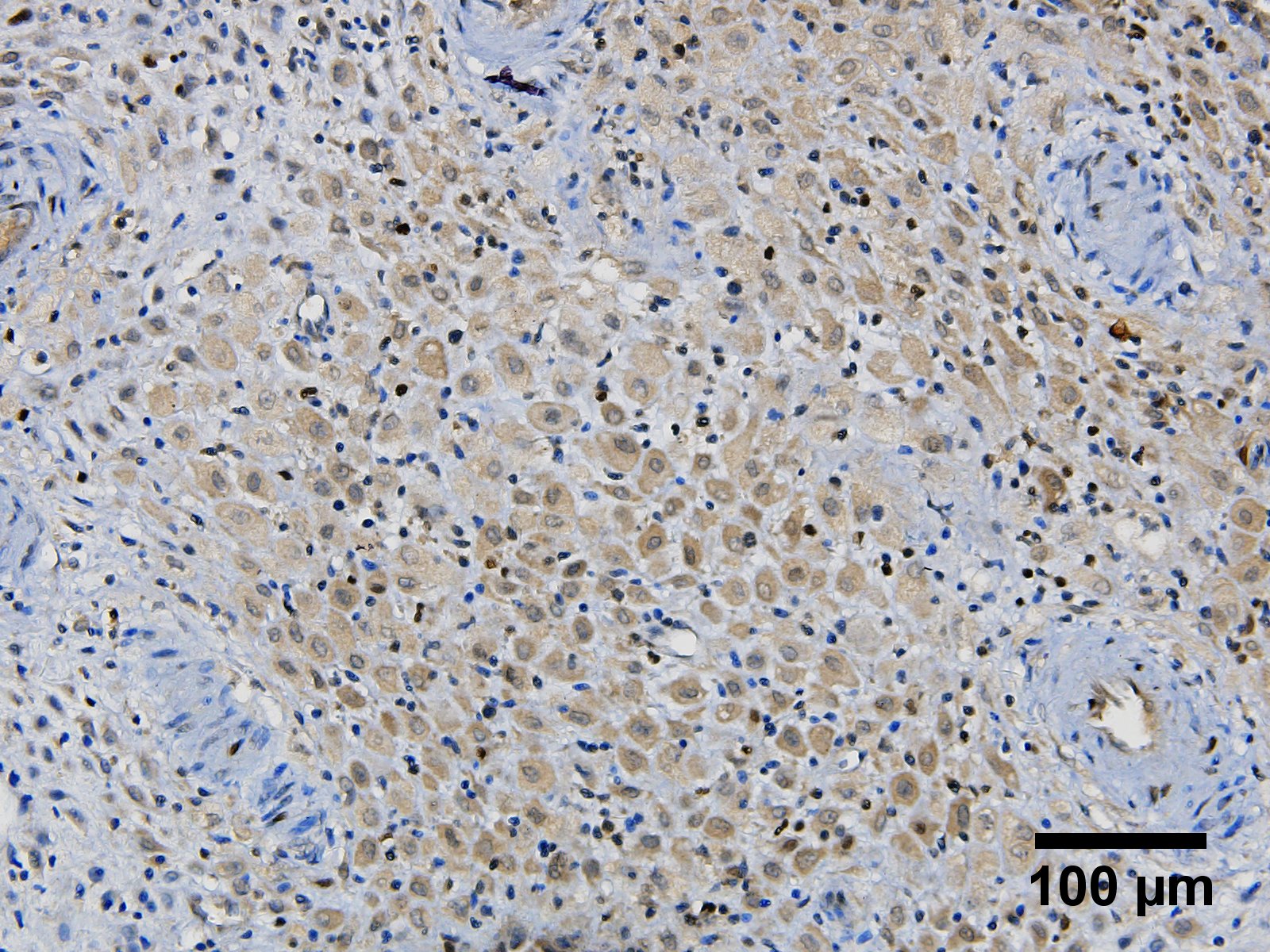

Supplement: Supplementary file 4 — Source data Fig. 2 [file 44318_2024_220_MOESM4_ESM.zip › Figure2/2A/RSA-CDKN1A-200-1.jpg]

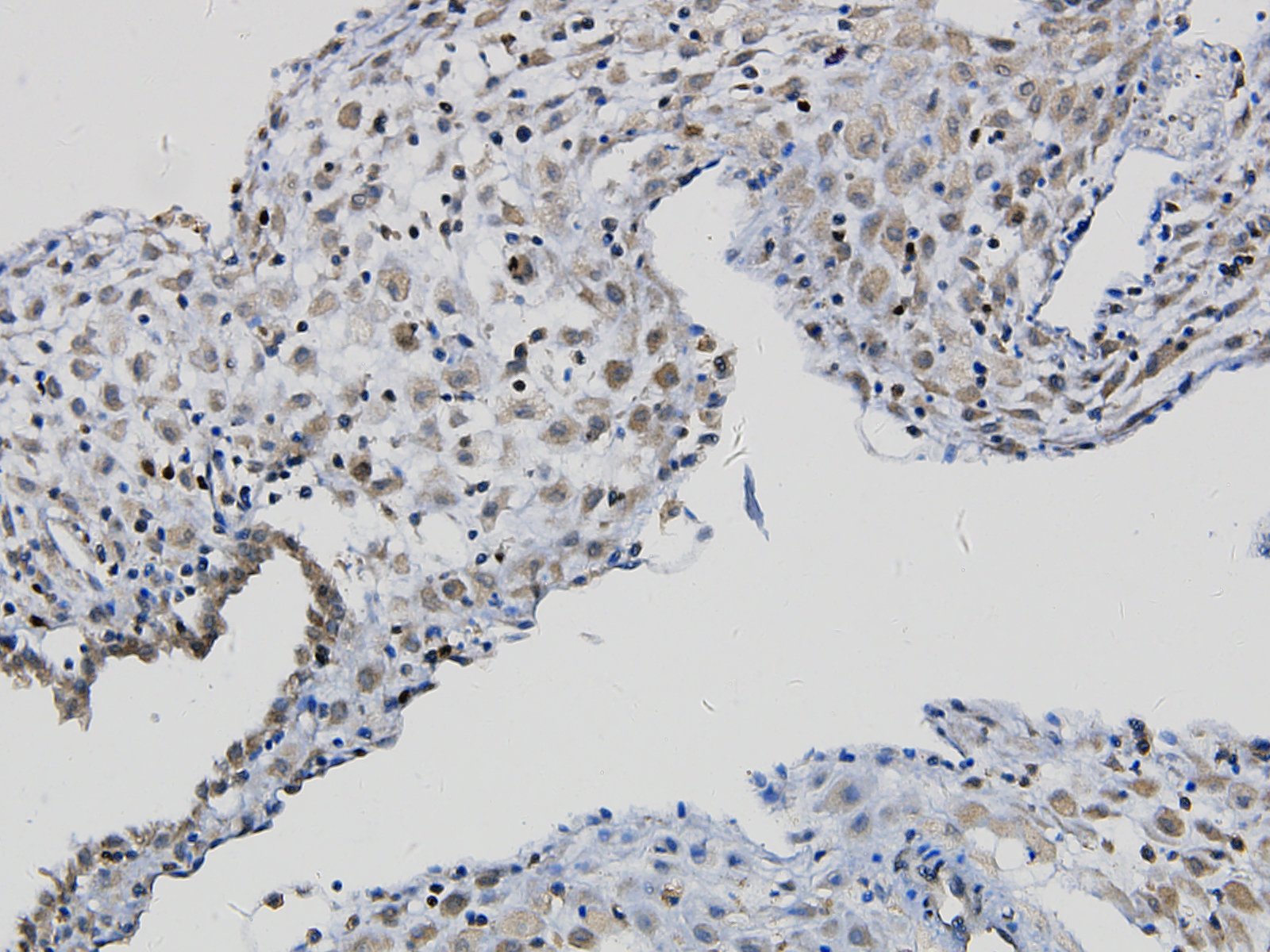

Supplement: Supplementary file 4 — Source data Fig. 2 [file 44318_2024_220_MOESM4_ESM.zip › Figure2/2A/RSA-CDKN1A-200-2.jpg]

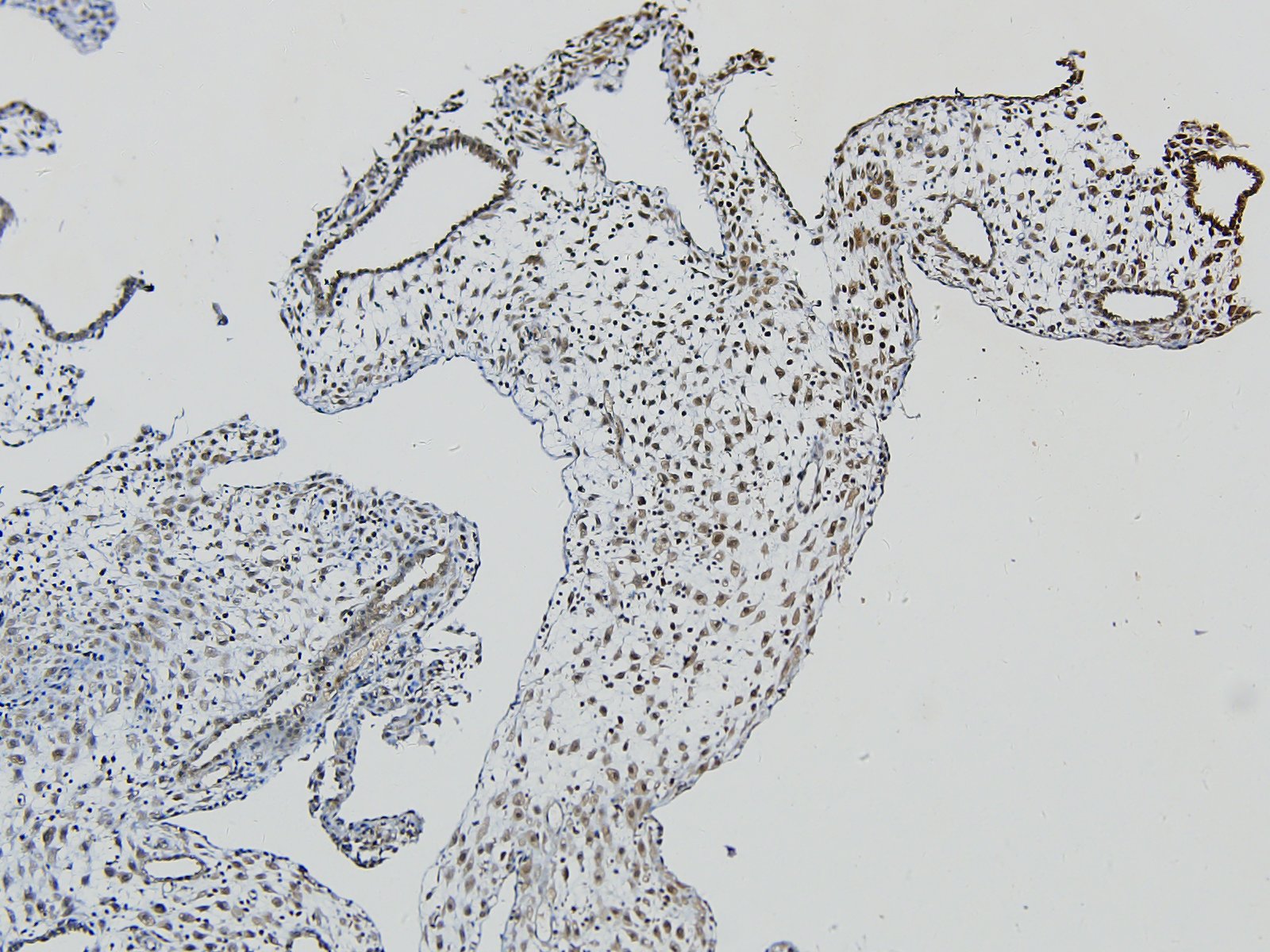

Supplement: Supplementary file 4 — Source data Fig. 2 [file 44318_2024_220_MOESM4_ESM.zip › Figure2/2A/RSA-CDKN1A-200-3.jpg]

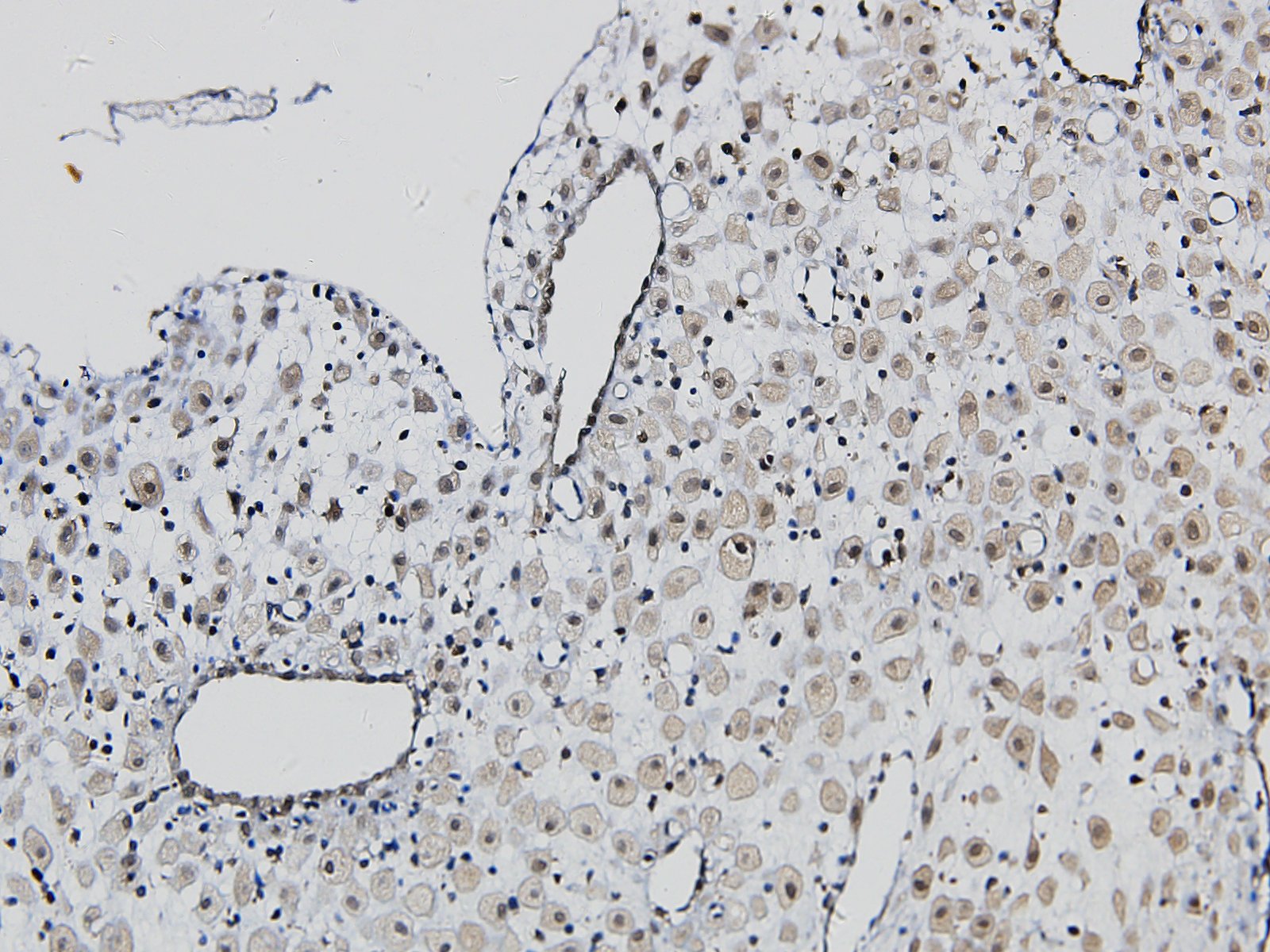

Supplement: Supplementary file 4 — Source data Fig. 2 [file 44318_2024_220_MOESM4_ESM.zip › Figure2/2A/RSA-CDKN1A-200-4.jpg]

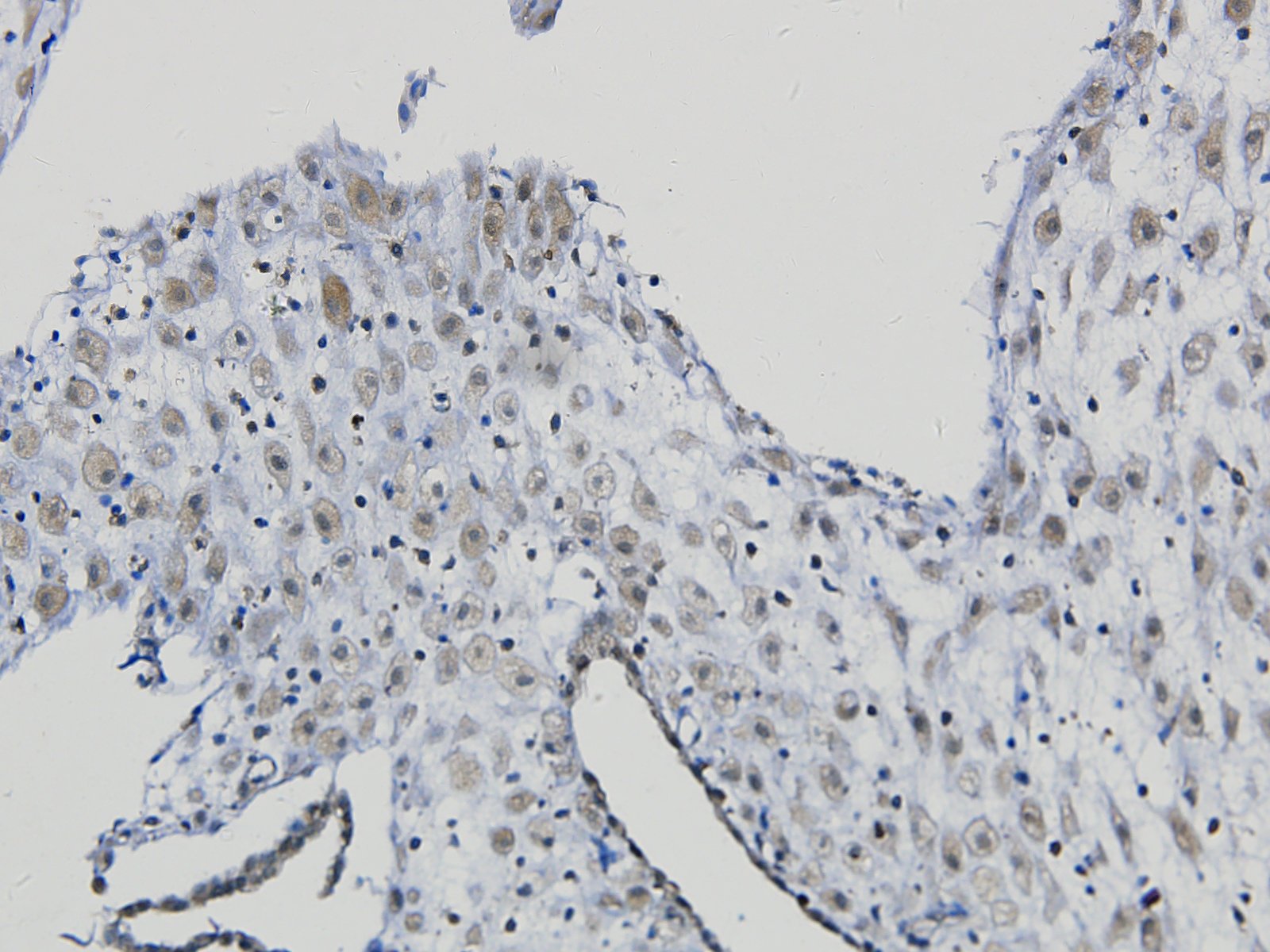

Supplement: Supplementary file 4 — Source data Fig. 2 [file 44318_2024_220_MOESM4_ESM.zip › Figure2/2A/RSA-CDKN1A-200-5.jpg]

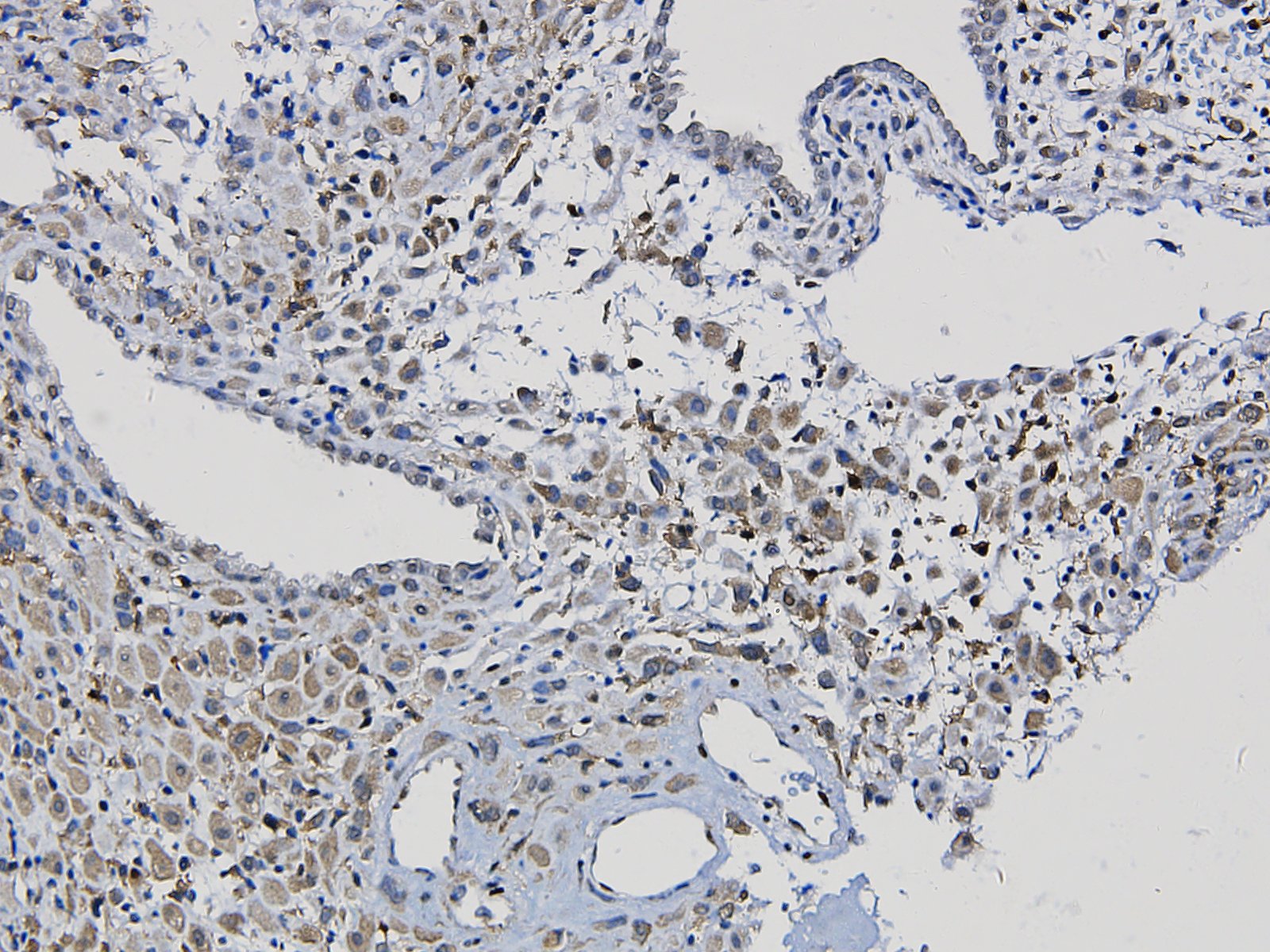

Supplement: Supplementary file 4 — Source data Fig. 2 [file 44318_2024_220_MOESM4_ESM.zip › Figure2/2A/RSA-CDKN1A-200-6.jpg]

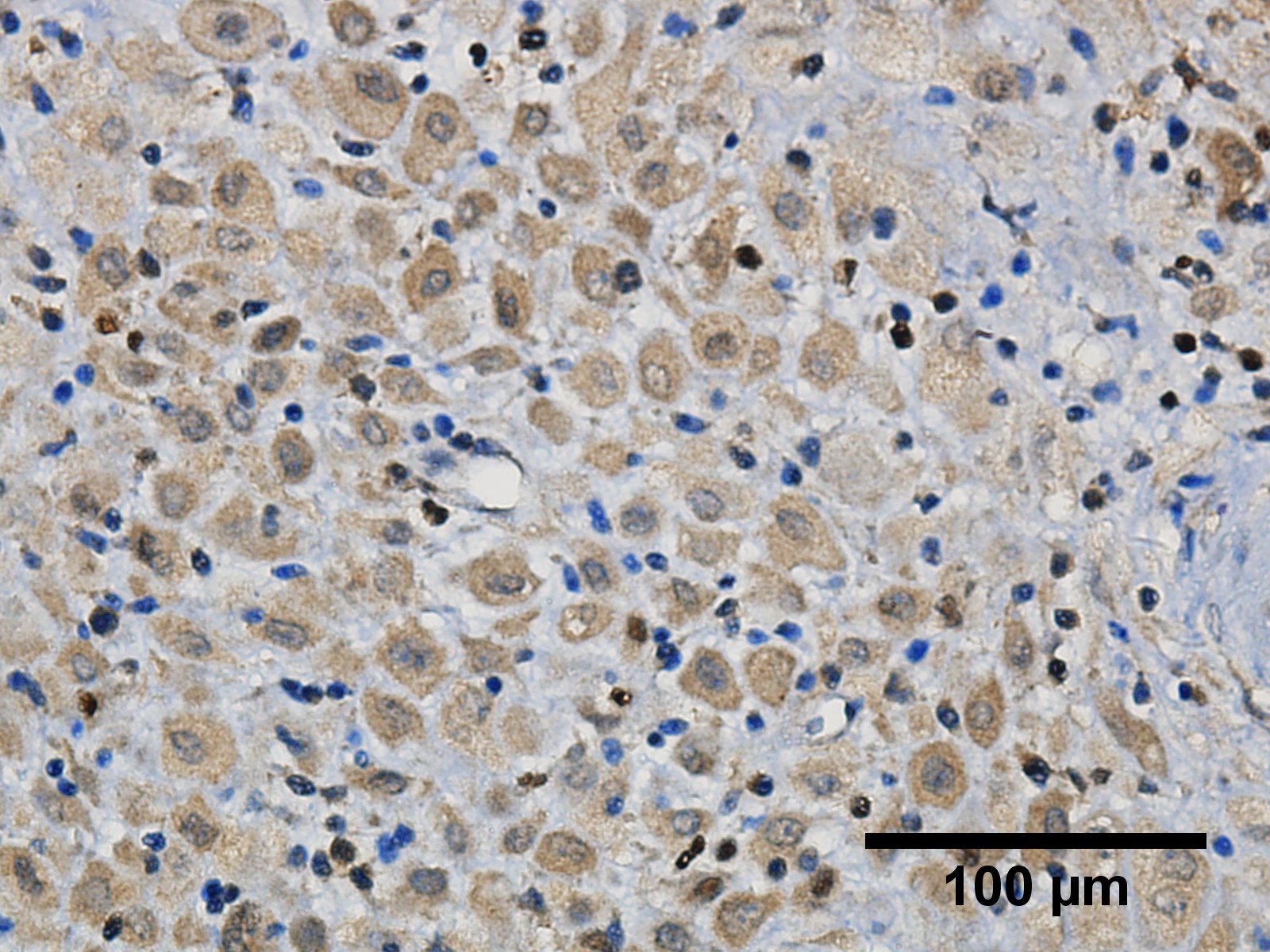

Supplement: Supplementary file 4 — Source data Fig. 2 [file 44318_2024_220_MOESM4_ESM.zip › Figure2/2A/RSA-CDKN1A-400-1.jpg]

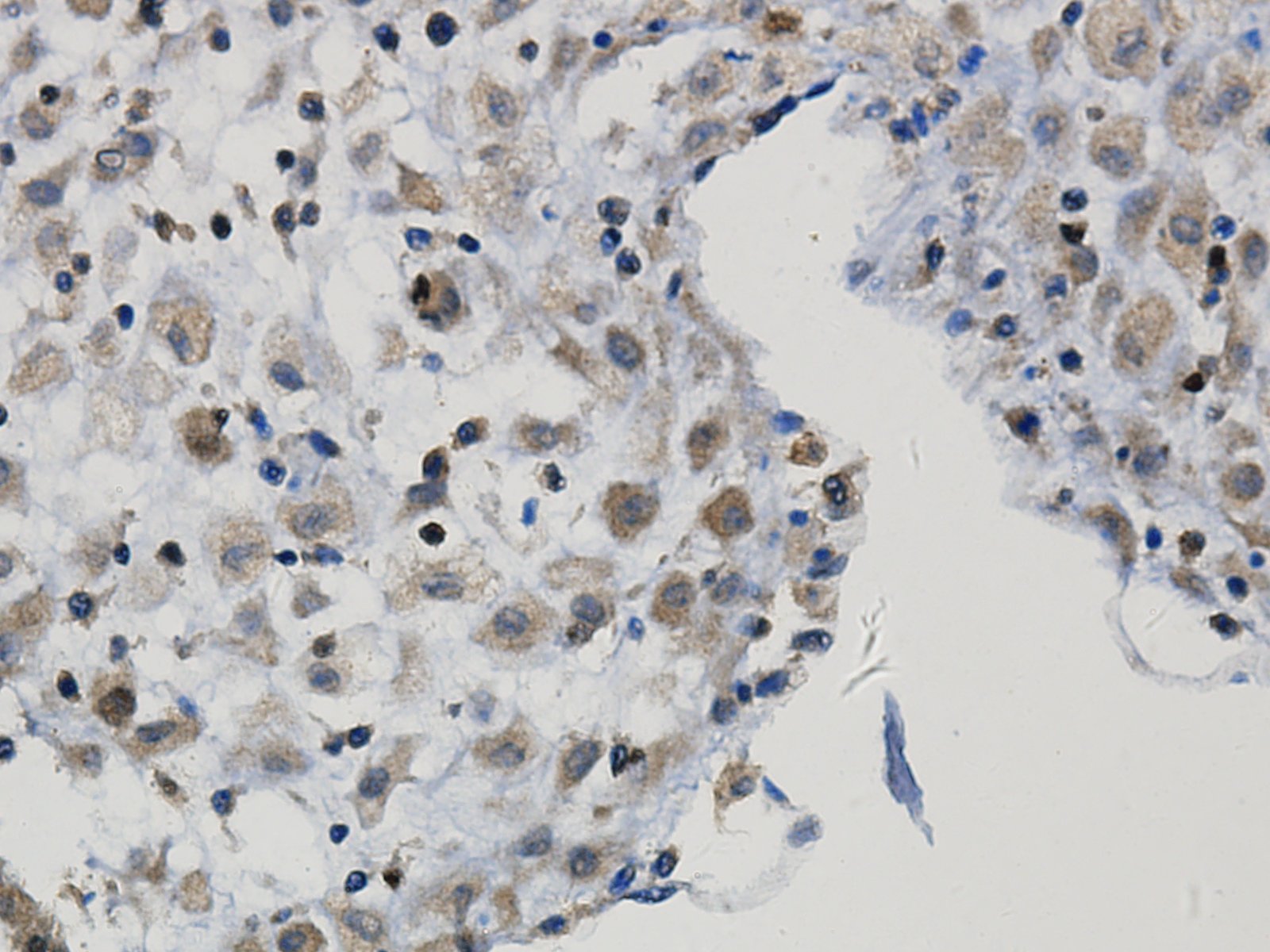

Supplement: Supplementary file 4 — Source data Fig. 2 [file 44318_2024_220_MOESM4_ESM.zip › Figure2/2A/RSA-CDKN1A-400-2.jpg]

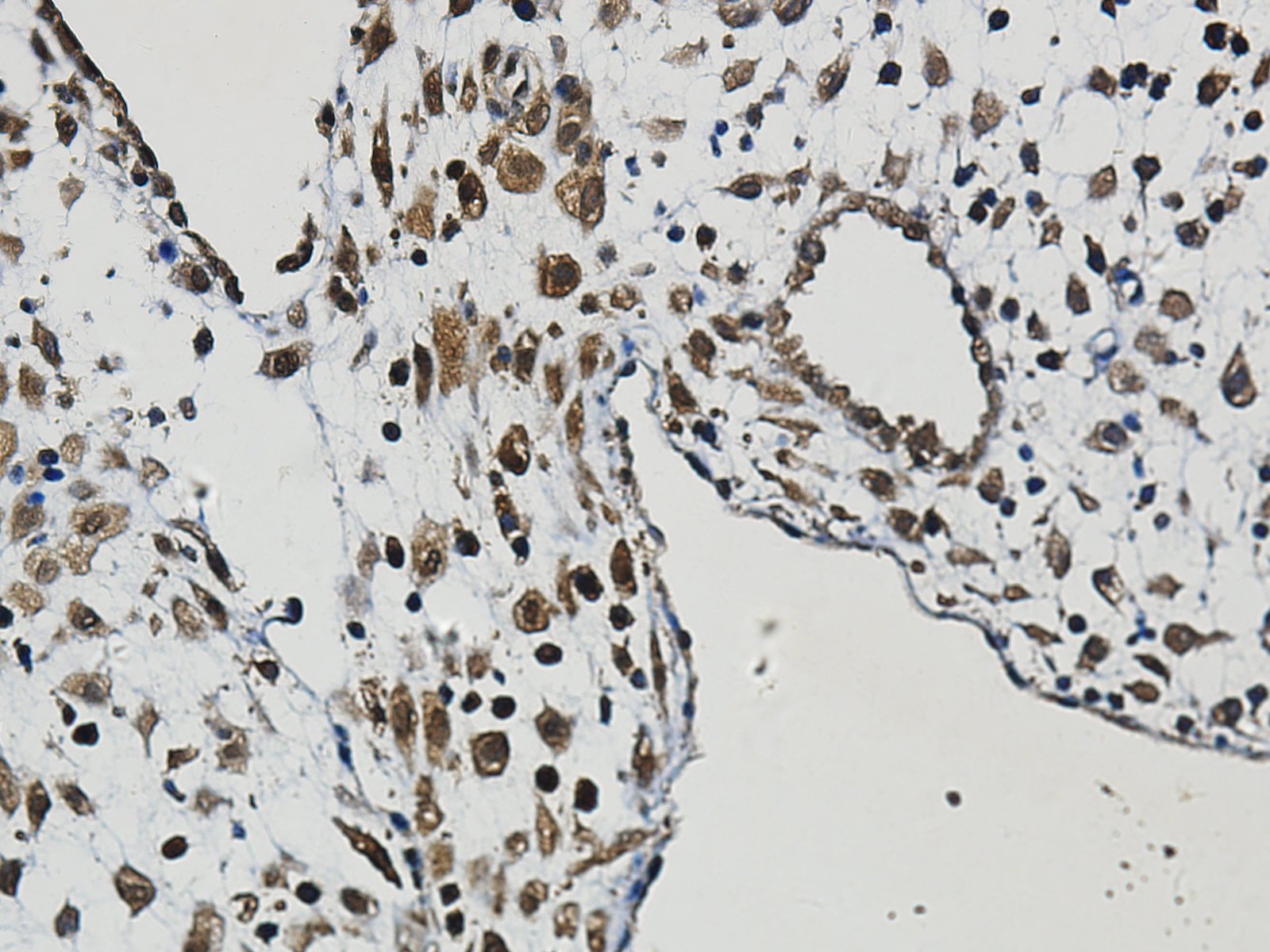

Supplement: Supplementary file 4 — Source data Fig. 2 [file 44318_2024_220_MOESM4_ESM.zip › Figure2/2A/RSA-CDKN1A-400-3.jpg]

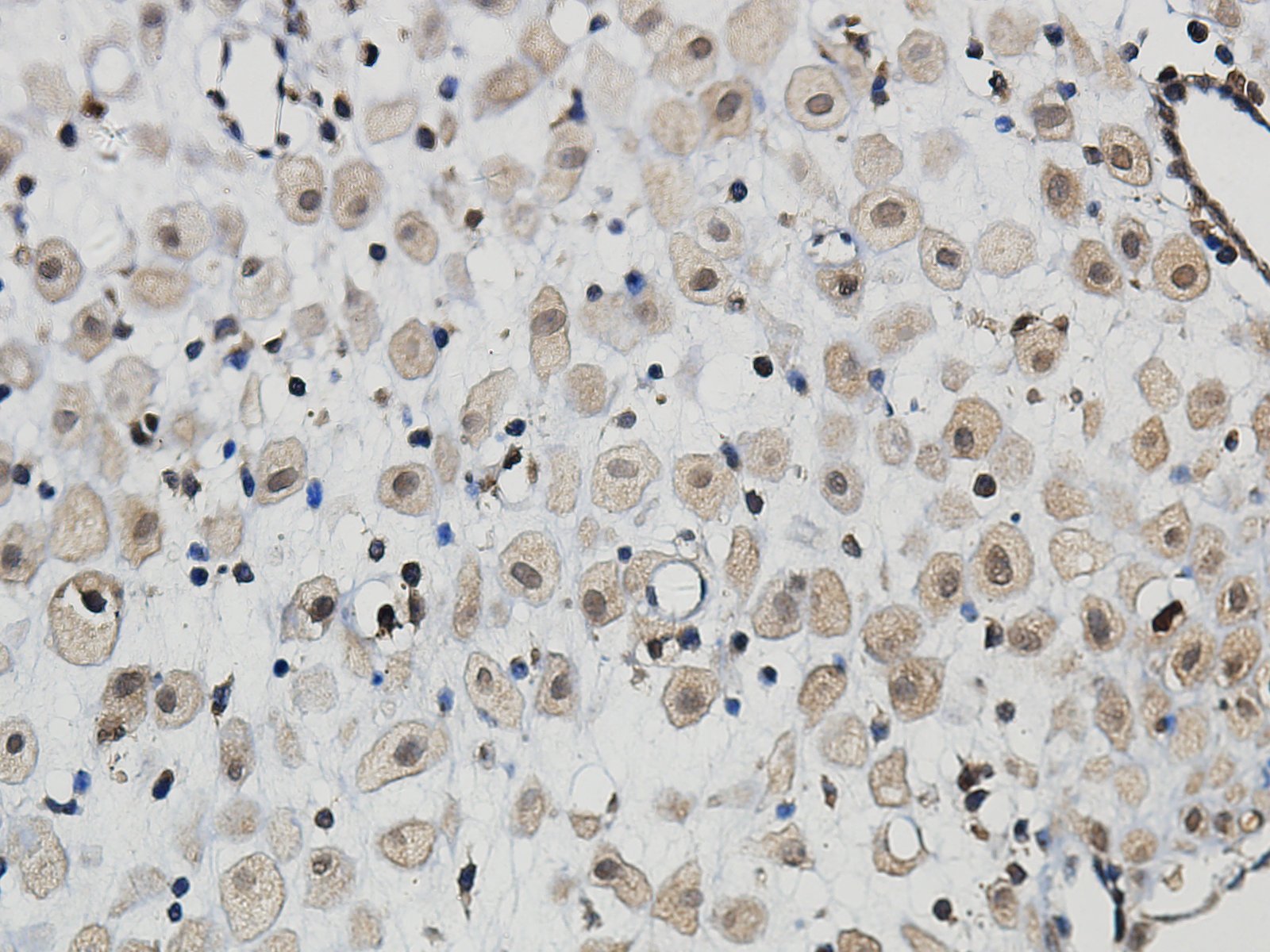

Supplement: Supplementary file 4 — Source data Fig. 2 [file 44318_2024_220_MOESM4_ESM.zip › Figure2/2A/RSA-CDKN1A-400-4.jpg]

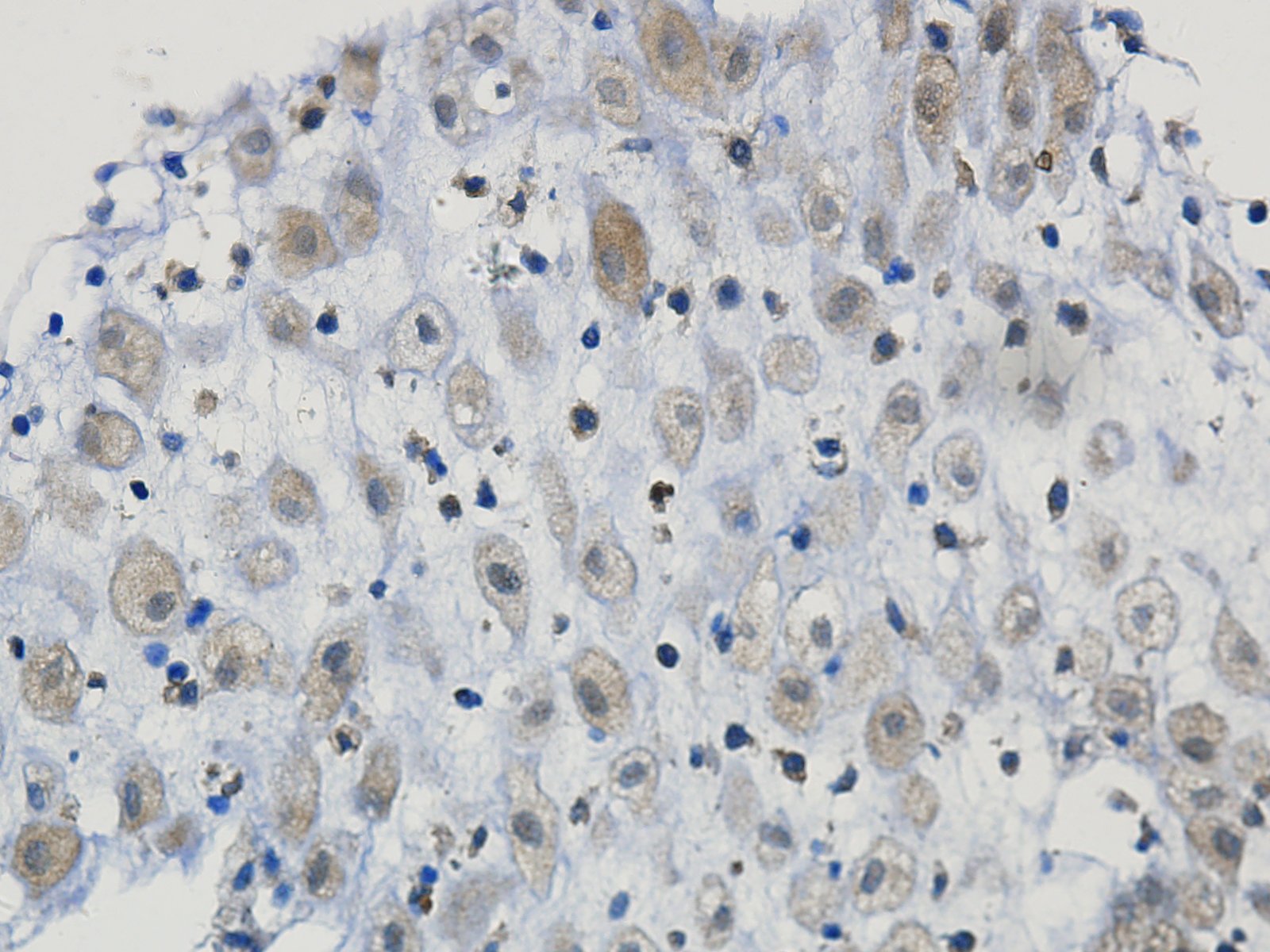

Supplement: Supplementary file 4 — Source data Fig. 2 [file 44318_2024_220_MOESM4_ESM.zip › Figure2/2A/RSA-CDKN1A-400-5.jpg]

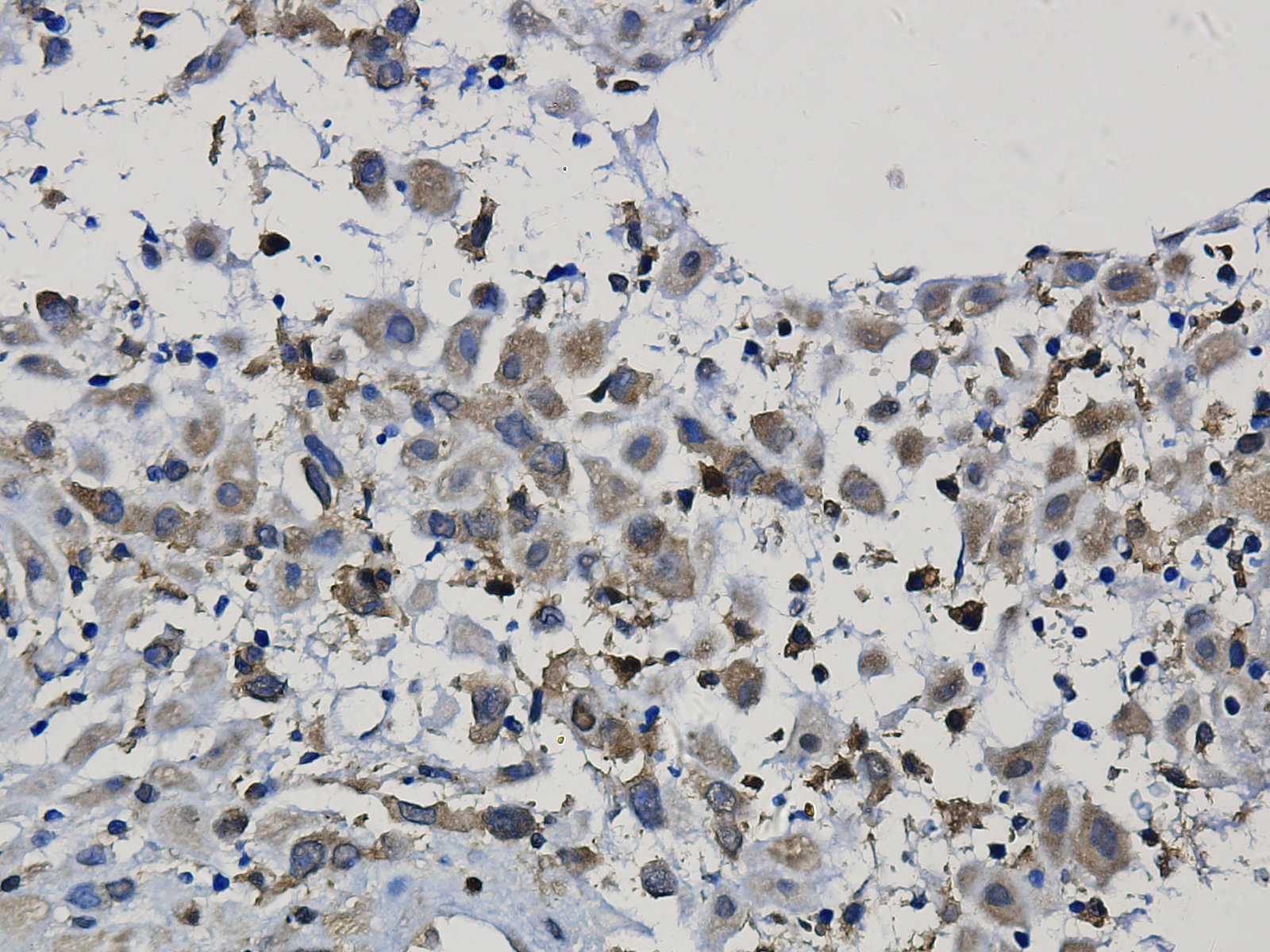

Supplement: Supplementary file 4 — Source data Fig. 2 [file 44318_2024_220_MOESM4_ESM.zip › Figure2/2A/RSA-CDKN1A-400-6.jpg]

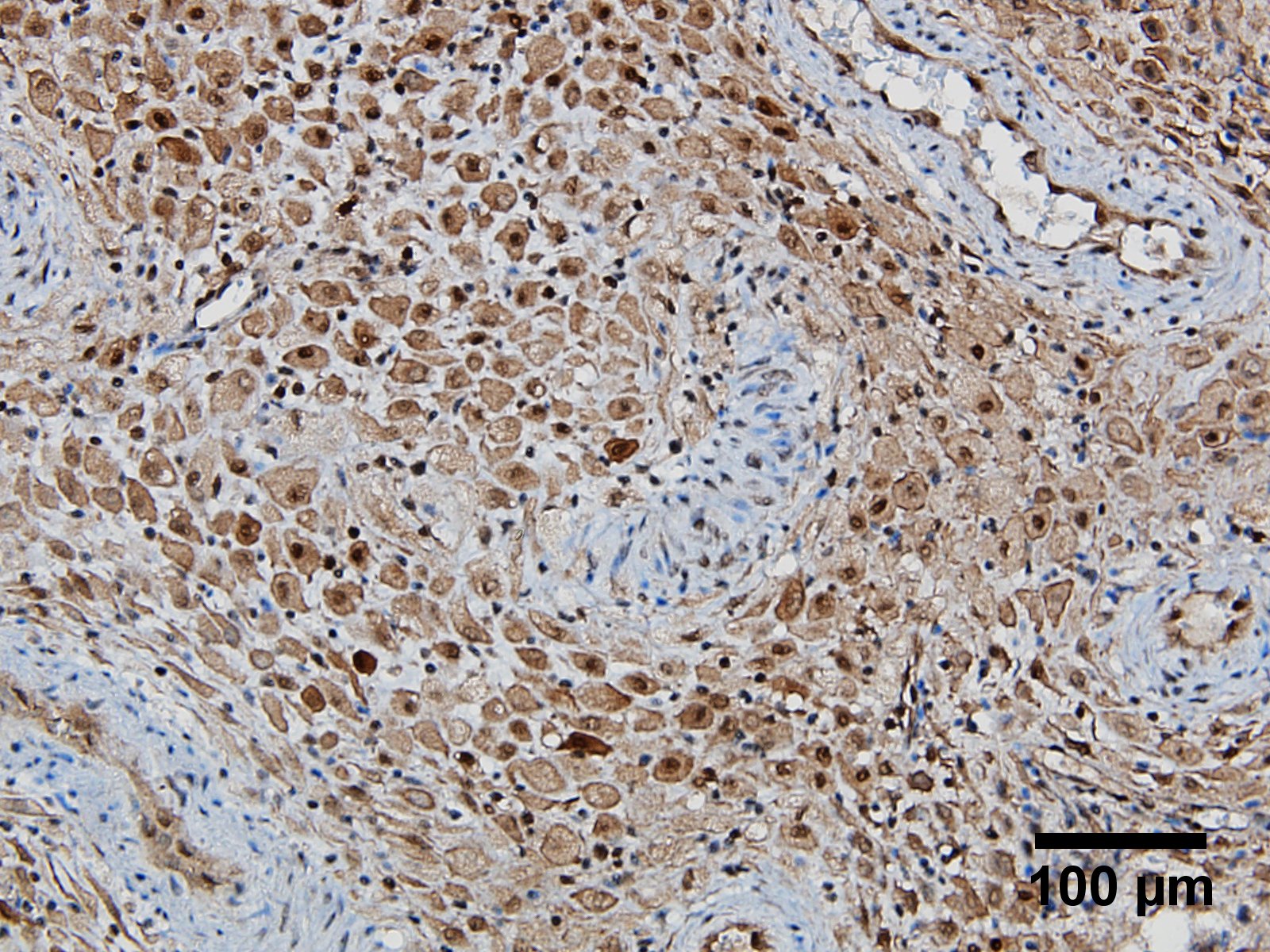

Supplement: Supplementary file 4 — Source data Fig. 2 [file 44318_2024_220_MOESM4_ESM.zip › Figure2/2A/RSA-CDKN2A-200-1.jpg]

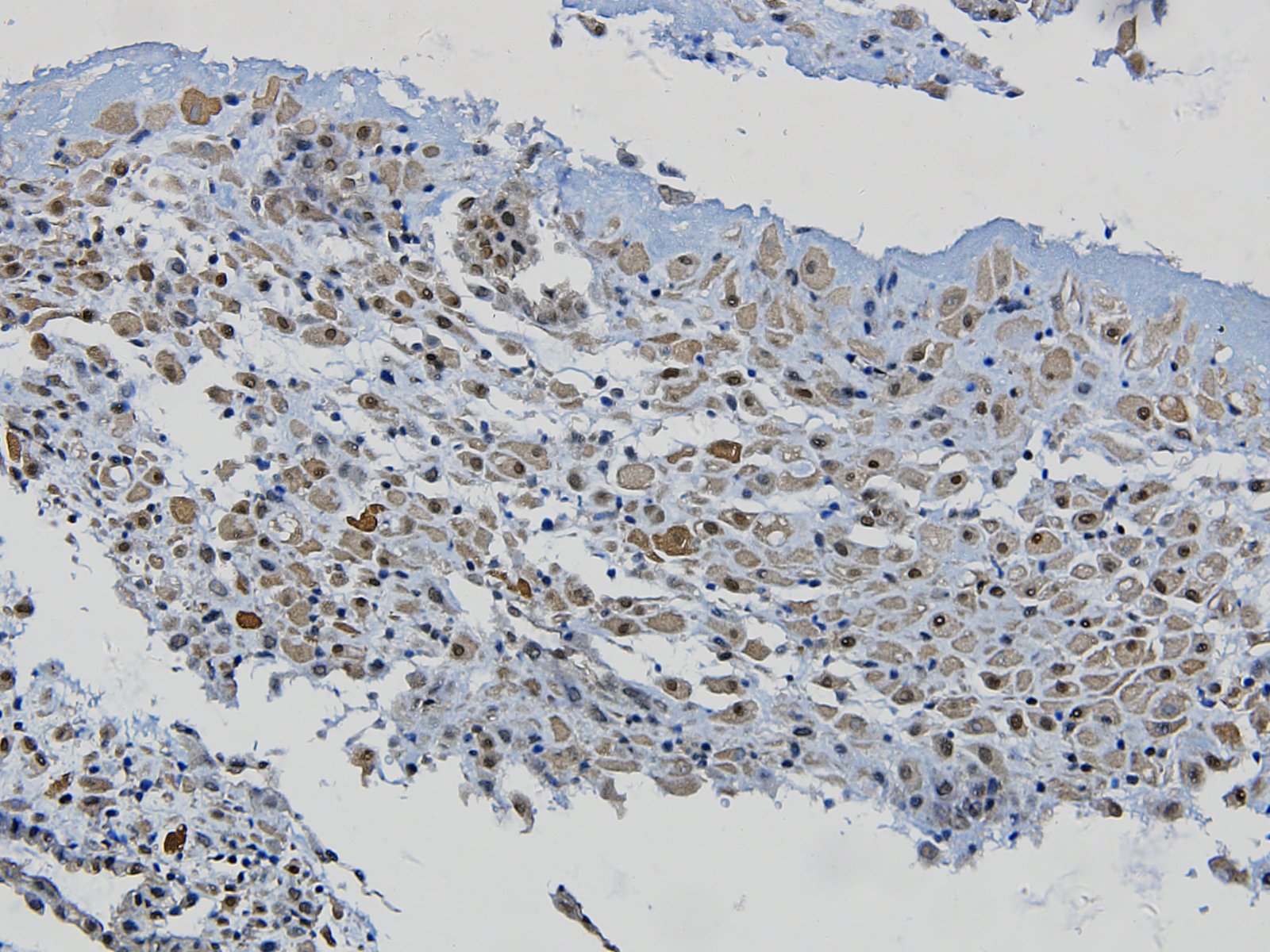

Supplement: Supplementary file 4 — Source data Fig. 2 [file 44318_2024_220_MOESM4_ESM.zip › Figure2/2A/RSA-CDKN2A-200-2.jpg]

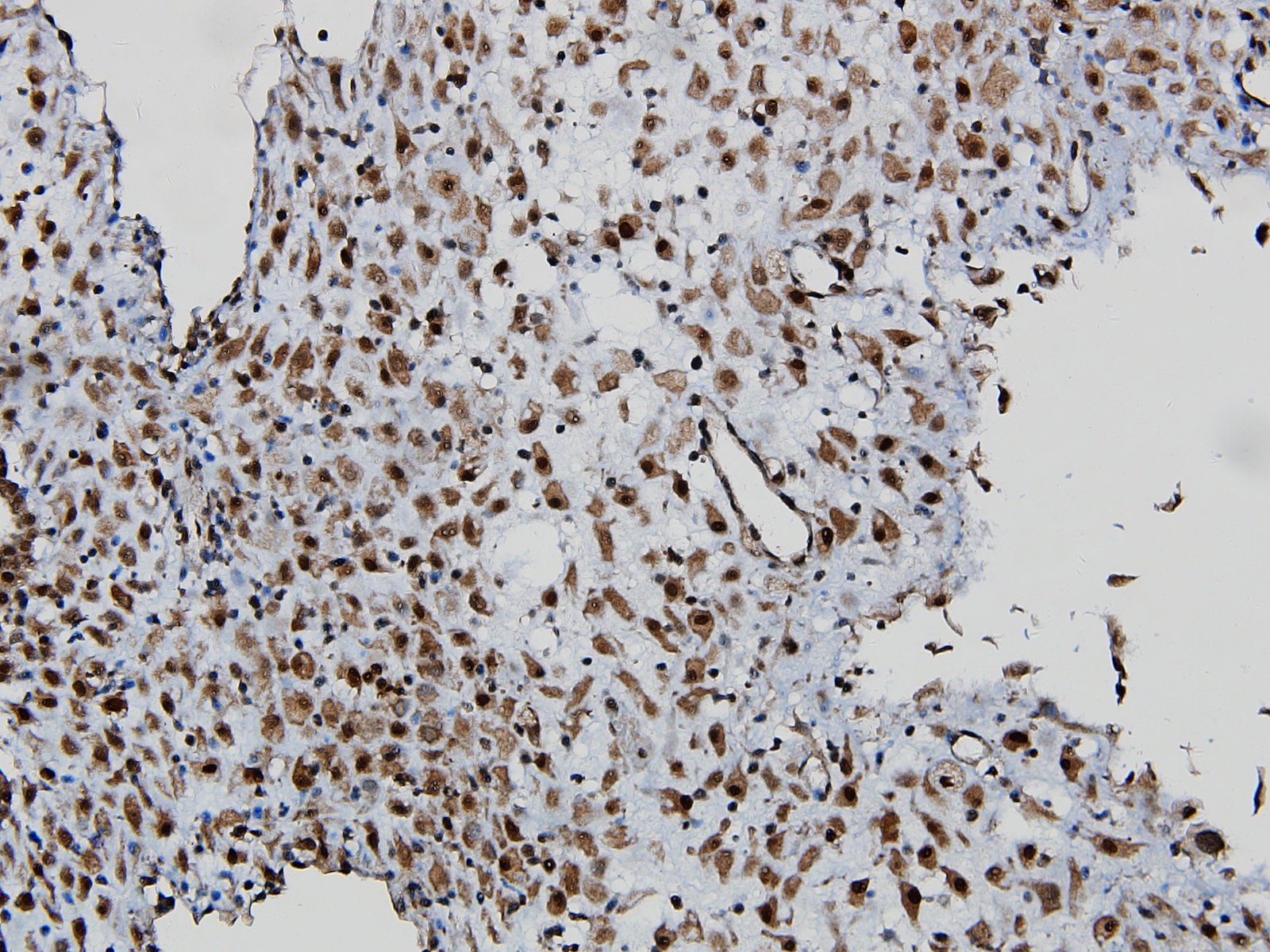

Supplement: Supplementary file 4 — Source data Fig. 2 [file 44318_2024_220_MOESM4_ESM.zip › Figure2/2A/RSA-CDKN2A-200-3.jpg]

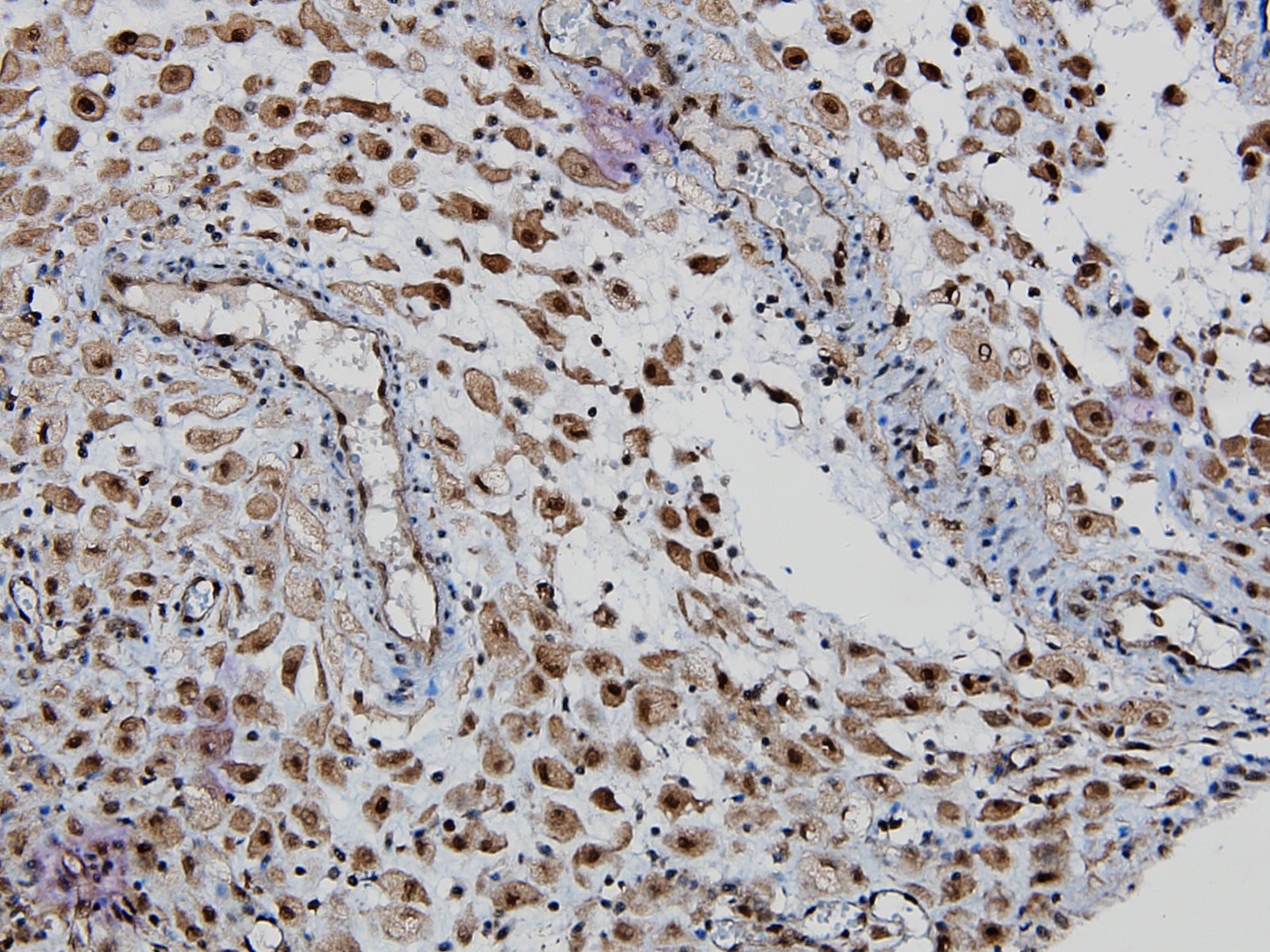

Supplement: Supplementary file 4 — Source data Fig. 2 [file 44318_2024_220_MOESM4_ESM.zip › Figure2/2A/RSA-CDKN2A-200-4.jpg]

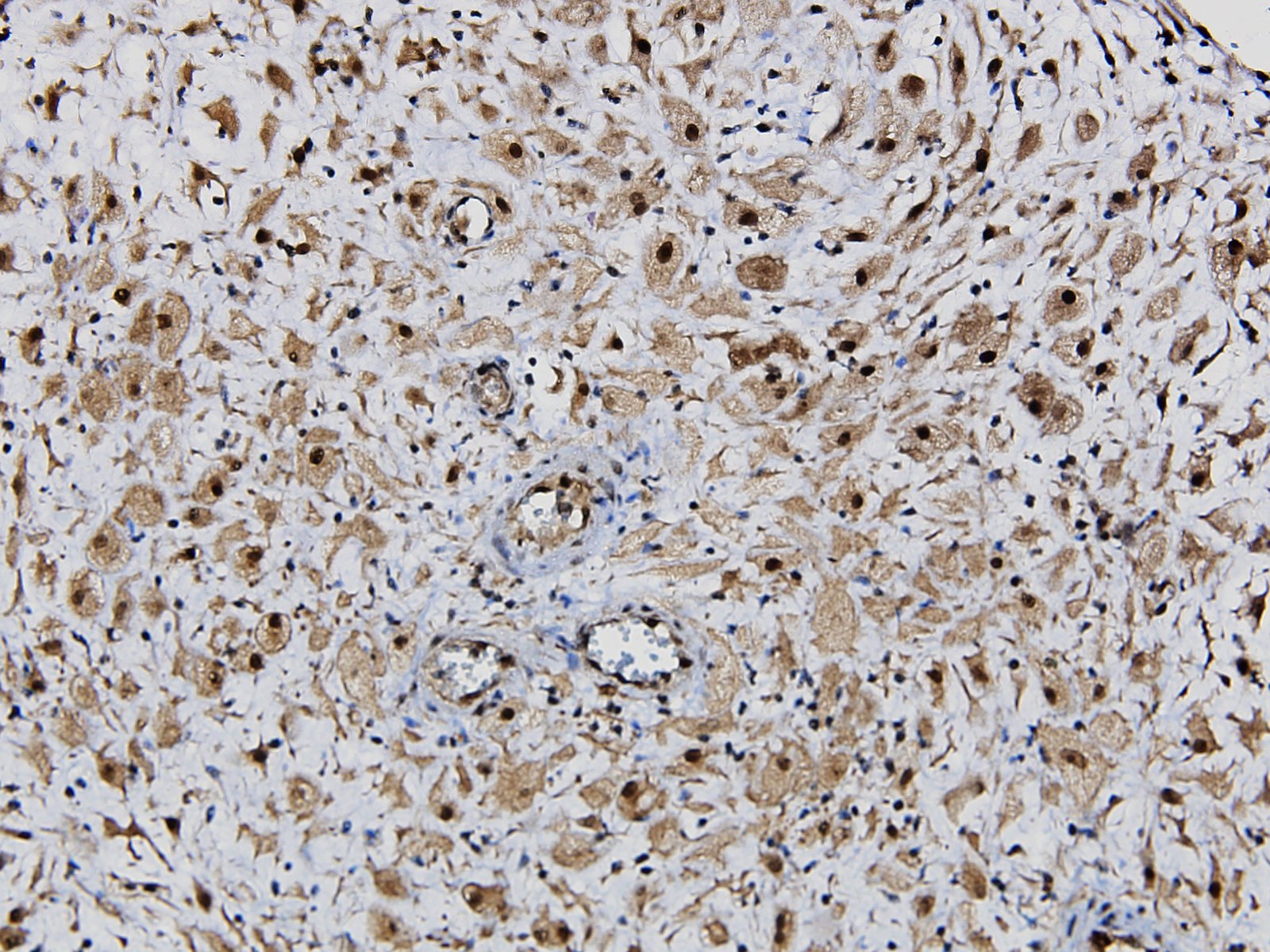

Supplement: Supplementary file 4 — Source data Fig. 2 [file 44318_2024_220_MOESM4_ESM.zip › Figure2/2A/RSA-CDKN2A-200-5.jpg]

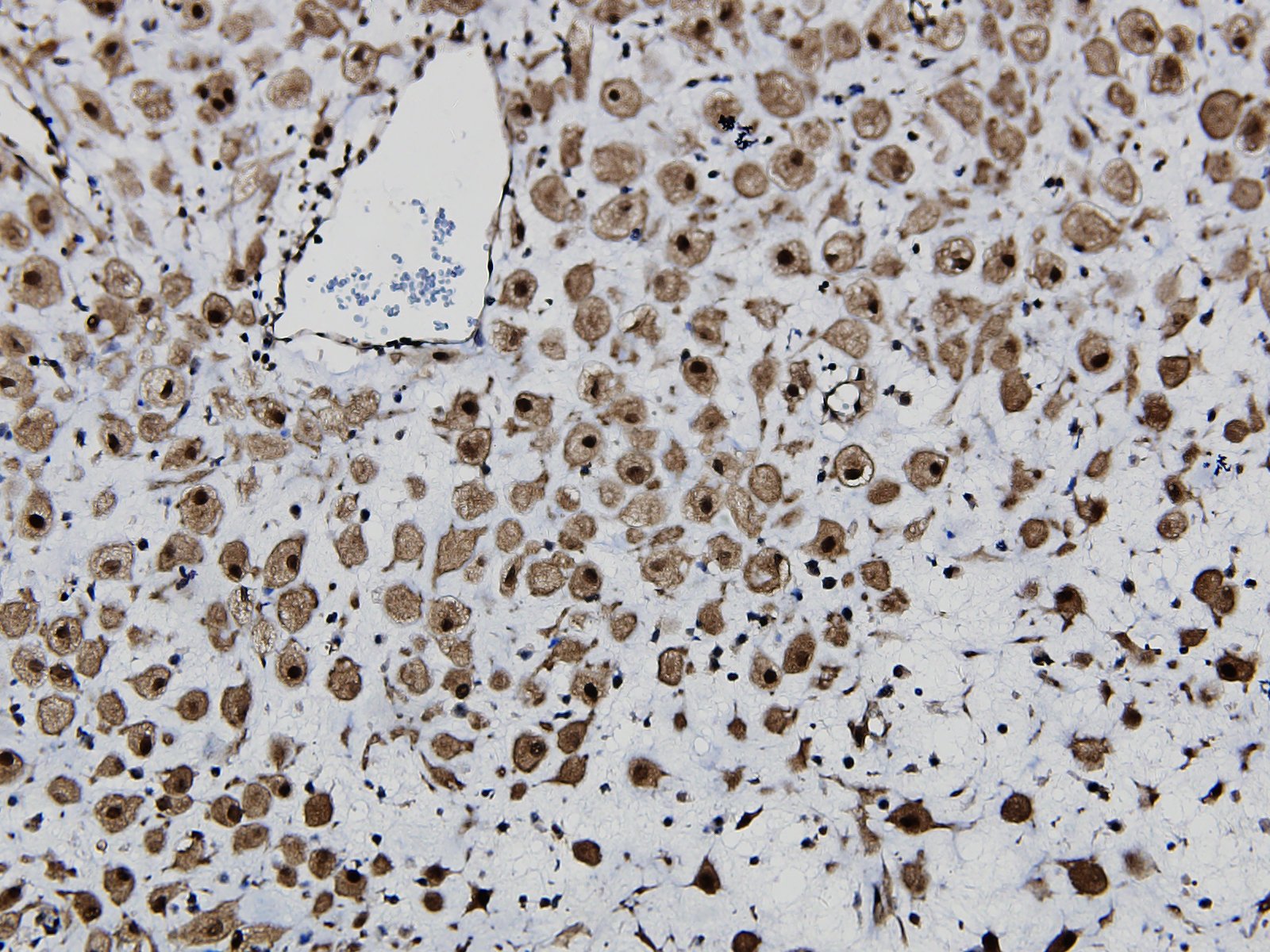

Supplement: Supplementary file 4 — Source data Fig. 2 [file 44318_2024_220_MOESM4_ESM.zip › Figure2/2A/RSA-CDKN2A-200-6.jpg]

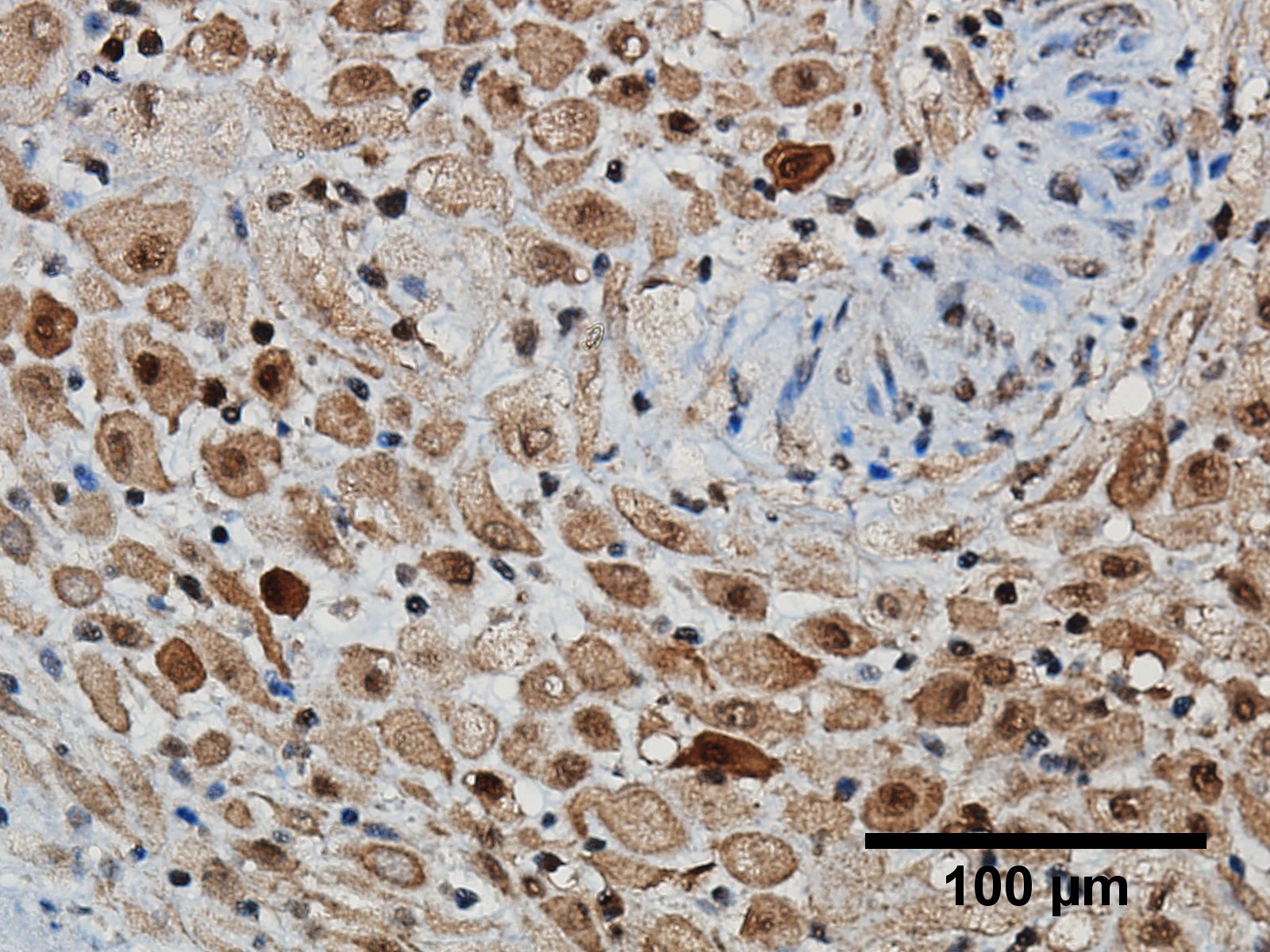

Supplement: Supplementary file 4 — Source data Fig. 2 [file 44318_2024_220_MOESM4_ESM.zip › Figure2/2A/RSA-CDKN2A-400-1.jpg]

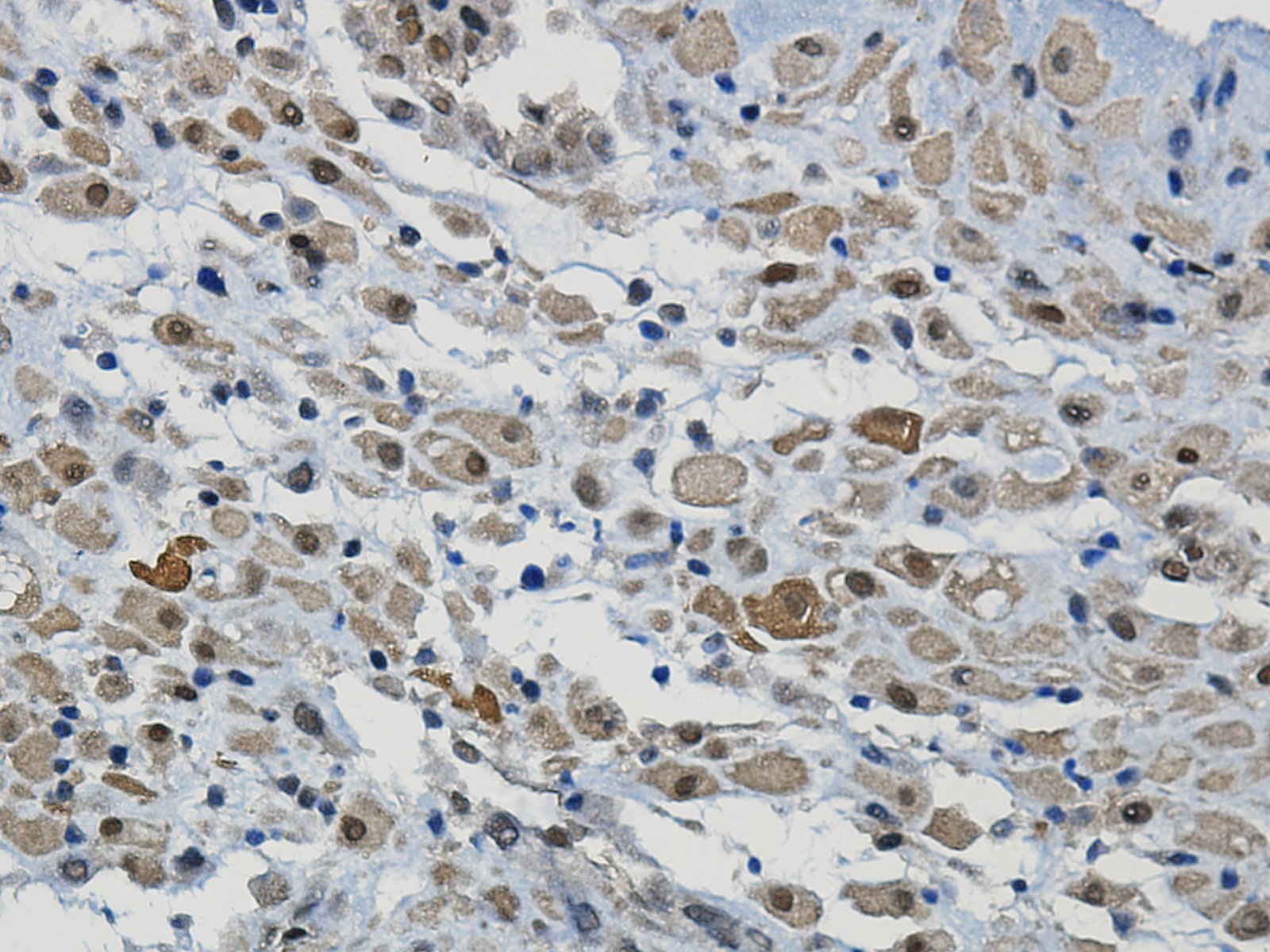

Supplement: Supplementary file 4 — Source data Fig. 2 [file 44318_2024_220_MOESM4_ESM.zip › Figure2/2A/RSA-CDKN2A-400-2.jpg]

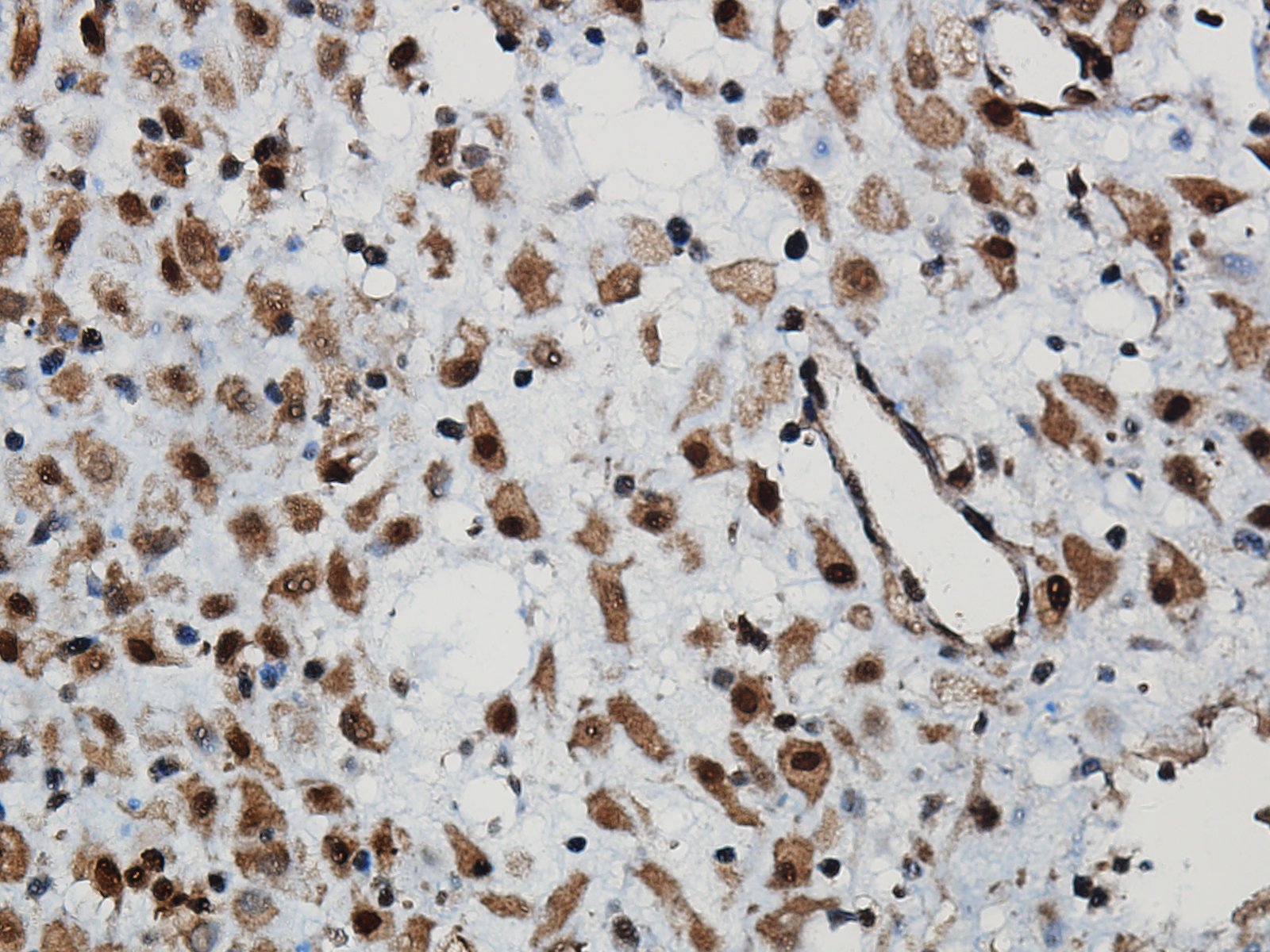

Supplement: Supplementary file 4 — Source data Fig. 2 [file 44318_2024_220_MOESM4_ESM.zip › Figure2/2A/RSA-CDKN2A-400-3.jpg]

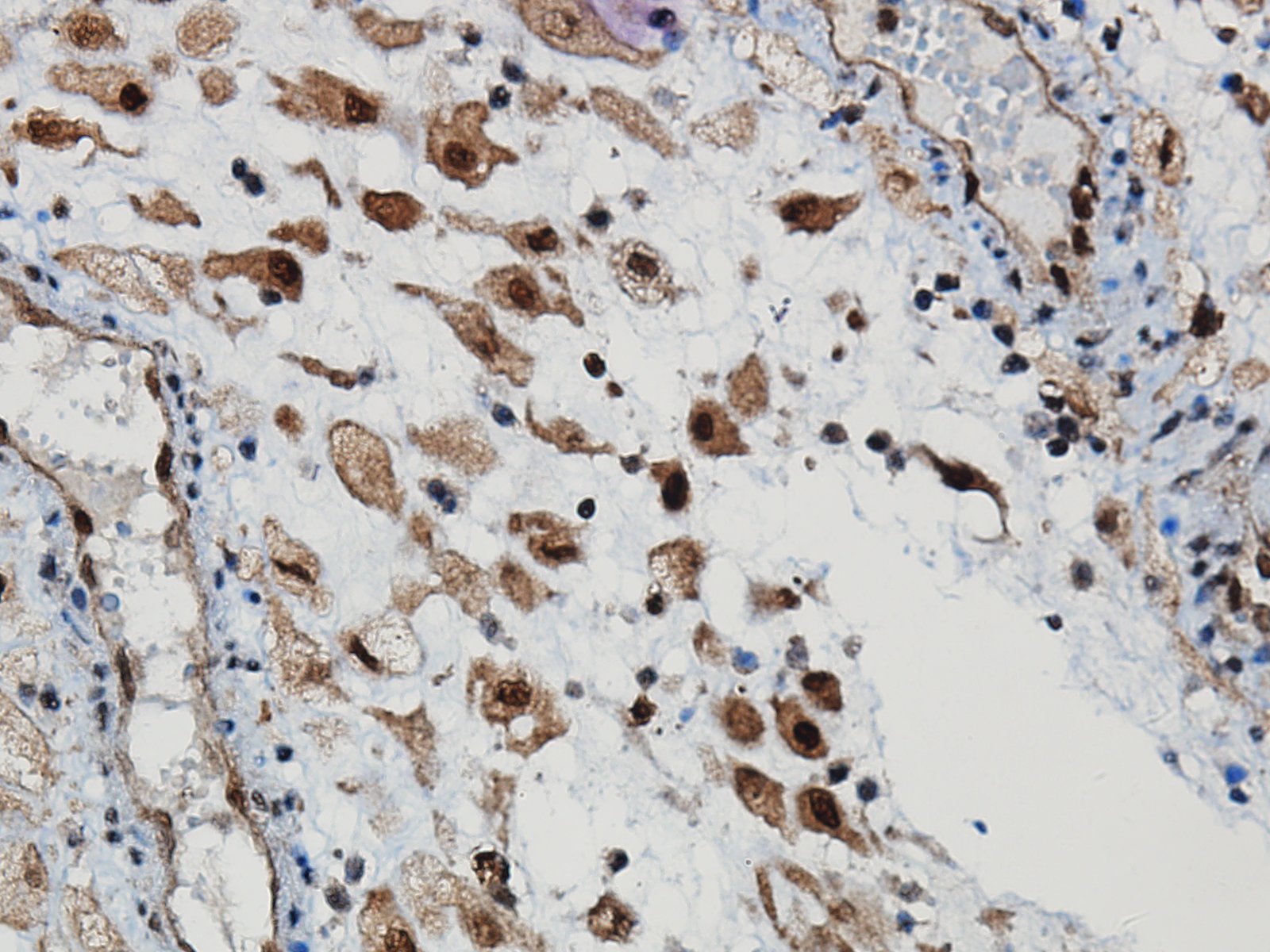

Supplement: Supplementary file 4 — Source data Fig. 2 [file 44318_2024_220_MOESM4_ESM.zip › Figure2/2A/RSA-CDKN2A-400-4.jpg]

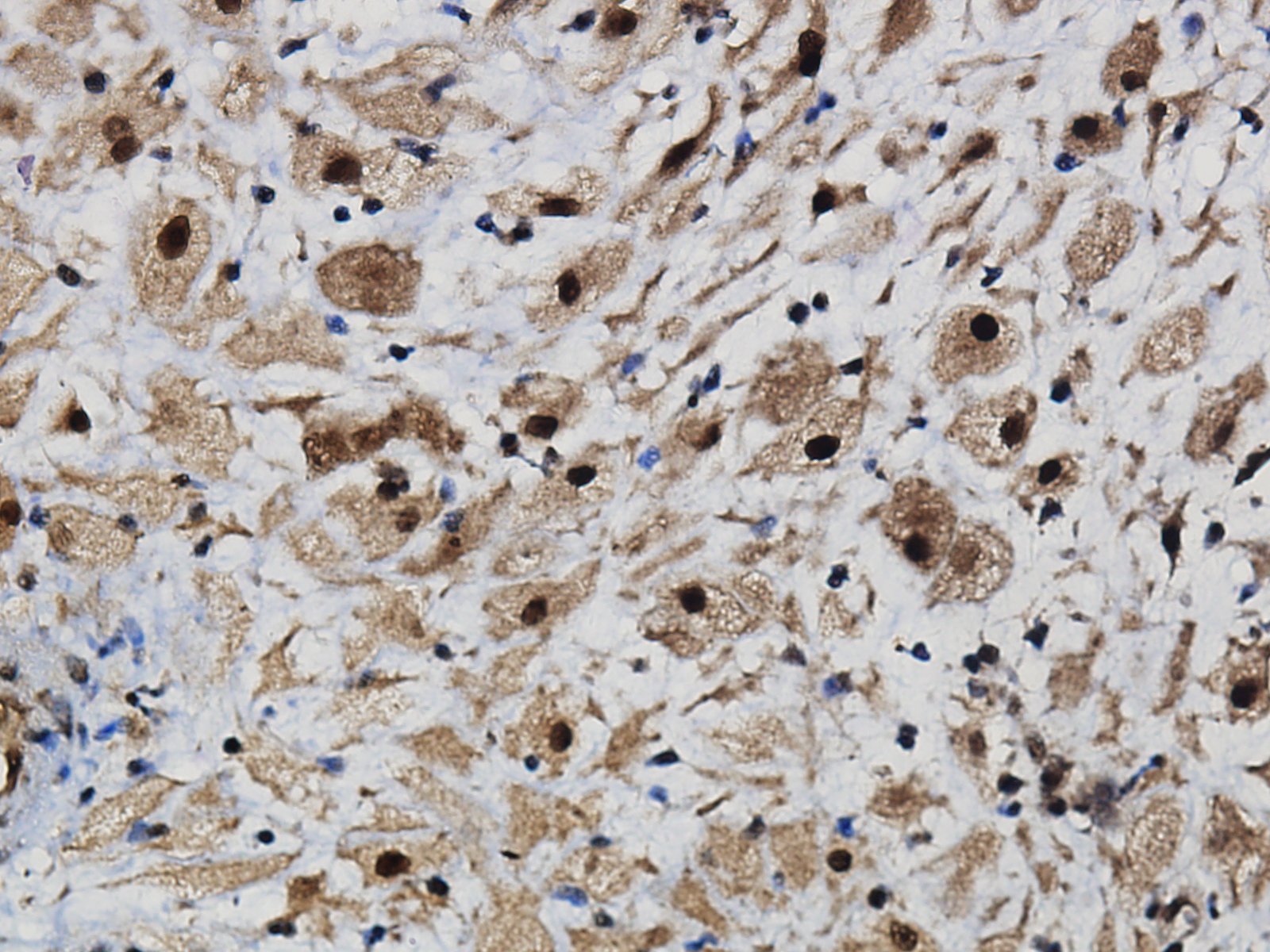

Supplement: Supplementary file 4 — Source data Fig. 2 [file 44318_2024_220_MOESM4_ESM.zip › Figure2/2A/RSA-CDKN2A-400-5.jpg]

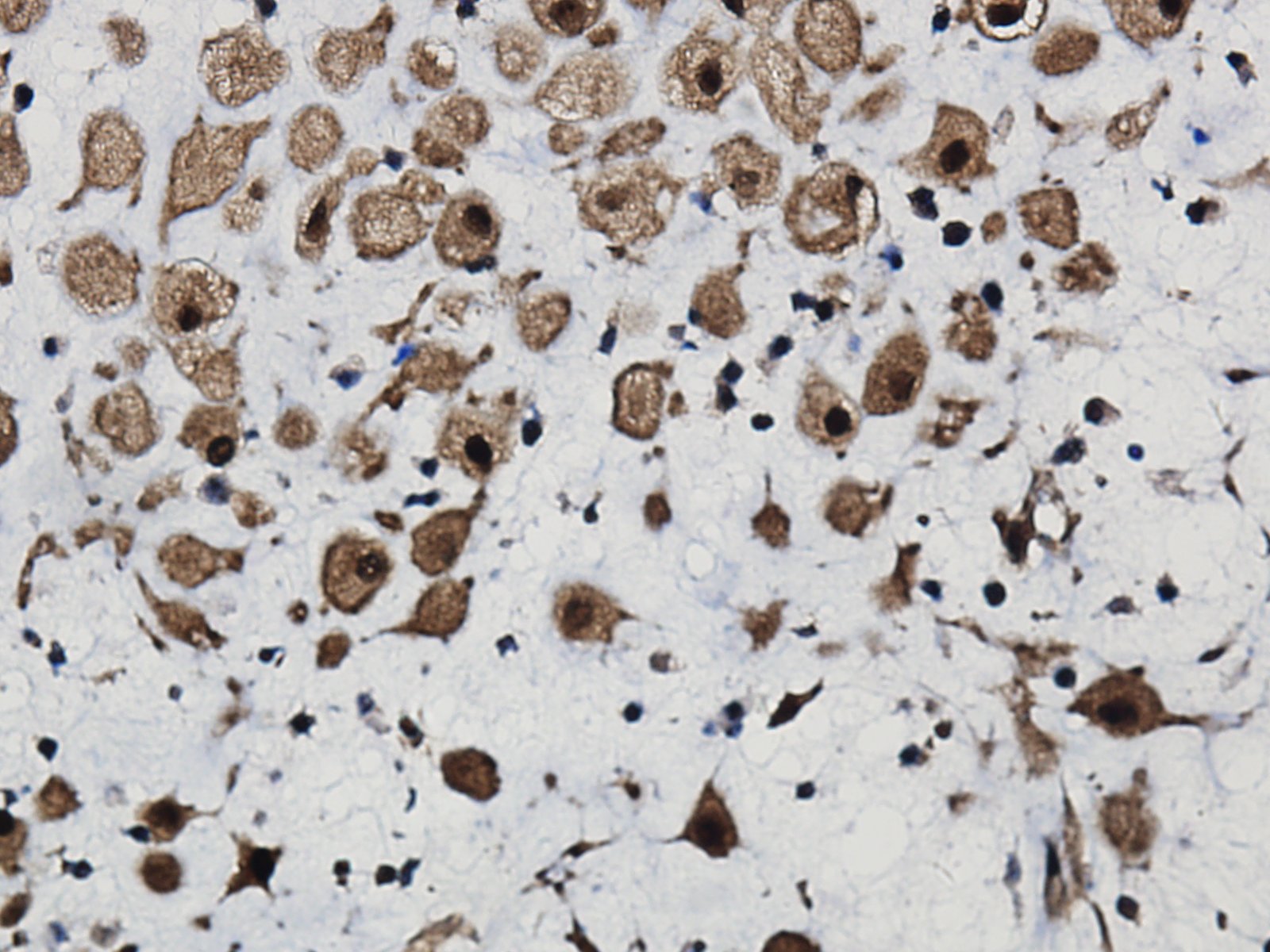

Supplement: Supplementary file 4 — Source data Fig. 2 [file 44318_2024_220_MOESM4_ESM.zip › Figure2/2A/RSA-CDKN2A-400-6.jpg]

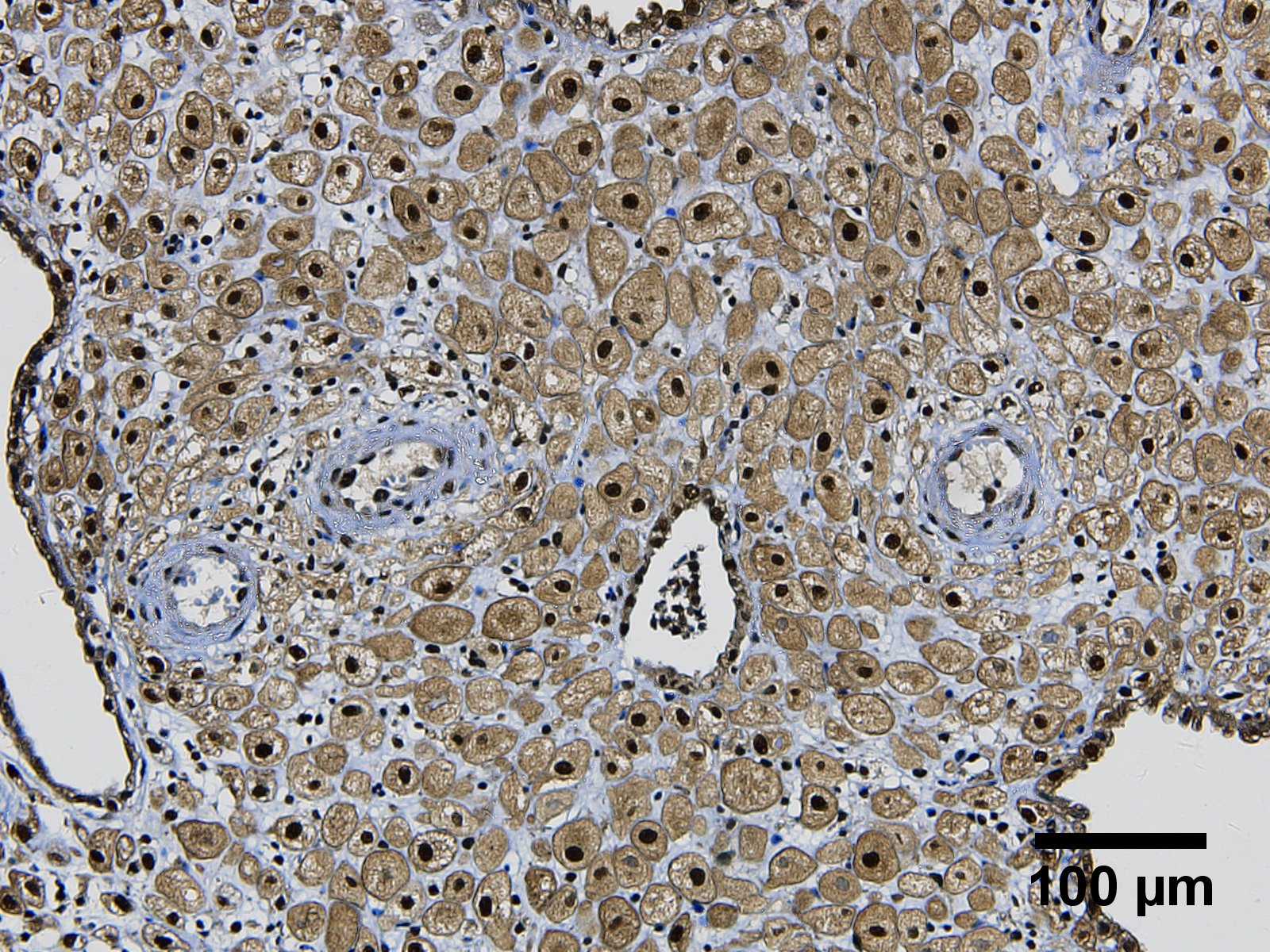

Supplement: Supplementary file 4 — Source data Fig. 2 [file 44318_2024_220_MOESM4_ESM.zip › Figure2/2A/RSA-TP53-200-1.jpg]

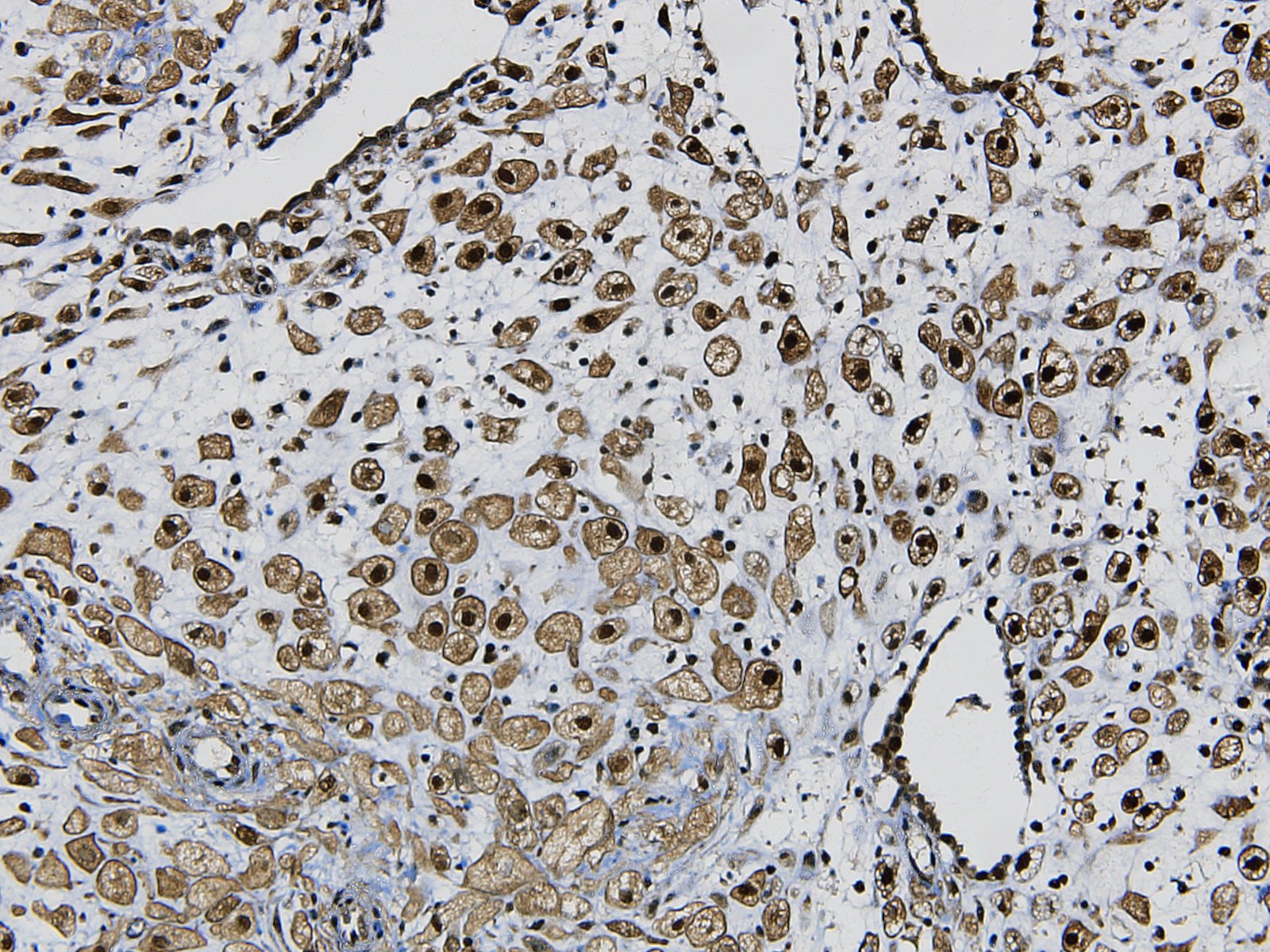

Supplement: Supplementary file 4 — Source data Fig. 2 [file 44318_2024_220_MOESM4_ESM.zip › Figure2/2A/RSA-TP53-200-2.jpg]

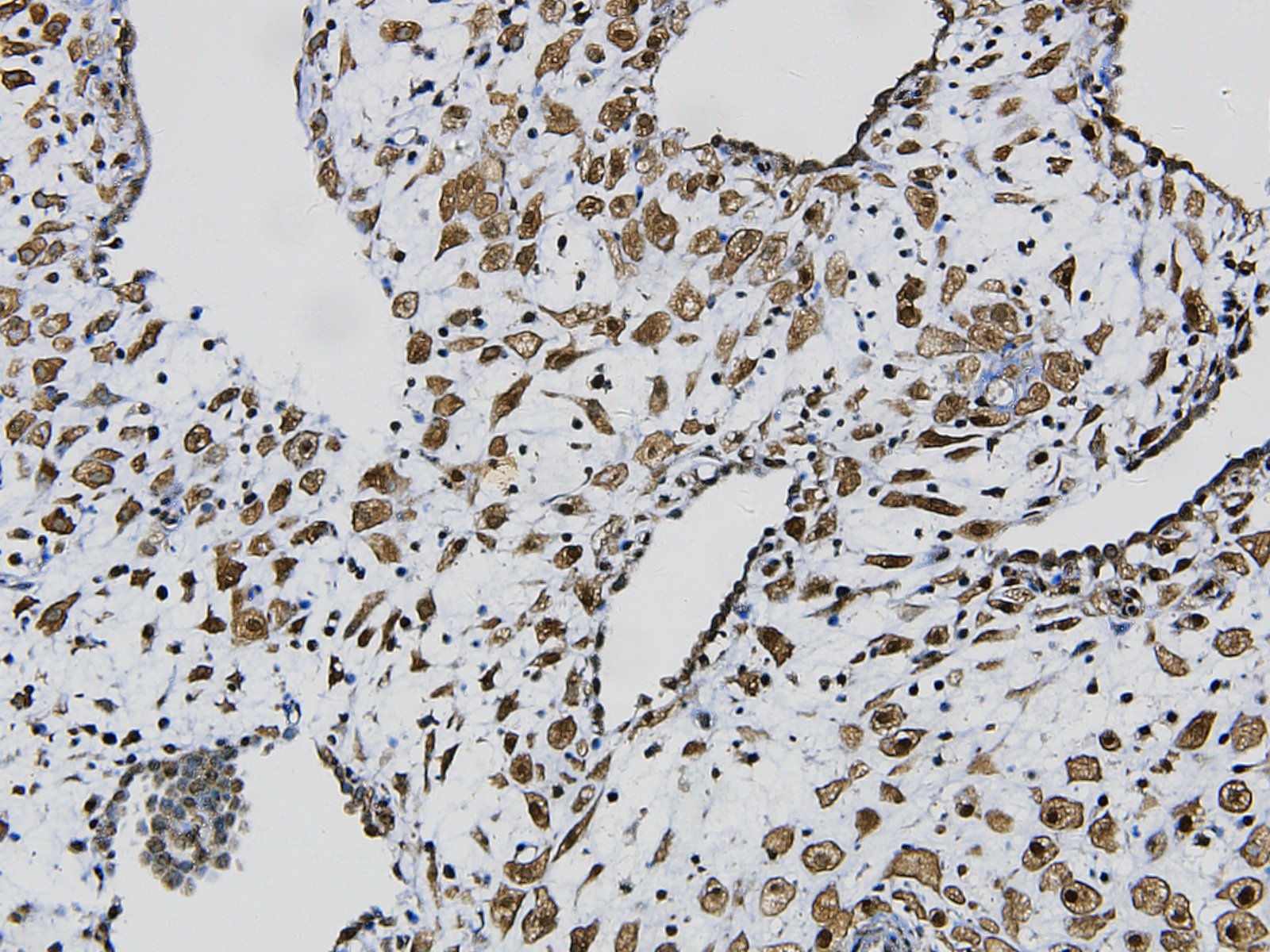

Supplement: Supplementary file 4 — Source data Fig. 2 [file 44318_2024_220_MOESM4_ESM.zip › Figure2/2A/RSA-TP53-200-3.jpg]

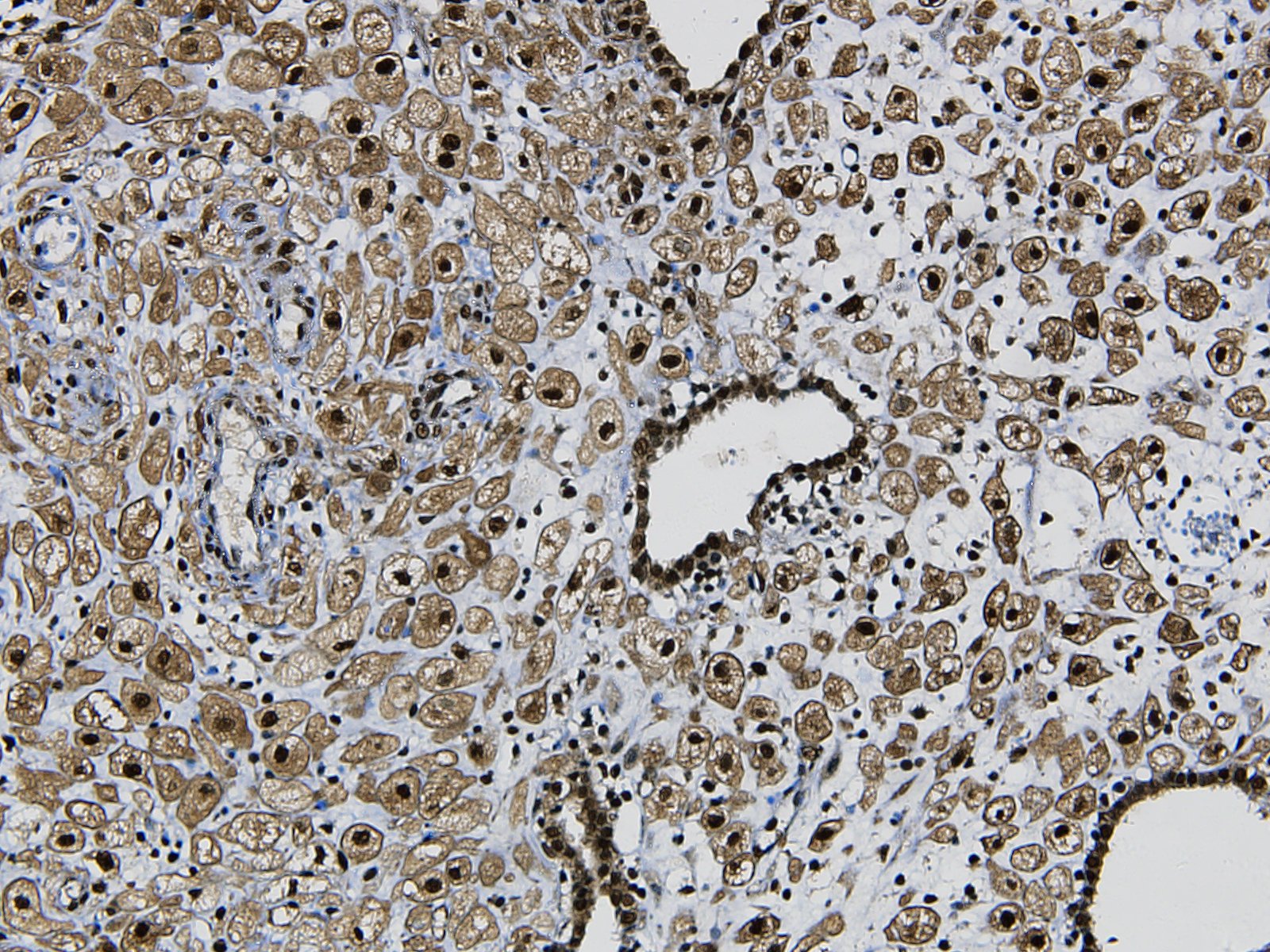

Supplement: Supplementary file 4 — Source data Fig. 2 [file 44318_2024_220_MOESM4_ESM.zip › Figure2/2A/RSA-TP53-200-4.jpg]

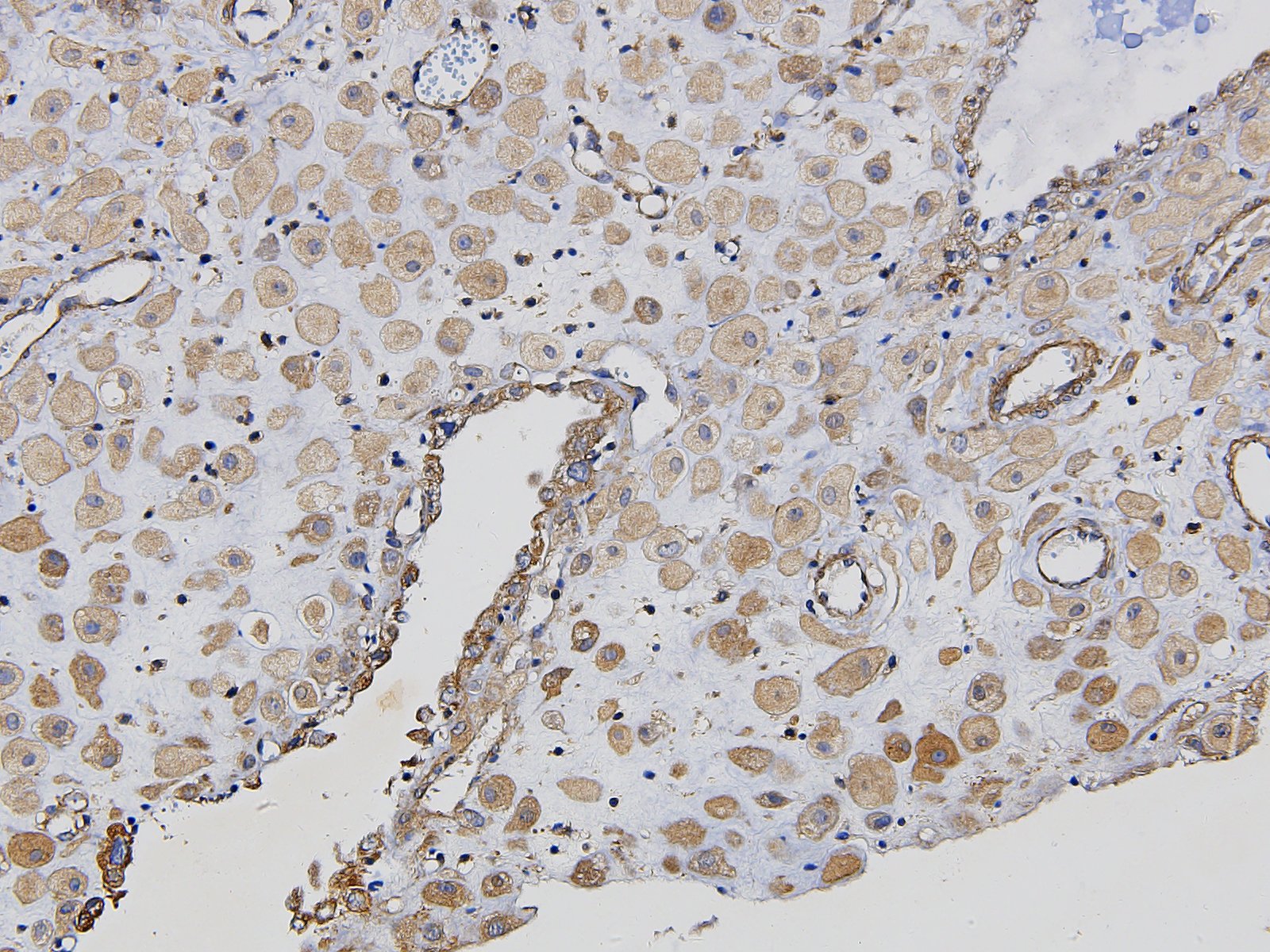

Supplement: Supplementary file 4 — Source data Fig. 2 [file 44318_2024_220_MOESM4_ESM.zip › Figure2/2A/RSA-TP53-200-5.jpg]

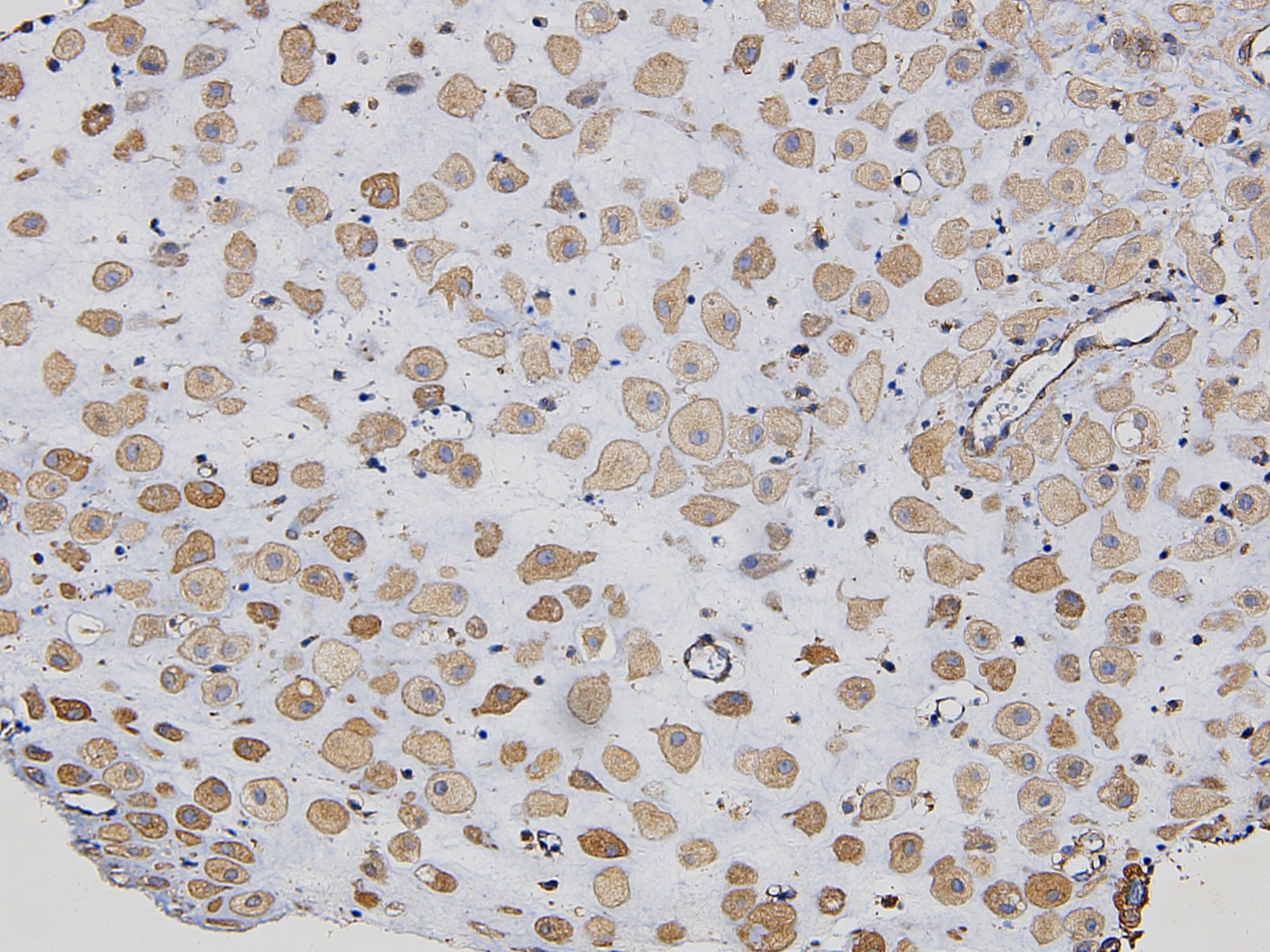

Supplement: Supplementary file 4 — Source data Fig. 2 [file 44318_2024_220_MOESM4_ESM.zip › Figure2/2A/RSA-TP53-200-6.jpg]

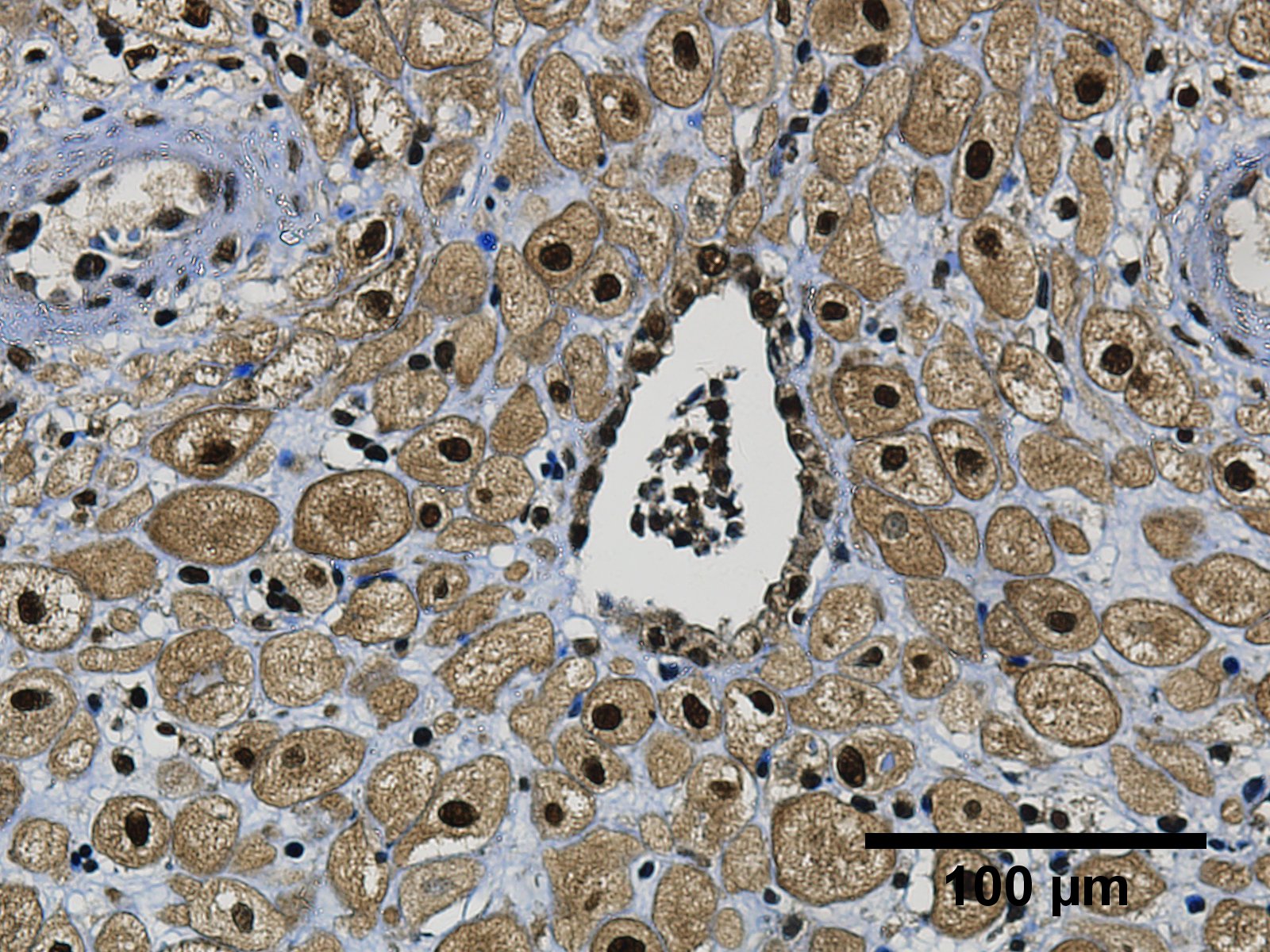

Supplement: Supplementary file 4 — Source data Fig. 2 [file 44318_2024_220_MOESM4_ESM.zip › Figure2/2A/RSA-TP53-400-1.jpg]

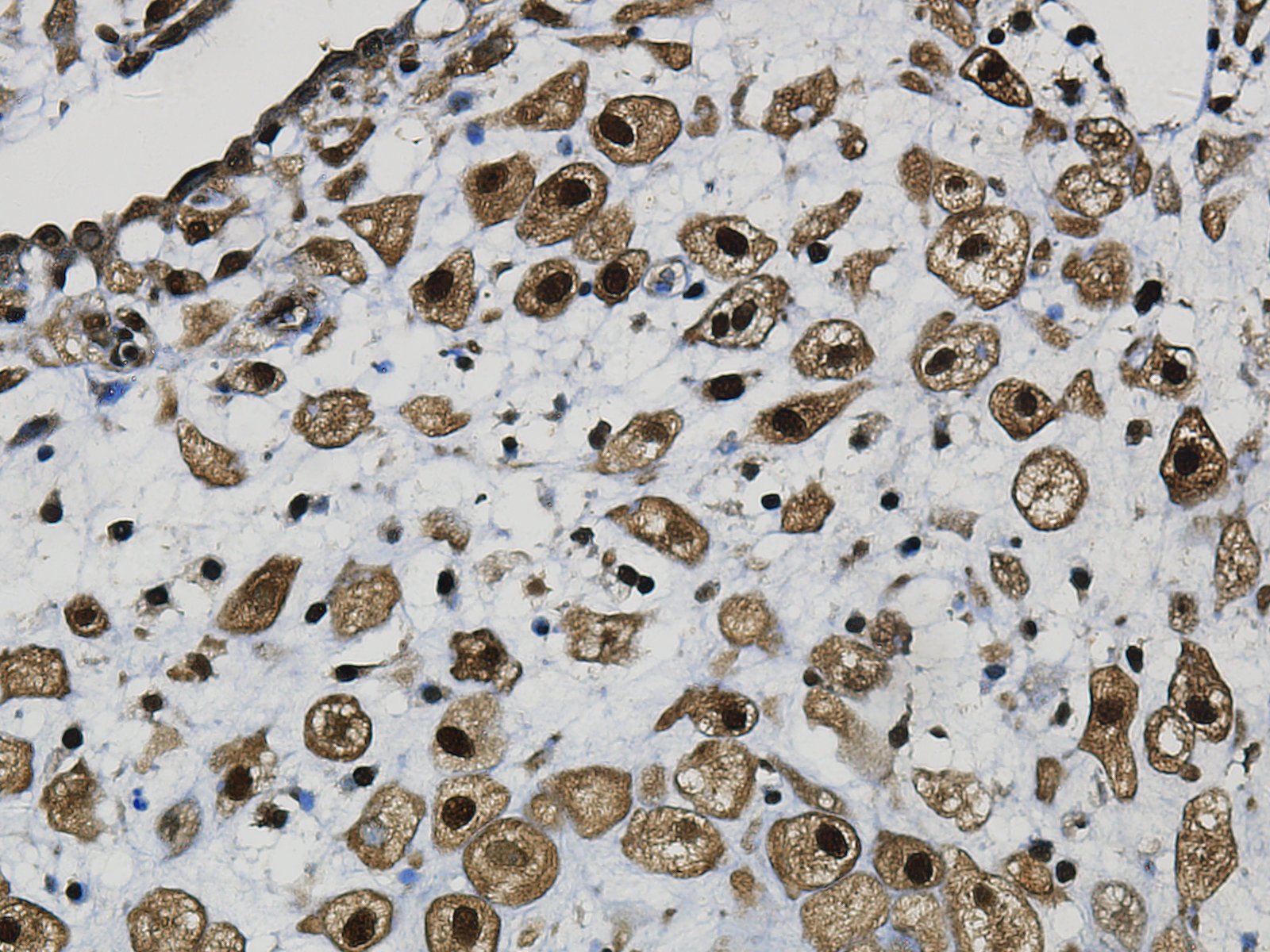

Supplement: Supplementary file 4 — Source data Fig. 2 [file 44318_2024_220_MOESM4_ESM.zip › Figure2/2A/RSA-TP53-400-2.jpg]

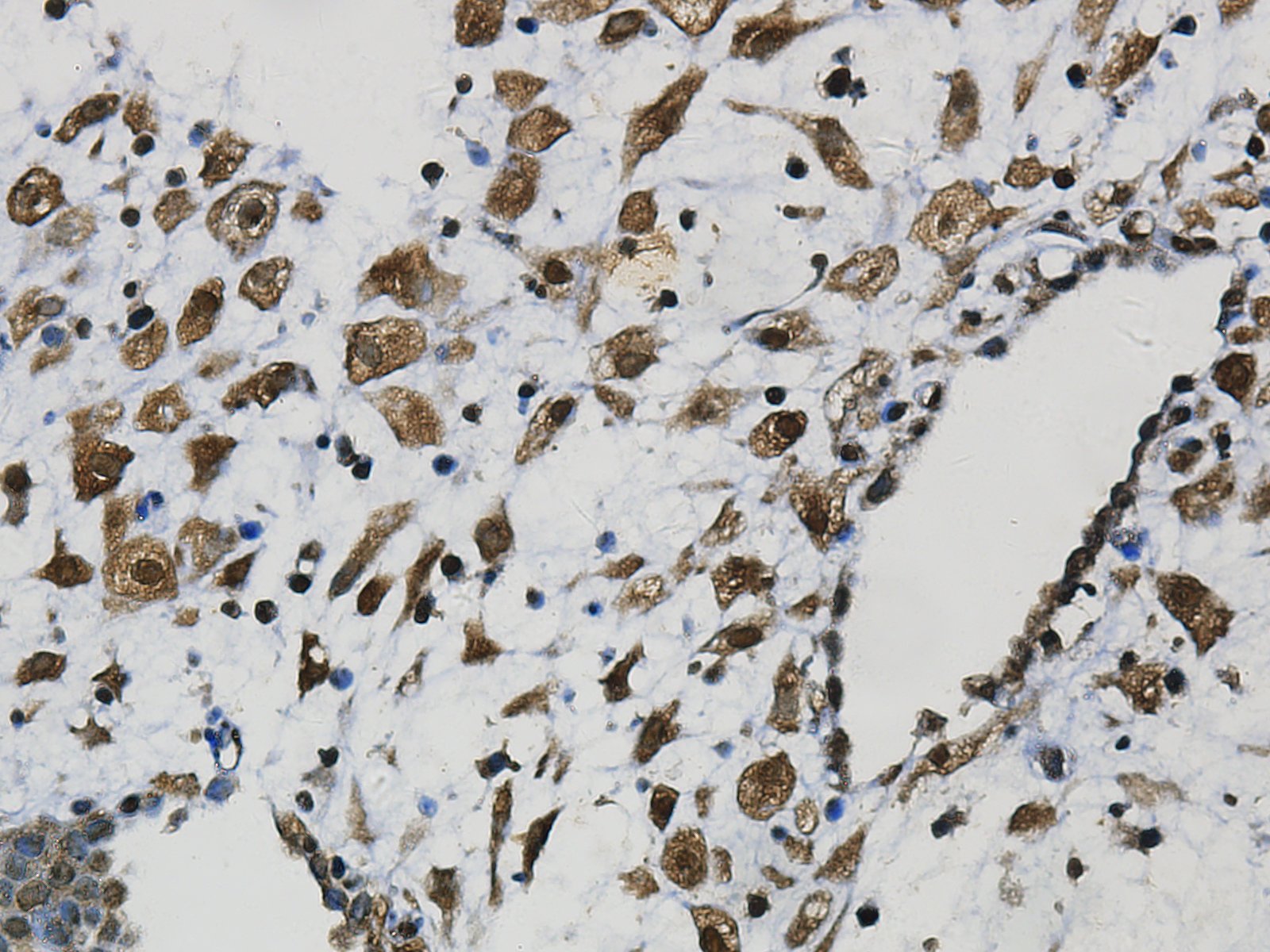

Supplement: Supplementary file 4 — Source data Fig. 2 [file 44318_2024_220_MOESM4_ESM.zip › Figure2/2A/RSA-TP53-400-3.jpg]

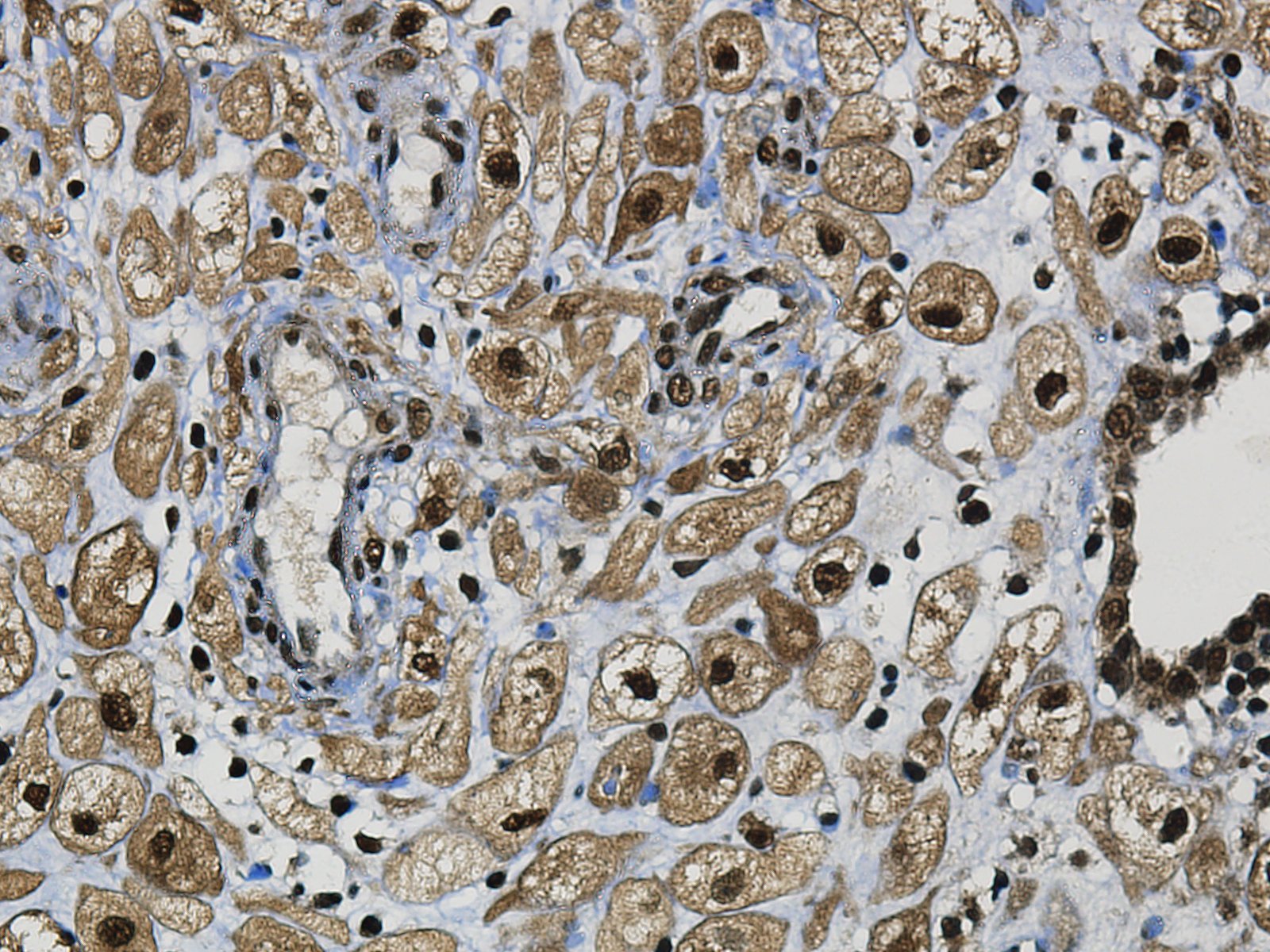

Supplement: Supplementary file 4 — Source data Fig. 2 [file 44318_2024_220_MOESM4_ESM.zip › Figure2/2A/RSA-TP53-400-4.jpg]

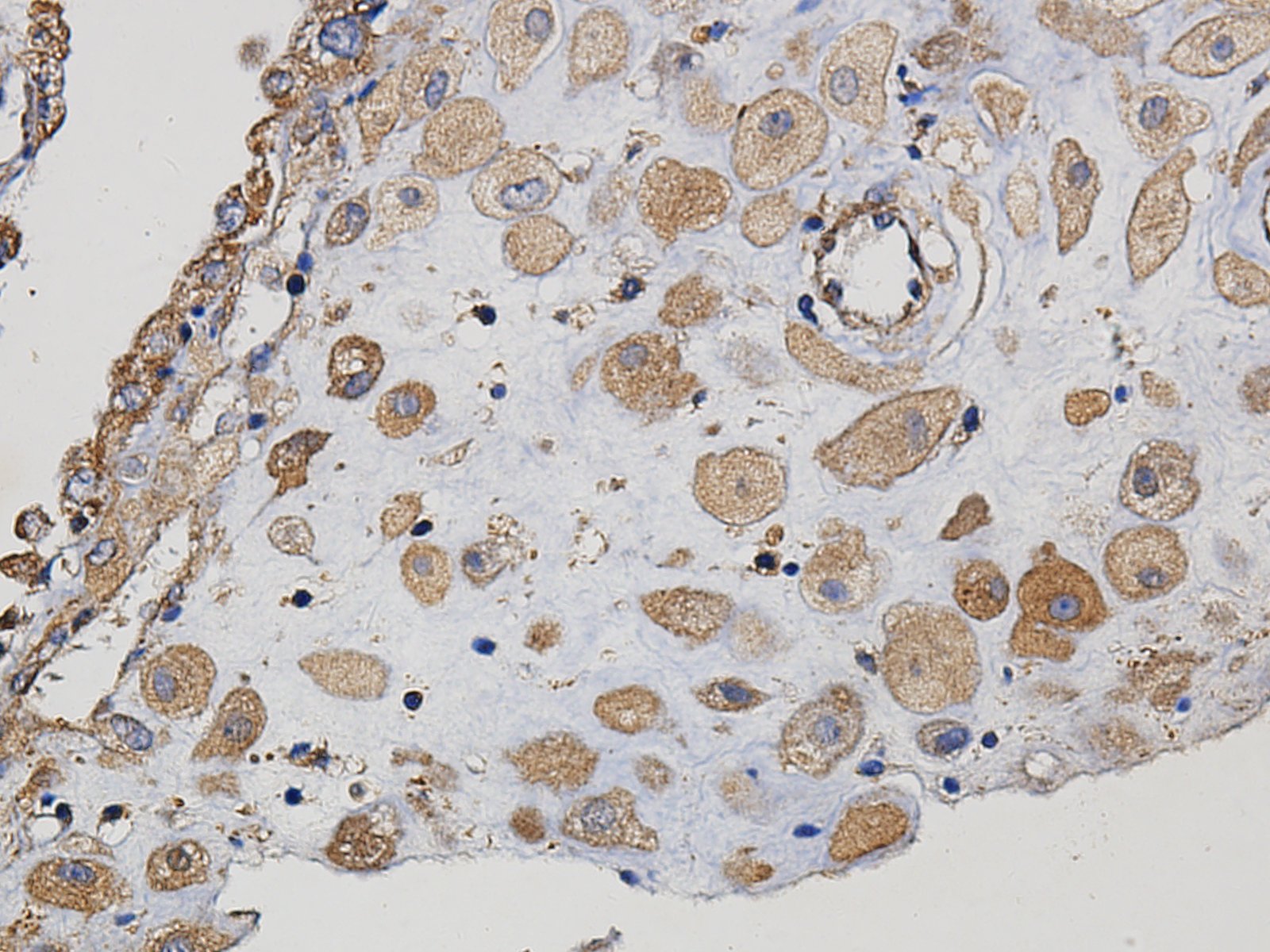

Supplement: Supplementary file 4 — Source data Fig. 2 [file 44318_2024_220_MOESM4_ESM.zip › Figure2/2A/RSA-TP53-400-5.jpg]

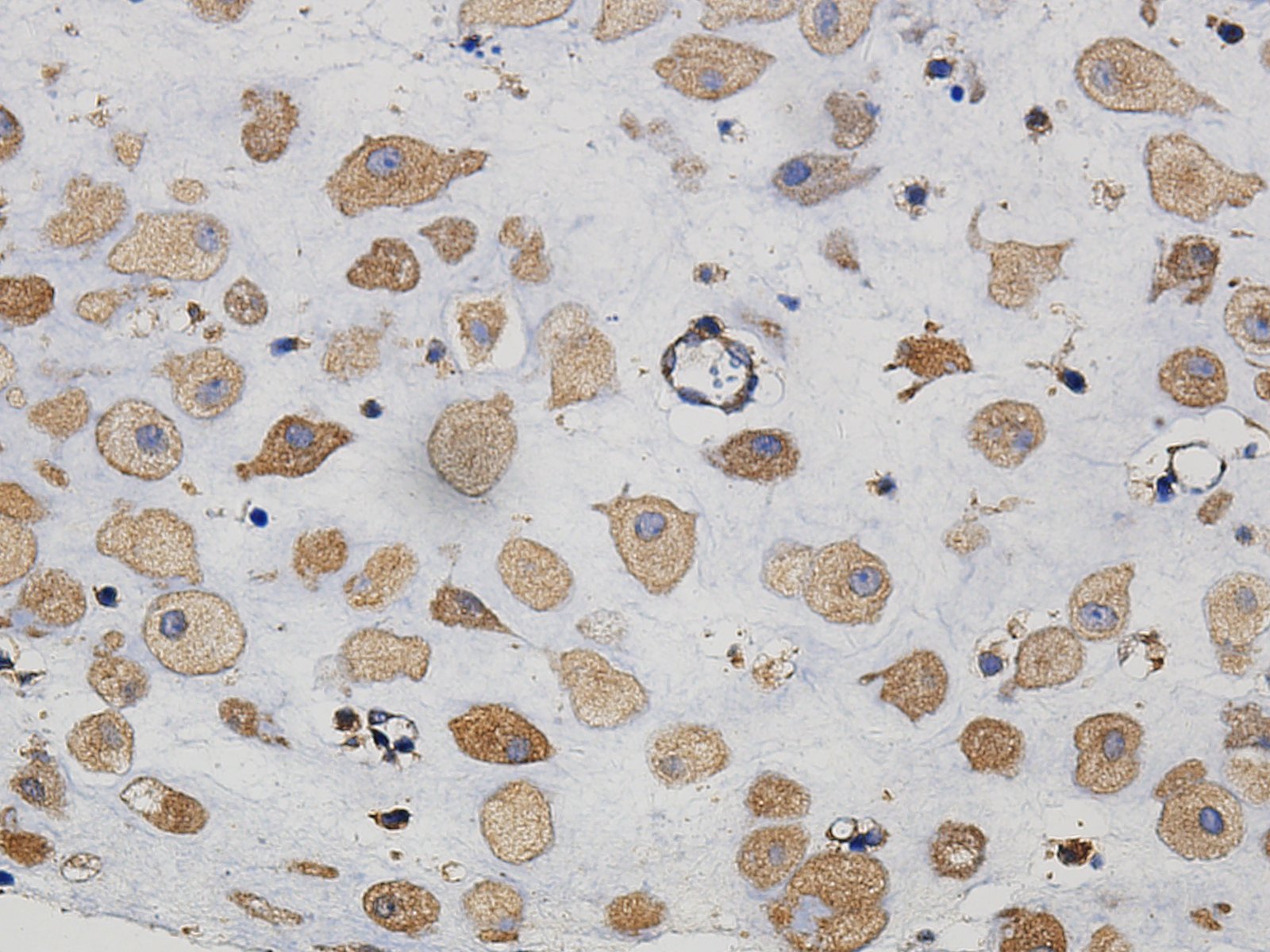

Supplement: Supplementary file 4 — Source data Fig. 2 [file 44318_2024_220_MOESM4_ESM.zip › Figure2/2A/RSA-TP53-400-6.jpg]

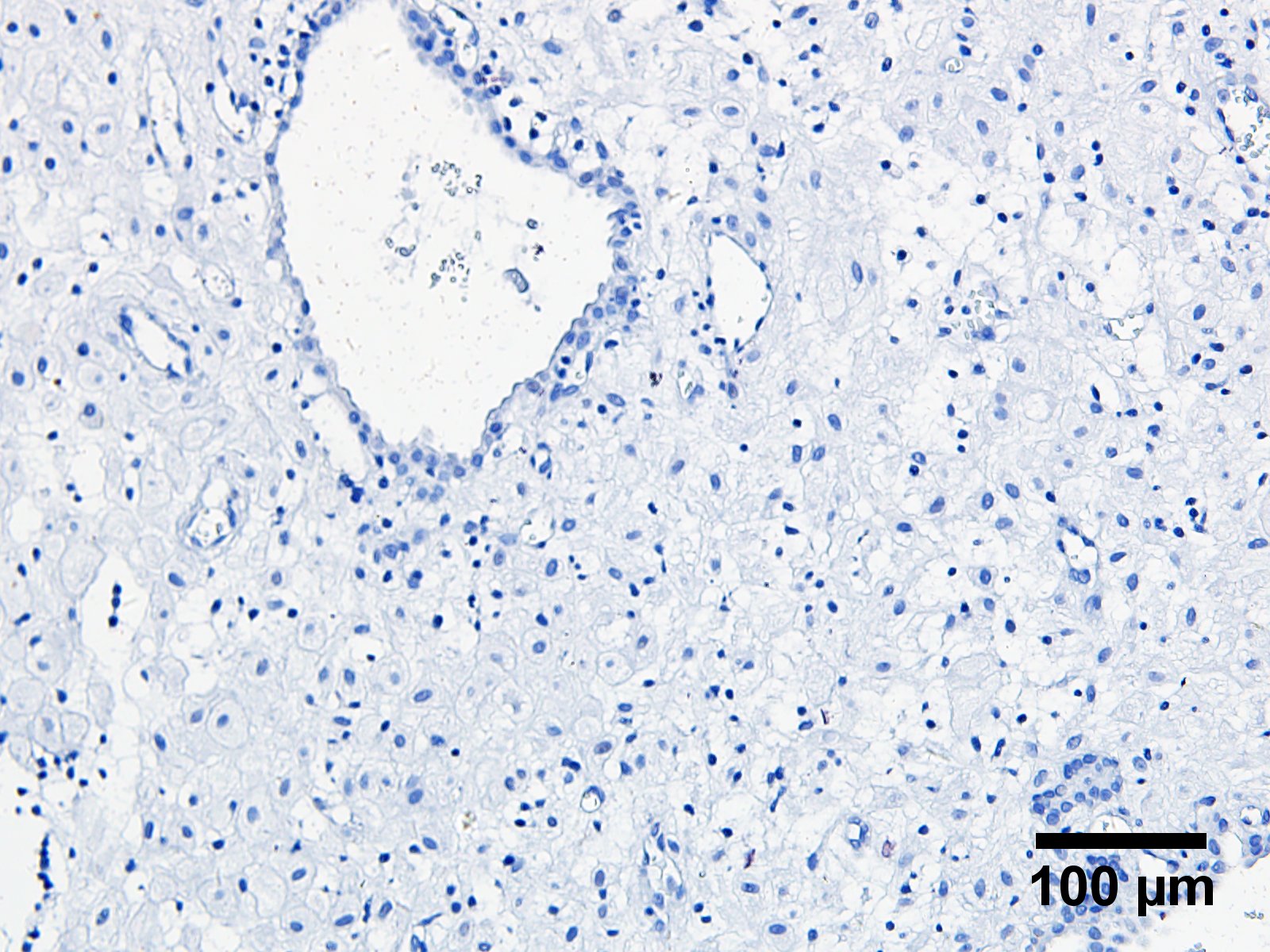

Supplement: Supplementary file 4 — Source data Fig. 2 [file 44318_2024_220_MOESM4_ESM.zip › Figure2/2A/TP53-BLANK-200.jpg]

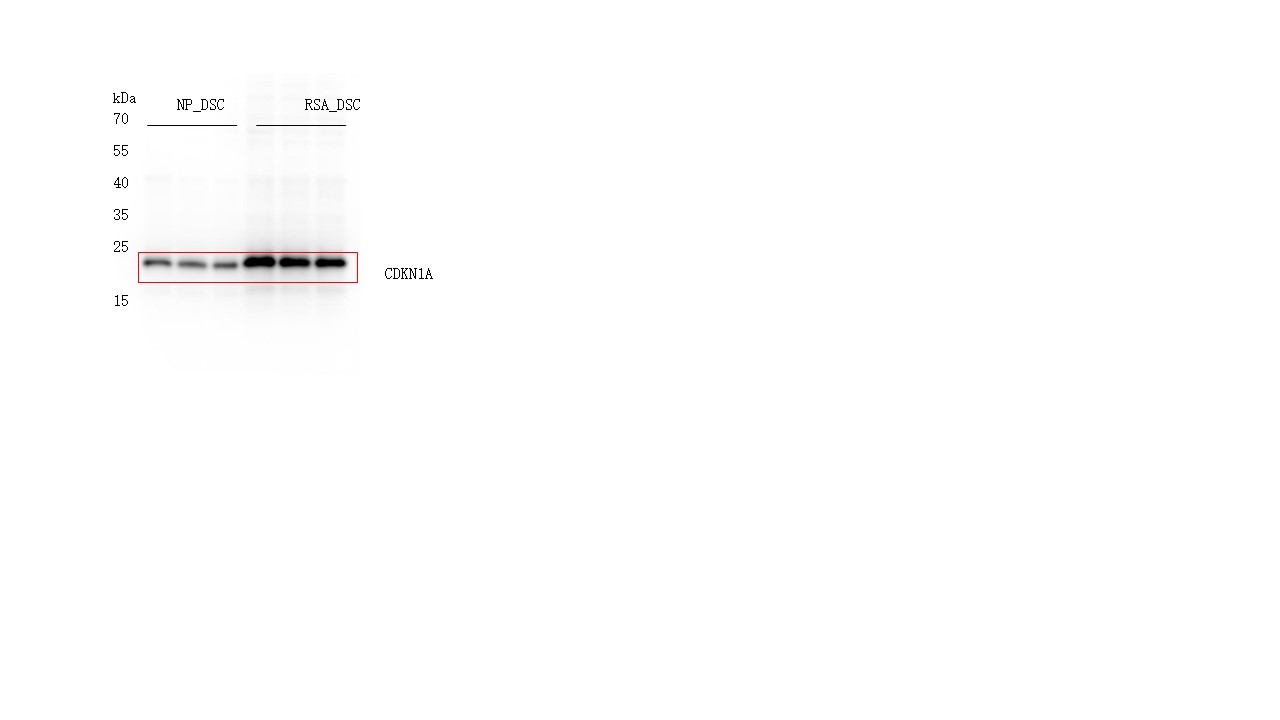

Supplement: Supplementary file 4 — Source data Fig. 2 [file 44318_2024_220_MOESM4_ESM.zip › Figure2/2B/western CDKN1A.jpg]

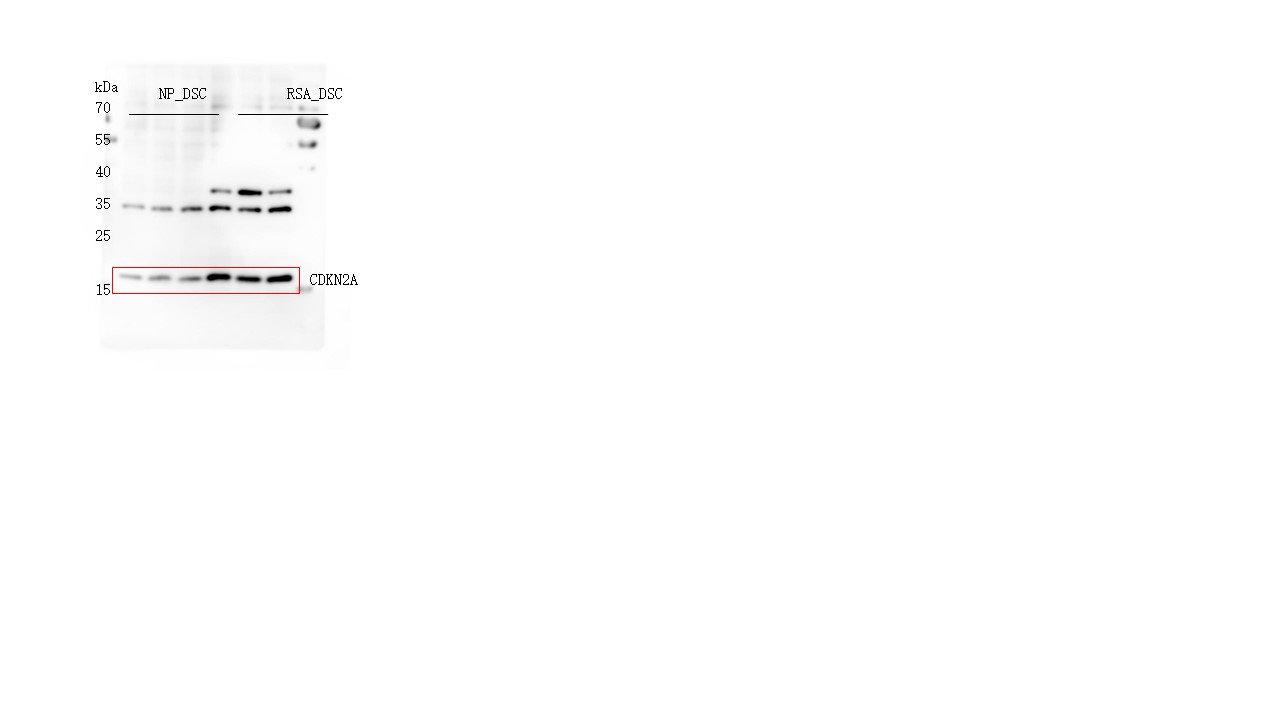

Supplement: Supplementary file 4 — Source data Fig. 2 [file 44318_2024_220_MOESM4_ESM.zip › Figure2/2B/western CDKN2A.jpg]

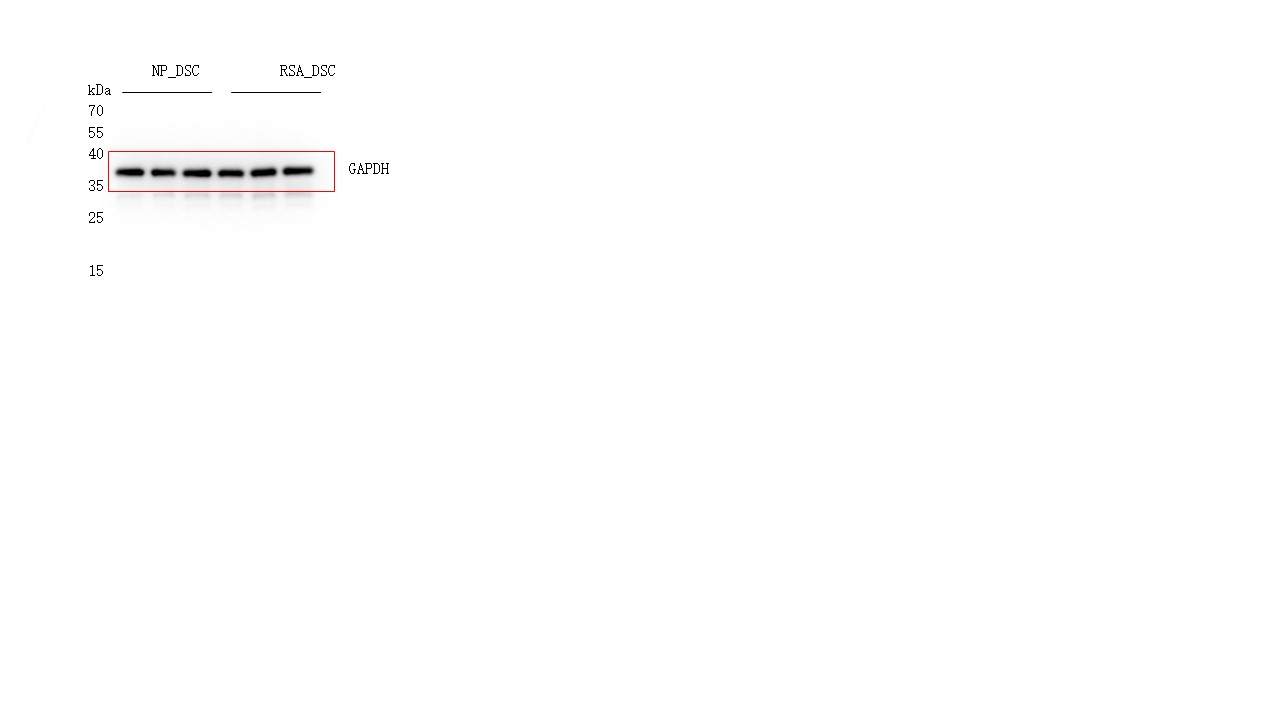

Supplement: Supplementary file 4 — Source data Fig. 2 [file 44318_2024_220_MOESM4_ESM.zip › Figure2/2B/western GAPDH.jpg]

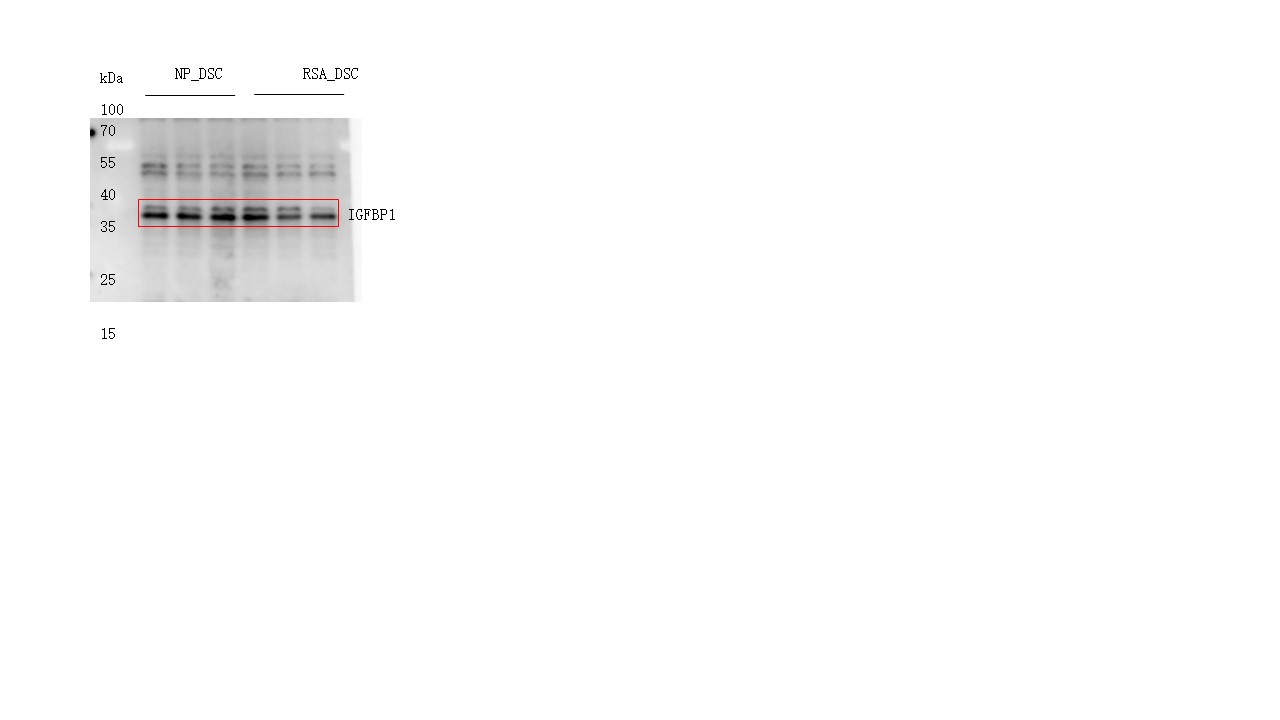

Supplement: Supplementary file 4 — Source data Fig. 2 [file 44318_2024_220_MOESM4_ESM.zip › Figure2/2B/western IGFBP1.jpg]

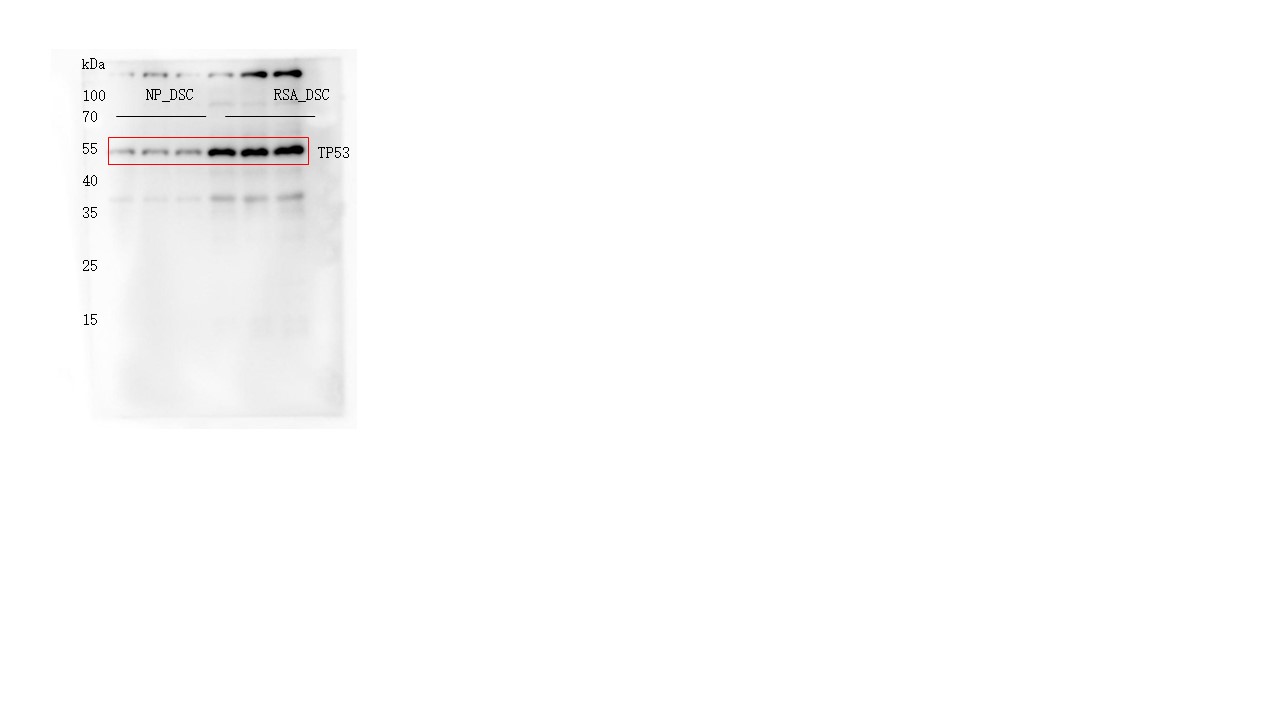

Supplement: Supplementary file 4 — Source data Fig. 2 [file 44318_2024_220_MOESM4_ESM.zip › Figure2/2B/western TP53.jpg]

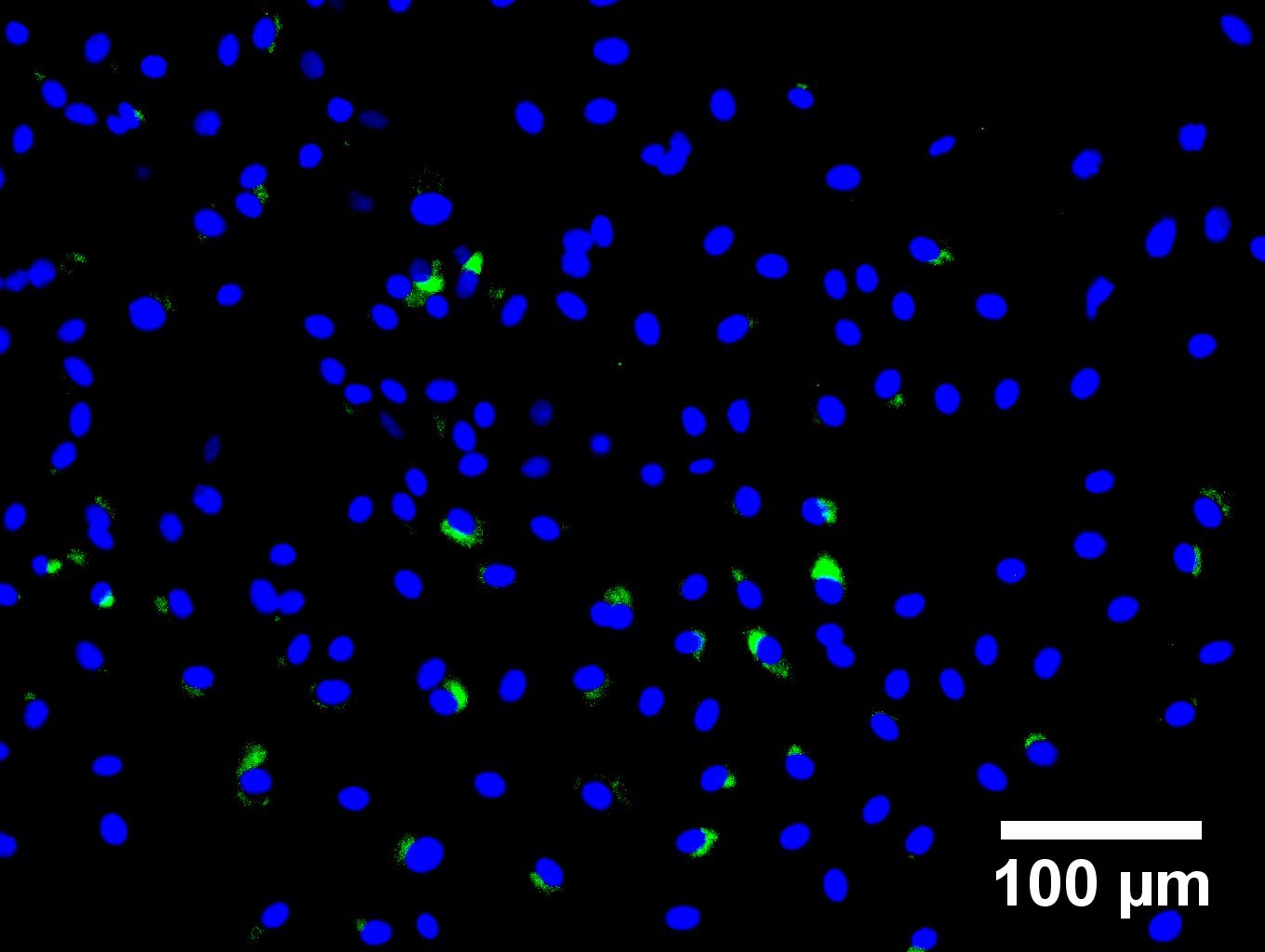

Supplement: Supplementary file 4 — Source data Fig. 2 [file 44318_2024_220_MOESM4_ESM.zip › Figure2/2C/NP1 (1).jpg]

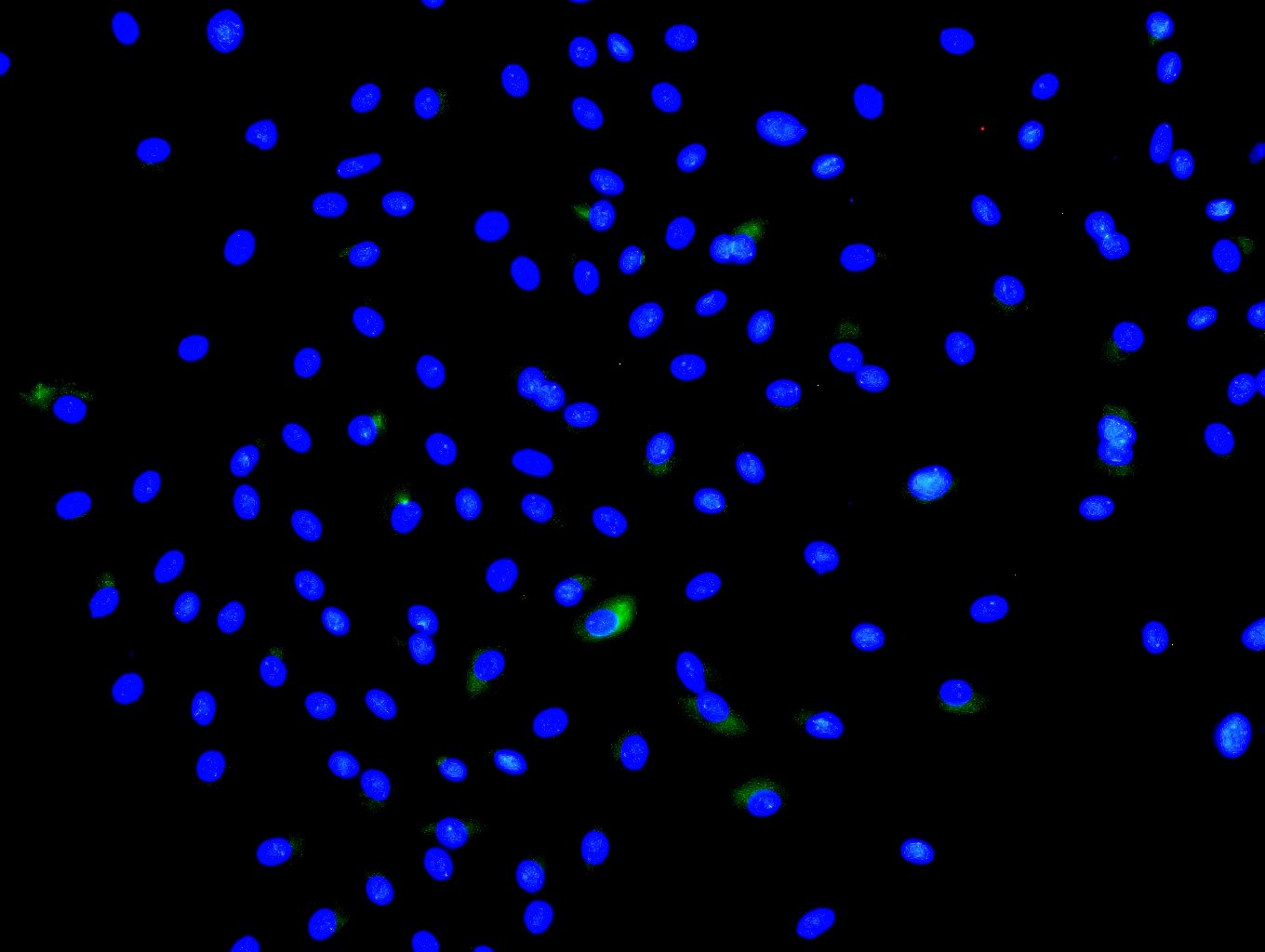

Supplement: Supplementary file 4 — Source data Fig. 2 [file 44318_2024_220_MOESM4_ESM.zip › Figure2/2C/NP1 (2).jpg]

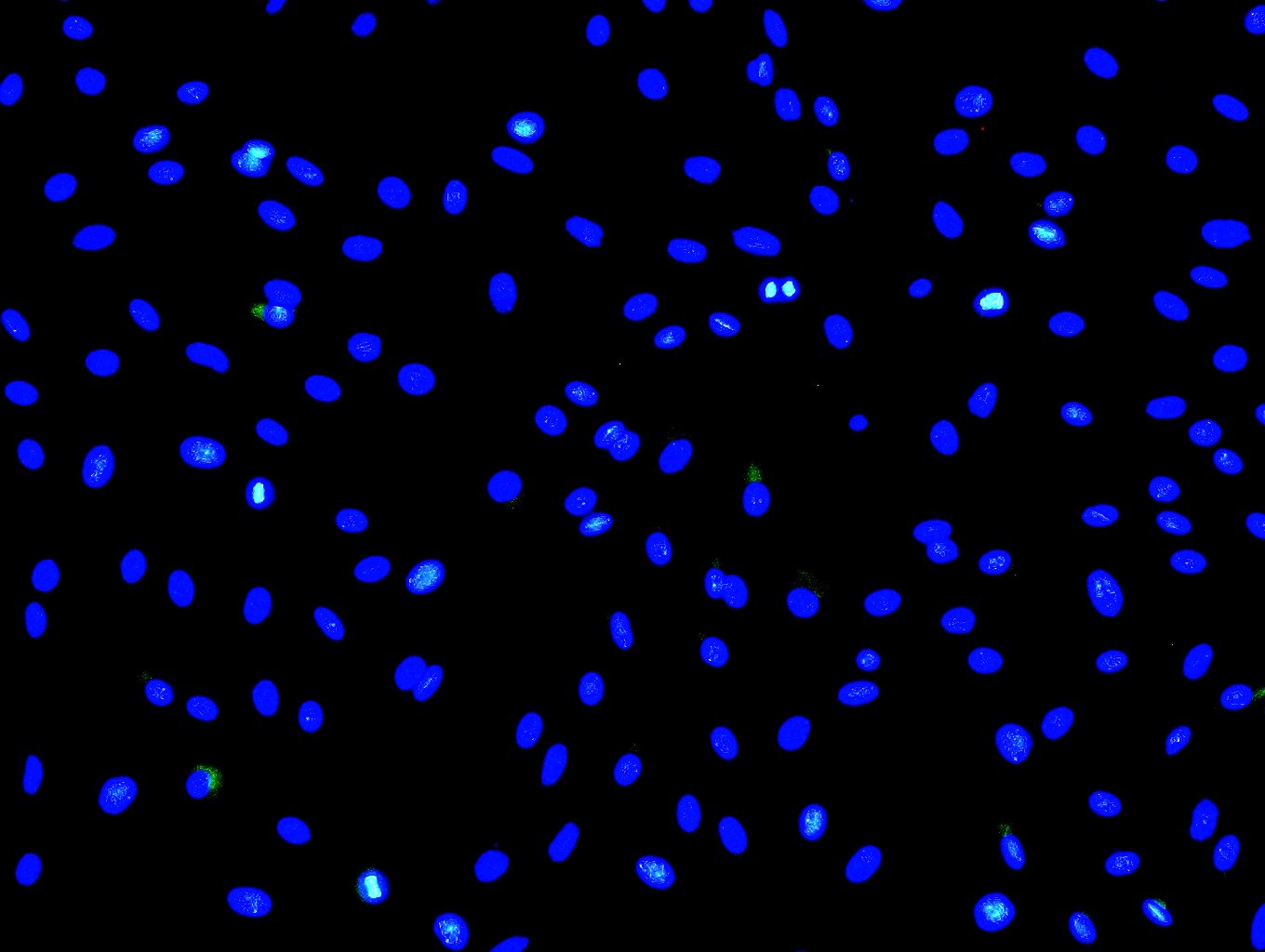

Supplement: Supplementary file 4 — Source data Fig. 2 [file 44318_2024_220_MOESM4_ESM.zip › Figure2/2C/NP1 (3).jpg]

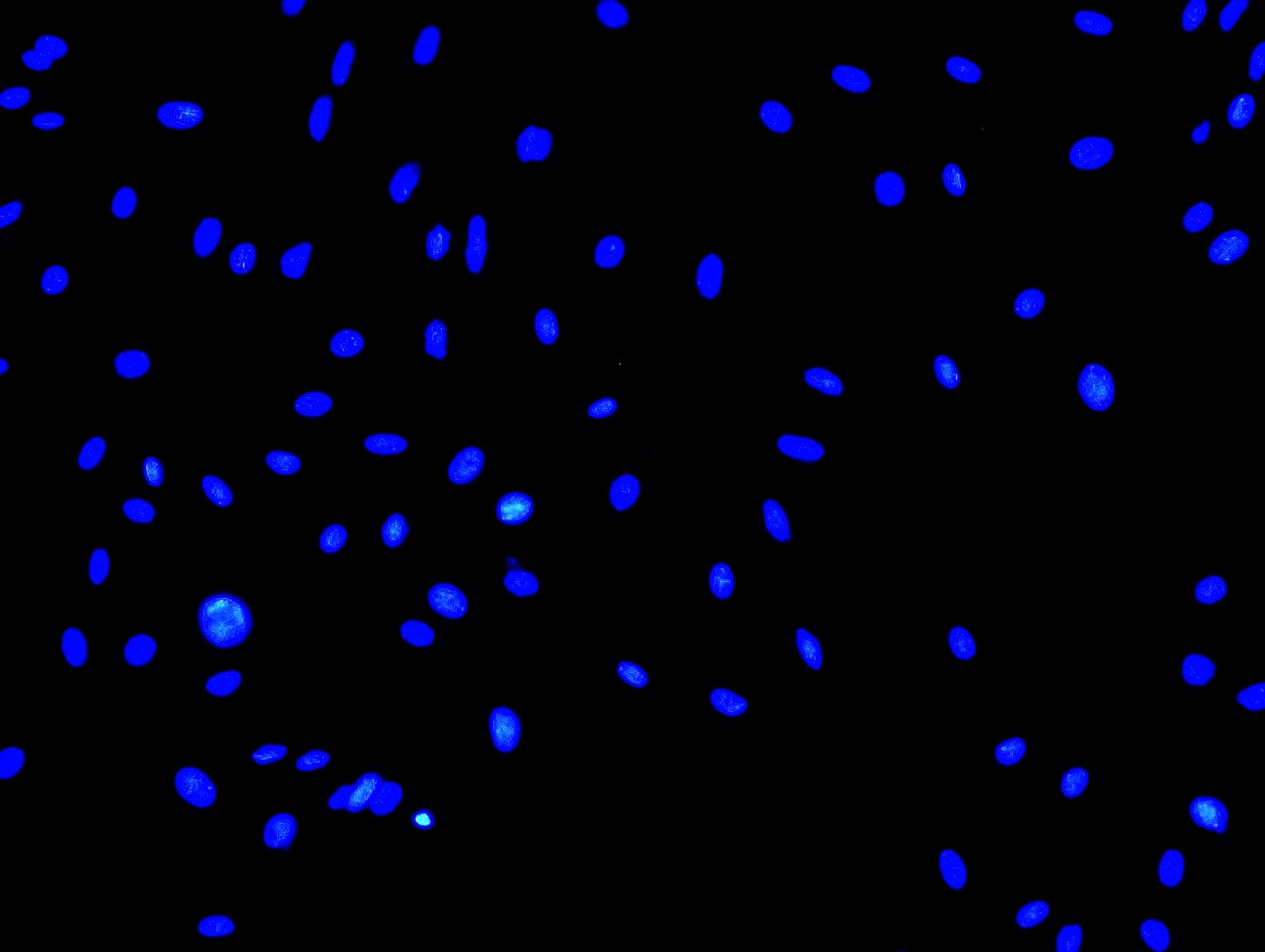

Supplement: Supplementary file 4 — Source data Fig. 2 [file 44318_2024_220_MOESM4_ESM.zip › Figure2/2C/NP2 (1).jpg]

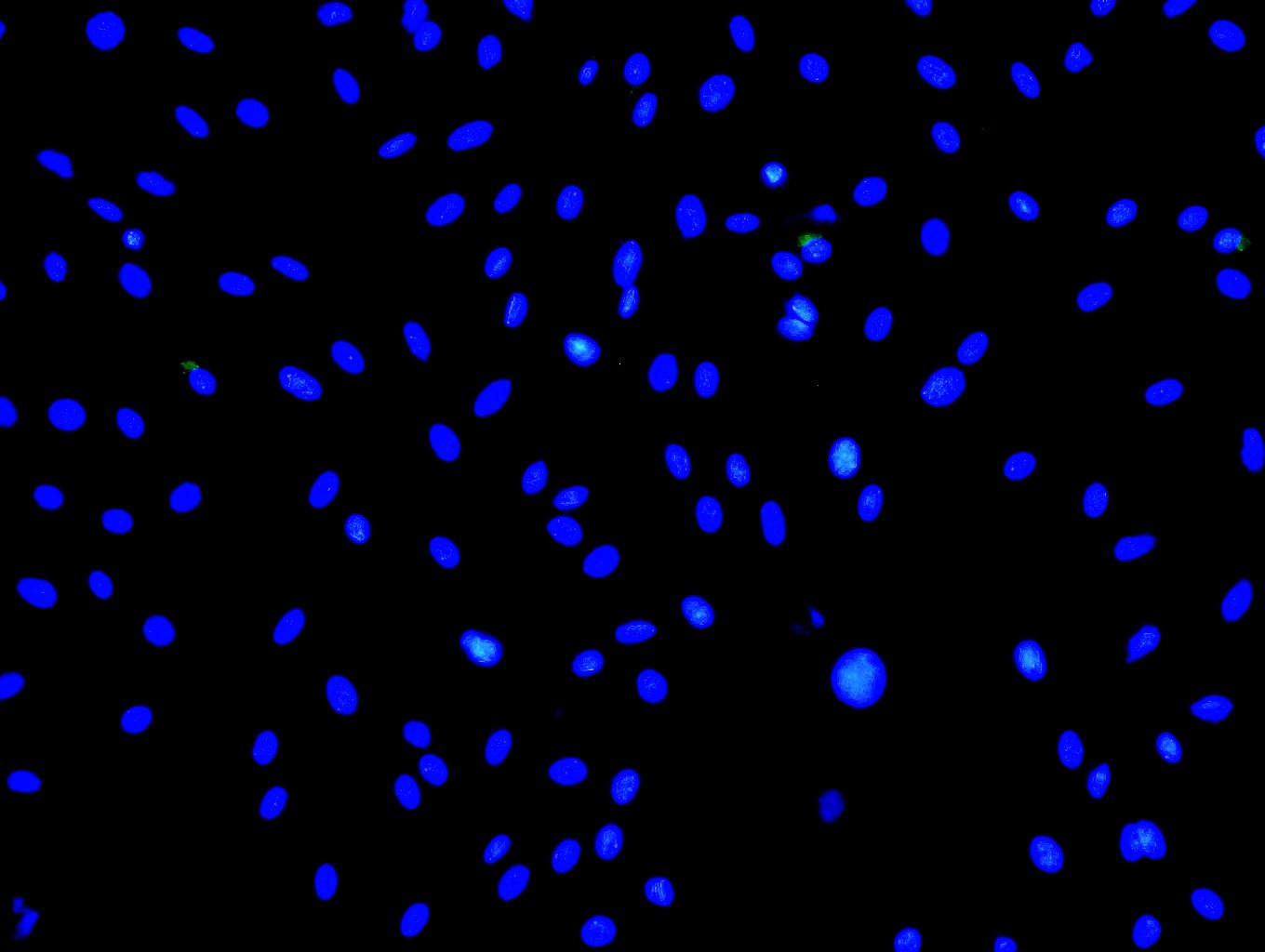

Supplement: Supplementary file 4 — Source data Fig. 2 [file 44318_2024_220_MOESM4_ESM.zip › Figure2/2C/NP2 (2).jpg]

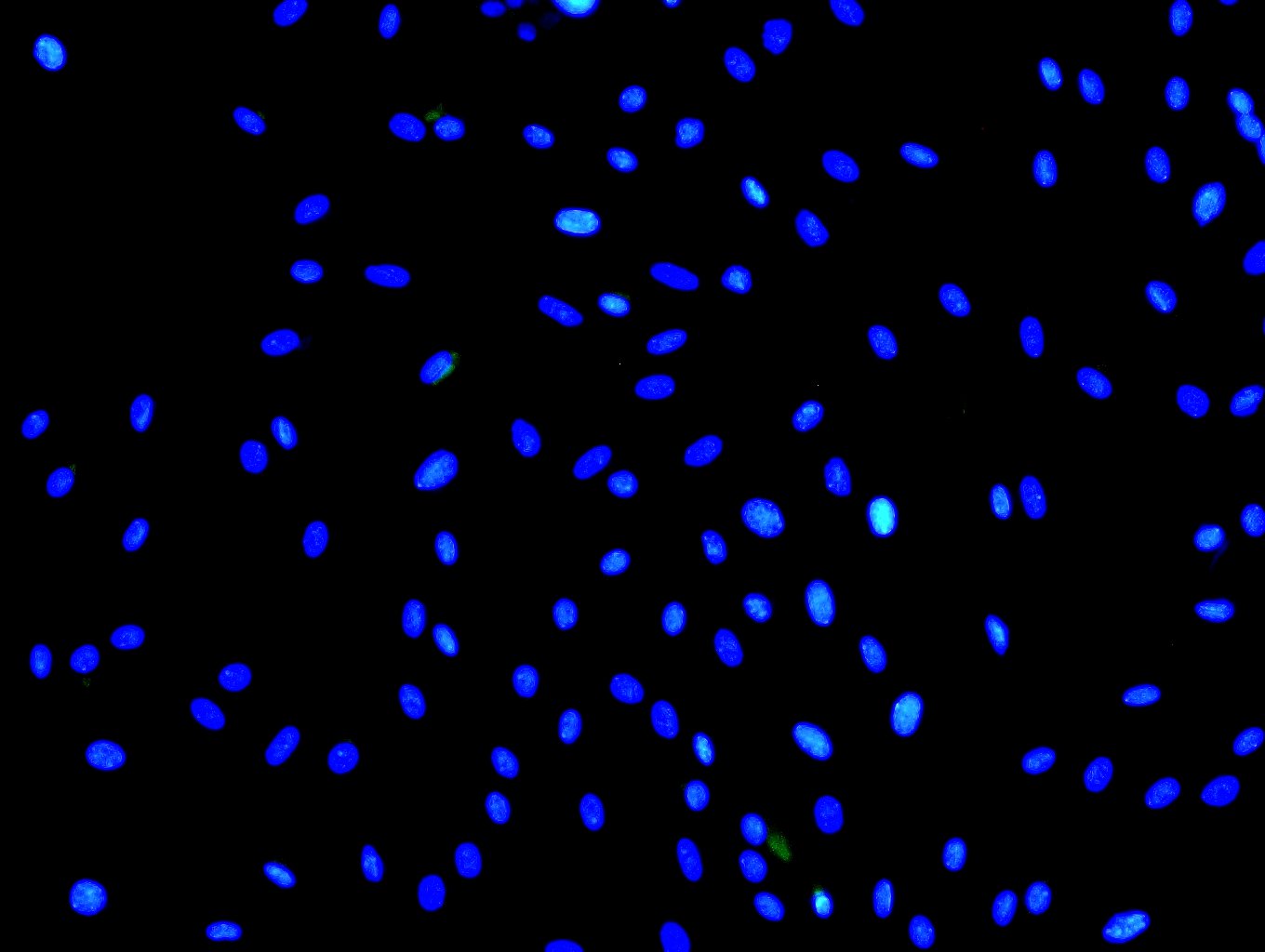

Supplement: Supplementary file 4 — Source data Fig. 2 [file 44318_2024_220_MOESM4_ESM.zip › Figure2/2C/NP2 (3).jpg]

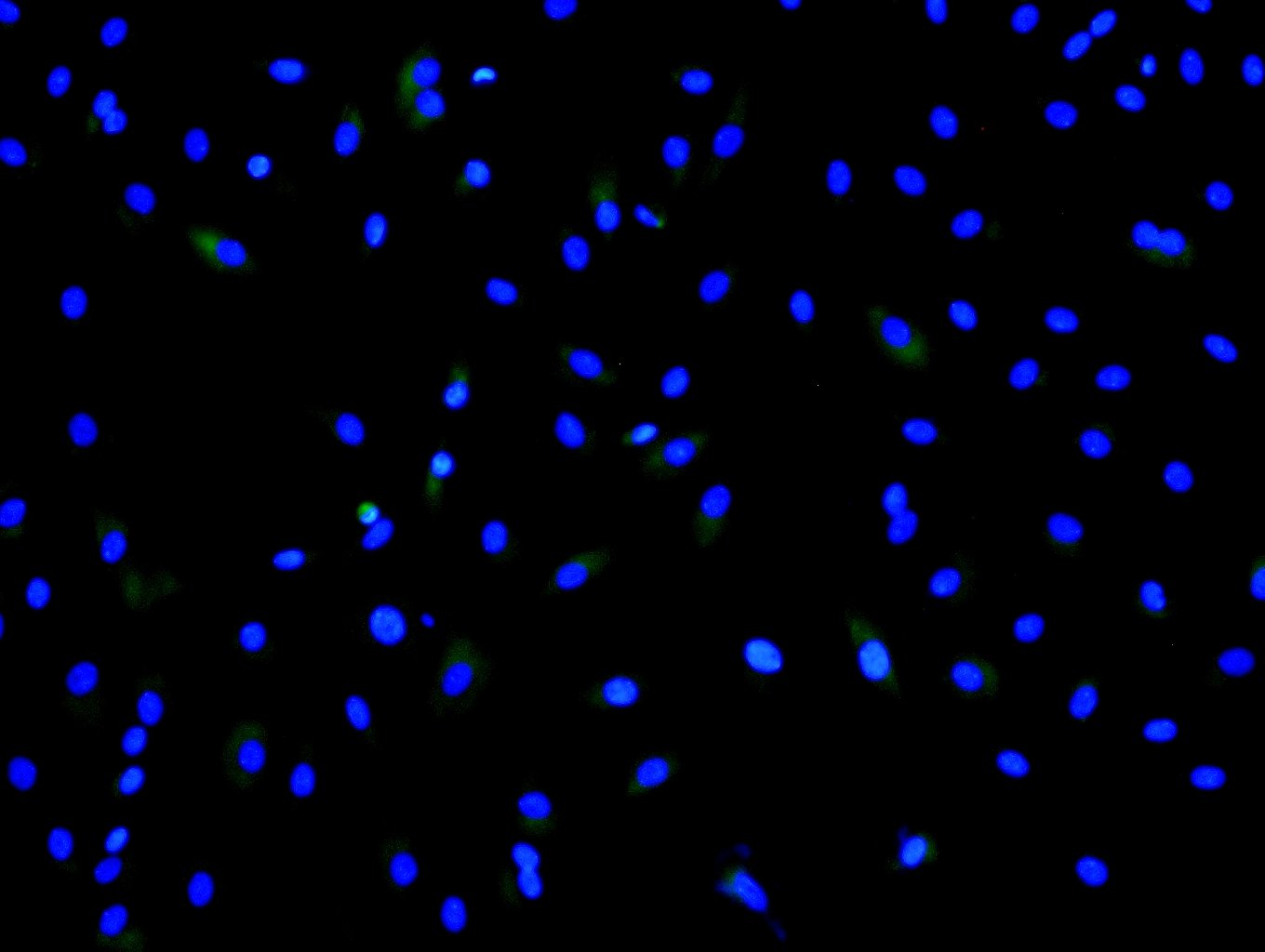

Supplement: Supplementary file 4 — Source data Fig. 2 [file 44318_2024_220_MOESM4_ESM.zip › Figure2/2C/NP3 (1).jpg]

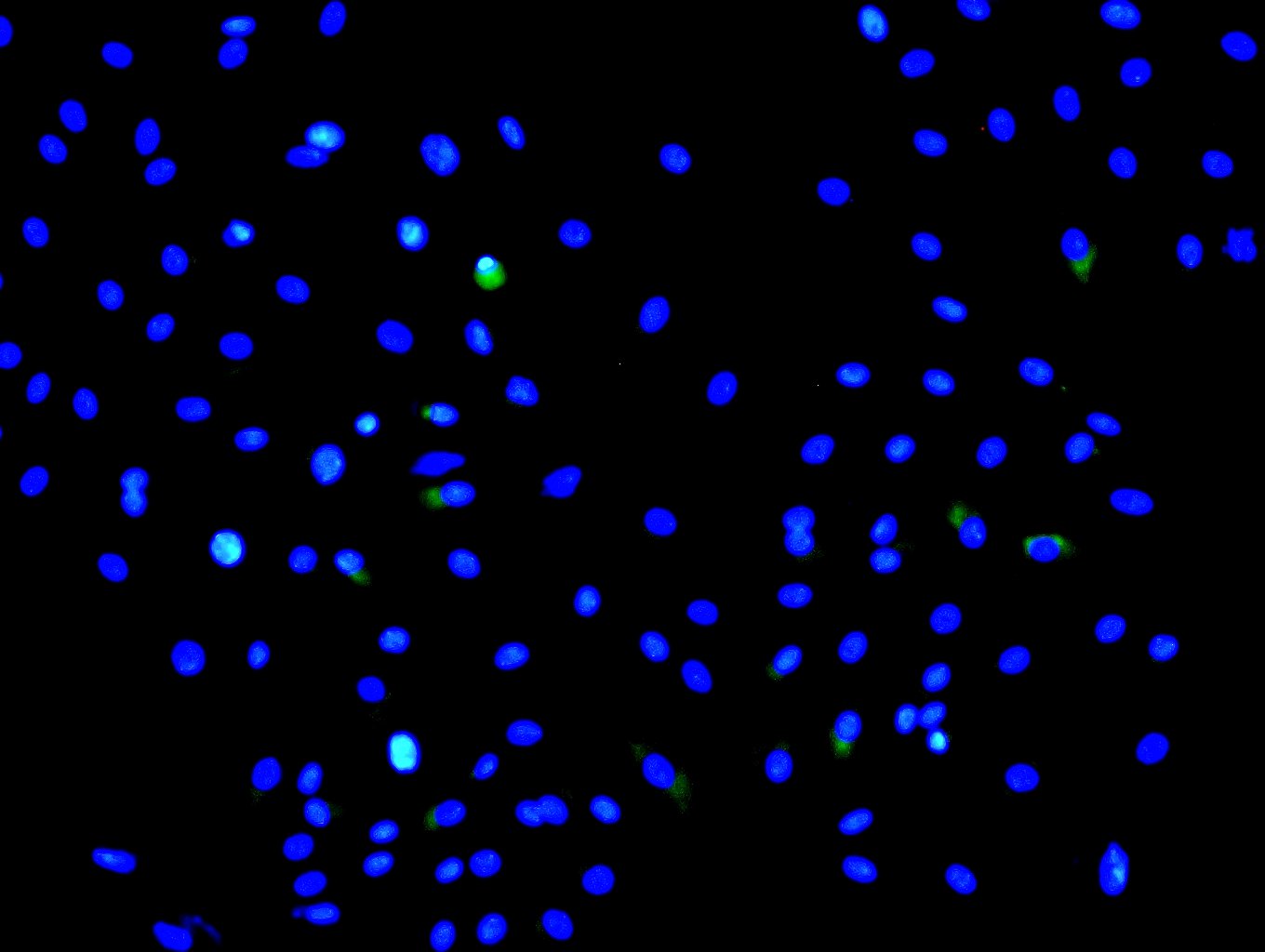

Supplement: Supplementary file 4 — Source data Fig. 2 [file 44318_2024_220_MOESM4_ESM.zip › Figure2/2C/NP3 (2).jpg]

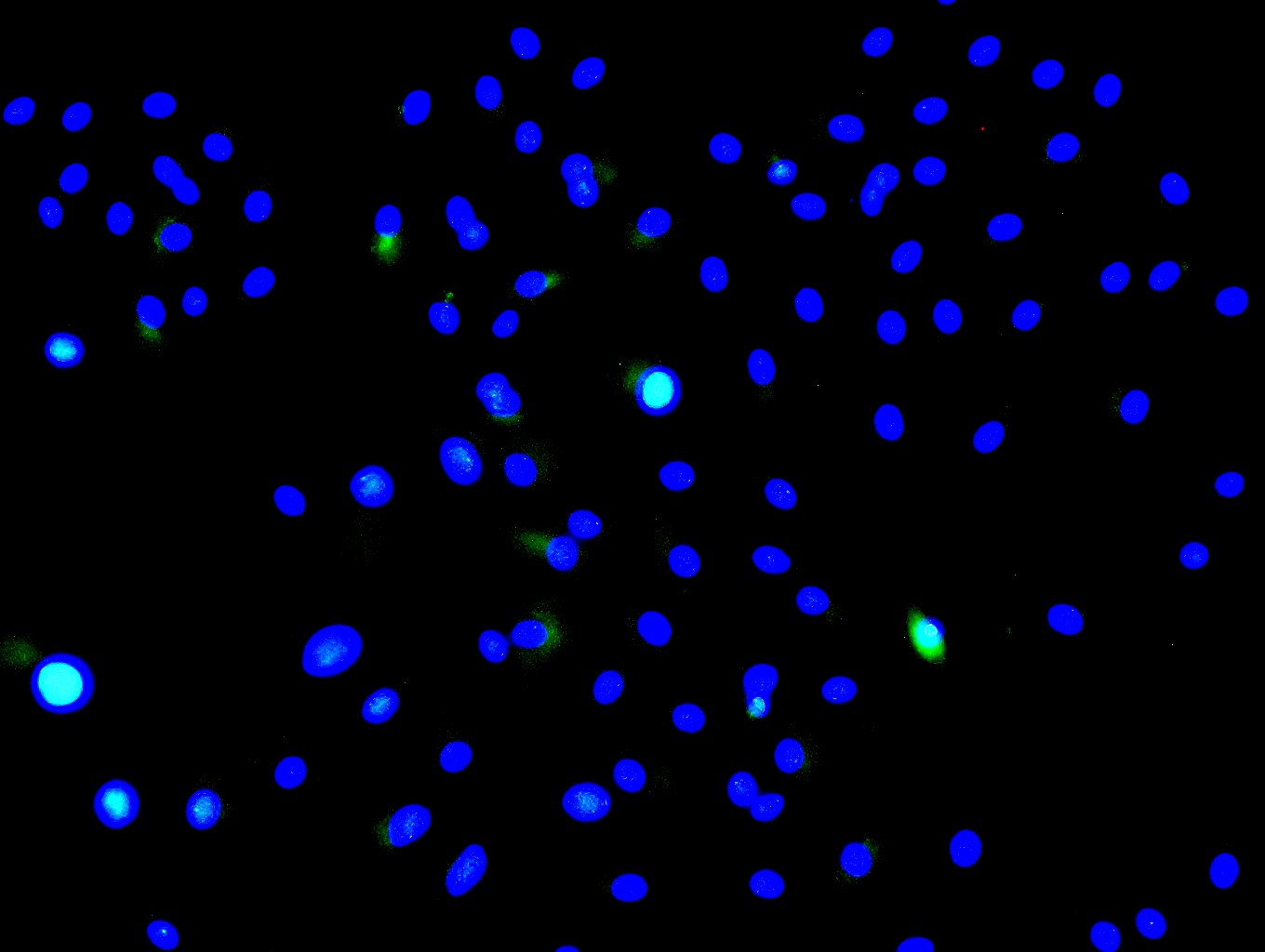

Supplement: Supplementary file 4 — Source data Fig. 2 [file 44318_2024_220_MOESM4_ESM.zip › Figure2/2C/NP3 (3).jpg]

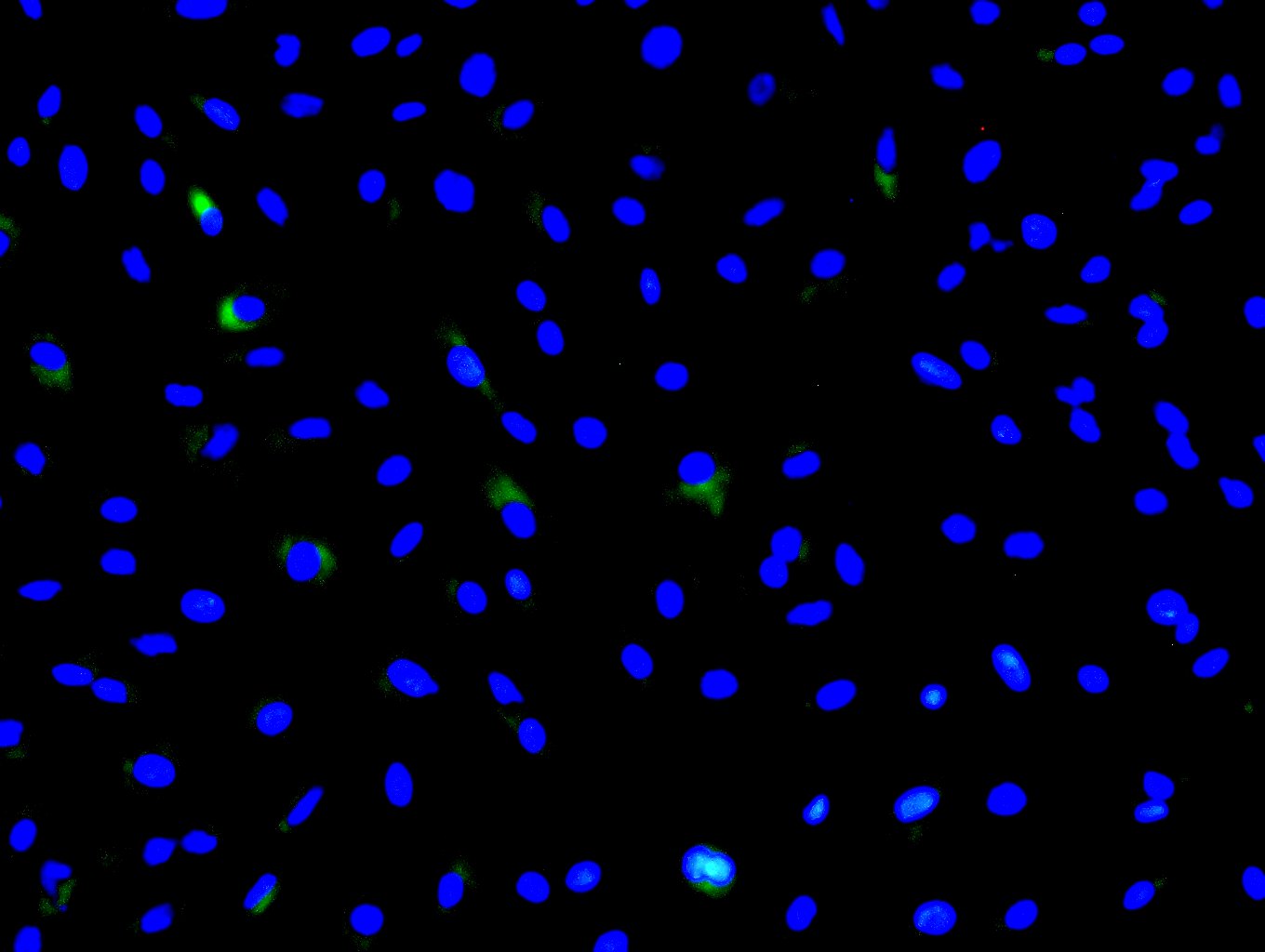

Supplement: Supplementary file 4 — Source data Fig. 2 [file 44318_2024_220_MOESM4_ESM.zip › Figure2/2C/NP4 (1).jpg]

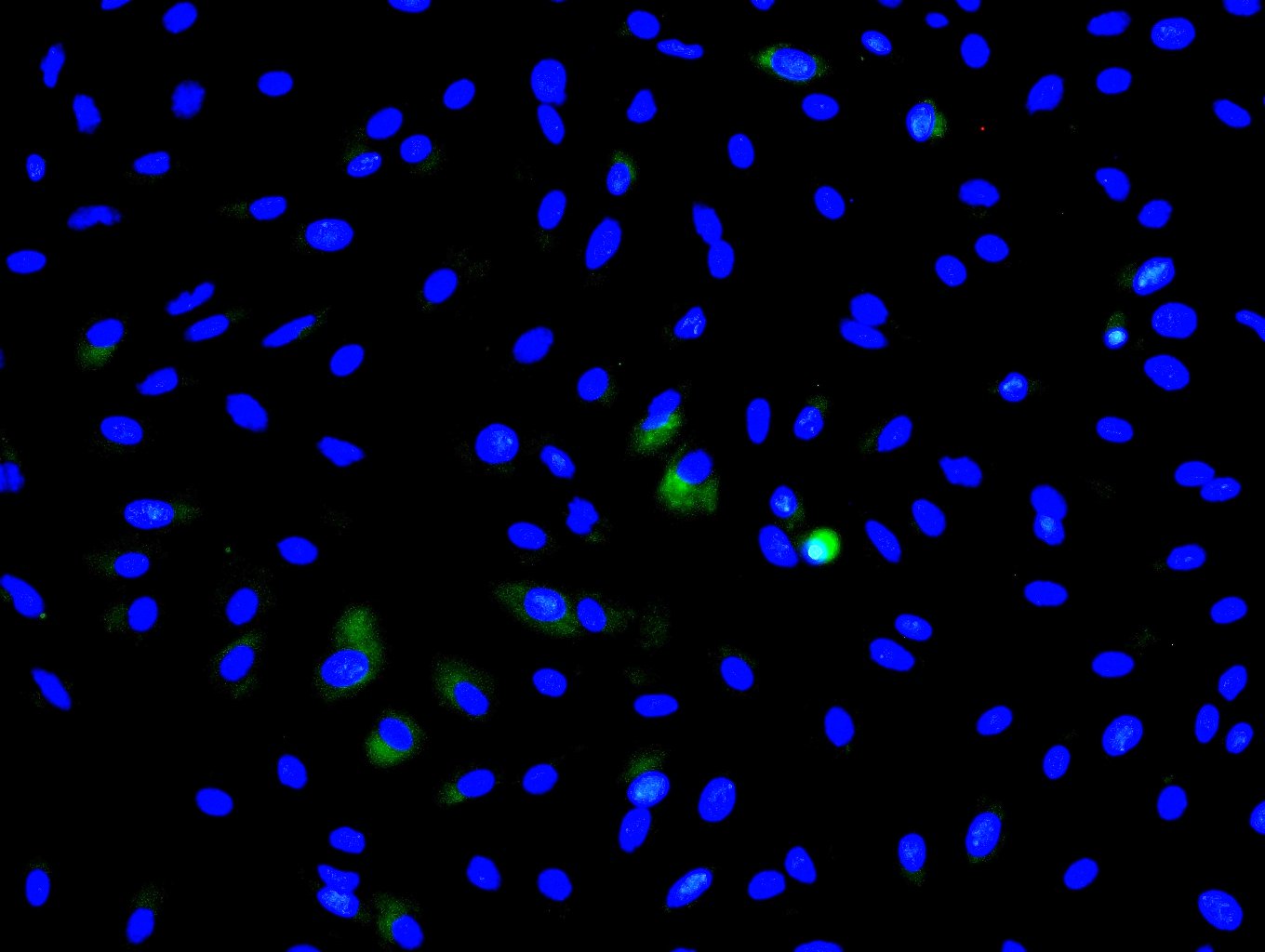

Supplement: Supplementary file 4 — Source data Fig. 2 [file 44318_2024_220_MOESM4_ESM.zip › Figure2/2C/NP4 (2).jpg]

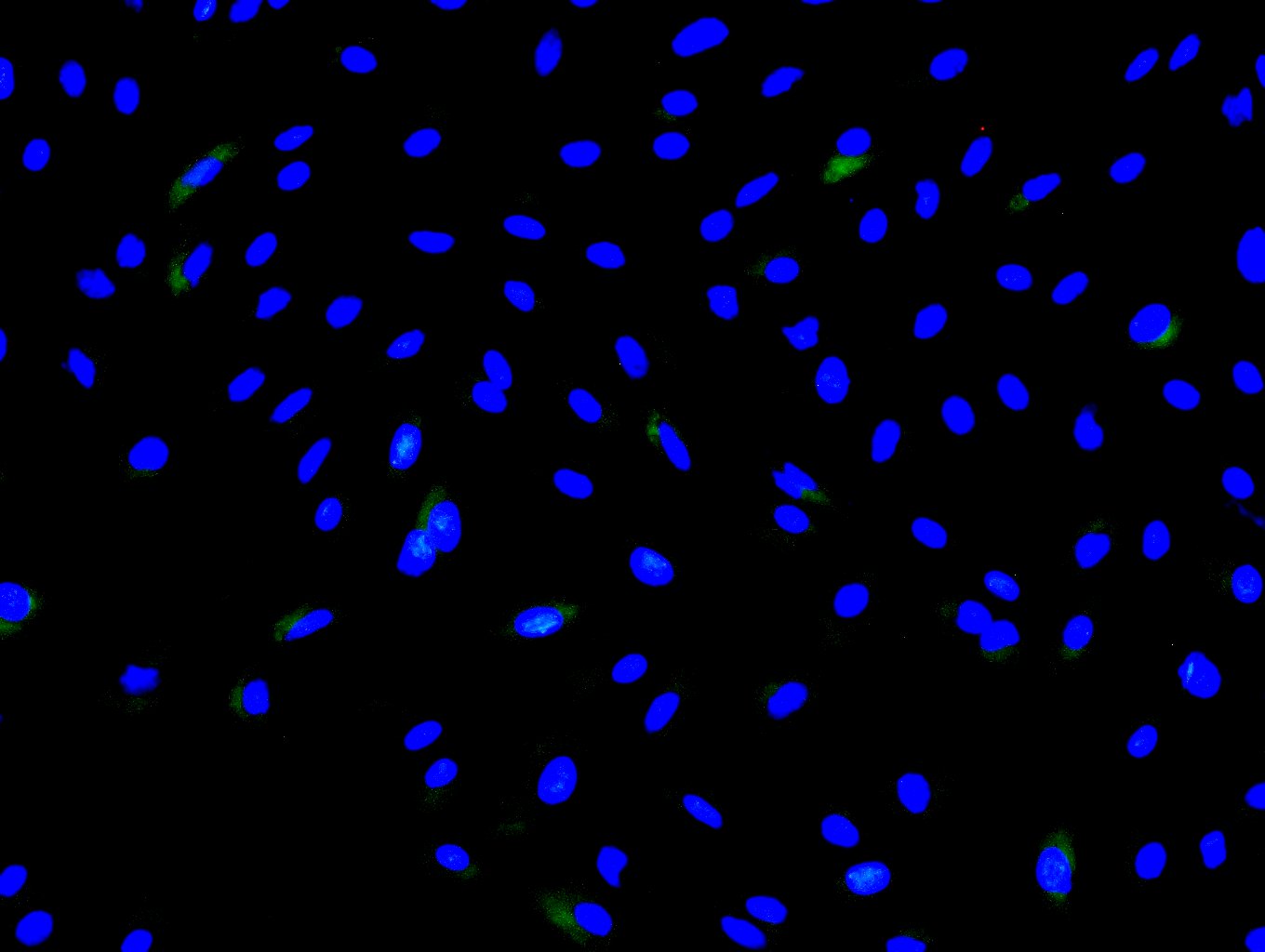

Supplement: Supplementary file 4 — Source data Fig. 2 [file 44318_2024_220_MOESM4_ESM.zip › Figure2/2C/NP4 (3).jpg]

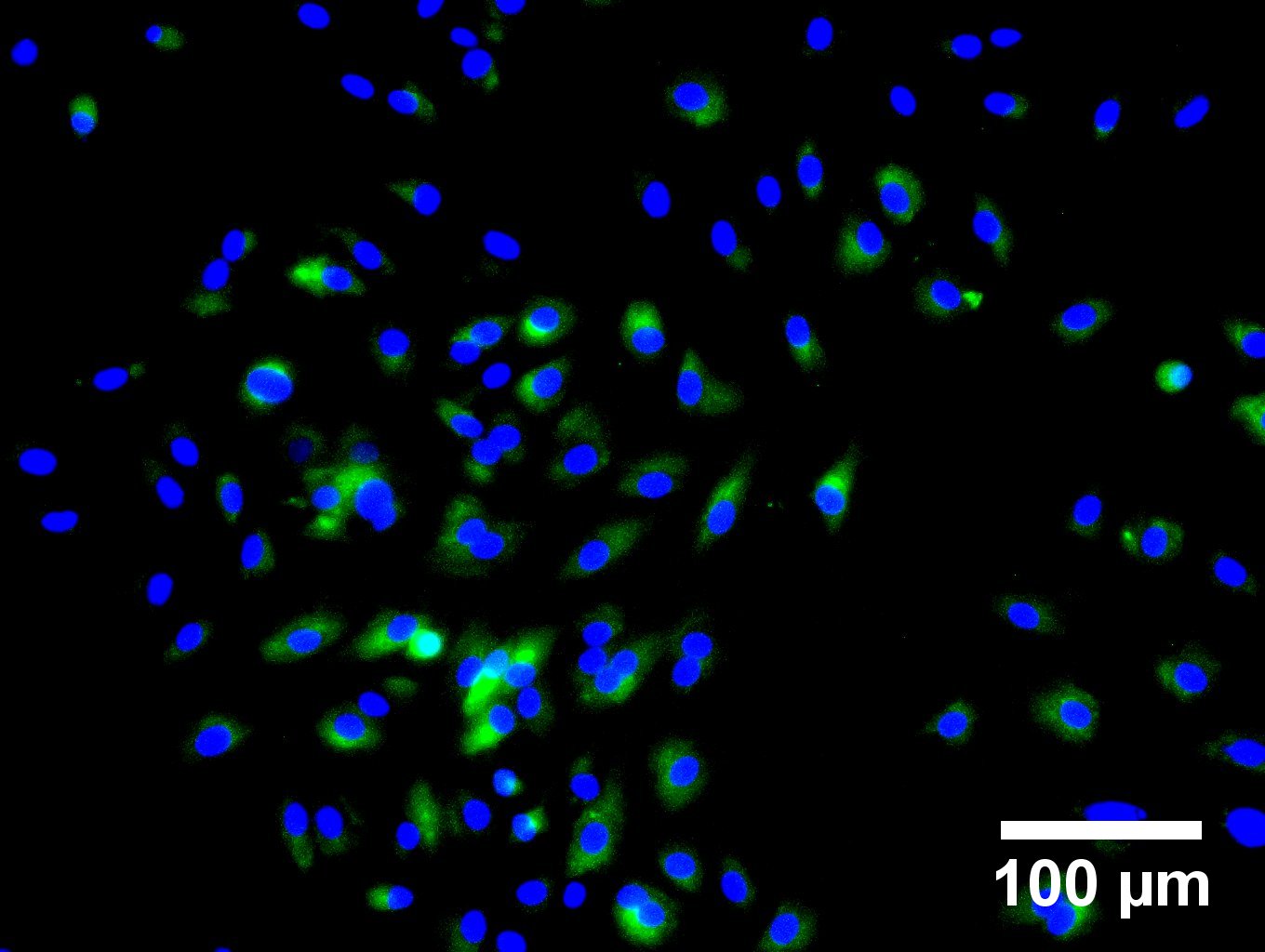

Supplement: Supplementary file 4 — Source data Fig. 2 [file 44318_2024_220_MOESM4_ESM.zip › Figure2/2C/RSA1 (1).jpg]

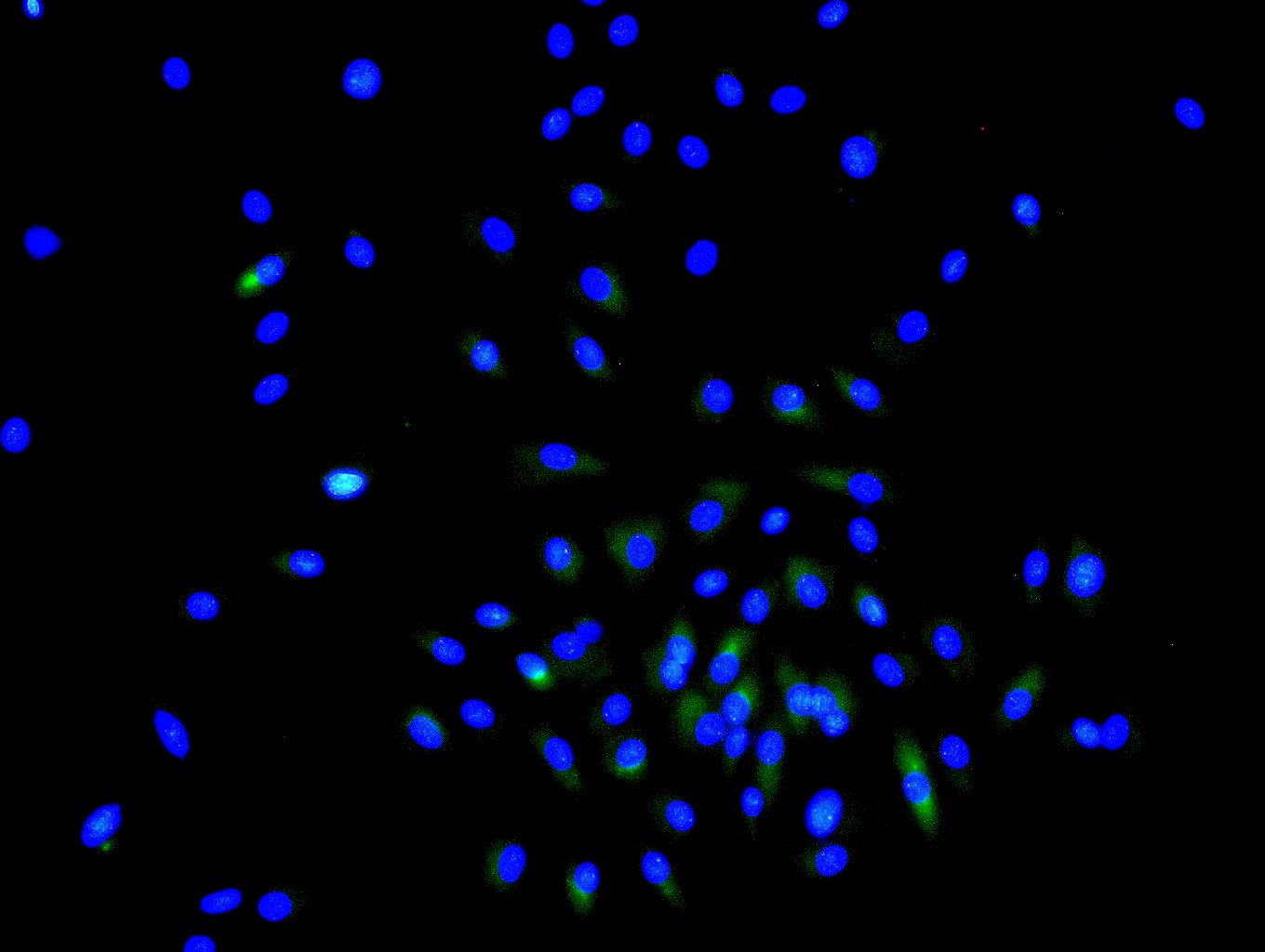

Supplement: Supplementary file 4 — Source data Fig. 2 [file 44318_2024_220_MOESM4_ESM.zip › Figure2/2C/RSA1 (2).jpg]

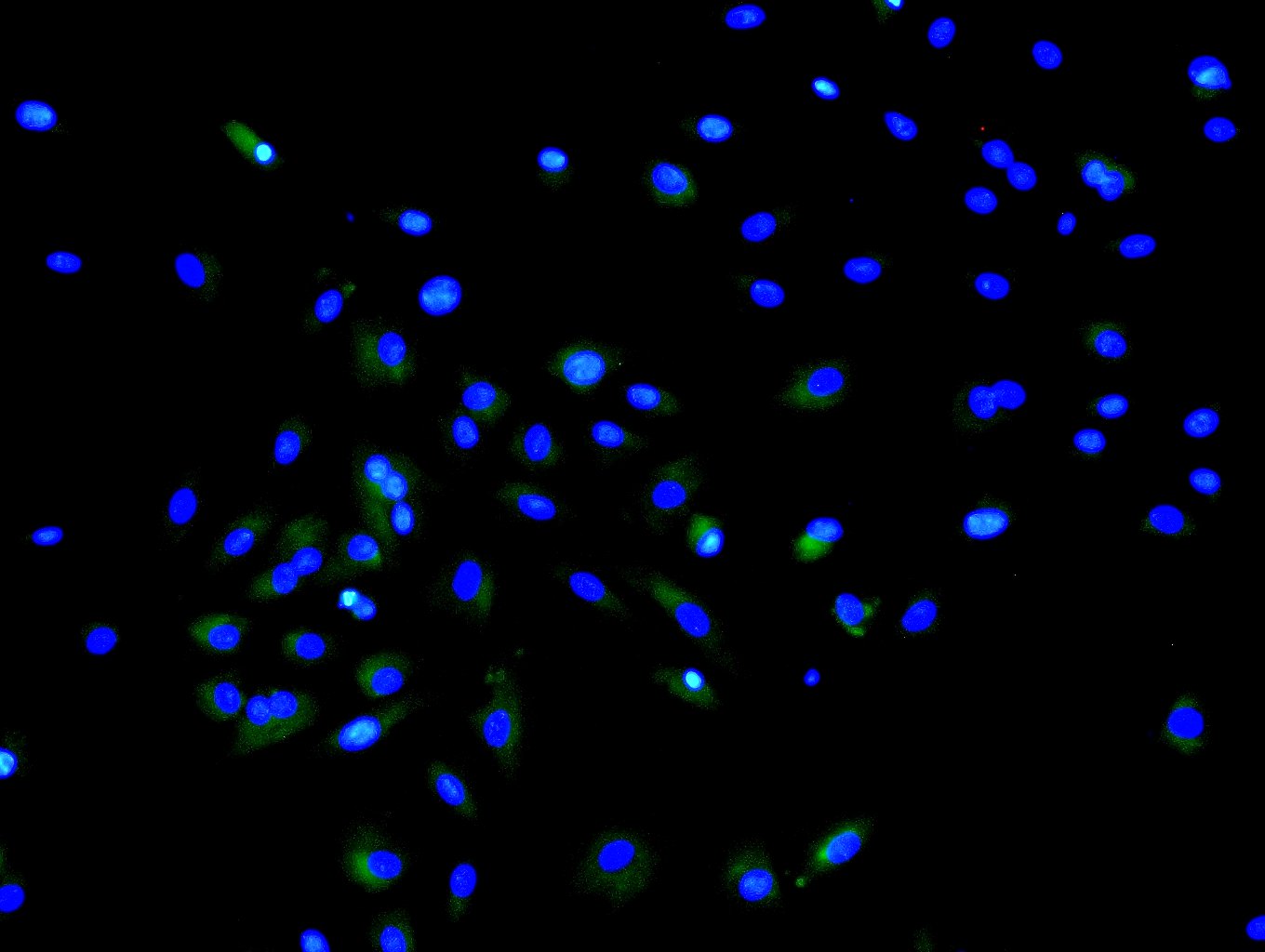

Supplement: Supplementary file 4 — Source data Fig. 2 [file 44318_2024_220_MOESM4_ESM.zip › Figure2/2C/RSA1 (3).jpg]

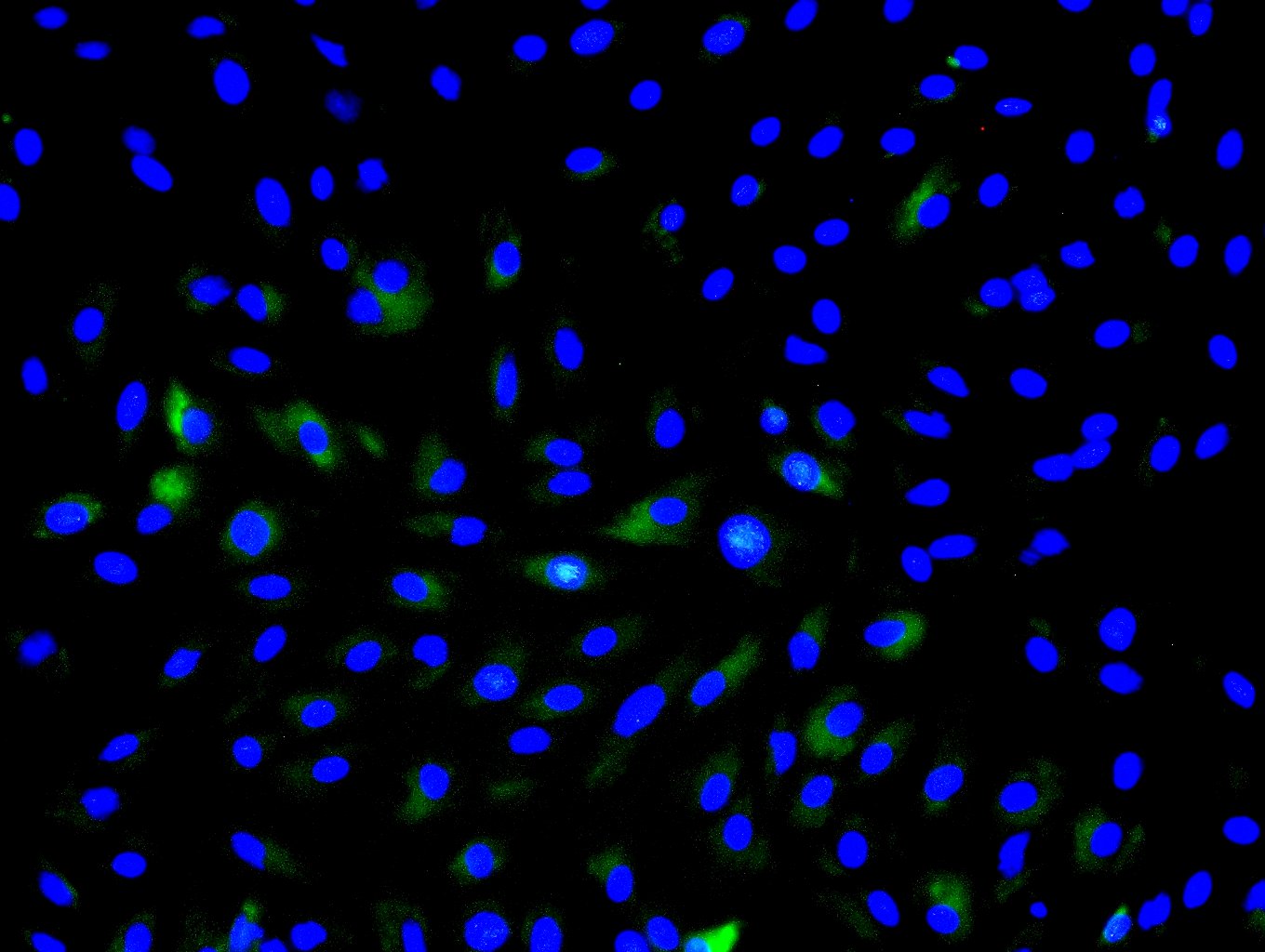

Supplement: Supplementary file 4 — Source data Fig. 2 [file 44318_2024_220_MOESM4_ESM.zip › Figure2/2C/RSA2 (1).jpg]

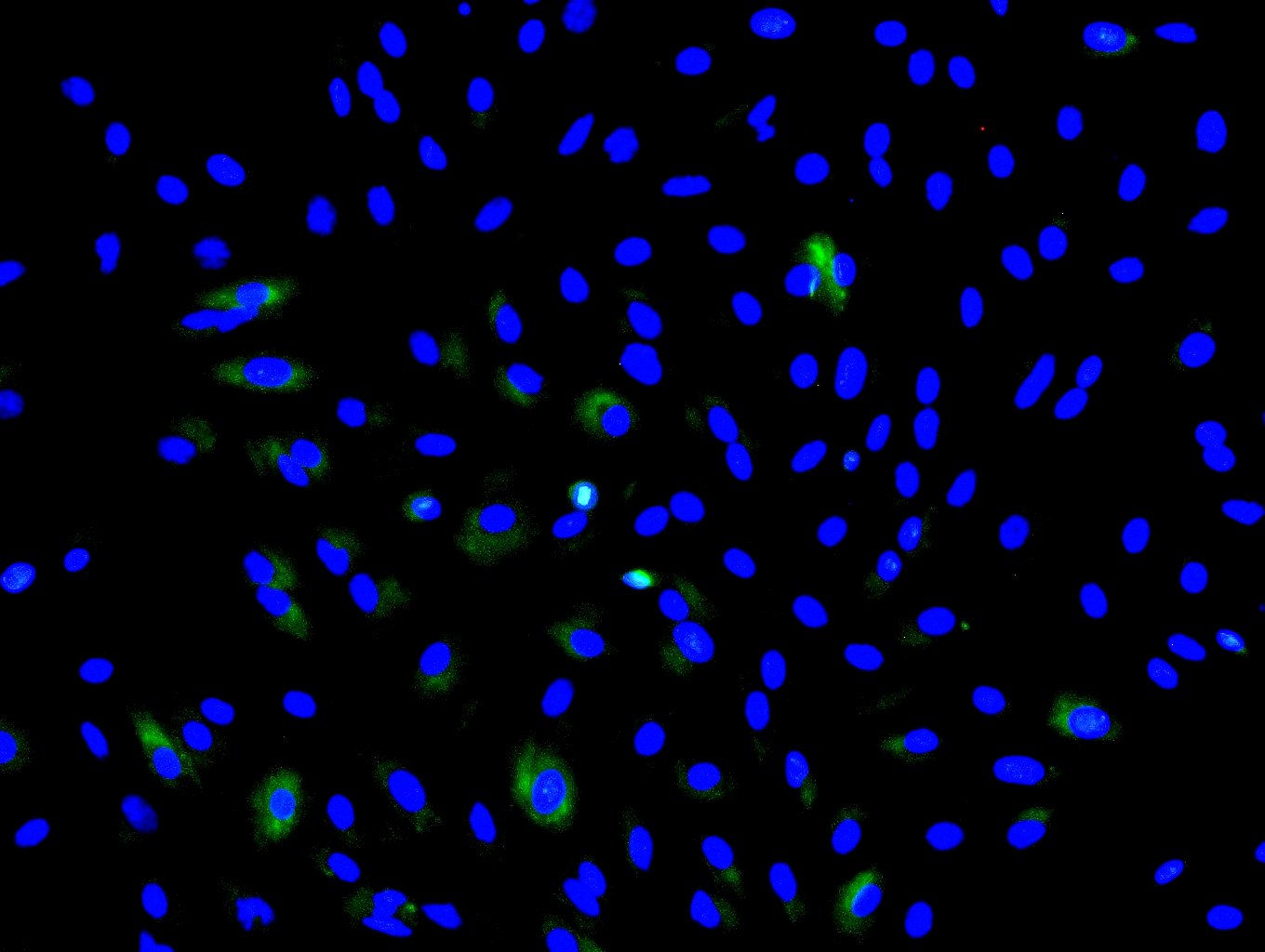

Supplement: Supplementary file 4 — Source data Fig. 2 [file 44318_2024_220_MOESM4_ESM.zip › Figure2/2C/RSA2 (2).jpg]

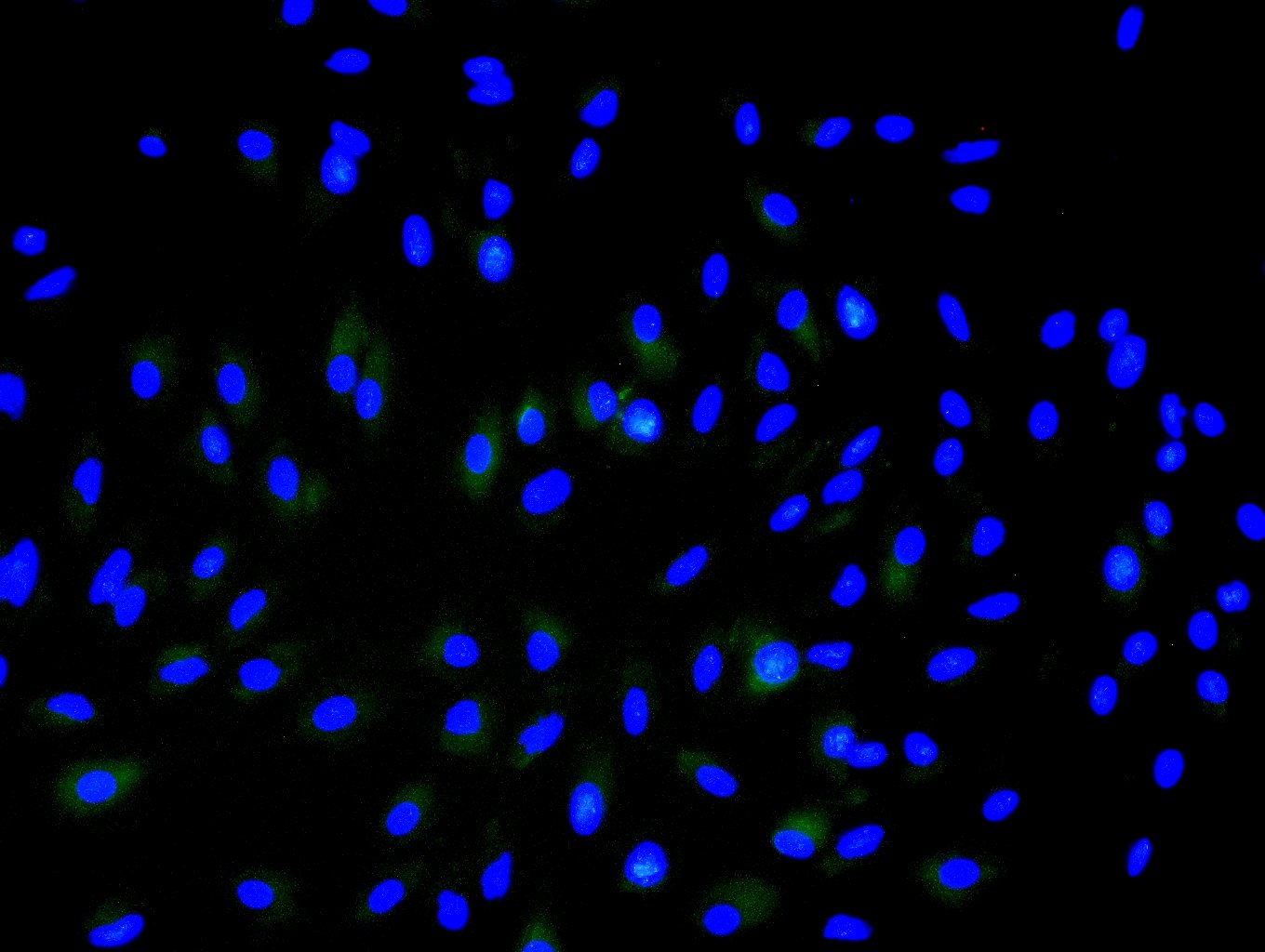

Supplement: Supplementary file 4 — Source data Fig. 2 [file 44318_2024_220_MOESM4_ESM.zip › Figure2/2C/RSA2 (3).jpg]

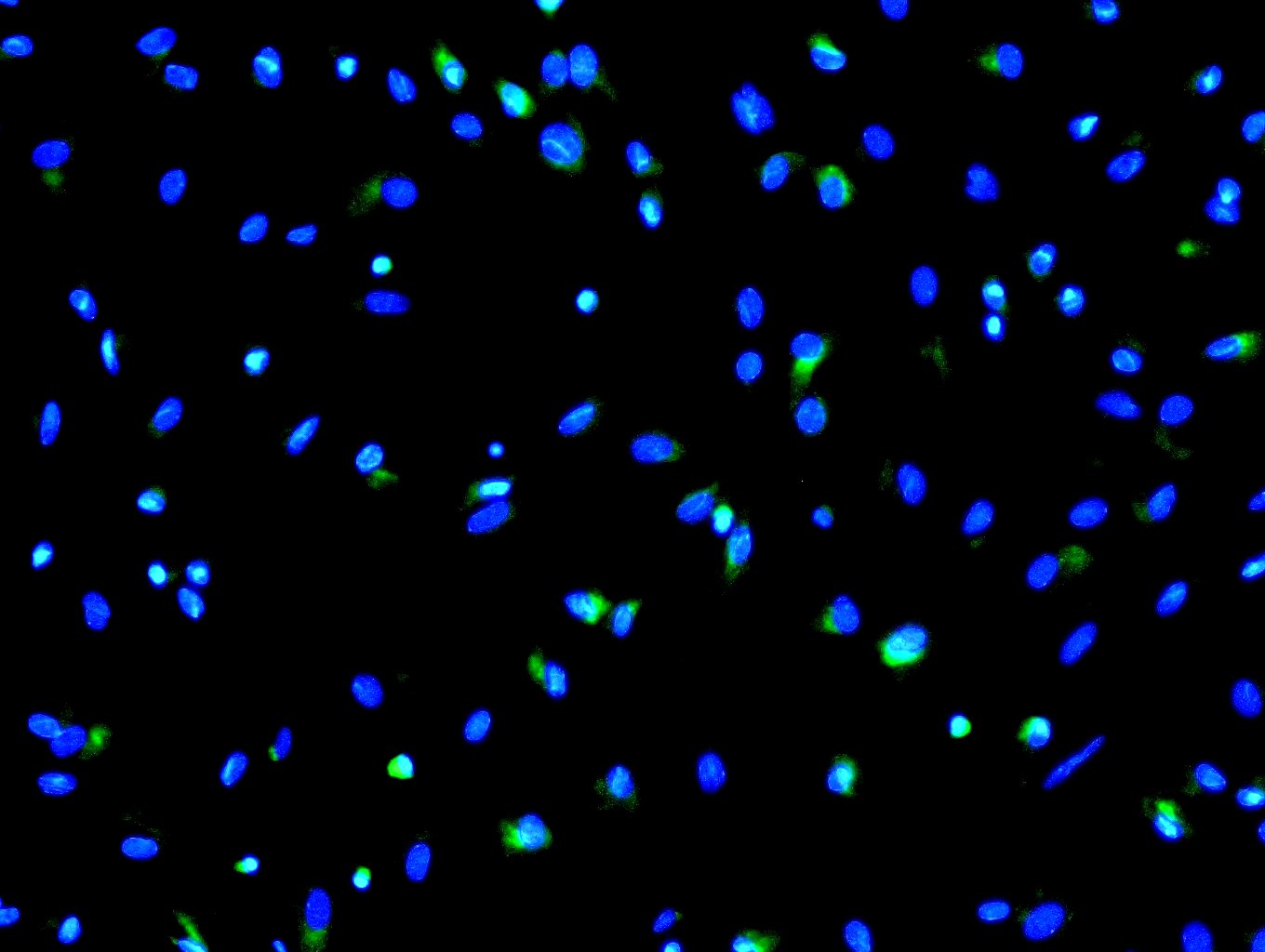

Supplement: Supplementary file 4 — Source data Fig. 2 [file 44318_2024_220_MOESM4_ESM.zip › Figure2/2C/RSA4 (1).jpg]

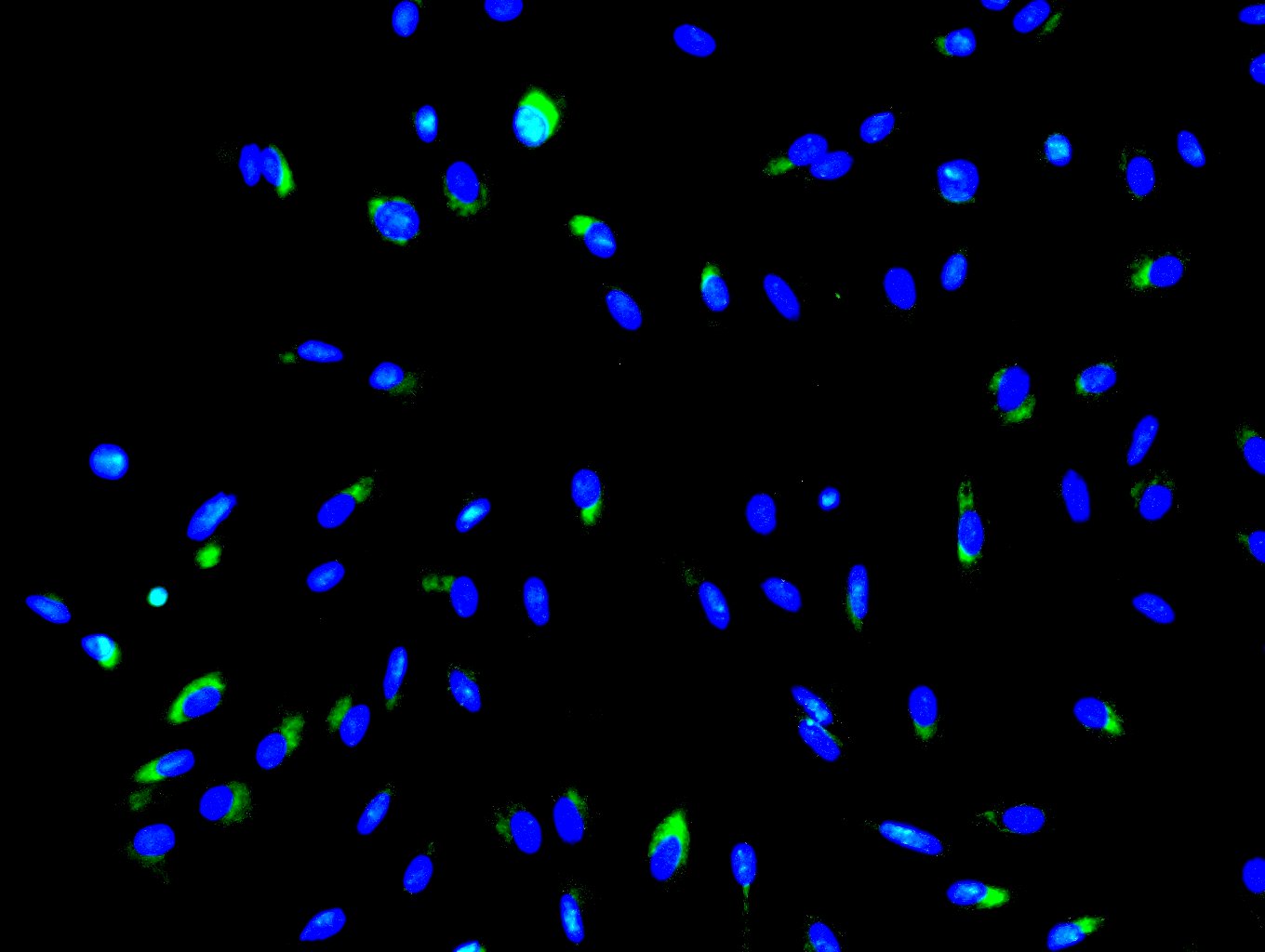

Supplement: Supplementary file 4 — Source data Fig. 2 [file 44318_2024_220_MOESM4_ESM.zip › Figure2/2C/RSA4 (2).jpg]

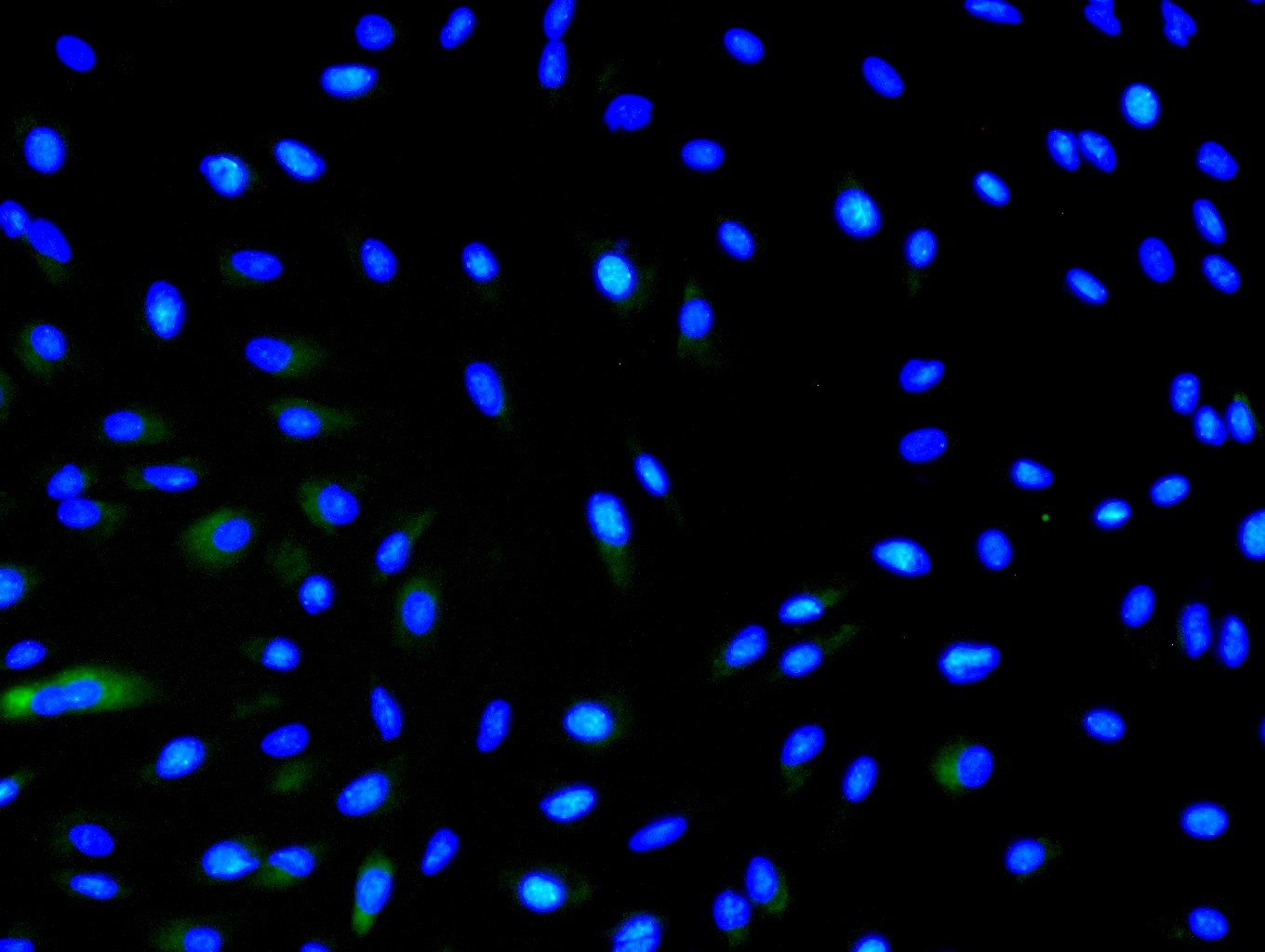

Supplement: Supplementary file 4 — Source data Fig. 2 [file 44318_2024_220_MOESM4_ESM.zip › Figure2/2C/RSA4 (3).jpg]

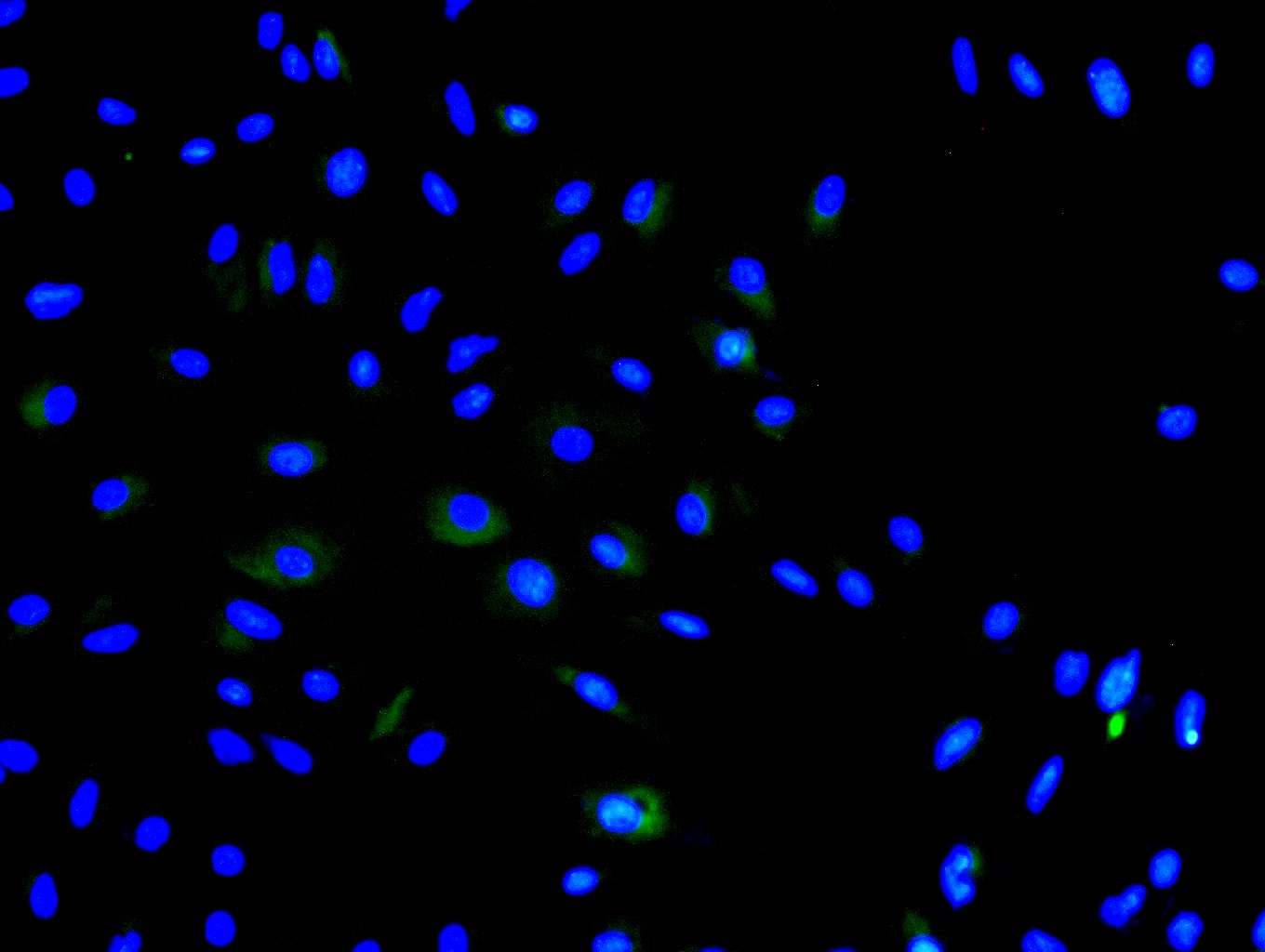

Supplement: Supplementary file 4 — Source data Fig. 2 [file 44318_2024_220_MOESM4_ESM.zip › Figure2/2C/RSA4 (4).jpg]

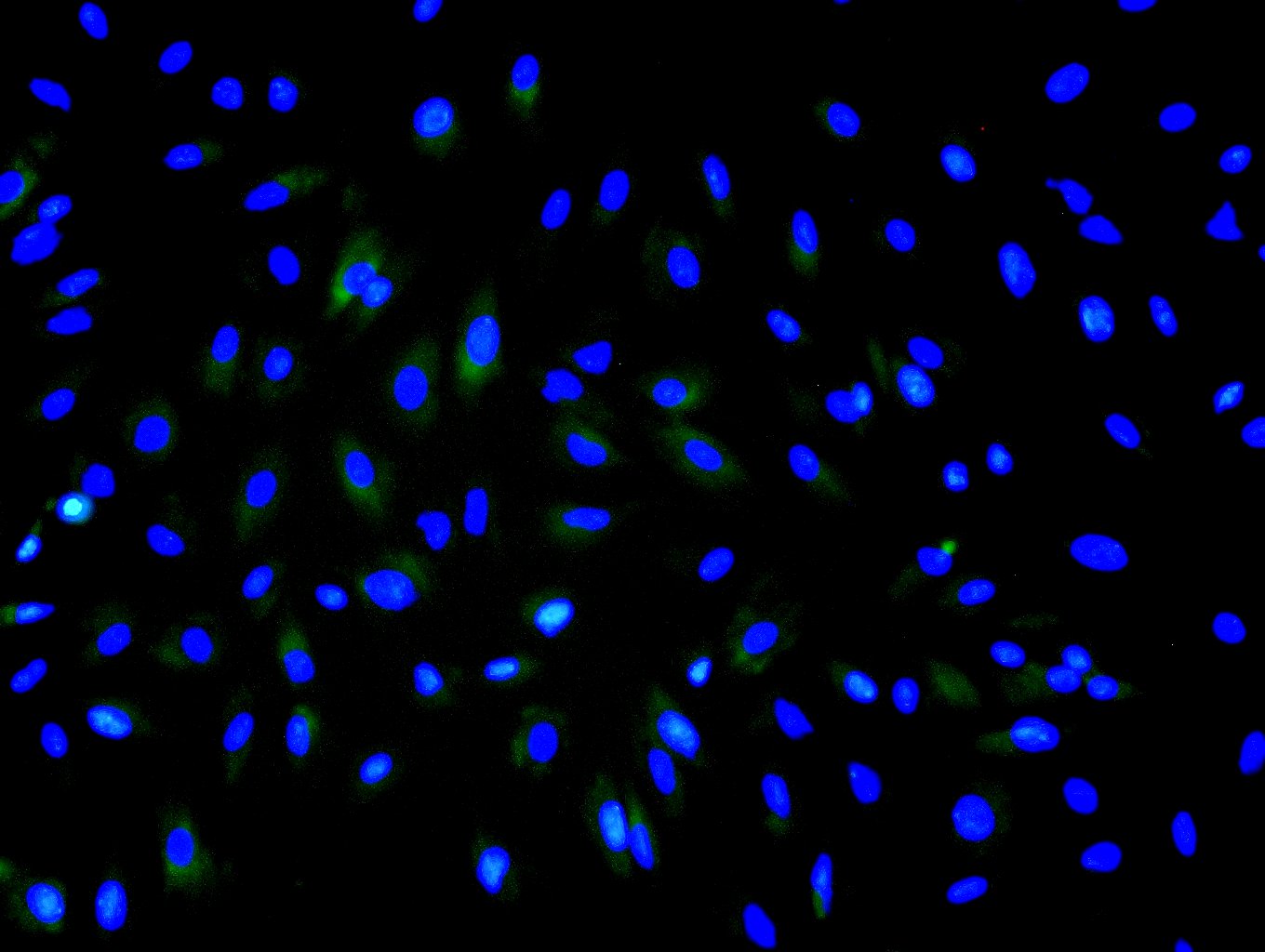

Supplement: Supplementary file 4 — Source data Fig. 2 [file 44318_2024_220_MOESM4_ESM.zip › Figure2/2C/RSA4 (5).jpg]

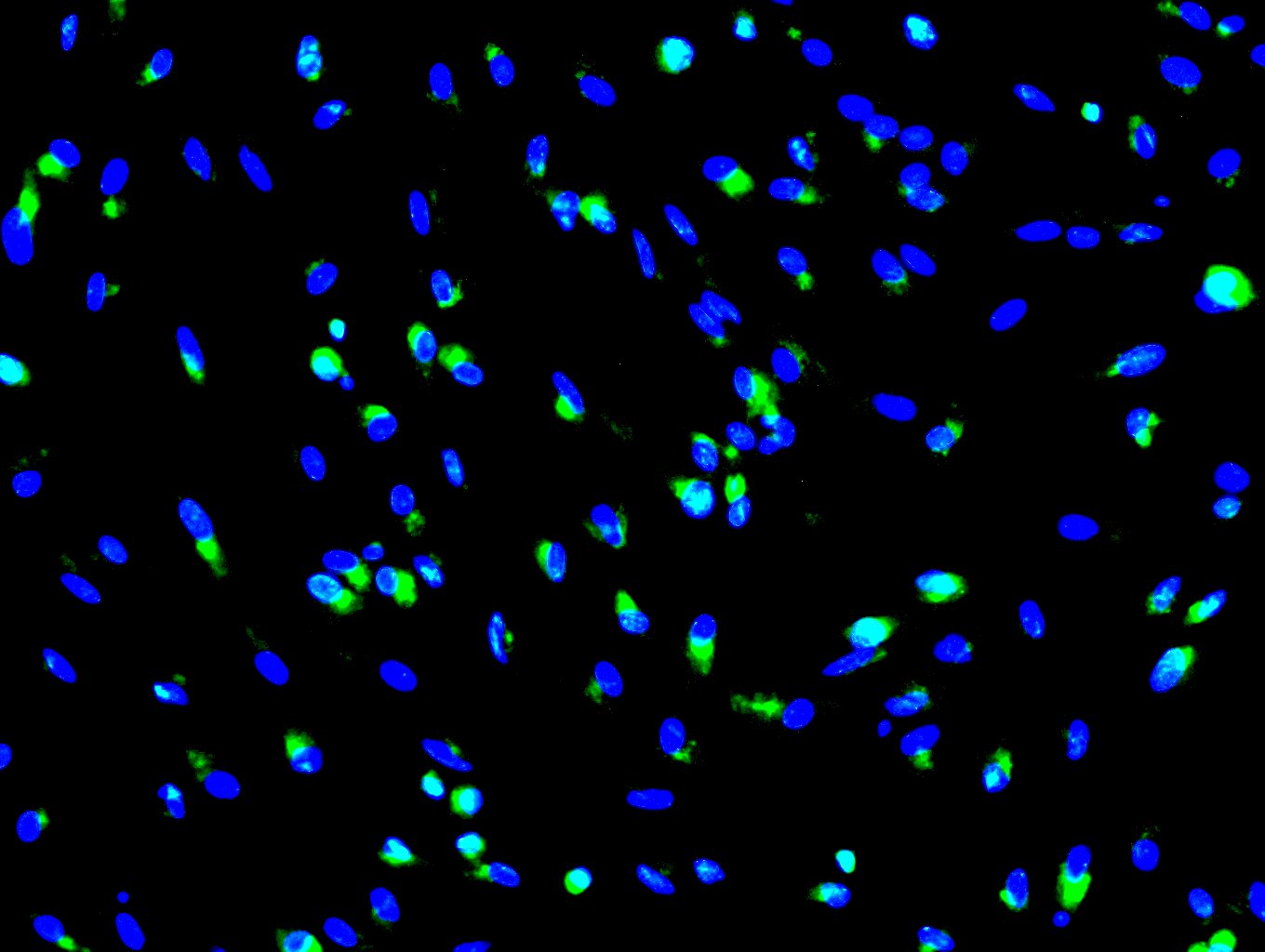

Supplement: Supplementary file 4 — Source data Fig. 2 [file 44318_2024_220_MOESM4_ESM.zip › Figure2/2C/RSA4 (6).jpg]

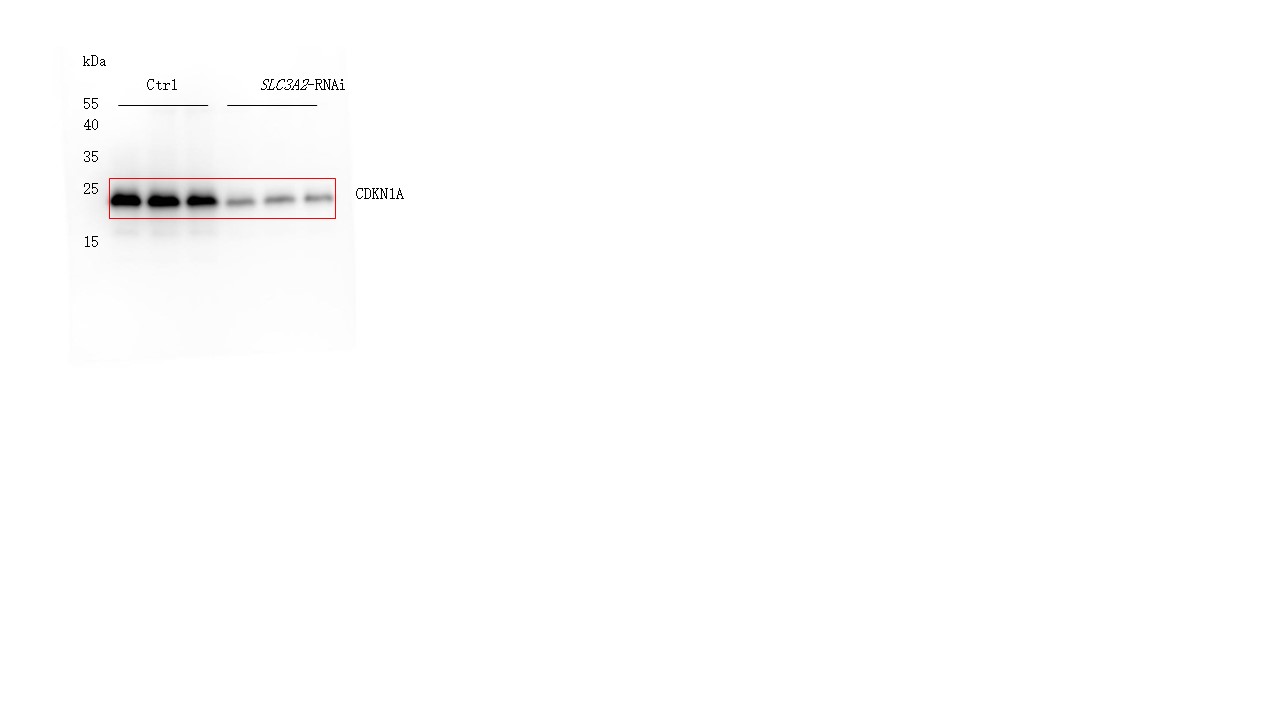

Supplement: Supplementary file 5 — Source data Fig. 3 [file 44318_2024_220_MOESM5_ESM.zip › Figure3/3C/western CDKN1A.jpg]

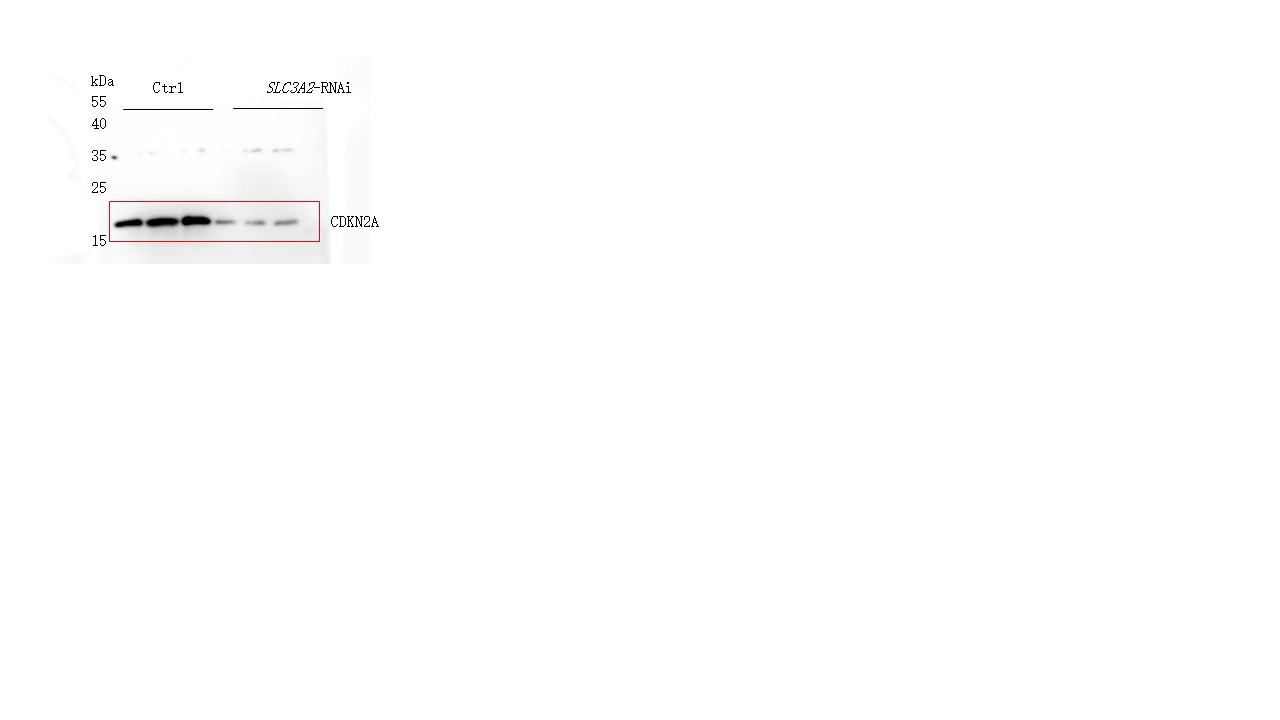

Supplement: Supplementary file 5 — Source data Fig. 3 [file 44318_2024_220_MOESM5_ESM.zip › Figure3/3C/western CDKN2A.jpg]

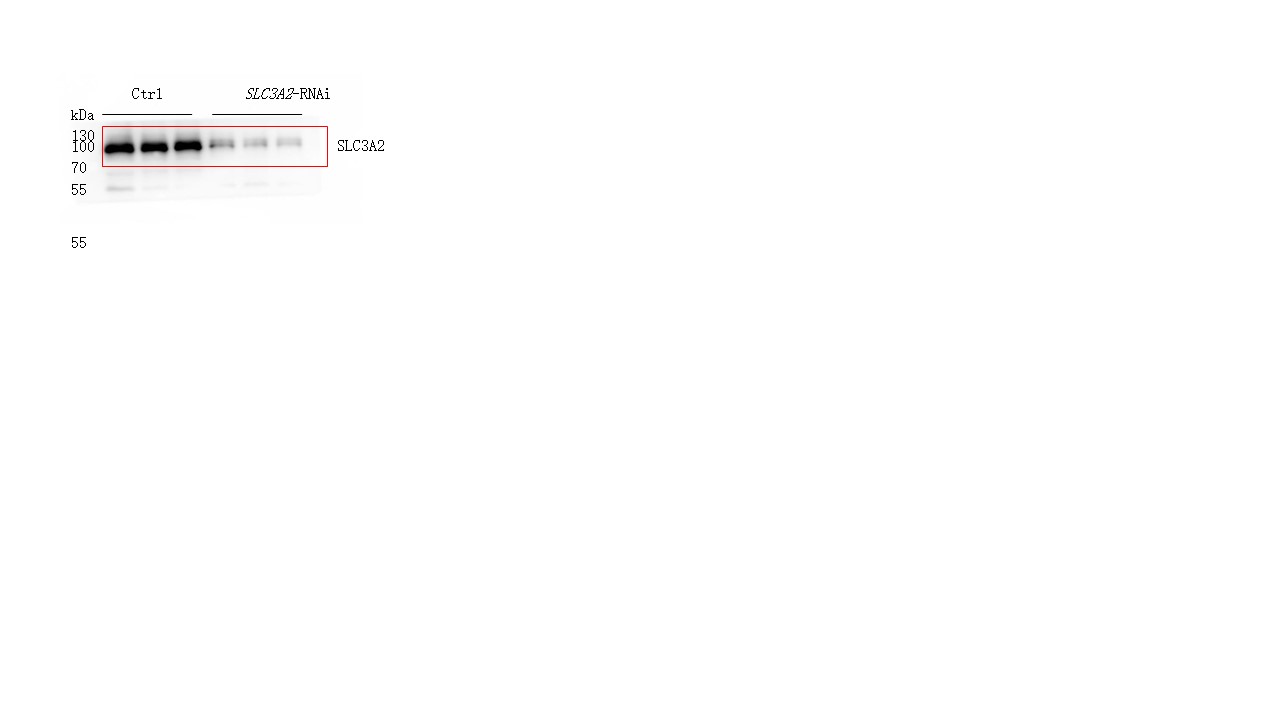

Supplement: Supplementary file 5 — Source data Fig. 3 [file 44318_2024_220_MOESM5_ESM.zip › Figure3/3C/western SLC3A2.jpg]

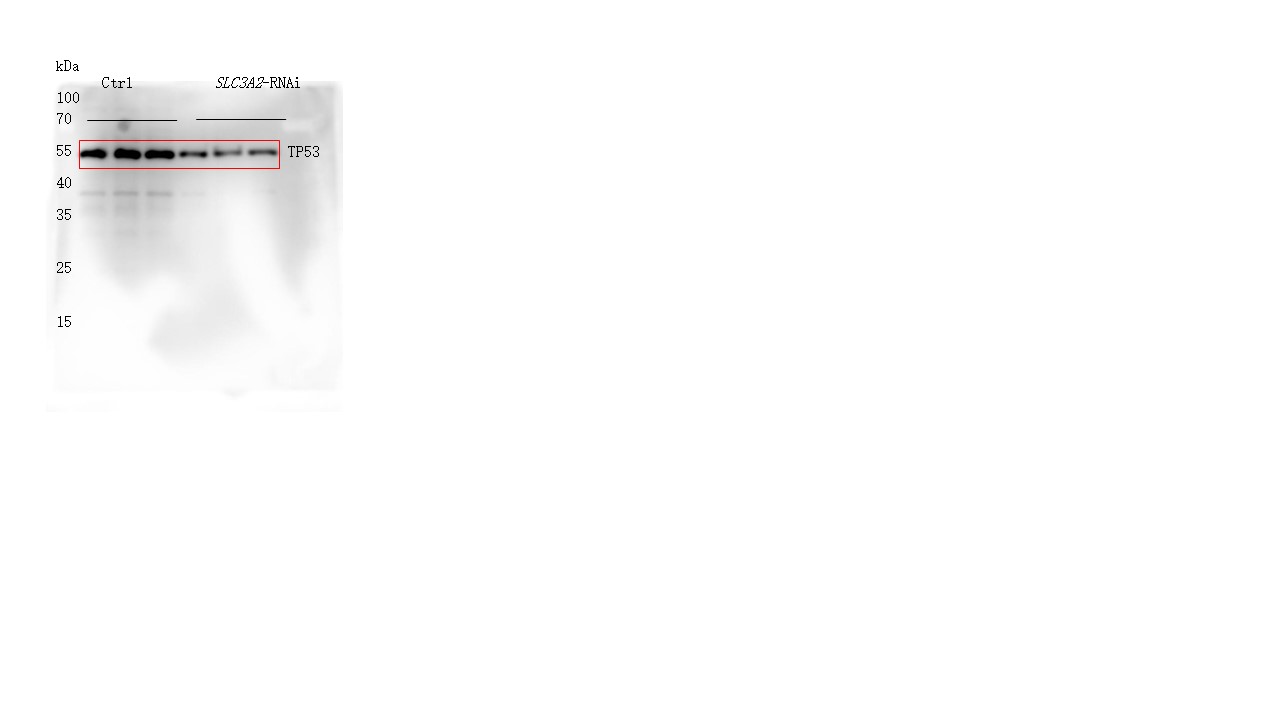

Supplement: Supplementary file 5 — Source data Fig. 3 [file 44318_2024_220_MOESM5_ESM.zip › Figure3/3C/western TP53.jpg]

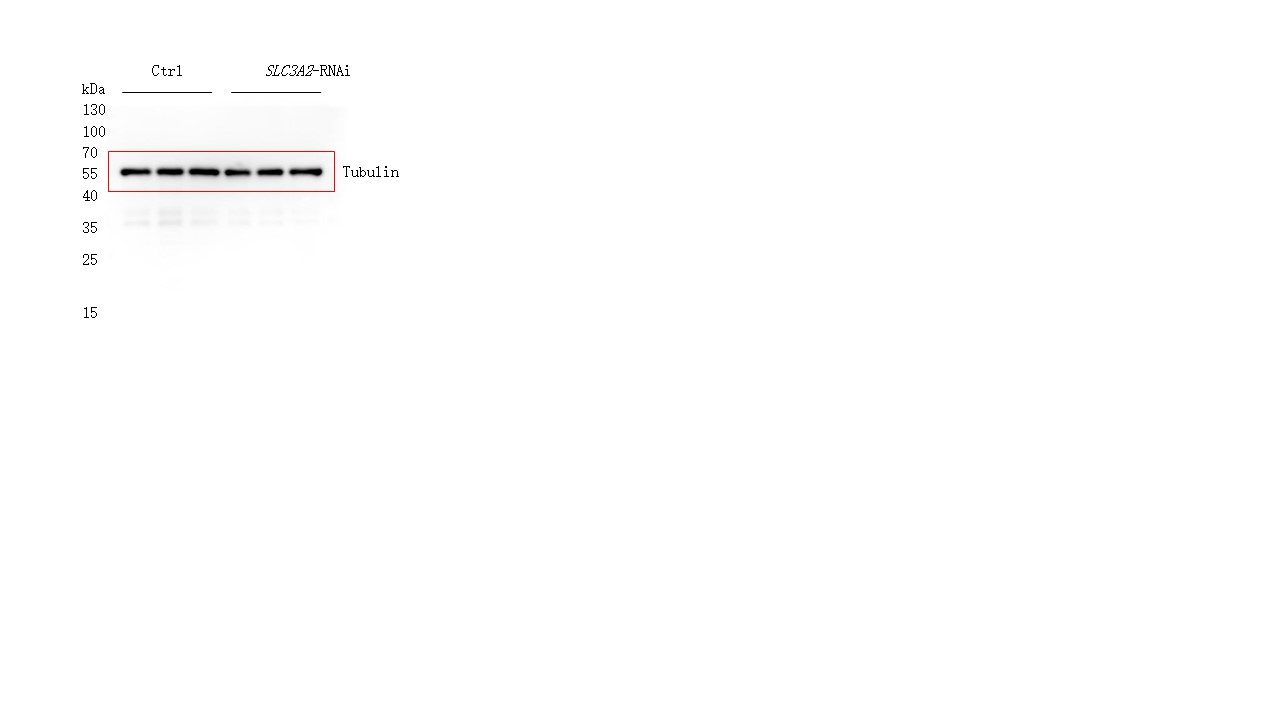

Supplement: Supplementary file 5 — Source data Fig. 3 [file 44318_2024_220_MOESM5_ESM.zip › Figure3/3C/western Tubulin.jpg]

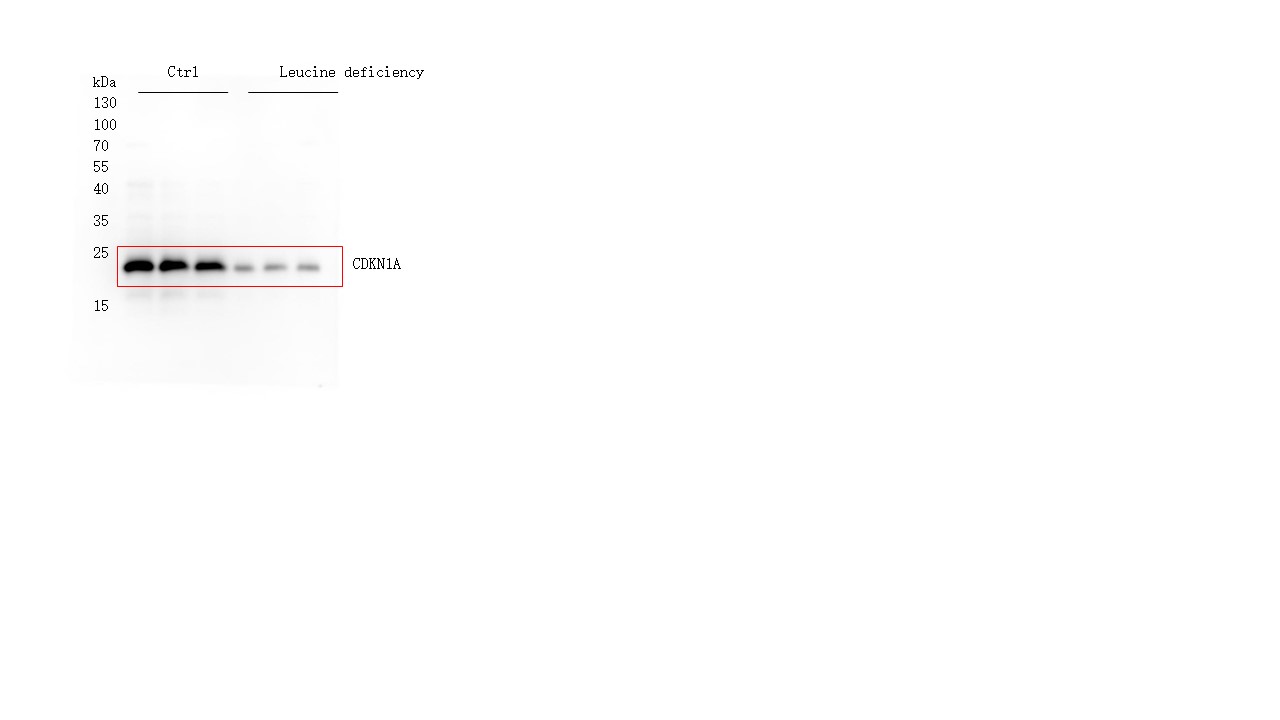

Supplement: Supplementary file 5 — Source data Fig. 3 [file 44318_2024_220_MOESM5_ESM.zip › Figure3/3D/western CDKN1A.jpg]

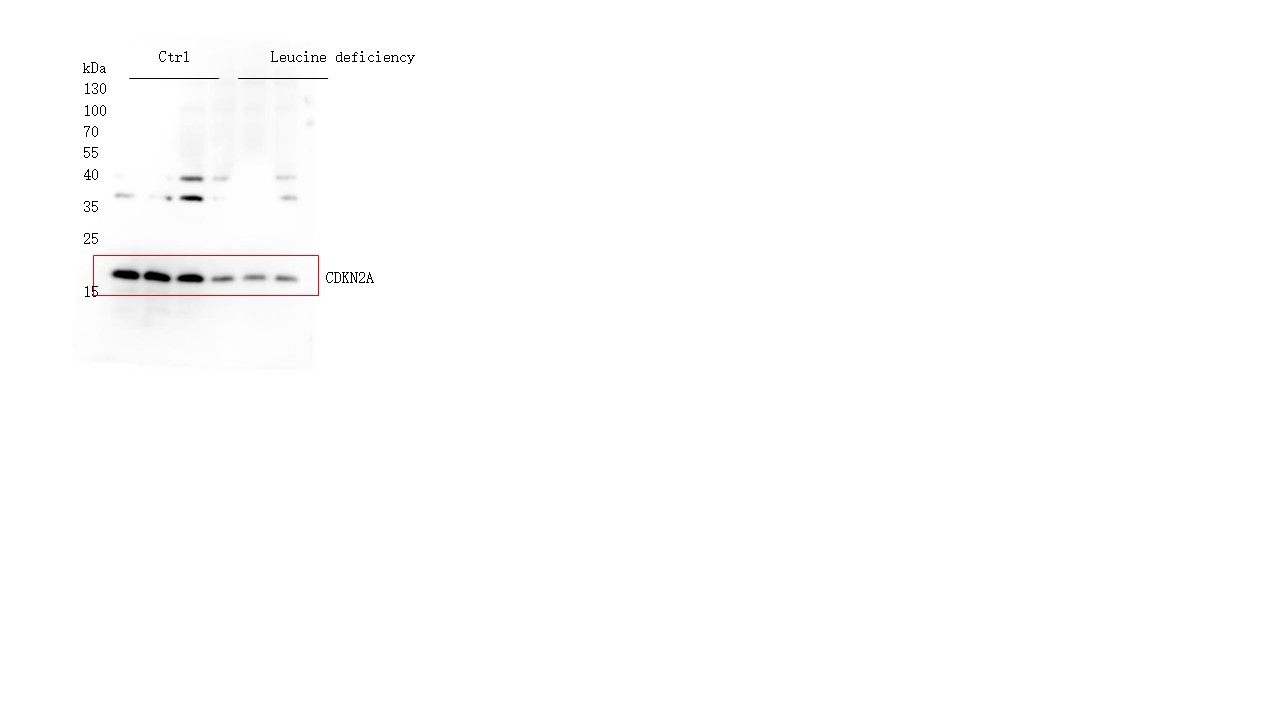

Supplement: Supplementary file 5 — Source data Fig. 3 [file 44318_2024_220_MOESM5_ESM.zip › Figure3/3D/western CDKN2A.jpg]

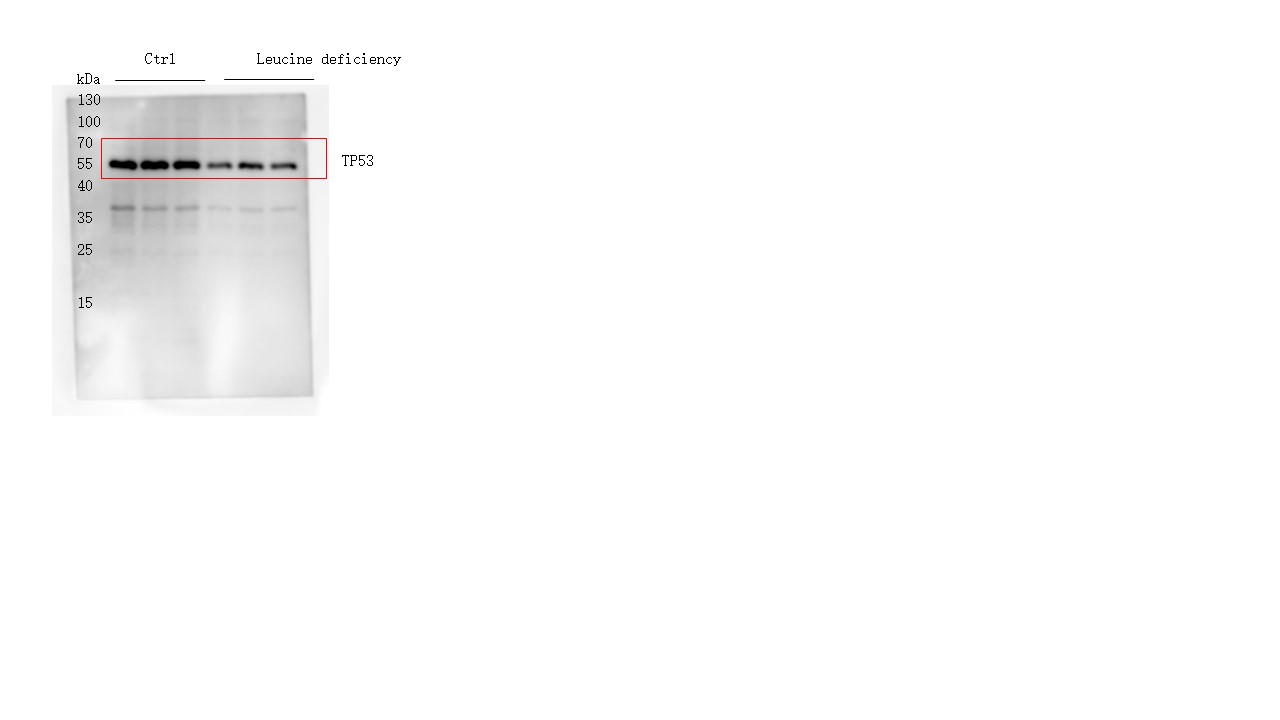

Supplement: Supplementary file 5 — Source data Fig. 3 [file 44318_2024_220_MOESM5_ESM.zip › Figure3/3D/western TP53.jpg]

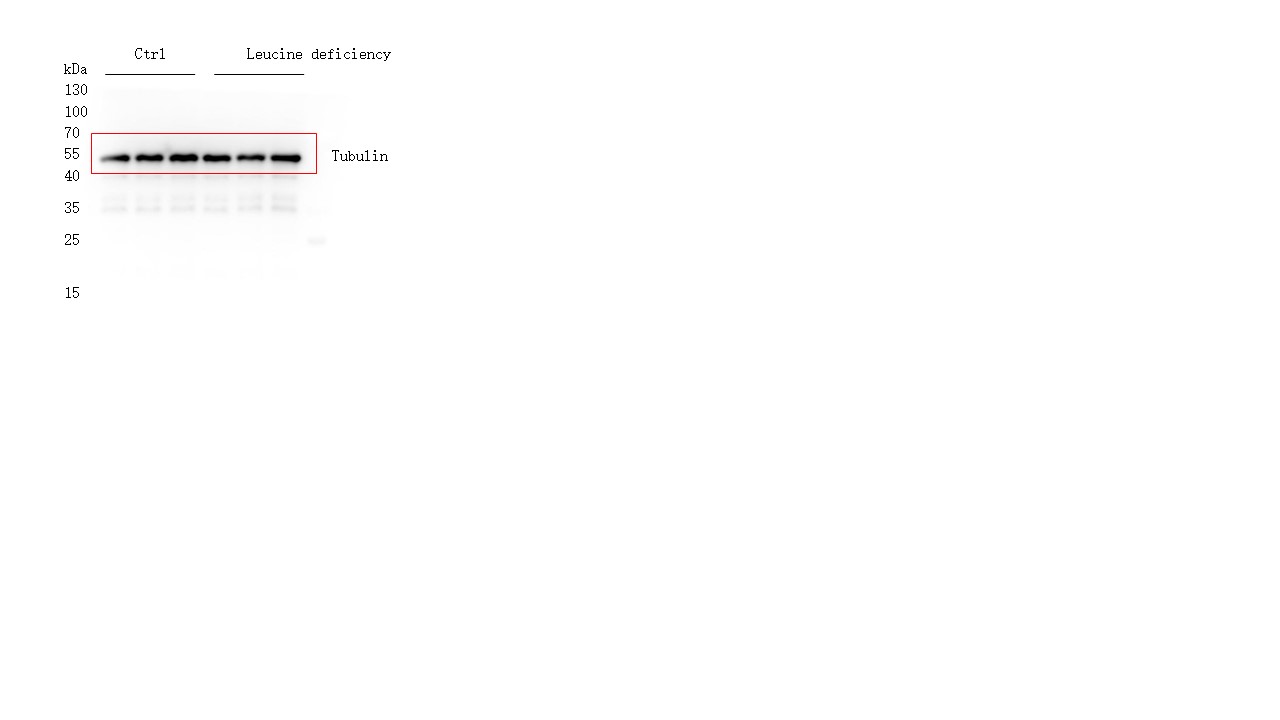

Supplement: Supplementary file 5 — Source data Fig. 3 [file 44318_2024_220_MOESM5_ESM.zip › Figure3/3D/western Tubulin.jpg]

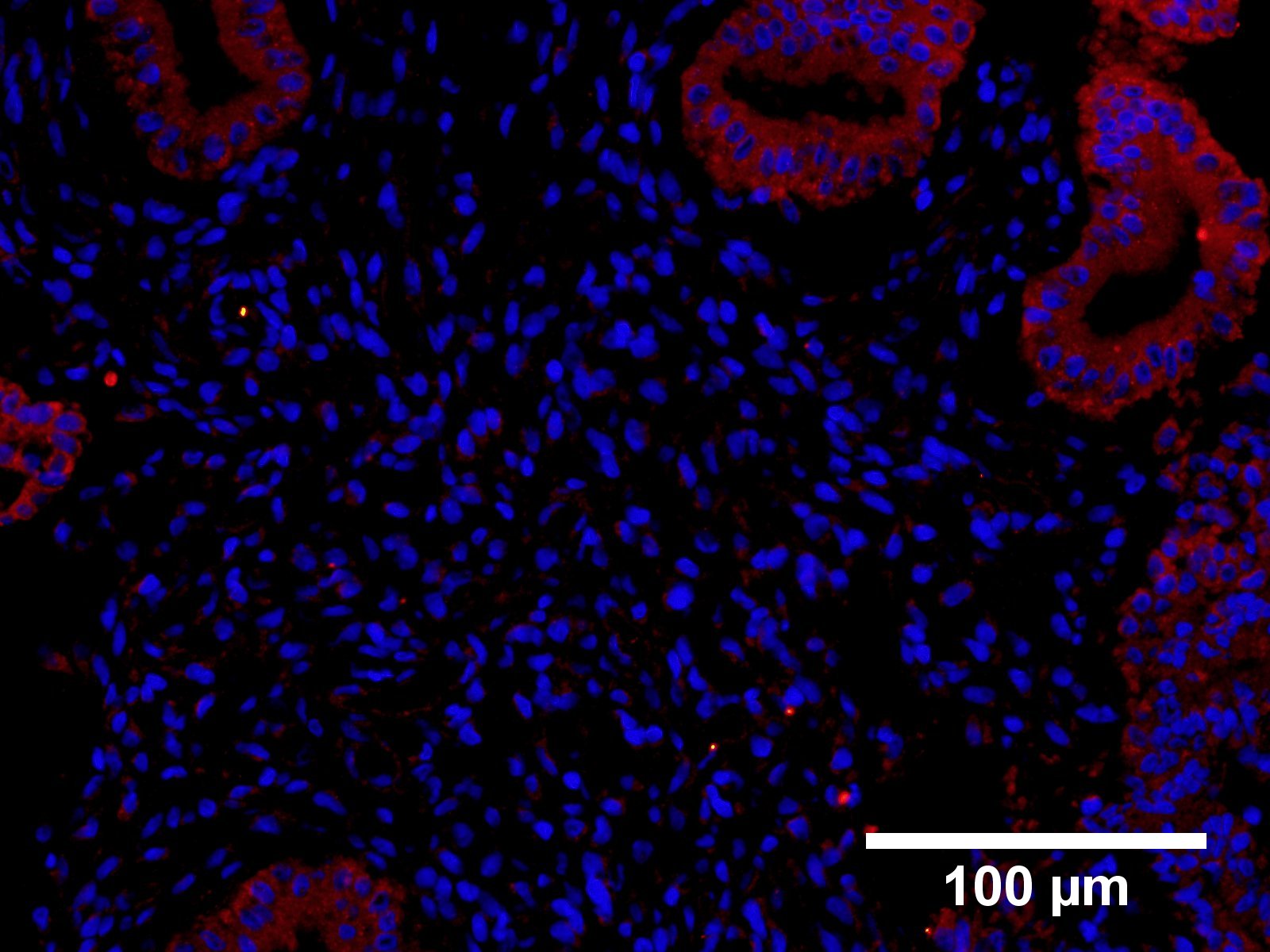

Supplement: Supplementary file 5 — Source data Fig. 3 [file 44318_2024_220_MOESM5_ESM.zip › Figure3/3F/endometrium-400 (1).jpg]

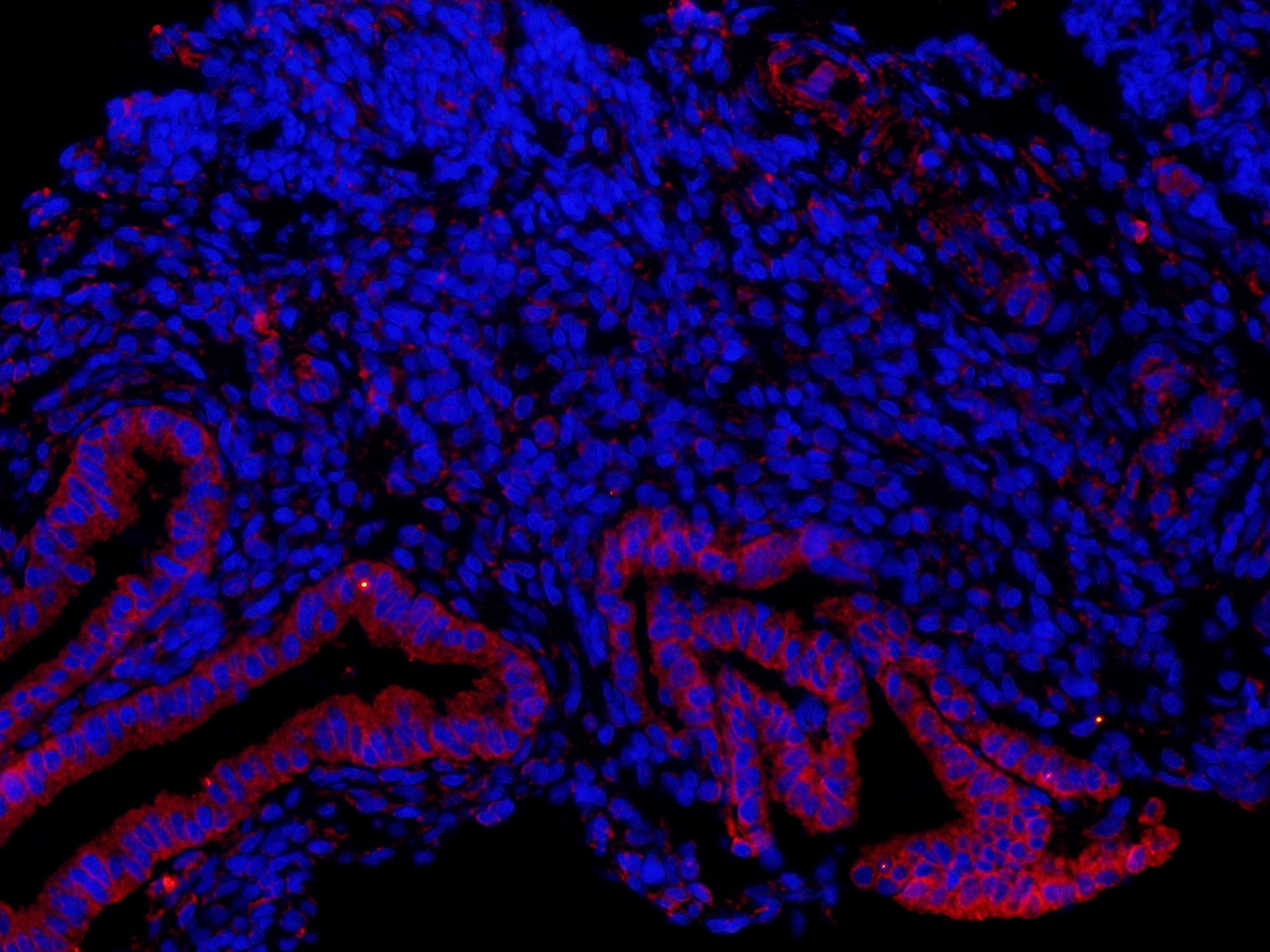

Supplement: Supplementary file 5 — Source data Fig. 3 [file 44318_2024_220_MOESM5_ESM.zip › Figure3/3F/endometrium-400 (2).jpg]

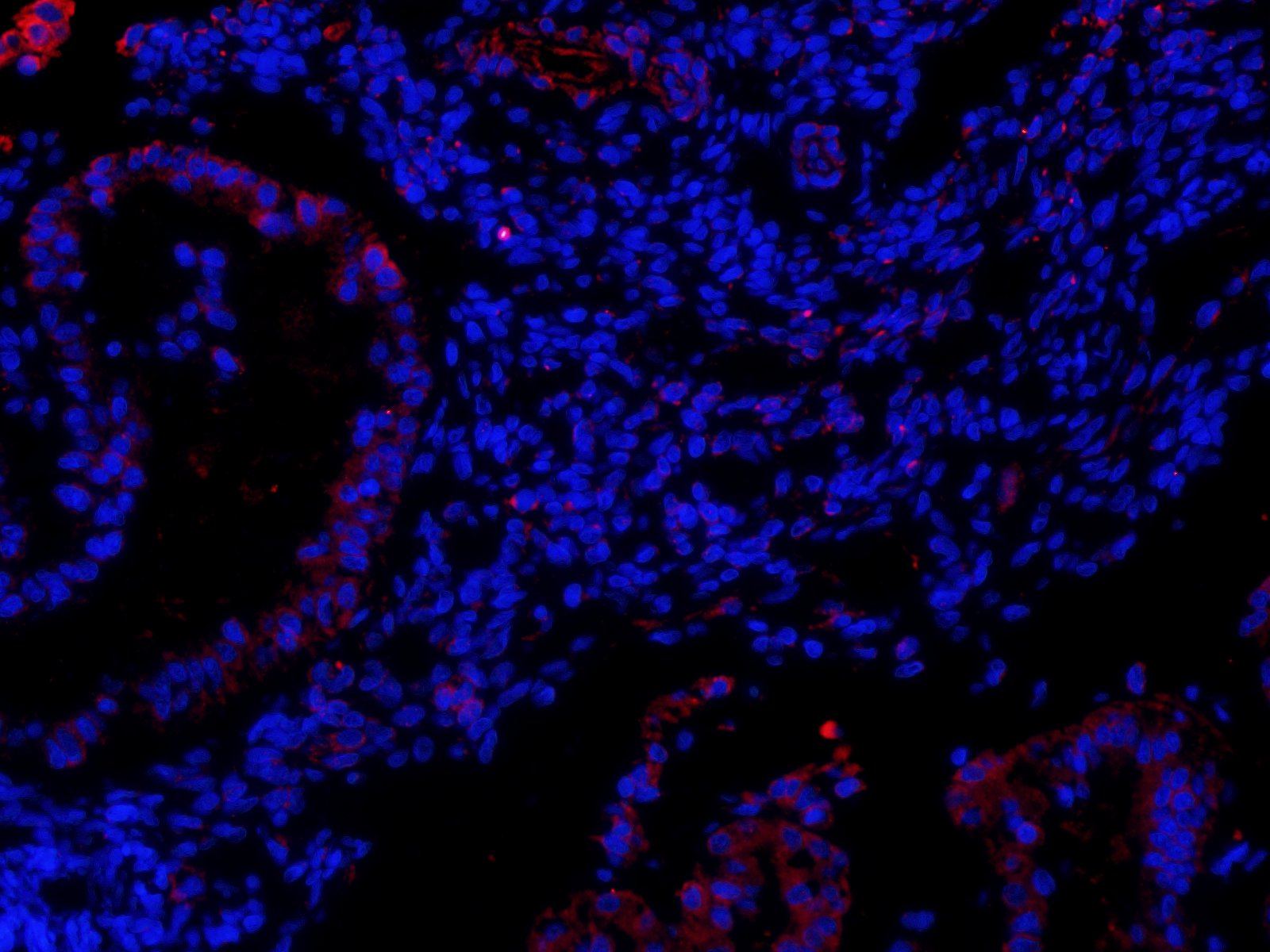

Supplement: Supplementary file 5 — Source data Fig. 3 [file 44318_2024_220_MOESM5_ESM.zip › Figure3/3F/endometrium-400 (3).jpg]

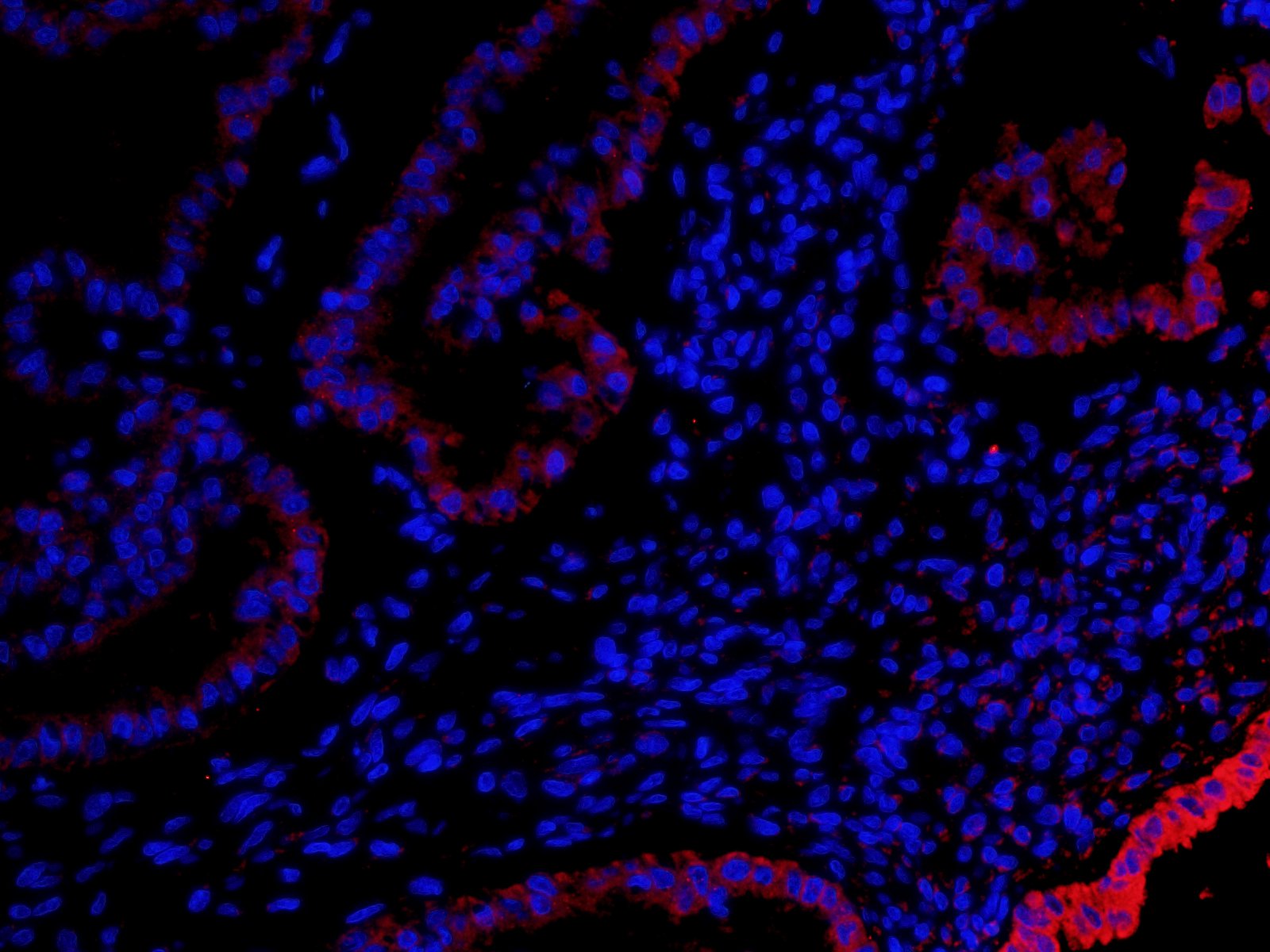

Supplement: Supplementary file 5 — Source data Fig. 3 [file 44318_2024_220_MOESM5_ESM.zip › Figure3/3F/endometrium-400 (4).jpg]

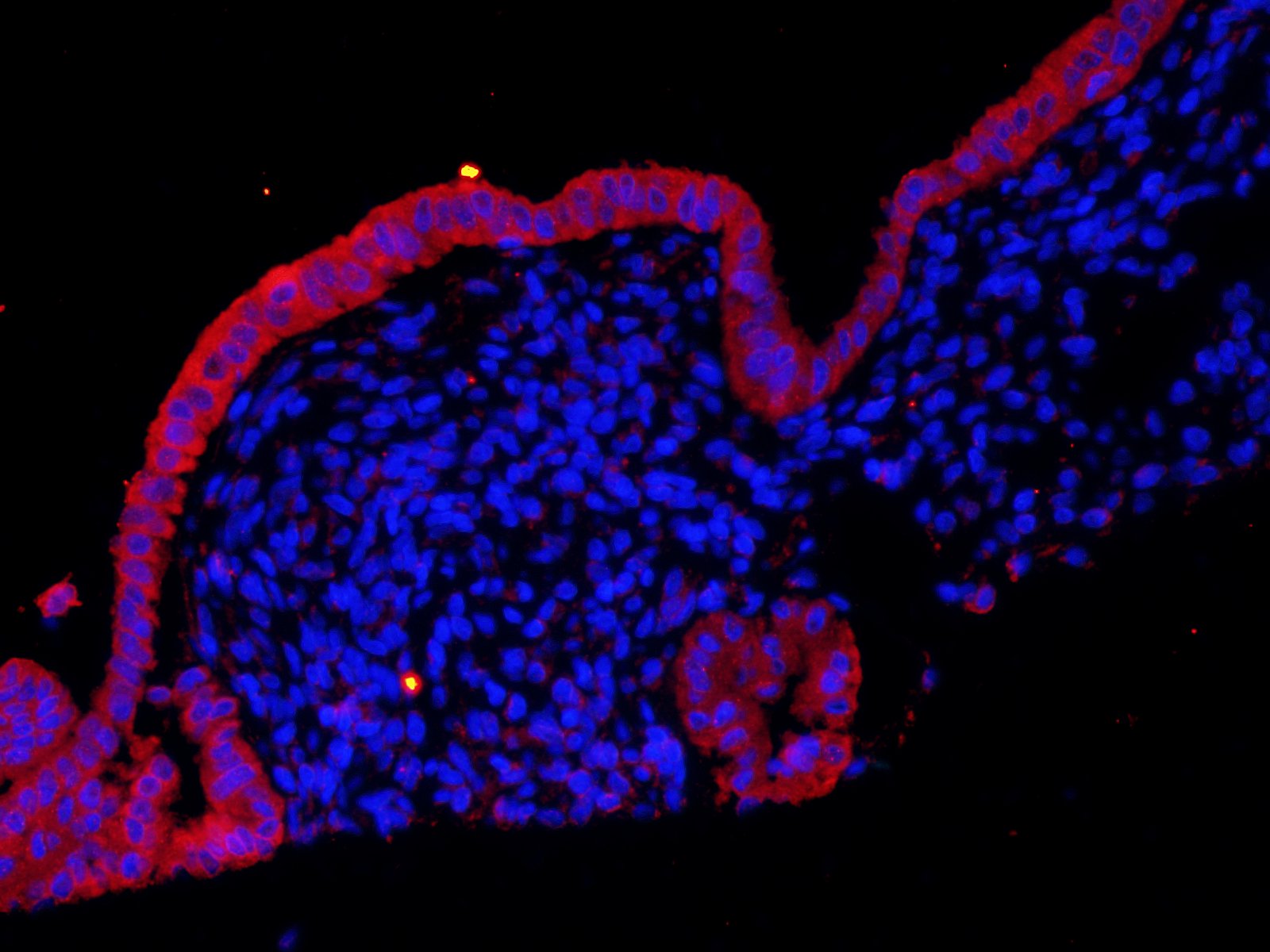

Supplement: Supplementary file 5 — Source data Fig. 3 [file 44318_2024_220_MOESM5_ESM.zip › Figure3/3F/endometrium-400 (5).jpg]

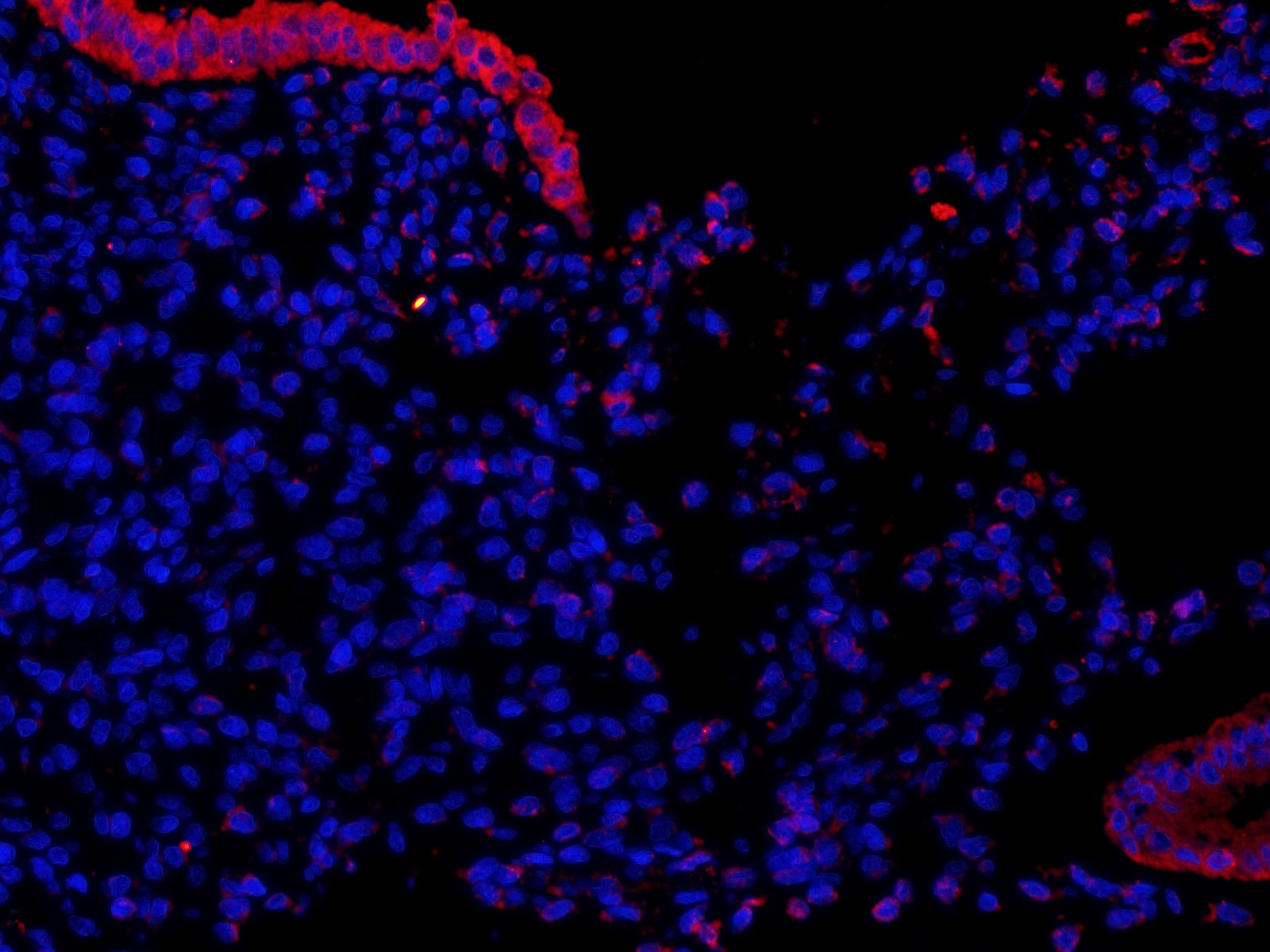

Supplement: Supplementary file 5 — Source data Fig. 3 [file 44318_2024_220_MOESM5_ESM.zip › Figure3/3F/endometrium-400 (6).jpg]

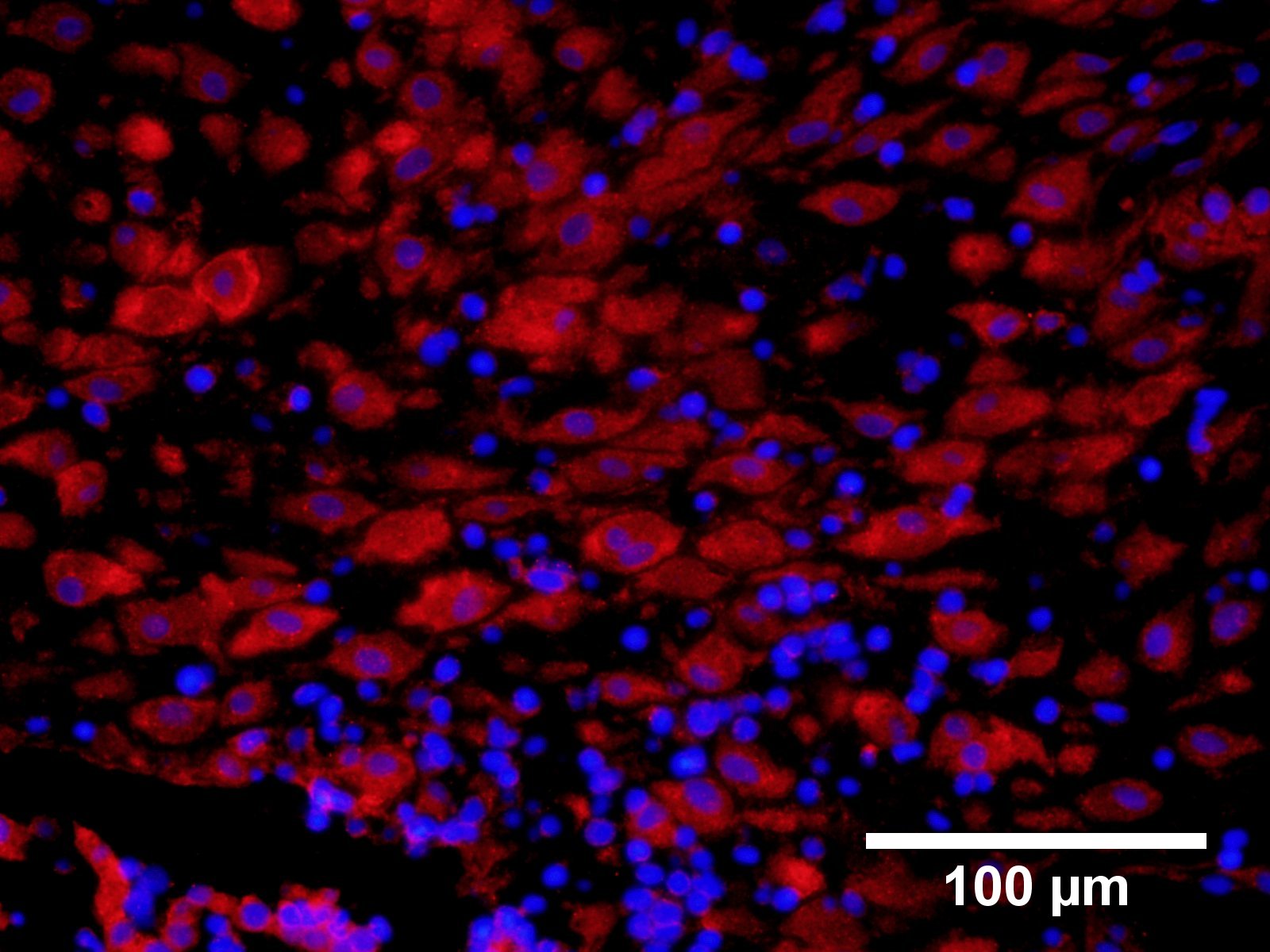

Supplement: Supplementary file 5 — Source data Fig. 3 [file 44318_2024_220_MOESM5_ESM.zip › Figure3/3F/NP-400 (1).jpg]

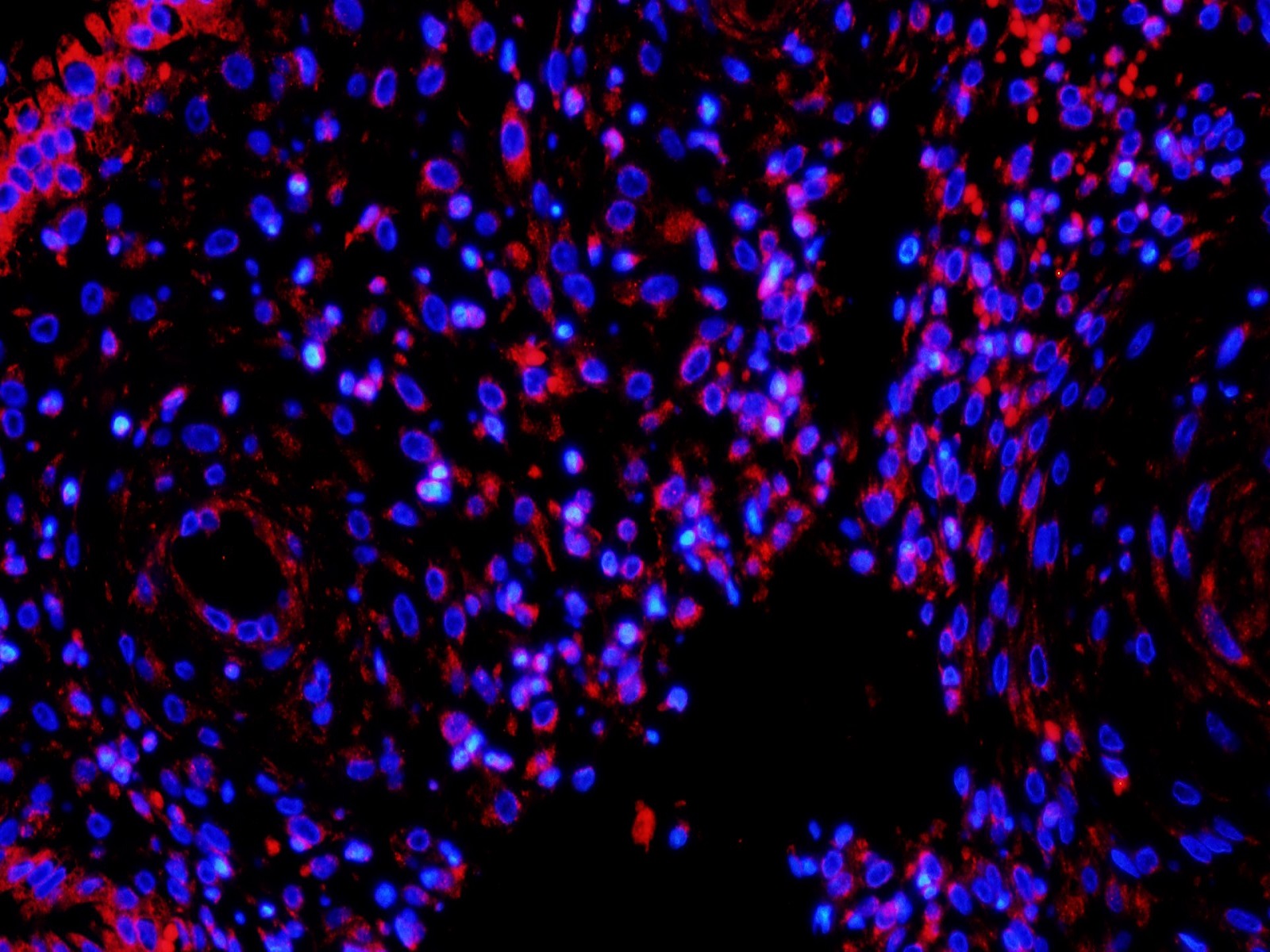

Supplement: Supplementary file 5 — Source data Fig. 3 [file 44318_2024_220_MOESM5_ESM.zip › Figure3/3F/NP-400 (2).jpg]
