# Supplementary figures and images for: TNFSF14+ natural killer cells prevent spontaneous abortion by restricting leucine-mediated decidual stromal cell senescence (part 3 of 4)
Source: EMBO J. 2024 Sep 11;43(21):5018–36. doi: 10.1038/s44318-024-00220-3 (PMC11535022; doi:10.1038/s44318-024-00220-3)

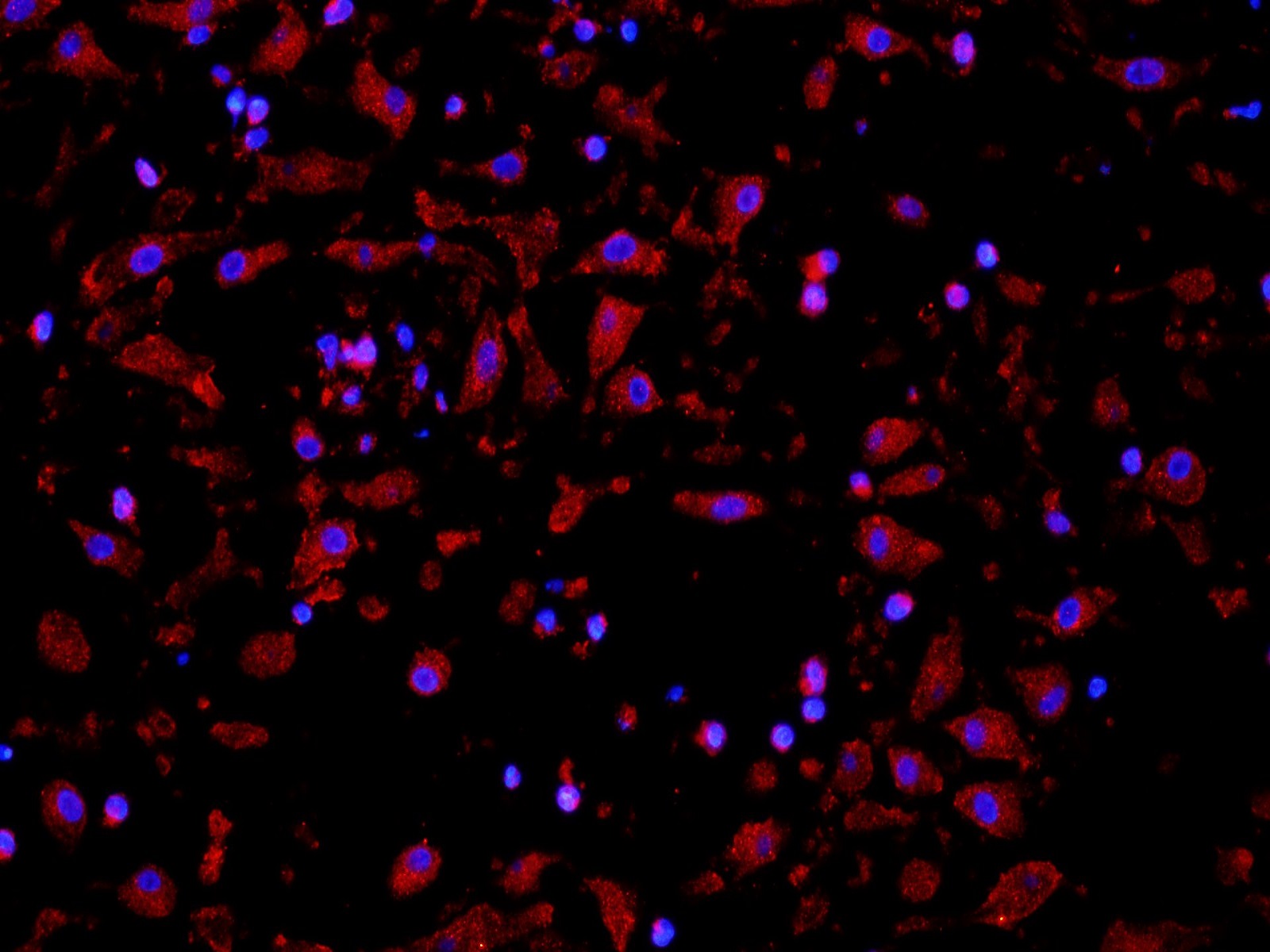

Supplement: Supplementary file 5 — Source data Fig. 3 [file 44318_2024_220_MOESM5_ESM.zip › Figure3/3F/NP-400 (3).jpg]

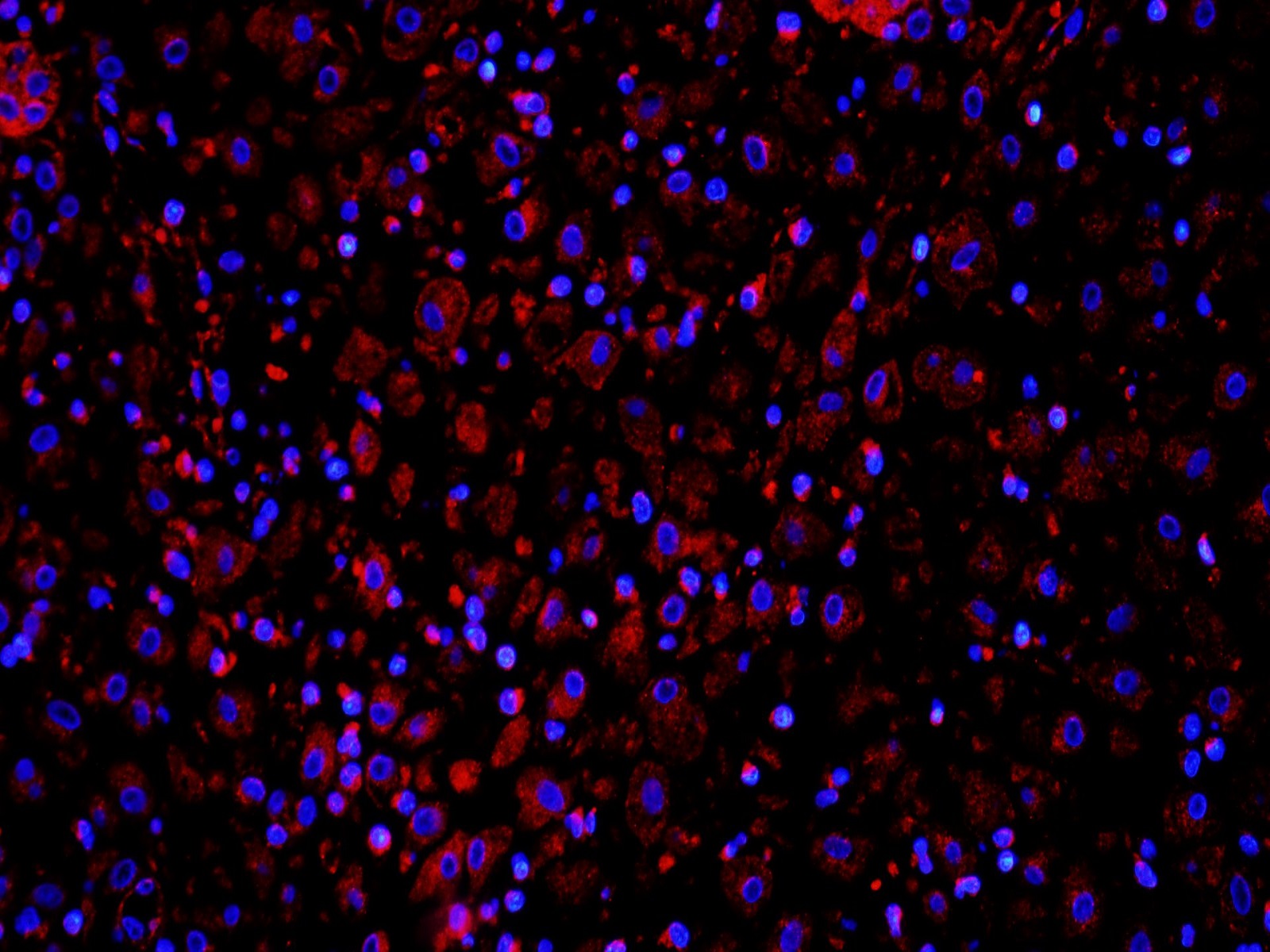

Supplement: Supplementary file 5 — Source data Fig. 3 [file 44318_2024_220_MOESM5_ESM.zip › Figure3/3F/NP-400 (4).jpg]

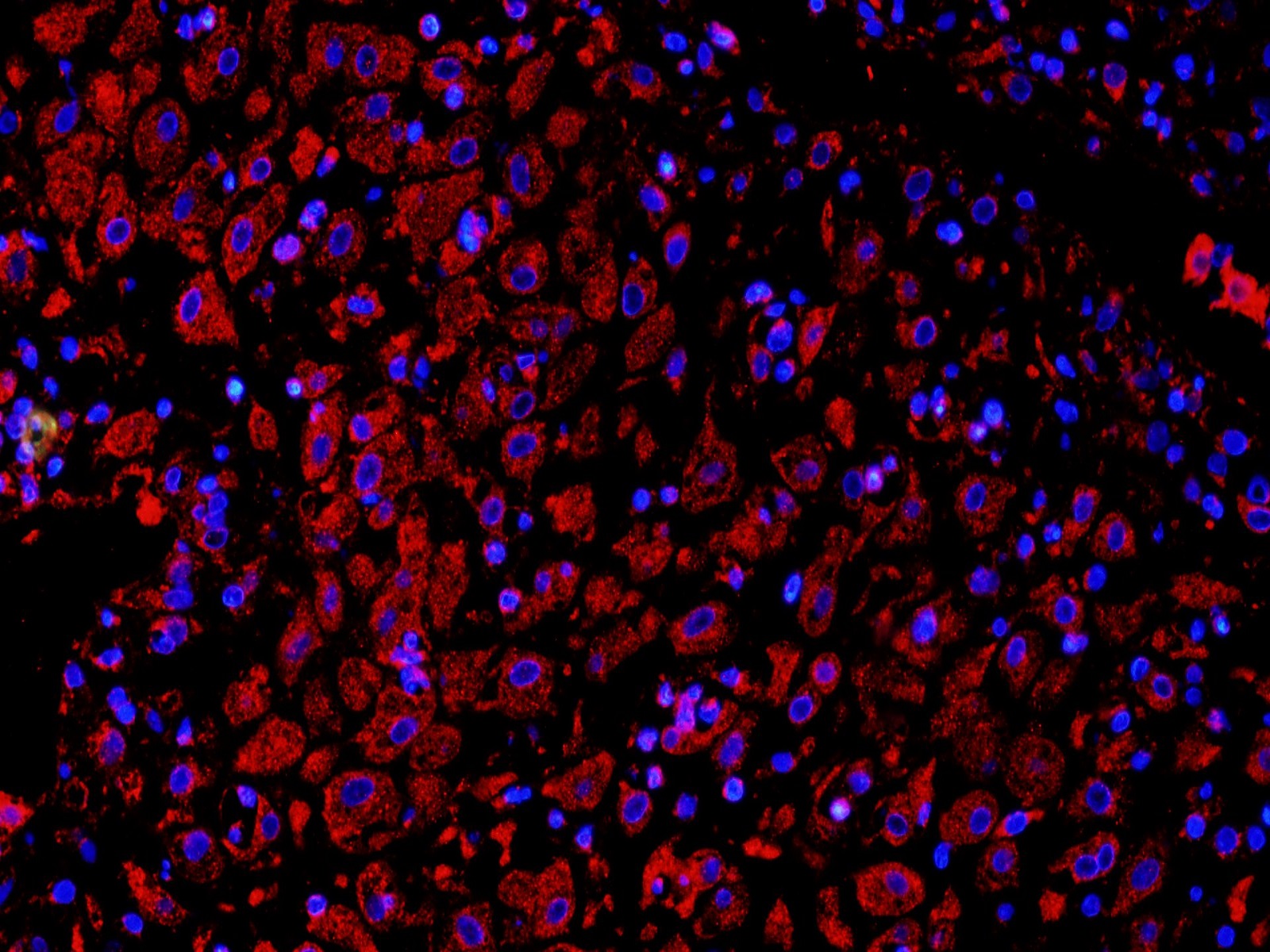

Supplement: Supplementary file 5 — Source data Fig. 3 [file 44318_2024_220_MOESM5_ESM.zip › Figure3/3F/NP-400 (5).jpg]

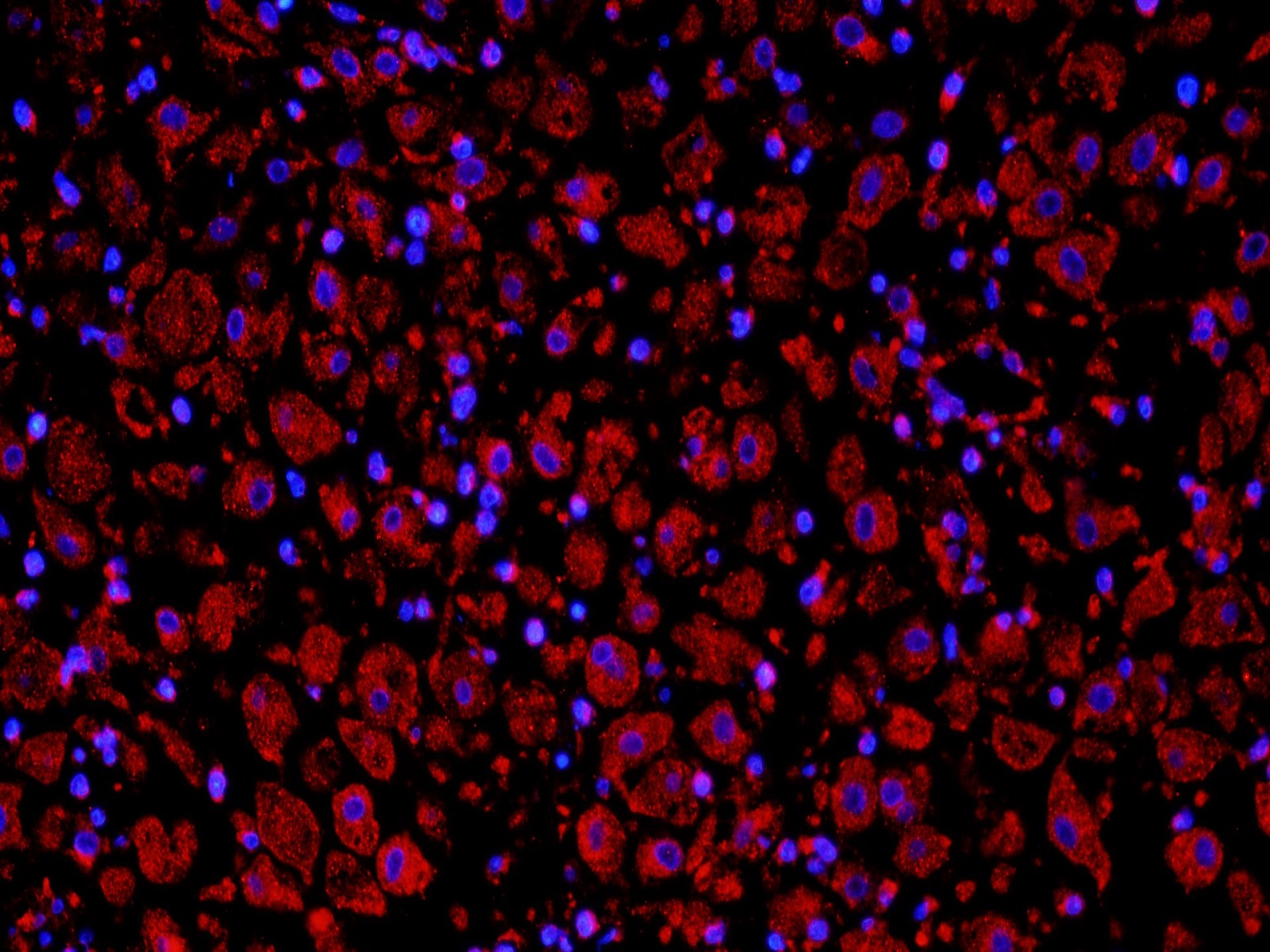

Supplement: Supplementary file 5 — Source data Fig. 3 [file 44318_2024_220_MOESM5_ESM.zip › Figure3/3F/NP-400 (6).jpg]

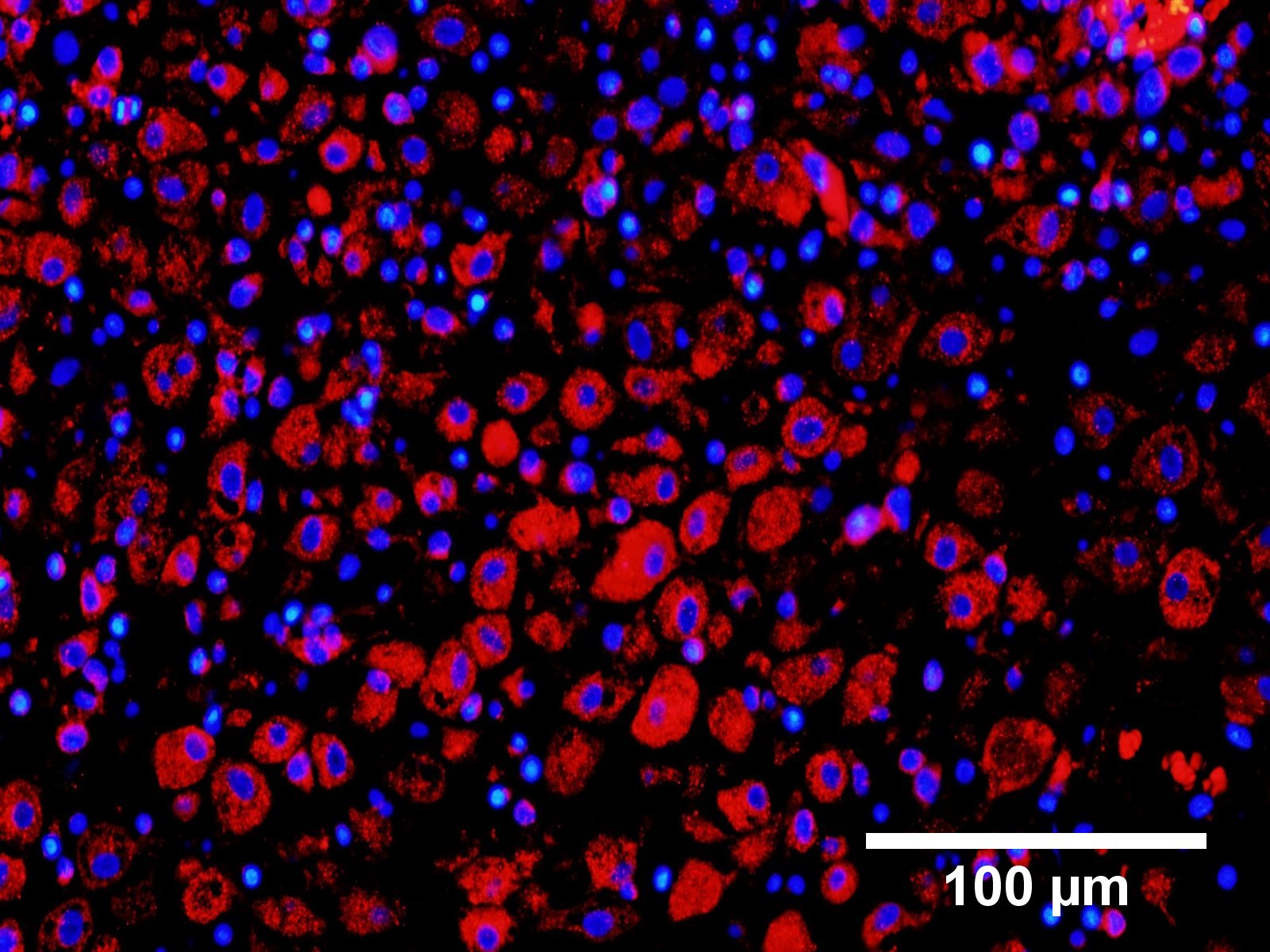

Supplement: Supplementary file 5 — Source data Fig. 3 [file 44318_2024_220_MOESM5_ESM.zip › Figure3/3F/RSA-400 (1).jpg]

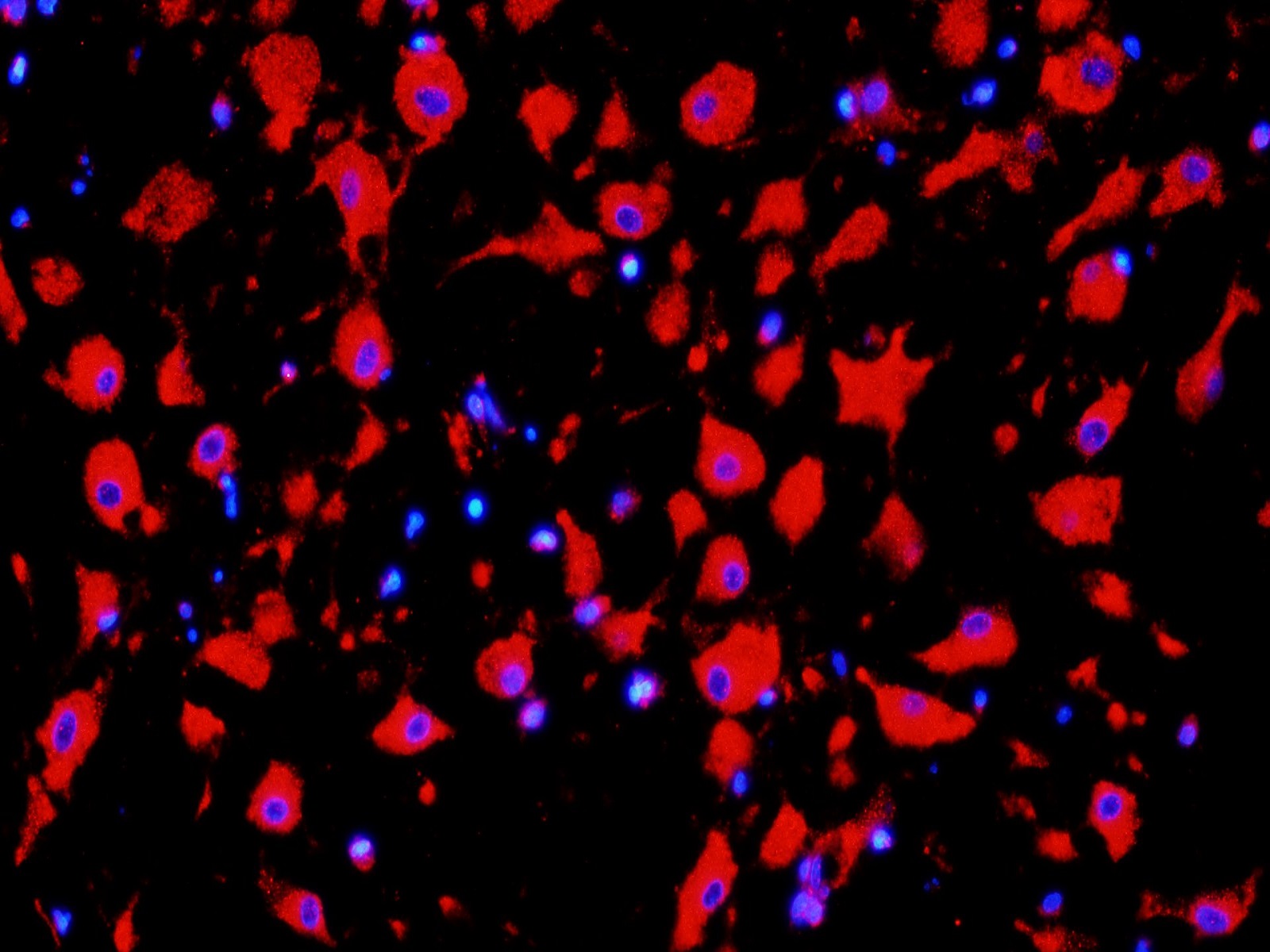

Supplement: Supplementary file 5 — Source data Fig. 3 [file 44318_2024_220_MOESM5_ESM.zip › Figure3/3F/RSA-400 (2).jpg]

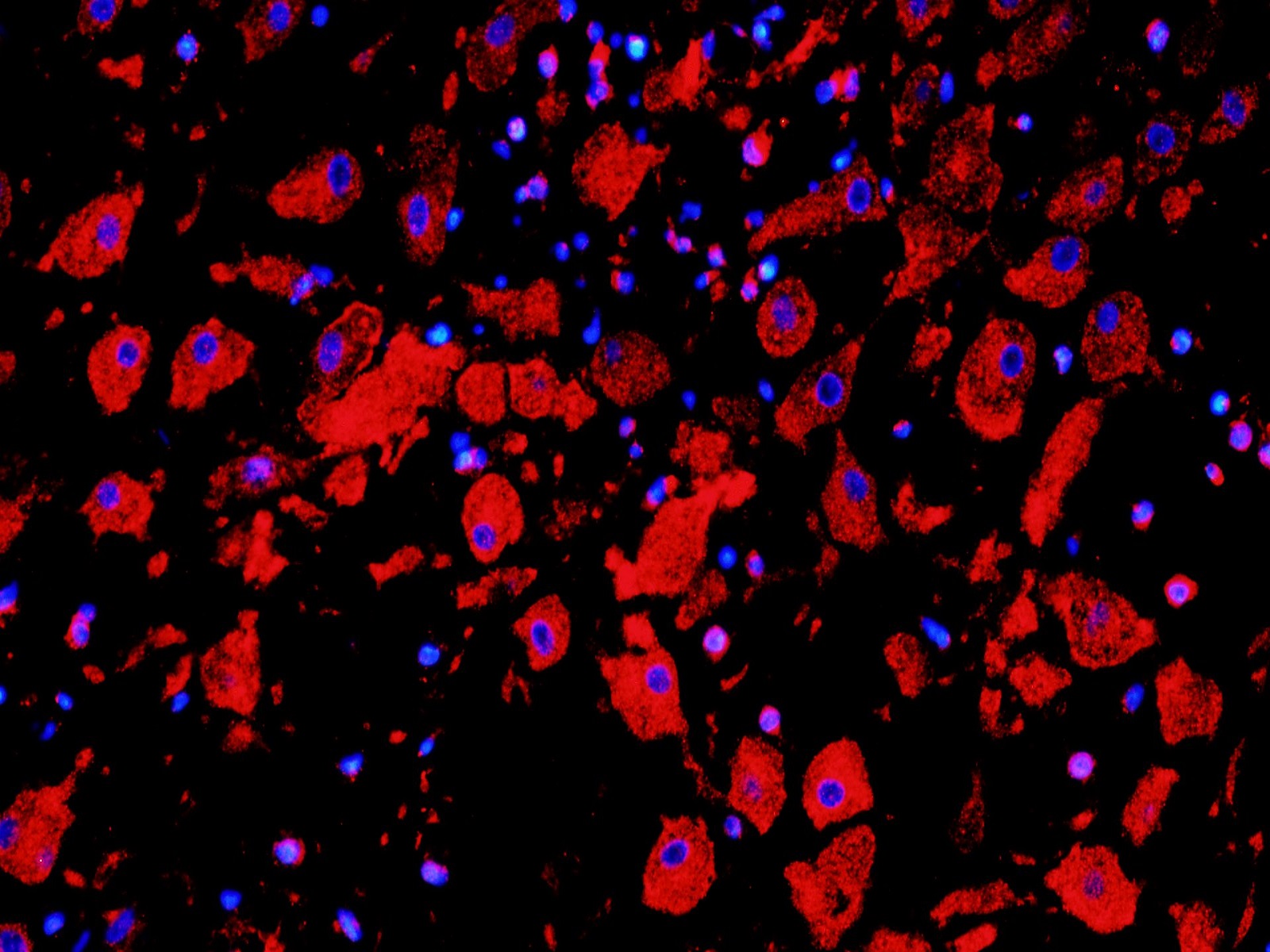

Supplement: Supplementary file 5 — Source data Fig. 3 [file 44318_2024_220_MOESM5_ESM.zip › Figure3/3F/RSA-400 (3).jpg]

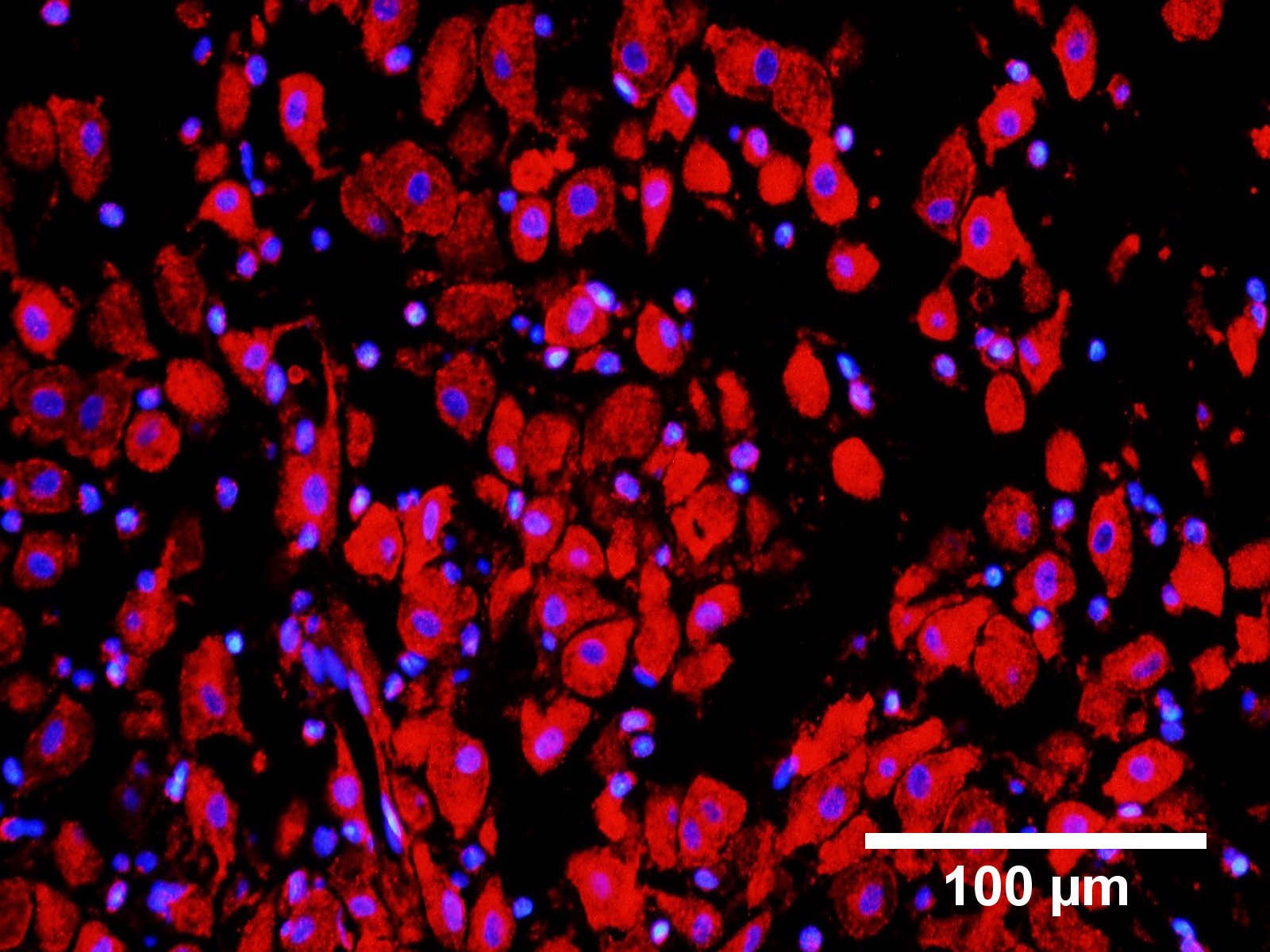

Supplement: Supplementary file 5 — Source data Fig. 3 [file 44318_2024_220_MOESM5_ESM.zip › Figure3/3F/RSA-400 (4).jpg]

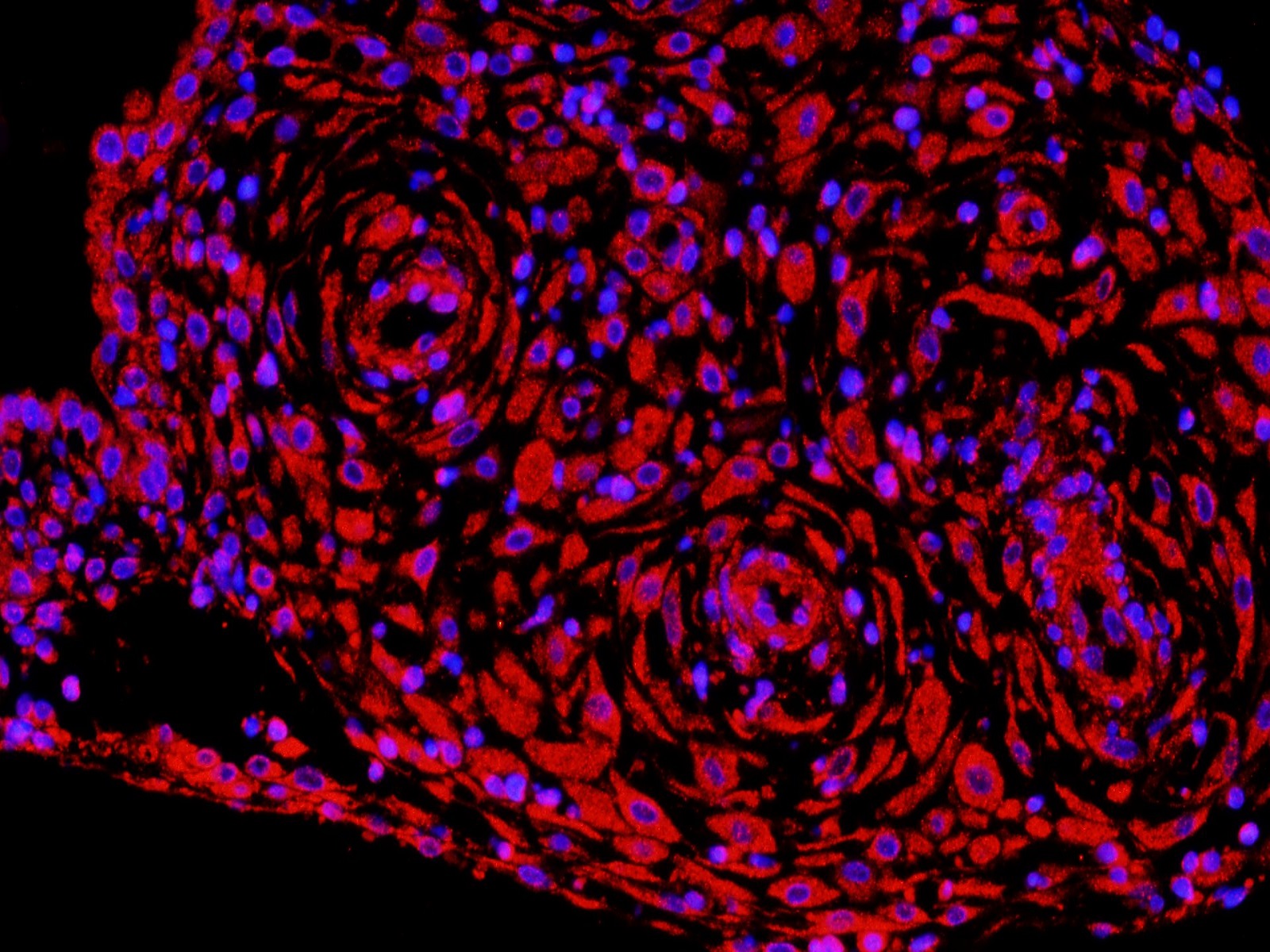

Supplement: Supplementary file 5 — Source data Fig. 3 [file 44318_2024_220_MOESM5_ESM.zip › Figure3/3F/RSA-400 (5).jpg]

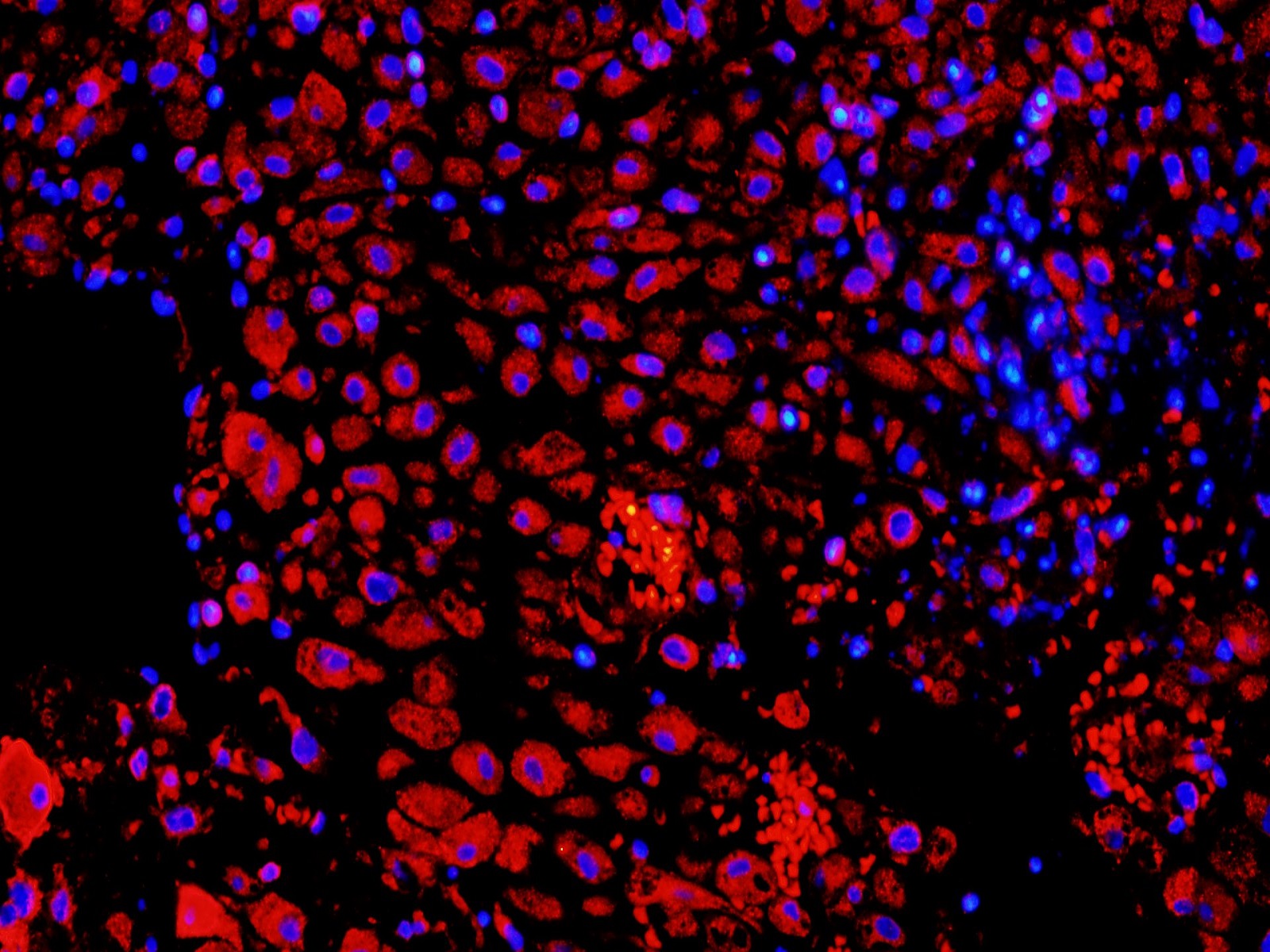

Supplement: Supplementary file 5 — Source data Fig. 3 [file 44318_2024_220_MOESM5_ESM.zip › Figure3/3F/RSA-400 (6).jpg]

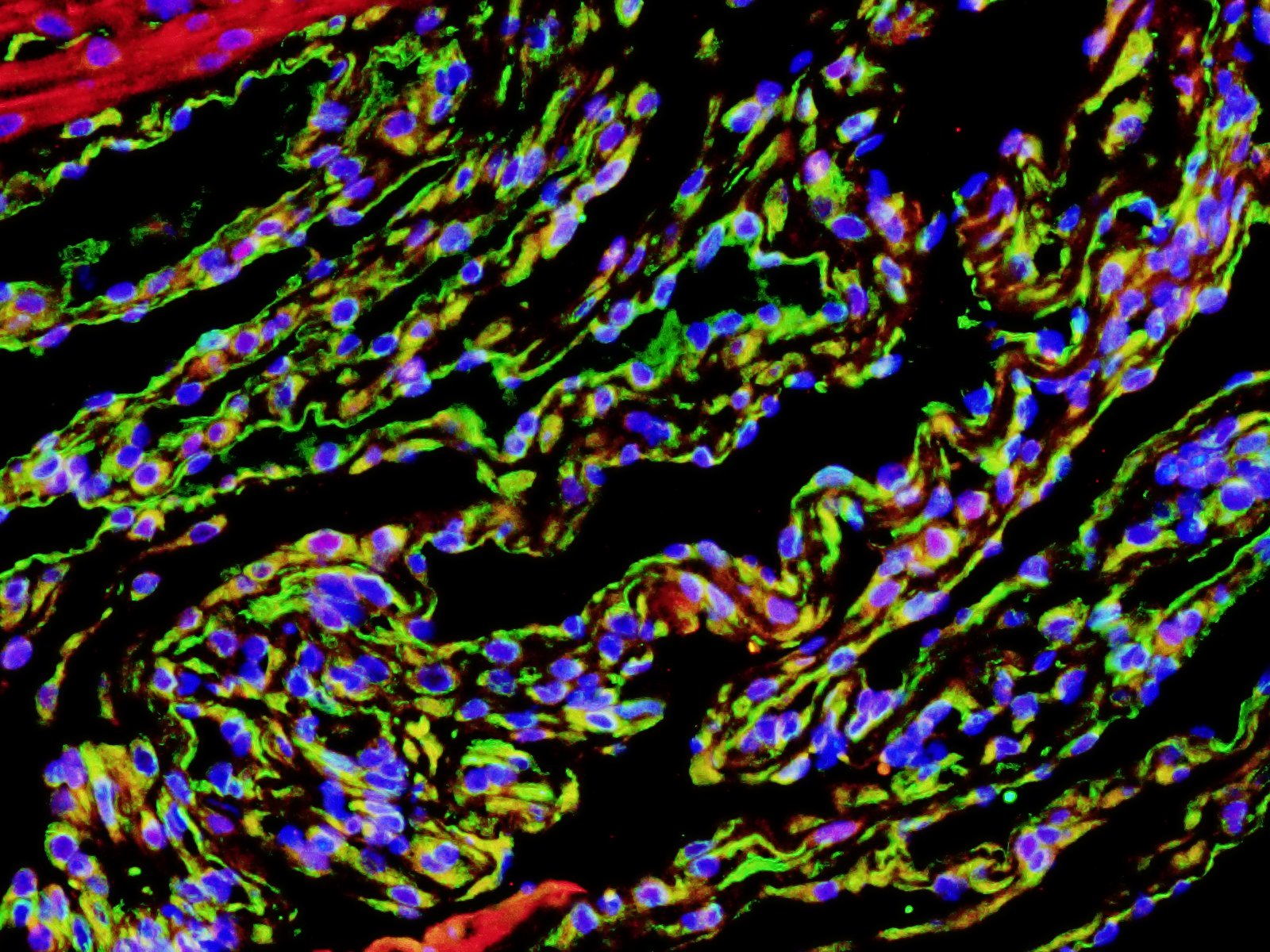

Supplement: Supplementary file 6 — Source data Fig. 4 [file 44318_2024_220_MOESM6_ESM.zip › Figure4/4B/CDKN1A-400-6%Leu-1.jpg]

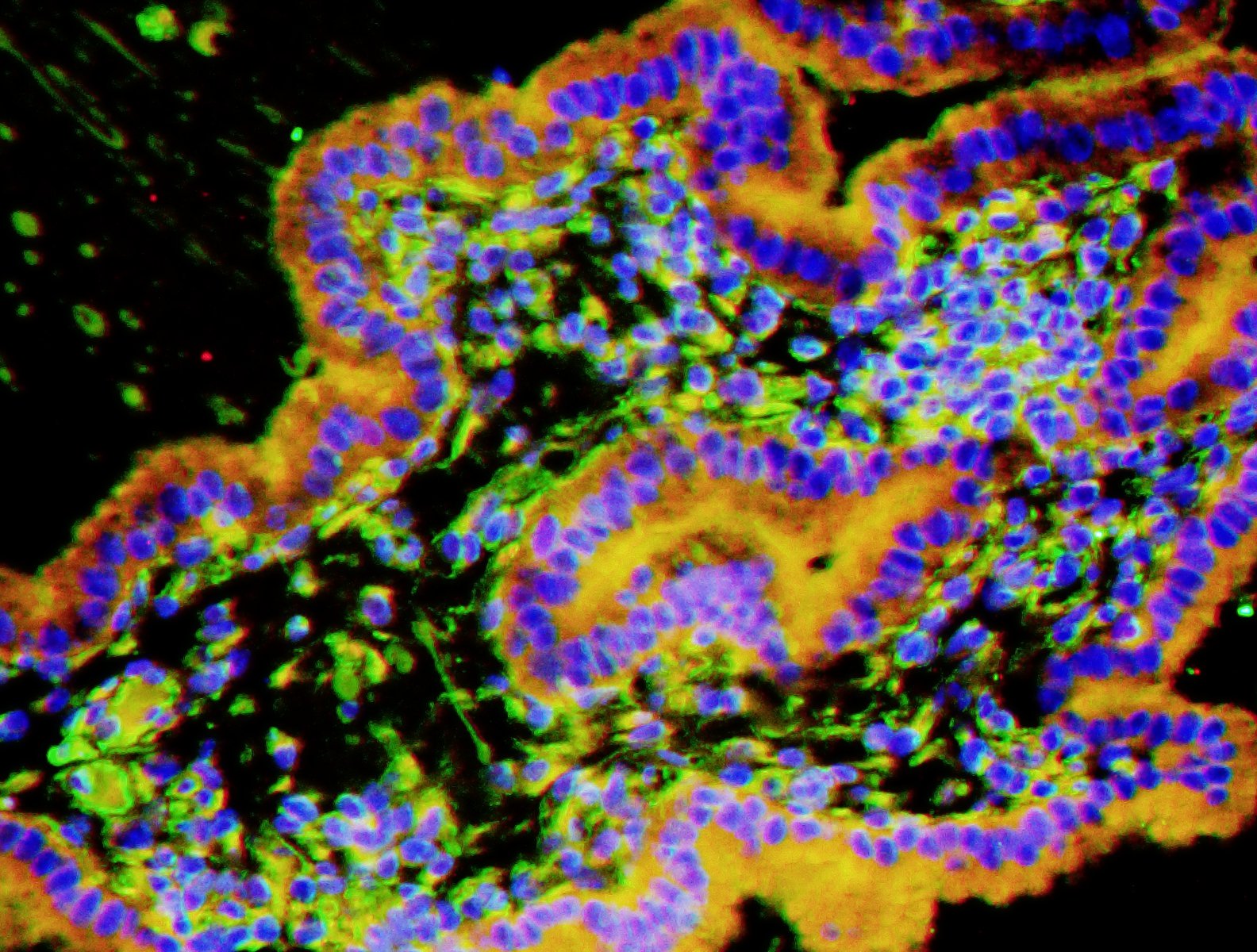

Supplement: Supplementary file 6 — Source data Fig. 4 [file 44318_2024_220_MOESM6_ESM.zip › Figure4/4B/CDKN1A-400-6%Leu-2.jpg]

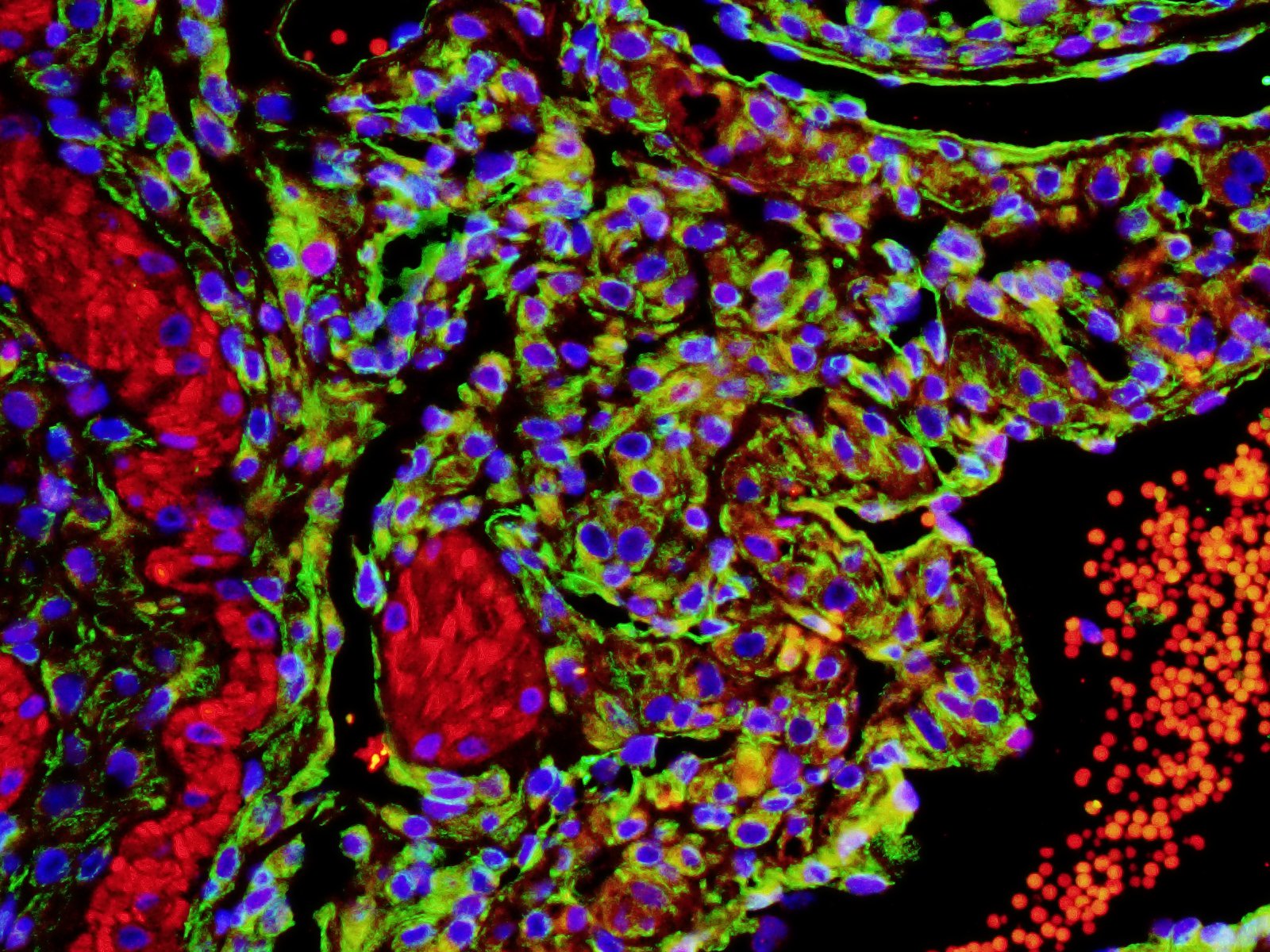

Supplement: Supplementary file 6 — Source data Fig. 4 [file 44318_2024_220_MOESM6_ESM.zip › Figure4/4B/CDKN1A-400-6%Leu-3.jpg]

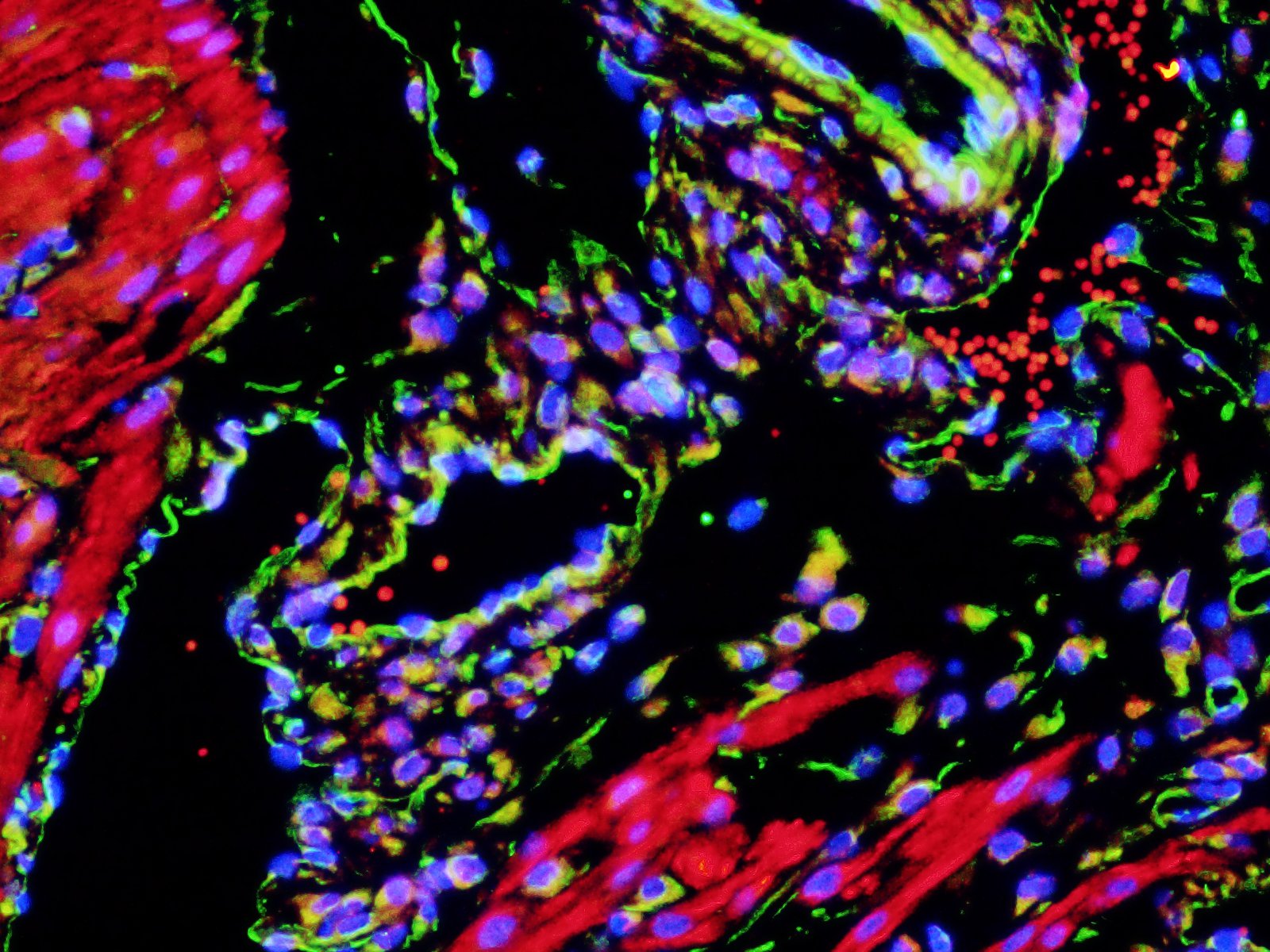

Supplement: Supplementary file 6 — Source data Fig. 4 [file 44318_2024_220_MOESM6_ESM.zip › Figure4/4B/CDKN1A-400-6%Leu-4.jpg]

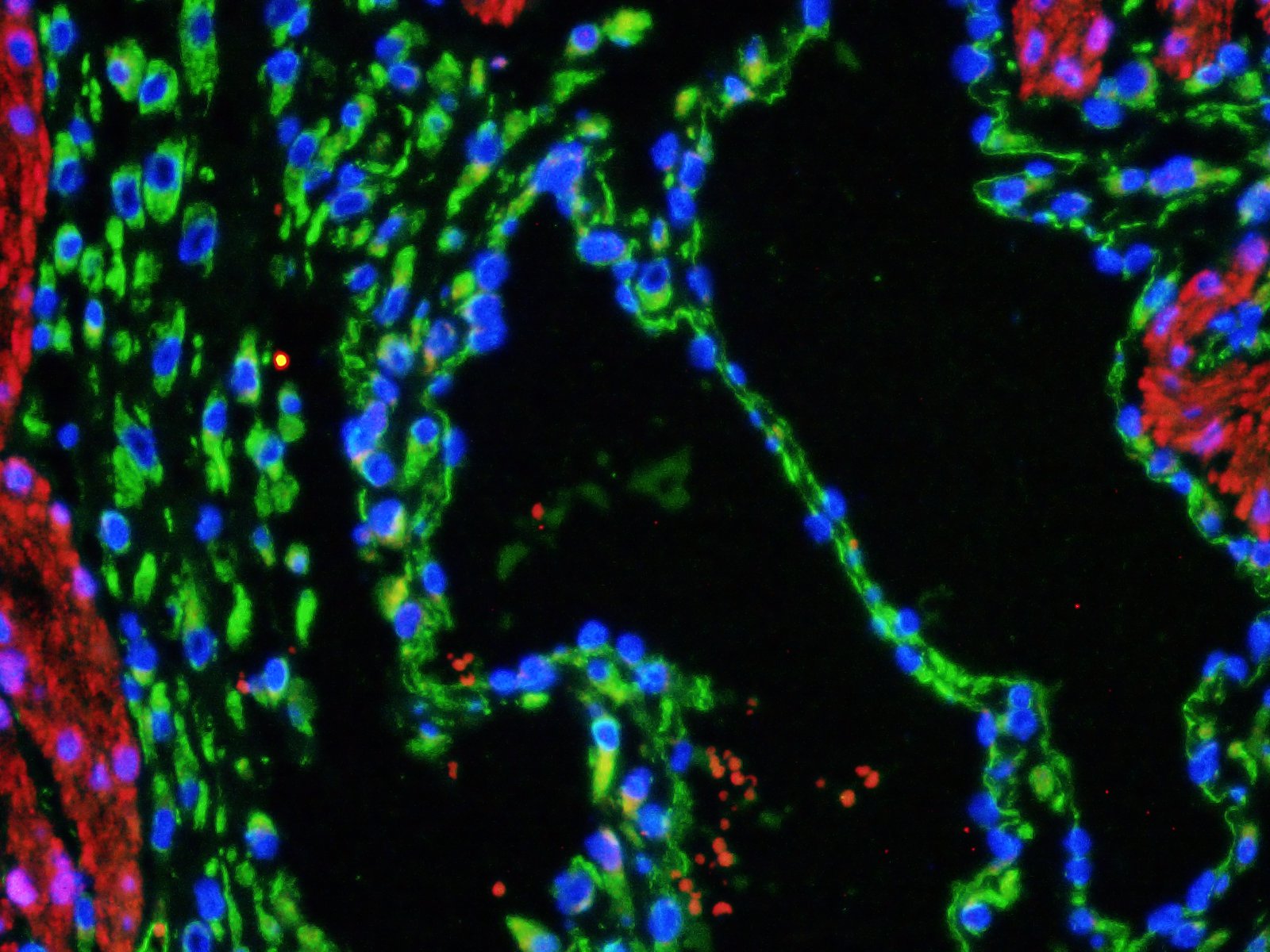

Supplement: Supplementary file 6 — Source data Fig. 4 [file 44318_2024_220_MOESM6_ESM.zip › Figure4/4B/CDKN1A-400-Ctrl1.jpg]

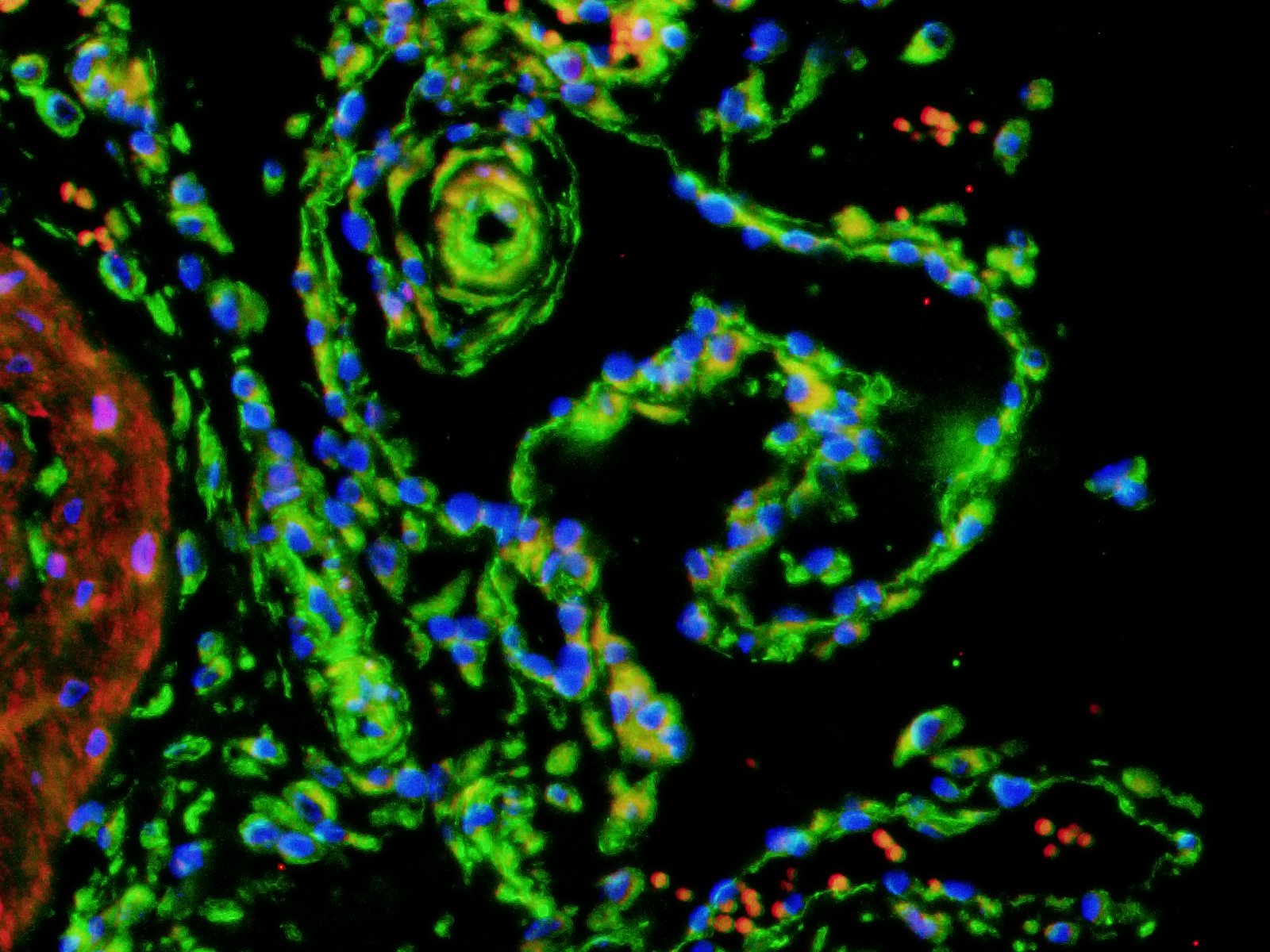

Supplement: Supplementary file 6 — Source data Fig. 4 [file 44318_2024_220_MOESM6_ESM.zip › Figure4/4B/CDKN1A-400-Ctrl2.jpg]

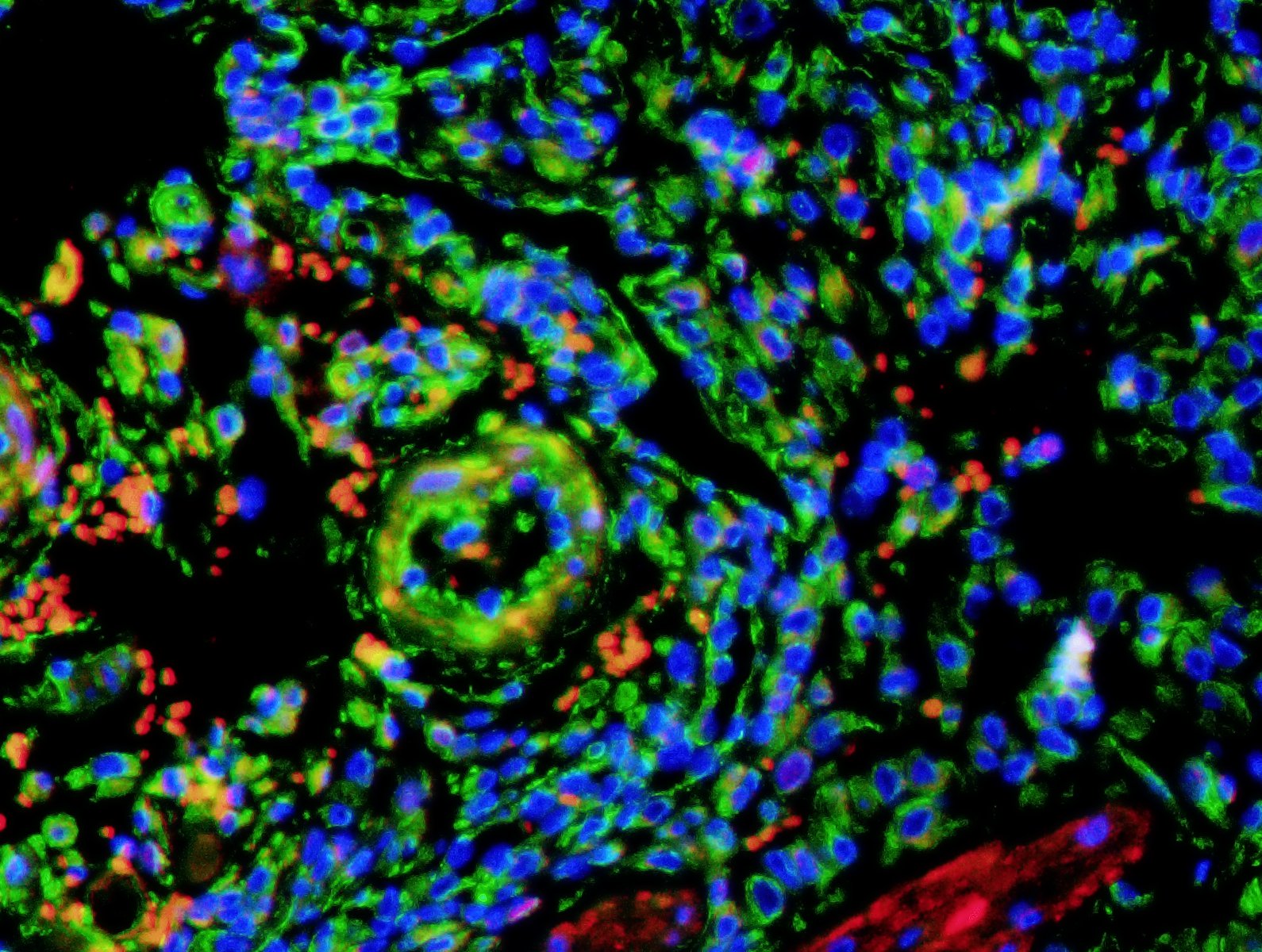

Supplement: Supplementary file 6 — Source data Fig. 4 [file 44318_2024_220_MOESM6_ESM.zip › Figure4/4B/CDKN1A-400-Ctrl3.jpg]

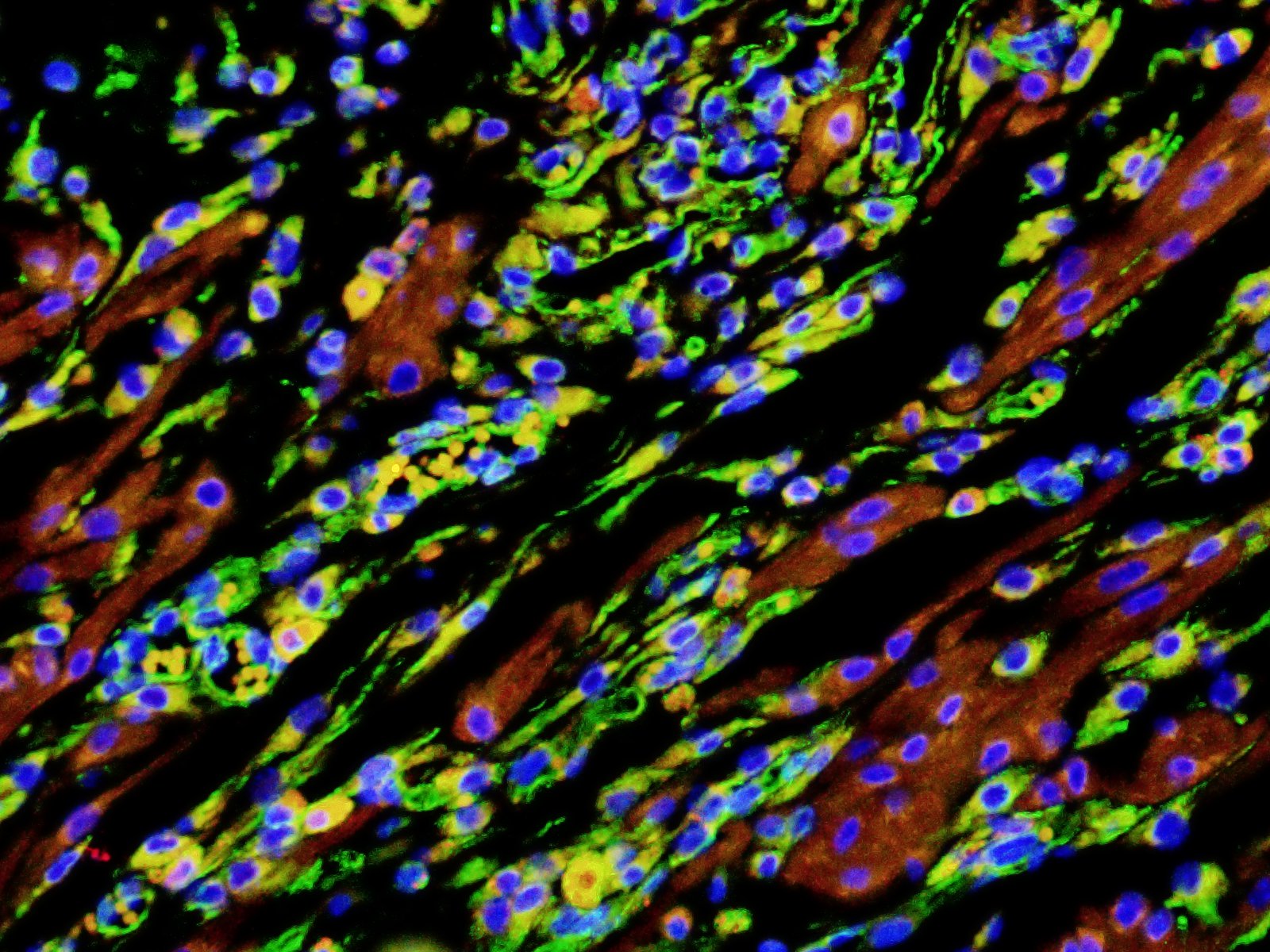

Supplement: Supplementary file 6 — Source data Fig. 4 [file 44318_2024_220_MOESM6_ESM.zip › Figure4/4B/CDKN2A-400-6%Leu-1.jpg]

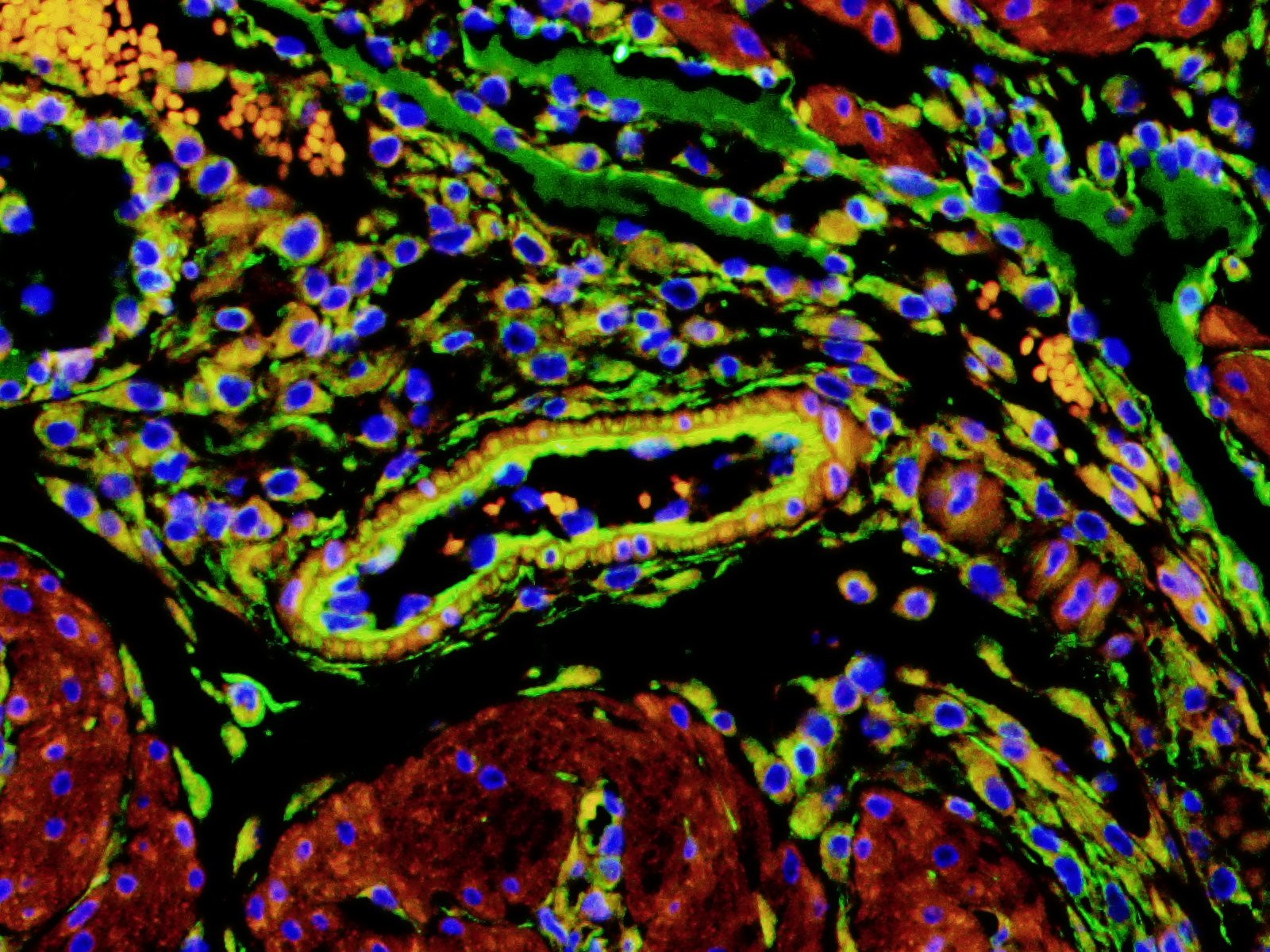

Supplement: Supplementary file 6 — Source data Fig. 4 [file 44318_2024_220_MOESM6_ESM.zip › Figure4/4B/CDKN2A-400-6%Leu-2.jpg]

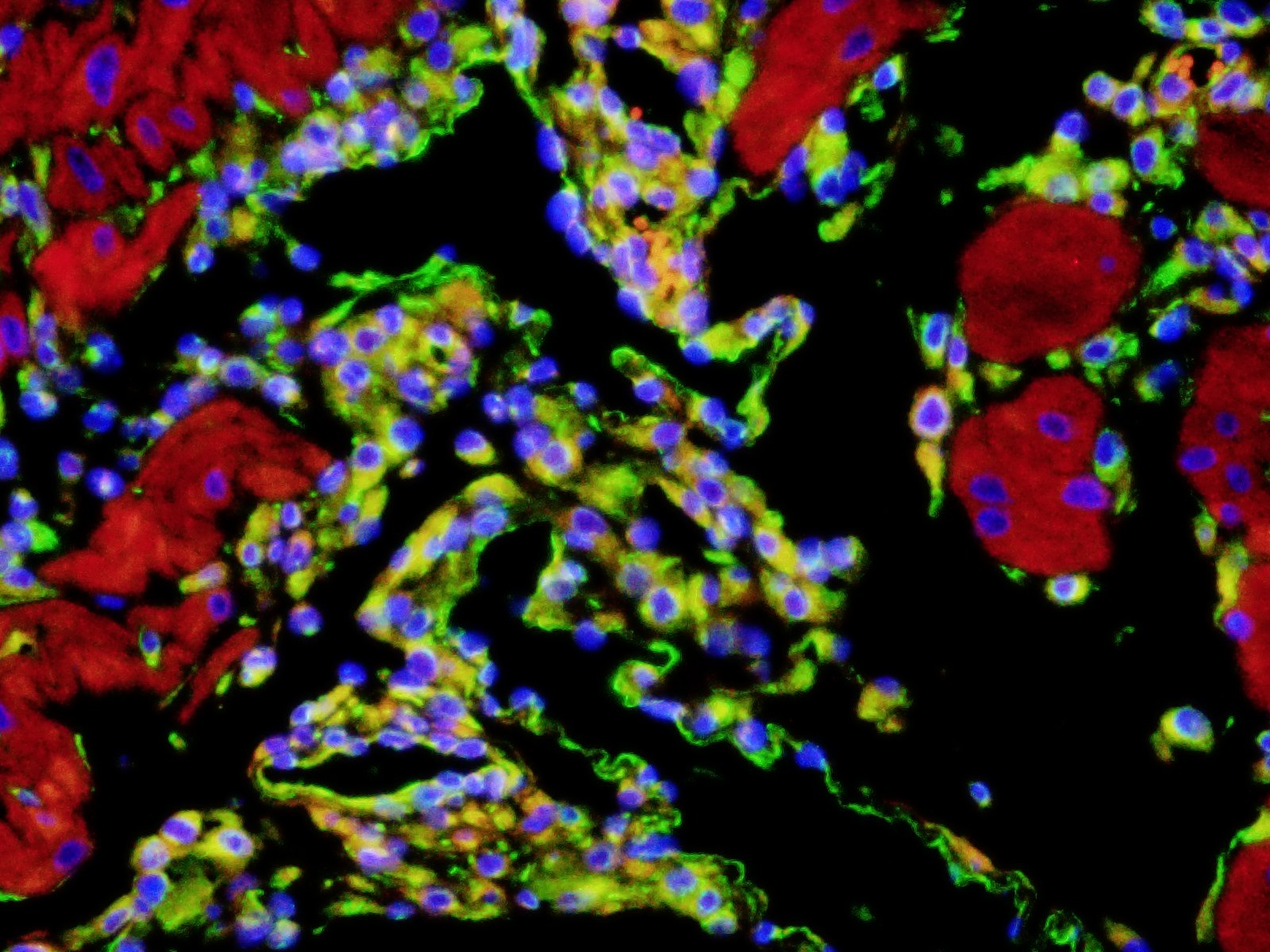

Supplement: Supplementary file 6 — Source data Fig. 4 [file 44318_2024_220_MOESM6_ESM.zip › Figure4/4B/CDKN2A-400-6%Leu-3.jpg]

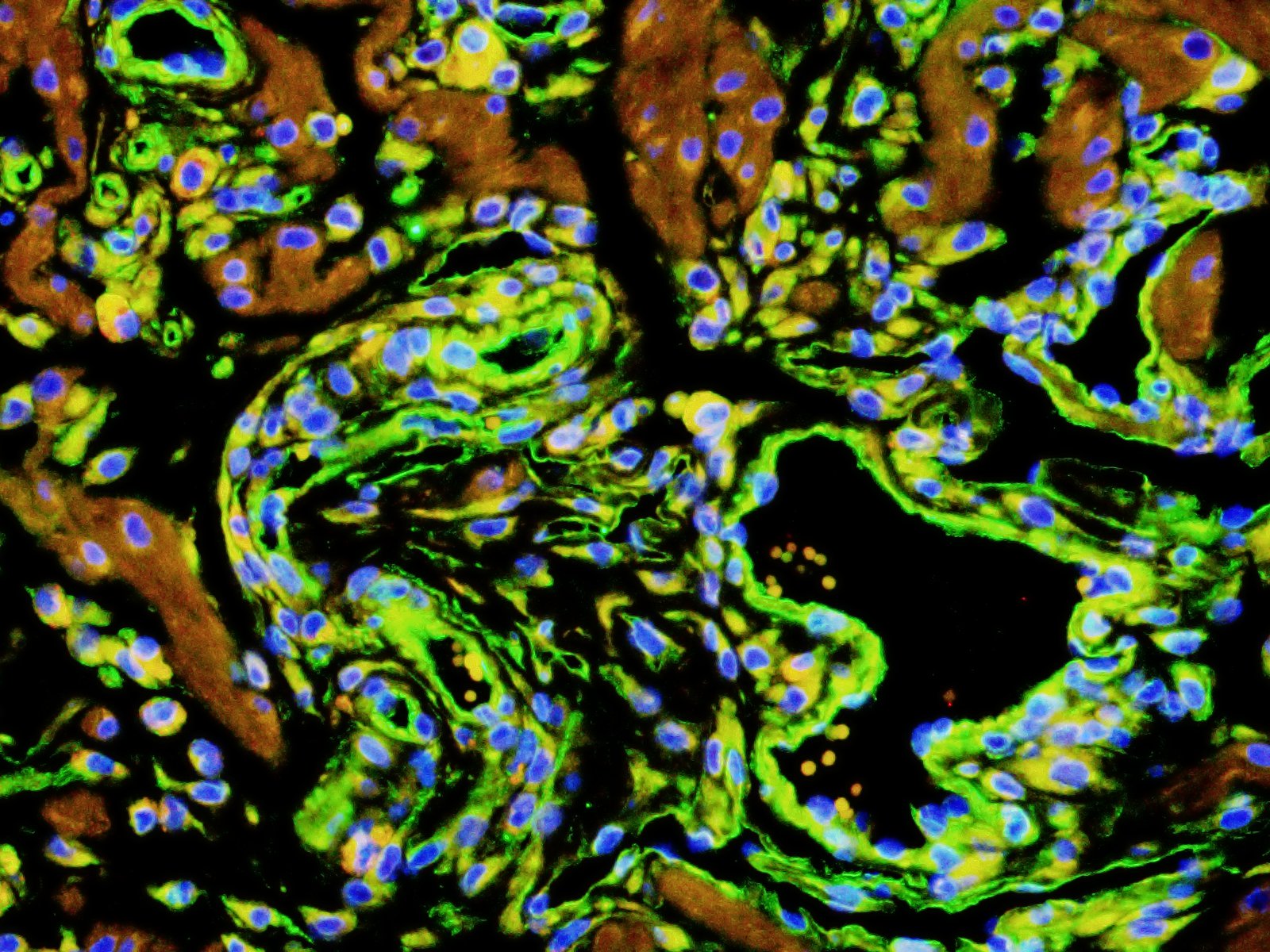

Supplement: Supplementary file 6 — Source data Fig. 4 [file 44318_2024_220_MOESM6_ESM.zip › Figure4/4B/CDKN2A-400-6%Leu-4.jpg]

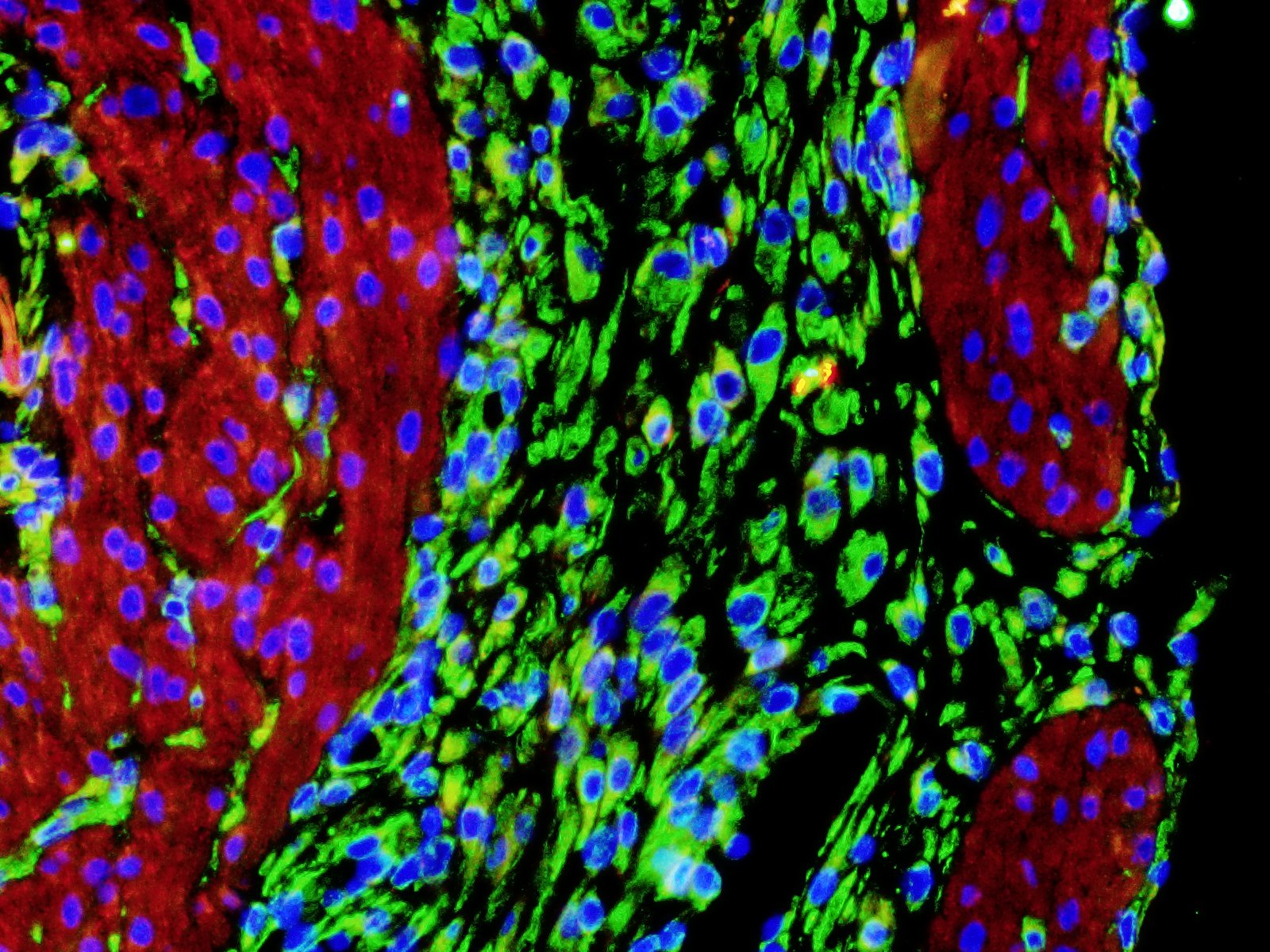

Supplement: Supplementary file 6 — Source data Fig. 4 [file 44318_2024_220_MOESM6_ESM.zip › Figure4/4B/CDKN2A-400-Ctrl1.jpg]

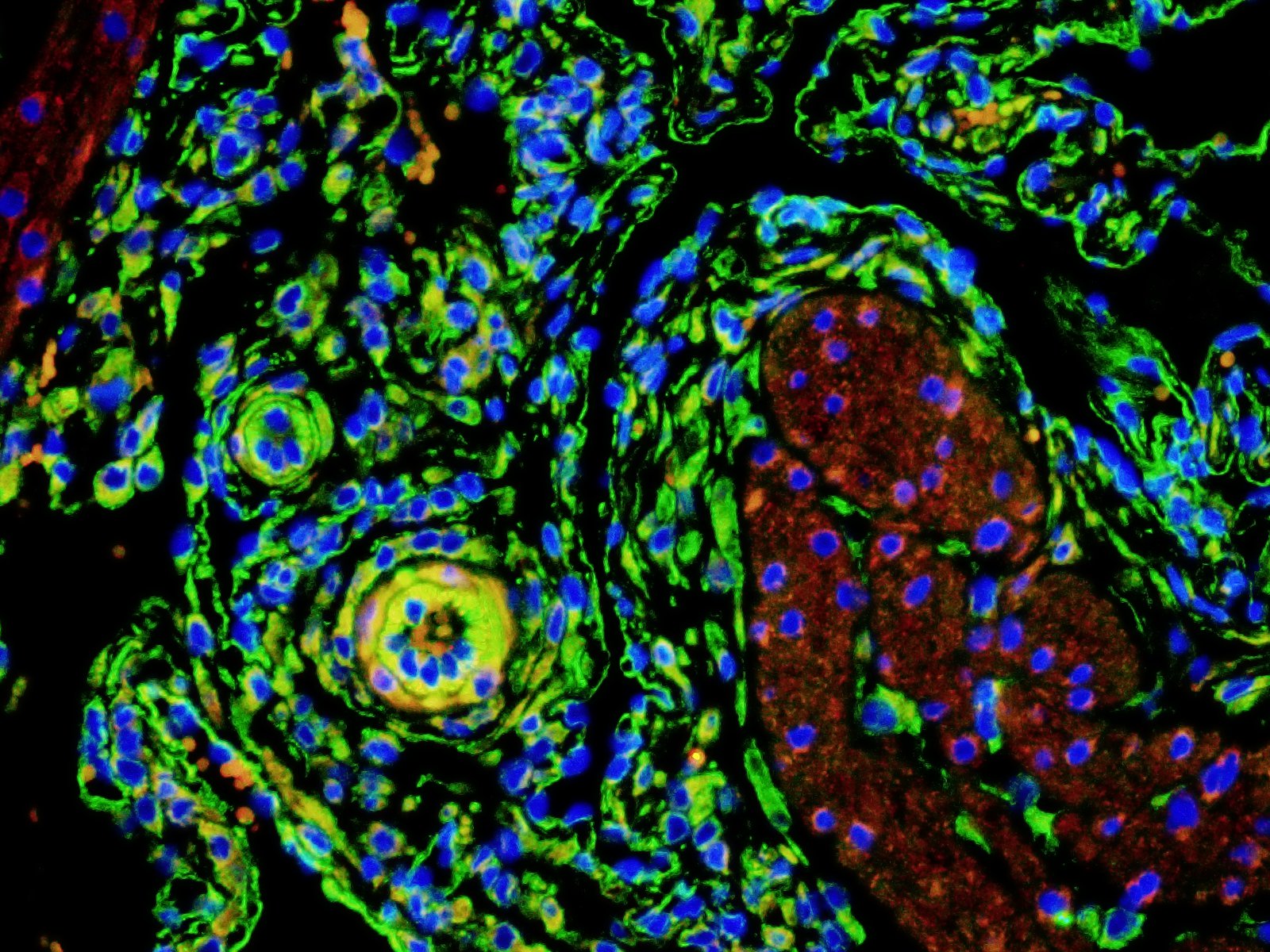

Supplement: Supplementary file 6 — Source data Fig. 4 [file 44318_2024_220_MOESM6_ESM.zip › Figure4/4B/CDKN2A-400-Ctrl2.jpg]

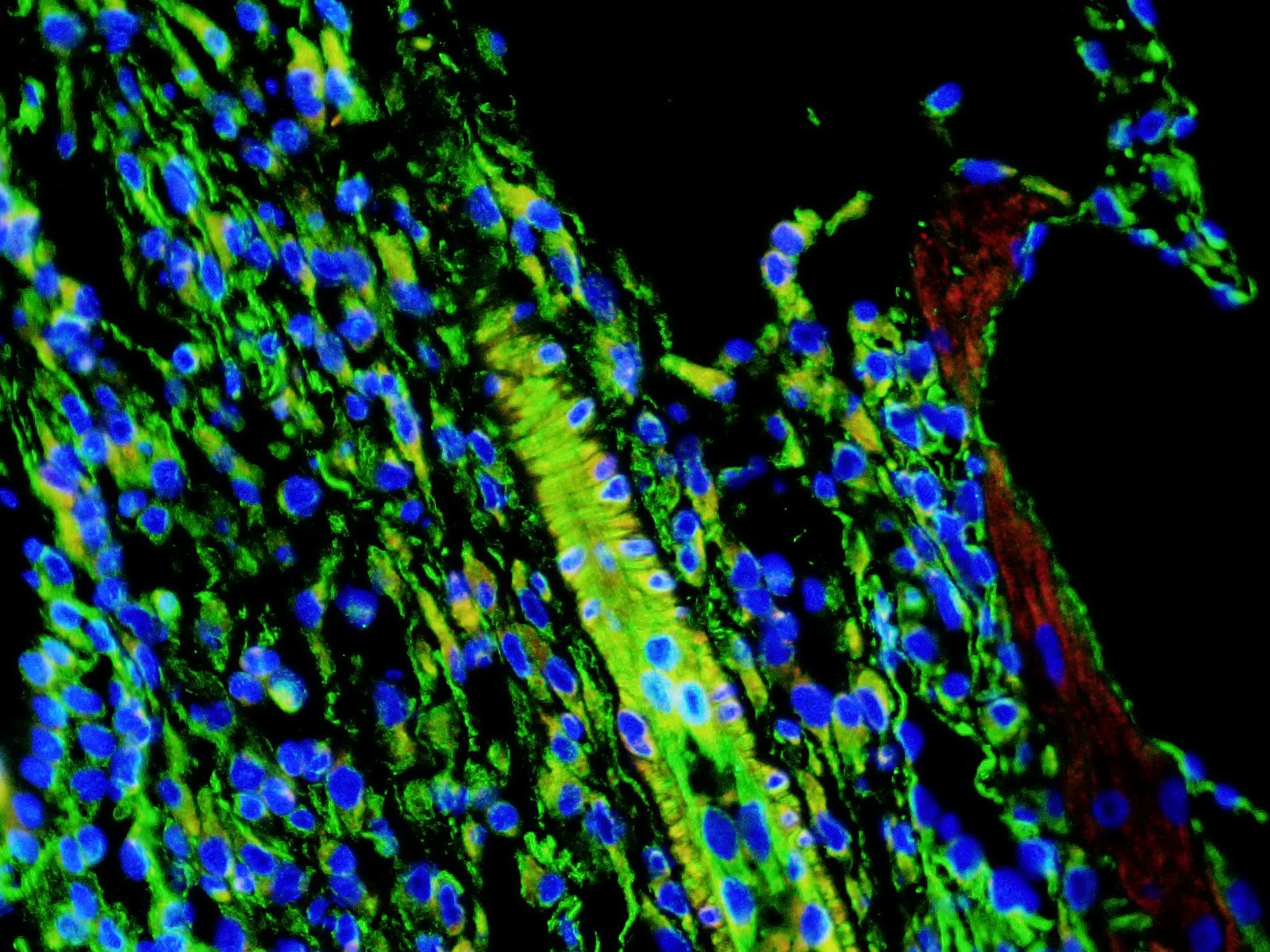

Supplement: Supplementary file 6 — Source data Fig. 4 [file 44318_2024_220_MOESM6_ESM.zip › Figure4/4B/CDKN2A-400-Ctrl3.jpg]

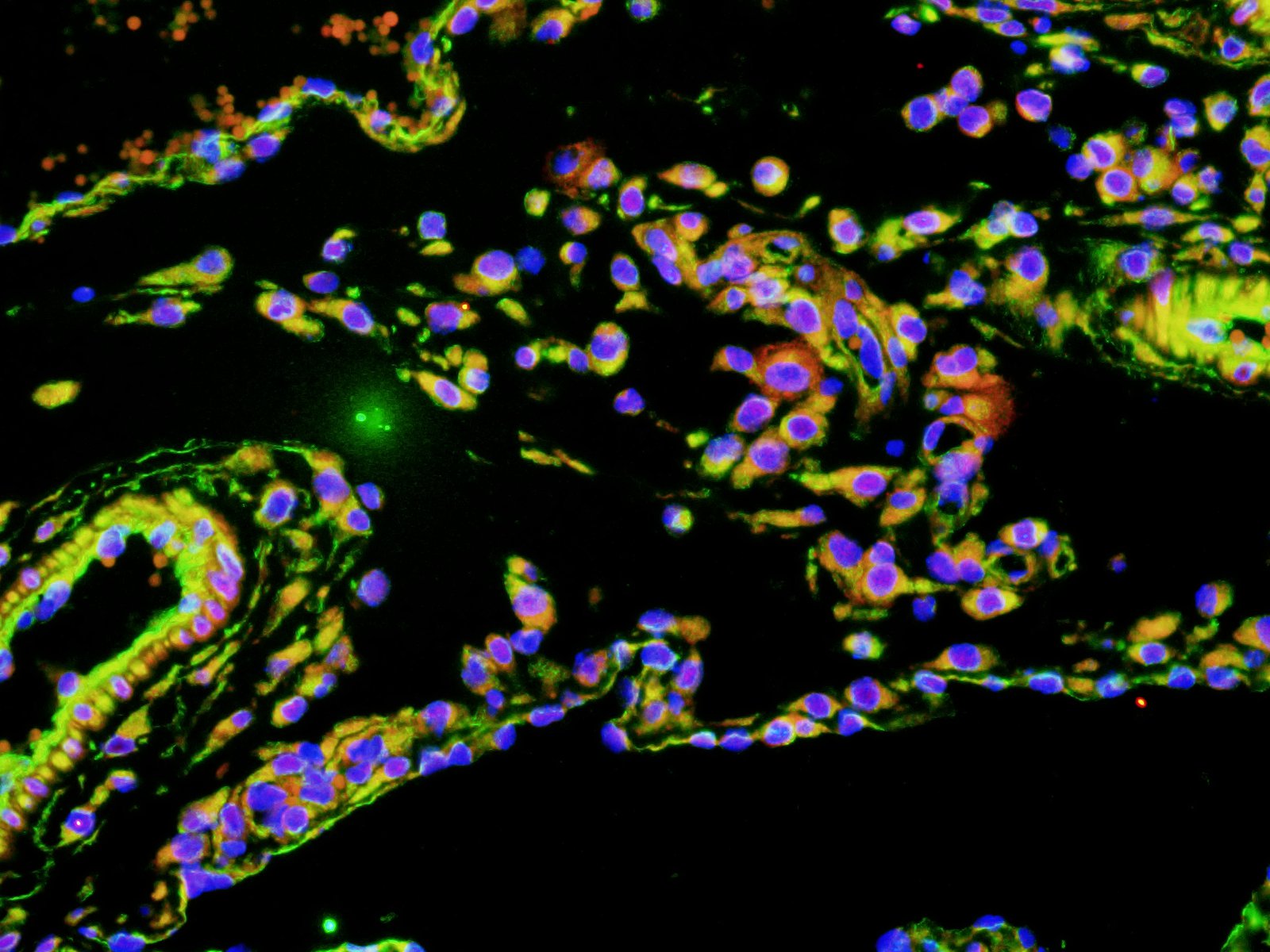

Supplement: Supplementary file 6 — Source data Fig. 4 [file 44318_2024_220_MOESM6_ESM.zip › Figure4/4B/TP53-400-6%Leu-1.jpg]

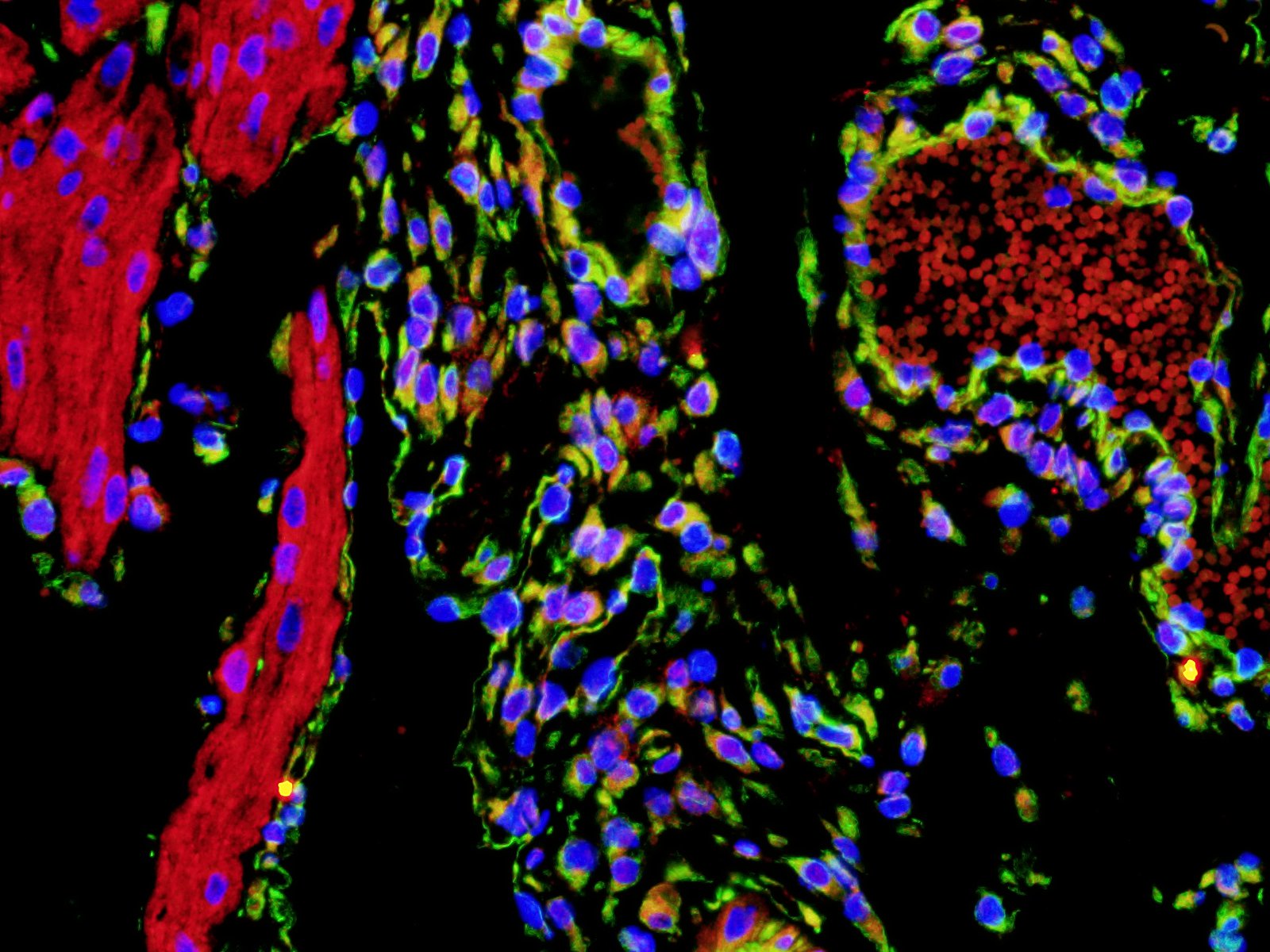

Supplement: Supplementary file 6 — Source data Fig. 4 [file 44318_2024_220_MOESM6_ESM.zip › Figure4/4B/TP53-400-6%Leu-2.jpg]

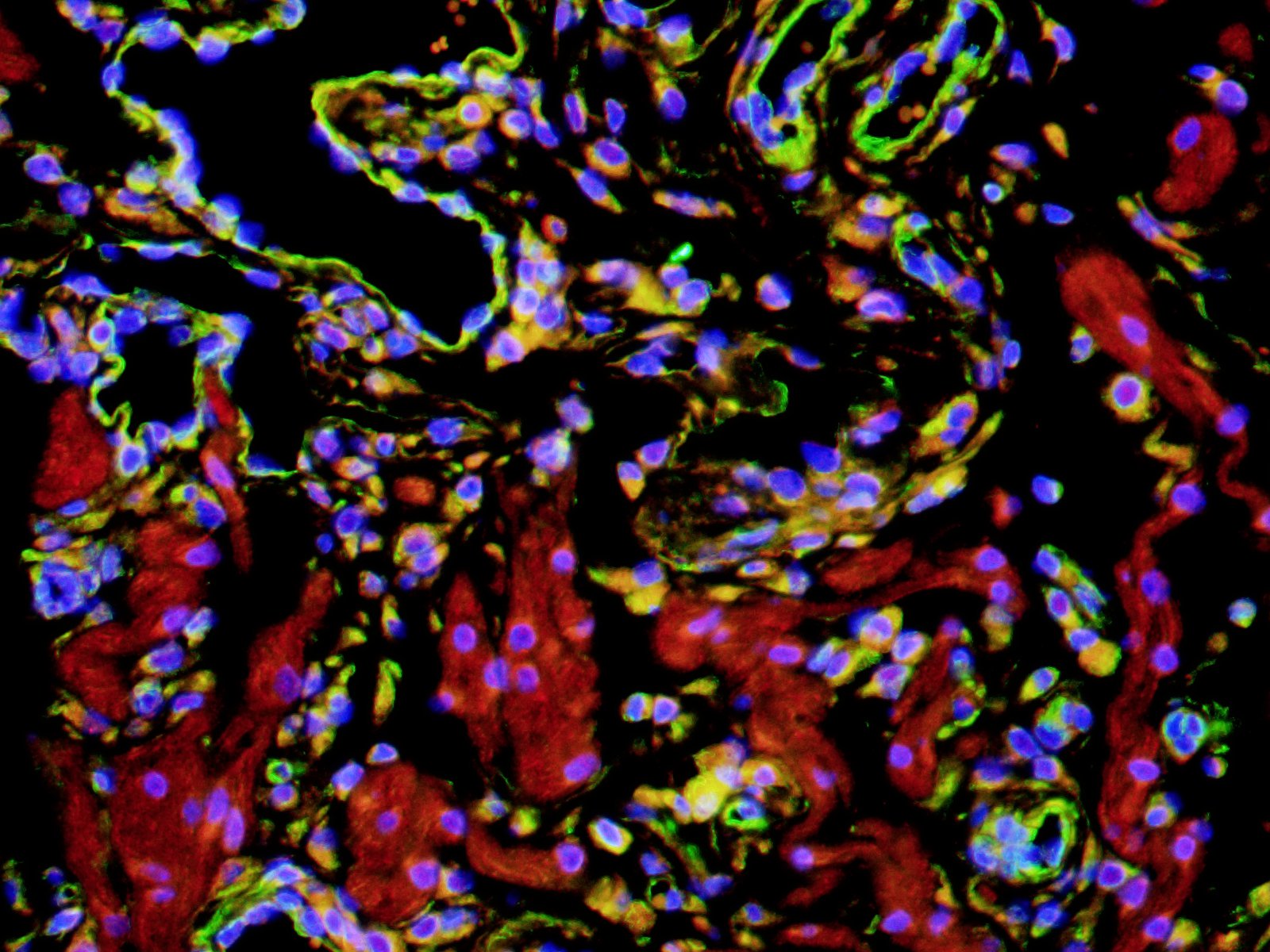

Supplement: Supplementary file 6 — Source data Fig. 4 [file 44318_2024_220_MOESM6_ESM.zip › Figure4/4B/TP53-400-6%Leu-3.jpg]

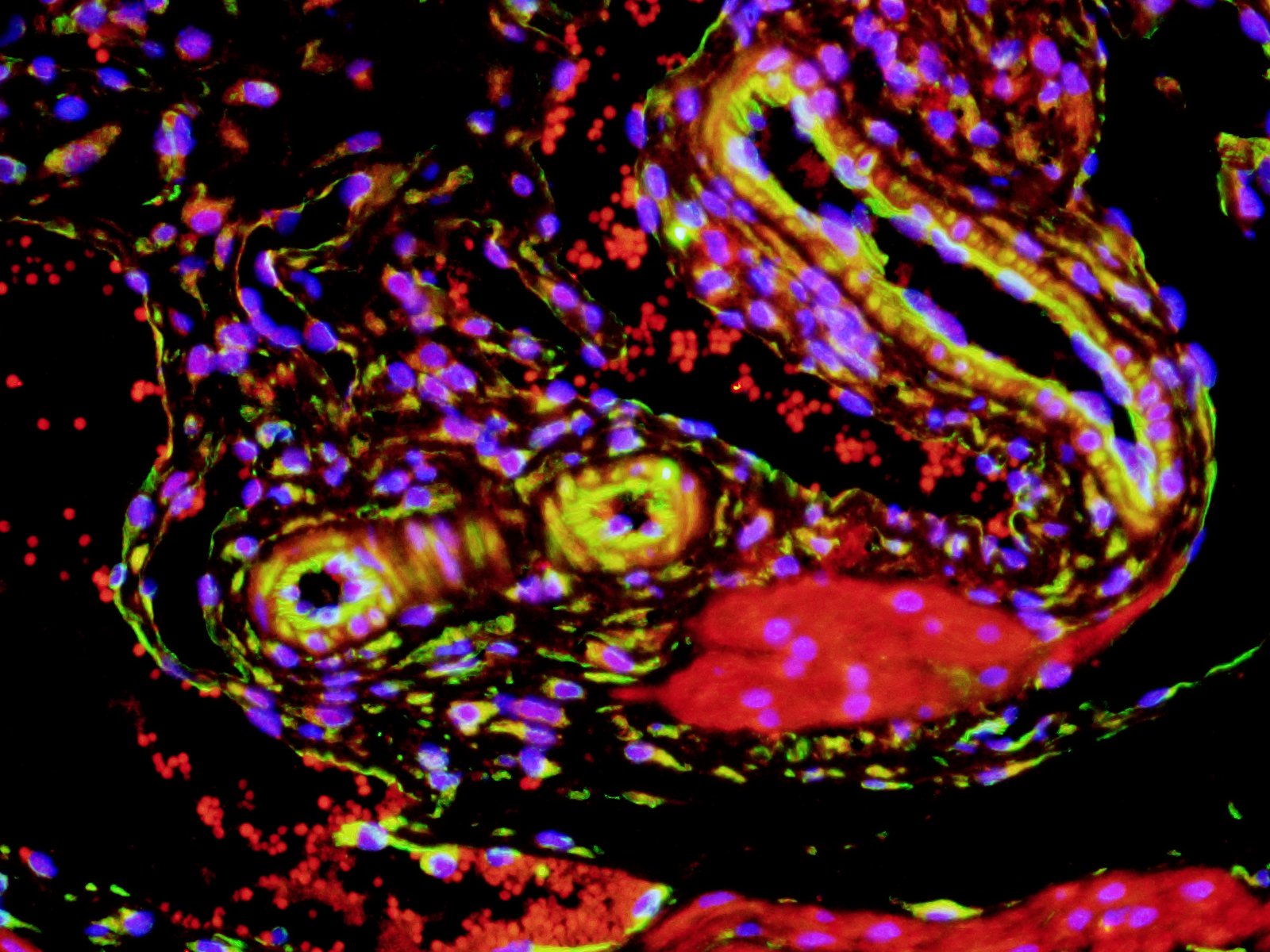

Supplement: Supplementary file 6 — Source data Fig. 4 [file 44318_2024_220_MOESM6_ESM.zip › Figure4/4B/TP53-400-6%Leu-4.jpg]

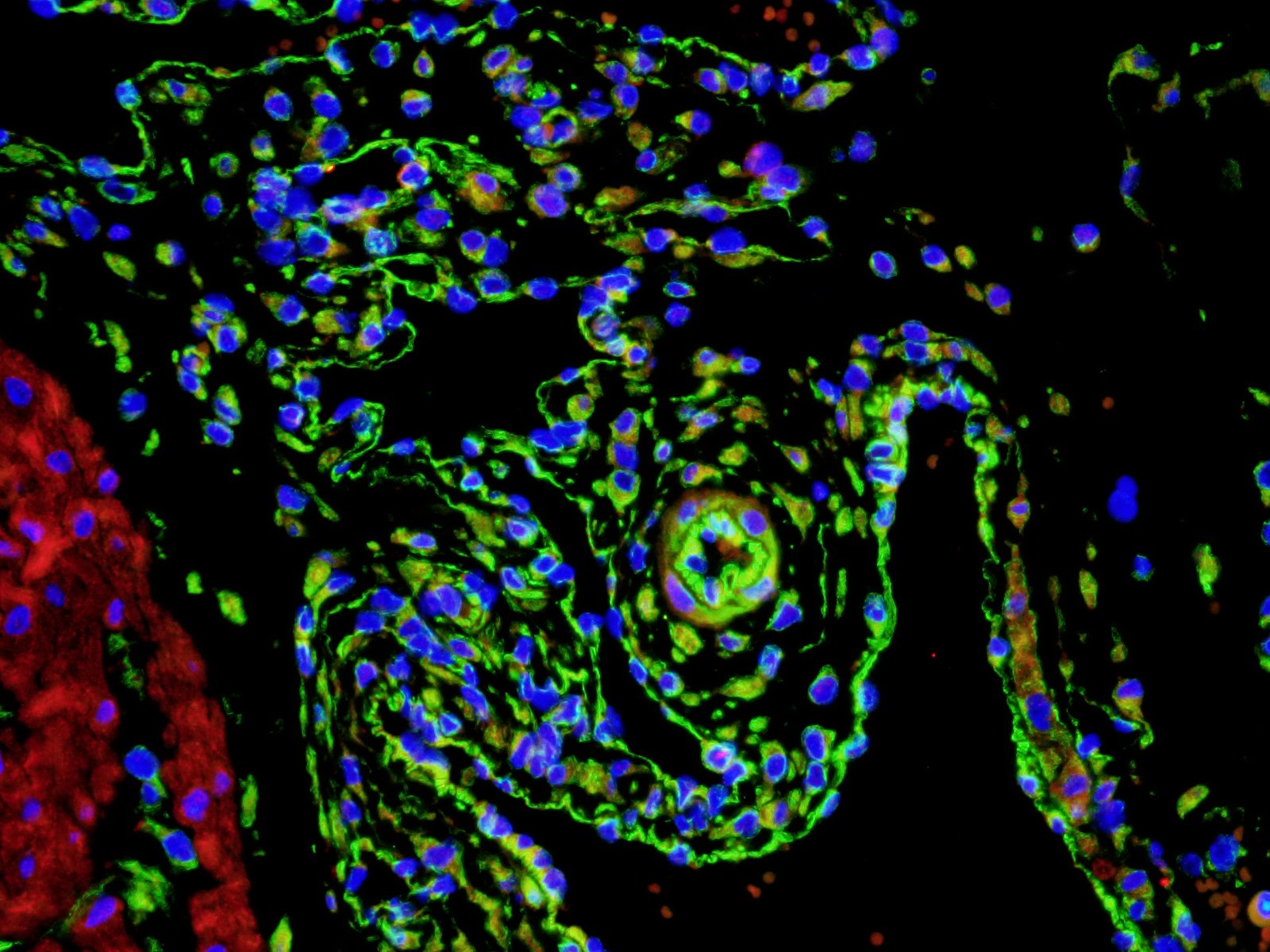

Supplement: Supplementary file 6 — Source data Fig. 4 [file 44318_2024_220_MOESM6_ESM.zip › Figure4/4B/TP53-400-Ctrl1.jpg]

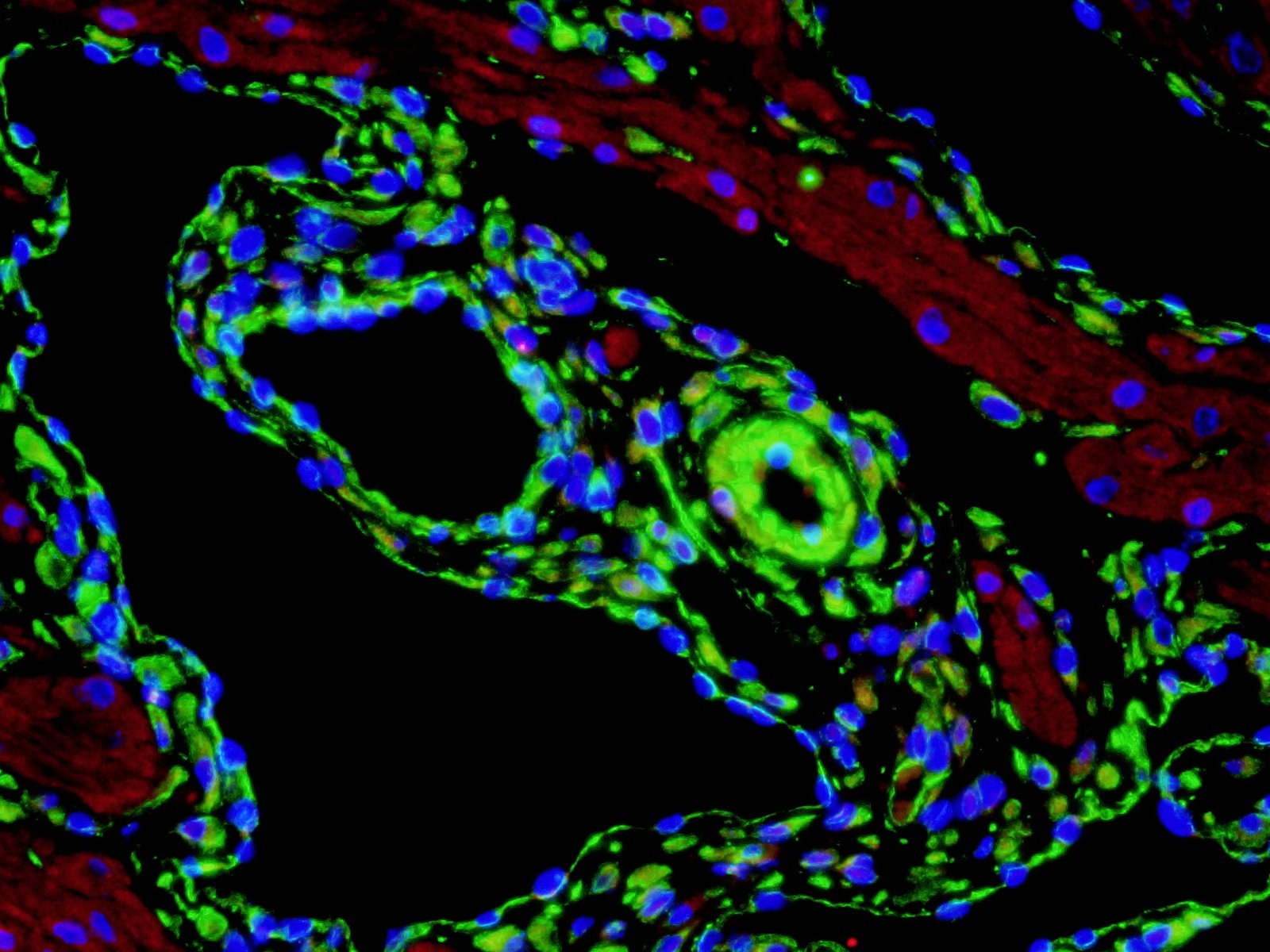

Supplement: Supplementary file 6 — Source data Fig. 4 [file 44318_2024_220_MOESM6_ESM.zip › Figure4/4B/TP53-400-Ctrl2.jpg]

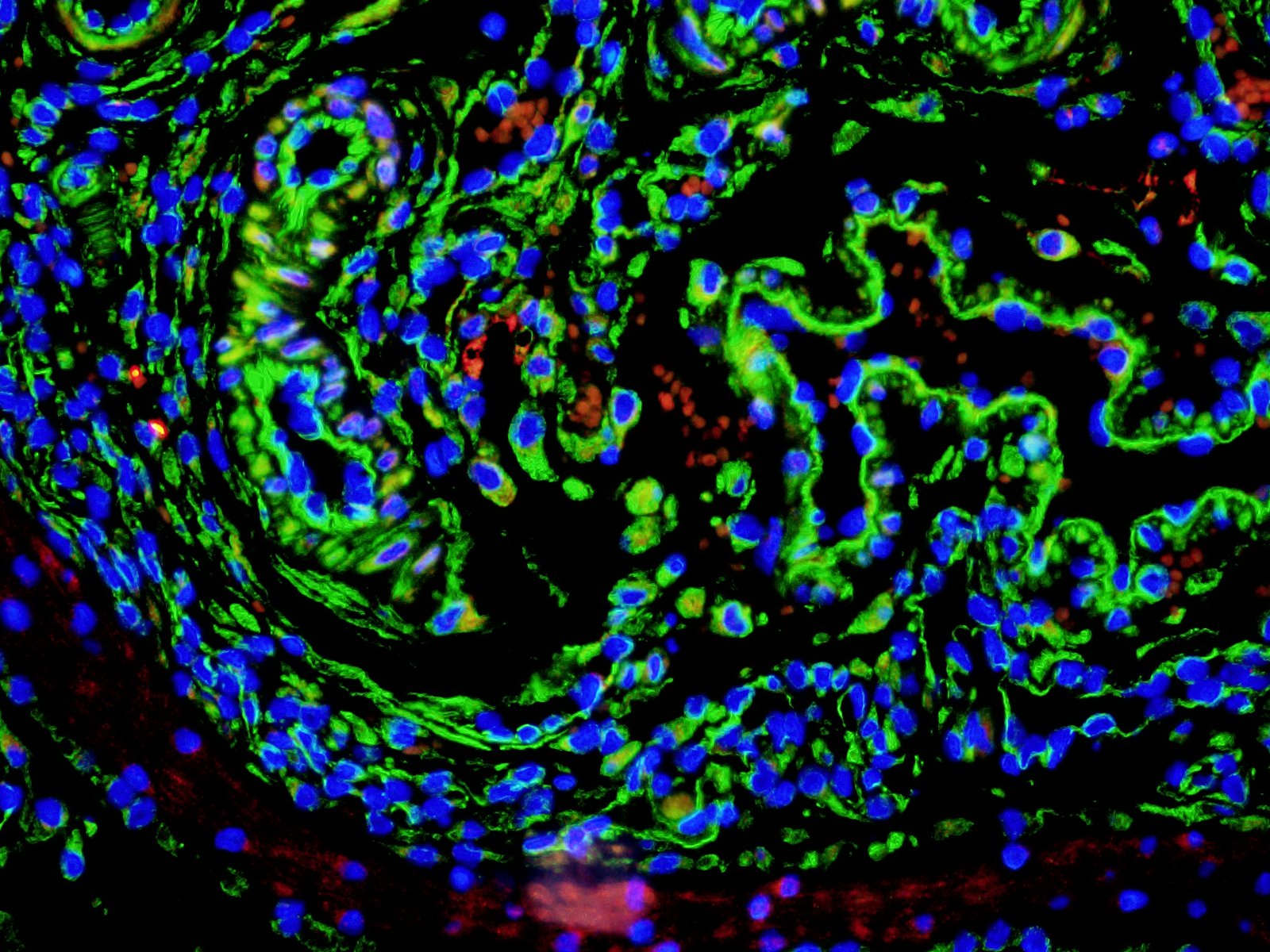

Supplement: Supplementary file 6 — Source data Fig. 4 [file 44318_2024_220_MOESM6_ESM.zip › Figure4/4B/TP53-400-Ctrl3.jpg]

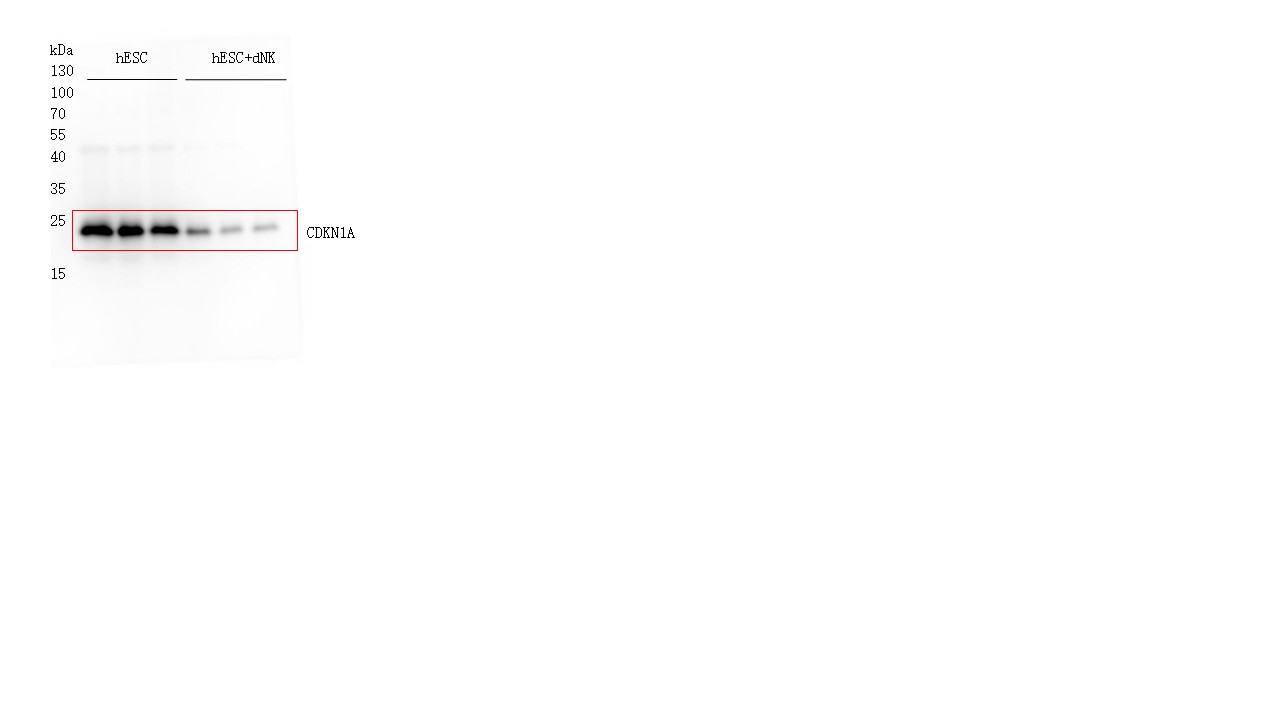

Supplement: Supplementary file 6 — Source data Fig. 4 [file 44318_2024_220_MOESM6_ESM.zip › Figure4/4E/western CDKN1A.jpg]

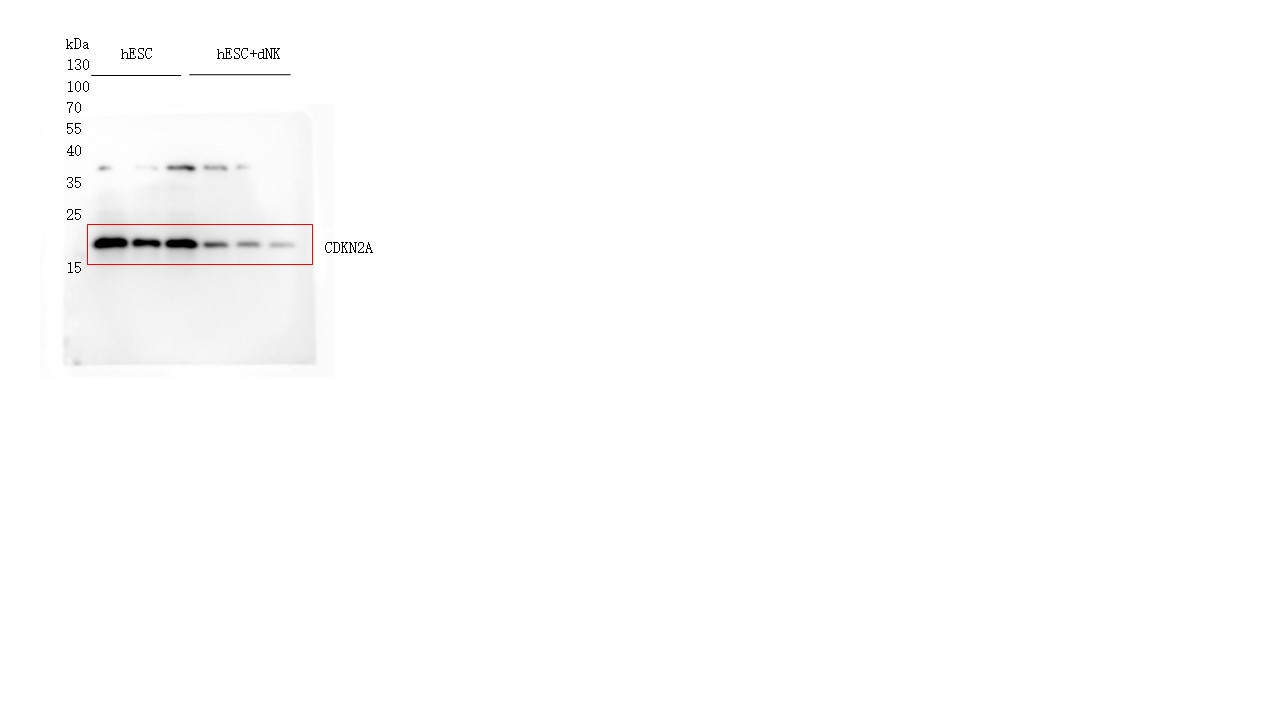

Supplement: Supplementary file 6 — Source data Fig. 4 [file 44318_2024_220_MOESM6_ESM.zip › Figure4/4E/western CDKN2A.jpg]

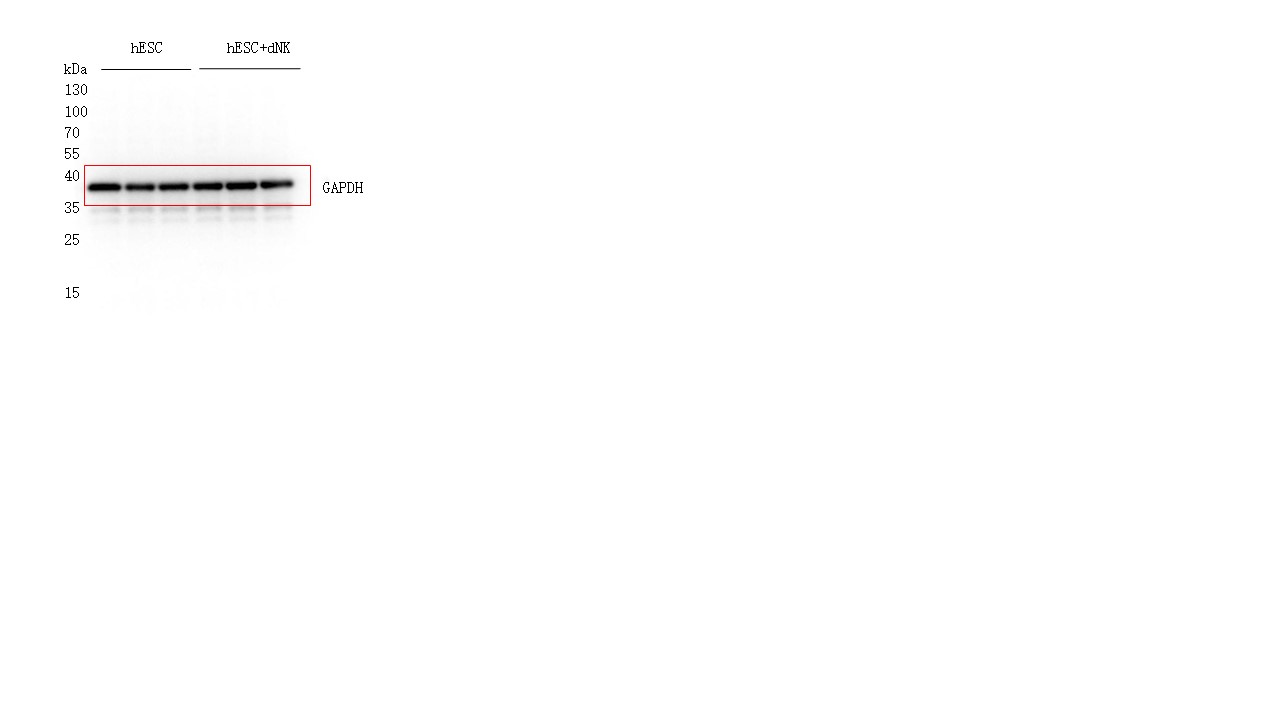

Supplement: Supplementary file 6 — Source data Fig. 4 [file 44318_2024_220_MOESM6_ESM.zip › Figure4/4E/western GAPDH.jpg]

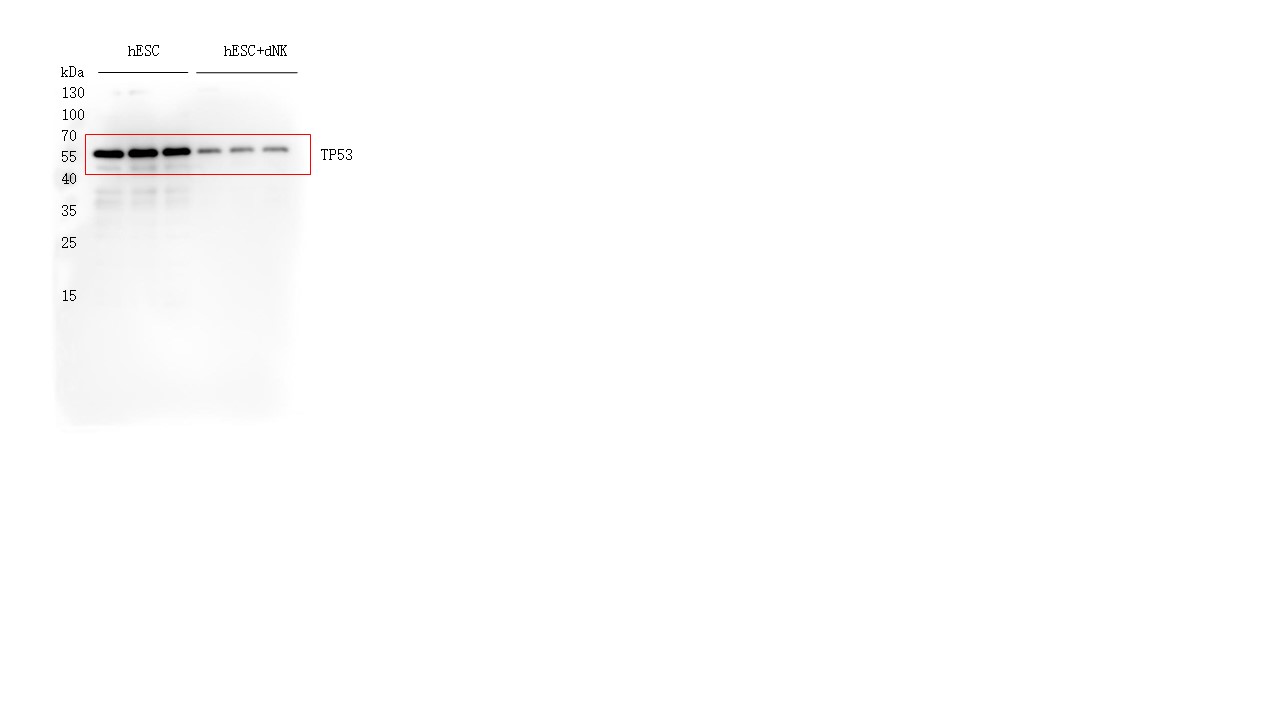

Supplement: Supplementary file 6 — Source data Fig. 4 [file 44318_2024_220_MOESM6_ESM.zip › Figure4/4E/western TP53.jpg]

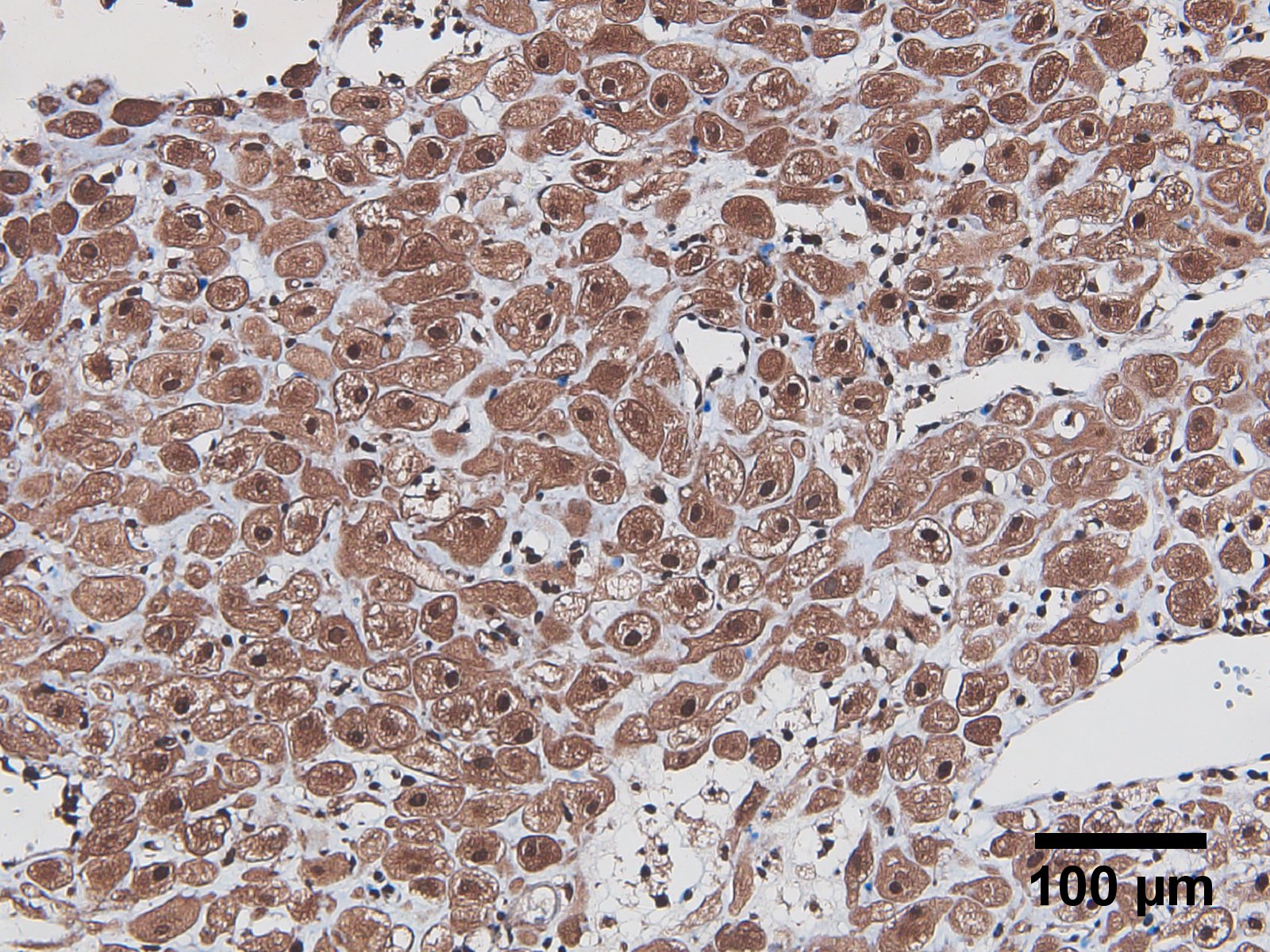

Supplement: Supplementary file 6 — Source data Fig. 4 [file 44318_2024_220_MOESM6_ESM.zip › Figure4/4G/NP-200-1.jpg]

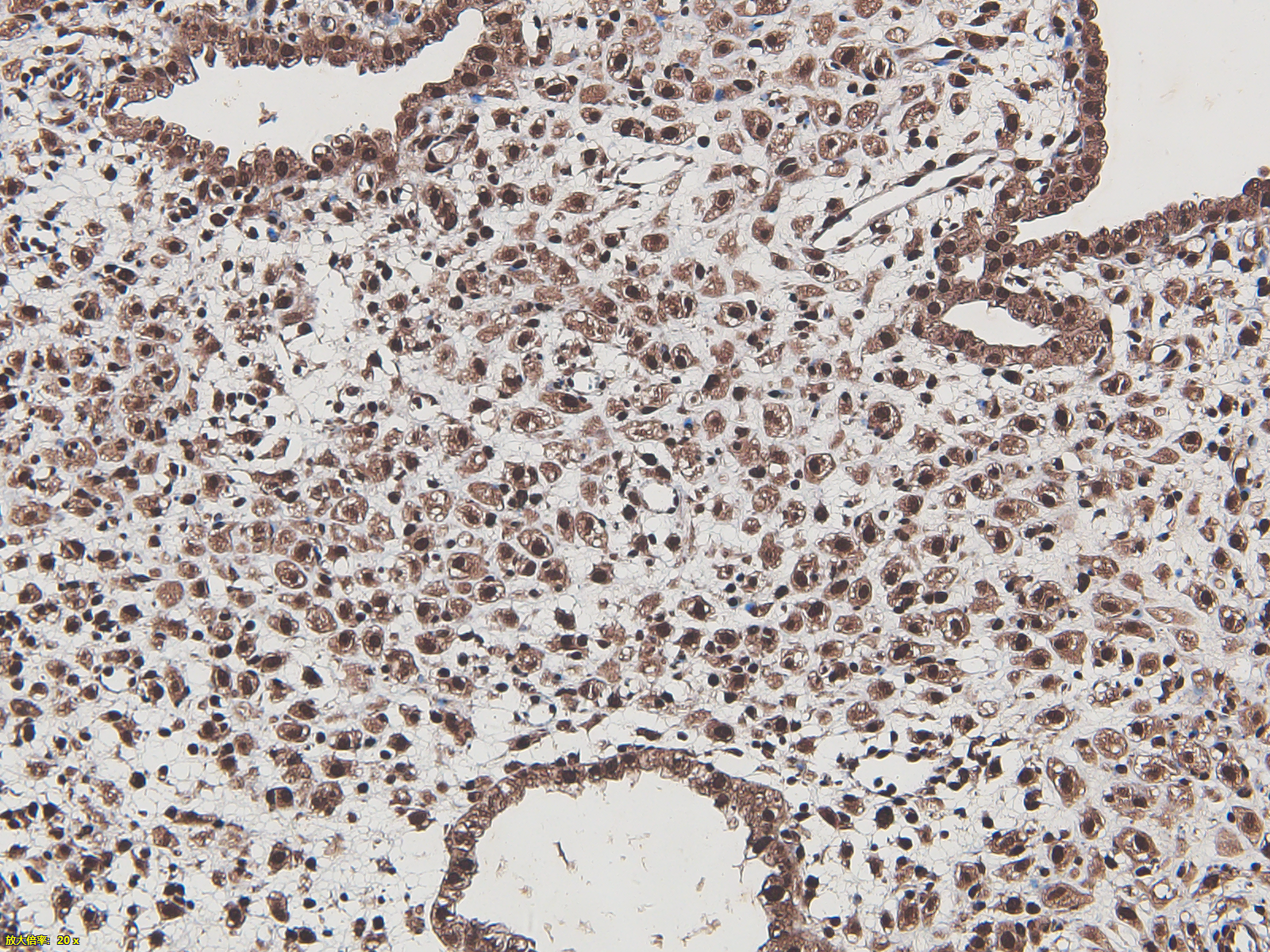

Supplement: Supplementary file 6 — Source data Fig. 4 [file 44318_2024_220_MOESM6_ESM.zip › Figure4/4G/NP-200-2.bmp]

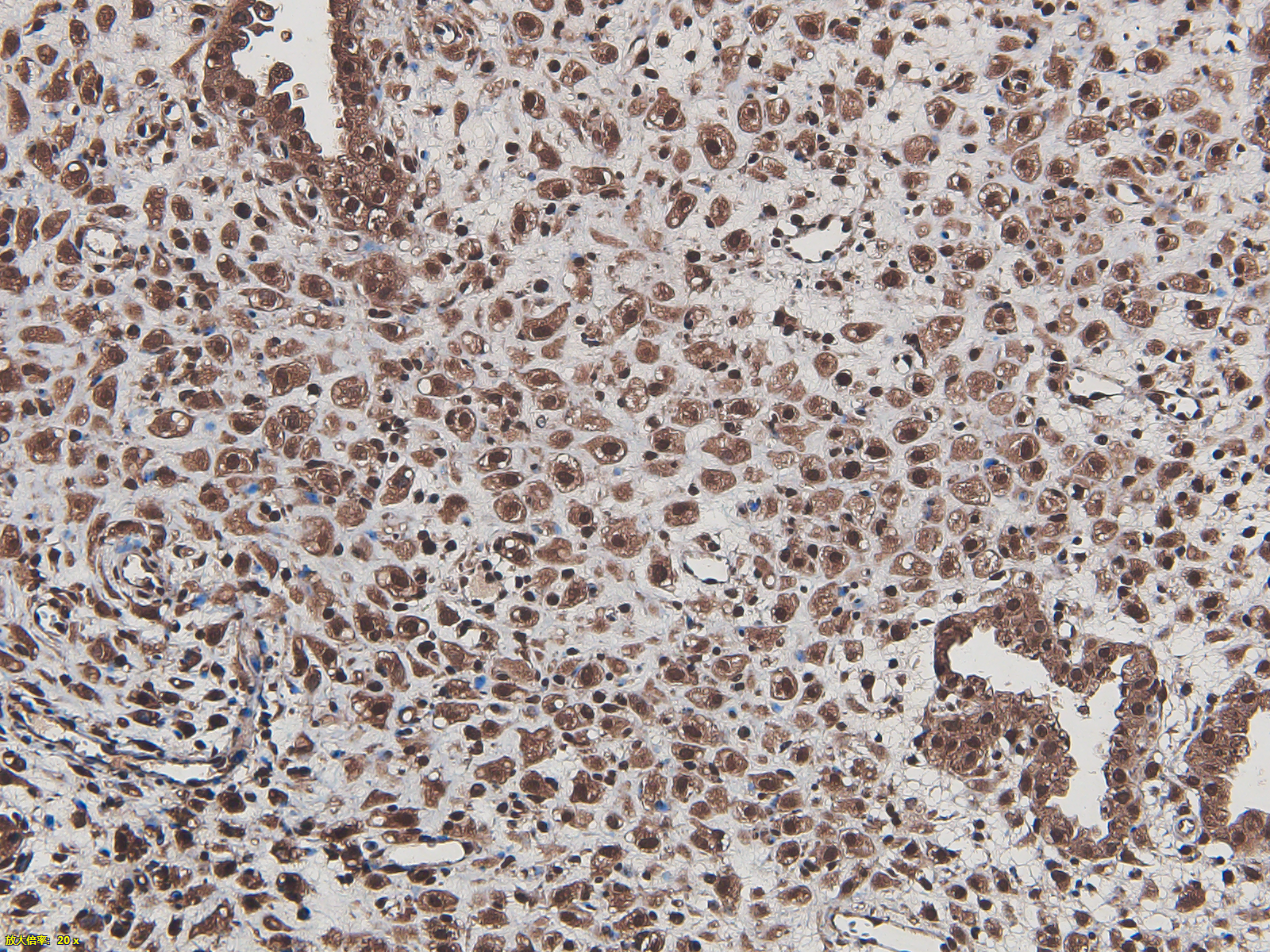

Supplement: Supplementary file 6 — Source data Fig. 4 [file 44318_2024_220_MOESM6_ESM.zip › Figure4/4G/NP-200-3.bmp]

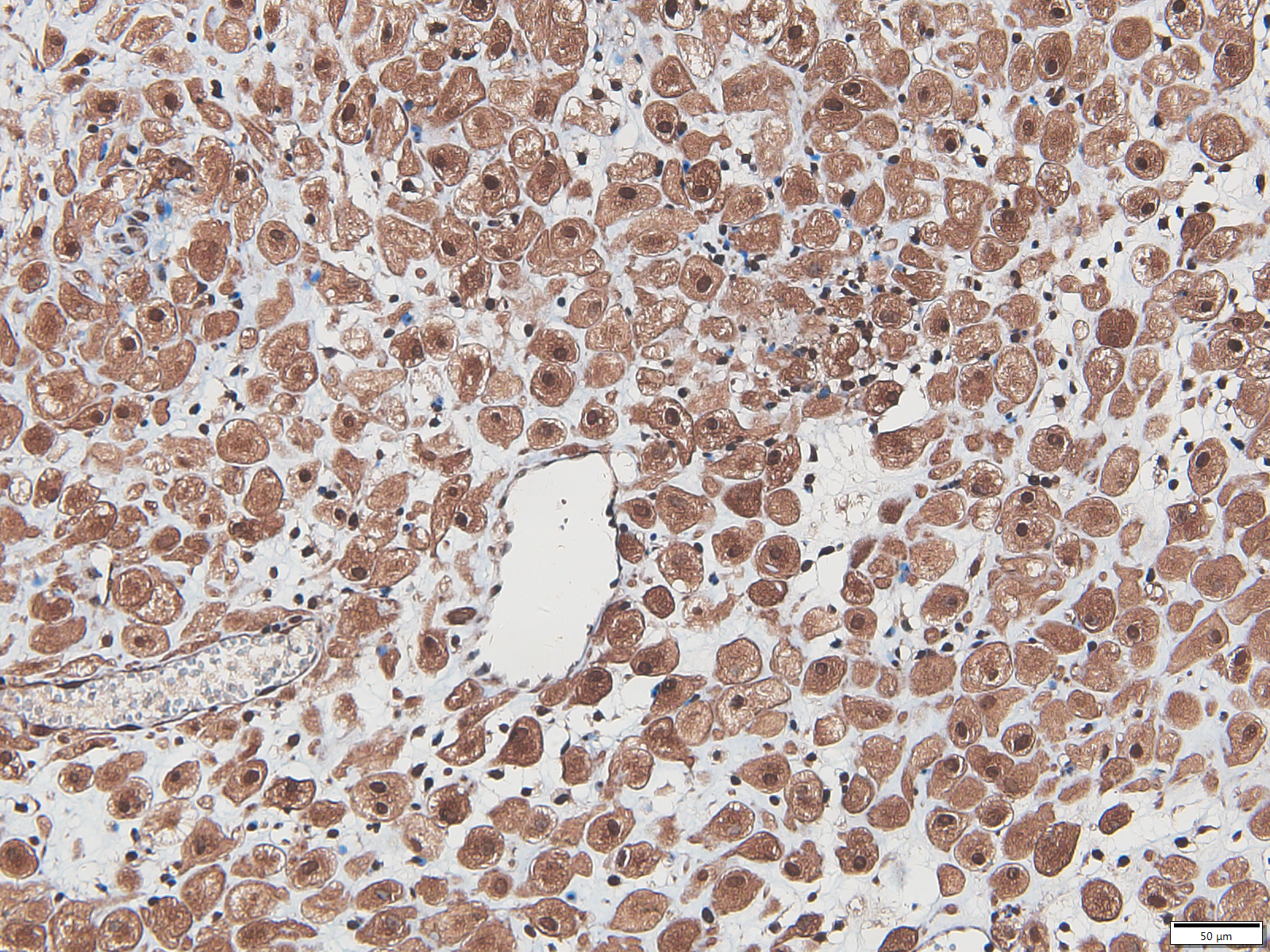

Supplement: Supplementary file 6 — Source data Fig. 4 [file 44318_2024_220_MOESM6_ESM.zip › Figure4/4G/NP-200-4.bmp]

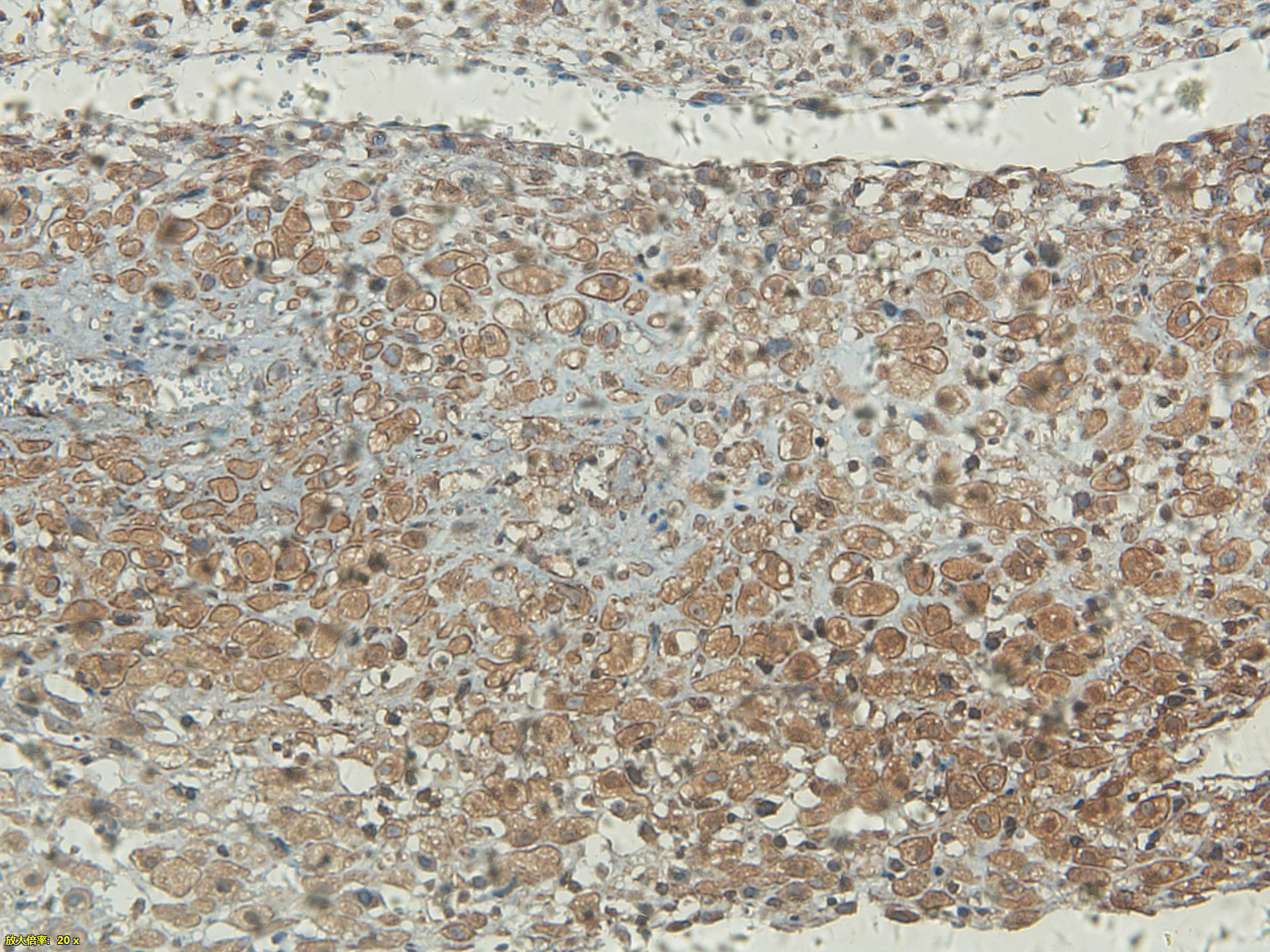

Supplement: Supplementary file 6 — Source data Fig. 4 [file 44318_2024_220_MOESM6_ESM.zip › Figure4/4G/NP-200-5.bmp]

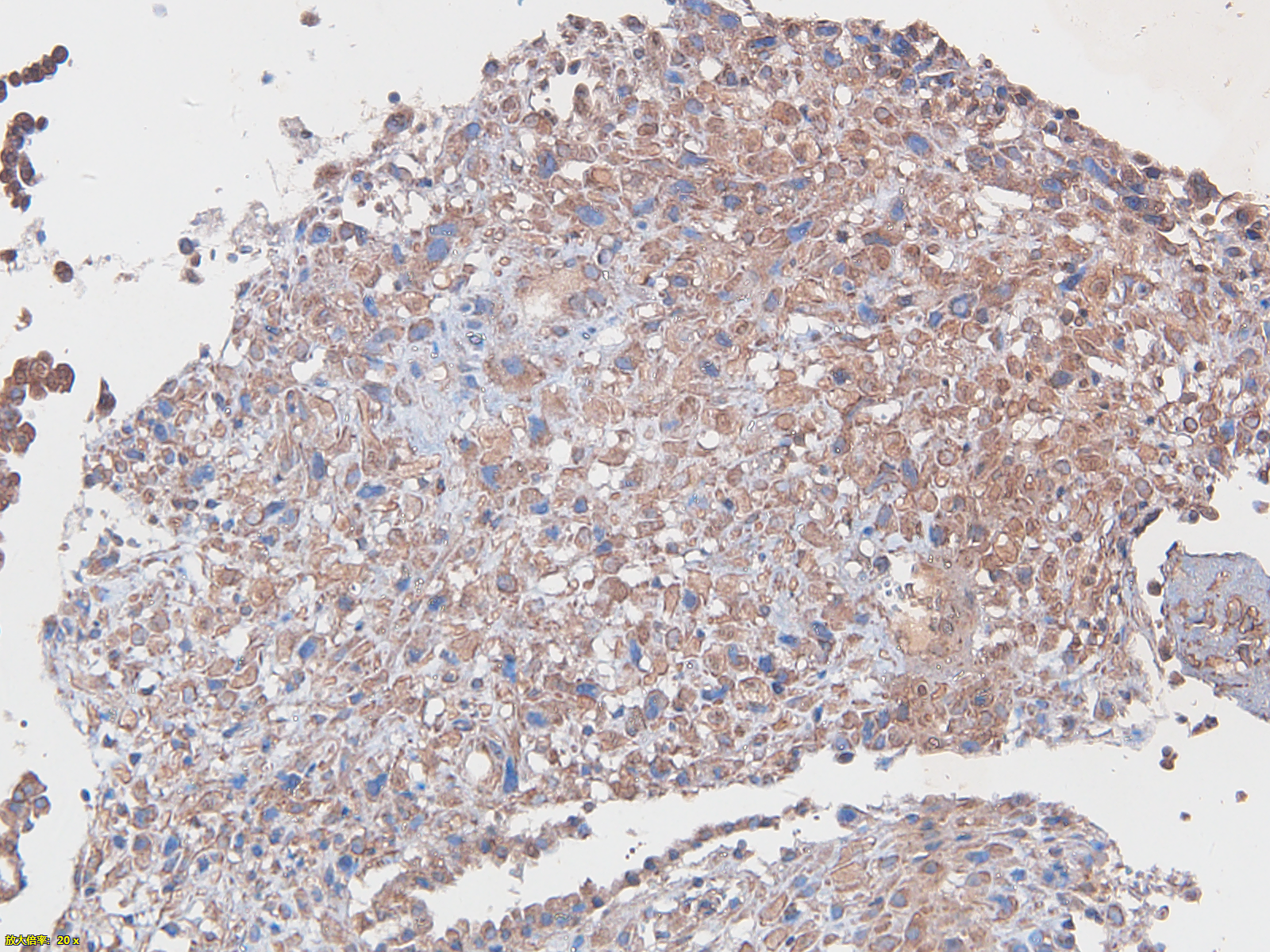

Supplement: Supplementary file 6 — Source data Fig. 4 [file 44318_2024_220_MOESM6_ESM.zip › Figure4/4G/NP-200-6.bmp]

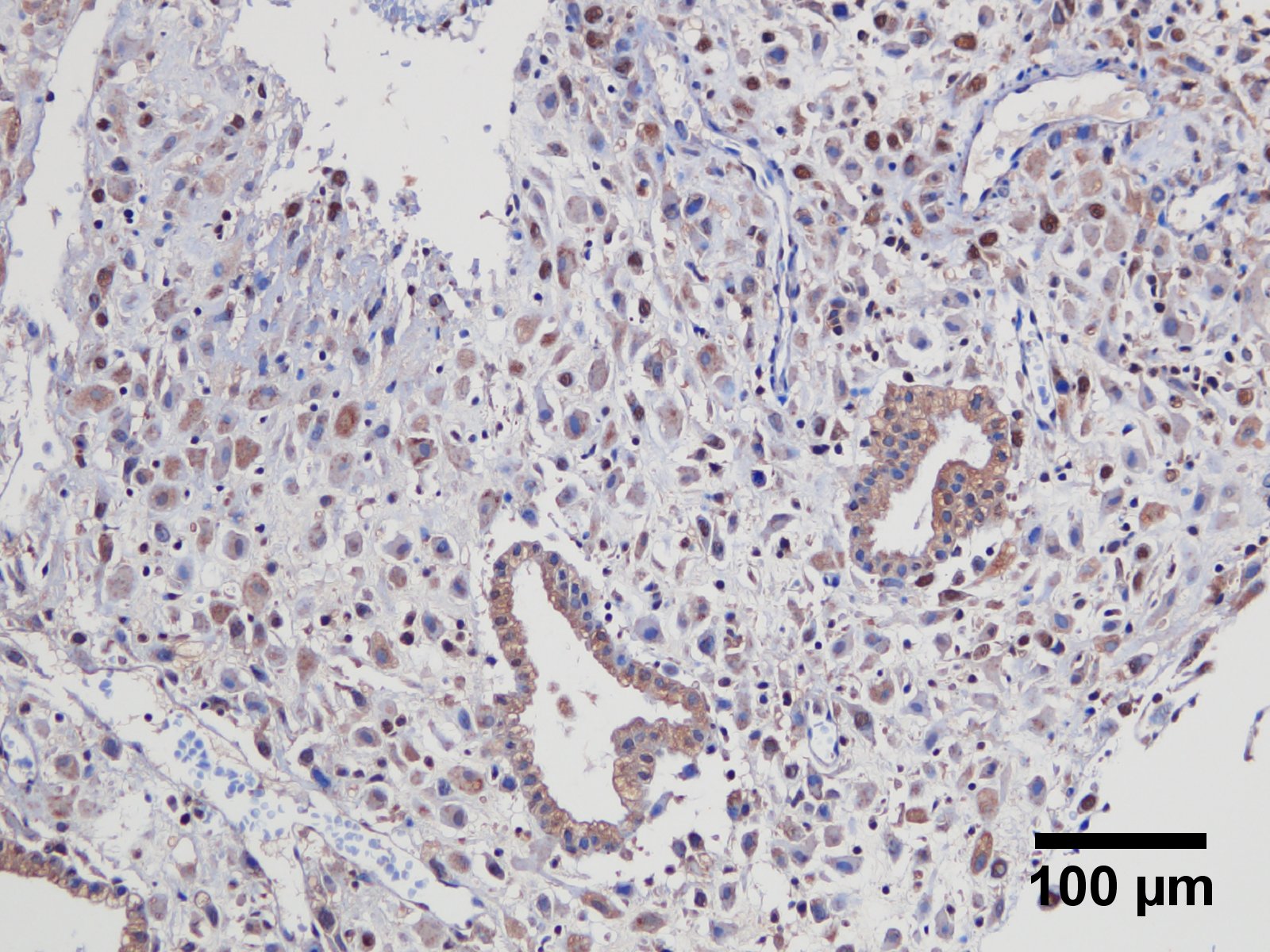

Supplement: Supplementary file 6 — Source data Fig. 4 [file 44318_2024_220_MOESM6_ESM.zip › Figure4/4G/RSA-200-1.jpg]

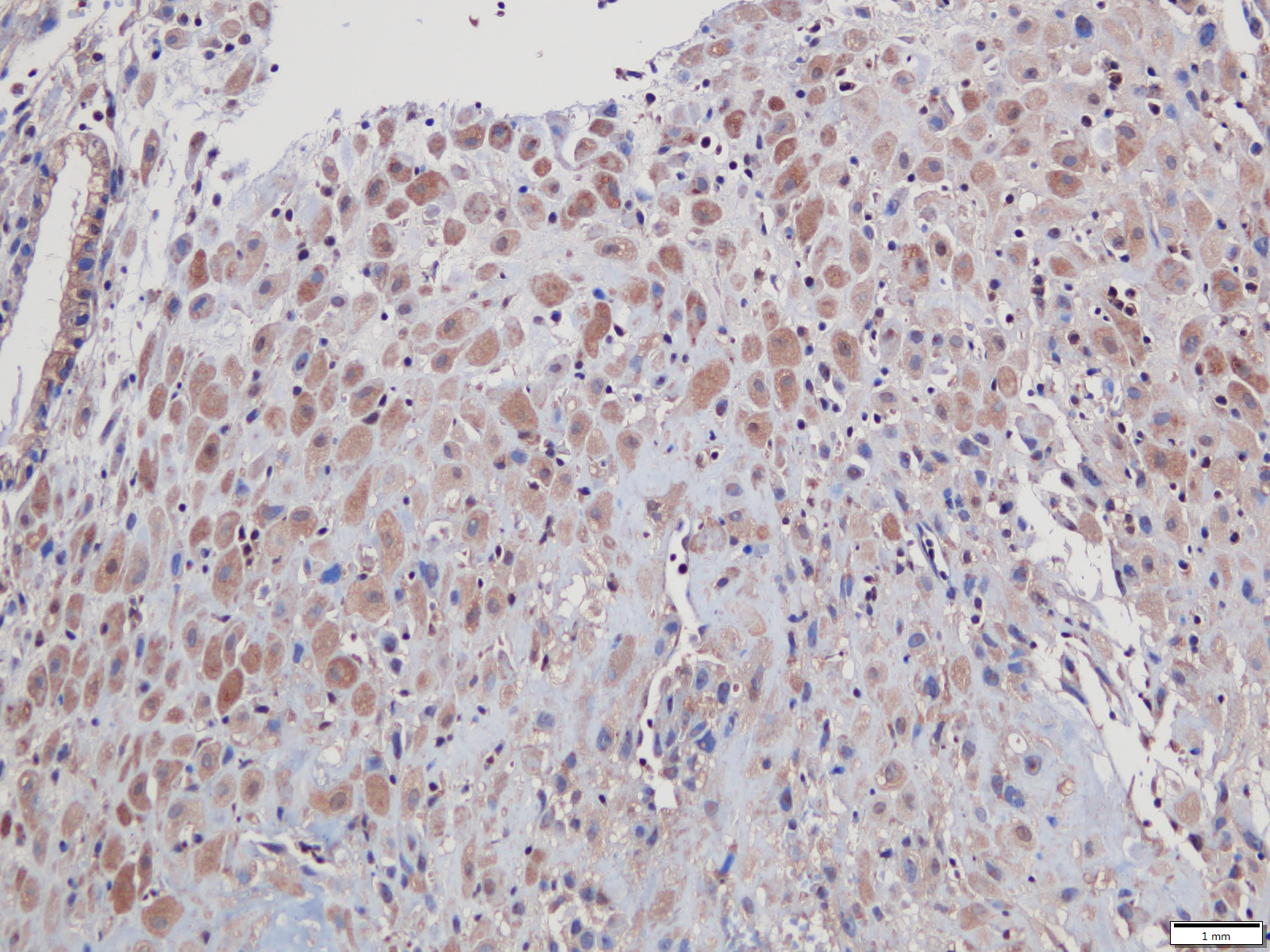

Supplement: Supplementary file 6 — Source data Fig. 4 [file 44318_2024_220_MOESM6_ESM.zip › Figure4/4G/RSA-200-2.bmp]

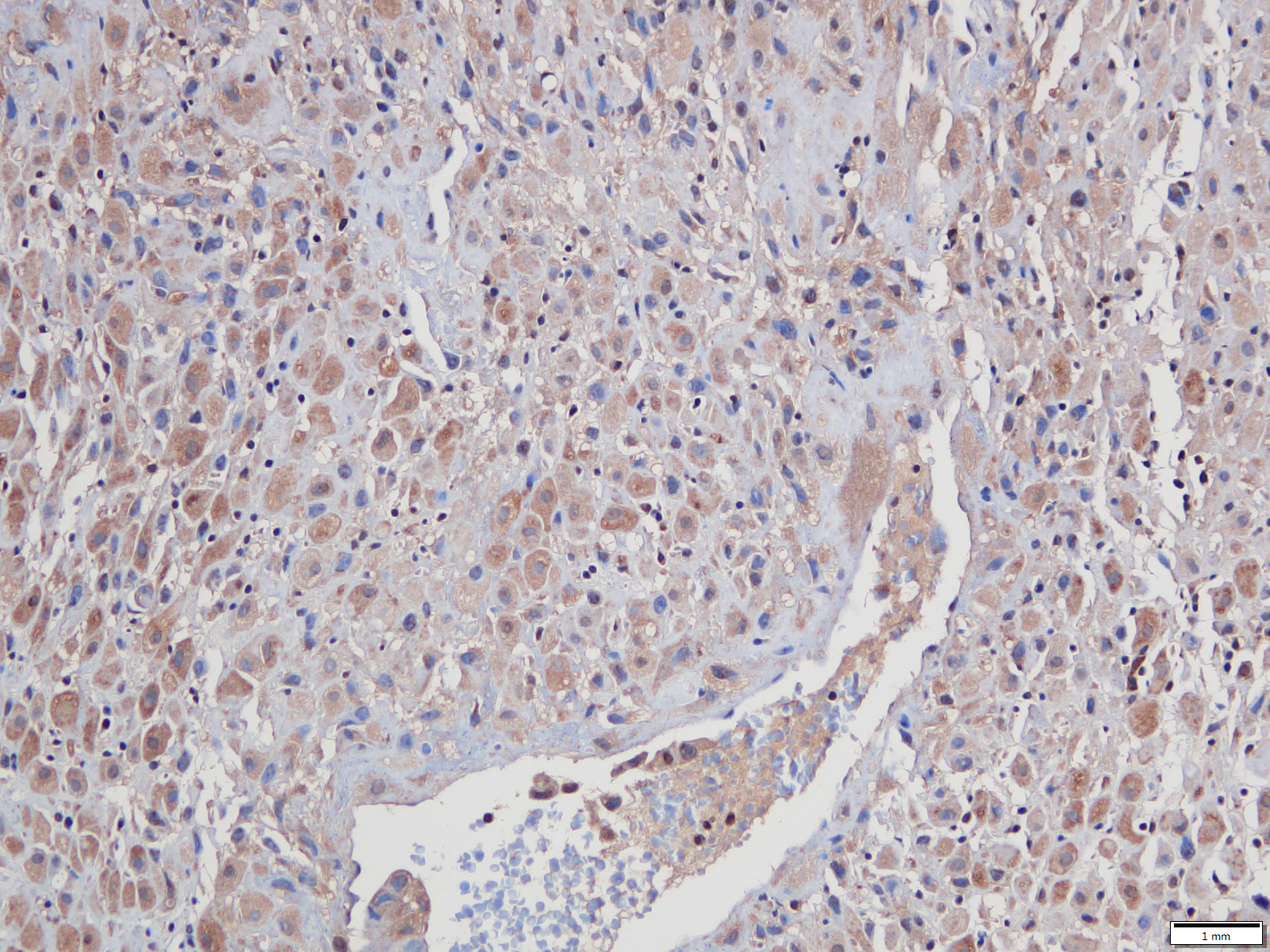

Supplement: Supplementary file 6 — Source data Fig. 4 [file 44318_2024_220_MOESM6_ESM.zip › Figure4/4G/RSA-200-3.bmp]

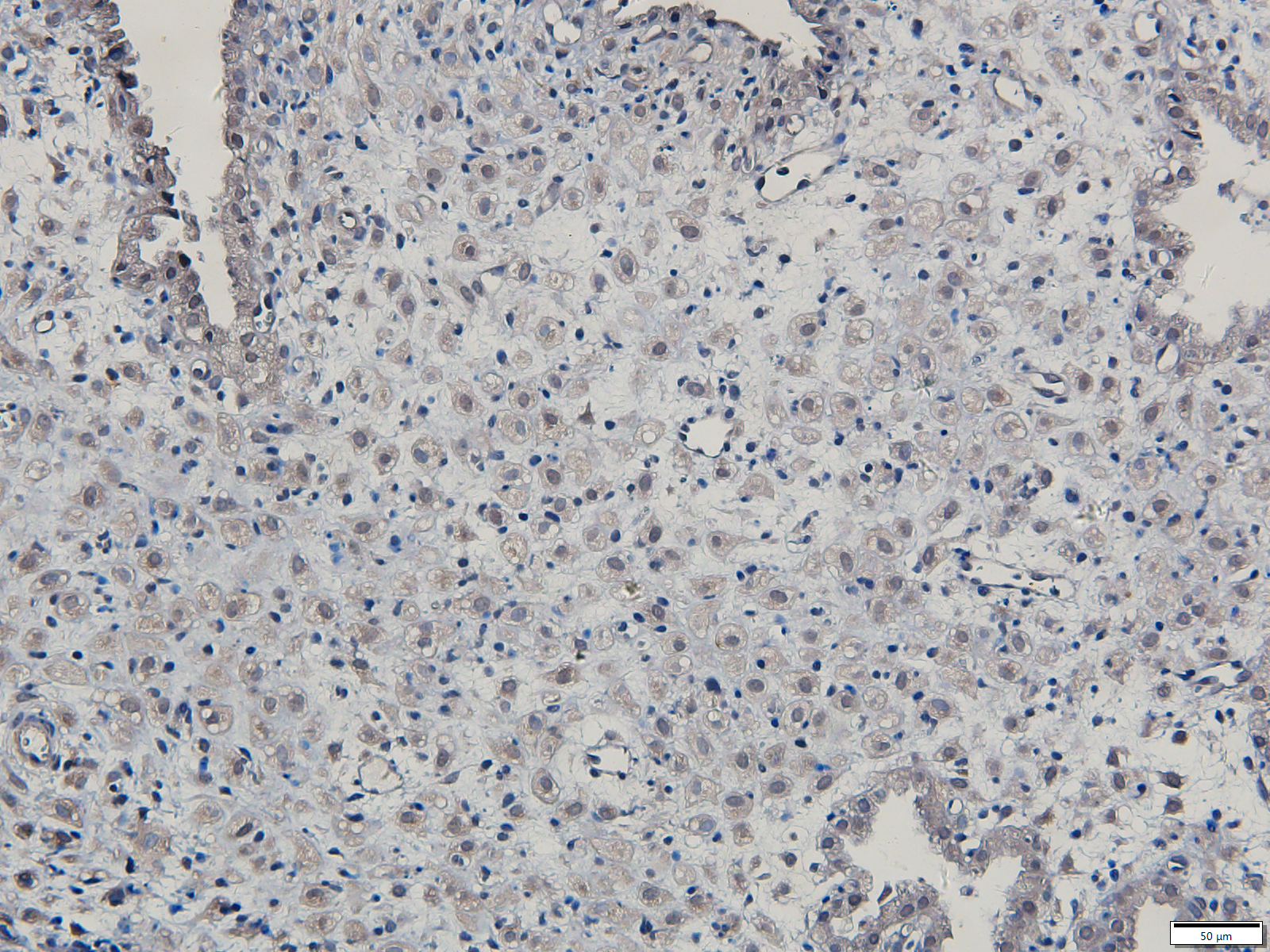

Supplement: Supplementary file 6 — Source data Fig. 4 [file 44318_2024_220_MOESM6_ESM.zip › Figure4/4G/RSA-200-4.bmp]

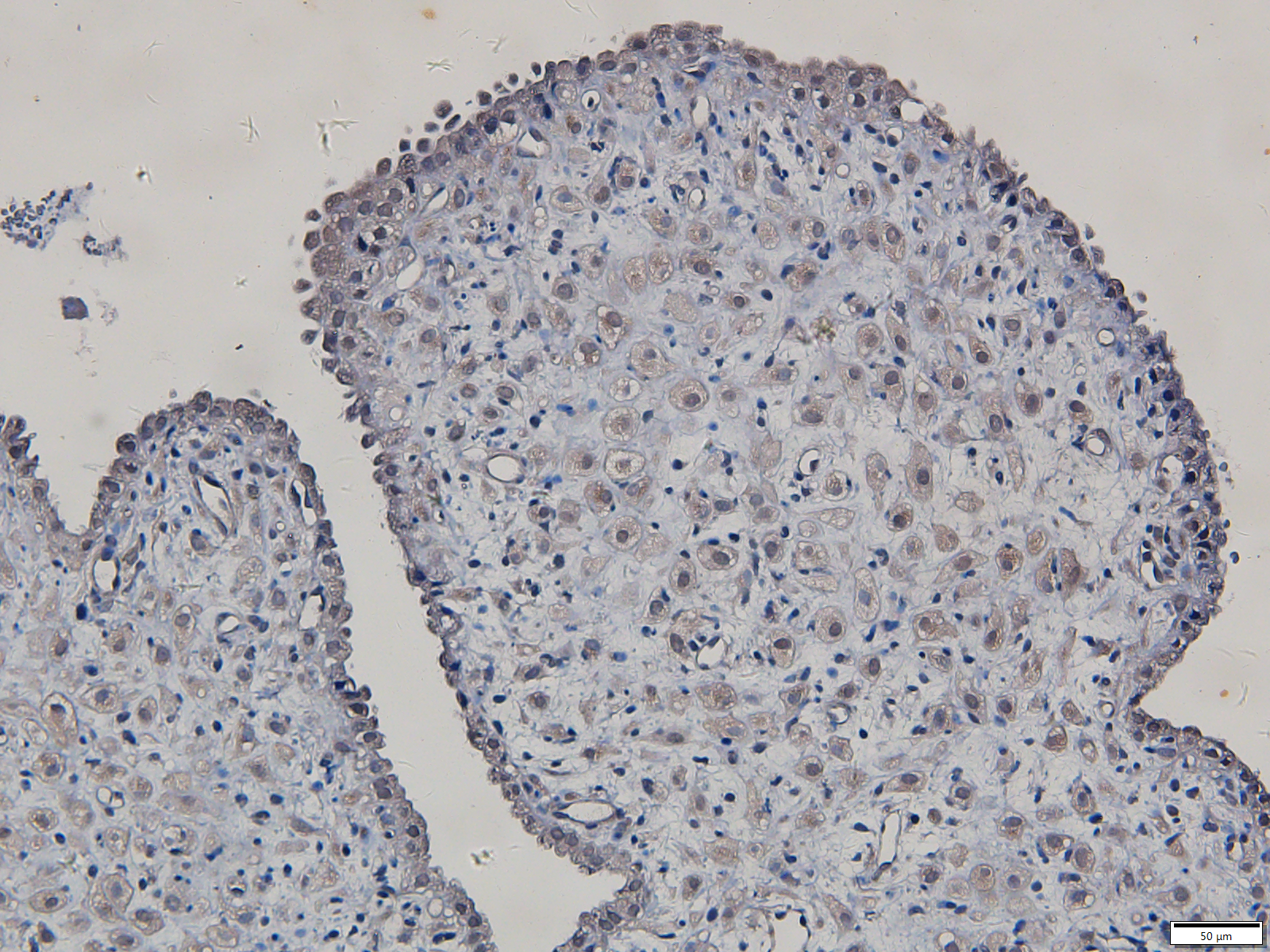

Supplement: Supplementary file 6 — Source data Fig. 4 [file 44318_2024_220_MOESM6_ESM.zip › Figure4/4G/RSA-200-5.bmp]

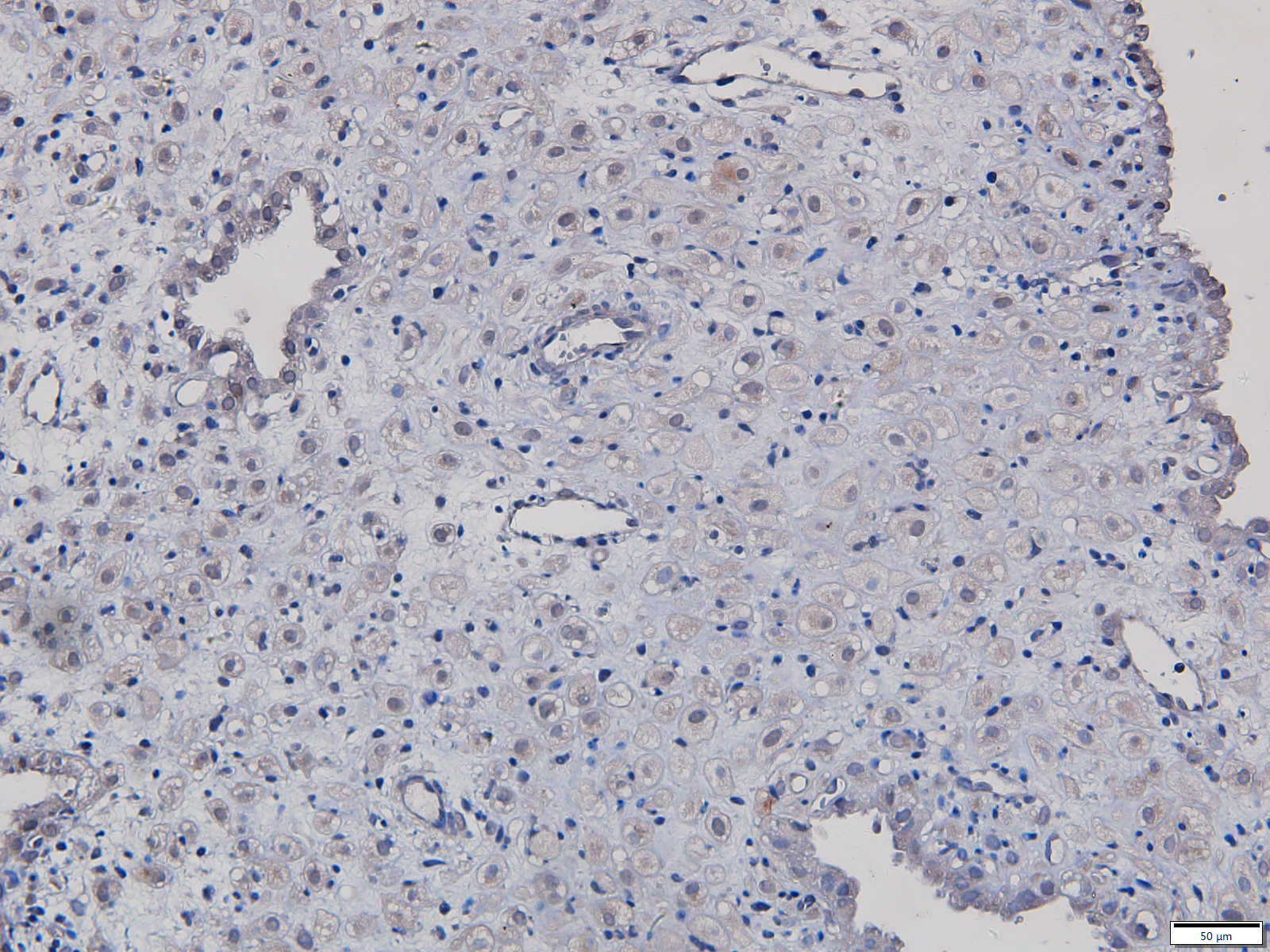

Supplement: Supplementary file 6 — Source data Fig. 4 [file 44318_2024_220_MOESM6_ESM.zip › Figure4/4G/RSA-200-6.bmp]

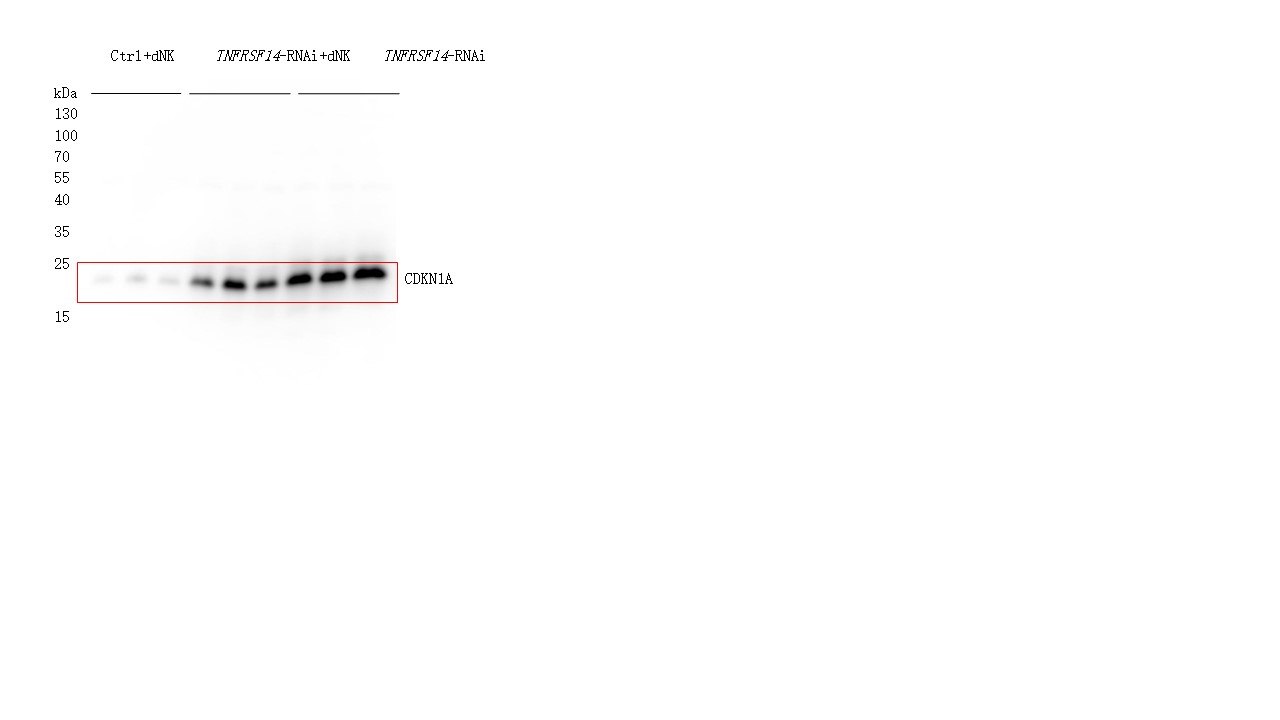

Supplement: Supplementary file 6 — Source data Fig. 4 [file 44318_2024_220_MOESM6_ESM.zip › Figure4/4H/western CDKN1A.jpg]

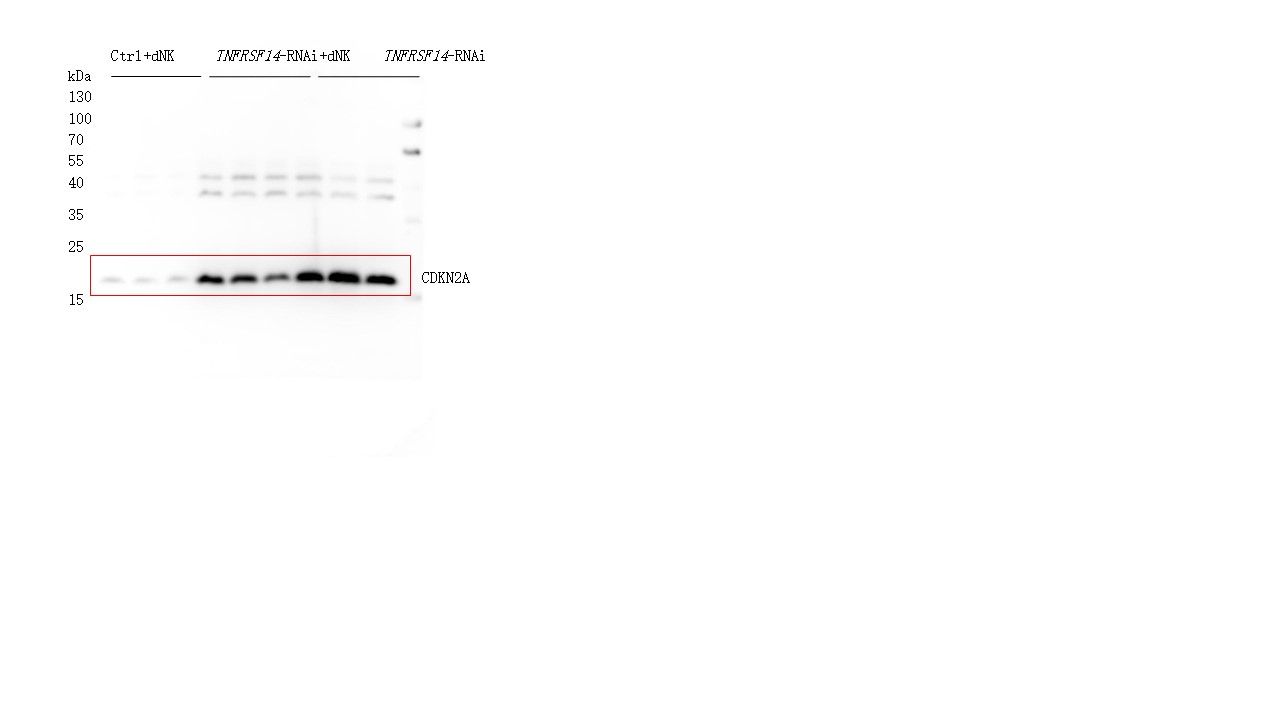

Supplement: Supplementary file 6 — Source data Fig. 4 [file 44318_2024_220_MOESM6_ESM.zip › Figure4/4H/western CDKN2A.jpg]

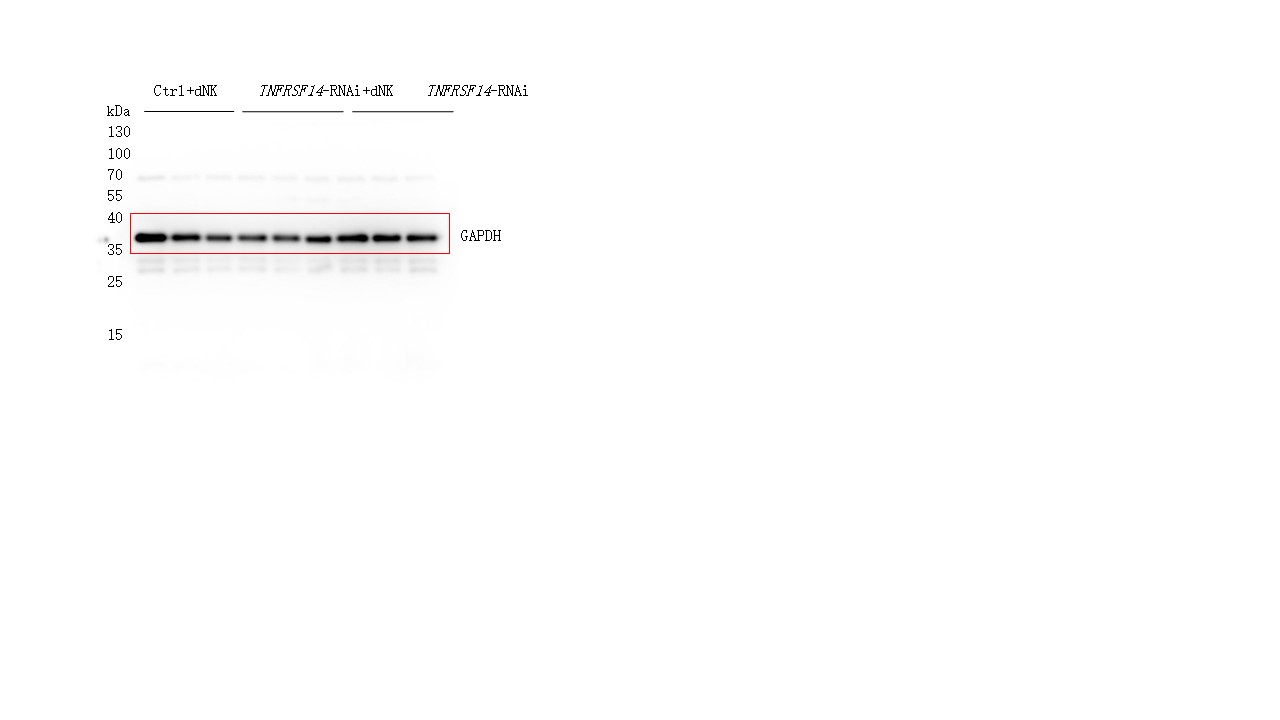

Supplement: Supplementary file 6 — Source data Fig. 4 [file 44318_2024_220_MOESM6_ESM.zip › Figure4/4H/western GAPDH.jpg]

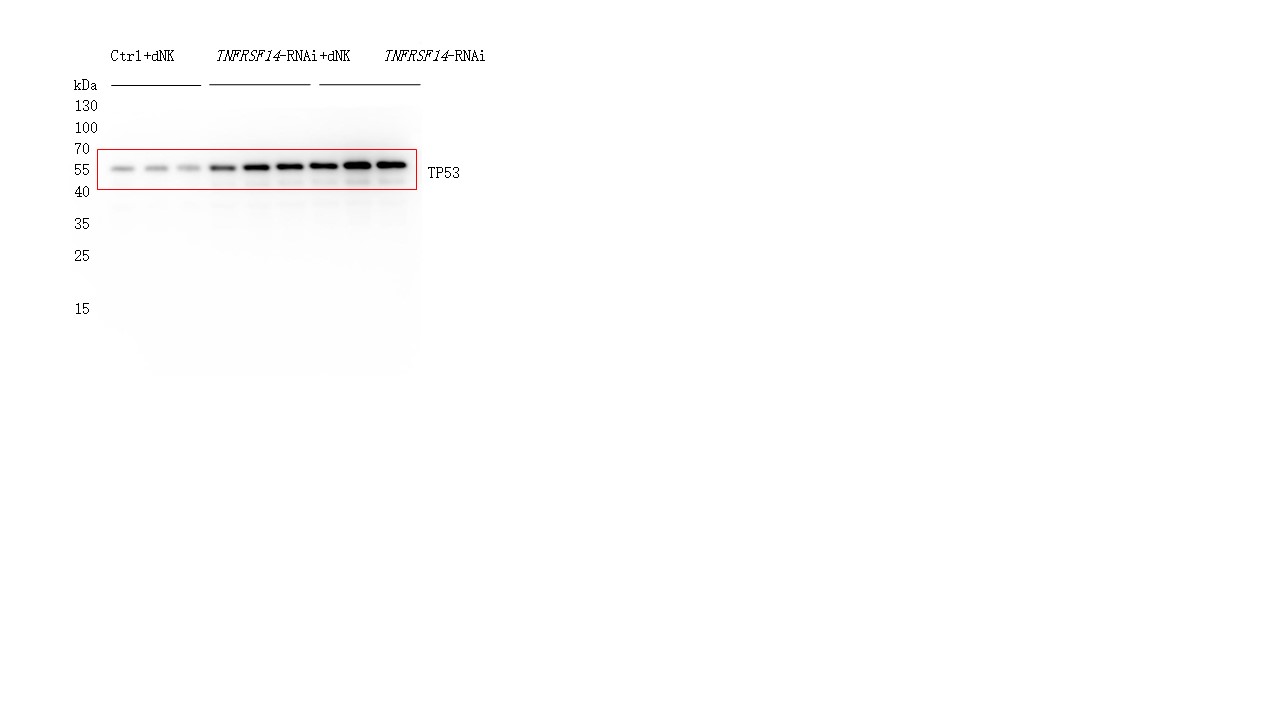

Supplement: Supplementary file 6 — Source data Fig. 4 [file 44318_2024_220_MOESM6_ESM.zip › Figure4/4H/western TP53.jpg]

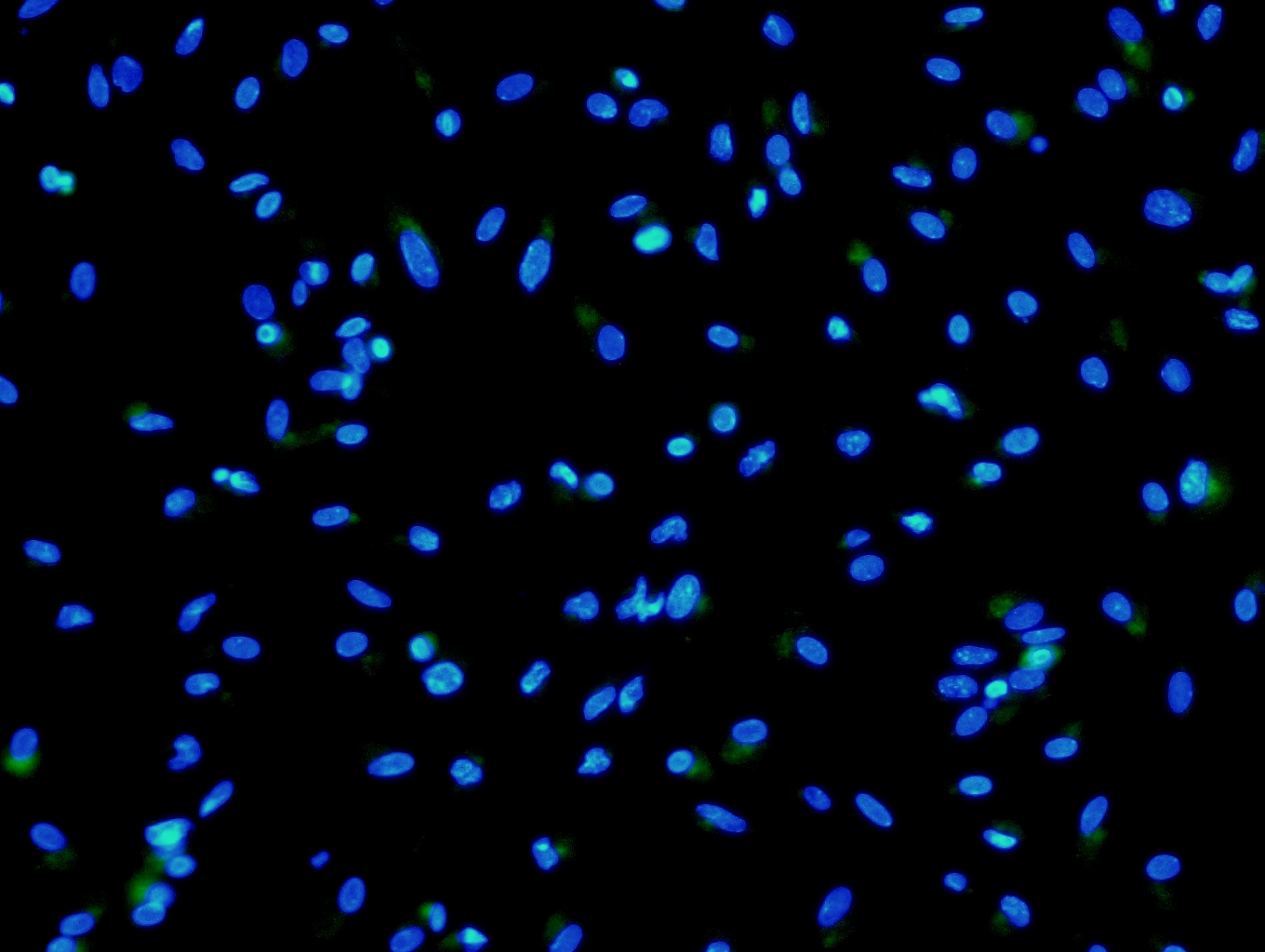

Supplement: Supplementary file 6 — Source data Fig. 4 [file 44318_2024_220_MOESM6_ESM.zip › Figure4/4I/Ctrl+NK-1 (1).jpg]

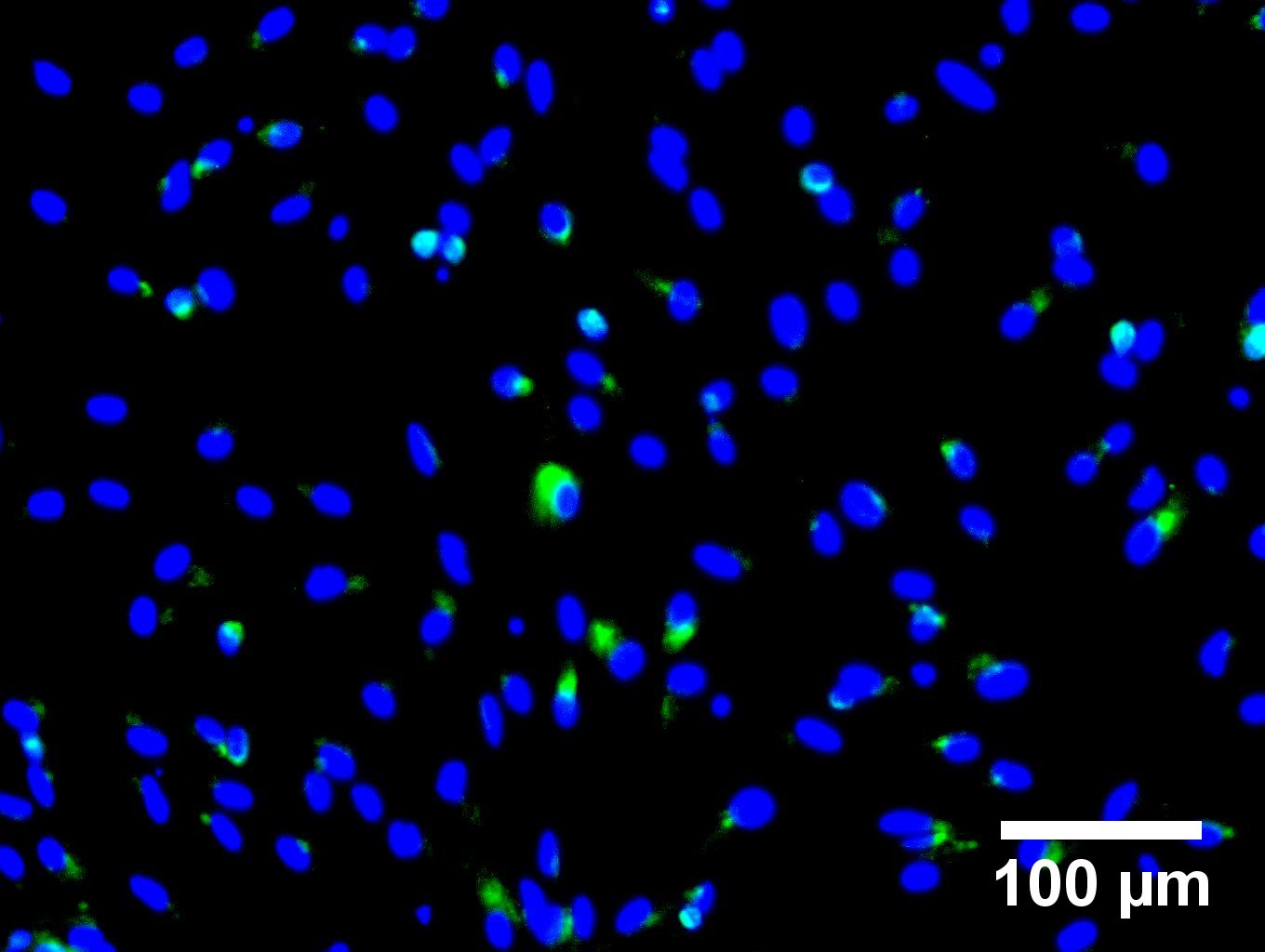

Supplement: Supplementary file 6 — Source data Fig. 4 [file 44318_2024_220_MOESM6_ESM.zip › Figure4/4I/Ctrl+NK-1 (2).jpg]

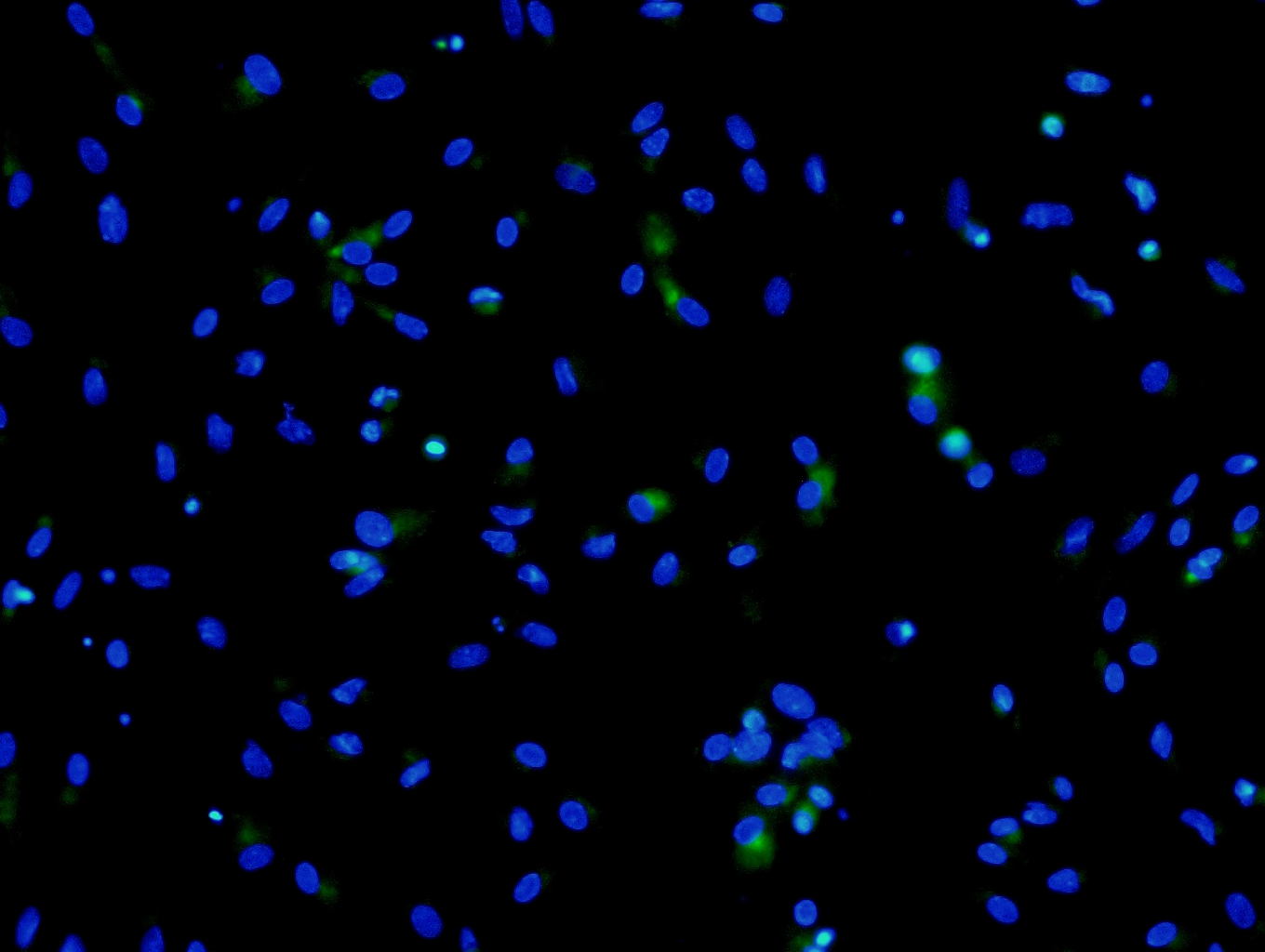

Supplement: Supplementary file 6 — Source data Fig. 4 [file 44318_2024_220_MOESM6_ESM.zip › Figure4/4I/Ctrl+NK-1 (3).jpg]

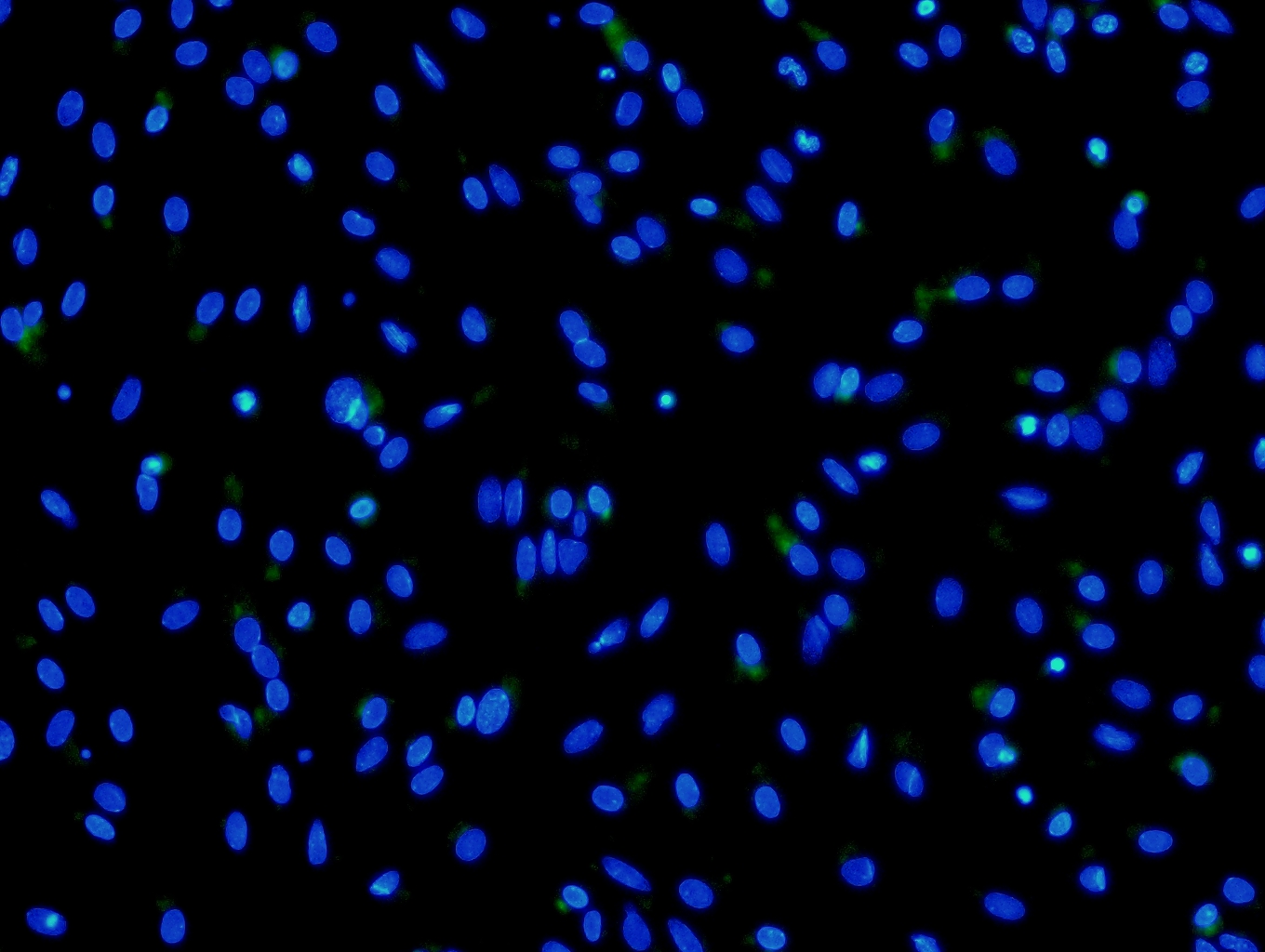

Supplement: Supplementary file 6 — Source data Fig. 4 [file 44318_2024_220_MOESM6_ESM.zip › Figure4/4I/Ctrl+NK-2 (1).jpg]

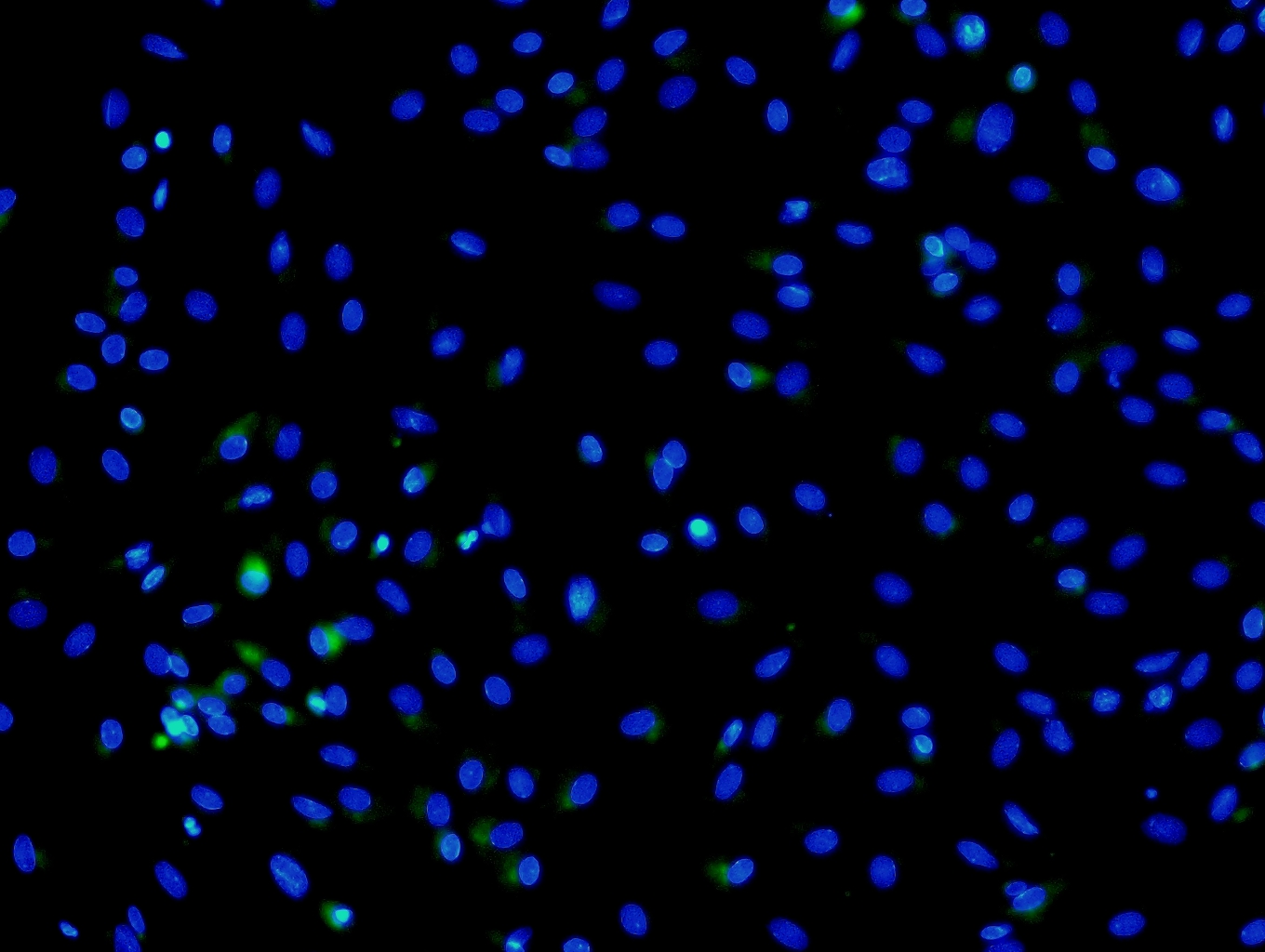

Supplement: Supplementary file 6 — Source data Fig. 4 [file 44318_2024_220_MOESM6_ESM.zip › Figure4/4I/Ctrl+NK-2 (2).jpg]

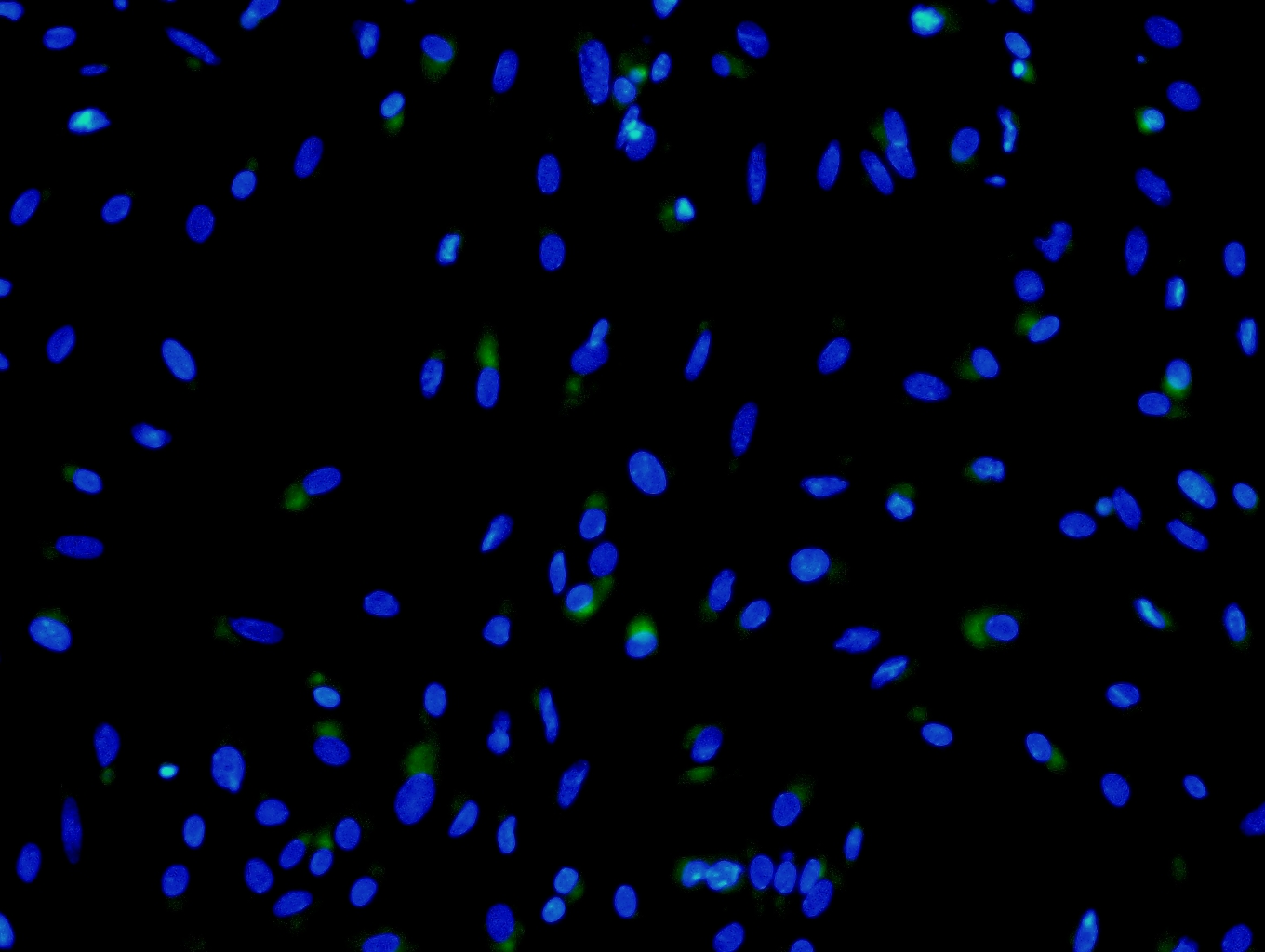

Supplement: Supplementary file 6 — Source data Fig. 4 [file 44318_2024_220_MOESM6_ESM.zip › Figure4/4I/Ctrl+NK-2 (3).jpg]

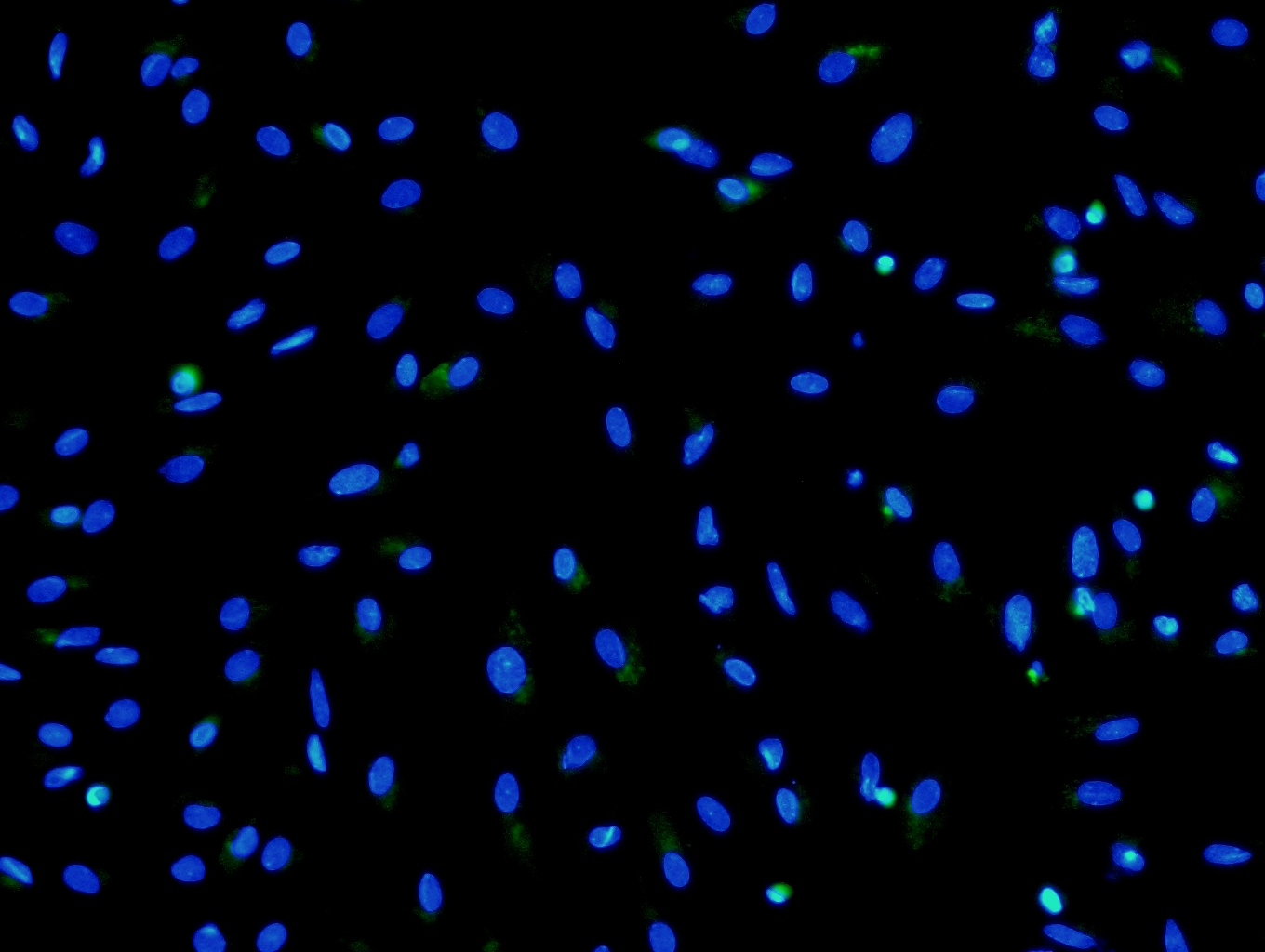

Supplement: Supplementary file 6 — Source data Fig. 4 [file 44318_2024_220_MOESM6_ESM.zip › Figure4/4I/Ctrl+NK-3 (1).jpg]

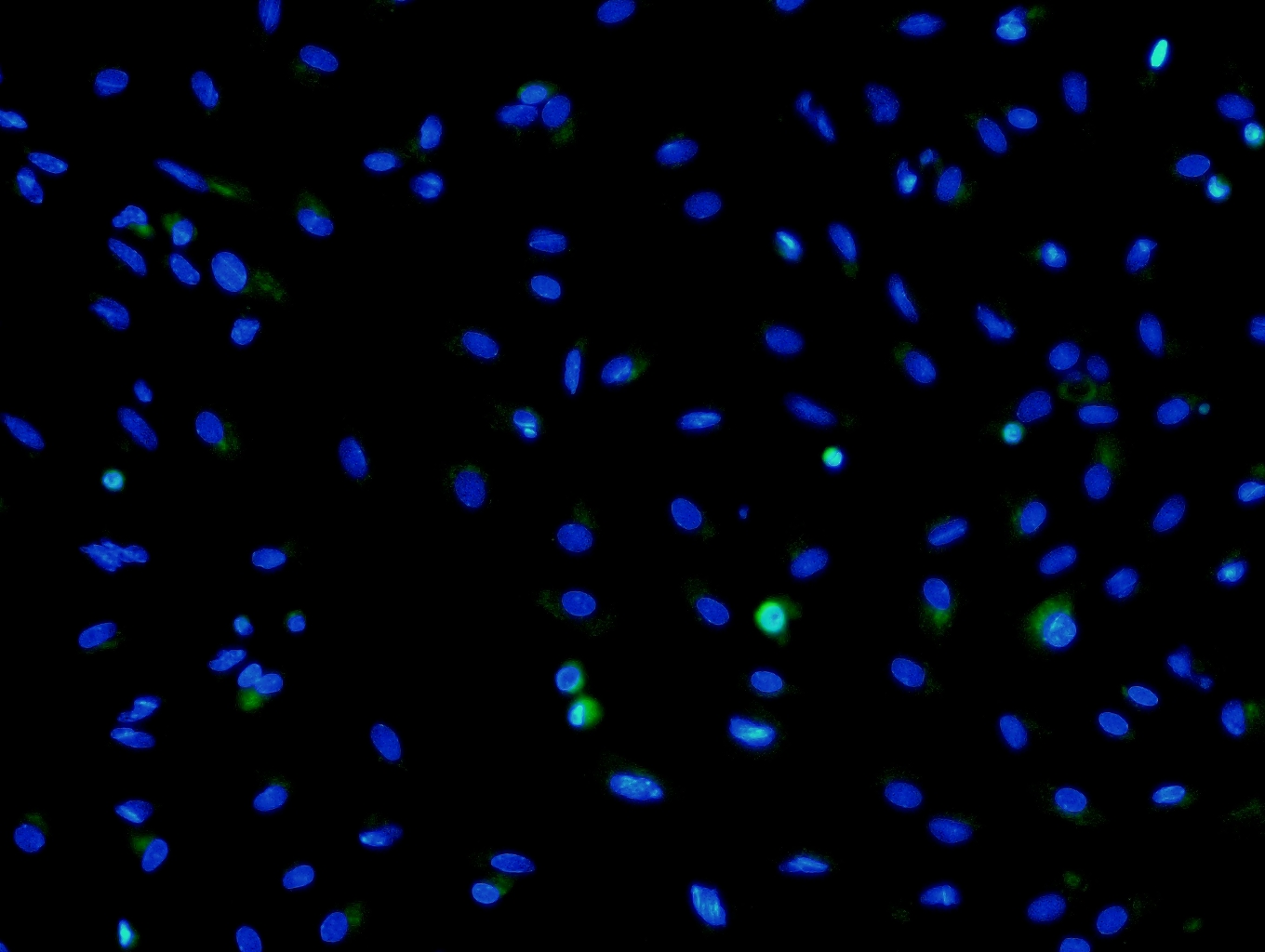

Supplement: Supplementary file 6 — Source data Fig. 4 [file 44318_2024_220_MOESM6_ESM.zip › Figure4/4I/Ctrl+NK-3 (2).jpg]

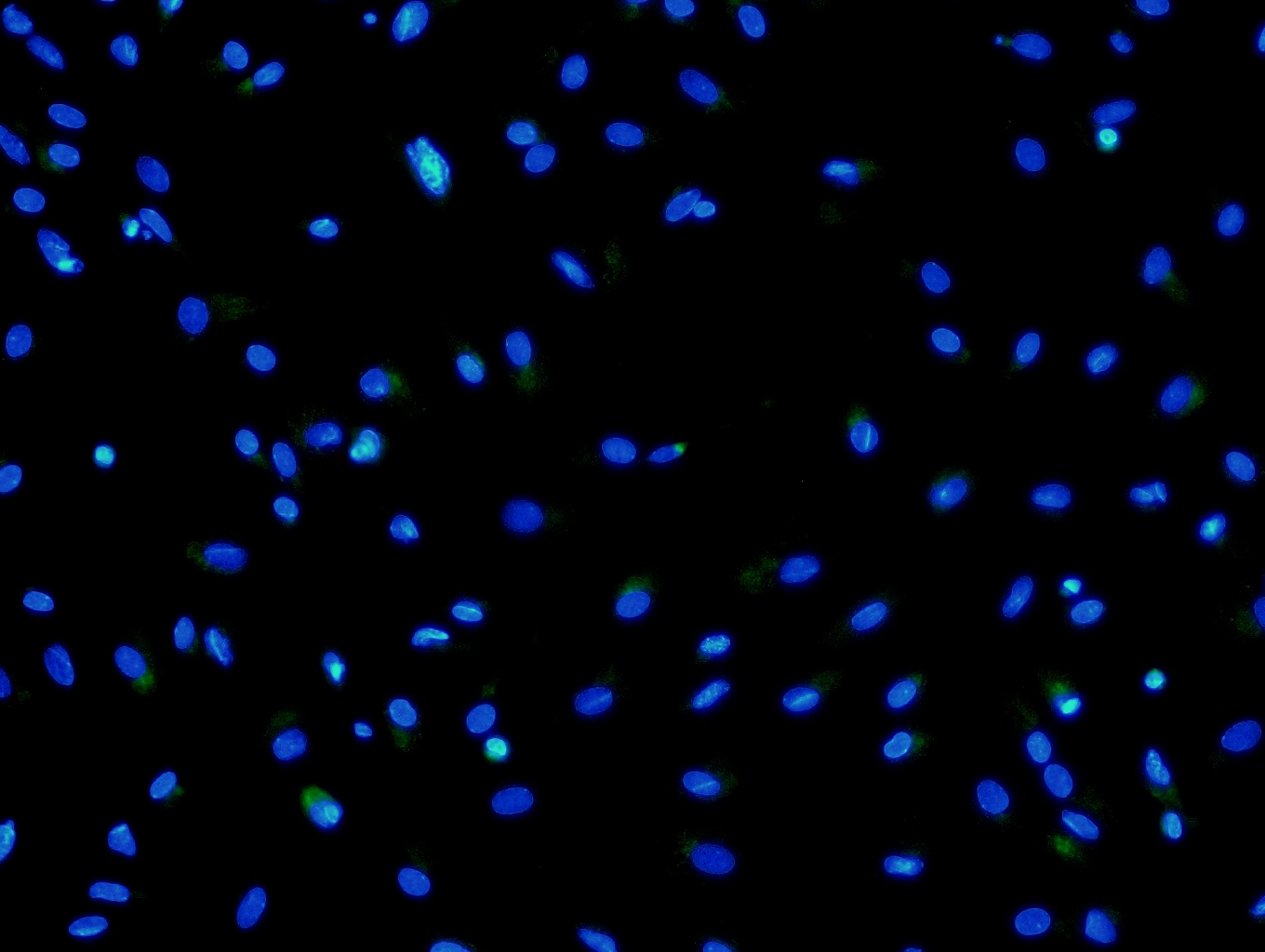

Supplement: Supplementary file 6 — Source data Fig. 4 [file 44318_2024_220_MOESM6_ESM.zip › Figure4/4I/Ctrl+NK-3 (3).jpg]

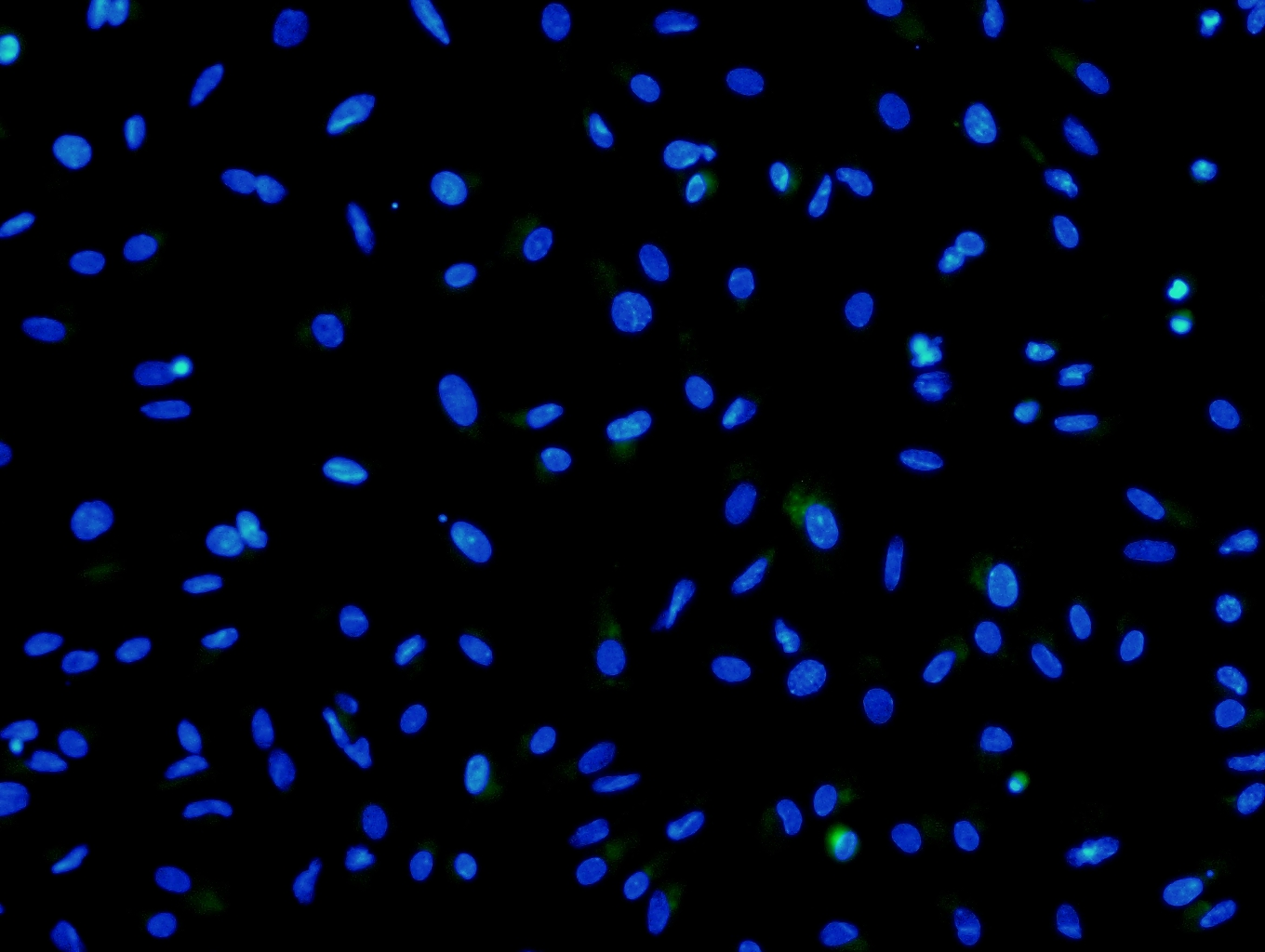

Supplement: Supplementary file 6 — Source data Fig. 4 [file 44318_2024_220_MOESM6_ESM.zip › Figure4/4I/Ctrl+NK-4 (1).jpg]

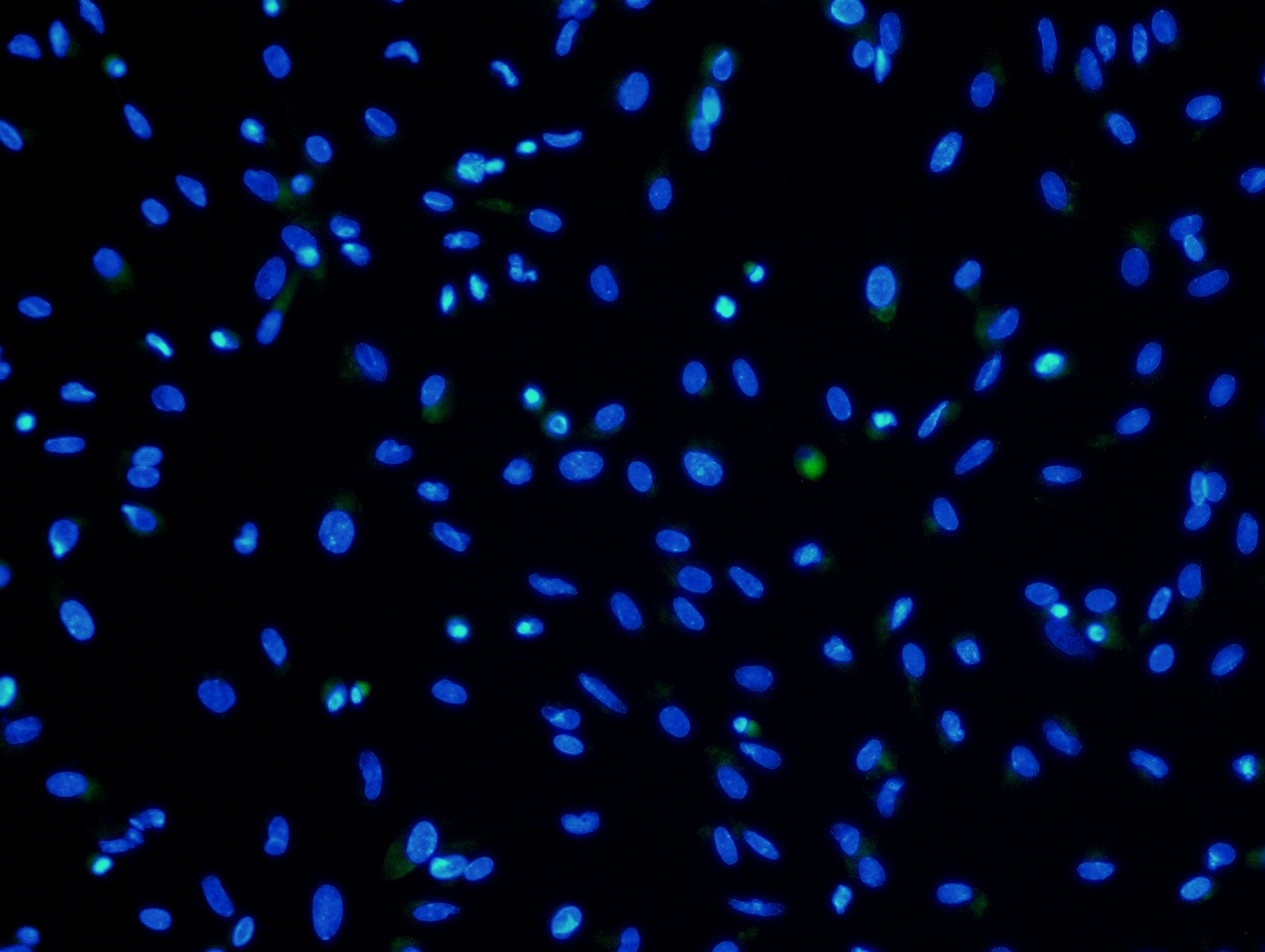

Supplement: Supplementary file 6 — Source data Fig. 4 [file 44318_2024_220_MOESM6_ESM.zip › Figure4/4I/Ctrl+NK-4 (2).jpg]

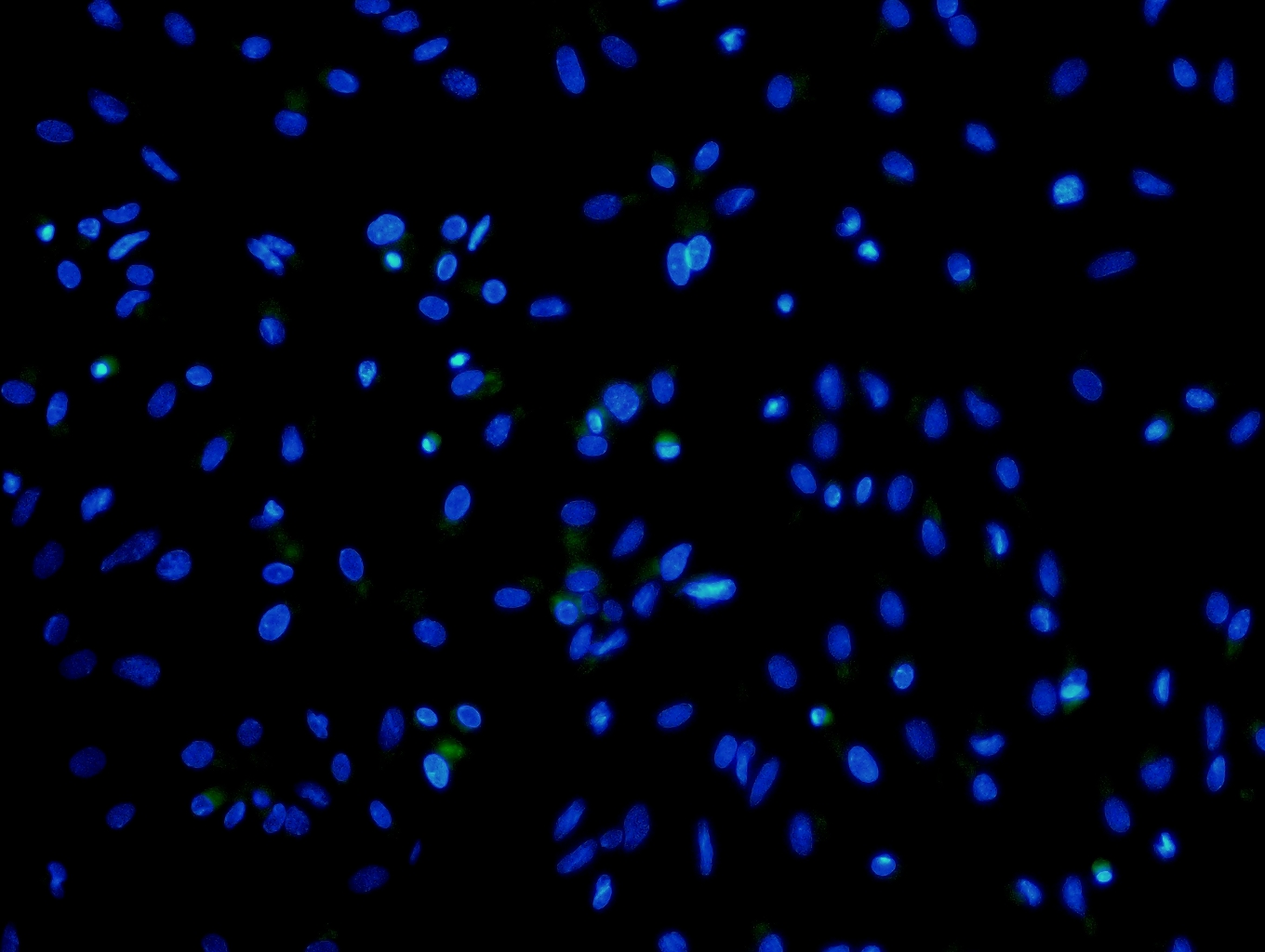

Supplement: Supplementary file 6 — Source data Fig. 4 [file 44318_2024_220_MOESM6_ESM.zip › Figure4/4I/Ctrl+NK-4 (3).jpg]

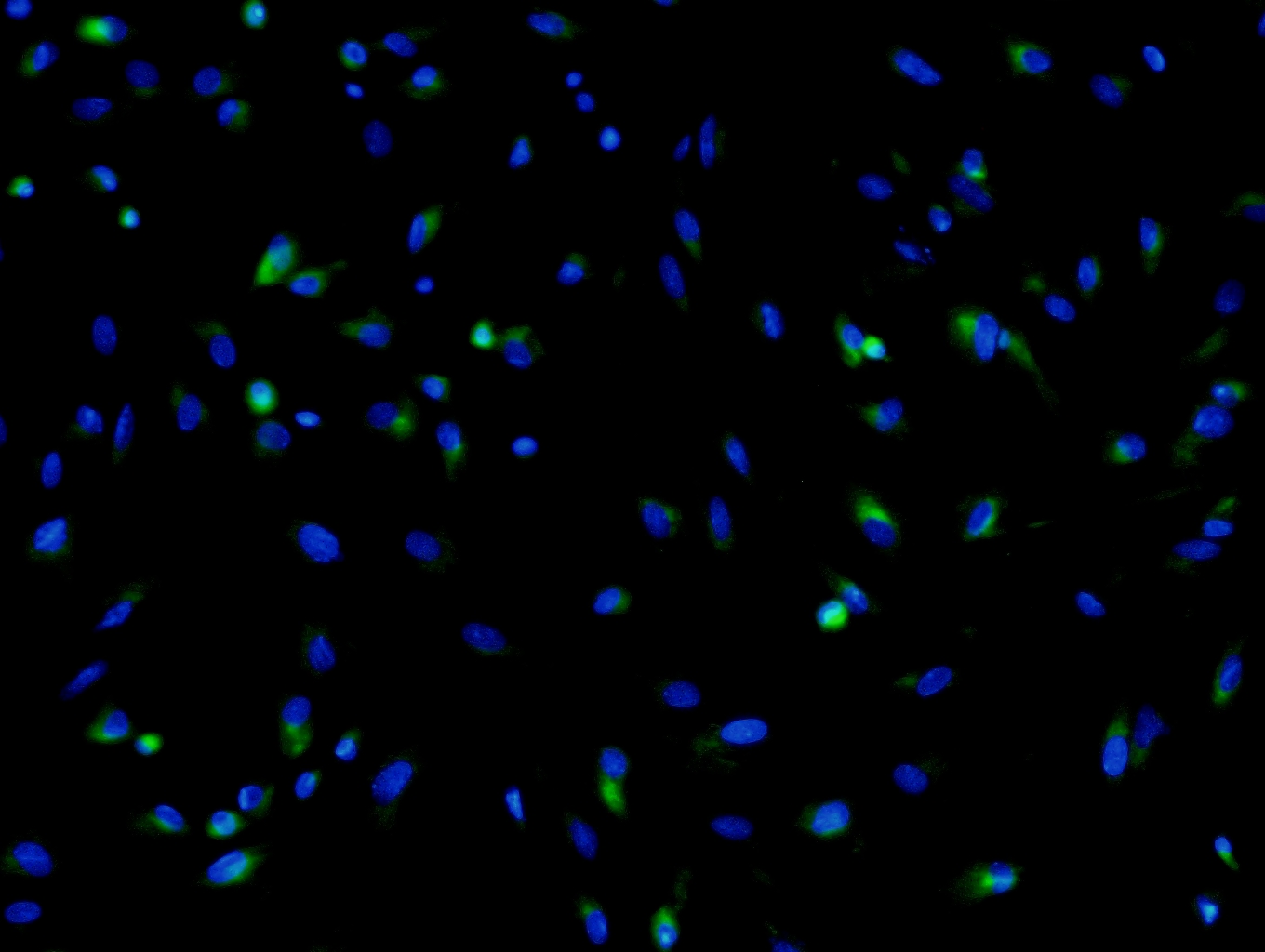

Supplement: Supplementary file 6 — Source data Fig. 4 [file 44318_2024_220_MOESM6_ESM.zip › Figure4/4I/TNFRSF14-+NK-1 (1).jpg]

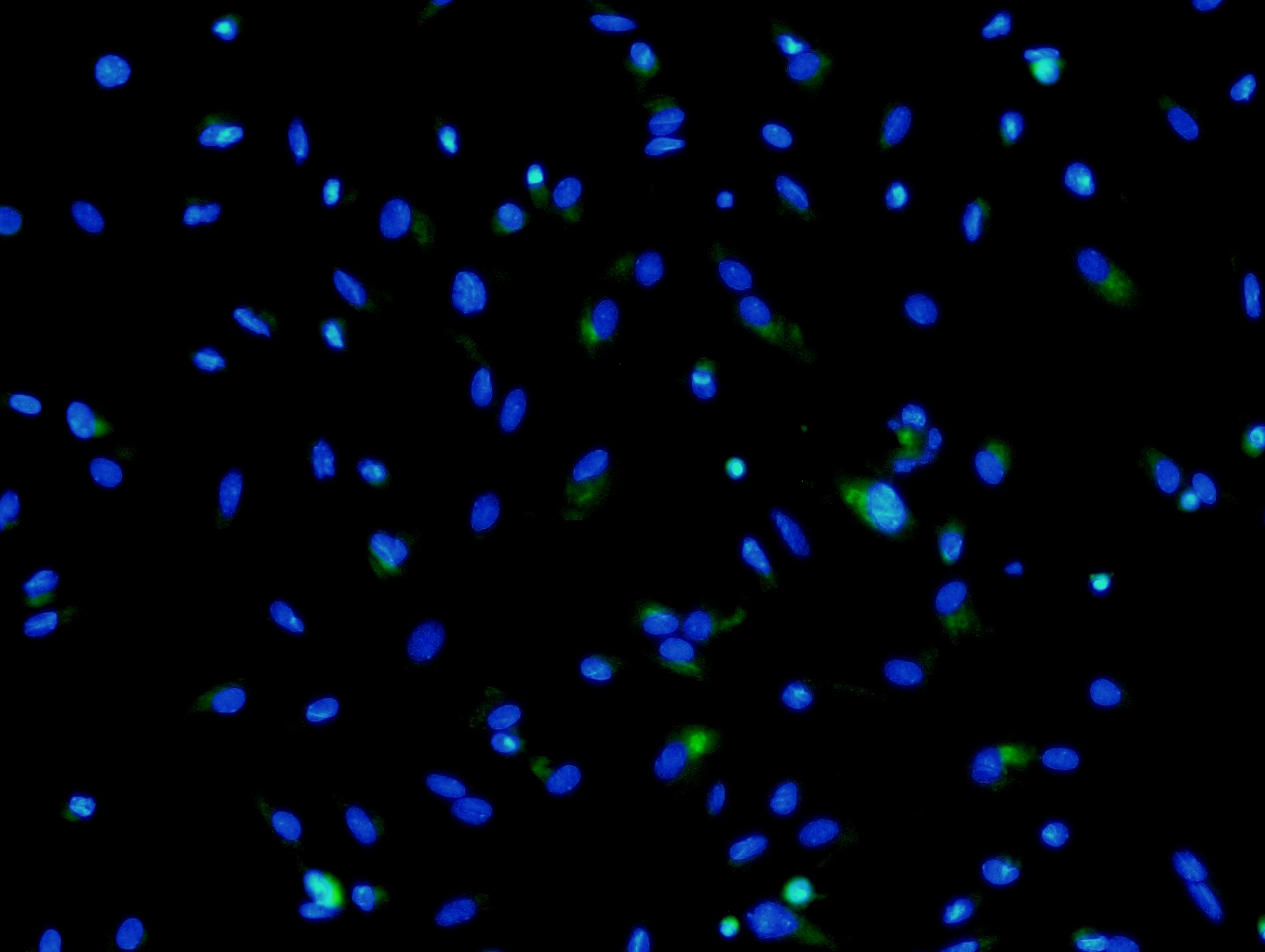

Supplement: Supplementary file 6 — Source data Fig. 4 [file 44318_2024_220_MOESM6_ESM.zip › Figure4/4I/TNFRSF14-+NK-1 (2).jpg]

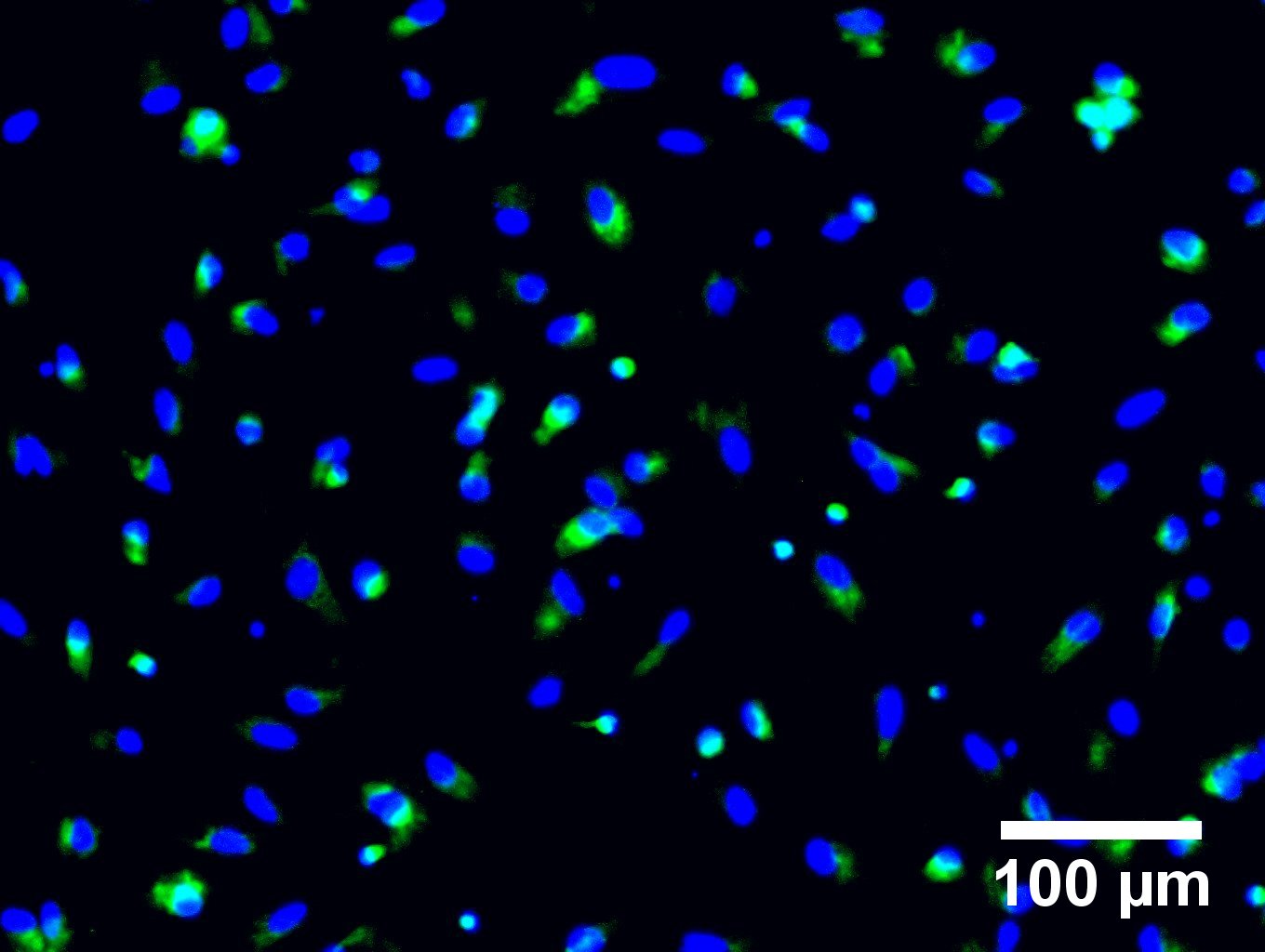

Supplement: Supplementary file 6 — Source data Fig. 4 [file 44318_2024_220_MOESM6_ESM.zip › Figure4/4I/TNFRSF14-+NK-1 (3).jpg]

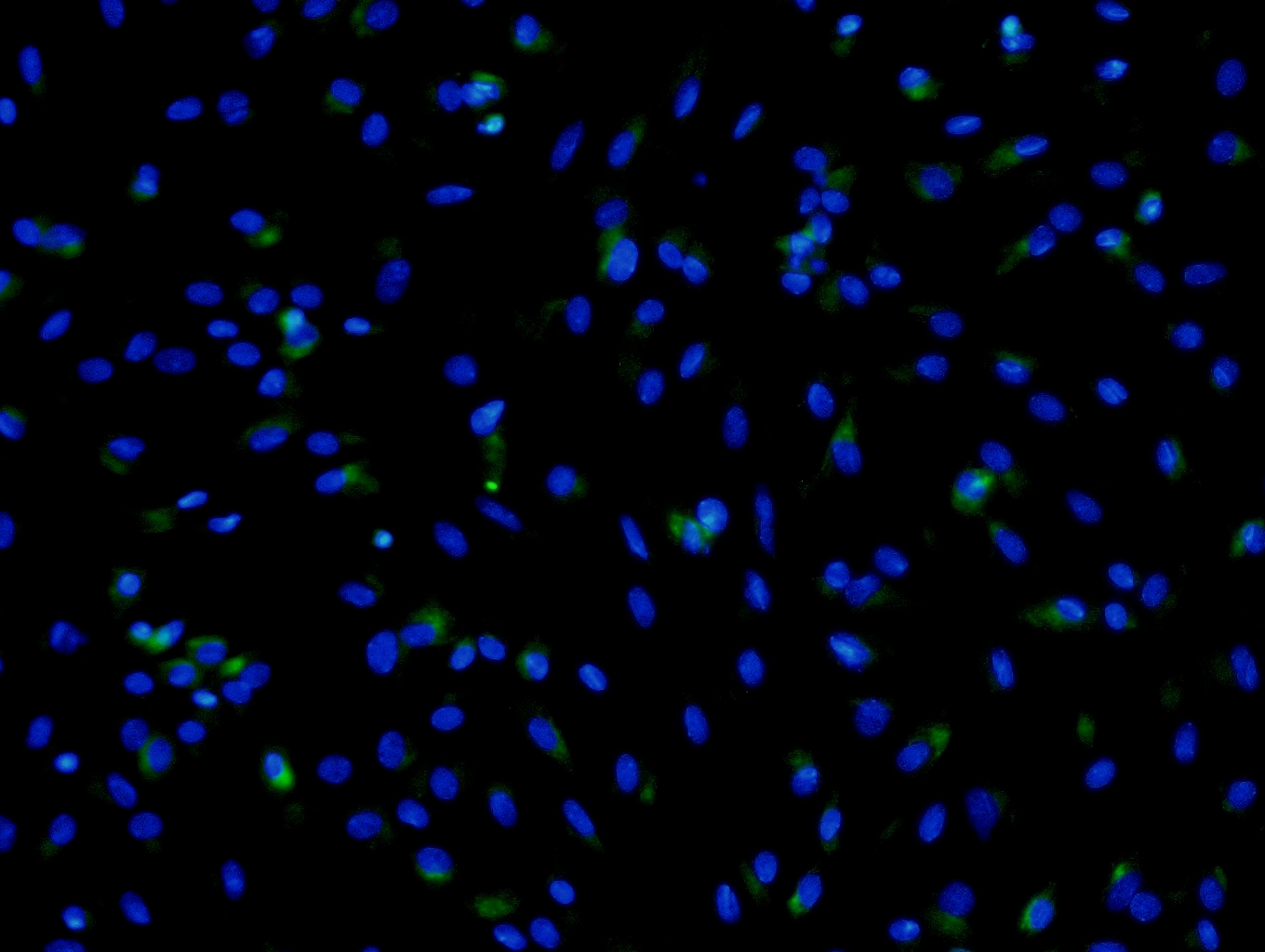

Supplement: Supplementary file 6 — Source data Fig. 4 [file 44318_2024_220_MOESM6_ESM.zip › Figure4/4I/TNFRSF14-+NK-2 (1).jpg]

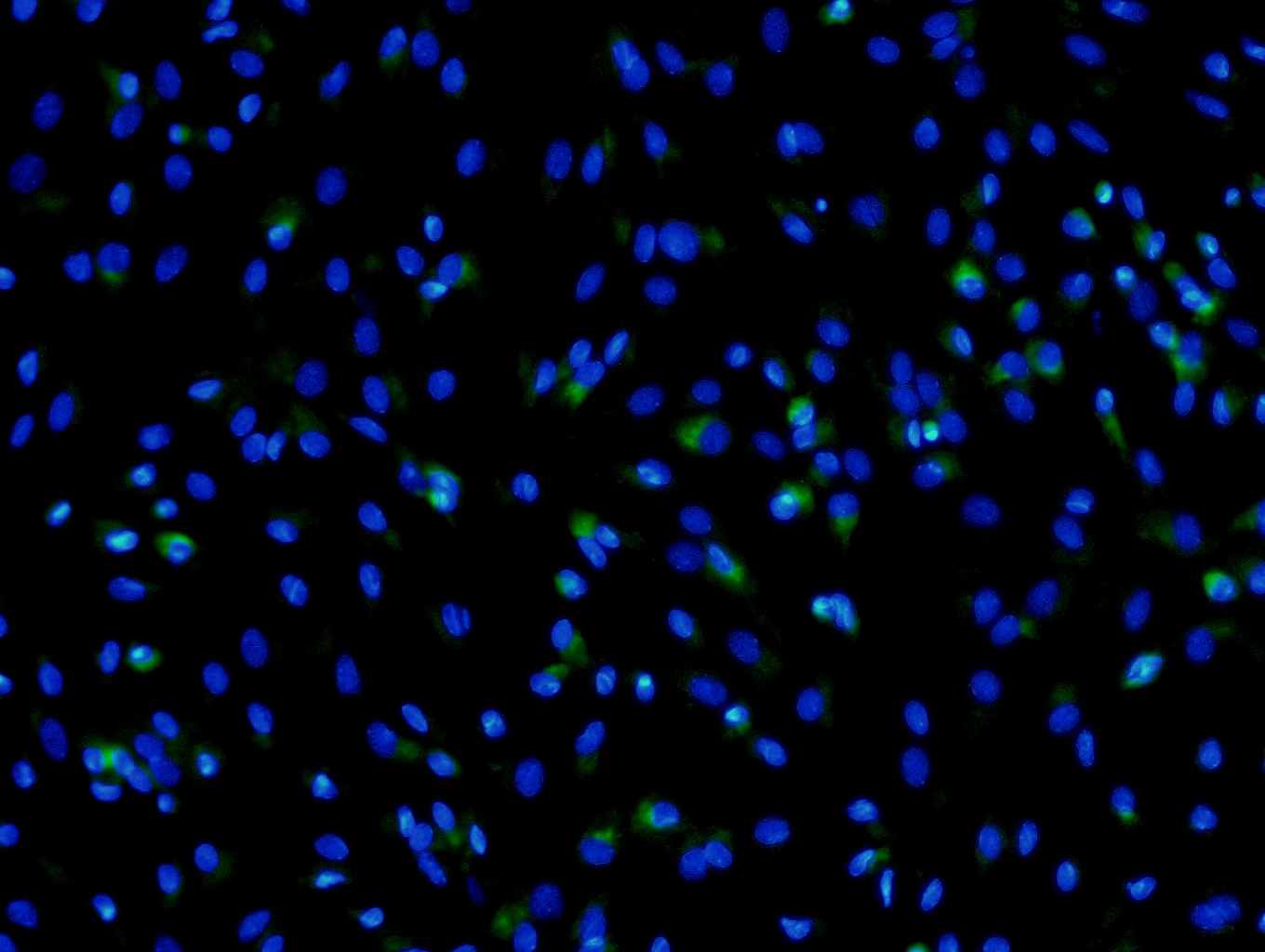

Supplement: Supplementary file 6 — Source data Fig. 4 [file 44318_2024_220_MOESM6_ESM.zip › Figure4/4I/TNFRSF14-+NK-2 (2).jpg]

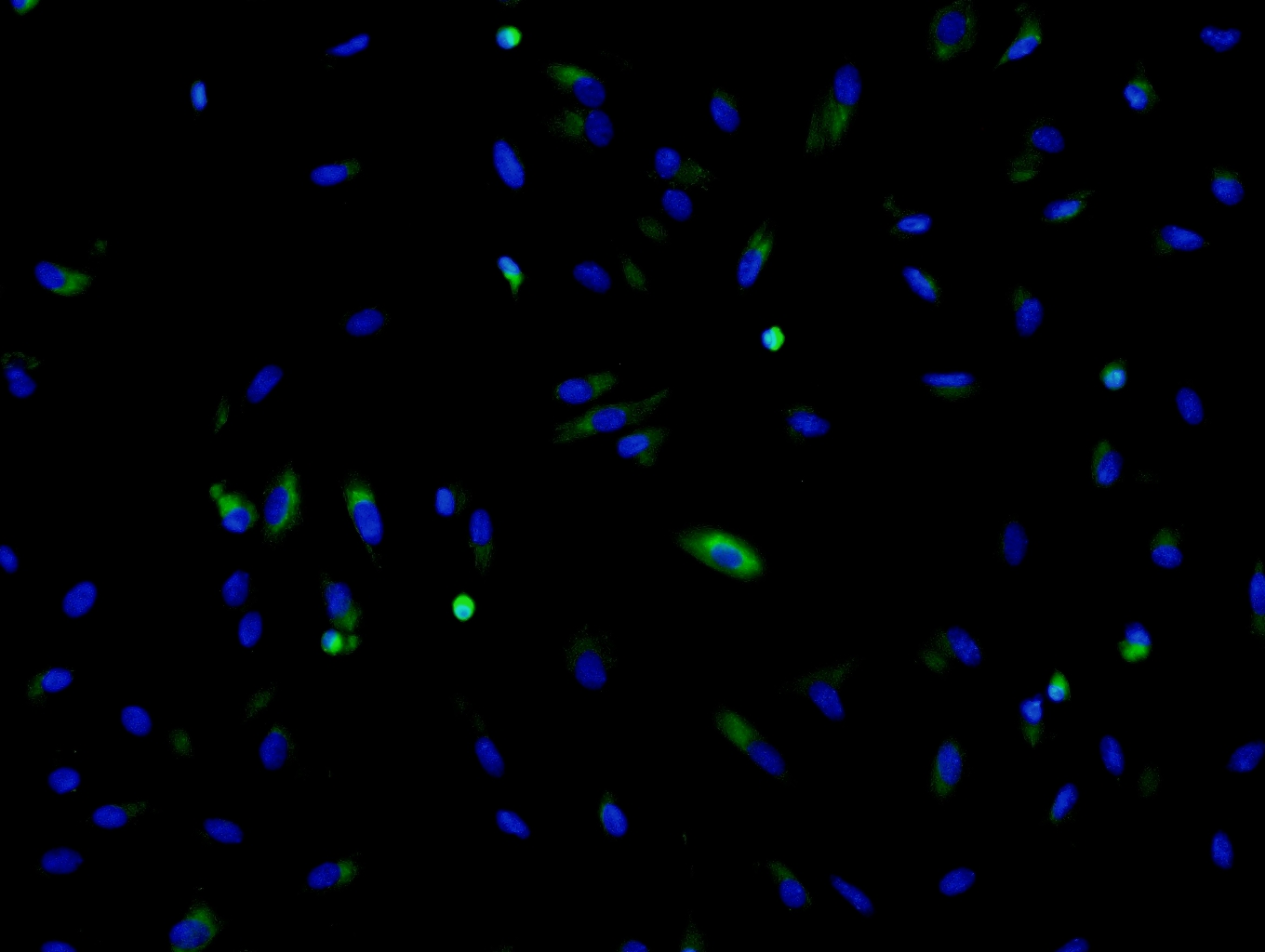

Supplement: Supplementary file 6 — Source data Fig. 4 [file 44318_2024_220_MOESM6_ESM.zip › Figure4/4I/TNFRSF14-+NK-2 (3).jpg]

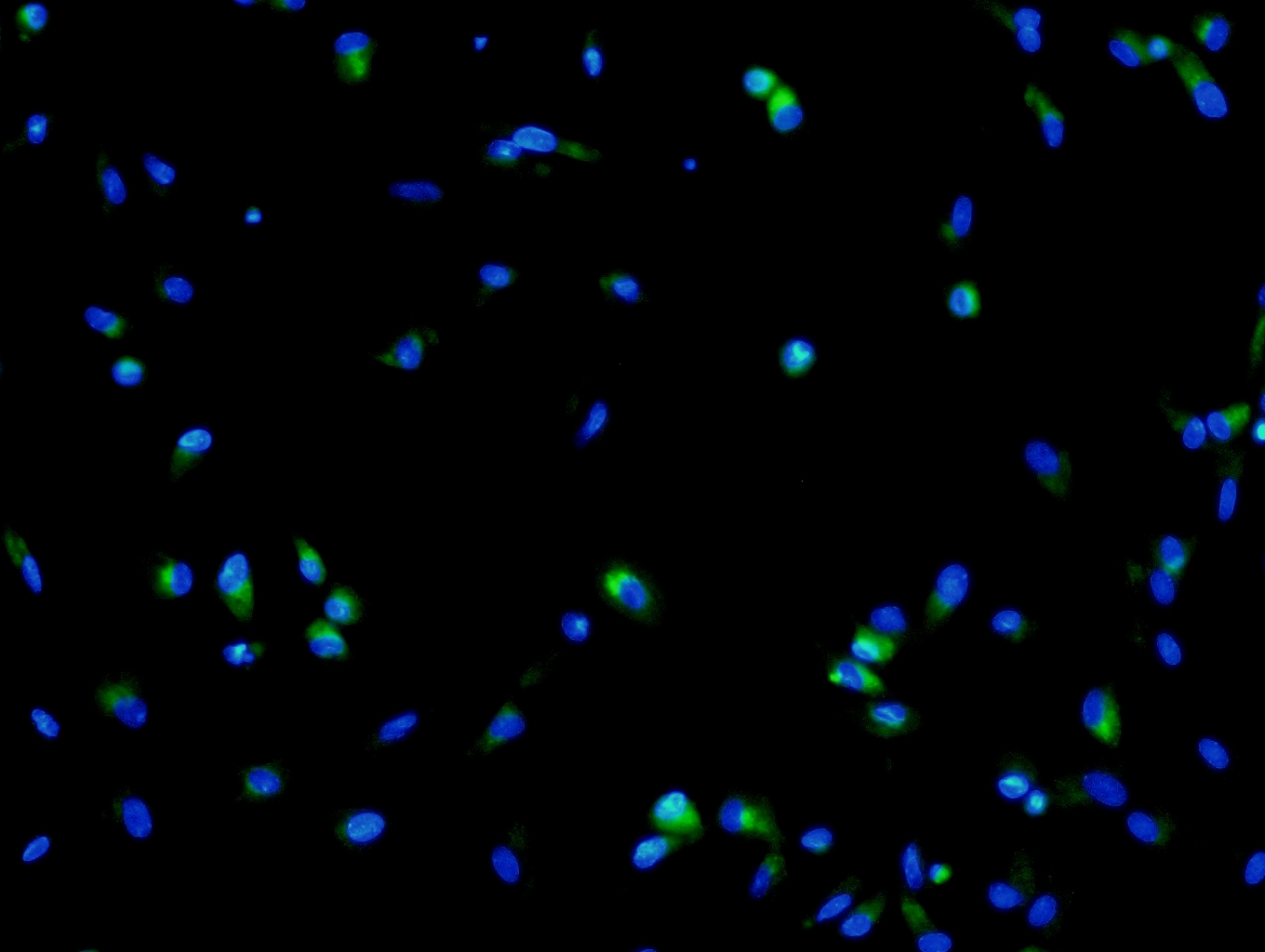

Supplement: Supplementary file 6 — Source data Fig. 4 [file 44318_2024_220_MOESM6_ESM.zip › Figure4/4I/TNFRSF14-+NK-3 (1).jpg]

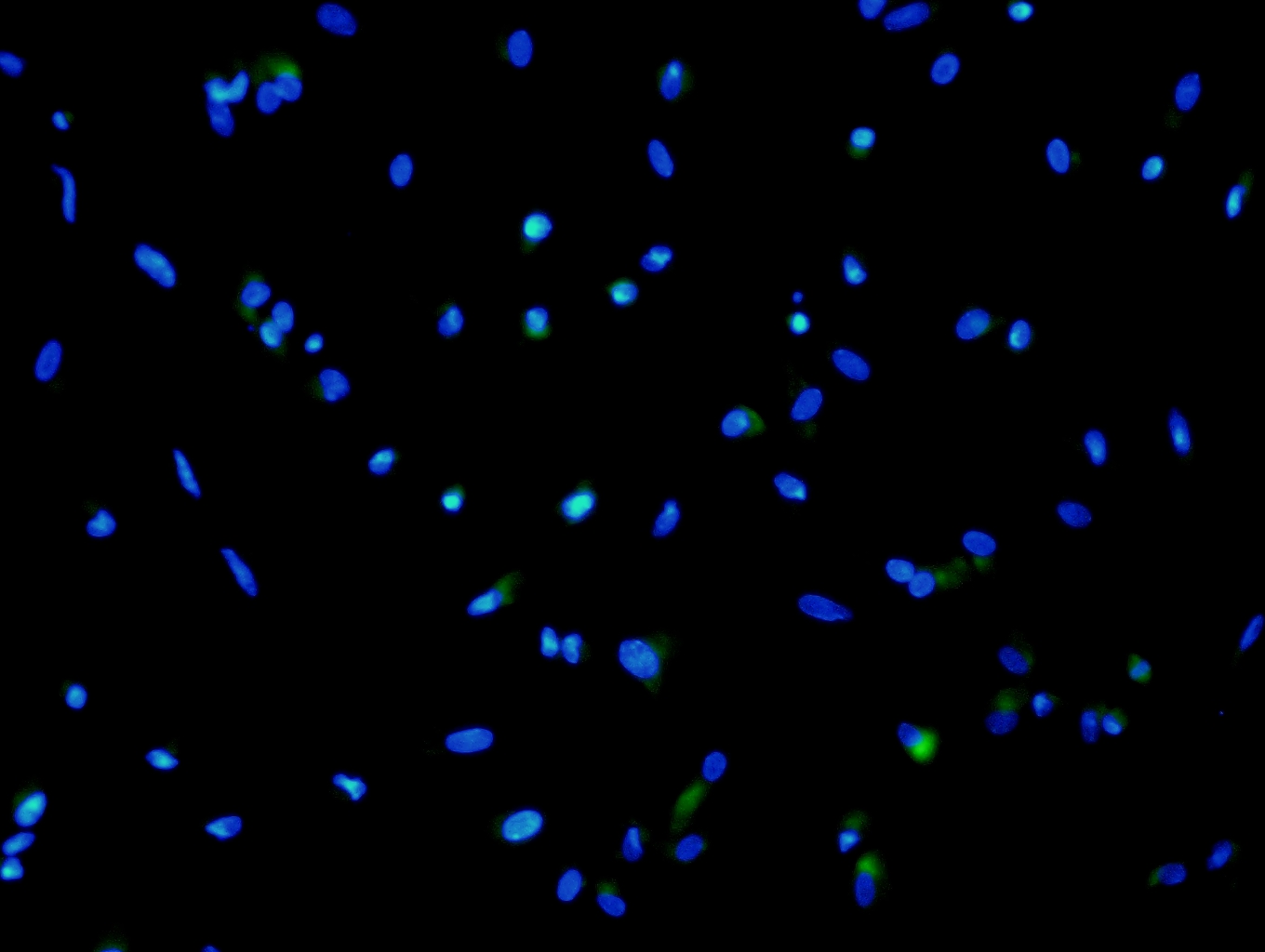

Supplement: Supplementary file 6 — Source data Fig. 4 [file 44318_2024_220_MOESM6_ESM.zip › Figure4/4I/TNFRSF14-+NK-3 (2).jpg]

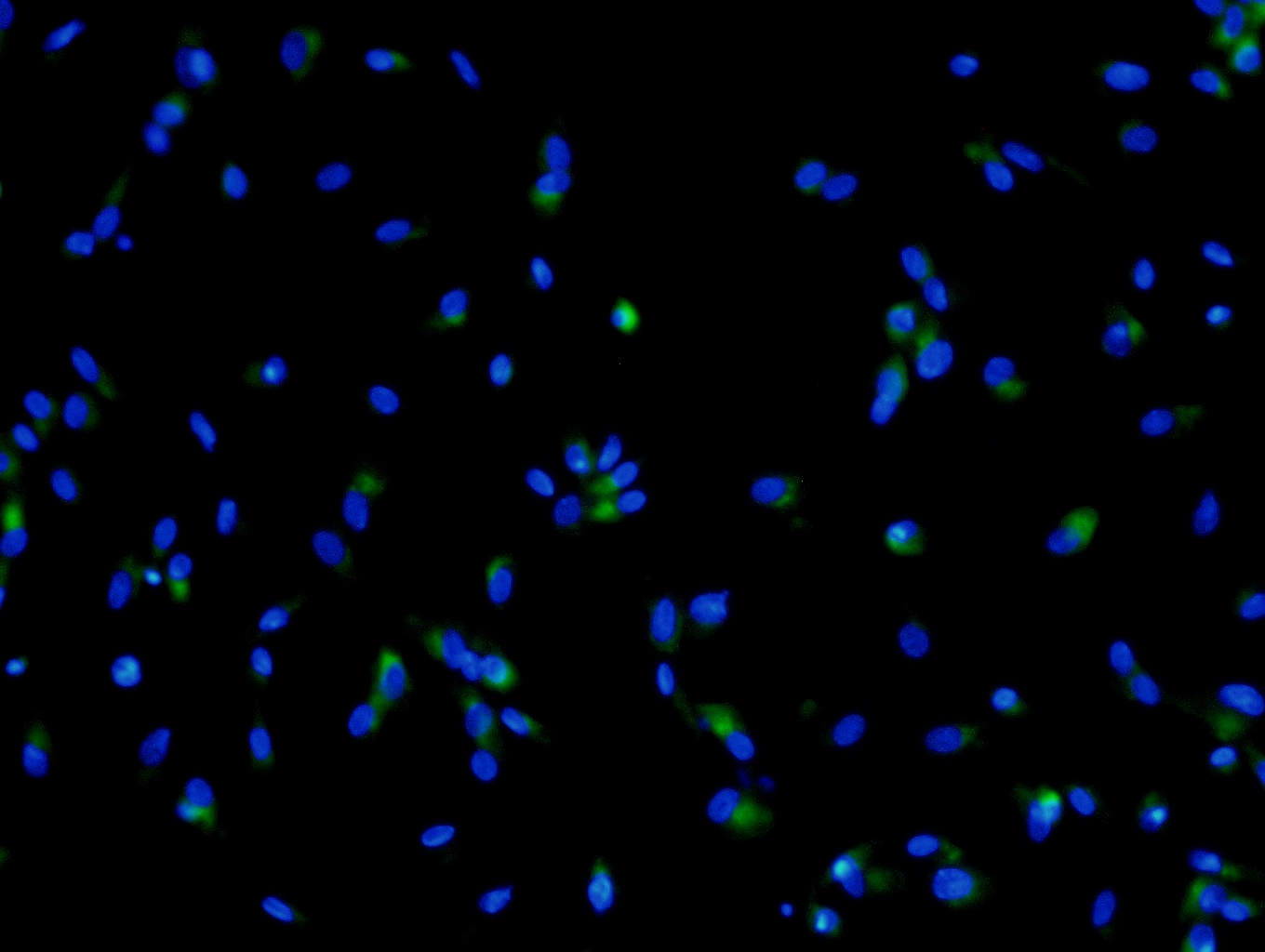

Supplement: Supplementary file 6 — Source data Fig. 4 [file 44318_2024_220_MOESM6_ESM.zip › Figure4/4I/TNFRSF14-+NK-3 (3).jpg]

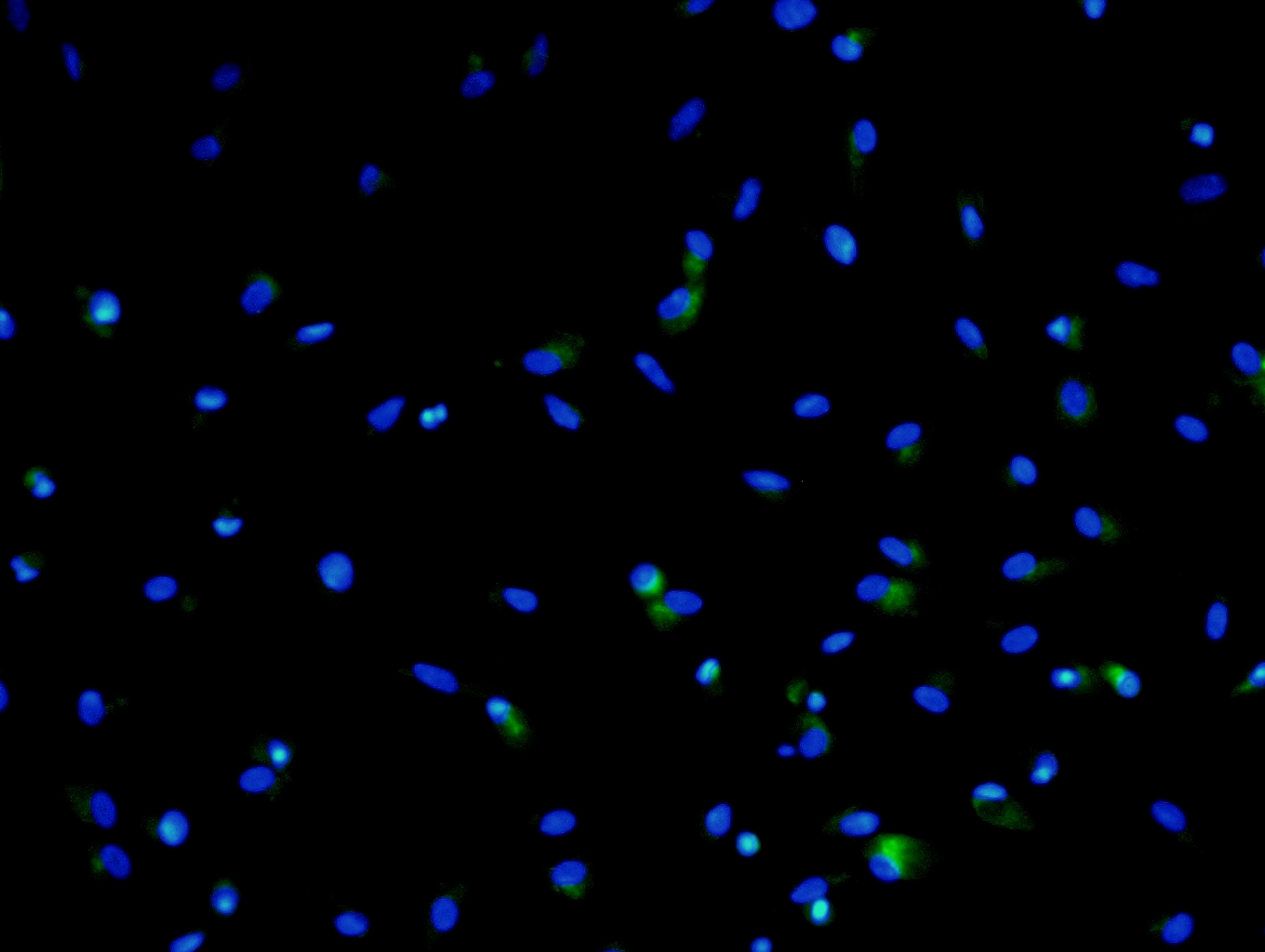

Supplement: Supplementary file 6 — Source data Fig. 4 [file 44318_2024_220_MOESM6_ESM.zip › Figure4/4I/TNFRSF14-+NK-4 (1).jpg]

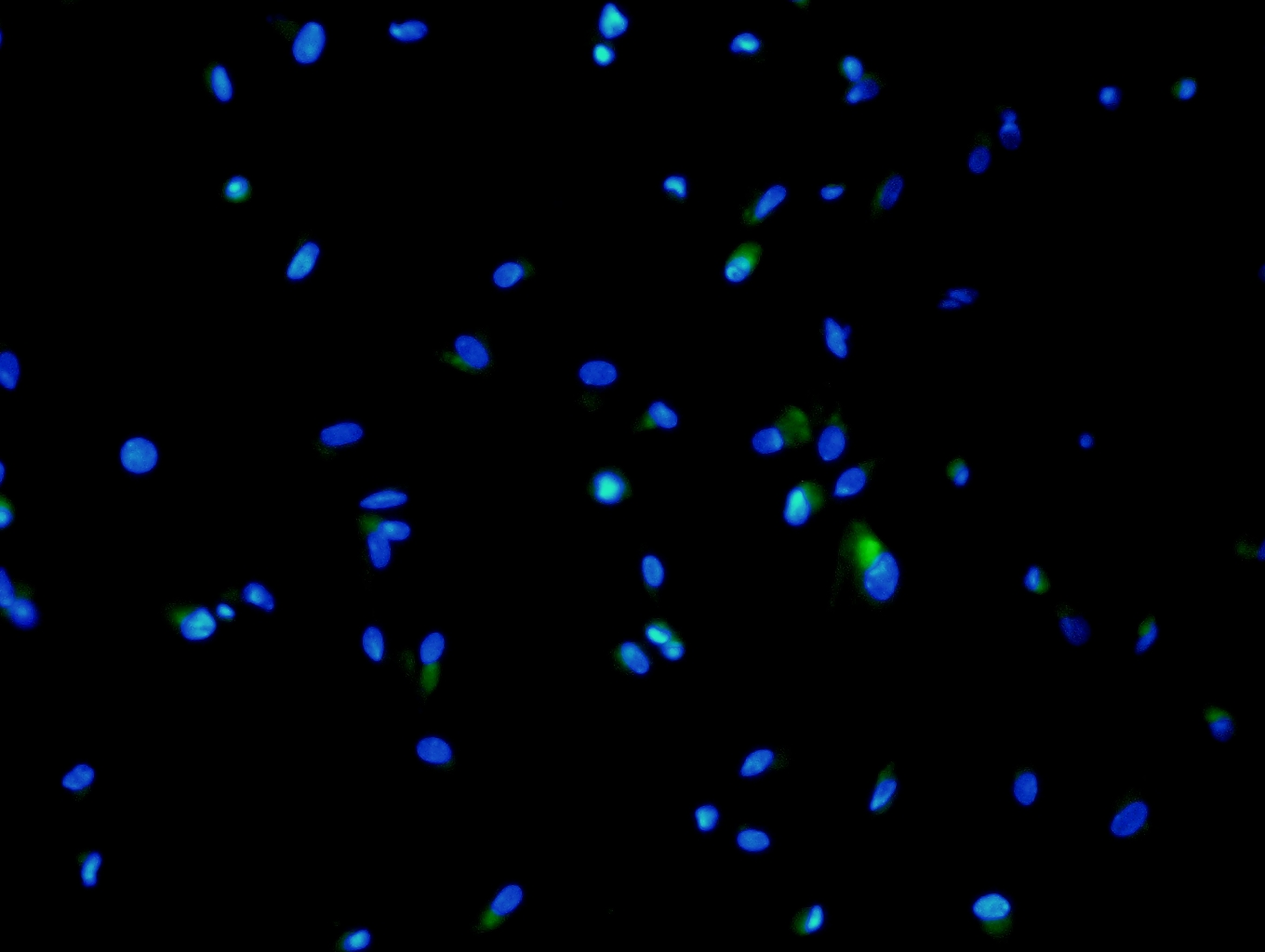

Supplement: Supplementary file 6 — Source data Fig. 4 [file 44318_2024_220_MOESM6_ESM.zip › Figure4/4I/TNFRSF14-+NK-4 (2).jpg]

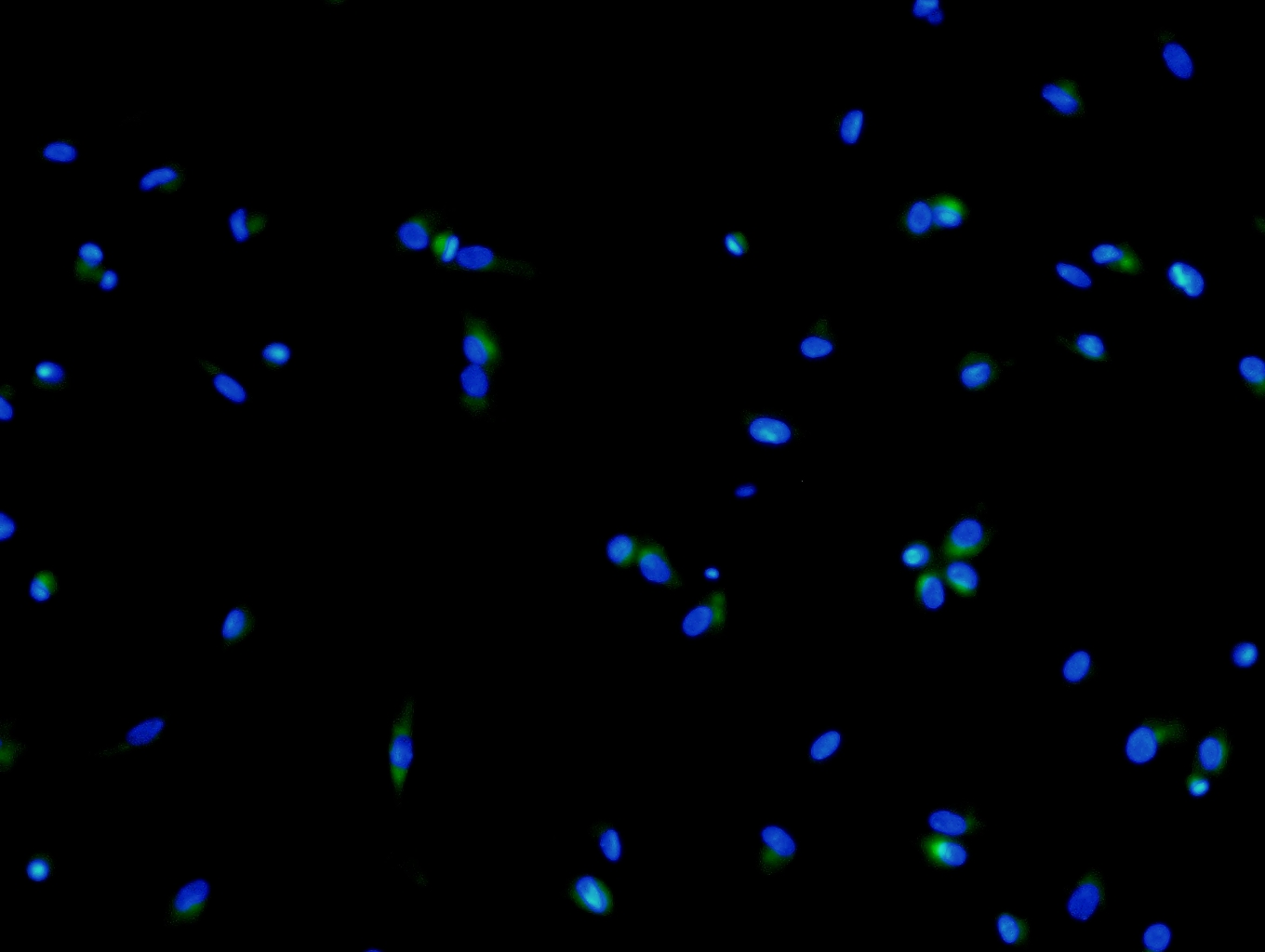

Supplement: Supplementary file 6 — Source data Fig. 4 [file 44318_2024_220_MOESM6_ESM.zip › Figure4/4I/TNFRSF14-+NK-4 (3).jpg]

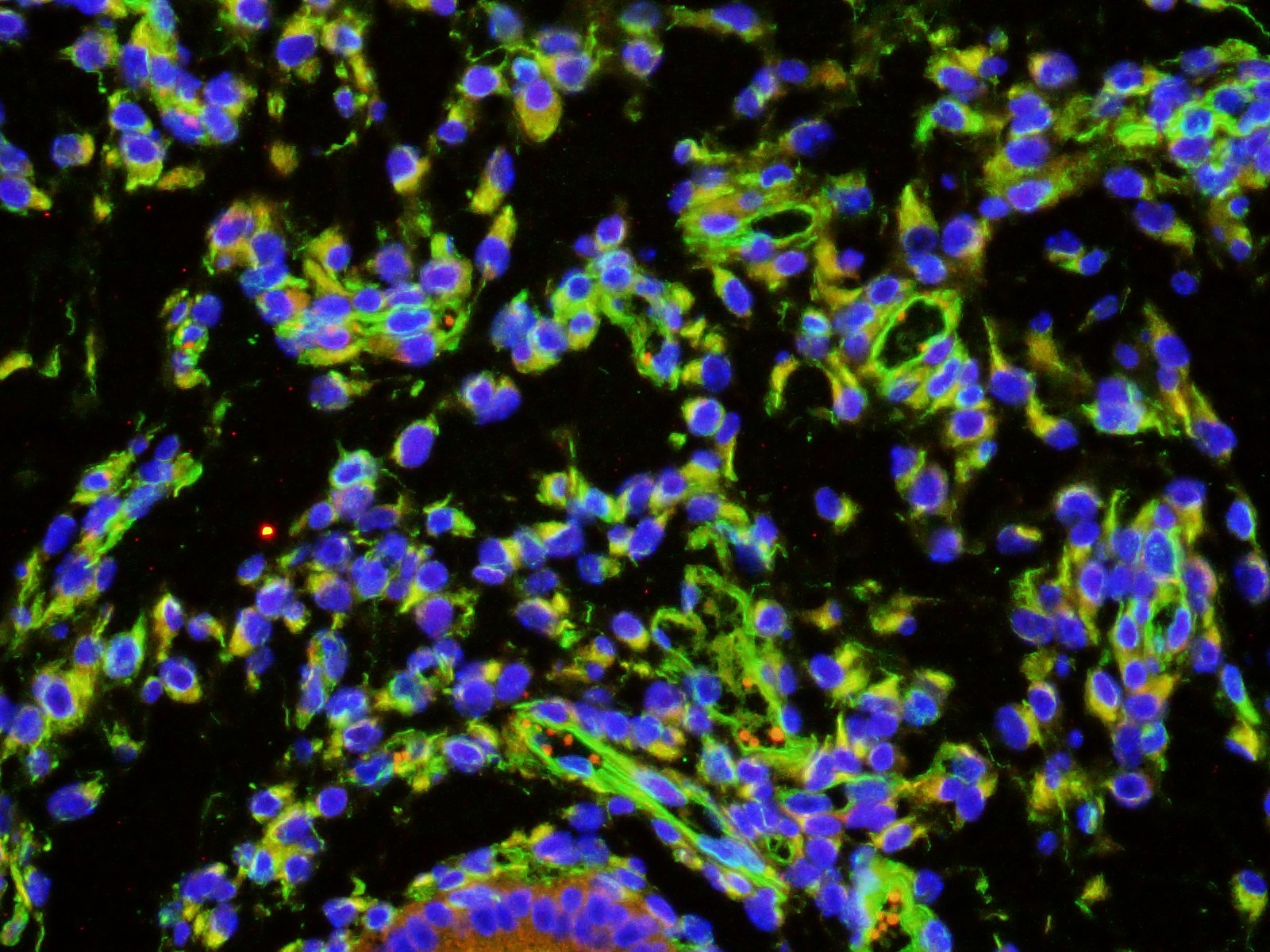

Supplement: Supplementary file 7 — Source data Fig. 5 [file 44318_2024_220_MOESM7_ESM.zip › Figure5/5D/CDKN1A-Tnfrsf14-400 (1).jpg]

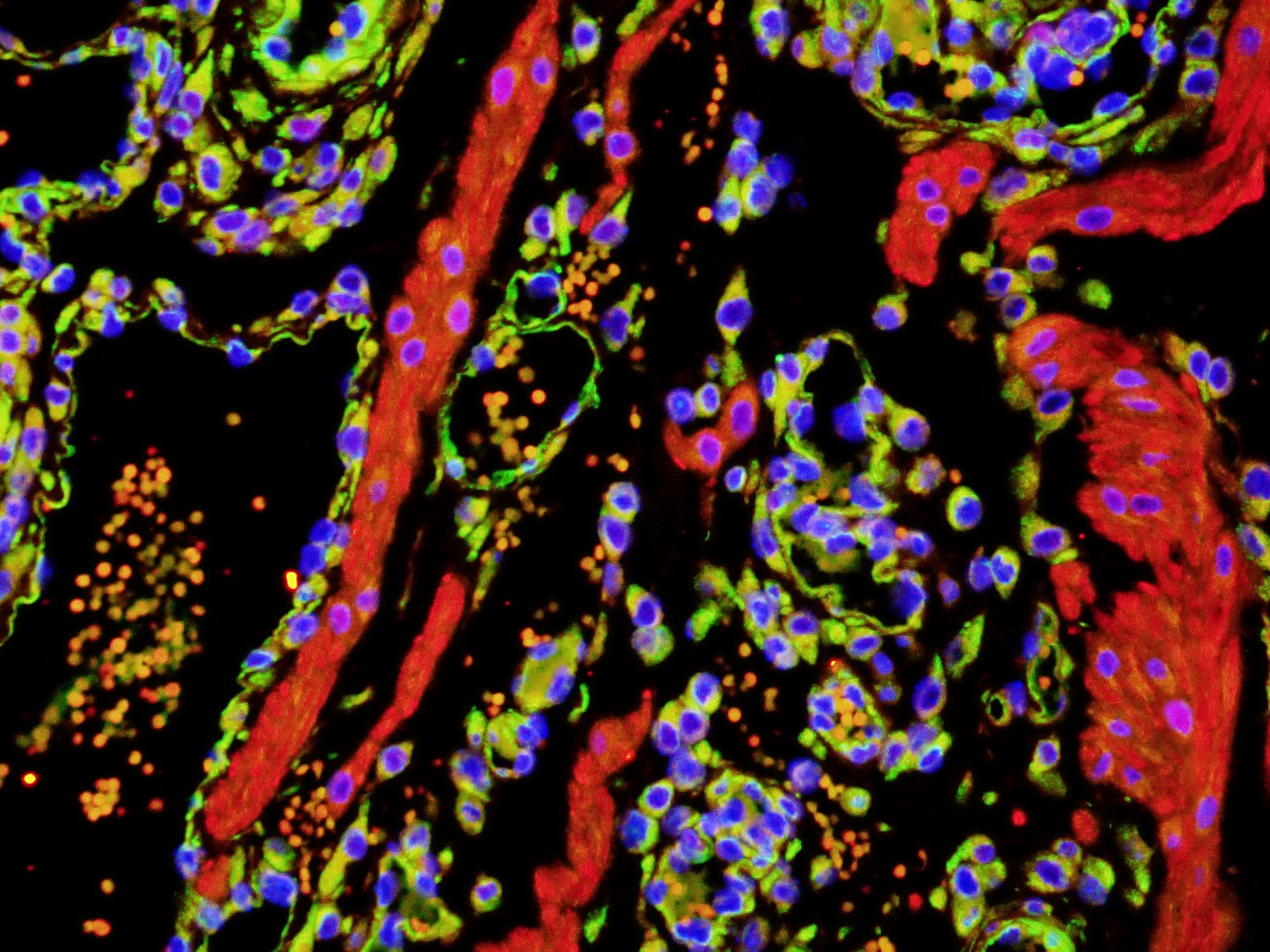

Supplement: Supplementary file 7 — Source data Fig. 5 [file 44318_2024_220_MOESM7_ESM.zip › Figure5/5D/CDKN1A-Tnfrsf14-400 (2).jpg]

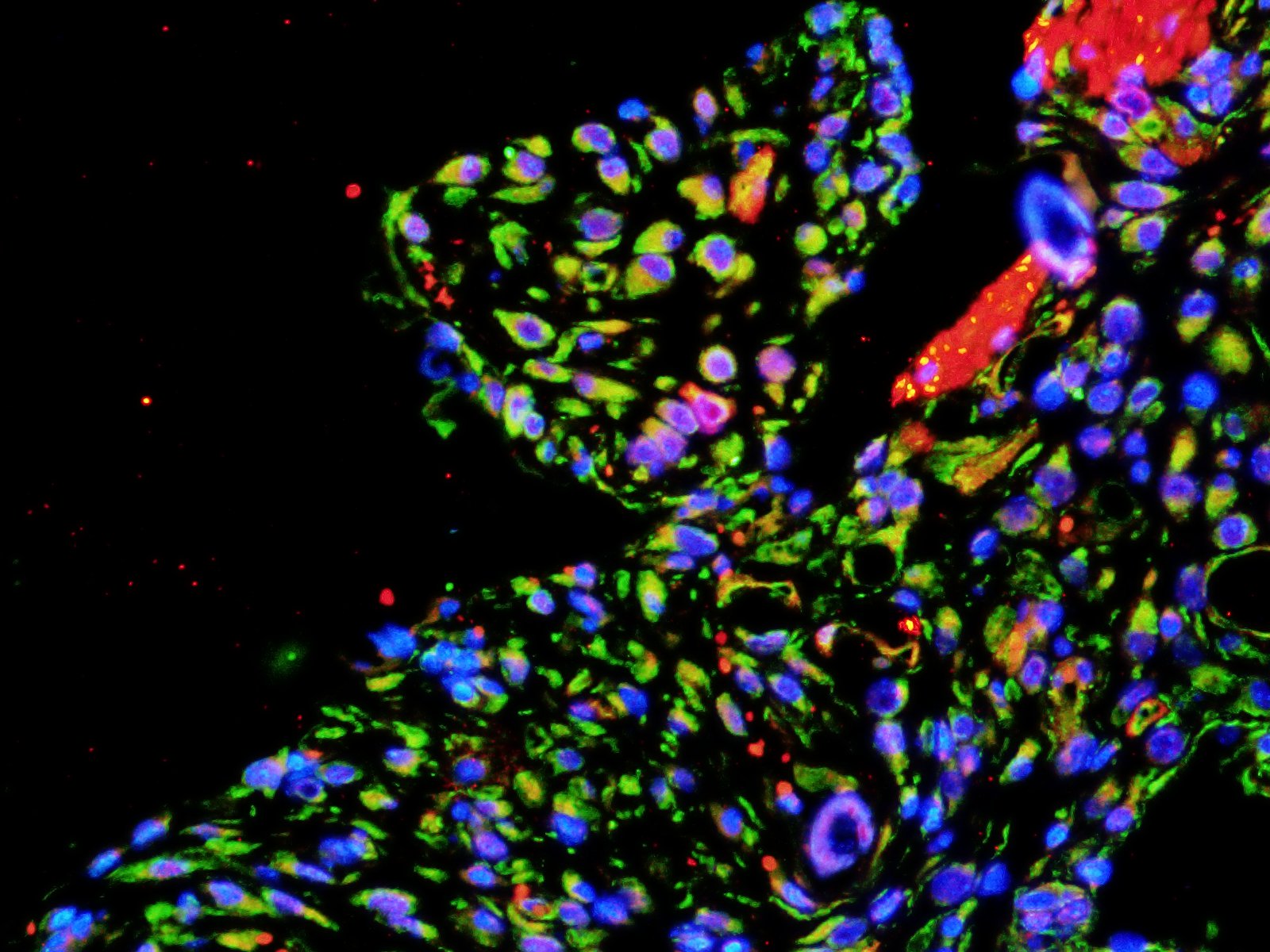

Supplement: Supplementary file 7 — Source data Fig. 5 [file 44318_2024_220_MOESM7_ESM.zip › Figure5/5D/CDKN1A-Tnfrsf14-400 (3).jpg]

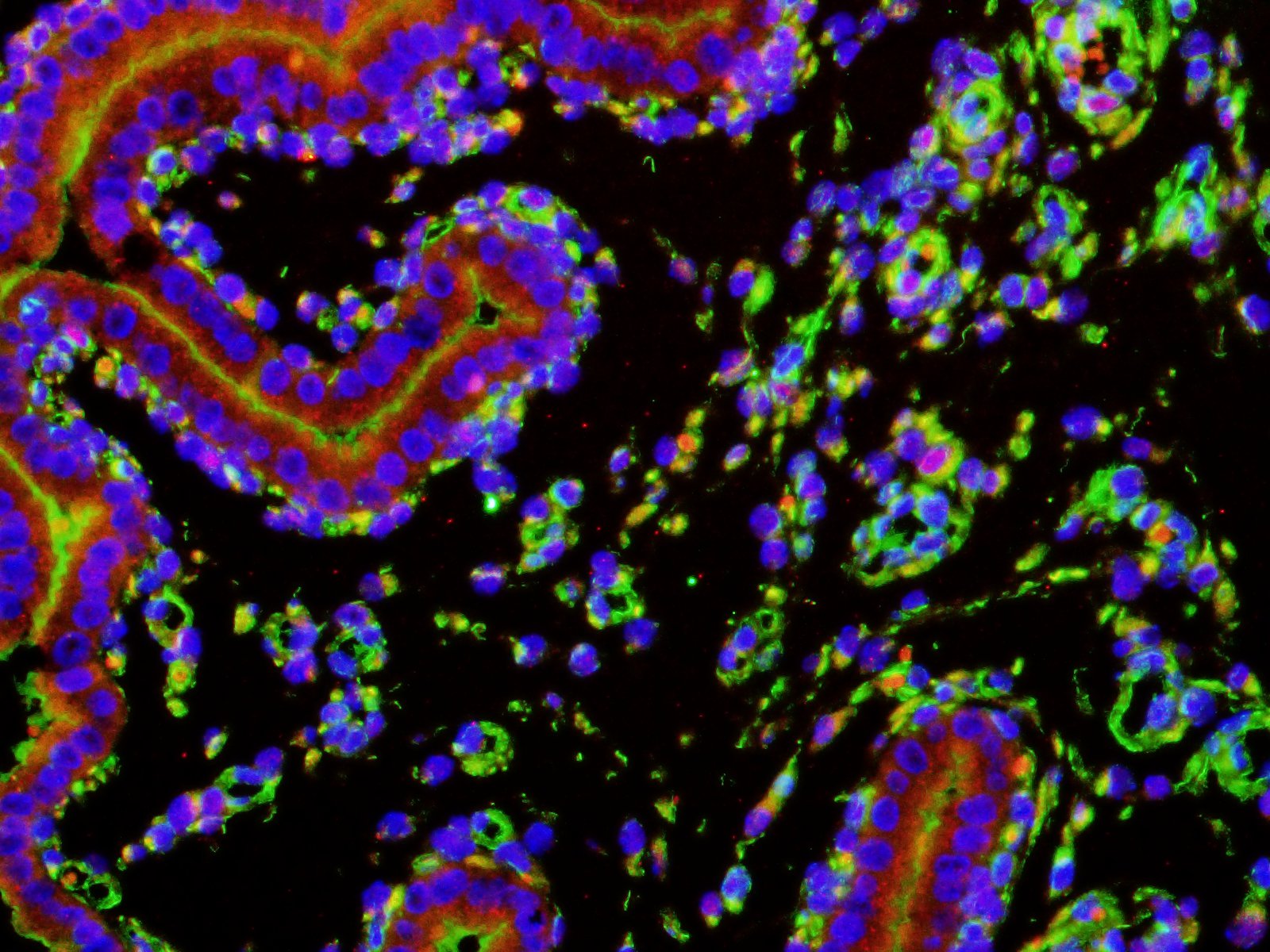

Supplement: Supplementary file 7 — Source data Fig. 5 [file 44318_2024_220_MOESM7_ESM.zip › Figure5/5D/CDKN1A-Tnfrsf14-400 (4).jpg]

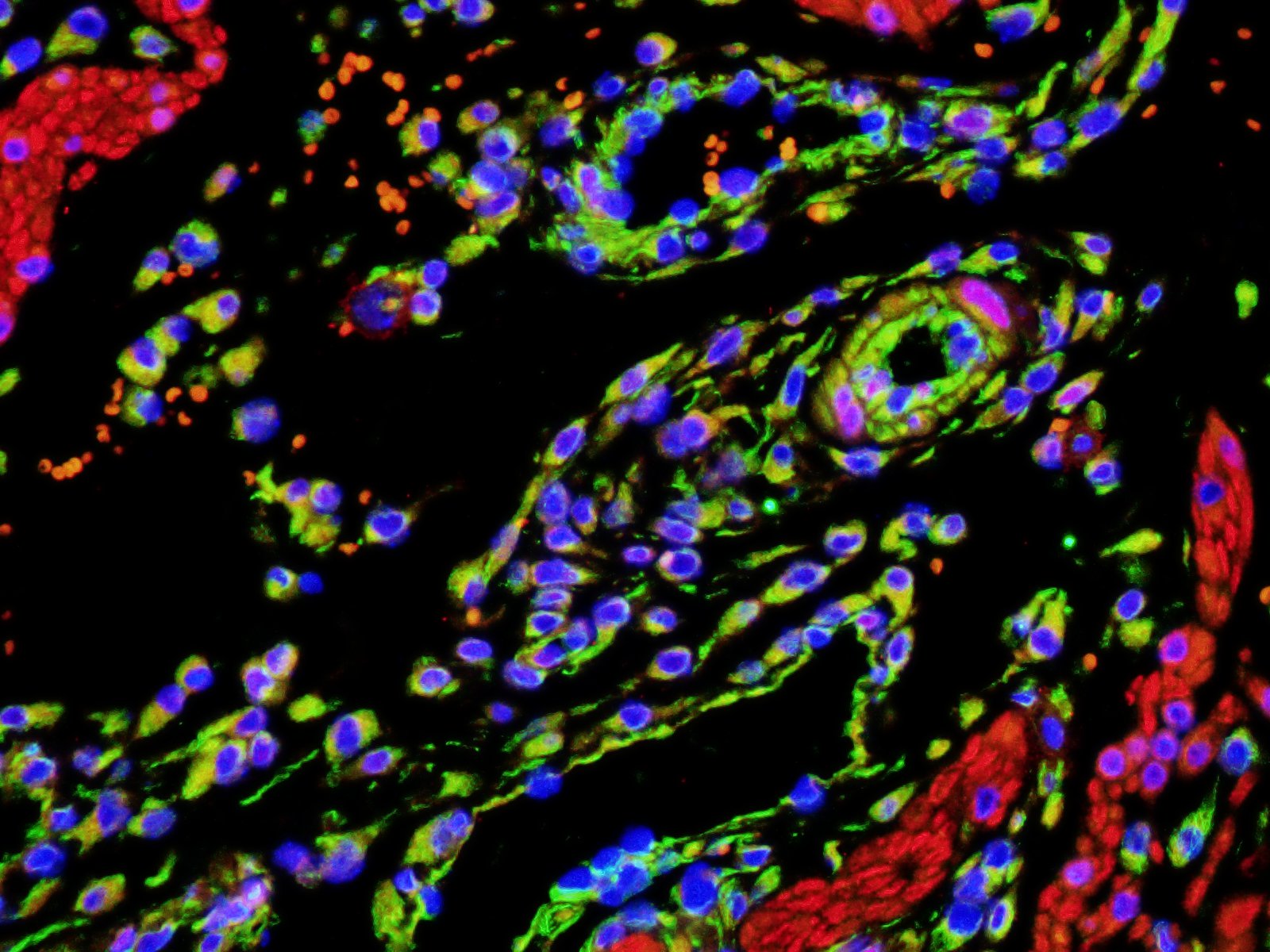

Supplement: Supplementary file 7 — Source data Fig. 5 [file 44318_2024_220_MOESM7_ESM.zip › Figure5/5D/CDKN1A-Tnfrsf14-400 (5).jpg]

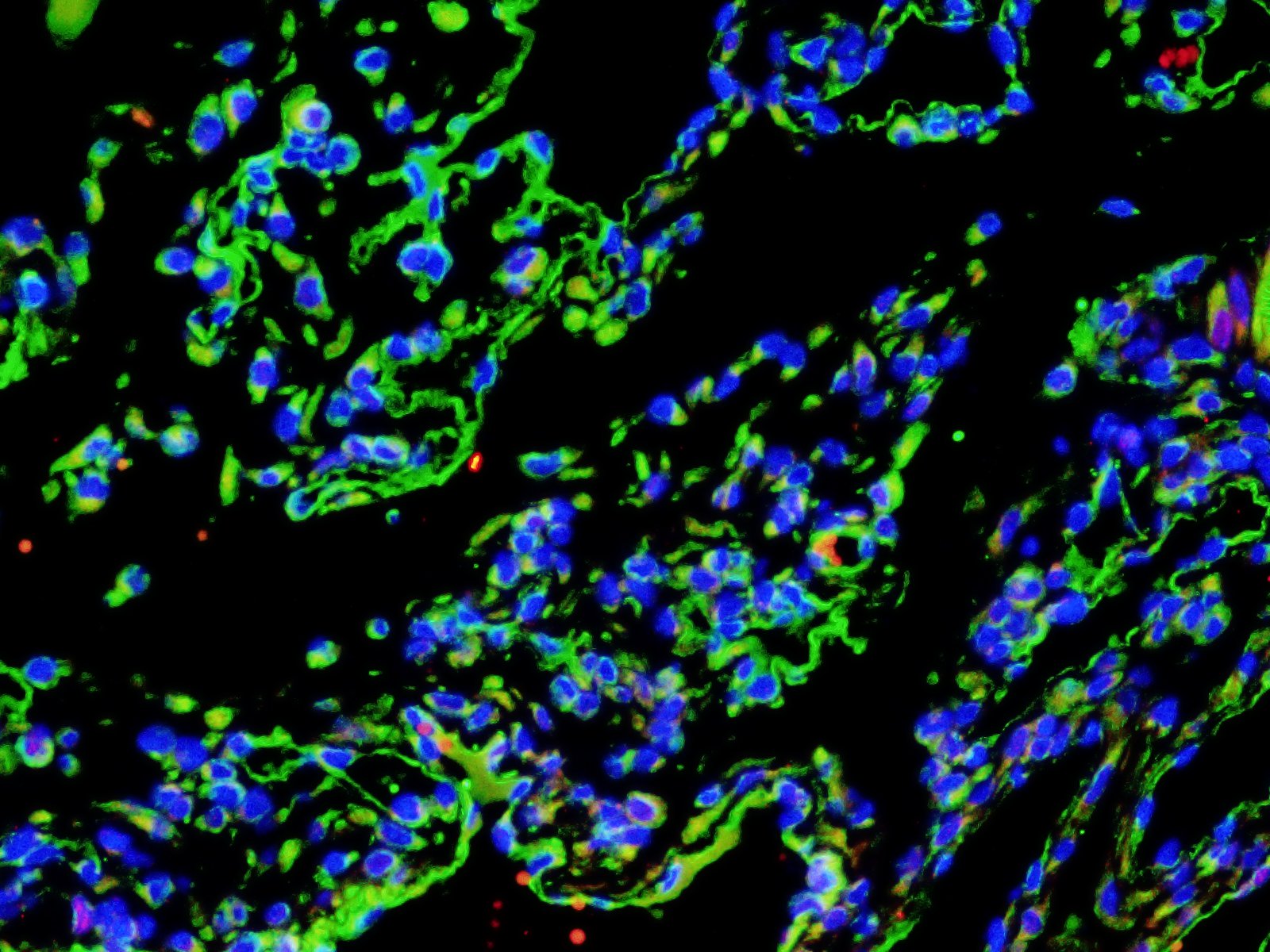

Supplement: Supplementary file 7 — Source data Fig. 5 [file 44318_2024_220_MOESM7_ESM.zip › Figure5/5D/CDKN1A-WT-400 (1).jpg]

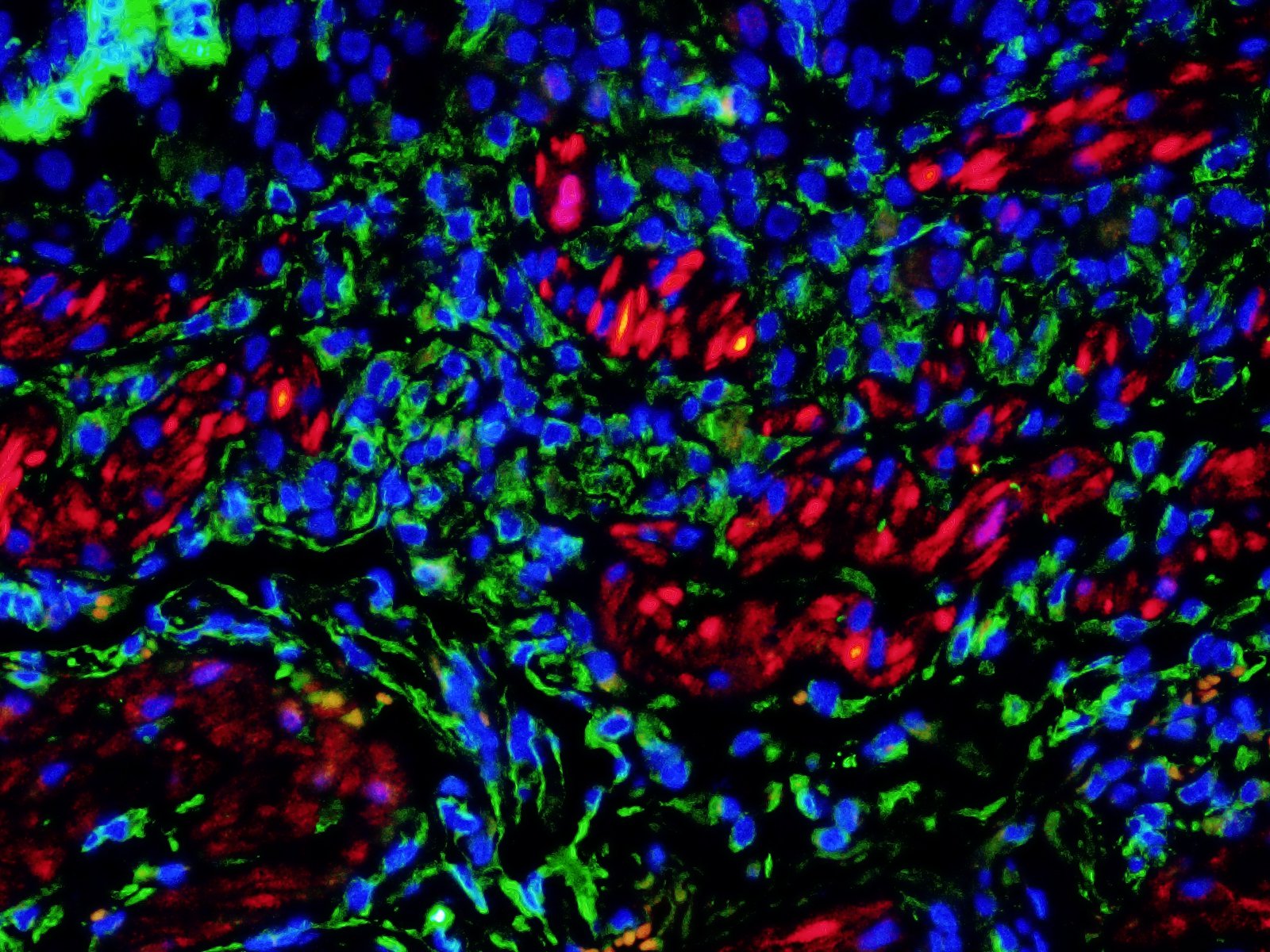

Supplement: Supplementary file 7 — Source data Fig. 5 [file 44318_2024_220_MOESM7_ESM.zip › Figure5/5D/CDKN1A-WT-400 (2).jpg]

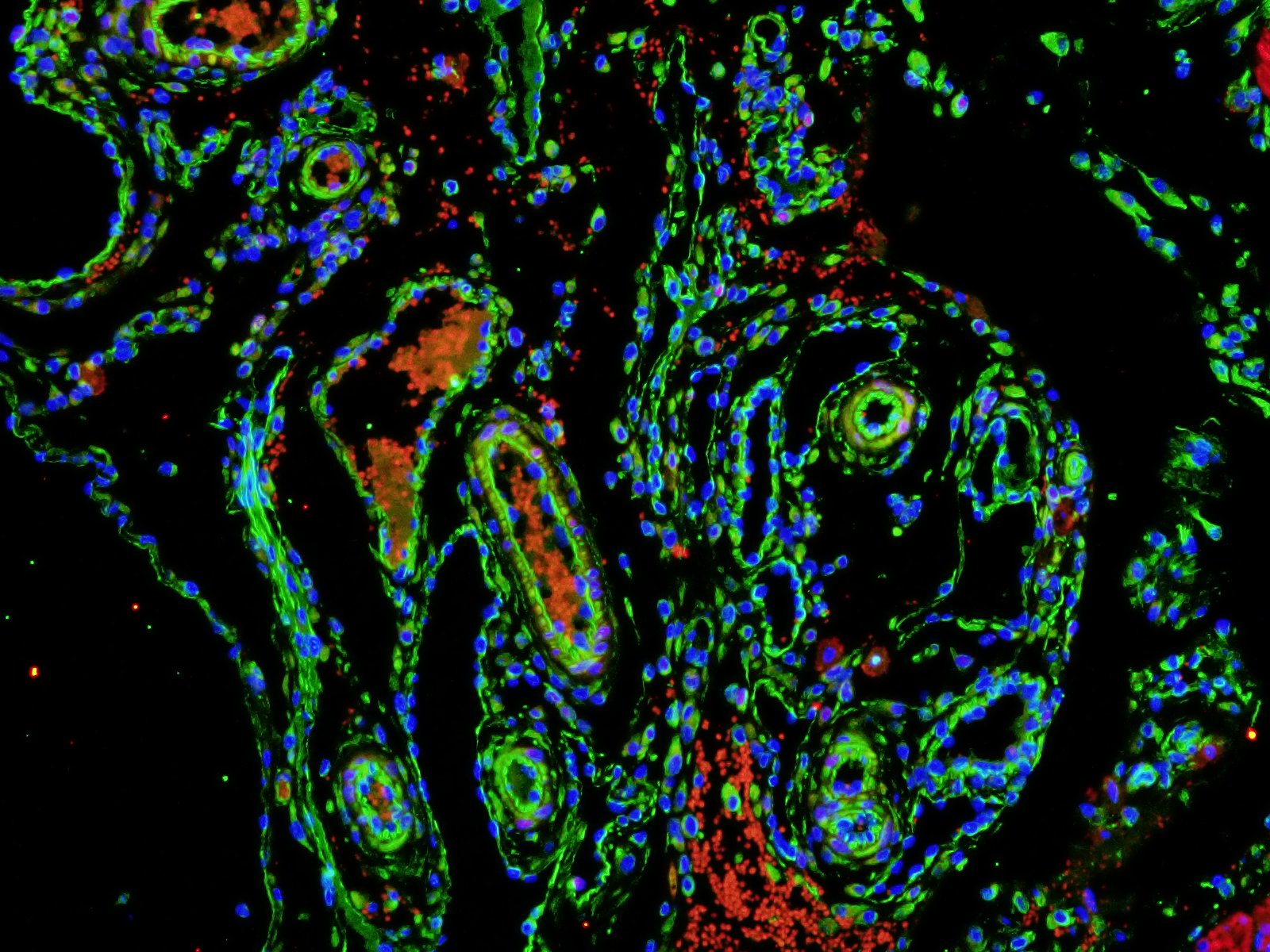

Supplement: Supplementary file 7 — Source data Fig. 5 [file 44318_2024_220_MOESM7_ESM.zip › Figure5/5D/CDKN1A-WT-400 (3).jpg]

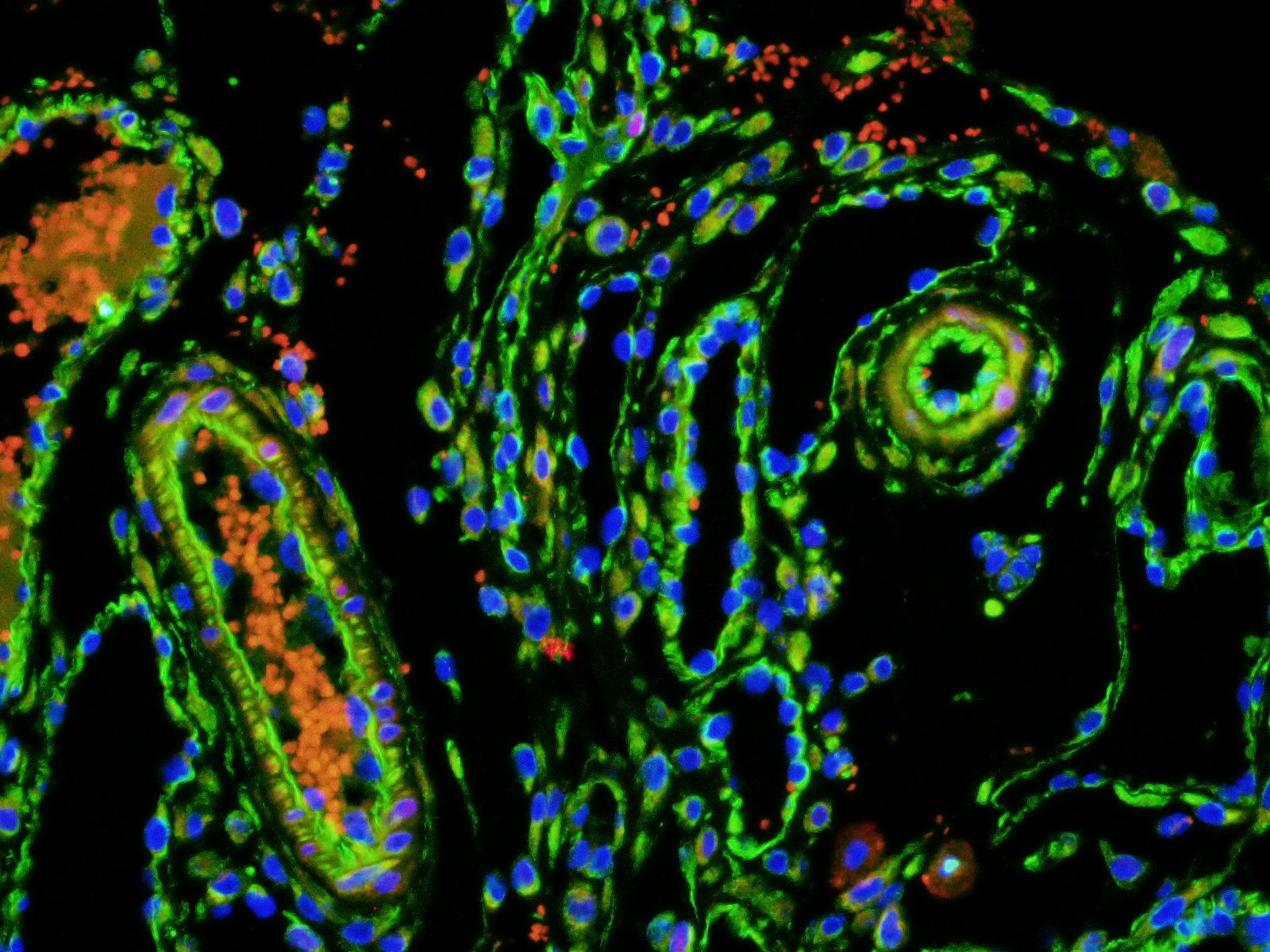

Supplement: Supplementary file 7 — Source data Fig. 5 [file 44318_2024_220_MOESM7_ESM.zip › Figure5/5D/CDKN1A-WT-400 (4).jpg]

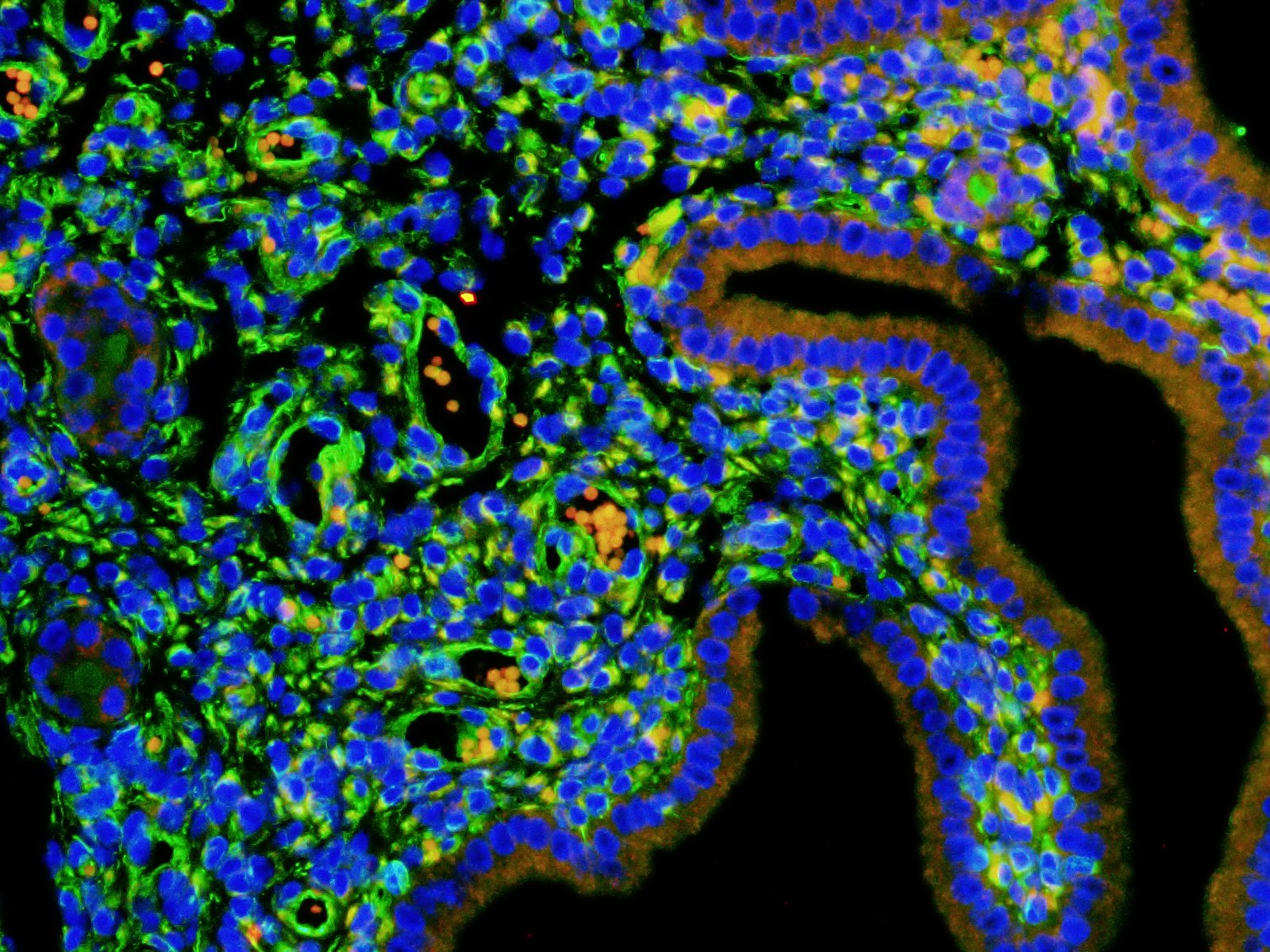

Supplement: Supplementary file 7 — Source data Fig. 5 [file 44318_2024_220_MOESM7_ESM.zip › Figure5/5D/CDKN1A-WT-400 (5).jpg]

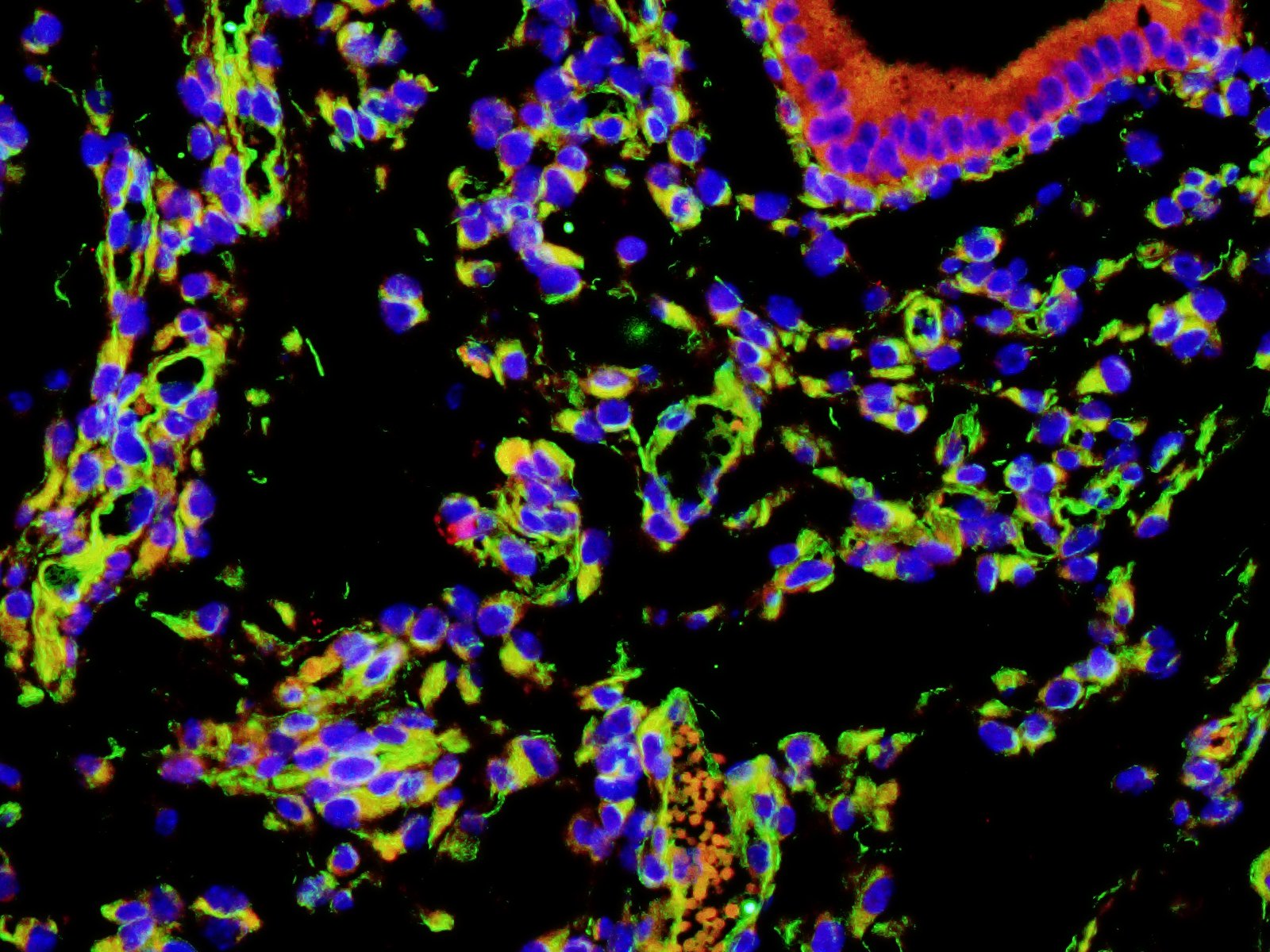

Supplement: Supplementary file 7 — Source data Fig. 5 [file 44318_2024_220_MOESM7_ESM.zip › Figure5/5D/CDKN2A-Tnfrsf14-400 (1).jpg]

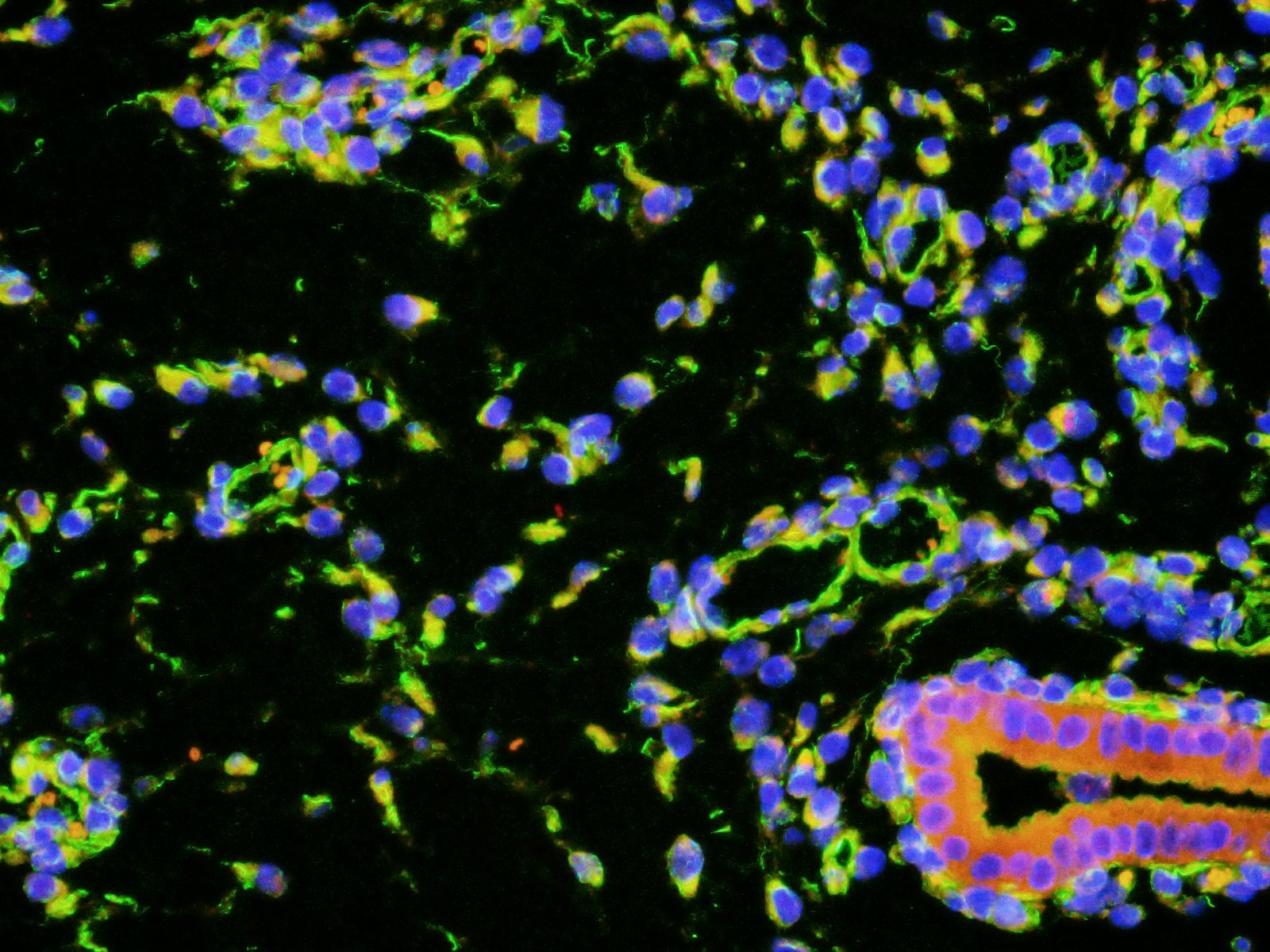

Supplement: Supplementary file 7 — Source data Fig. 5 [file 44318_2024_220_MOESM7_ESM.zip › Figure5/5D/CDKN2A-Tnfrsf14-400 (2).jpg]

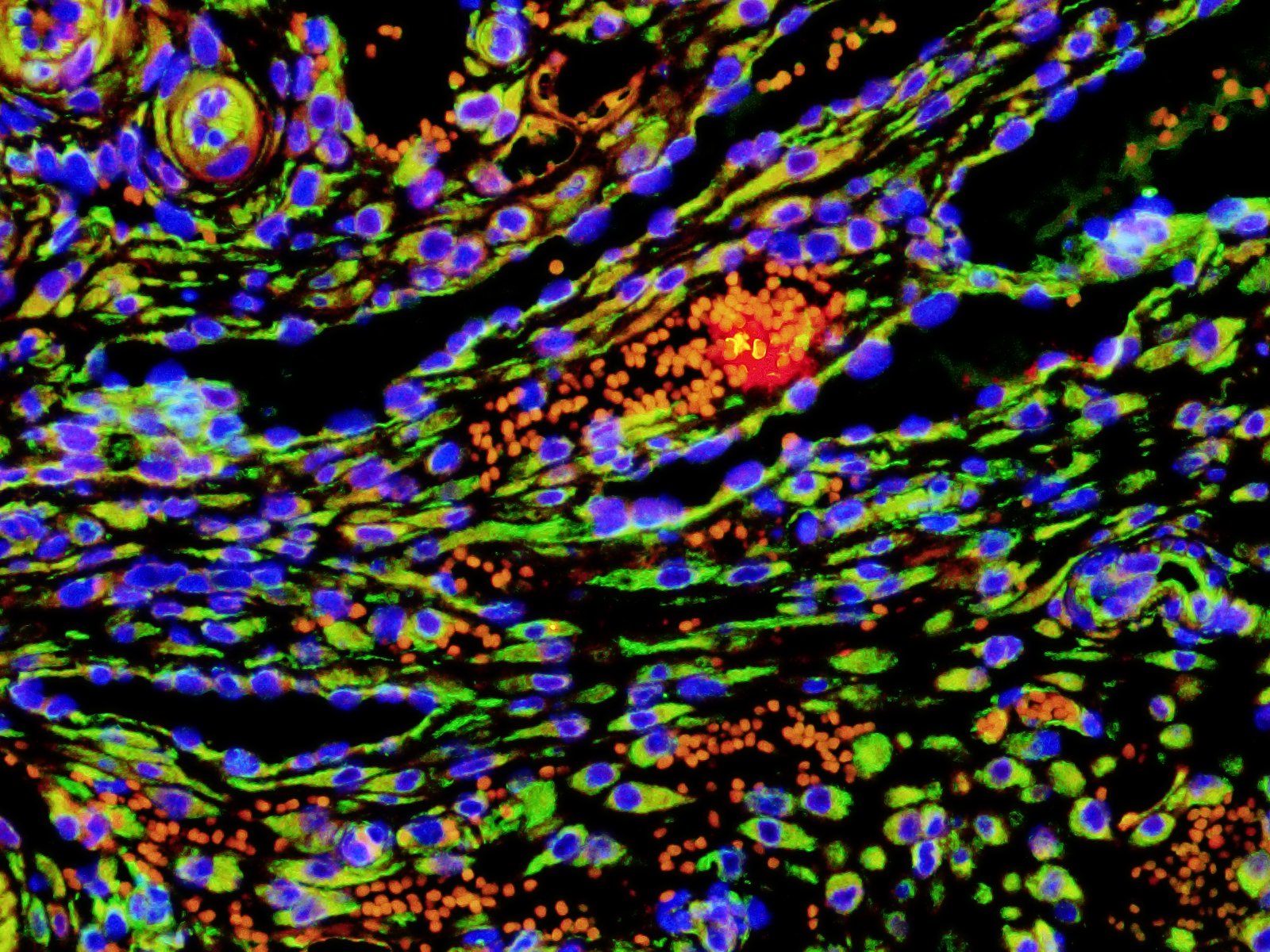

Supplement: Supplementary file 7 — Source data Fig. 5 [file 44318_2024_220_MOESM7_ESM.zip › Figure5/5D/CDKN2A-Tnfrsf14-400 (3).jpg]

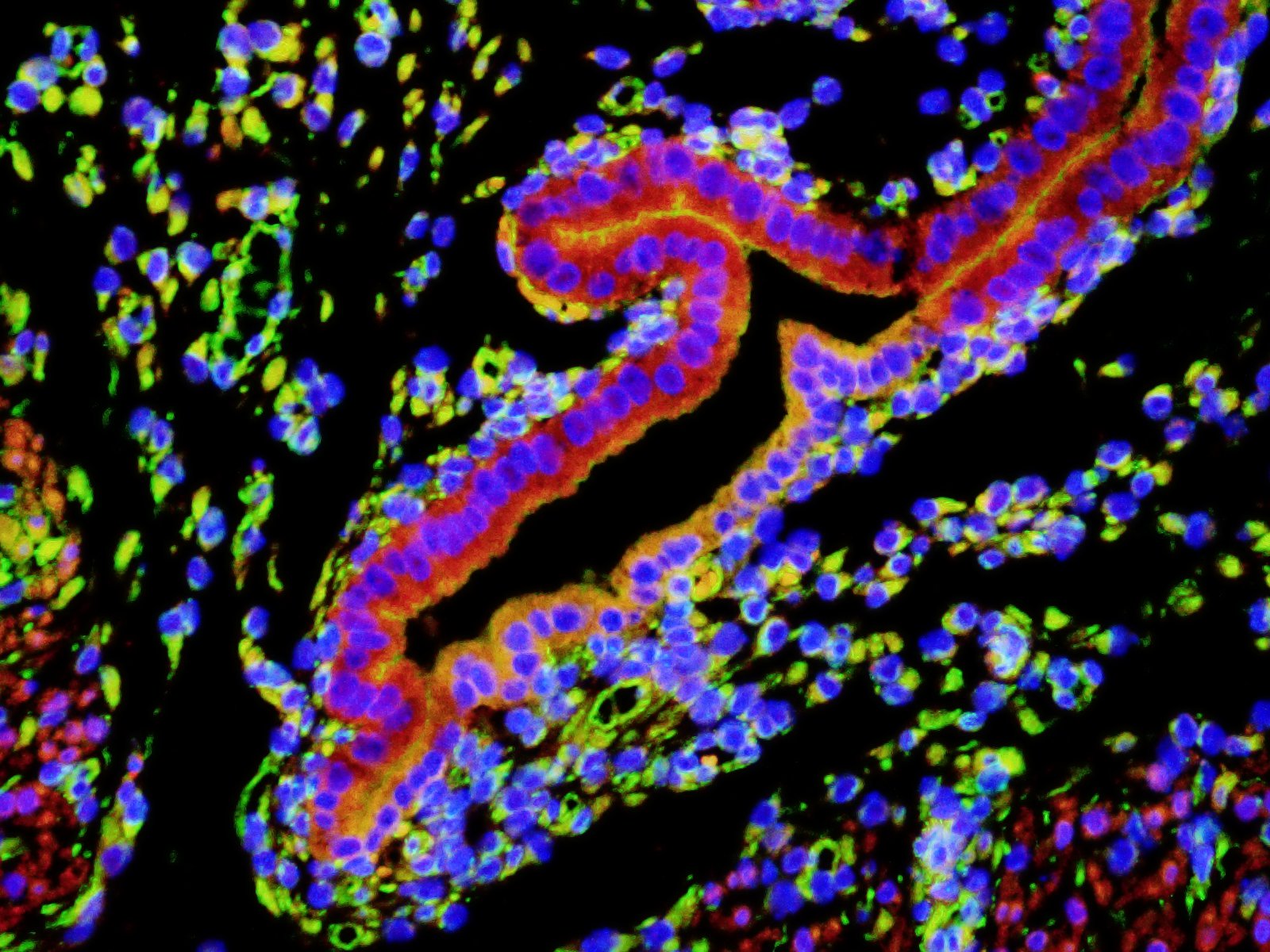

Supplement: Supplementary file 7 — Source data Fig. 5 [file 44318_2024_220_MOESM7_ESM.zip › Figure5/5D/CDKN2A-Tnfrsf14-400 (4).jpg]

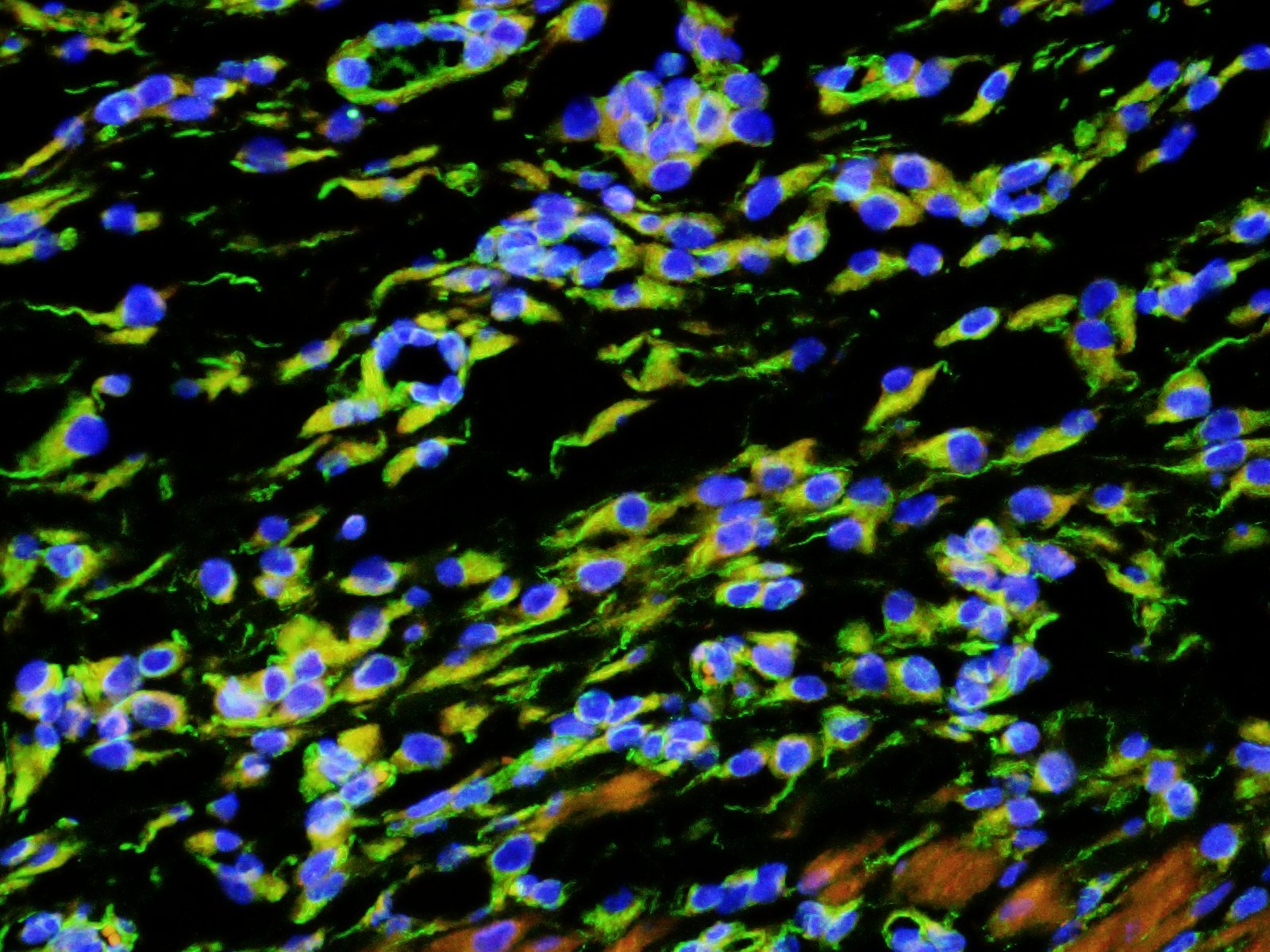

Supplement: Supplementary file 7 — Source data Fig. 5 [file 44318_2024_220_MOESM7_ESM.zip › Figure5/5D/CDKN2A-Tnfrsf14-400 (5).jpg]

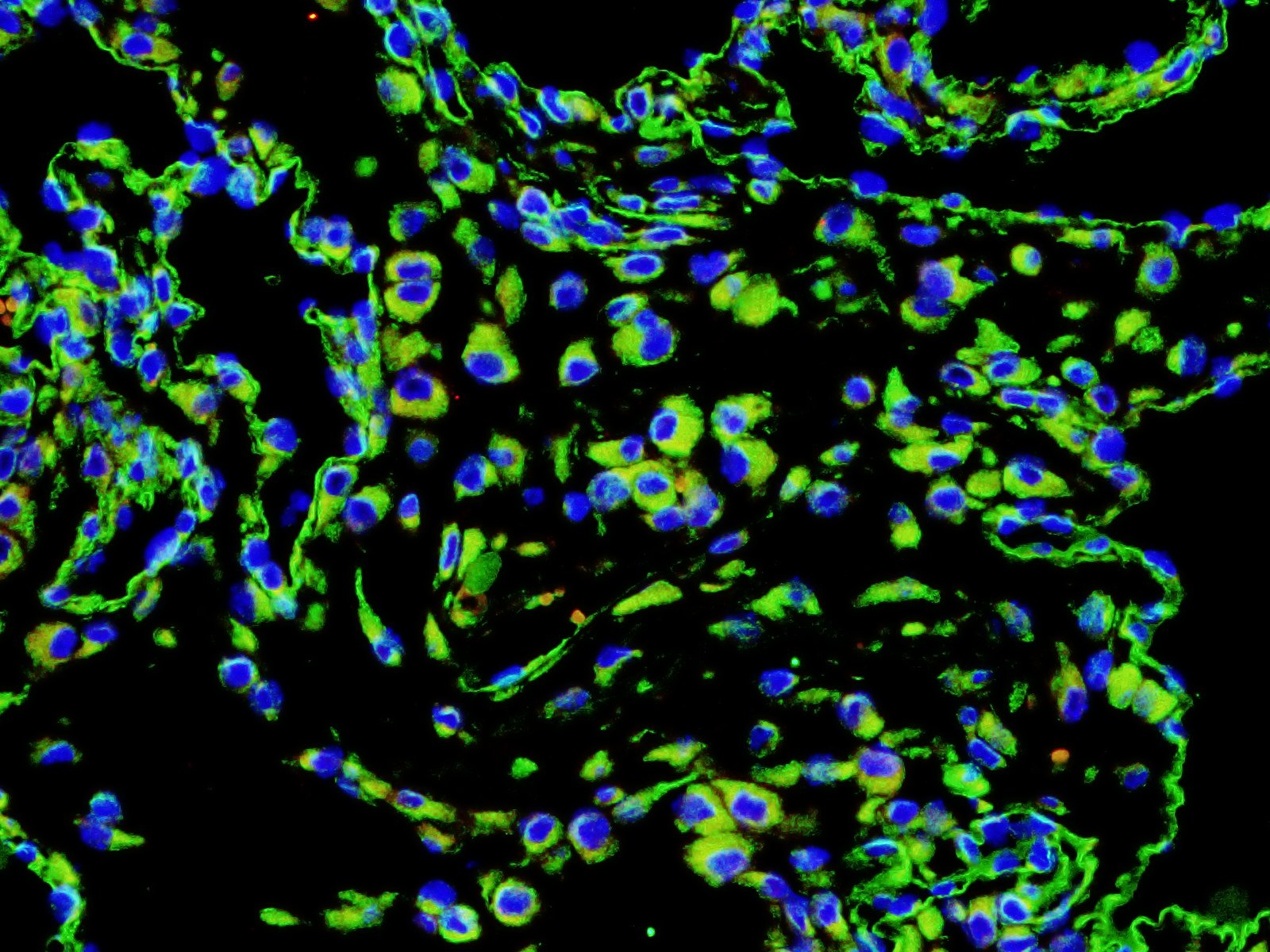

Supplement: Supplementary file 7 — Source data Fig. 5 [file 44318_2024_220_MOESM7_ESM.zip › Figure5/5D/CDKN2A-WT-400 (1).jpg]

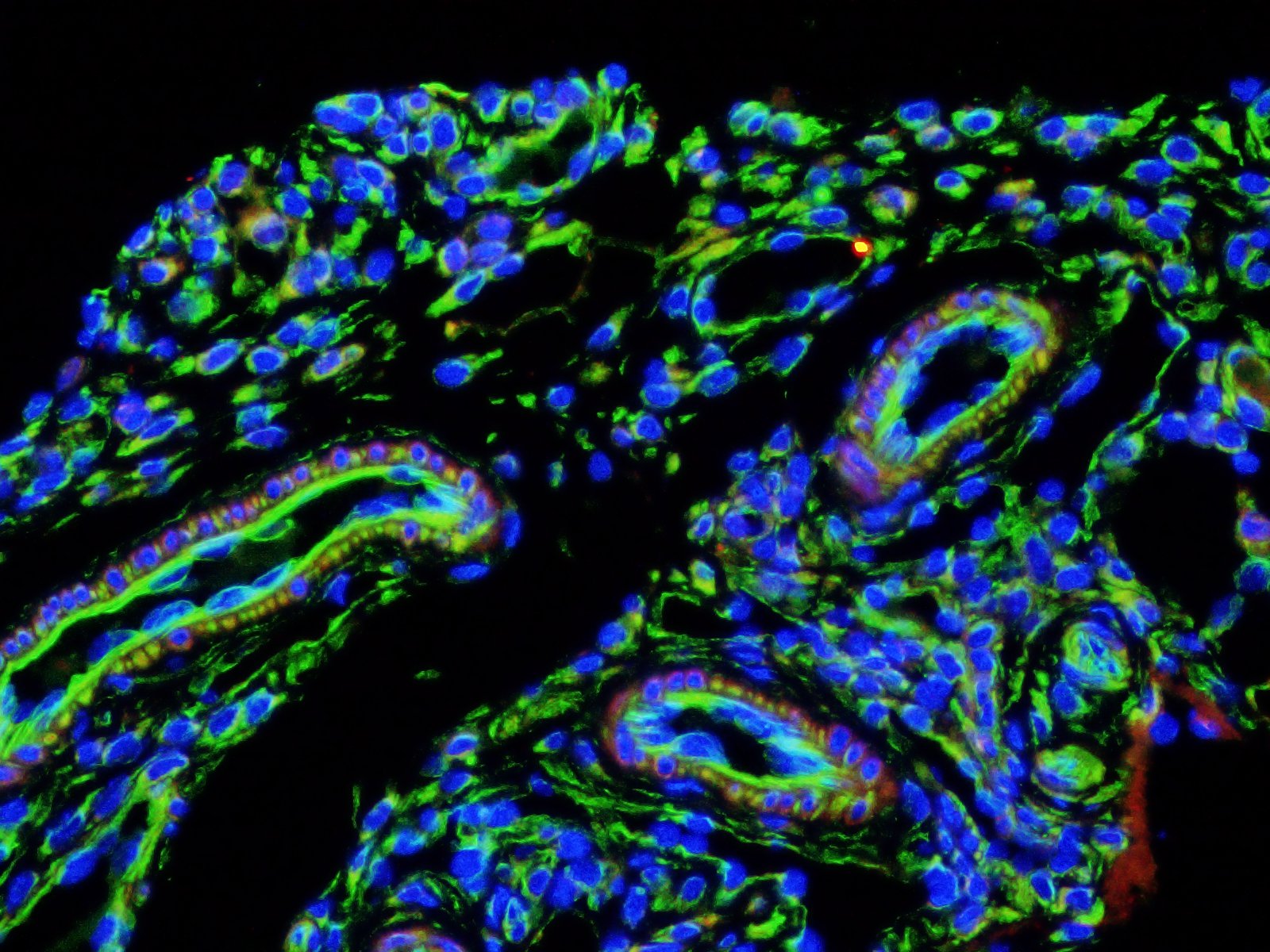

Supplement: Supplementary file 7 — Source data Fig. 5 [file 44318_2024_220_MOESM7_ESM.zip › Figure5/5D/CDKN2A-WT-400 (2).jpg]

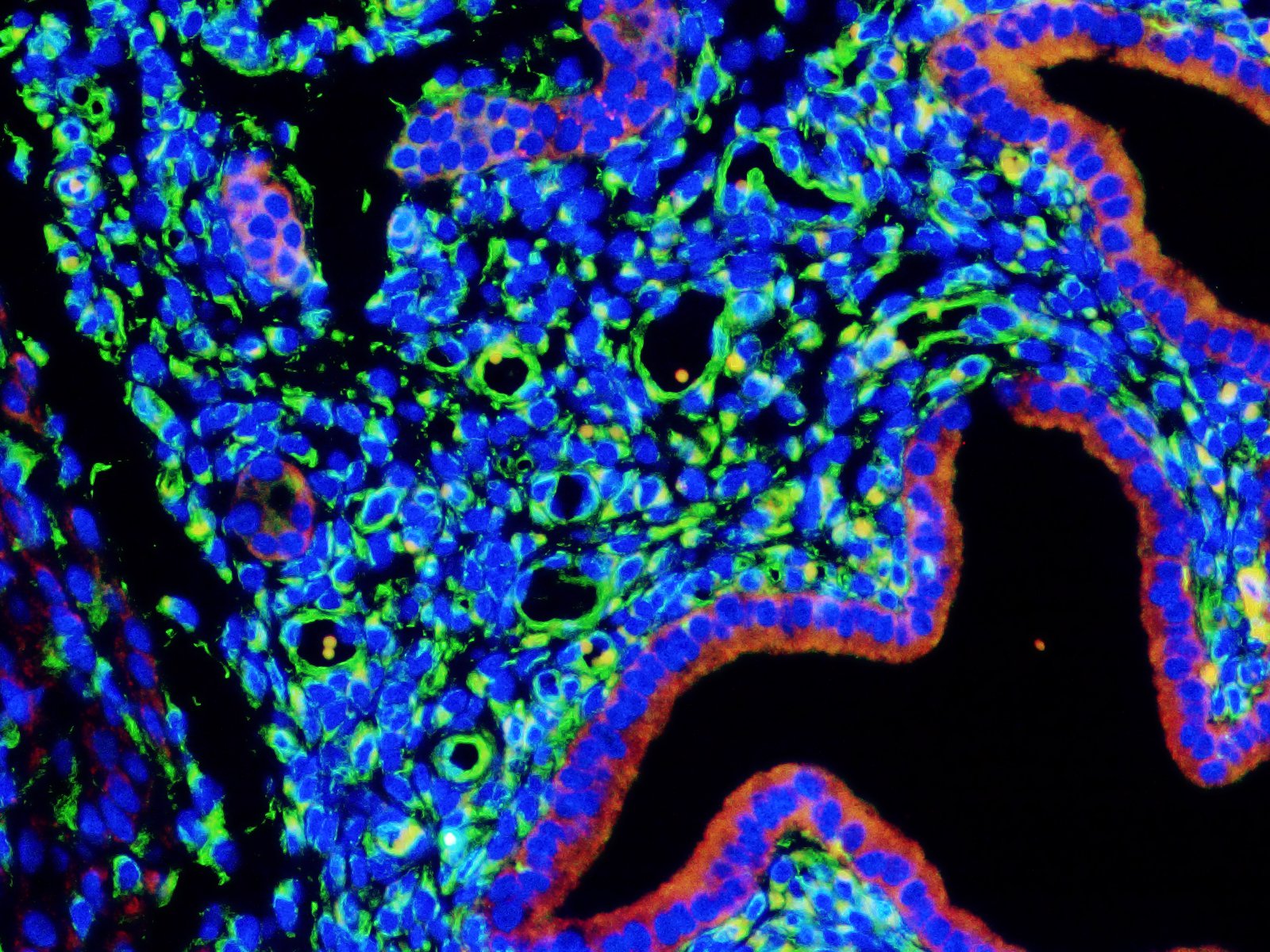

Supplement: Supplementary file 7 — Source data Fig. 5 [file 44318_2024_220_MOESM7_ESM.zip › Figure5/5D/CDKN2A-WT-400 (3).jpg]

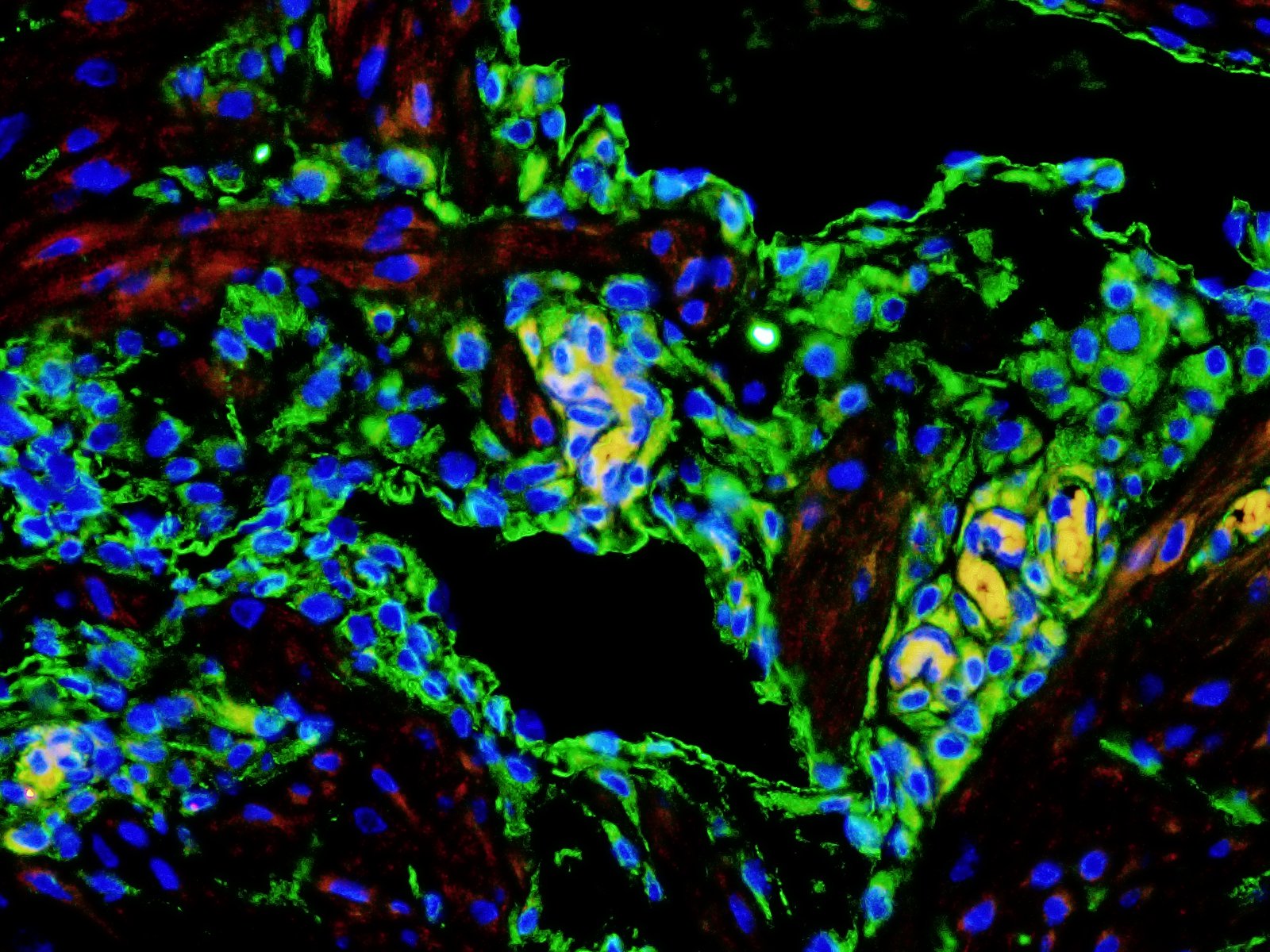

Supplement: Supplementary file 7 — Source data Fig. 5 [file 44318_2024_220_MOESM7_ESM.zip › Figure5/5D/CDKN2A-WT-400 (4).jpg]

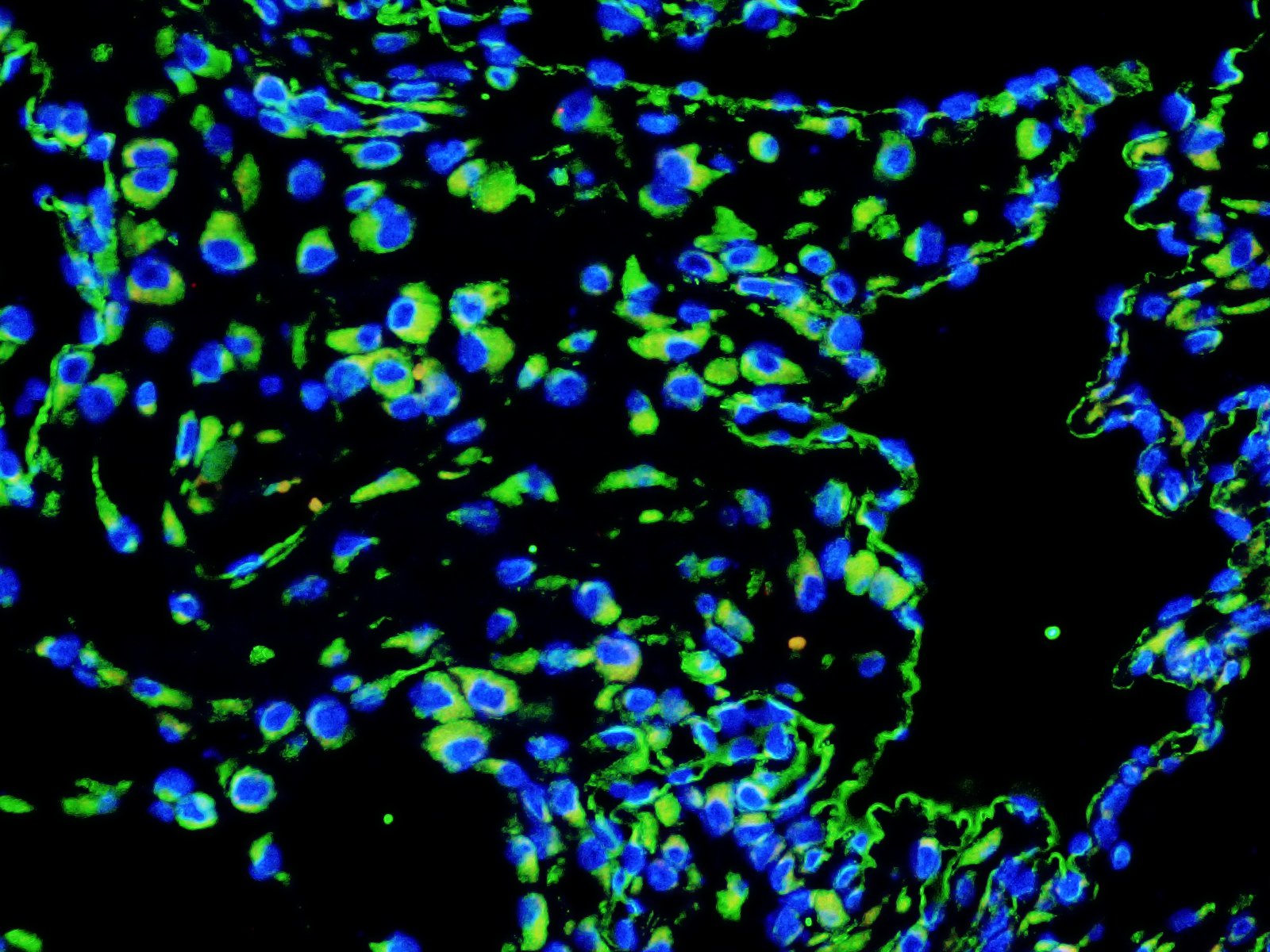

Supplement: Supplementary file 7 — Source data Fig. 5 [file 44318_2024_220_MOESM7_ESM.zip › Figure5/5D/CDKN2A-WT-400 (5).jpg]

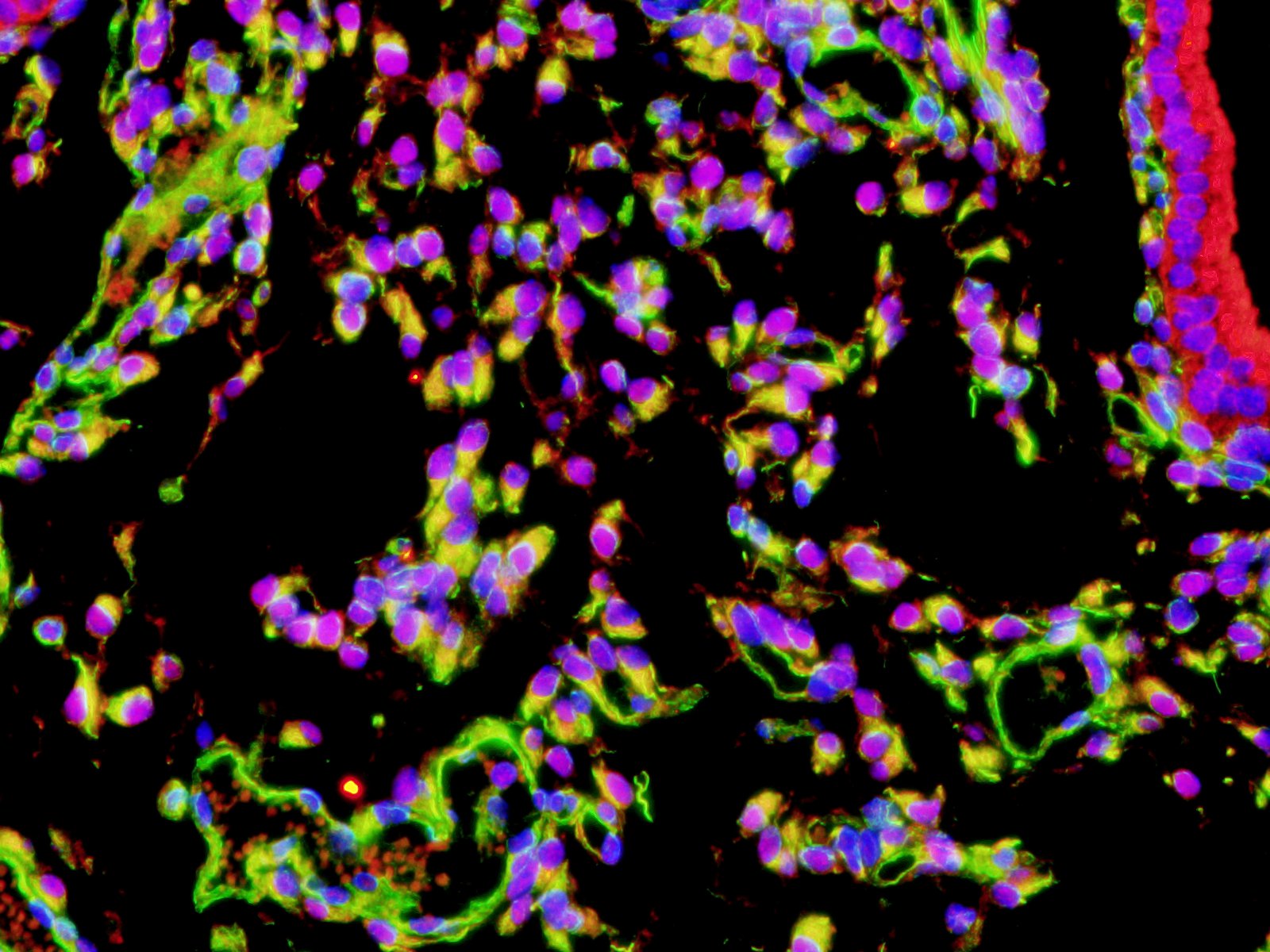

Supplement: Supplementary file 7 — Source data Fig. 5 [file 44318_2024_220_MOESM7_ESM.zip › Figure5/5D/TP53-Hvem-Tnfrsf14 (1).jpg]

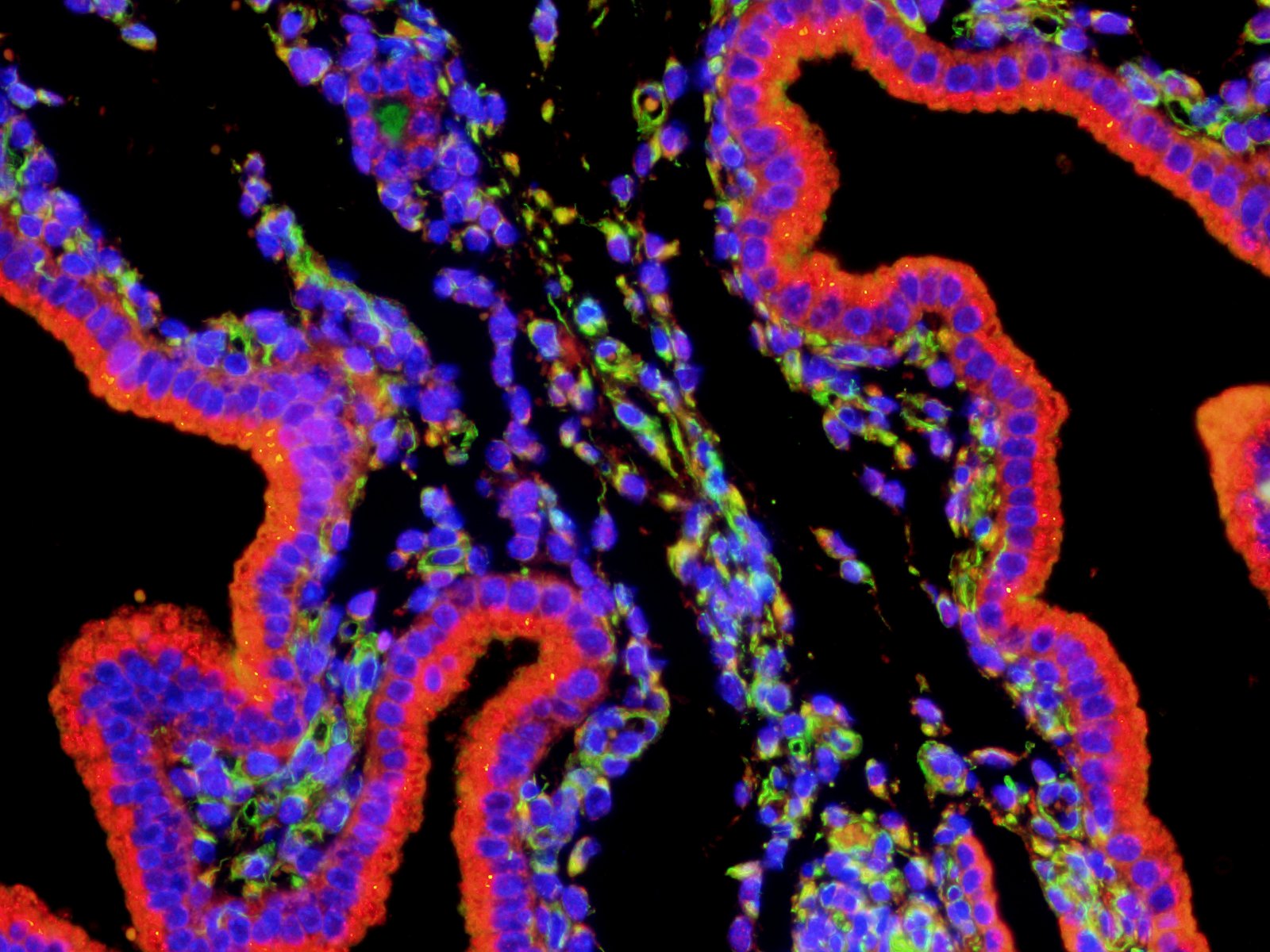

Supplement: Supplementary file 7 — Source data Fig. 5 [file 44318_2024_220_MOESM7_ESM.zip › Figure5/5D/TP53-Hvem-Tnfrsf14 (2).jpg]

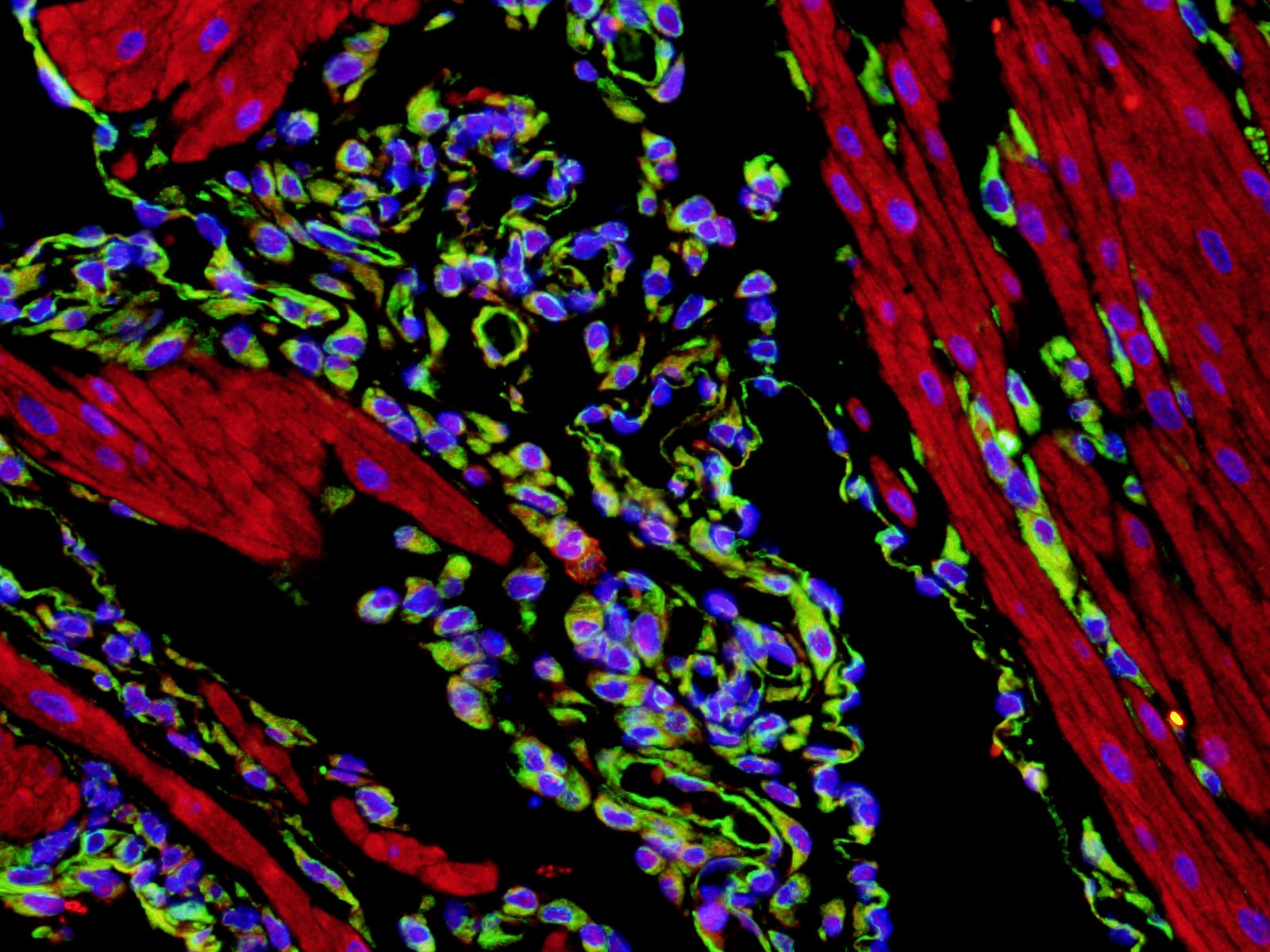

Supplement: Supplementary file 7 — Source data Fig. 5 [file 44318_2024_220_MOESM7_ESM.zip › Figure5/5D/TP53-Hvem-Tnfrsf14 (3).jpg]

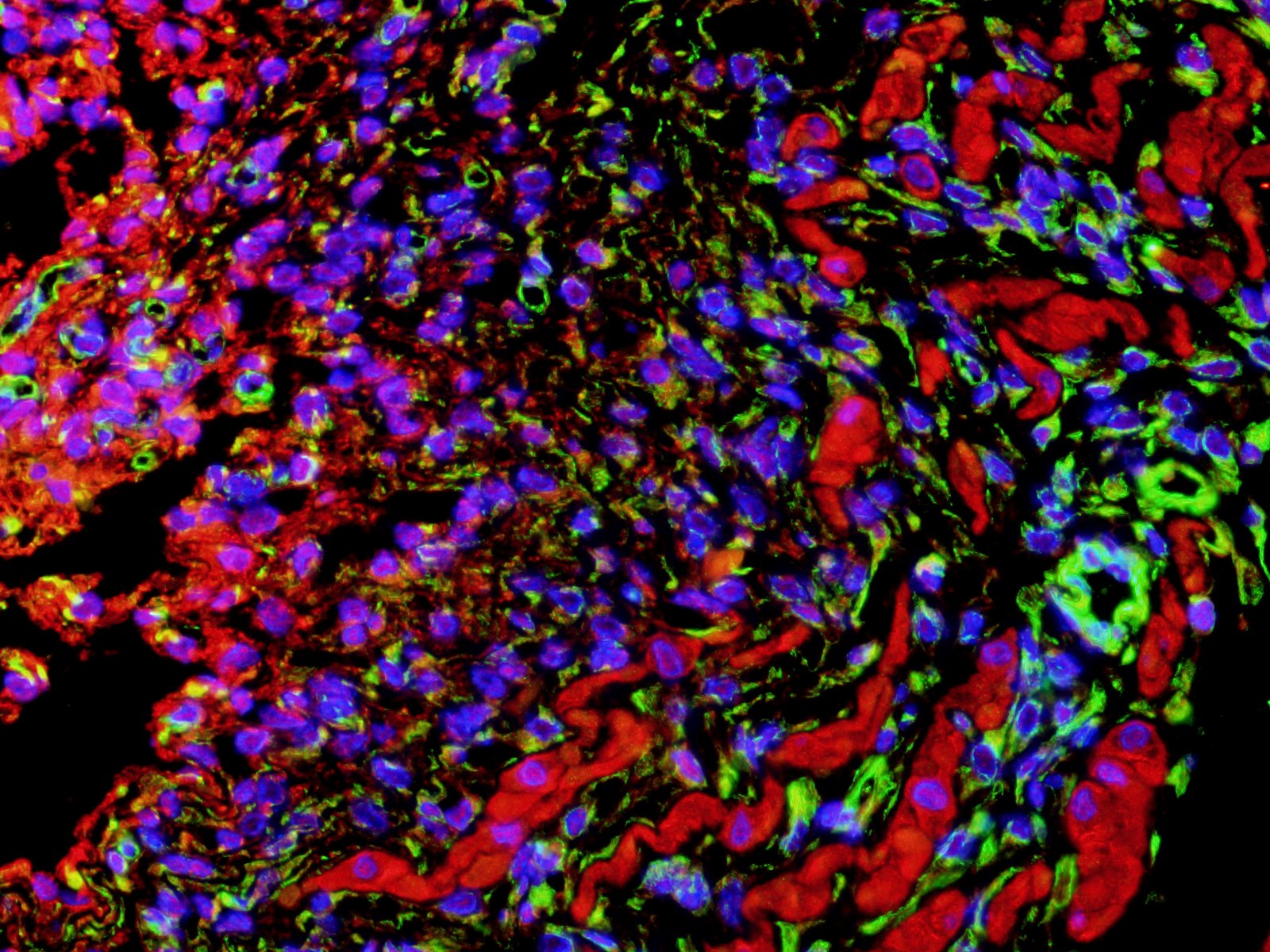

Supplement: Supplementary file 7 — Source data Fig. 5 [file 44318_2024_220_MOESM7_ESM.zip › Figure5/5D/TP53-Hvem-Tnfrsf14 (4).jpg]

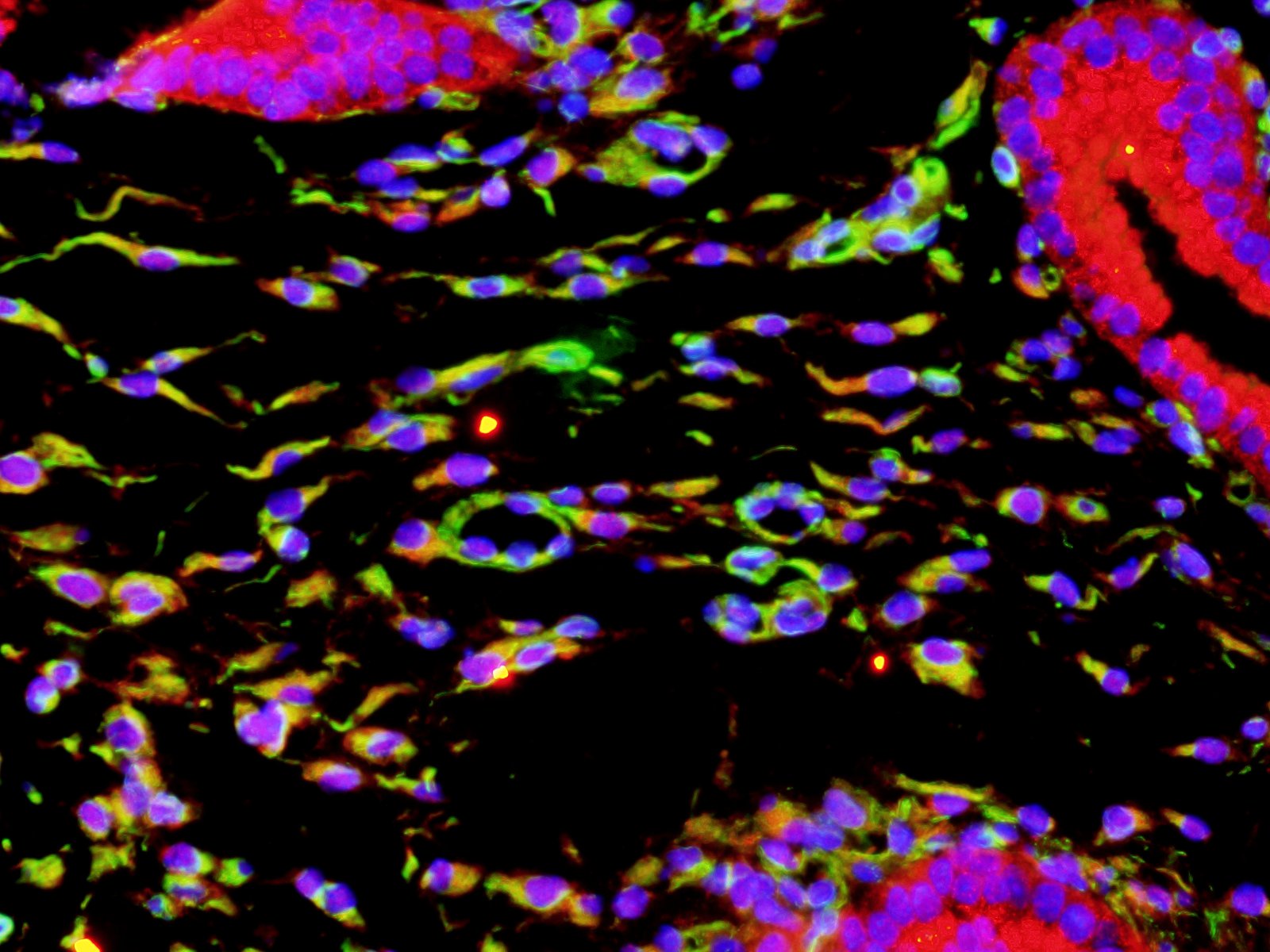

Supplement: Supplementary file 7 — Source data Fig. 5 [file 44318_2024_220_MOESM7_ESM.zip › Figure5/5D/TP53-Hvem-Tnfrsf14 (5).jpg]
