# Supplementary figures and images for: TNFSF14+ natural killer cells prevent spontaneous abortion by restricting leucine-mediated decidual stromal cell senescence (part 4 of 4)
Source: EMBO J. 2024 Sep 11;43(21):5018–36. doi: 10.1038/s44318-024-00220-3 (PMC11535022; doi:10.1038/s44318-024-00220-3)

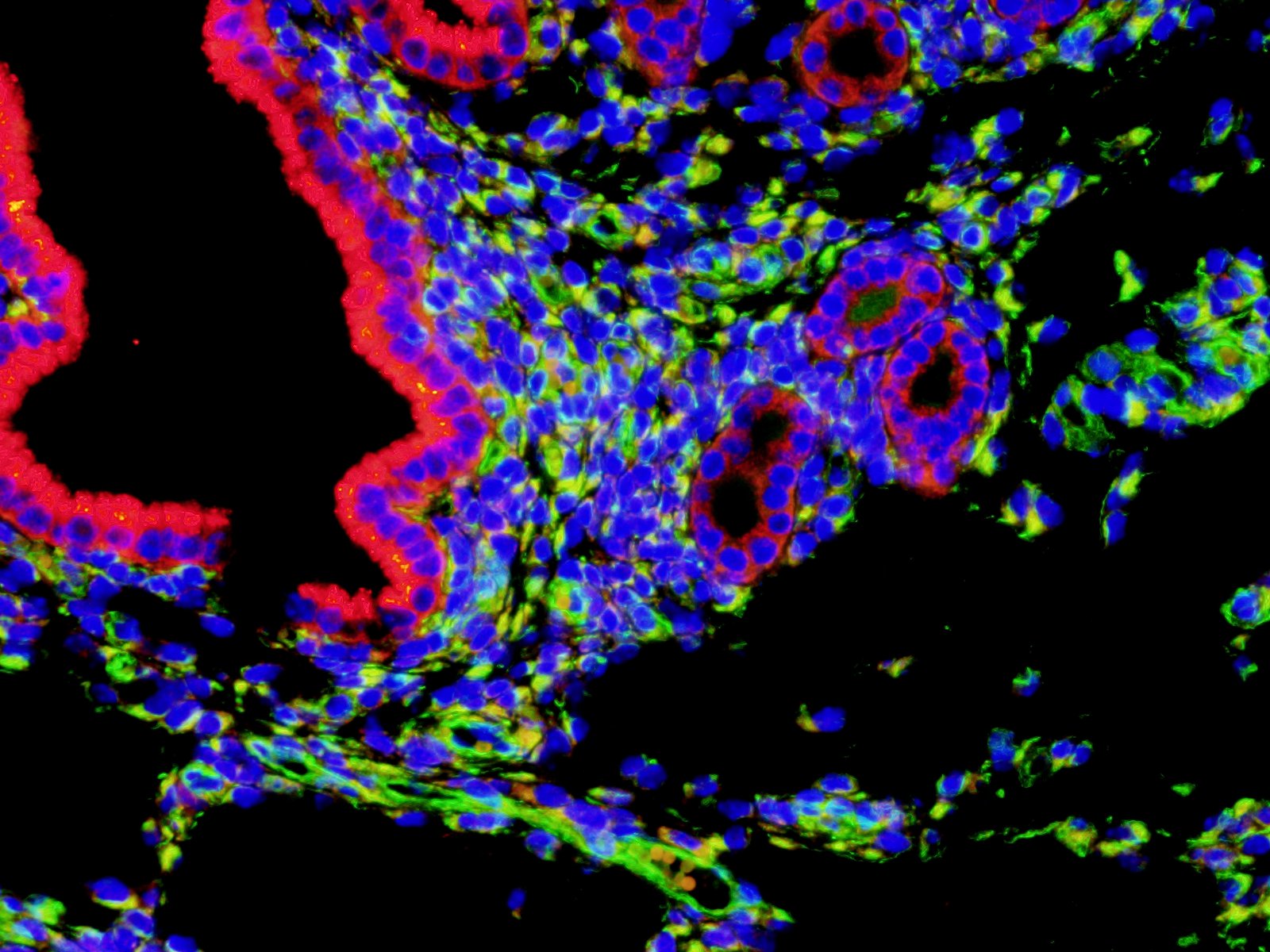

Supplement: Supplementary file 7 — Source data Fig. 5 [file 44318_2024_220_MOESM7_ESM.zip › Figure5/5D/TP53-WT-400 (1).jpg]

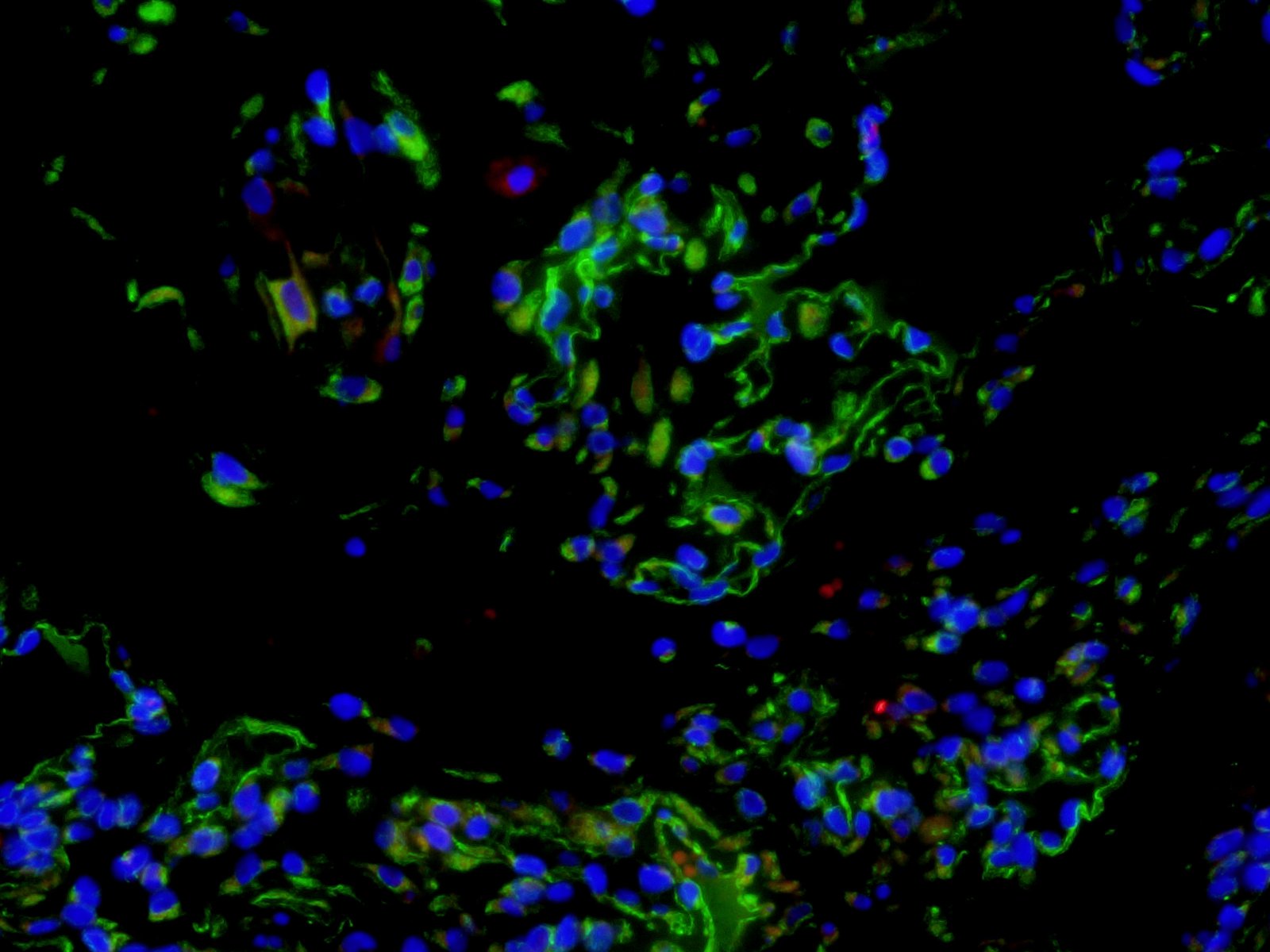

Supplement: Supplementary file 7 — Source data Fig. 5 [file 44318_2024_220_MOESM7_ESM.zip › Figure5/5D/TP53-WT-400 (2).jpg]

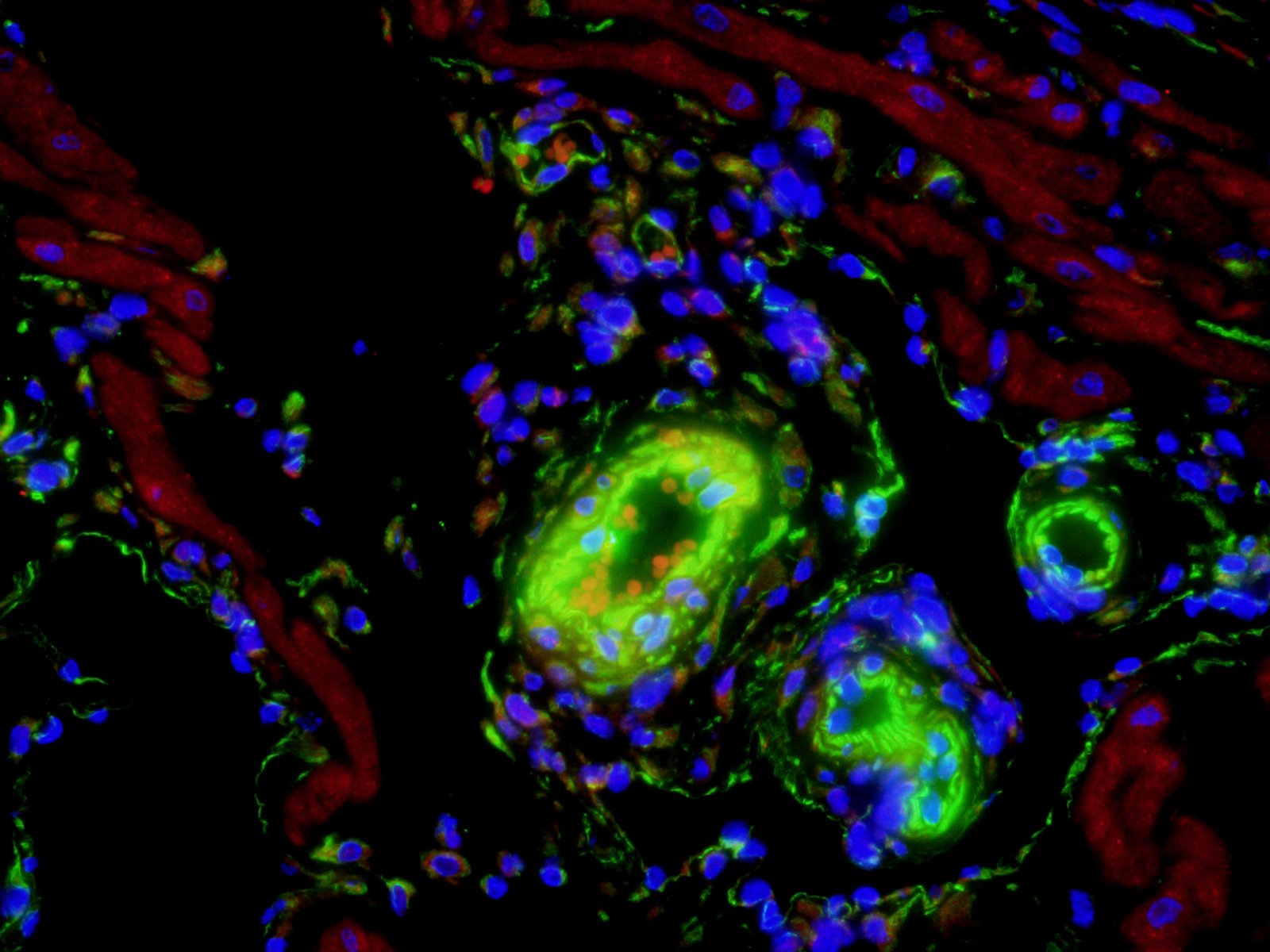

Supplement: Supplementary file 7 — Source data Fig. 5 [file 44318_2024_220_MOESM7_ESM.zip › Figure5/5D/TP53-WT-400 (3).jpg]

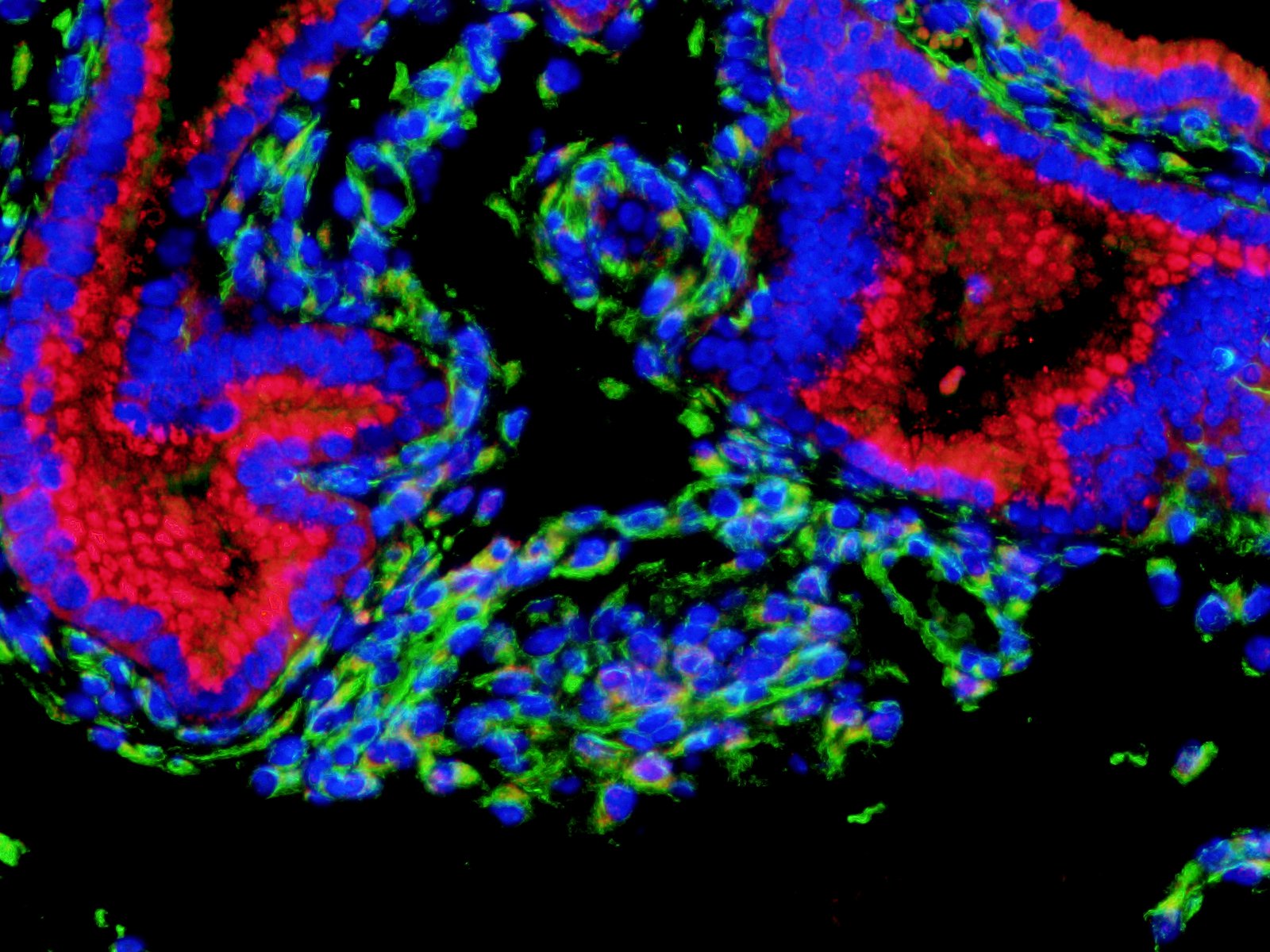

Supplement: Supplementary file 7 — Source data Fig. 5 [file 44318_2024_220_MOESM7_ESM.zip › Figure5/5D/TP53-WT-400 (4).jpg]

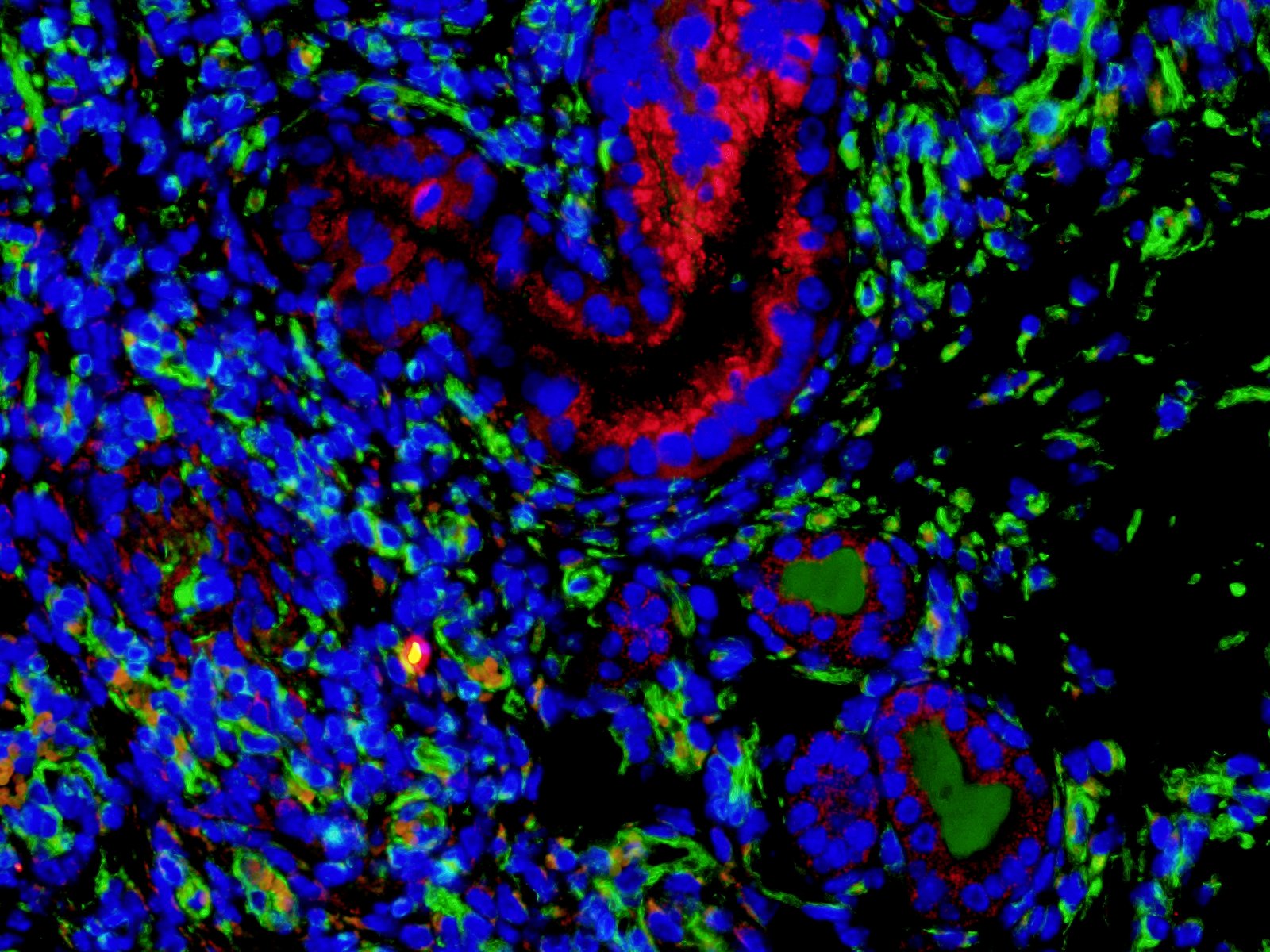

Supplement: Supplementary file 7 — Source data Fig. 5 [file 44318_2024_220_MOESM7_ESM.zip › Figure5/5D/TP53-WT-400 (5).jpg]

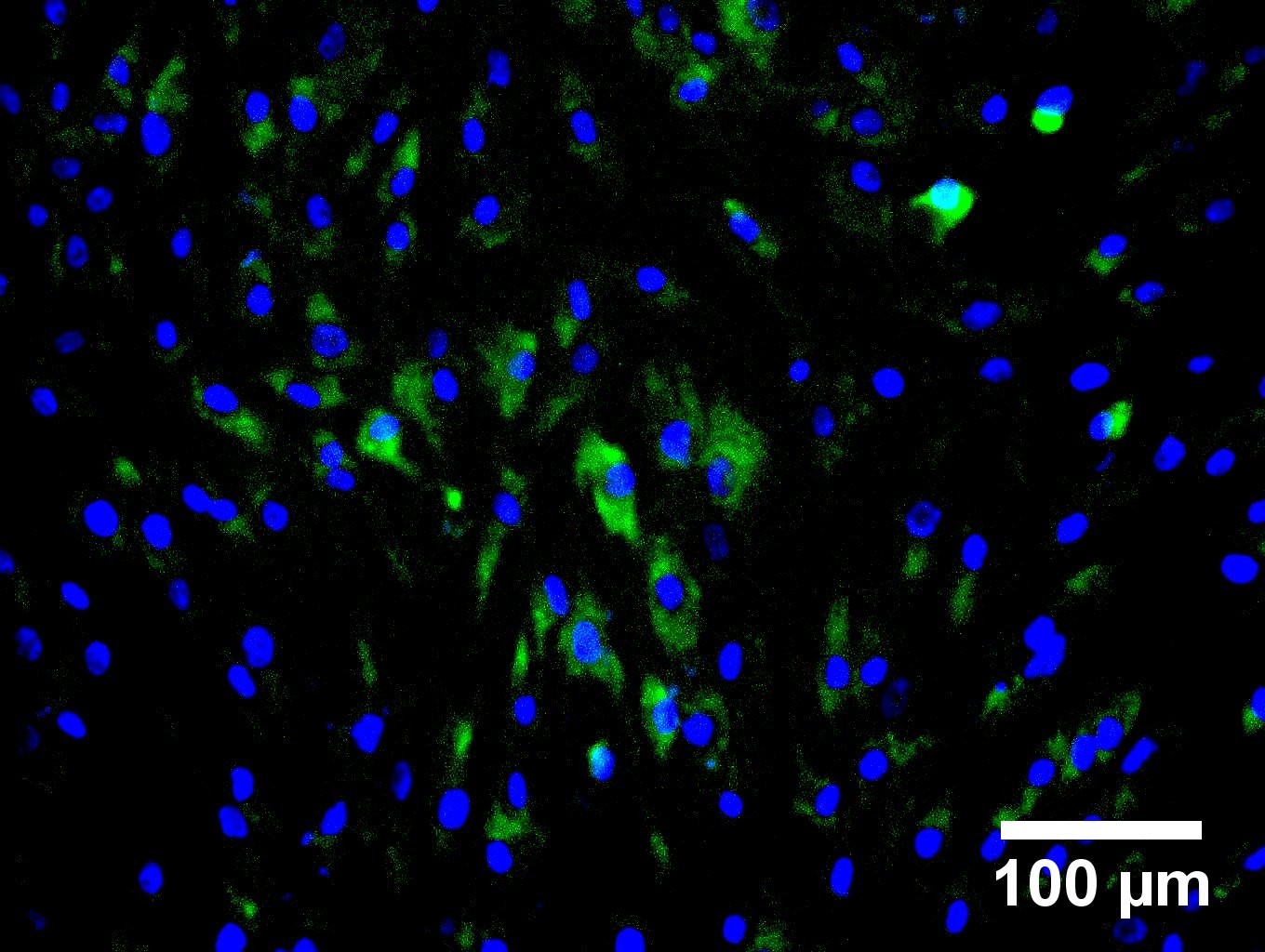

Supplement: Supplementary file 7 — Source data Fig. 5 [file 44318_2024_220_MOESM7_ESM.zip › Figure5/5F/Ctrl2 (3).jpg]

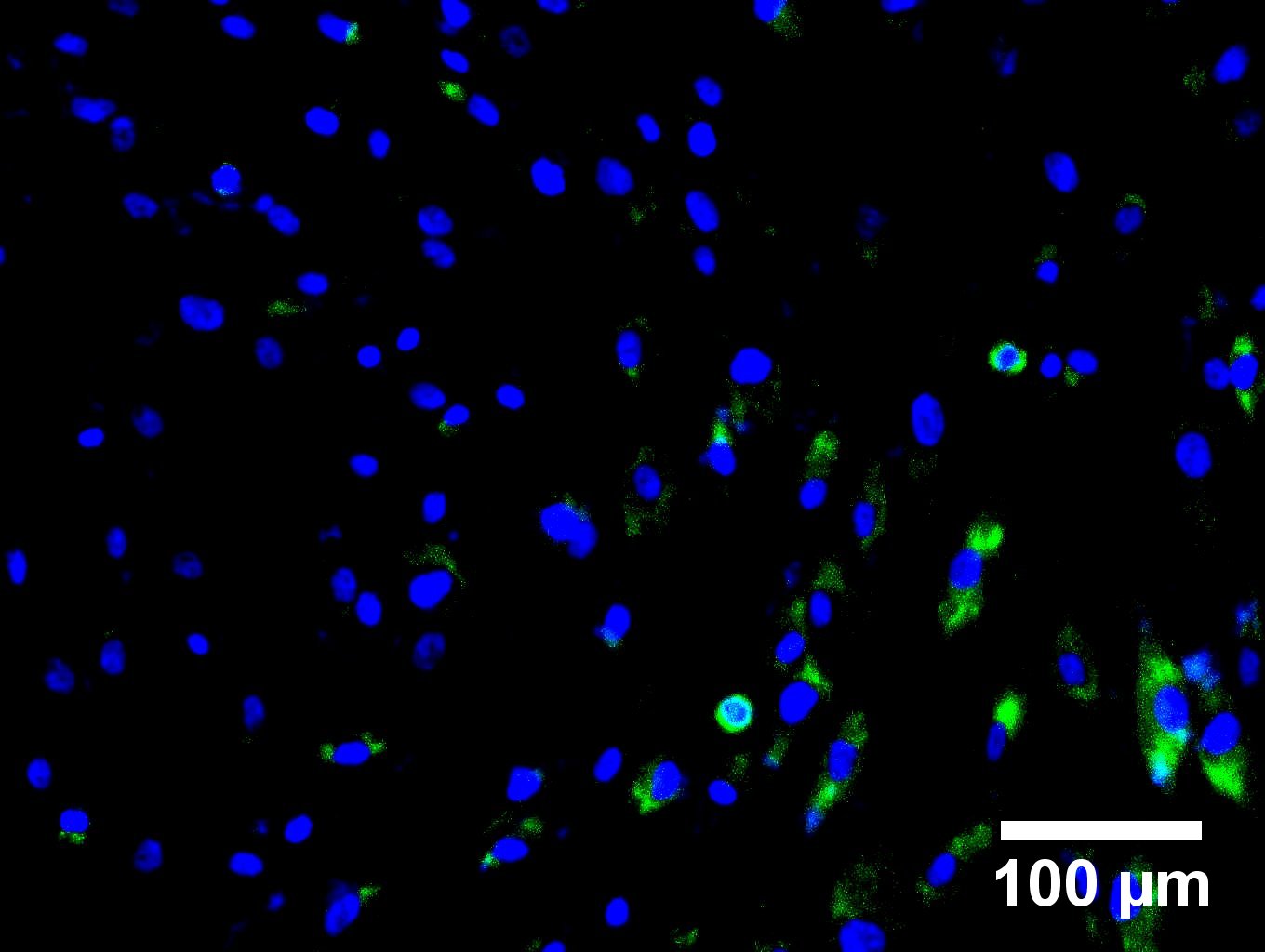

Supplement: Supplementary file 7 — Source data Fig. 5 [file 44318_2024_220_MOESM7_ESM.zip › Figure5/5F/TNFSF14-1 (1).jpg]

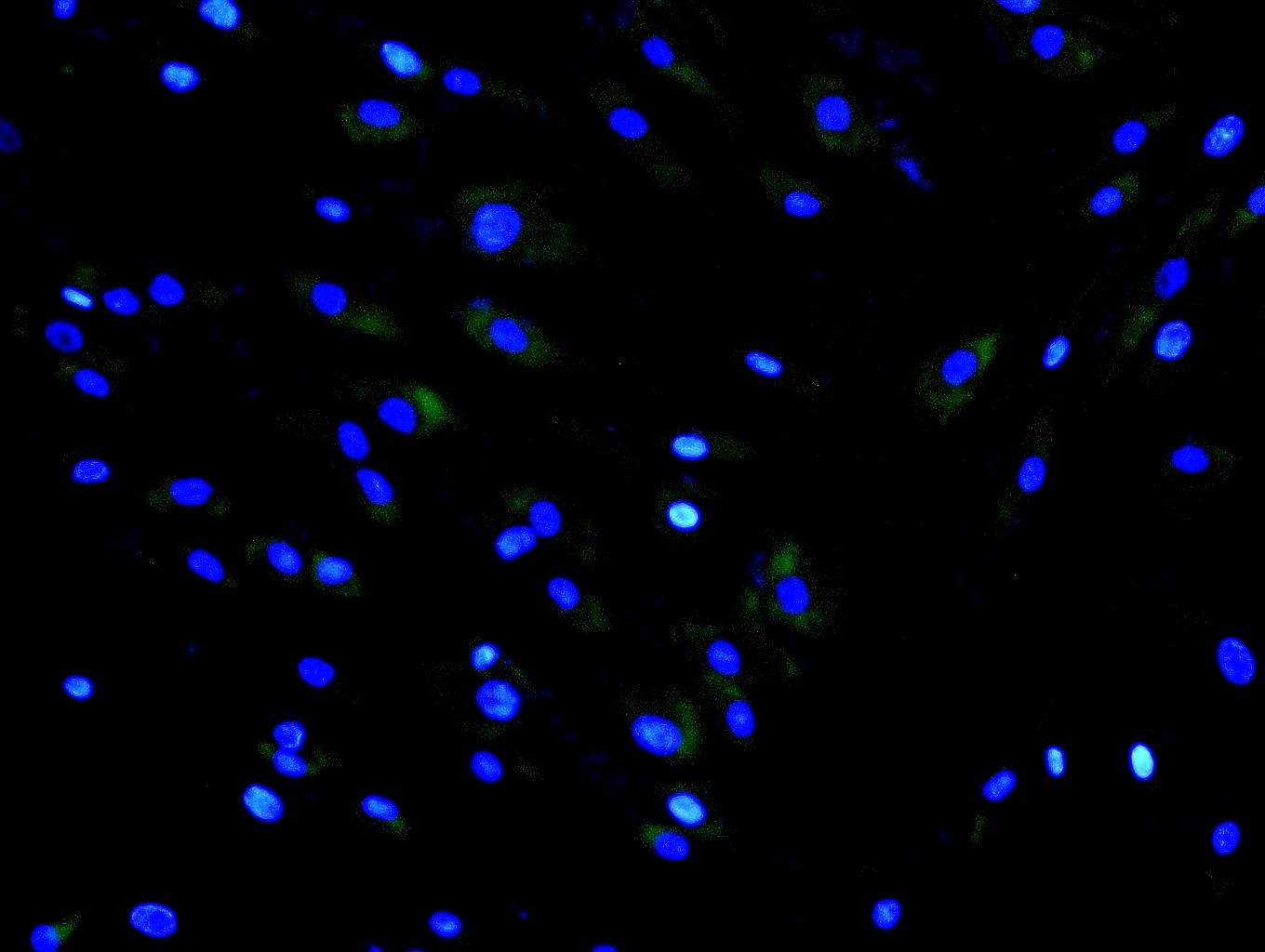

Supplement: Supplementary file 7 — Source data Fig. 5 [file 44318_2024_220_MOESM7_ESM.zip › Figure5/5F/TNFSF14-1 (2).jpg]

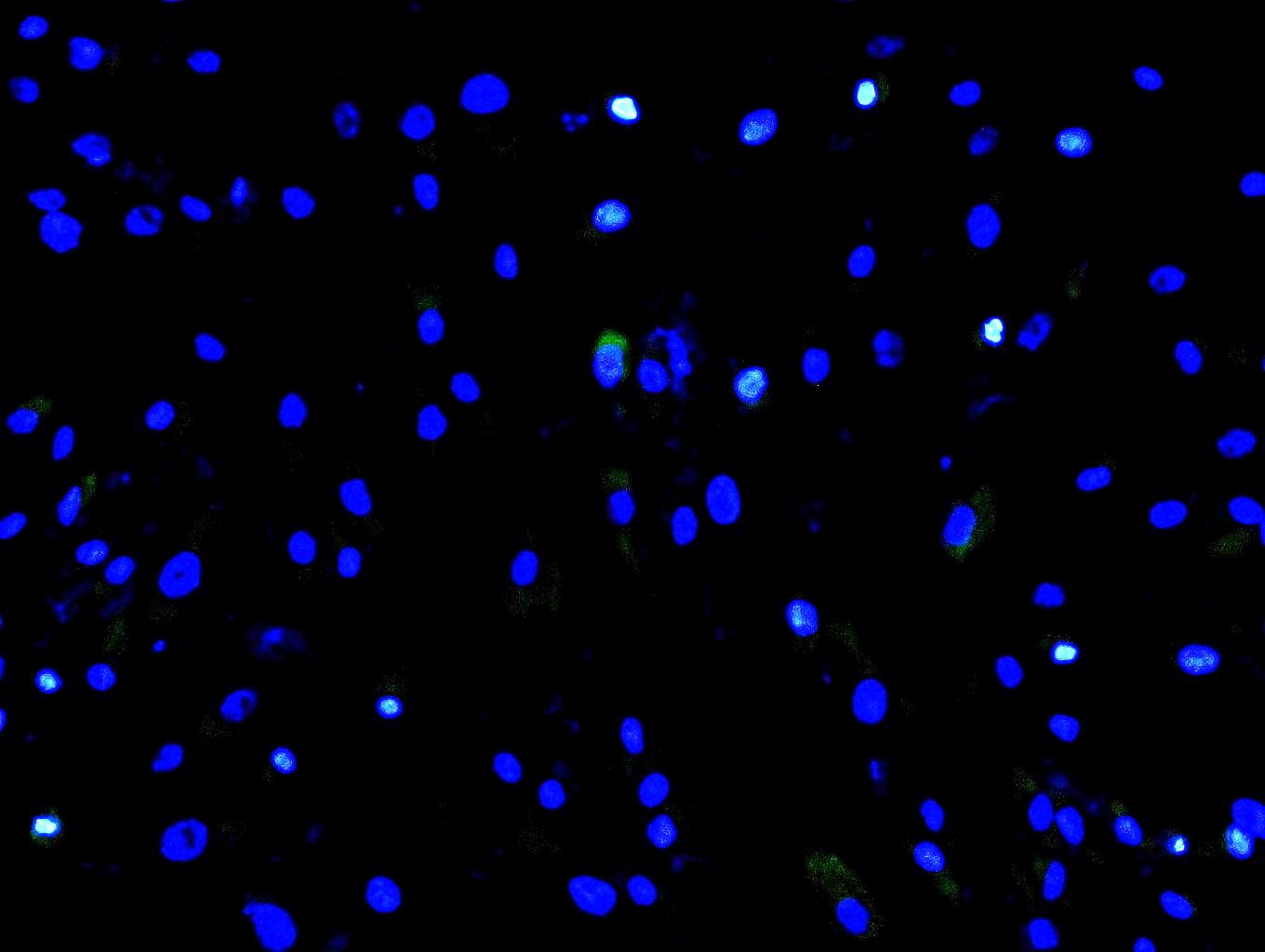

Supplement: Supplementary file 7 — Source data Fig. 5 [file 44318_2024_220_MOESM7_ESM.zip › Figure5/5F/TNFSF14-1 (3).jpg]

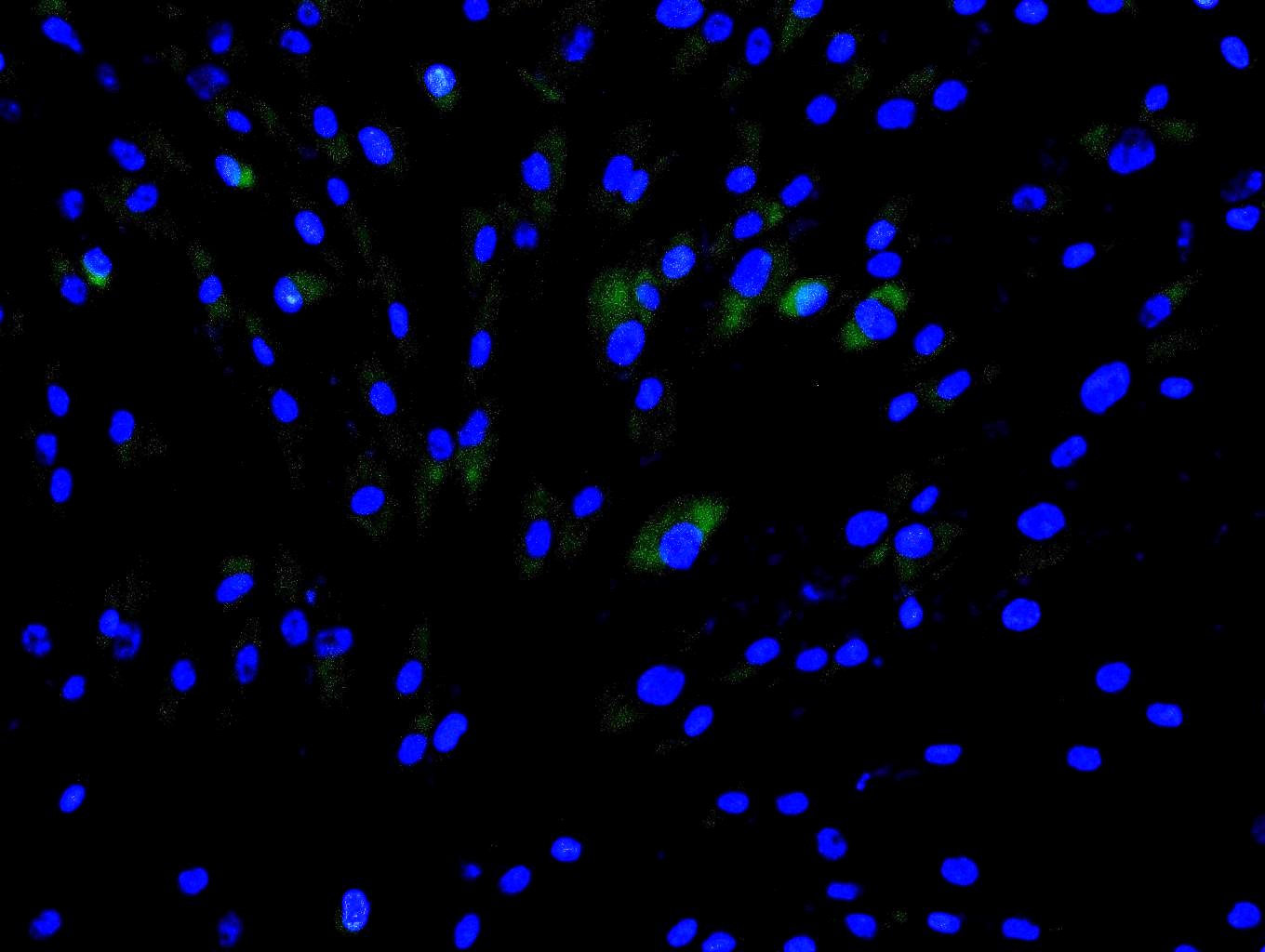

Supplement: Supplementary file 7 — Source data Fig. 5 [file 44318_2024_220_MOESM7_ESM.zip › Figure5/5F/TNFSF14-2 (1).jpg]

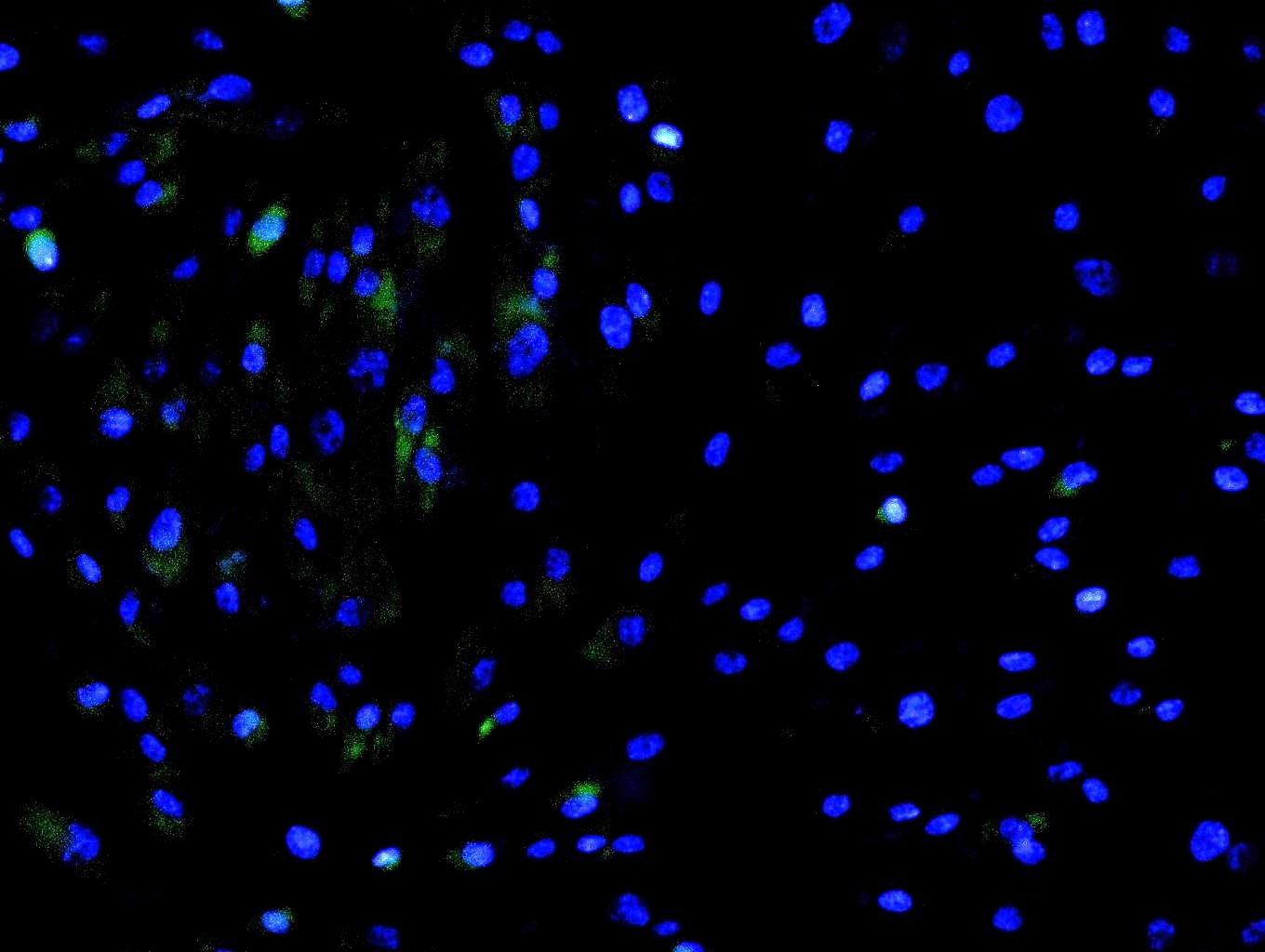

Supplement: Supplementary file 7 — Source data Fig. 5 [file 44318_2024_220_MOESM7_ESM.zip › Figure5/5F/TNFSF14-2 (2).jpg]

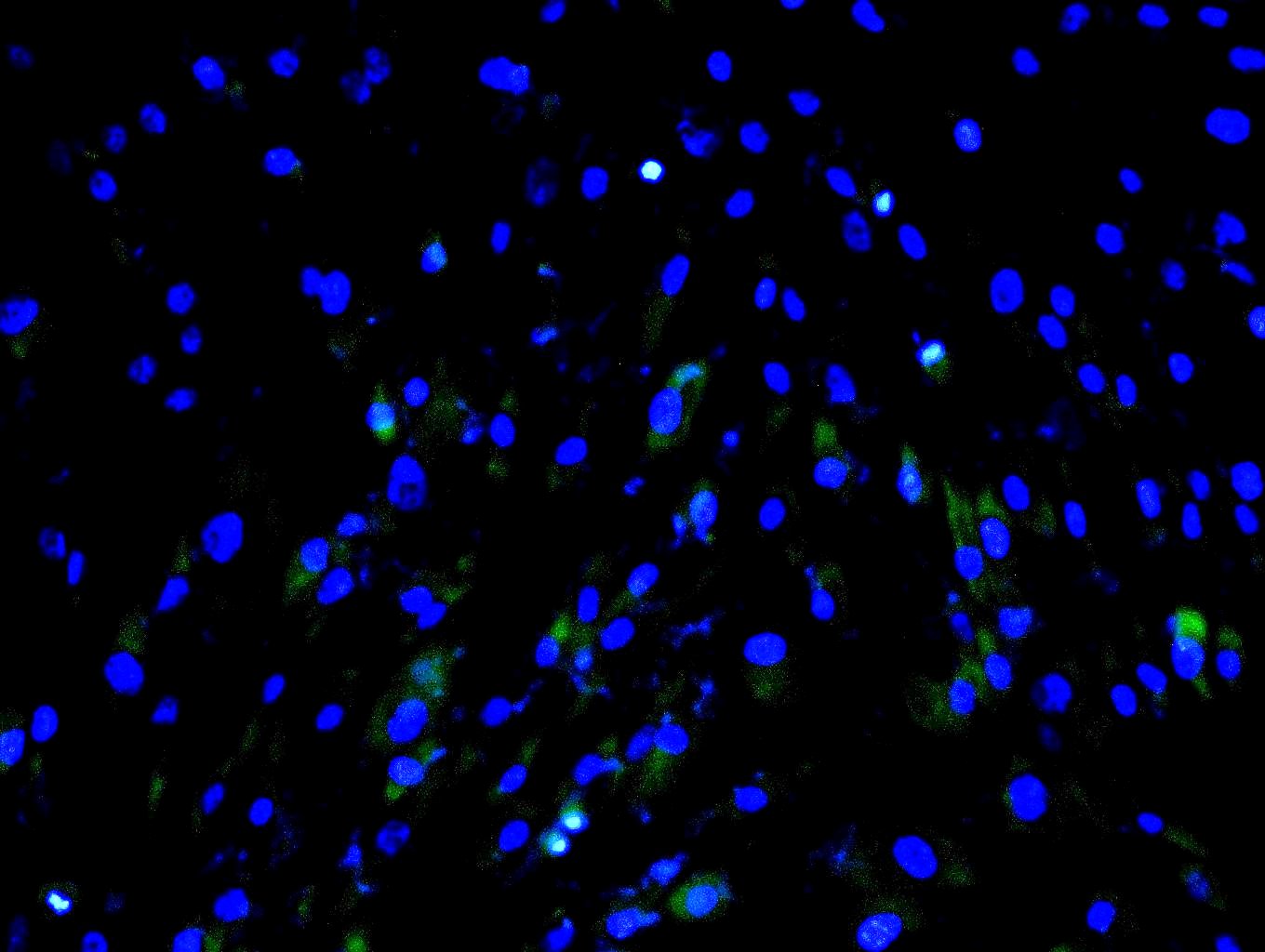

Supplement: Supplementary file 7 — Source data Fig. 5 [file 44318_2024_220_MOESM7_ESM.zip › Figure5/5F/TNFSF14-2 (3).jpg]

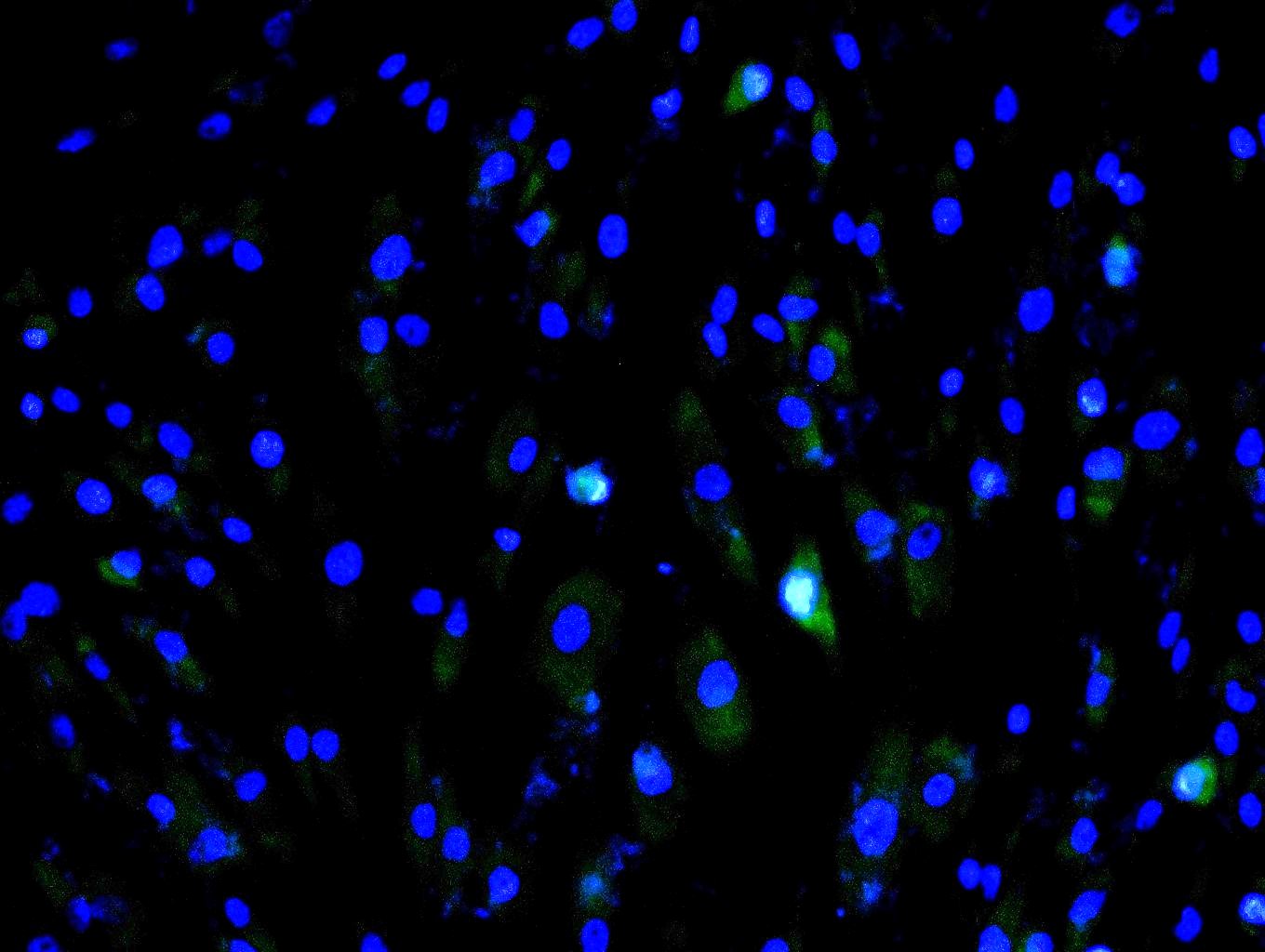

Supplement: Supplementary file 7 — Source data Fig. 5 [file 44318_2024_220_MOESM7_ESM.zip › Figure5/5F/TNFSF14-3 (1).jpg]

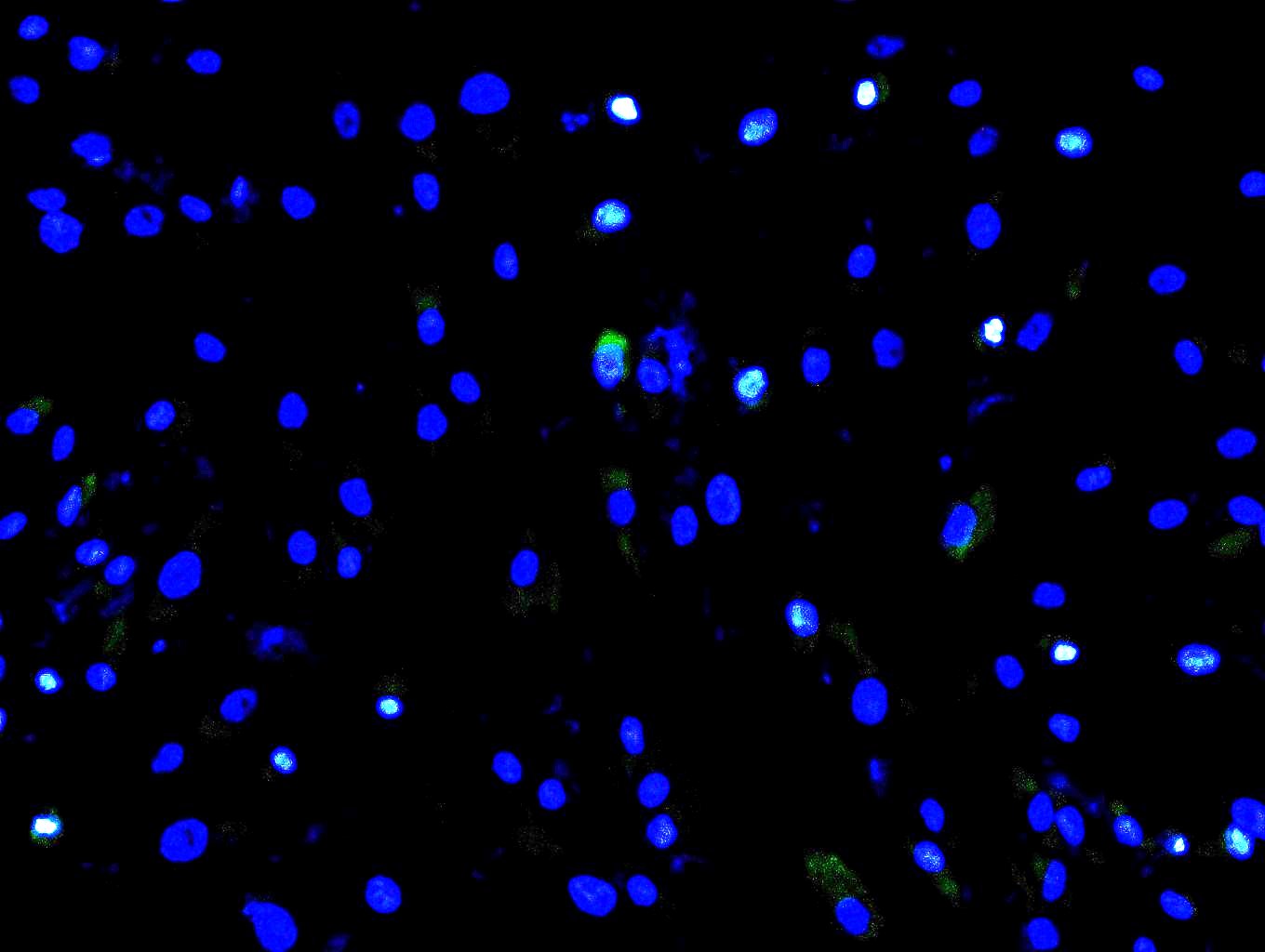

Supplement: Supplementary file 7 — Source data Fig. 5 [file 44318_2024_220_MOESM7_ESM.zip › Figure5/5F/TNFSF14-3 (2).jpg]

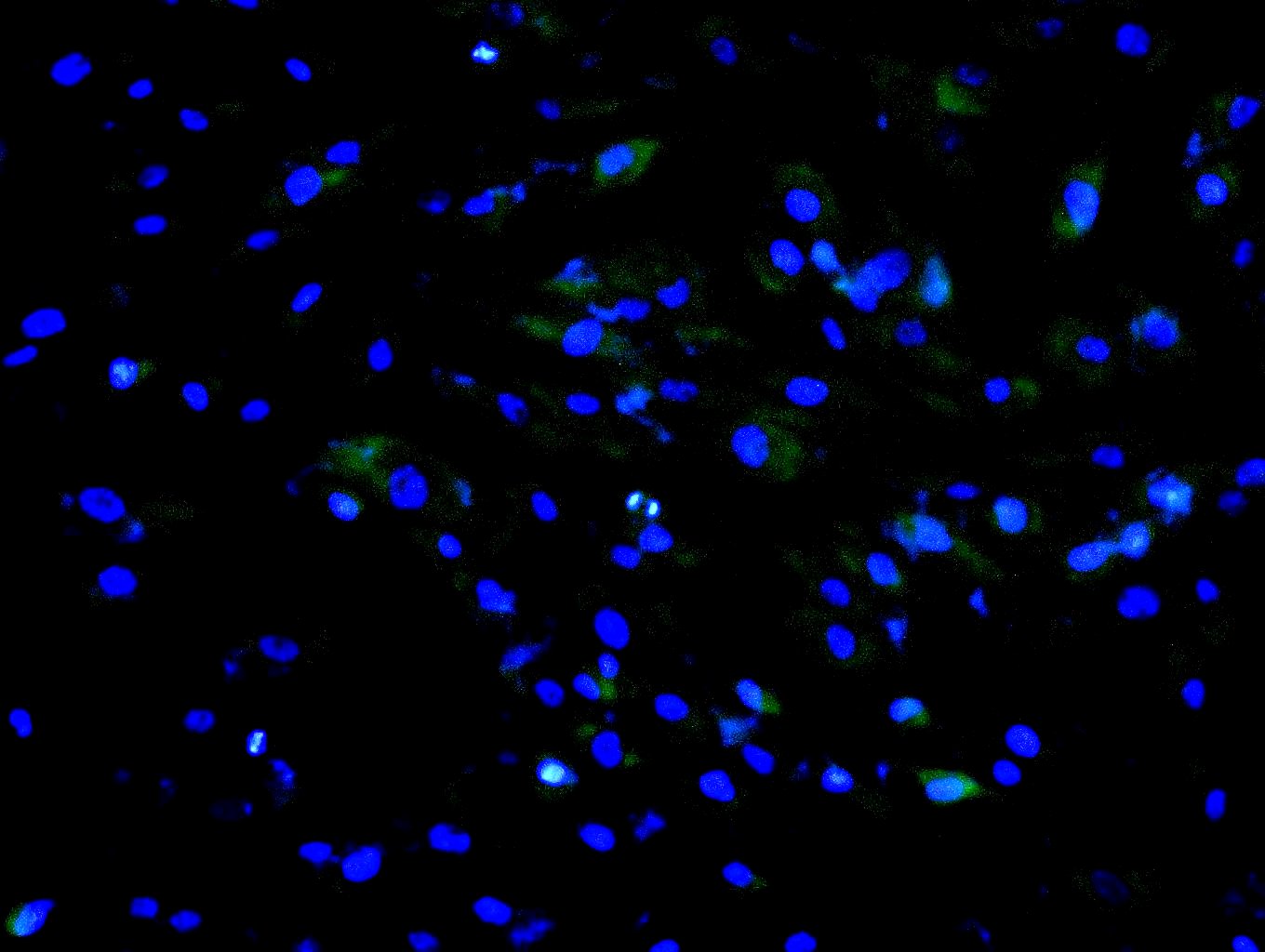

Supplement: Supplementary file 7 — Source data Fig. 5 [file 44318_2024_220_MOESM7_ESM.zip › Figure5/5F/TNFSF14-3 (3).jpg]

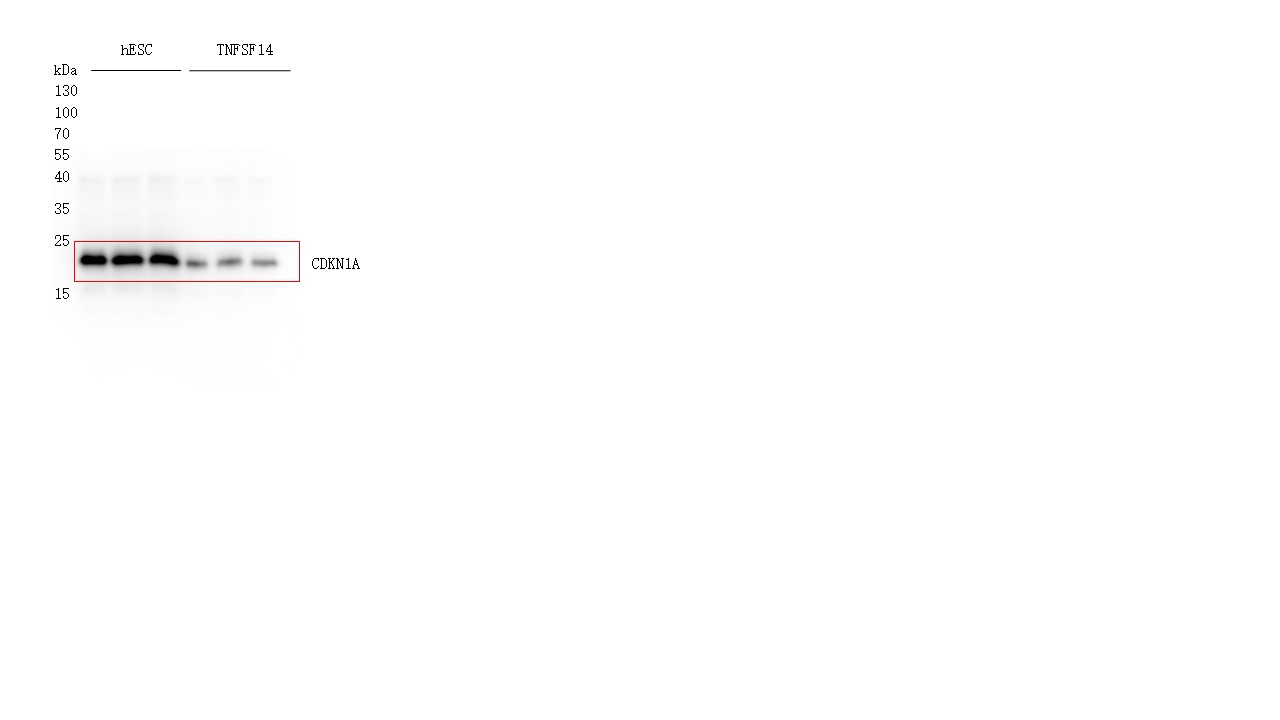

Supplement: Supplementary file 7 — Source data Fig. 5 [file 44318_2024_220_MOESM7_ESM.zip › Figure5/5G/western CDKN1A.jpg]

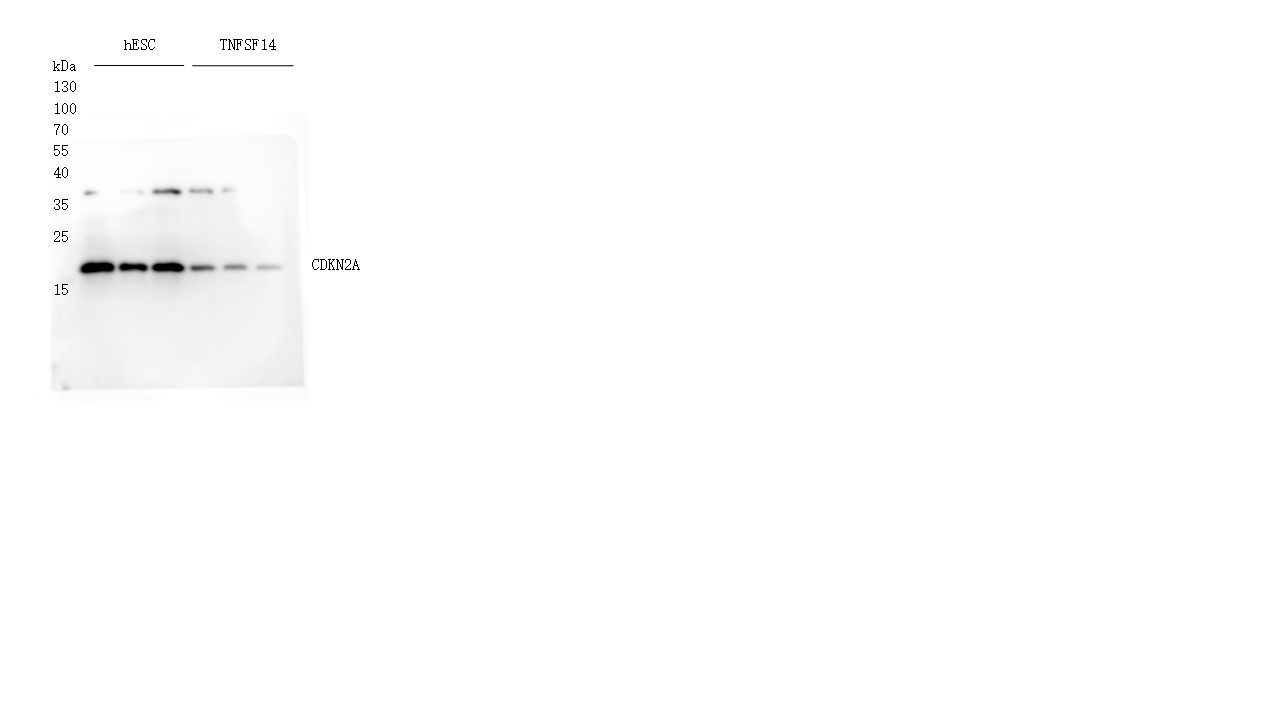

Supplement: Supplementary file 7 — Source data Fig. 5 [file 44318_2024_220_MOESM7_ESM.zip › Figure5/5G/western CDKN2A.jpg]

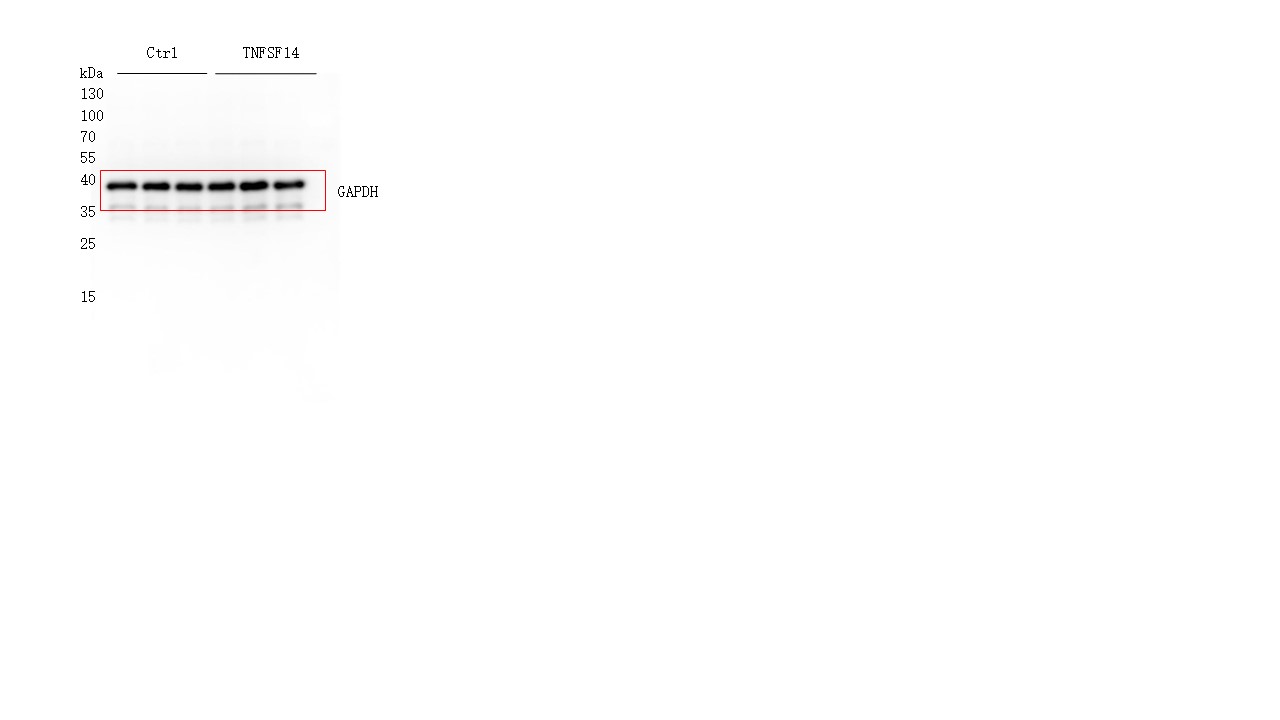

Supplement: Supplementary file 7 — Source data Fig. 5 [file 44318_2024_220_MOESM7_ESM.zip › Figure5/5G/western GAPDH.jpg]

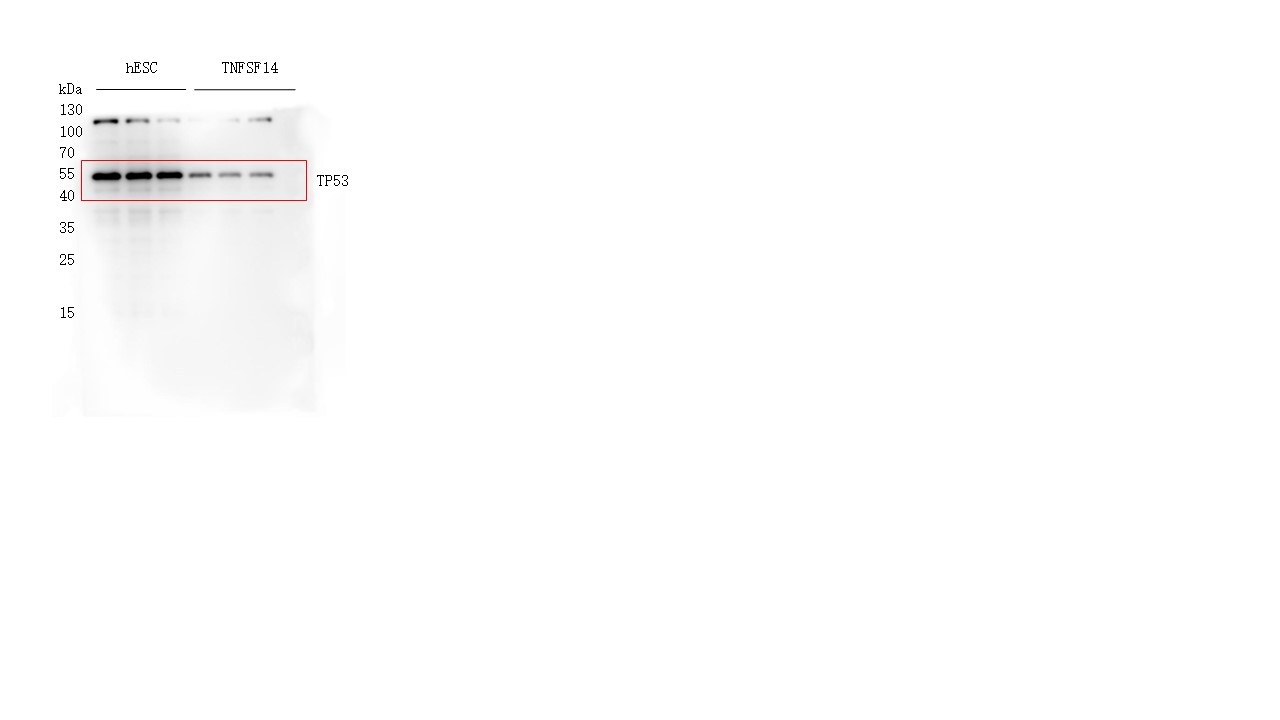

Supplement: Supplementary file 7 — Source data Fig. 5 [file 44318_2024_220_MOESM7_ESM.zip › Figure5/5G/western TP53.jpg]

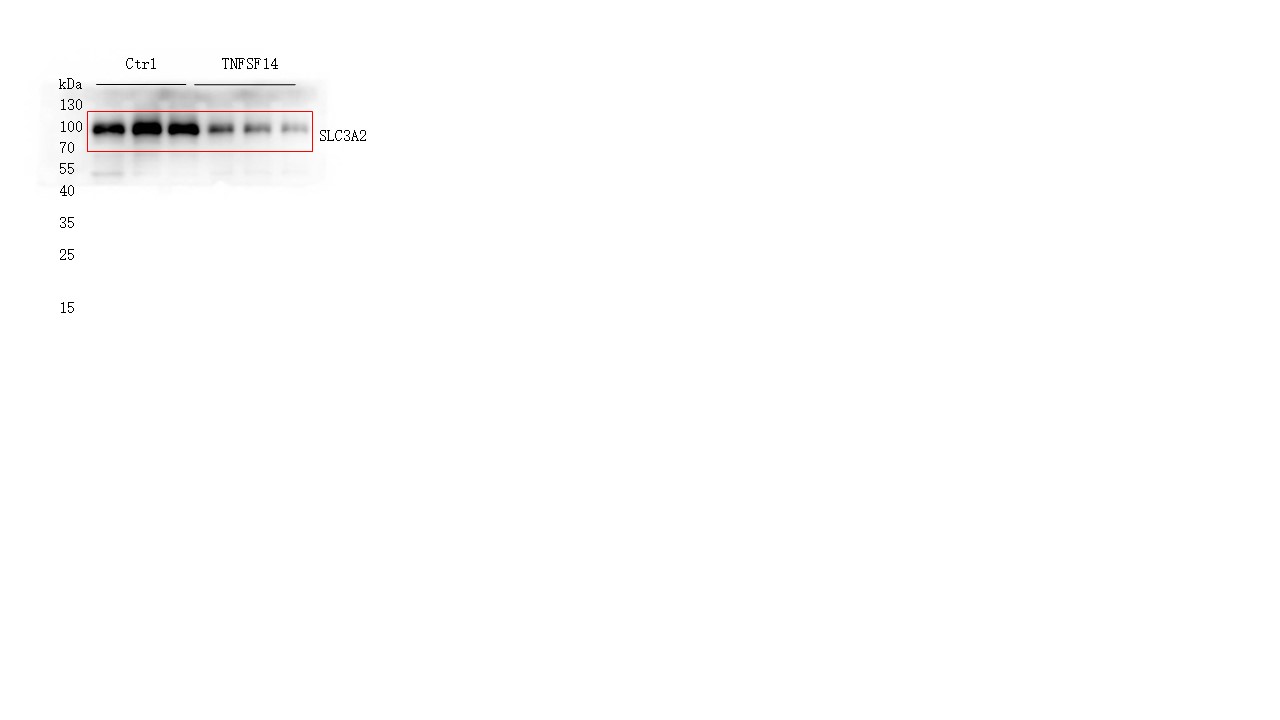

Supplement: Supplementary file 8 — Source data Fig. 6 [file 44318_2024_220_MOESM8_ESM.zip › Figure6/6C/western SLC3A2.jpg]

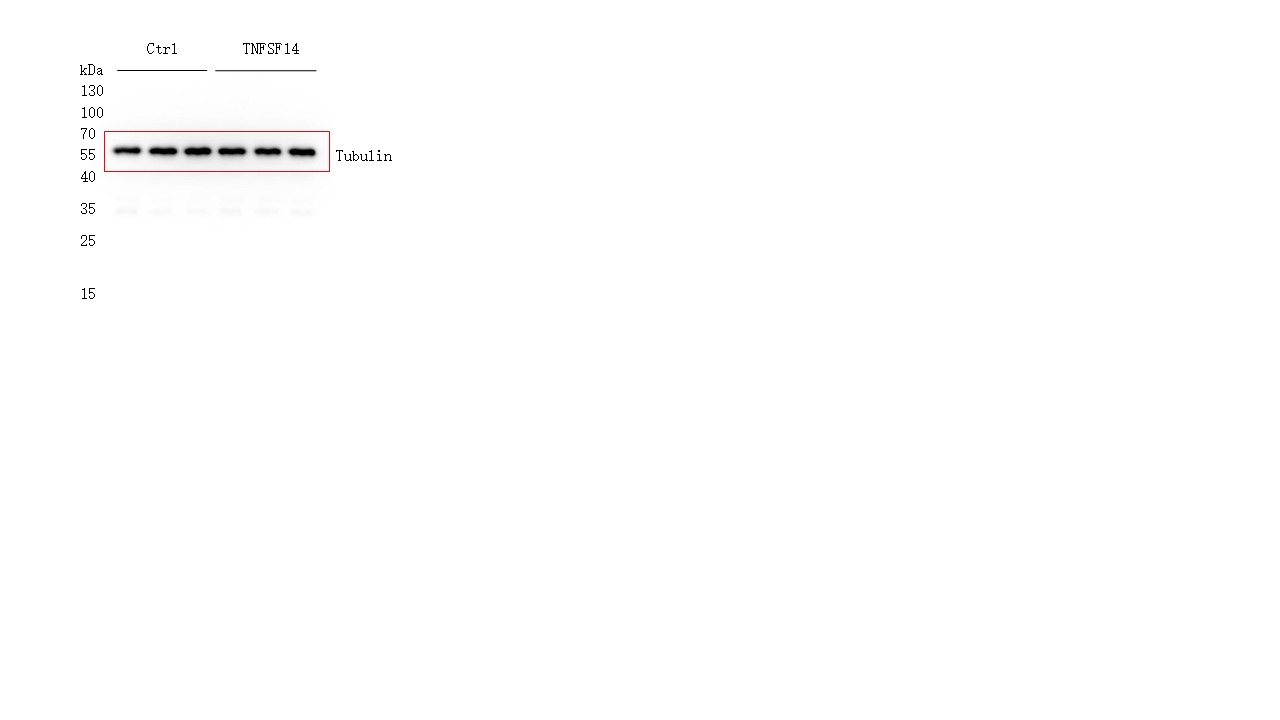

Supplement: Supplementary file 8 — Source data Fig. 6 [file 44318_2024_220_MOESM8_ESM.zip › Figure6/6C/western Tubulin.jpg]

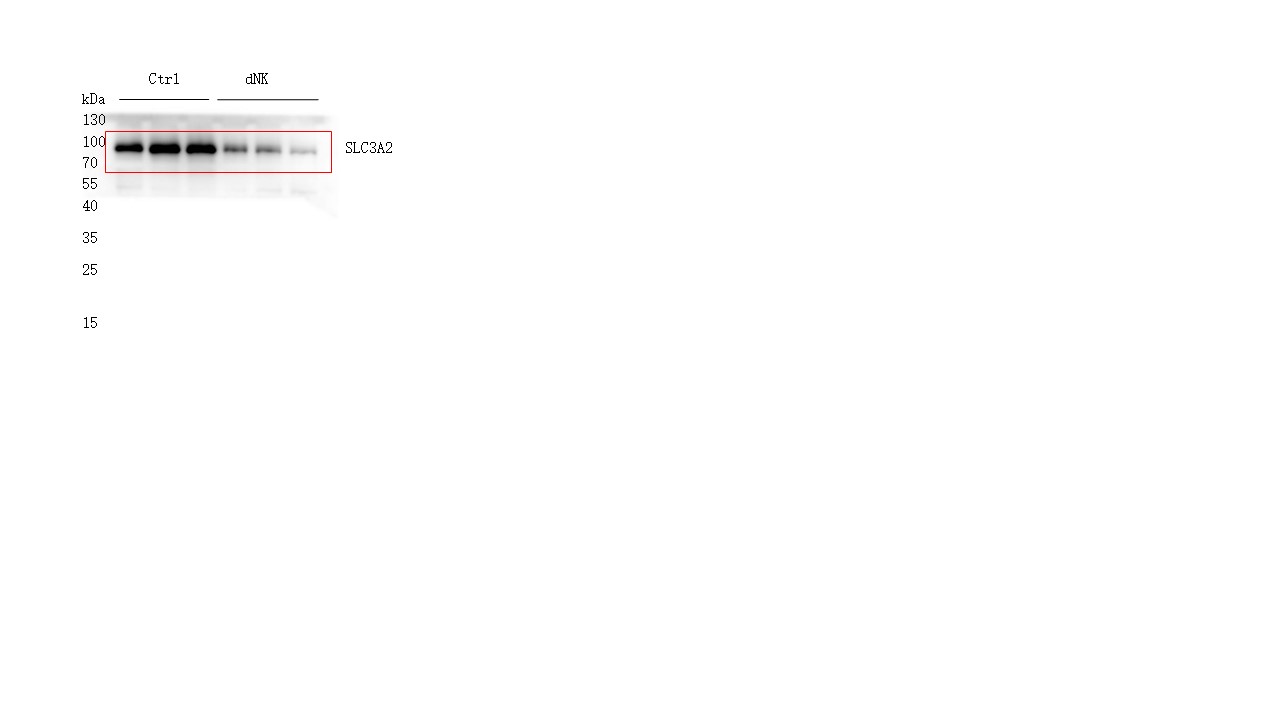

Supplement: Supplementary file 8 — Source data Fig. 6 [file 44318_2024_220_MOESM8_ESM.zip › Figure6/6D/western SLC3A2.jpg]

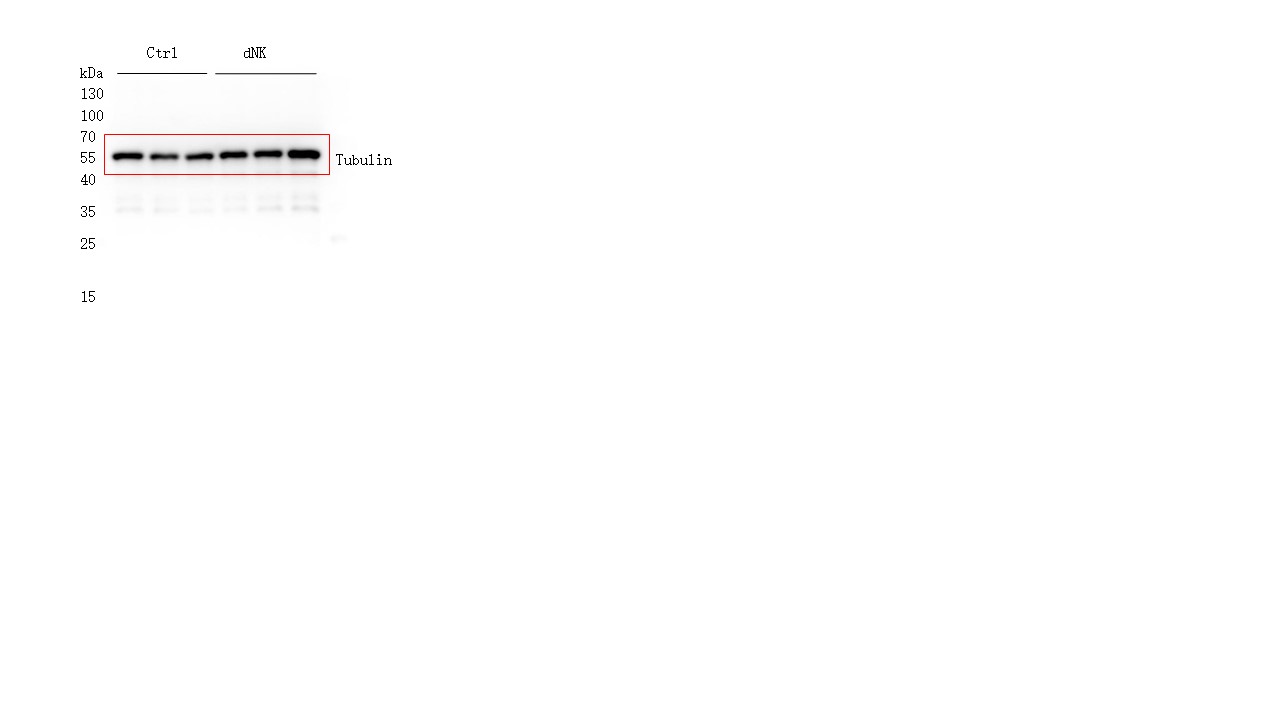

Supplement: Supplementary file 8 — Source data Fig. 6 [file 44318_2024_220_MOESM8_ESM.zip › Figure6/6D/western Tubulin.jpg]

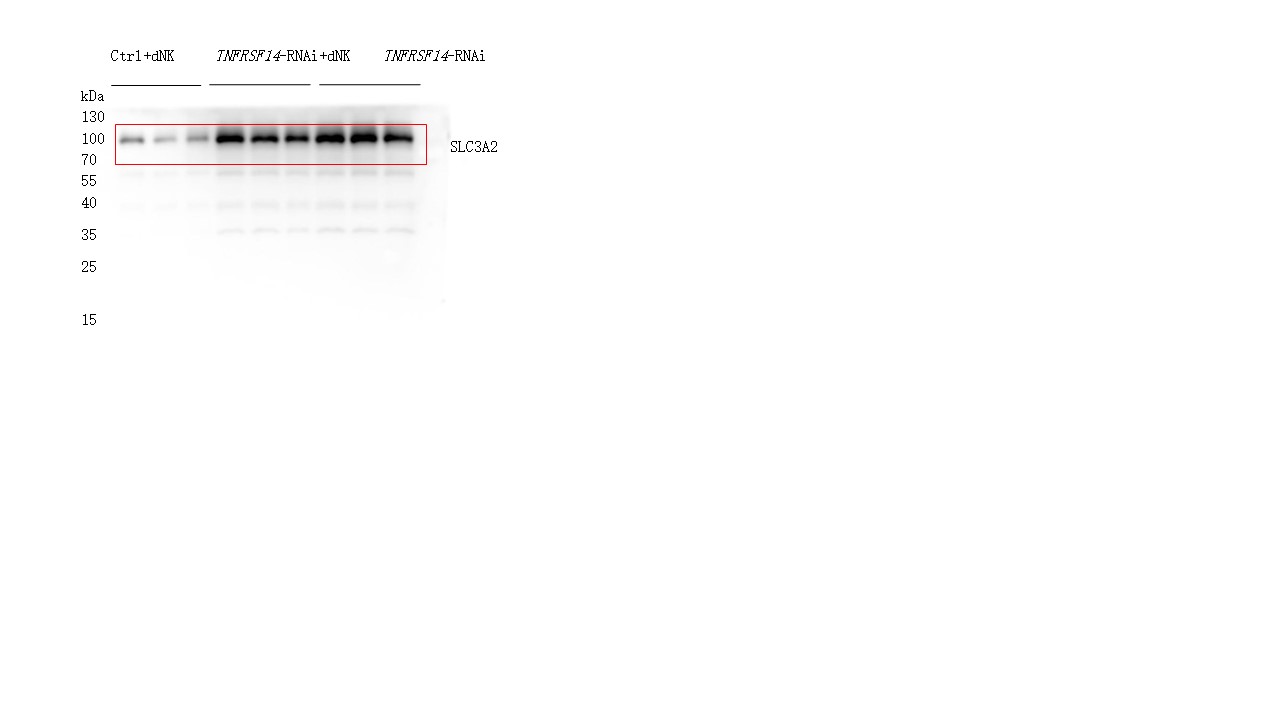

Supplement: Supplementary file 8 — Source data Fig. 6 [file 44318_2024_220_MOESM8_ESM.zip › Figure6/6E/western SLC3A2.jpg]

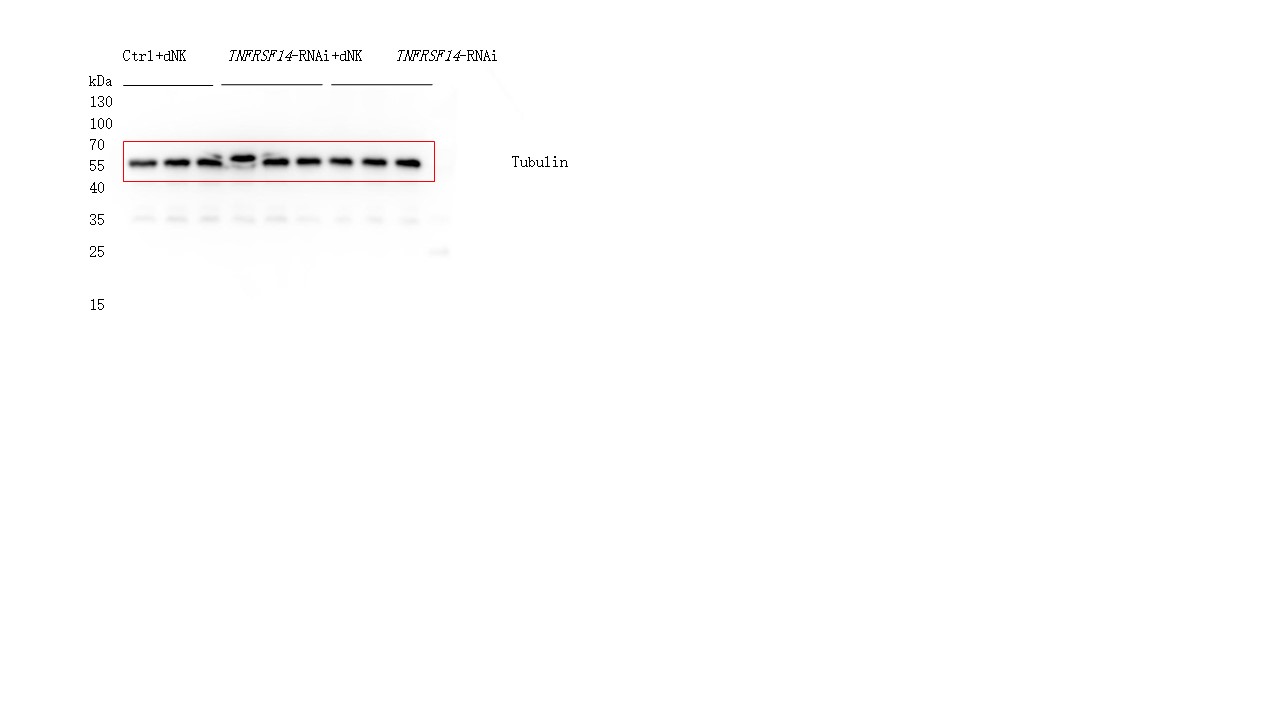

Supplement: Supplementary file 8 — Source data Fig. 6 [file 44318_2024_220_MOESM8_ESM.zip › Figure6/6E/western Tubulin.jpg]

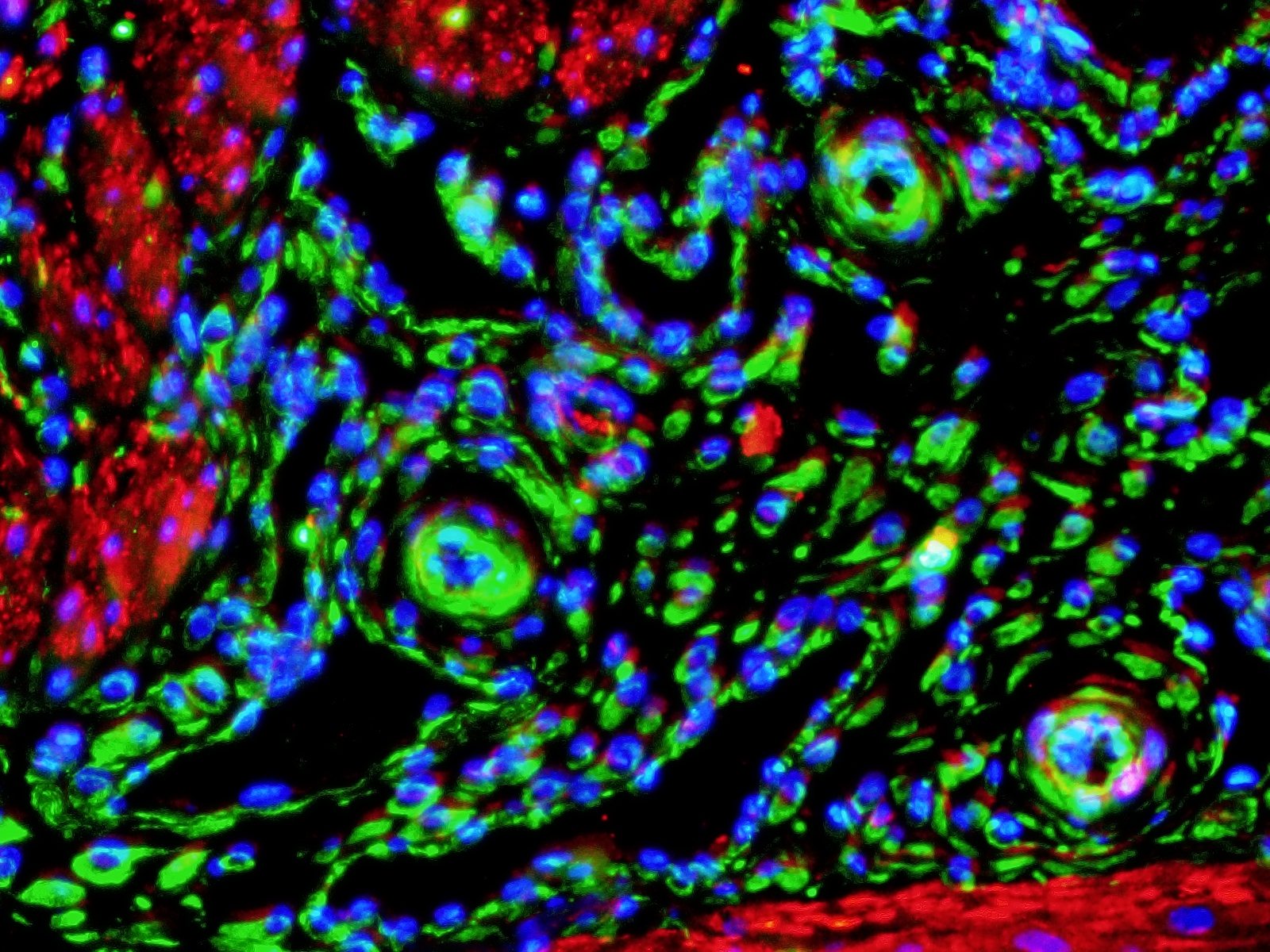

Supplement: Supplementary file 9 — Source data Fig. 7 [file 44318_2024_220_MOESM9_ESM.zip › Figure7/7A/CDKN1A-400-Ctrl (1).jpg]

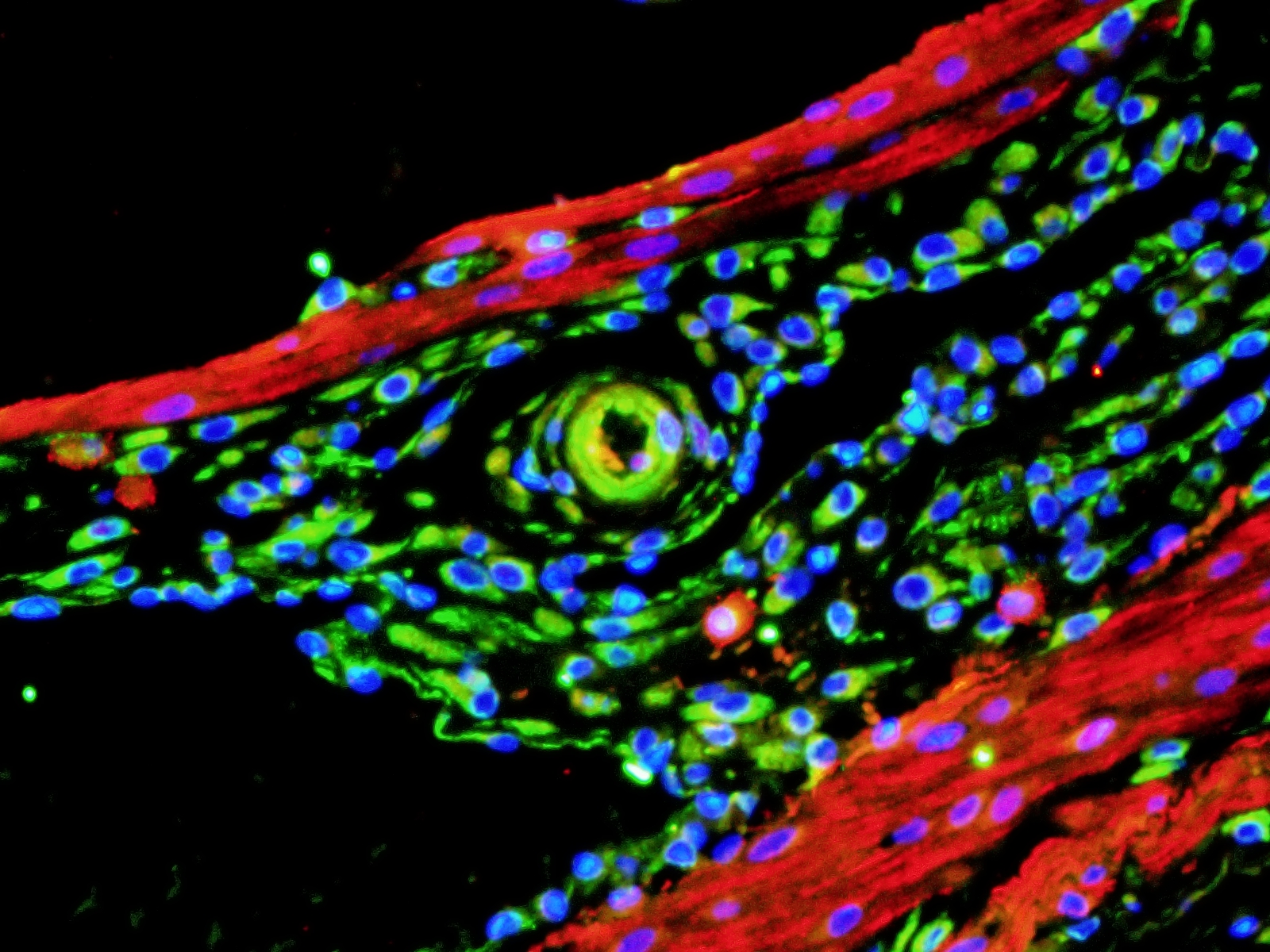

Supplement: Supplementary file 9 — Source data Fig. 7 [file 44318_2024_220_MOESM9_ESM.zip › Figure7/7A/CDKN1A-400-Ctrl (2).jpg]

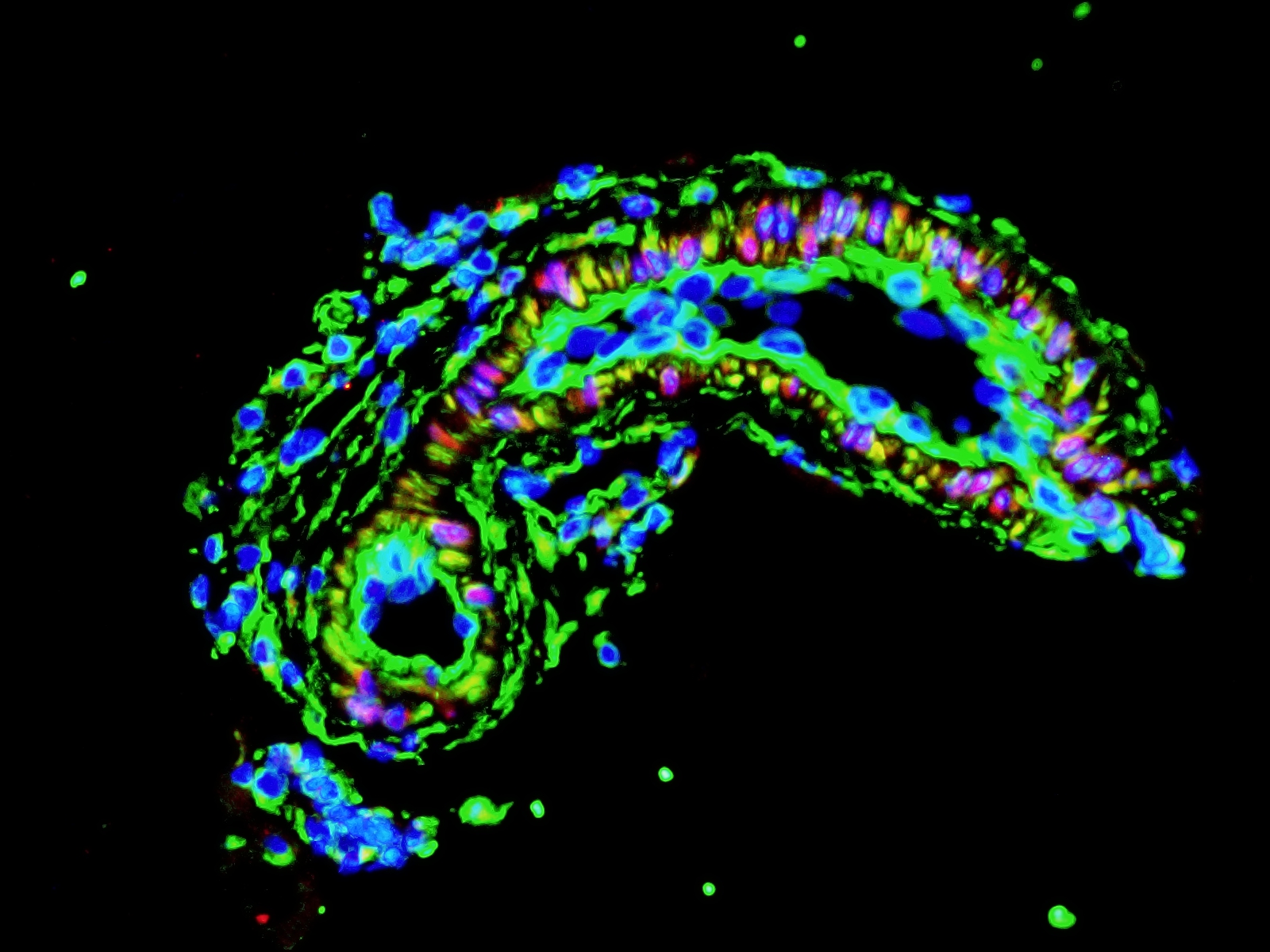

Supplement: Supplementary file 9 — Source data Fig. 7 [file 44318_2024_220_MOESM9_ESM.zip › Figure7/7A/CDKN1A-400-Ctrl (3).jpg]

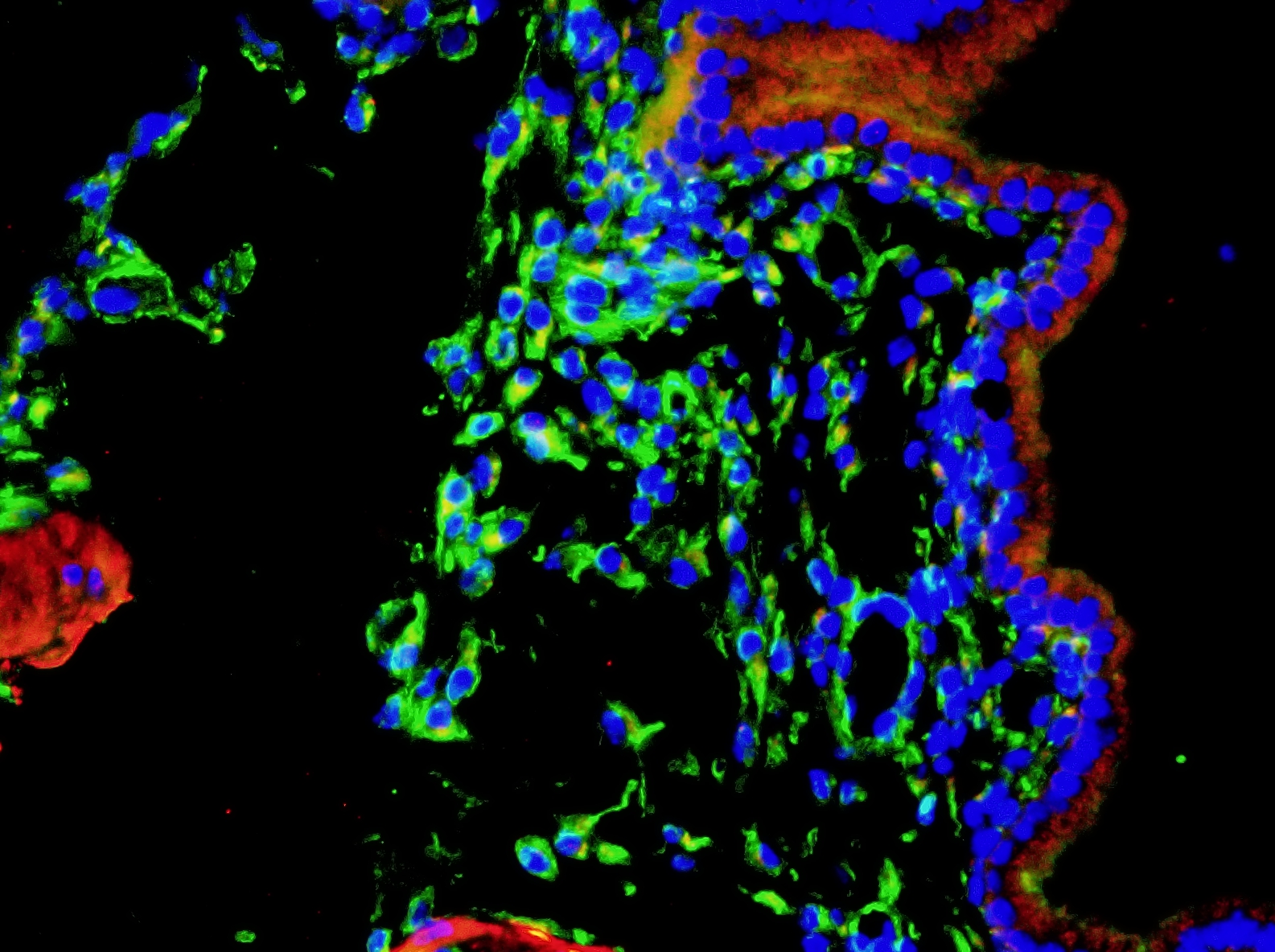

Supplement: Supplementary file 9 — Source data Fig. 7 [file 44318_2024_220_MOESM9_ESM.zip › Figure7/7A/CDKN1A-400-Ctrl (4).jpg]

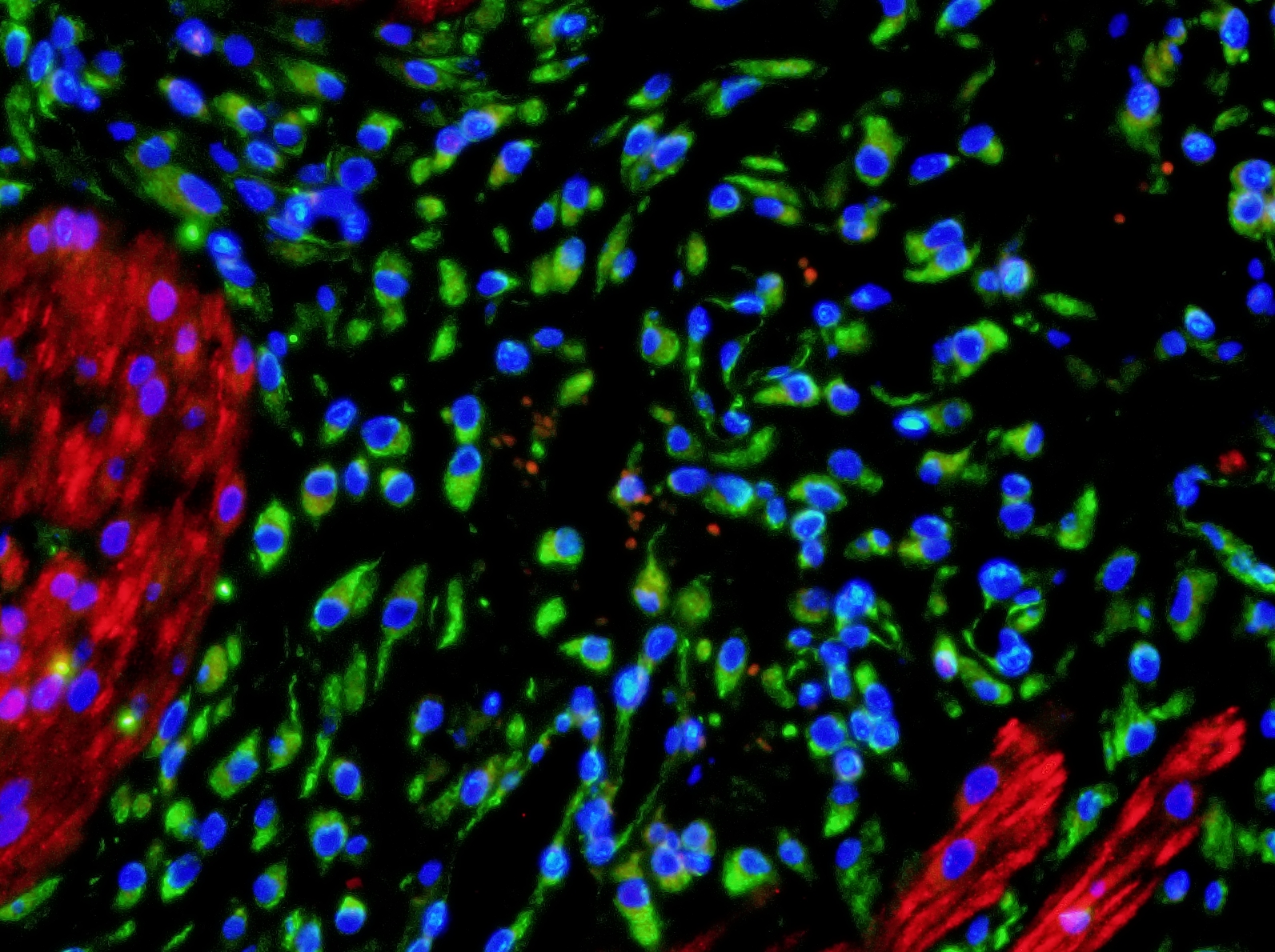

Supplement: Supplementary file 9 — Source data Fig. 7 [file 44318_2024_220_MOESM9_ESM.zip › Figure7/7A/CDKN1A-400-Ctrl (5).jpg]

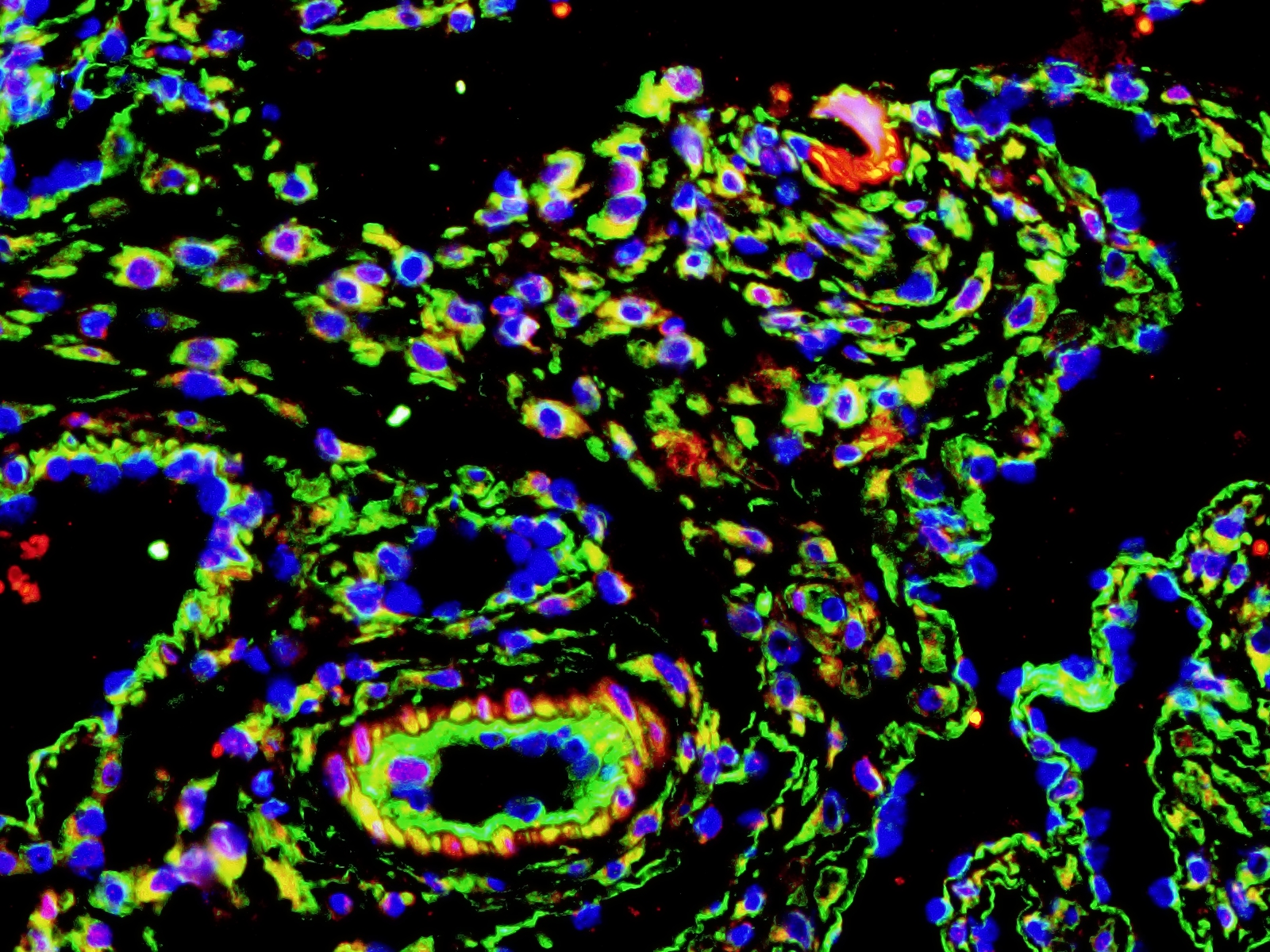

Supplement: Supplementary file 9 — Source data Fig. 7 [file 44318_2024_220_MOESM9_ESM.zip › Figure7/7A/CDKN1A-400-NK1.1 (1).jpg]

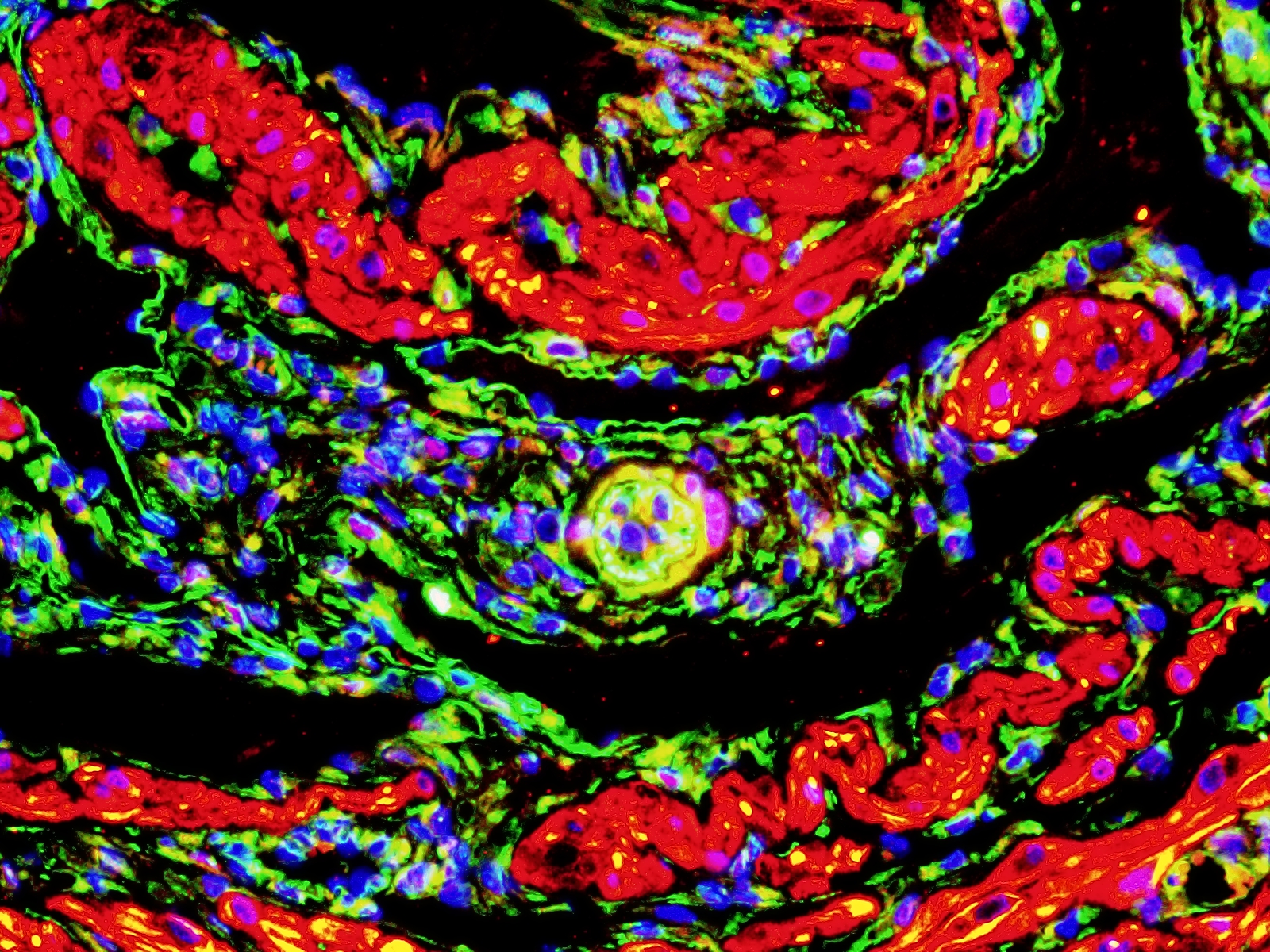

Supplement: Supplementary file 9 — Source data Fig. 7 [file 44318_2024_220_MOESM9_ESM.zip › Figure7/7A/CDKN1A-400-NK1.1 (2).jpg]

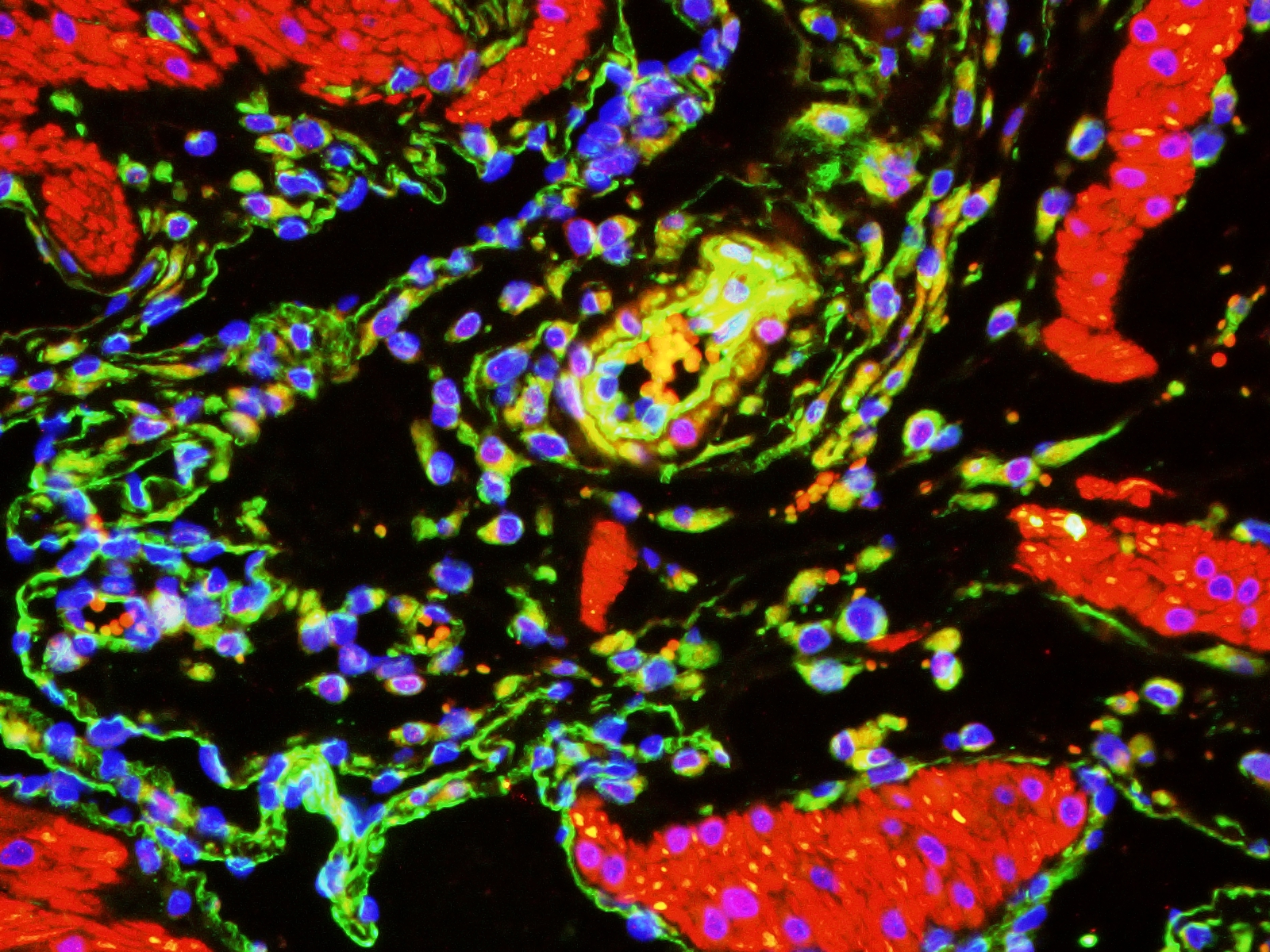

Supplement: Supplementary file 9 — Source data Fig. 7 [file 44318_2024_220_MOESM9_ESM.zip › Figure7/7A/CDKN1A-400-NK1.1 (3).jpg]

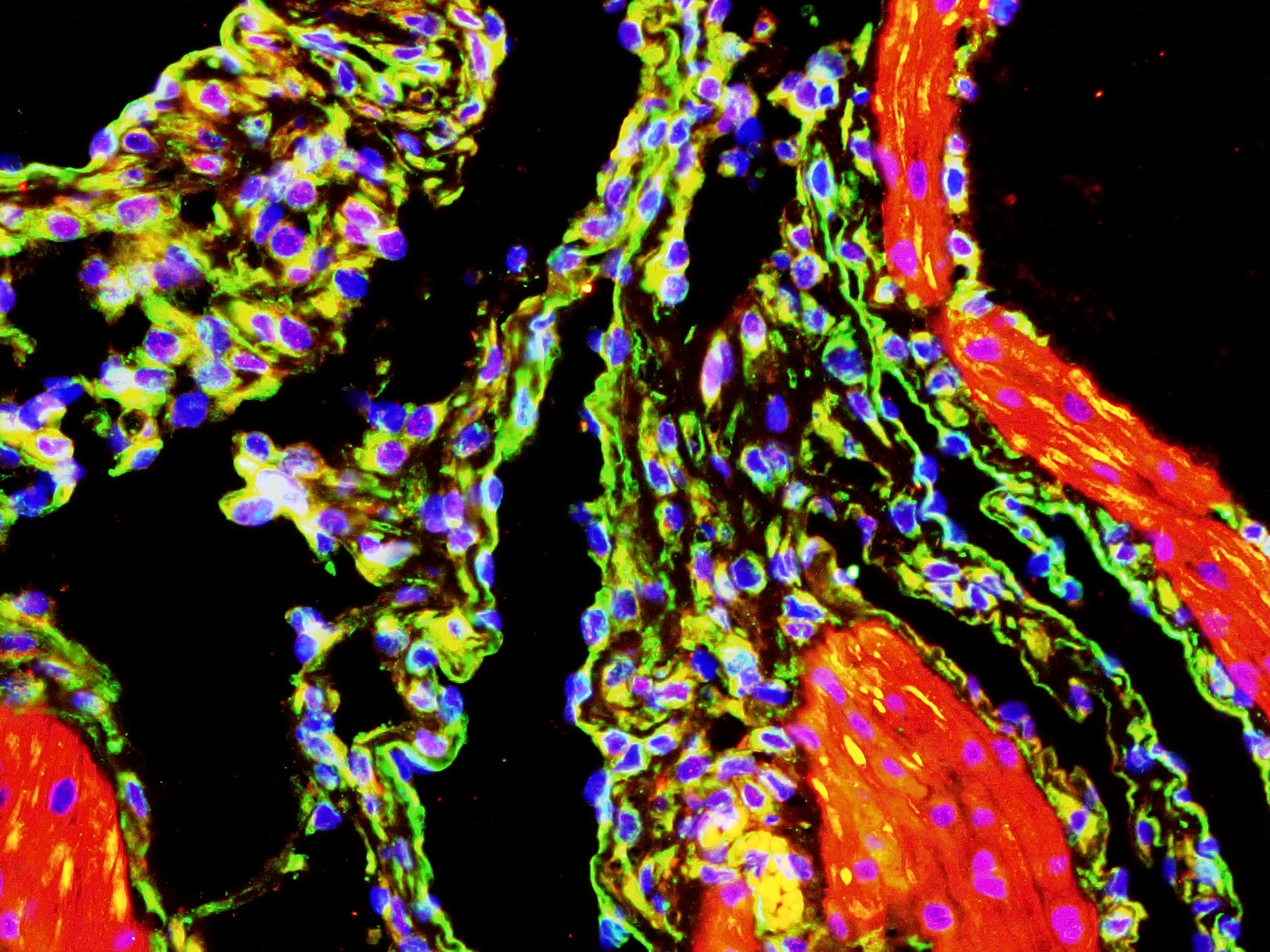

Supplement: Supplementary file 9 — Source data Fig. 7 [file 44318_2024_220_MOESM9_ESM.zip › Figure7/7A/CDKN1A-400-NK1.1 (4).jpg]

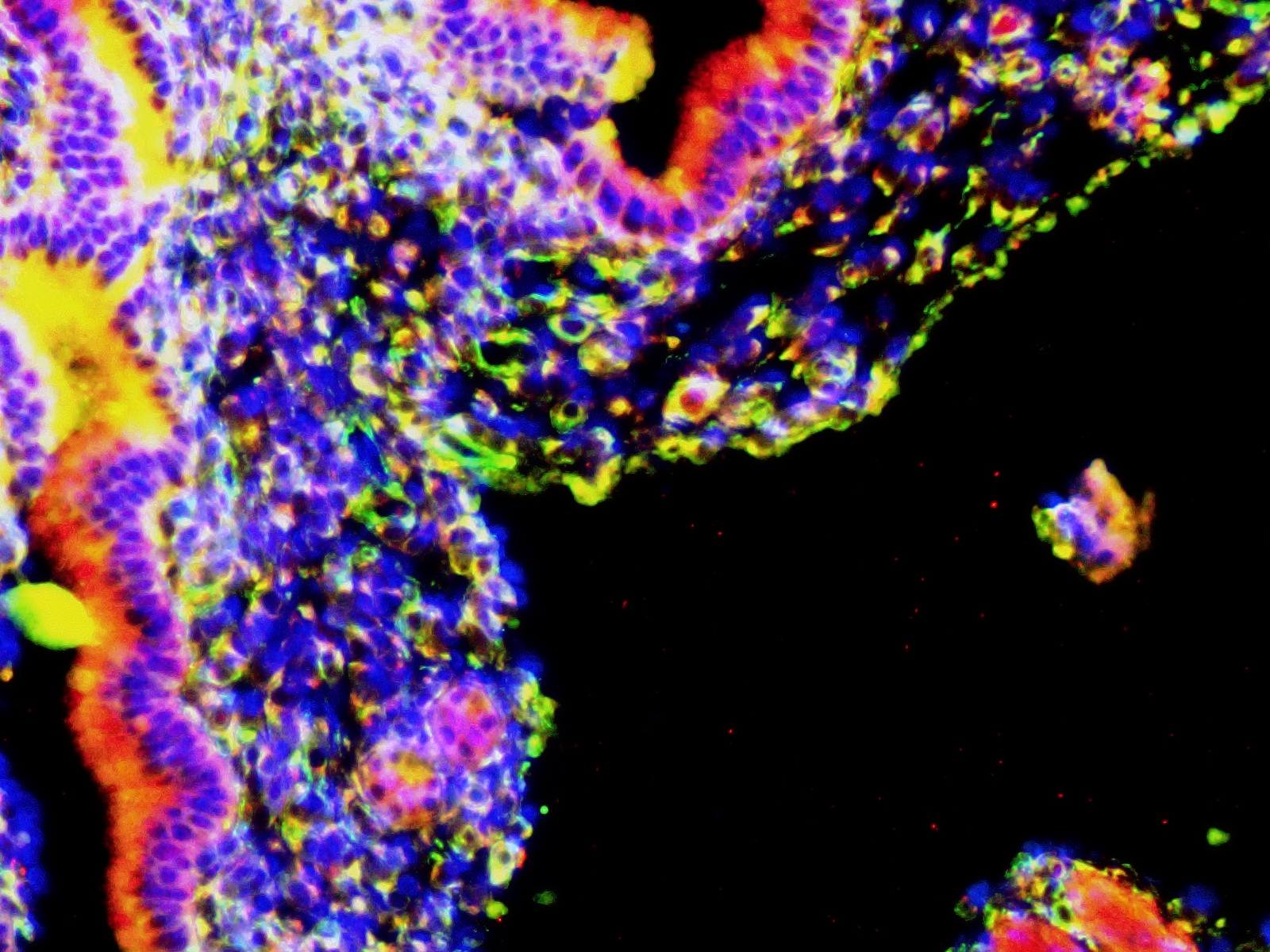

Supplement: Supplementary file 9 — Source data Fig. 7 [file 44318_2024_220_MOESM9_ESM.zip › Figure7/7A/CDKN1A-400-NK1.1 (5).jpg]

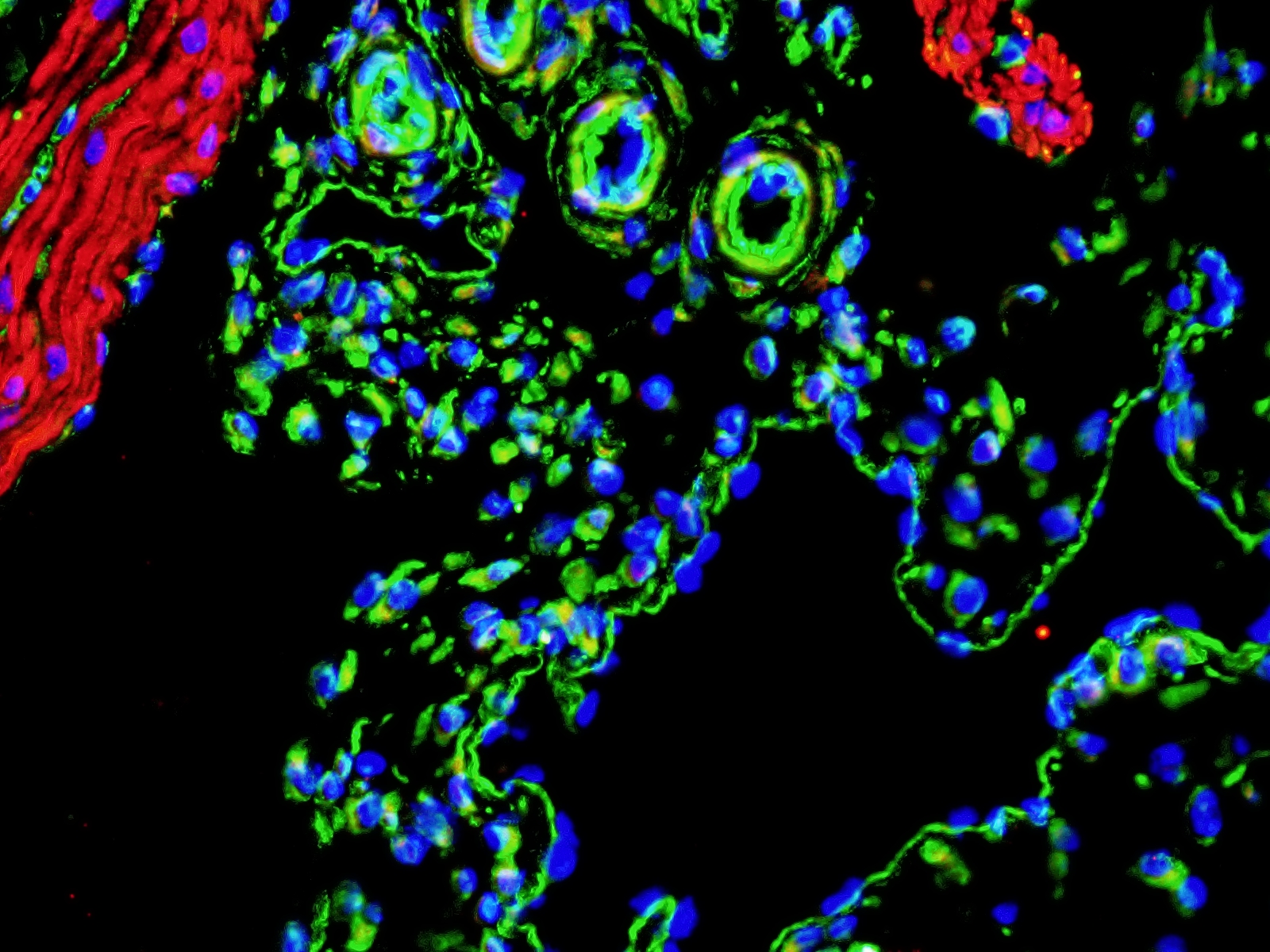

Supplement: Supplementary file 9 — Source data Fig. 7 [file 44318_2024_220_MOESM9_ESM.zip › Figure7/7A/CDKN1A-400-TNFSF14 (1).jpg]

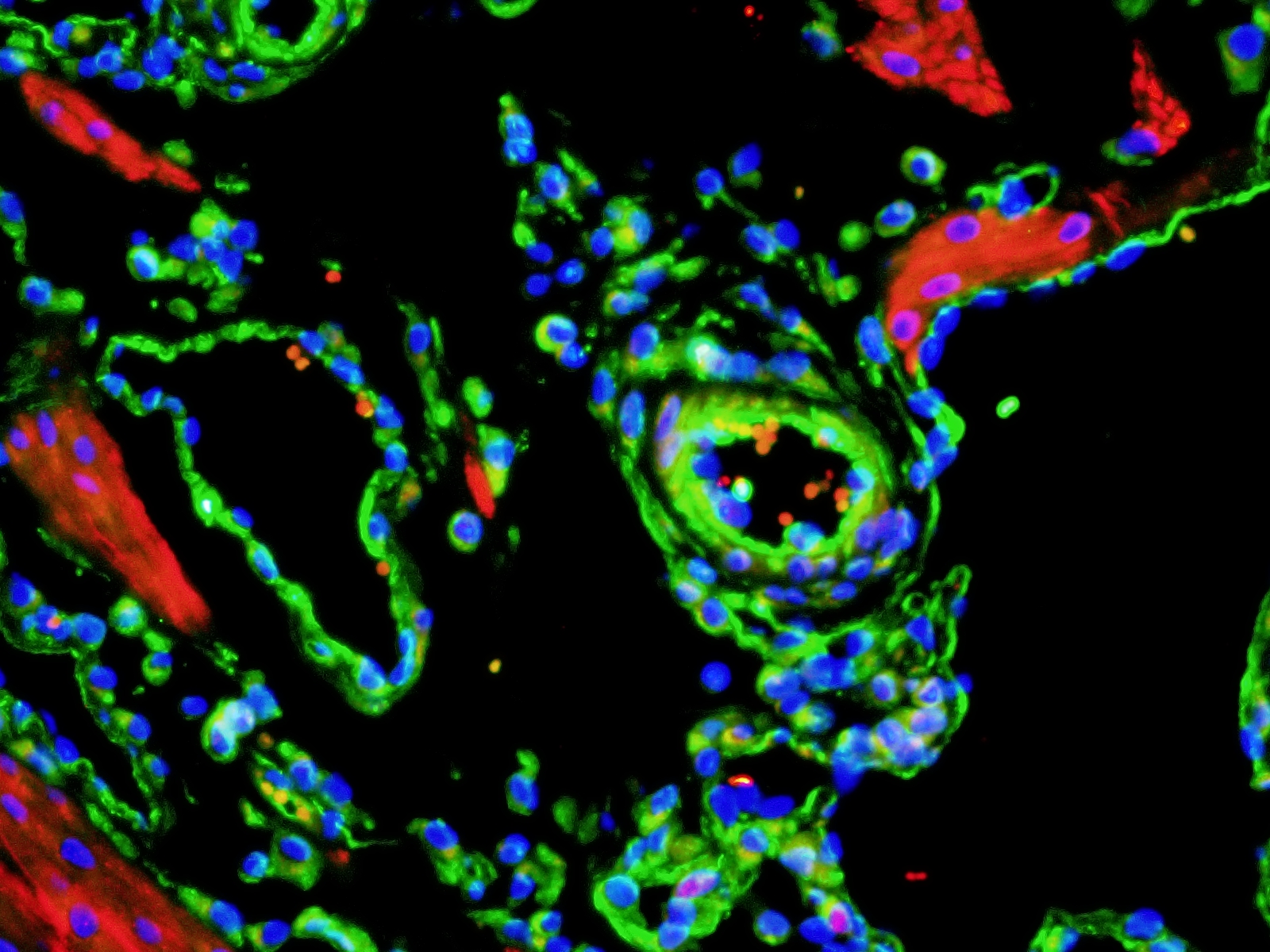

Supplement: Supplementary file 9 — Source data Fig. 7 [file 44318_2024_220_MOESM9_ESM.zip › Figure7/7A/CDKN1A-400-TNFSF14 (2).jpg]

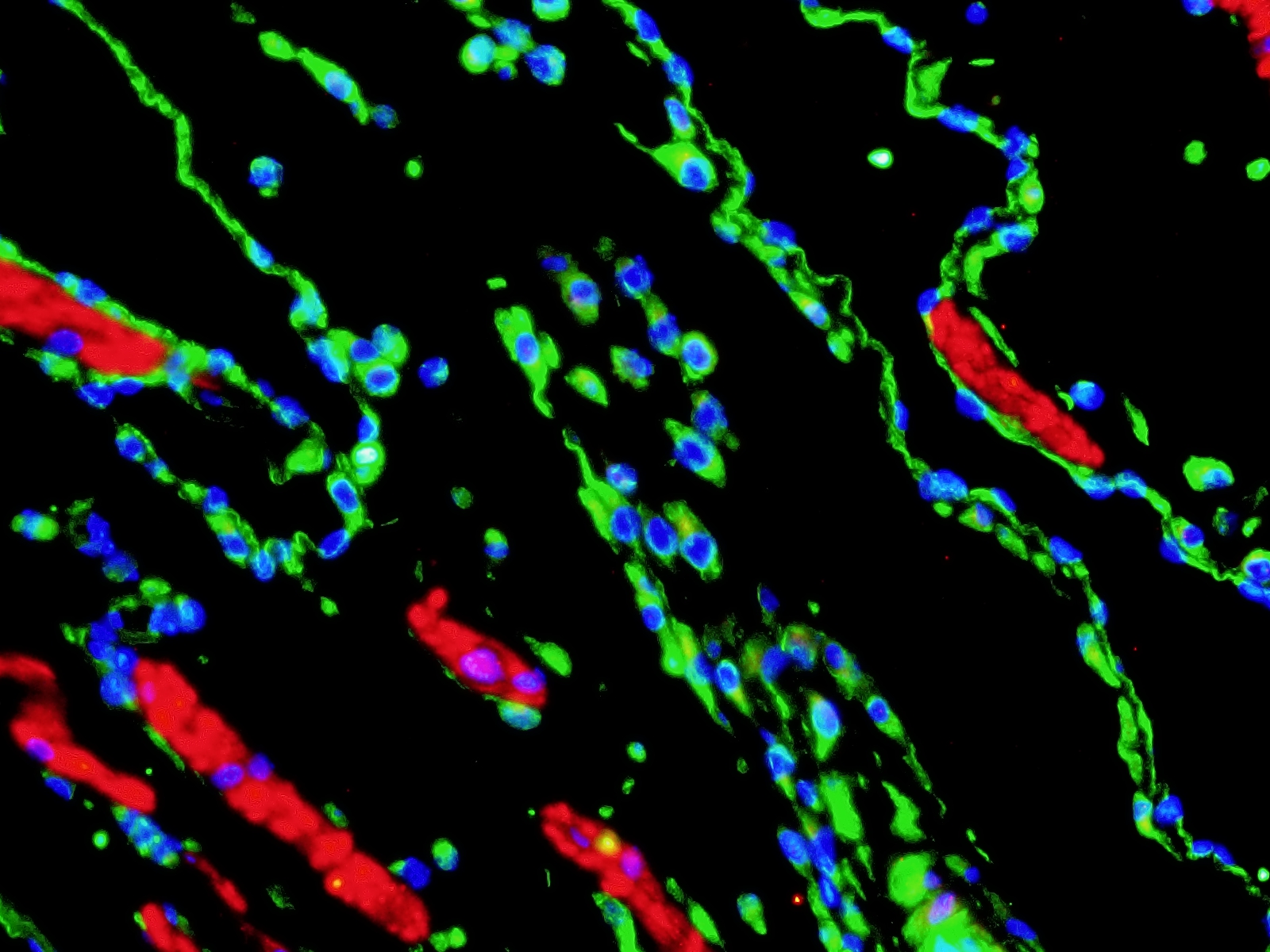

Supplement: Supplementary file 9 — Source data Fig. 7 [file 44318_2024_220_MOESM9_ESM.zip › Figure7/7A/CDKN1A-400-TNFSF14 (3).jpg]

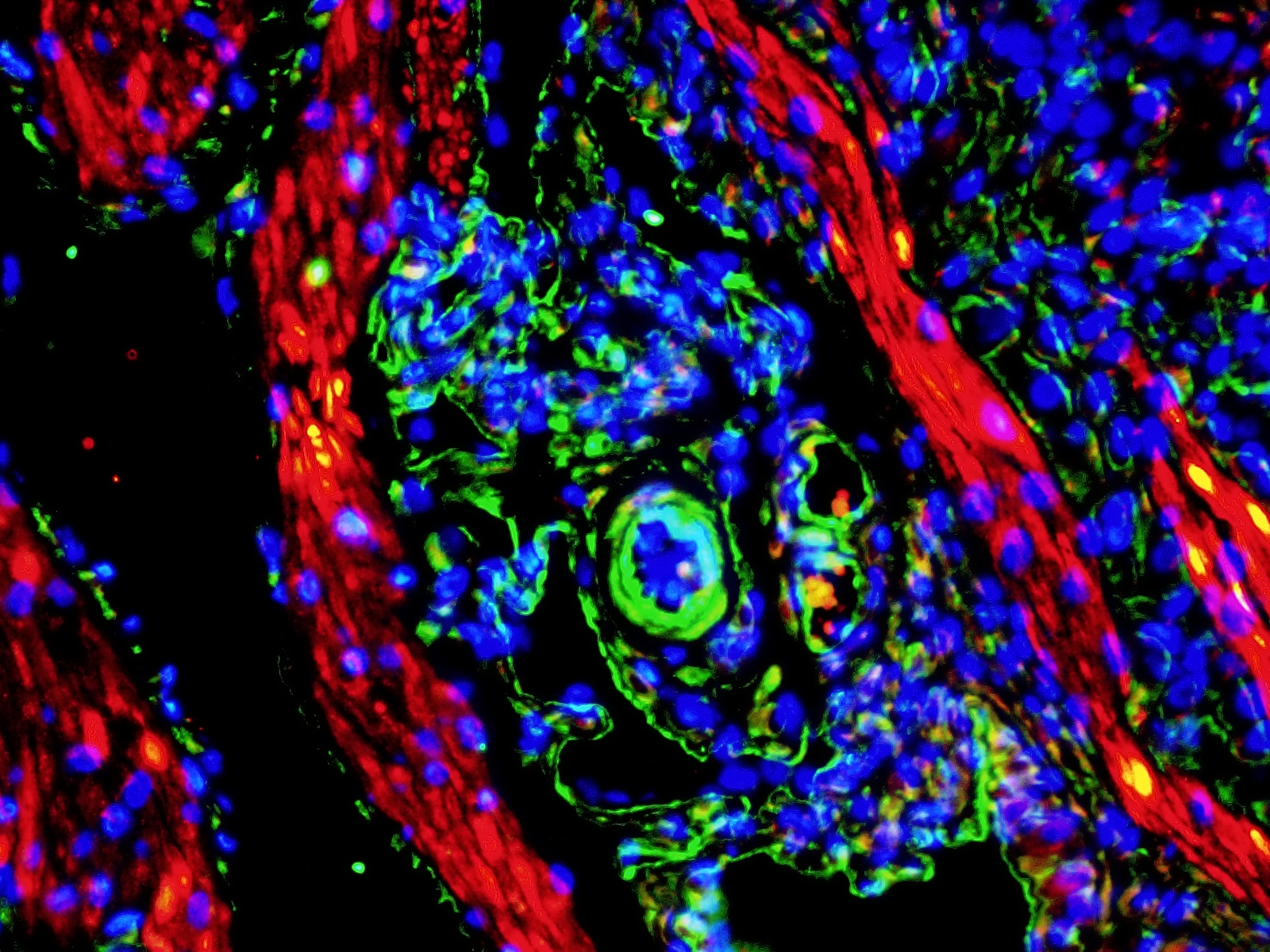

Supplement: Supplementary file 9 — Source data Fig. 7 [file 44318_2024_220_MOESM9_ESM.zip › Figure7/7A/CDKN1A-400-TNFSF14 (4).jpg]

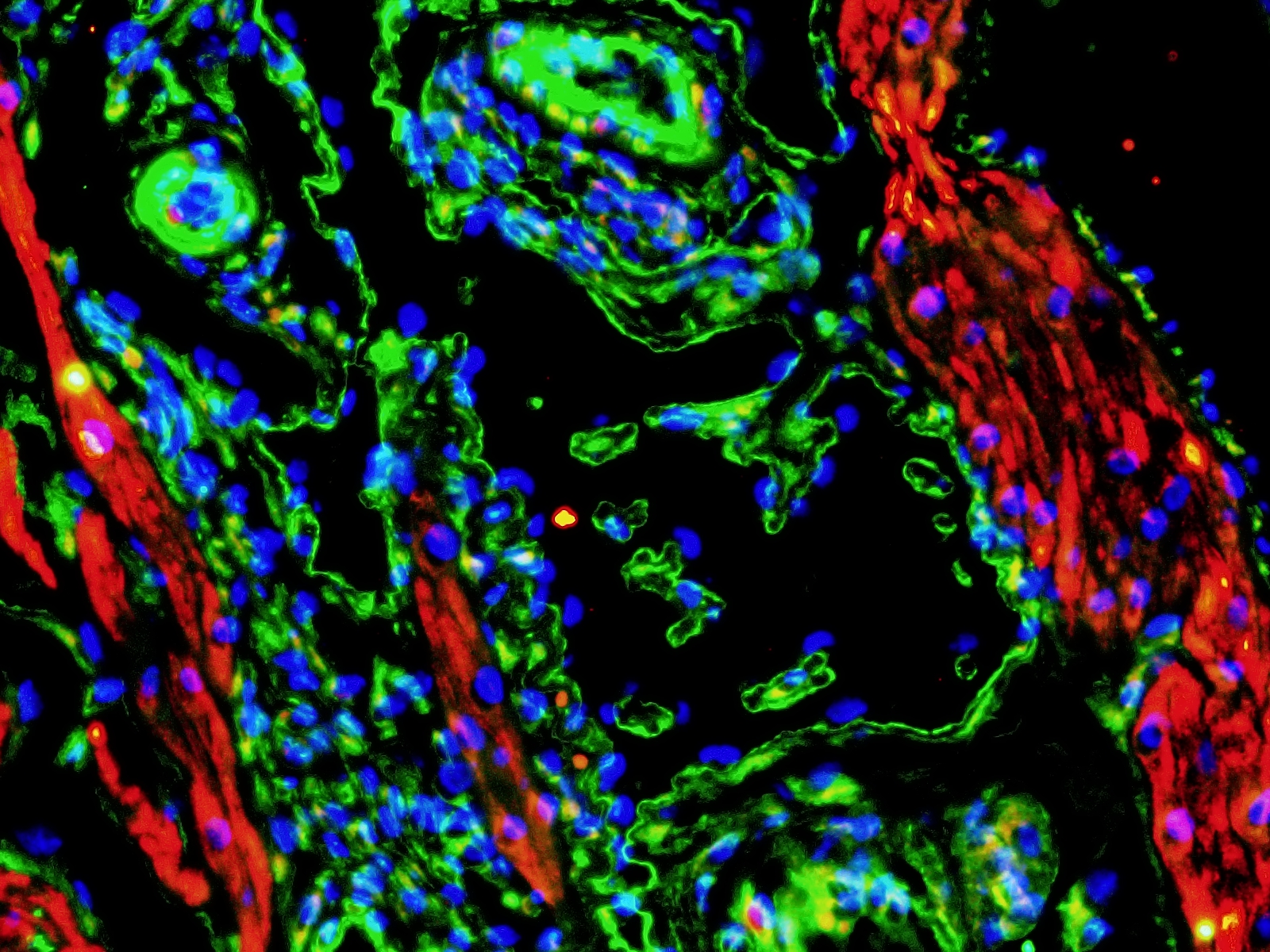

Supplement: Supplementary file 9 — Source data Fig. 7 [file 44318_2024_220_MOESM9_ESM.zip › Figure7/7A/CDKN1A-400-TNFSF14 (5).jpg]

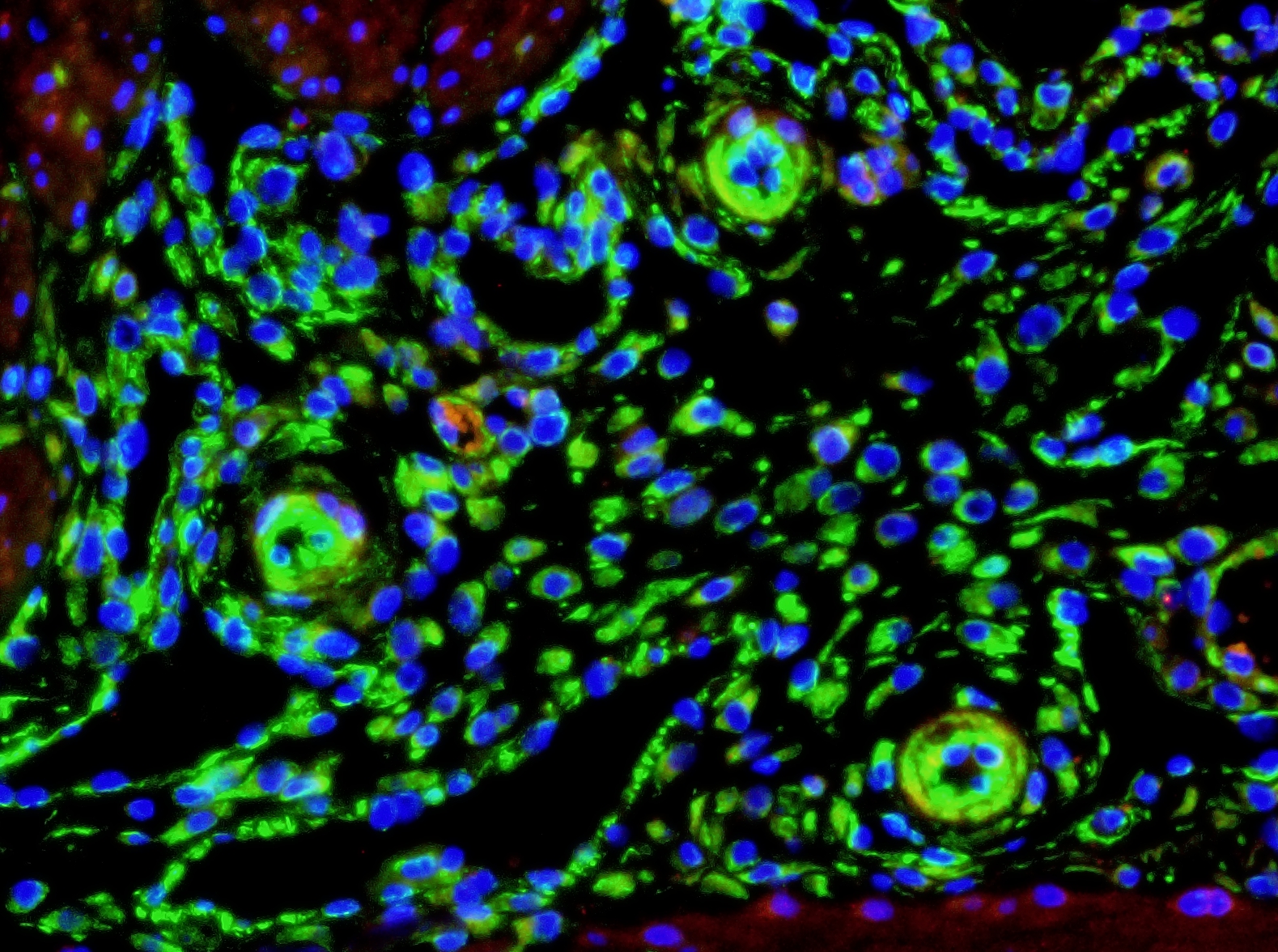

Supplement: Supplementary file 9 — Source data Fig. 7 [file 44318_2024_220_MOESM9_ESM.zip › Figure7/7A/CDKN2A-400-Ctrl (1).jpg]

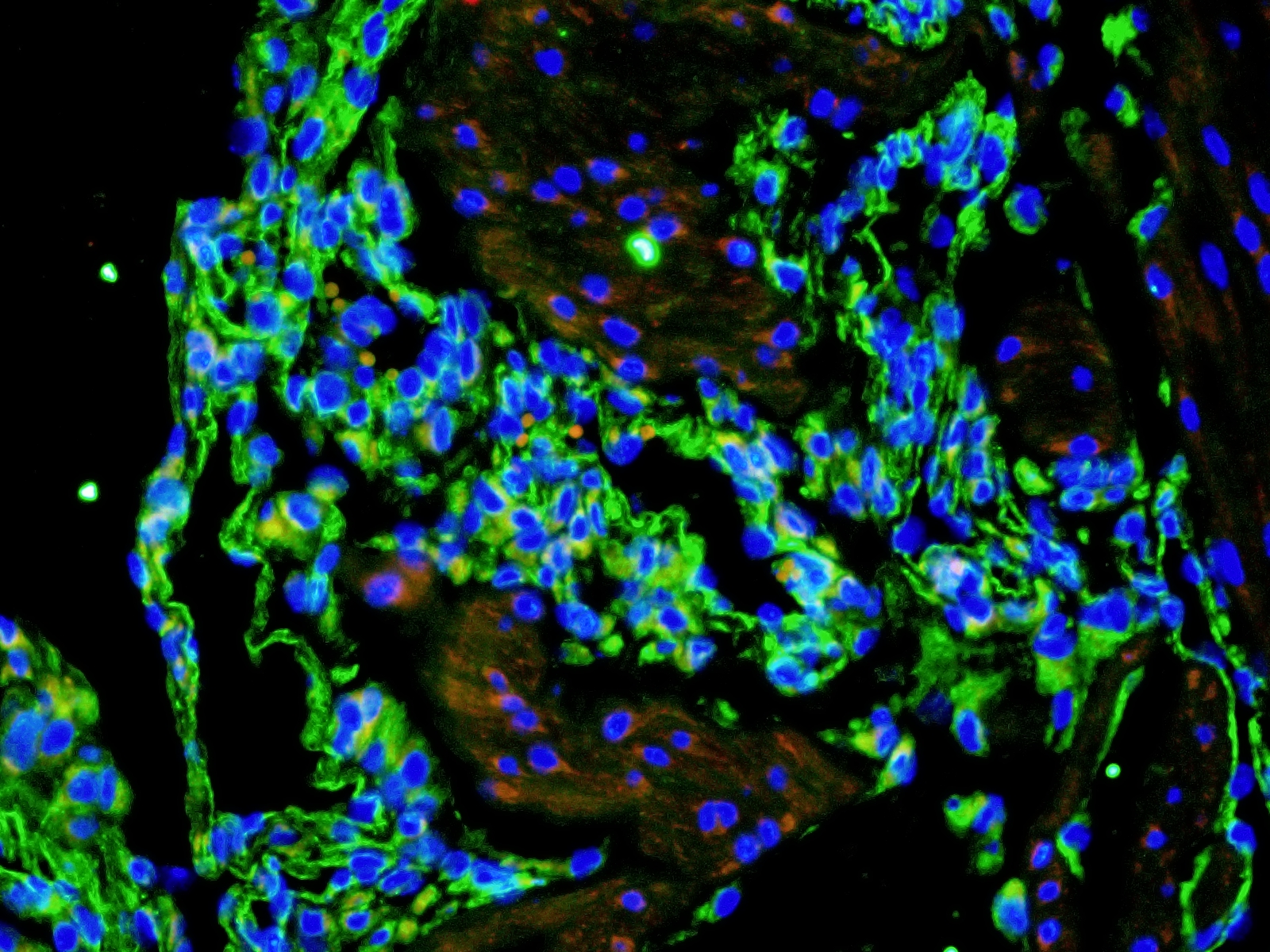

Supplement: Supplementary file 9 — Source data Fig. 7 [file 44318_2024_220_MOESM9_ESM.zip › Figure7/7A/CDKN2A-400-Ctrl (2).jpg]

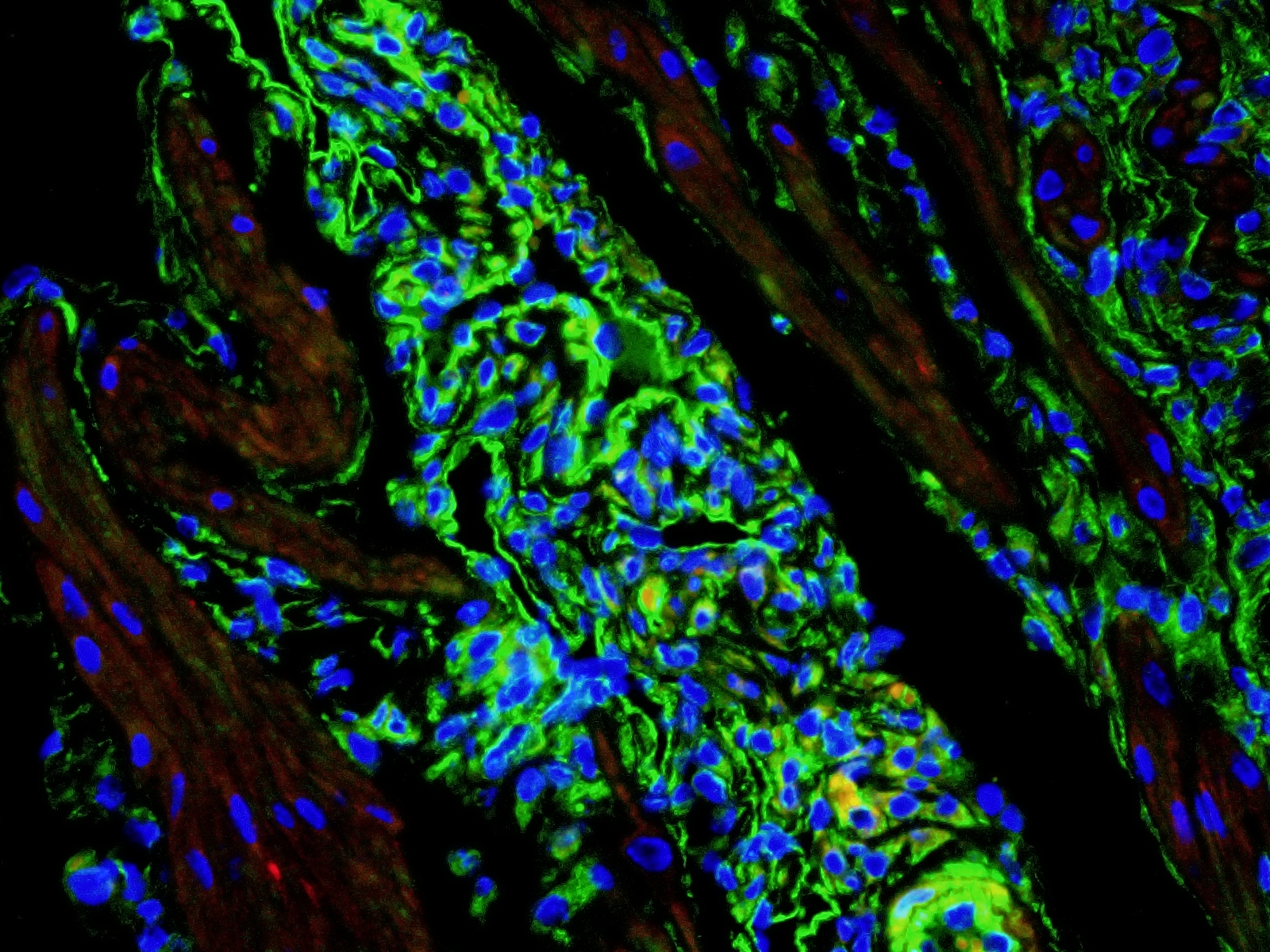

Supplement: Supplementary file 9 — Source data Fig. 7 [file 44318_2024_220_MOESM9_ESM.zip › Figure7/7A/CDKN2A-400-Ctrl (3).jpg]

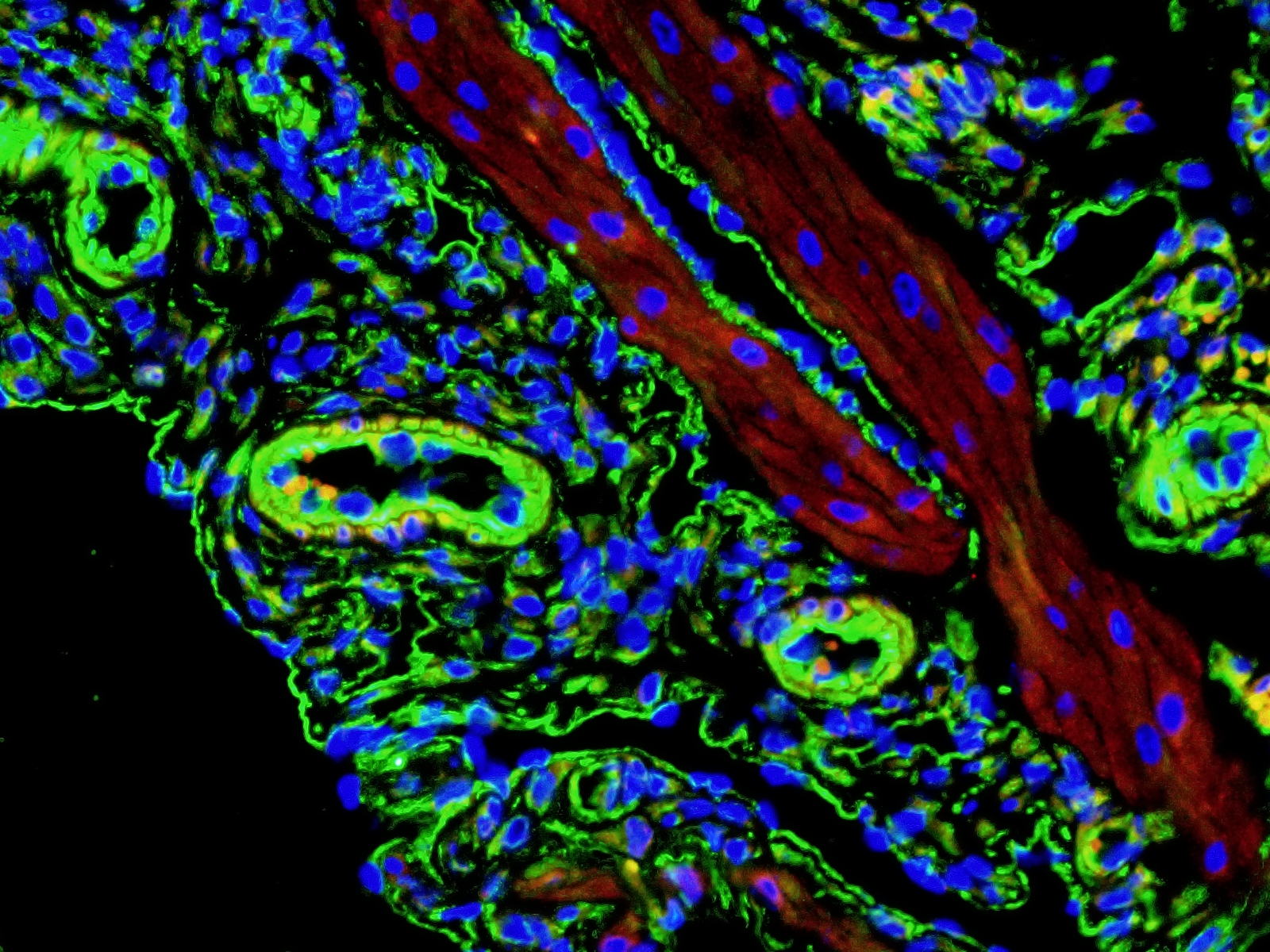

Supplement: Supplementary file 9 — Source data Fig. 7 [file 44318_2024_220_MOESM9_ESM.zip › Figure7/7A/CDKN2A-400-Ctrl (4).jpg]

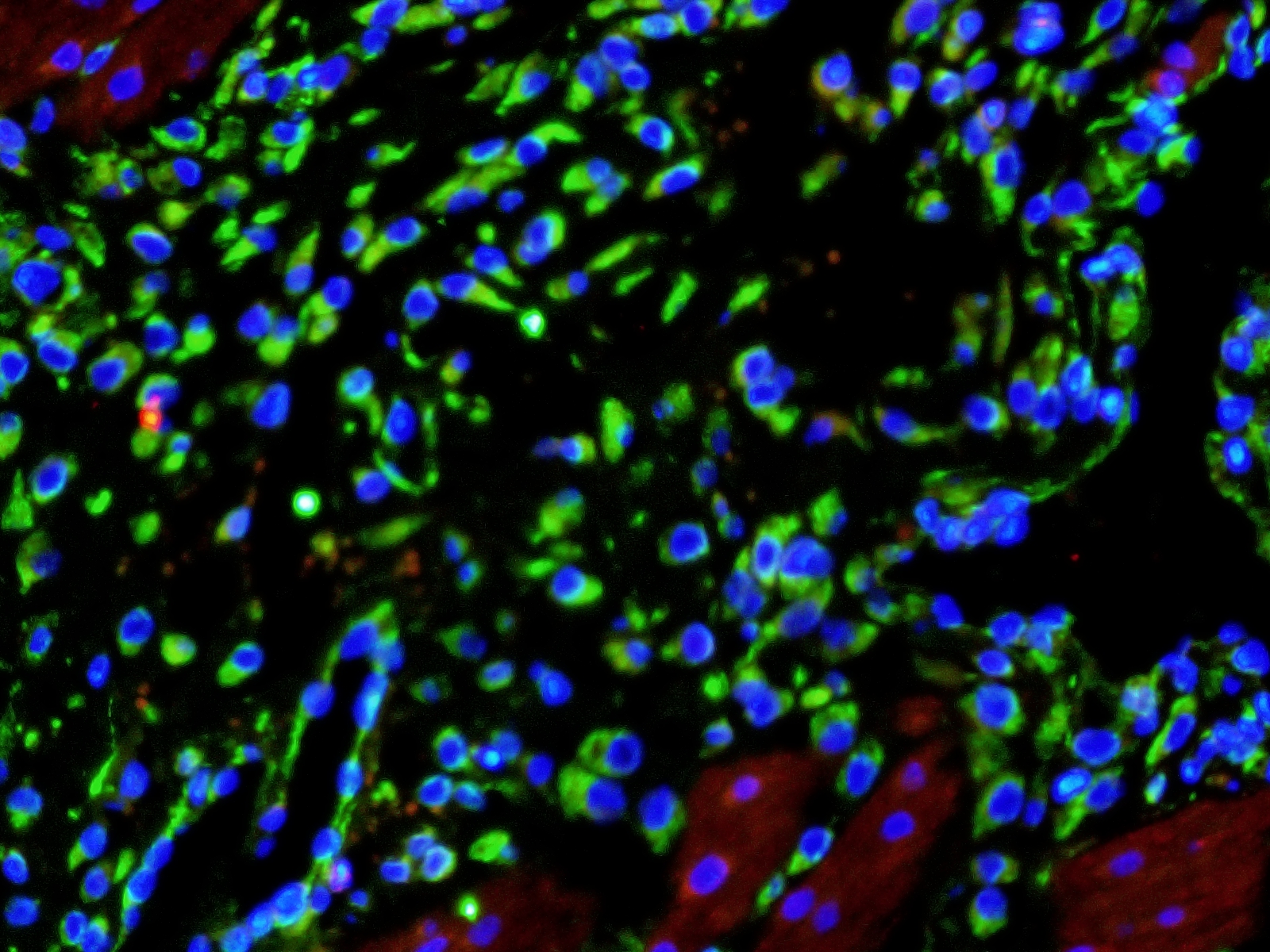

Supplement: Supplementary file 9 — Source data Fig. 7 [file 44318_2024_220_MOESM9_ESM.zip › Figure7/7A/CDKN2A-400-Ctrl (5).jpg]

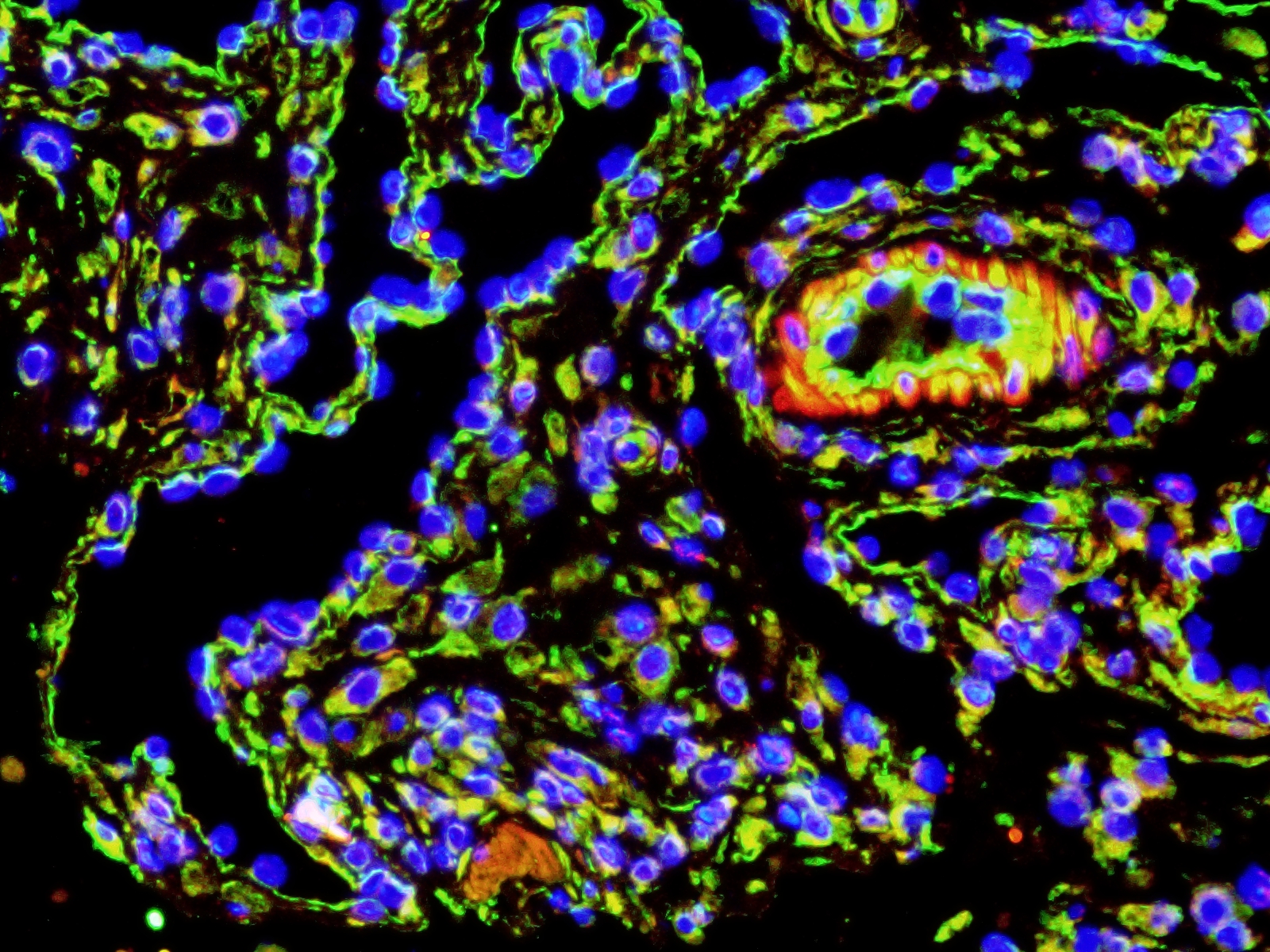

Supplement: Supplementary file 9 — Source data Fig. 7 [file 44318_2024_220_MOESM9_ESM.zip › Figure7/7A/CDKN2A-400-NK1.1 (1).jpg]

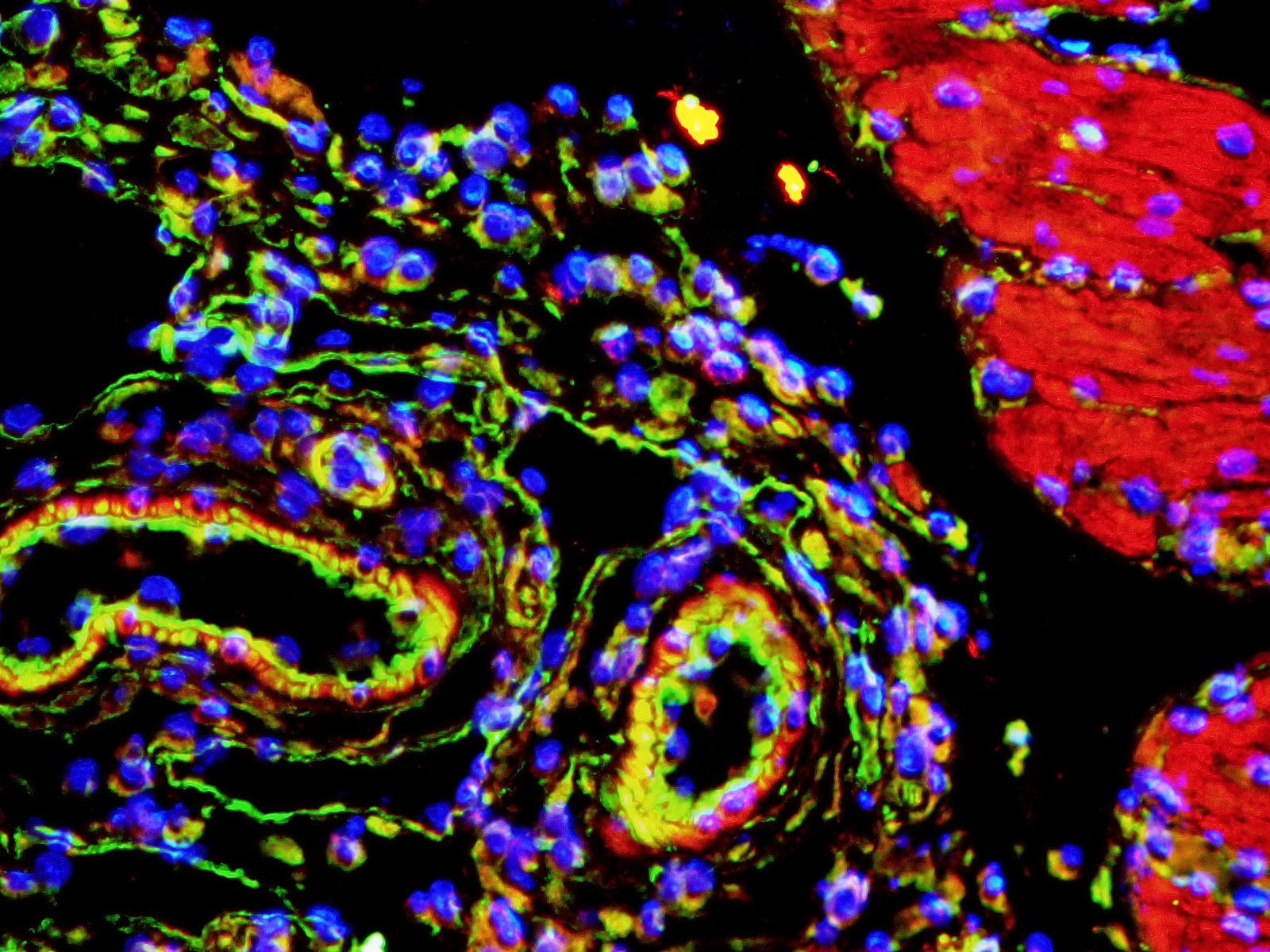

Supplement: Supplementary file 9 — Source data Fig. 7 [file 44318_2024_220_MOESM9_ESM.zip › Figure7/7A/CDKN2A-400-NK1.1 (2).jpg]

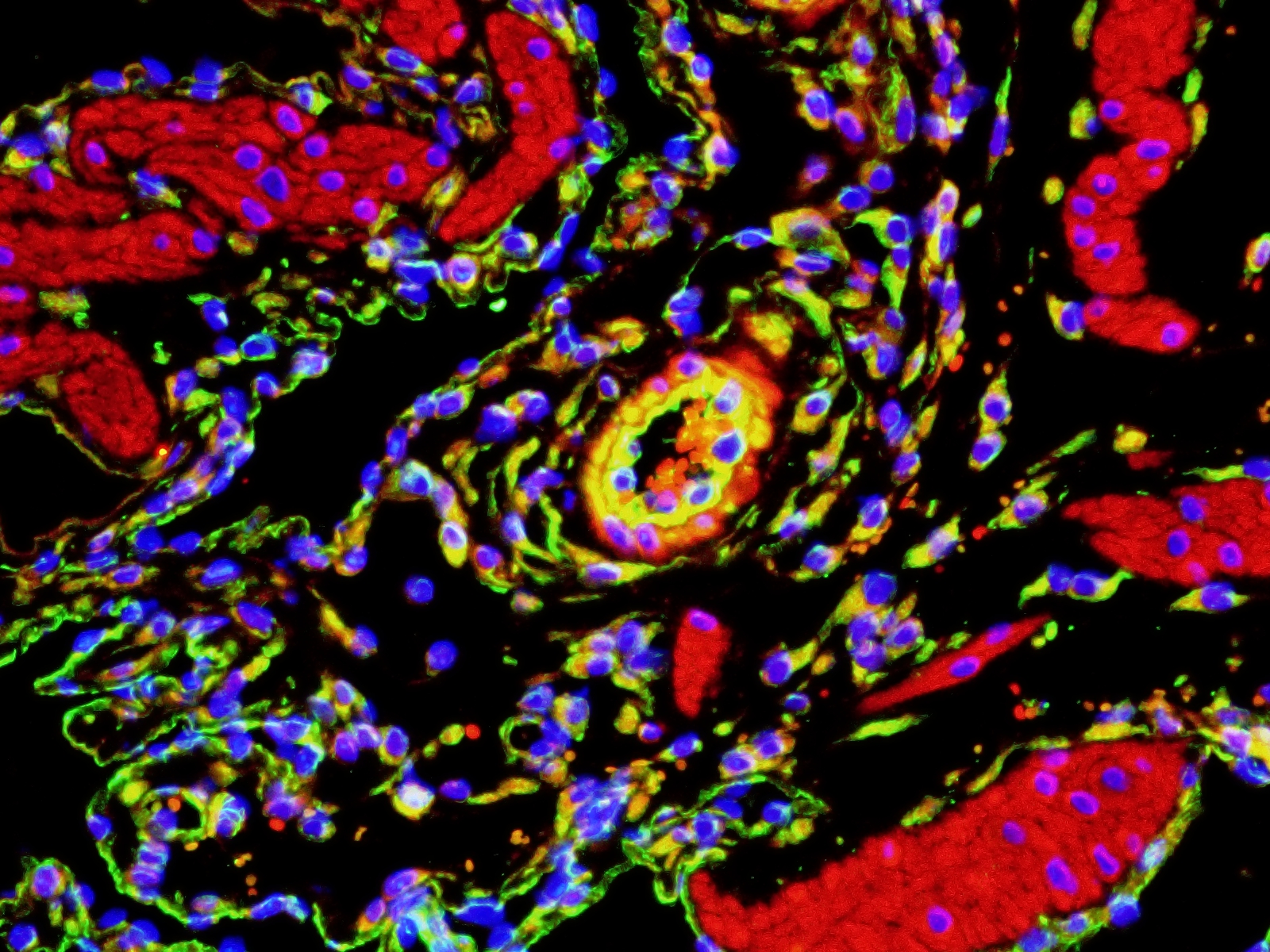

Supplement: Supplementary file 9 — Source data Fig. 7 [file 44318_2024_220_MOESM9_ESM.zip › Figure7/7A/CDKN2A-400-NK1.1 (3).jpg]

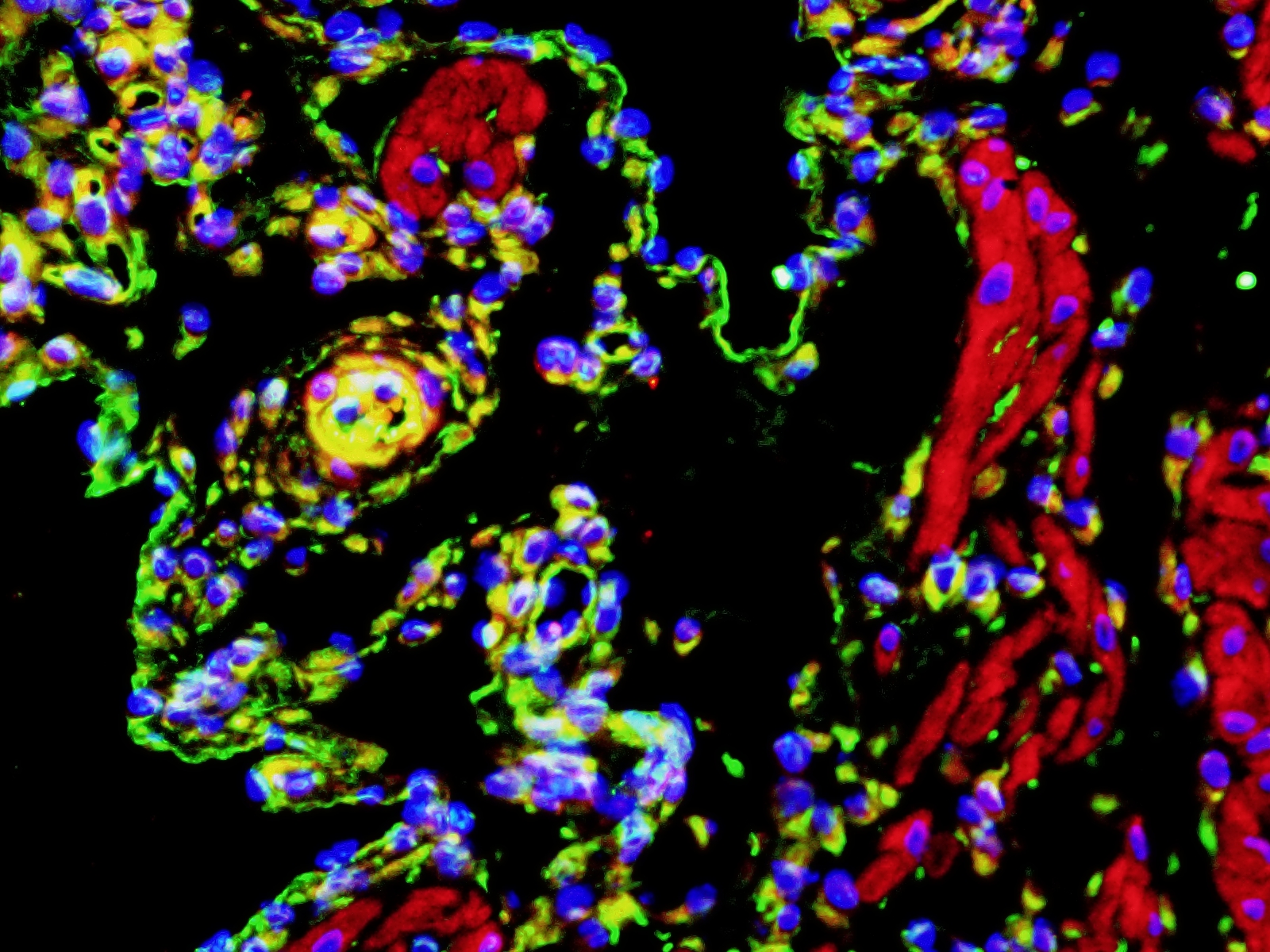

Supplement: Supplementary file 9 — Source data Fig. 7 [file 44318_2024_220_MOESM9_ESM.zip › Figure7/7A/CDKN2A-400-NK1.1 (4).jpg]

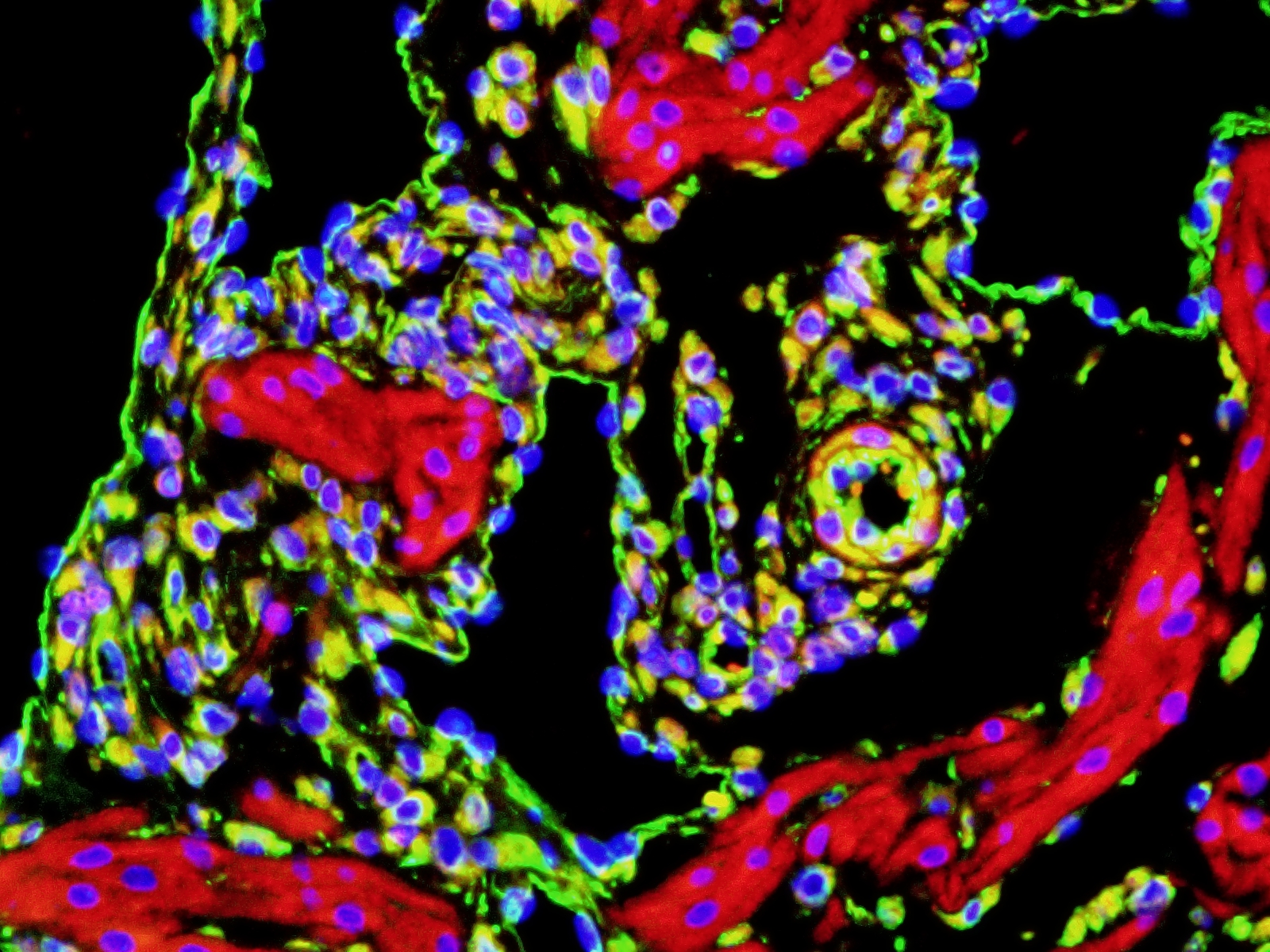

Supplement: Supplementary file 9 — Source data Fig. 7 [file 44318_2024_220_MOESM9_ESM.zip › Figure7/7A/CDKN2A-400-NK1.1 (5).jpg]

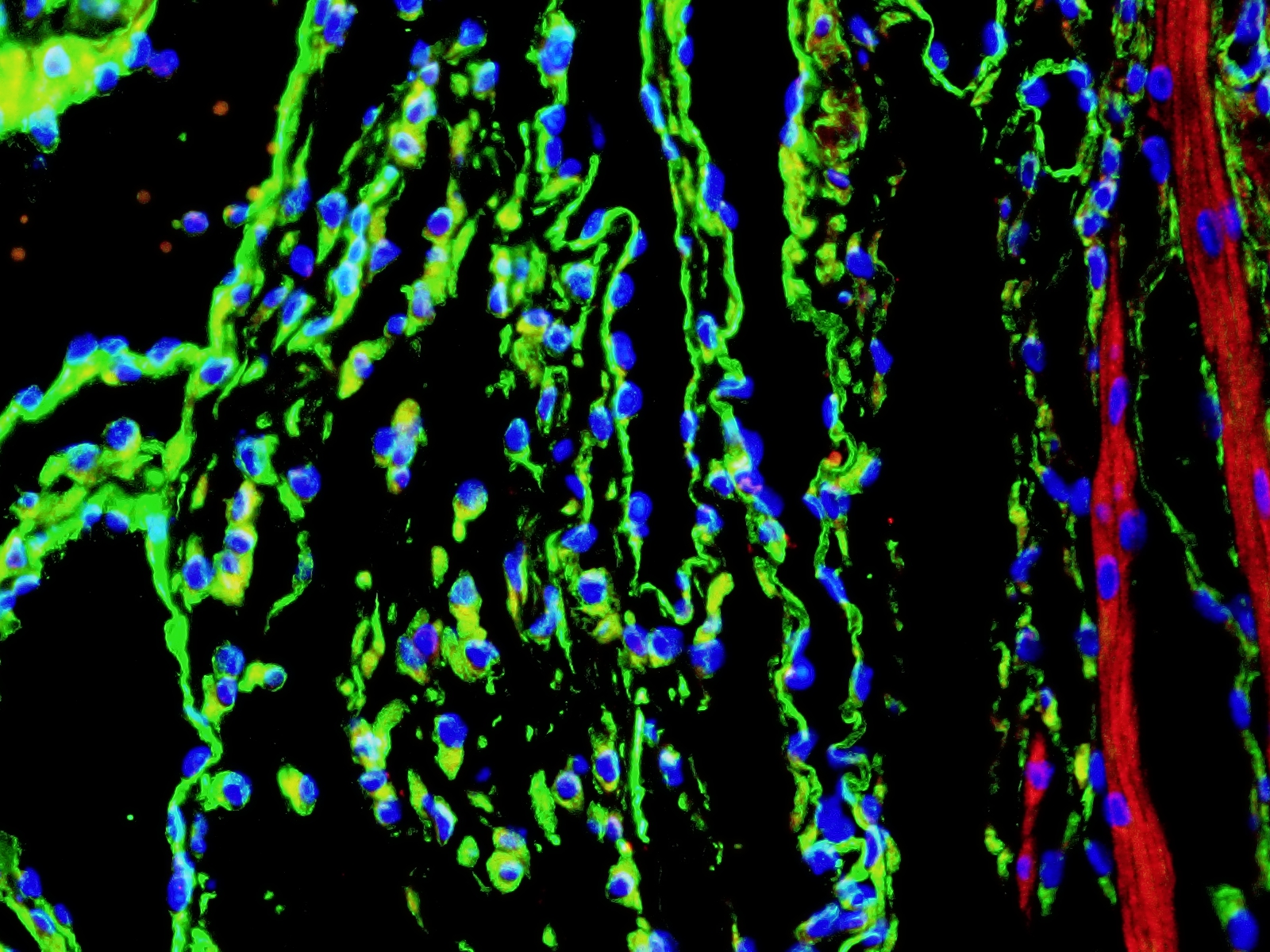

Supplement: Supplementary file 9 — Source data Fig. 7 [file 44318_2024_220_MOESM9_ESM.zip › Figure7/7A/CDKN2A-400-TNFSF14 (1).jpg]

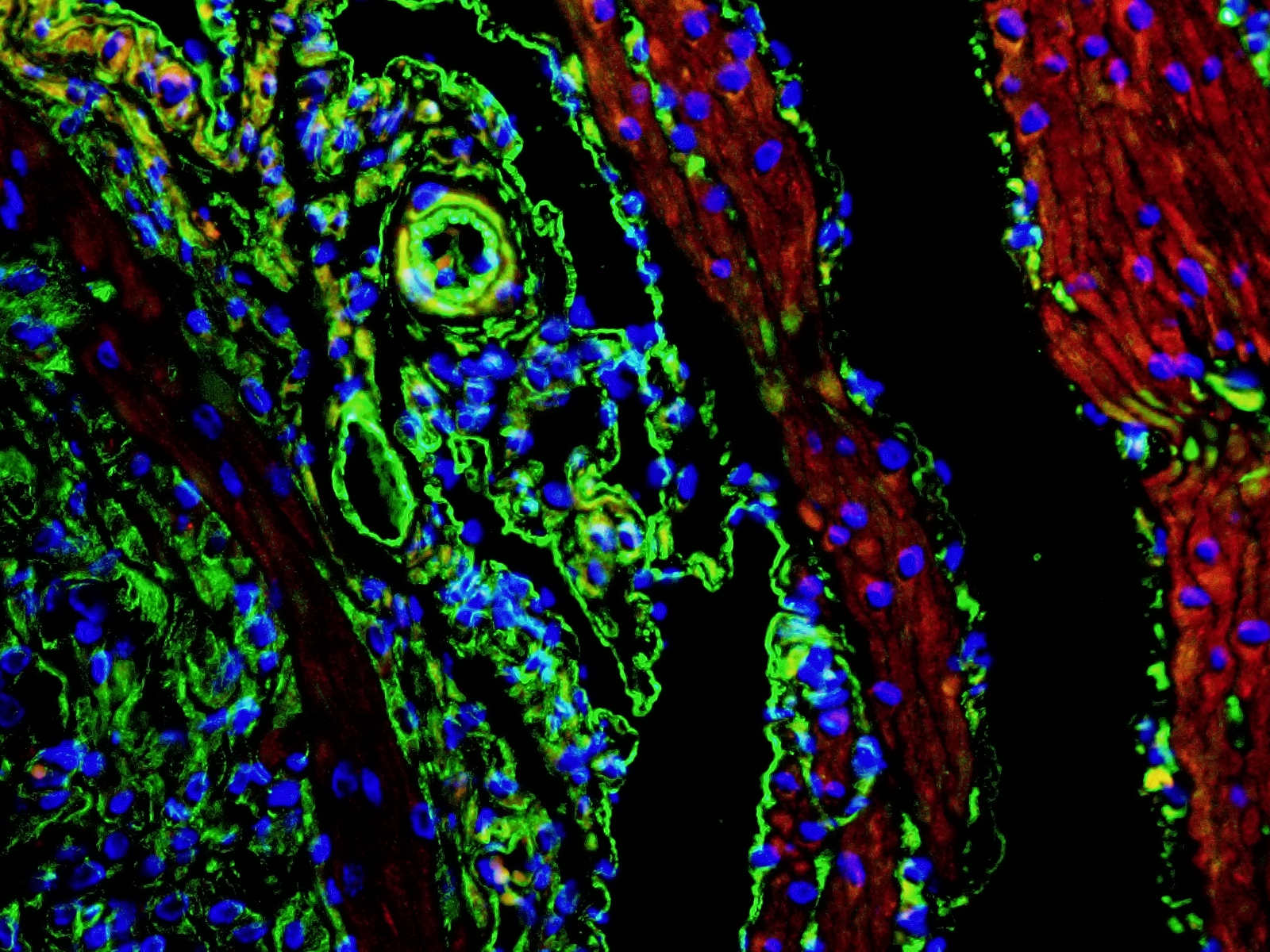

Supplement: Supplementary file 9 — Source data Fig. 7 [file 44318_2024_220_MOESM9_ESM.zip › Figure7/7A/CDKN2A-400-TNFSF14 (2).jpg]

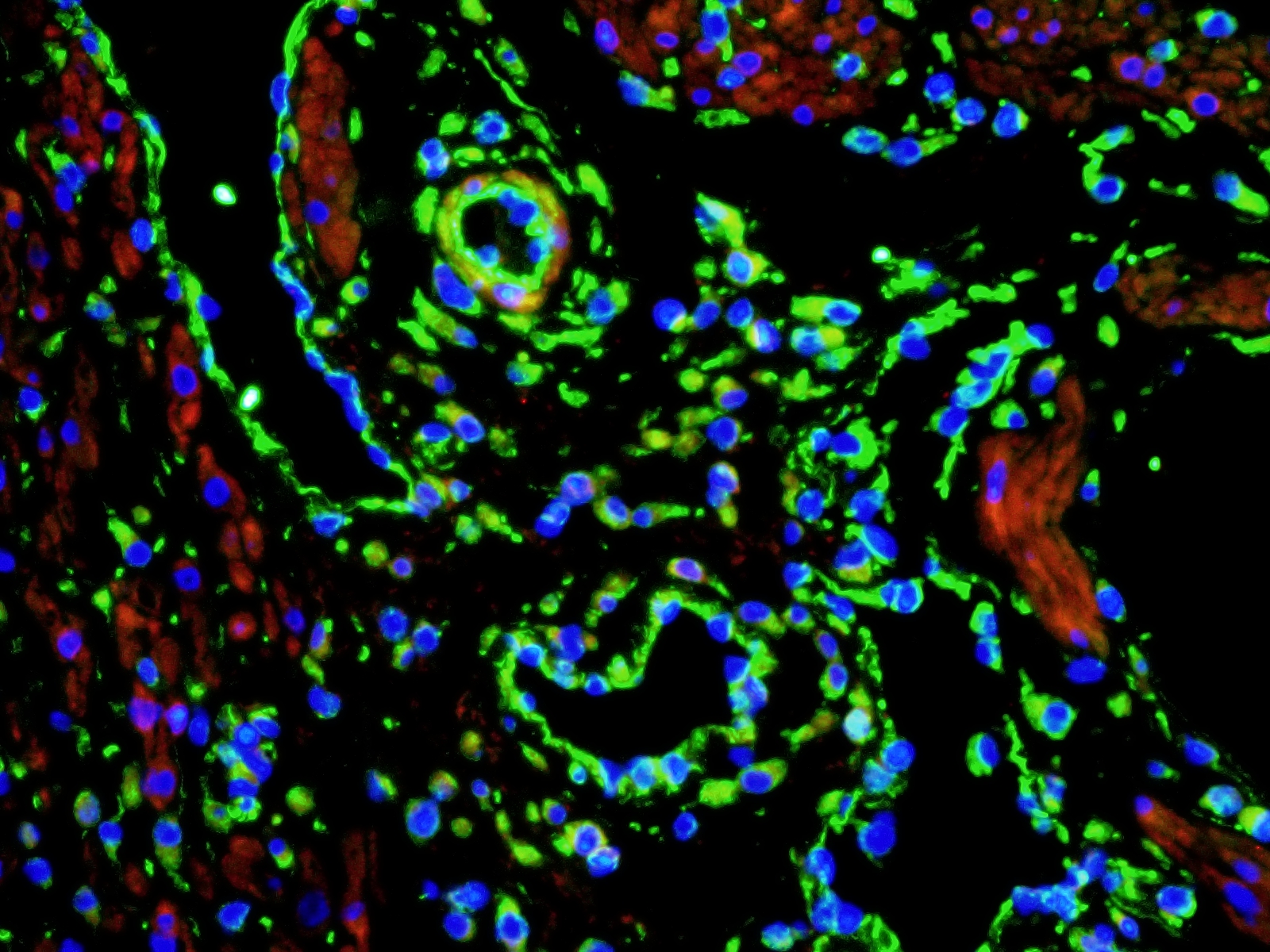

Supplement: Supplementary file 9 — Source data Fig. 7 [file 44318_2024_220_MOESM9_ESM.zip › Figure7/7A/CDKN2A-400-TNFSF14 (3).jpg]

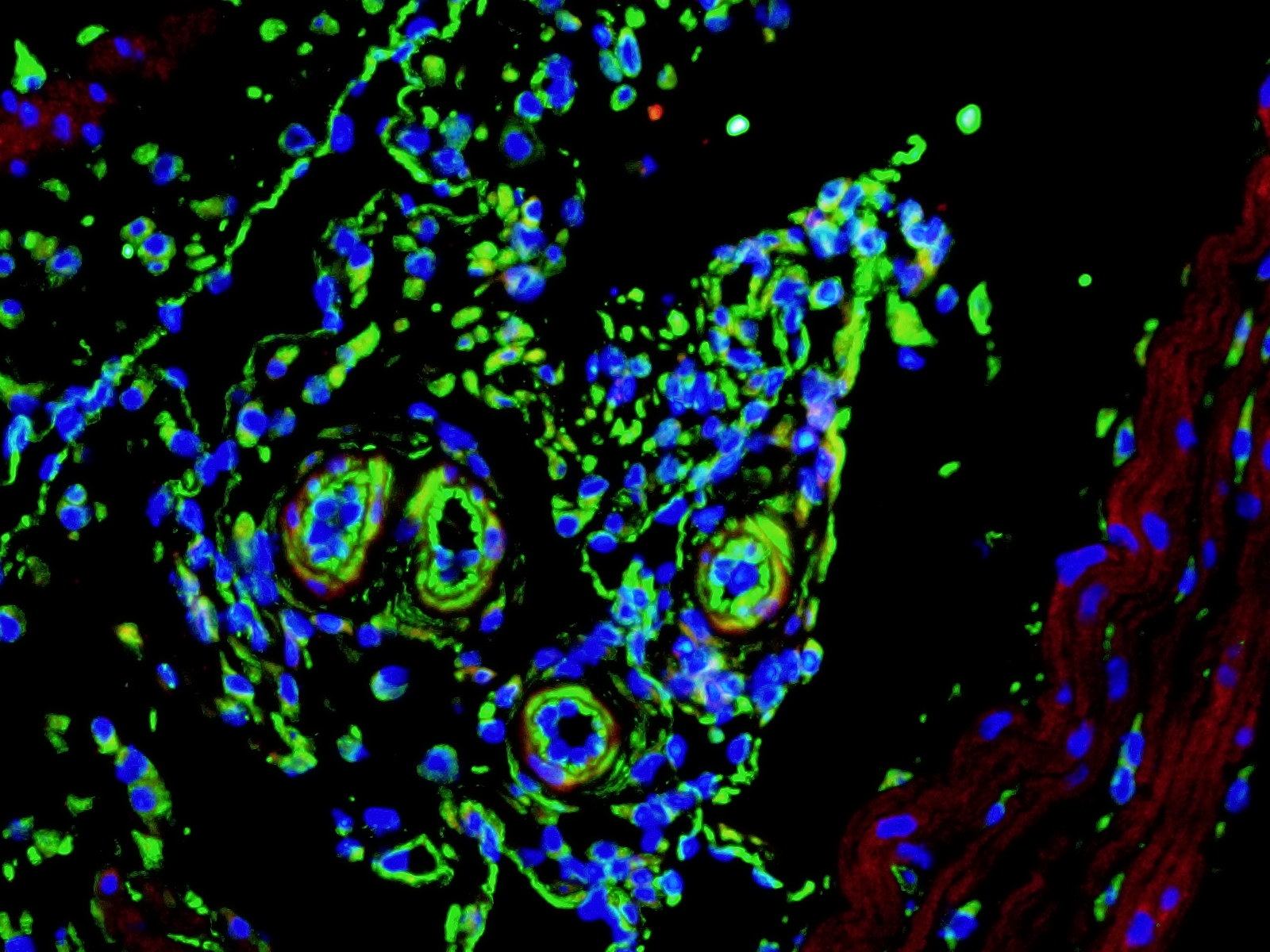

Supplement: Supplementary file 9 — Source data Fig. 7 [file 44318_2024_220_MOESM9_ESM.zip › Figure7/7A/CDKN2A-400-TNFSF14 (4).jpg]

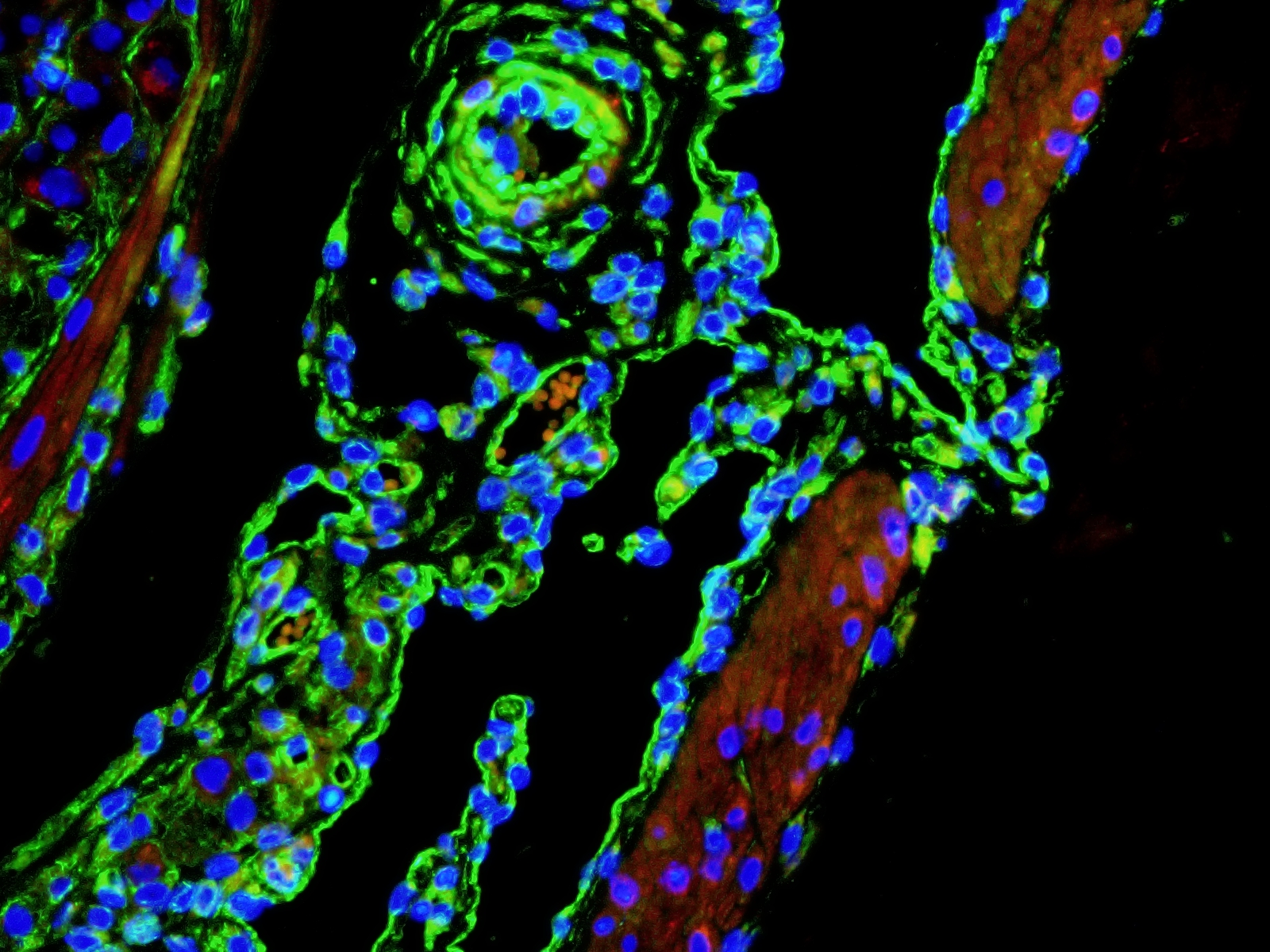

Supplement: Supplementary file 9 — Source data Fig. 7 [file 44318_2024_220_MOESM9_ESM.zip › Figure7/7A/CDKN2A-400-TNFSF14 (5).jpg]

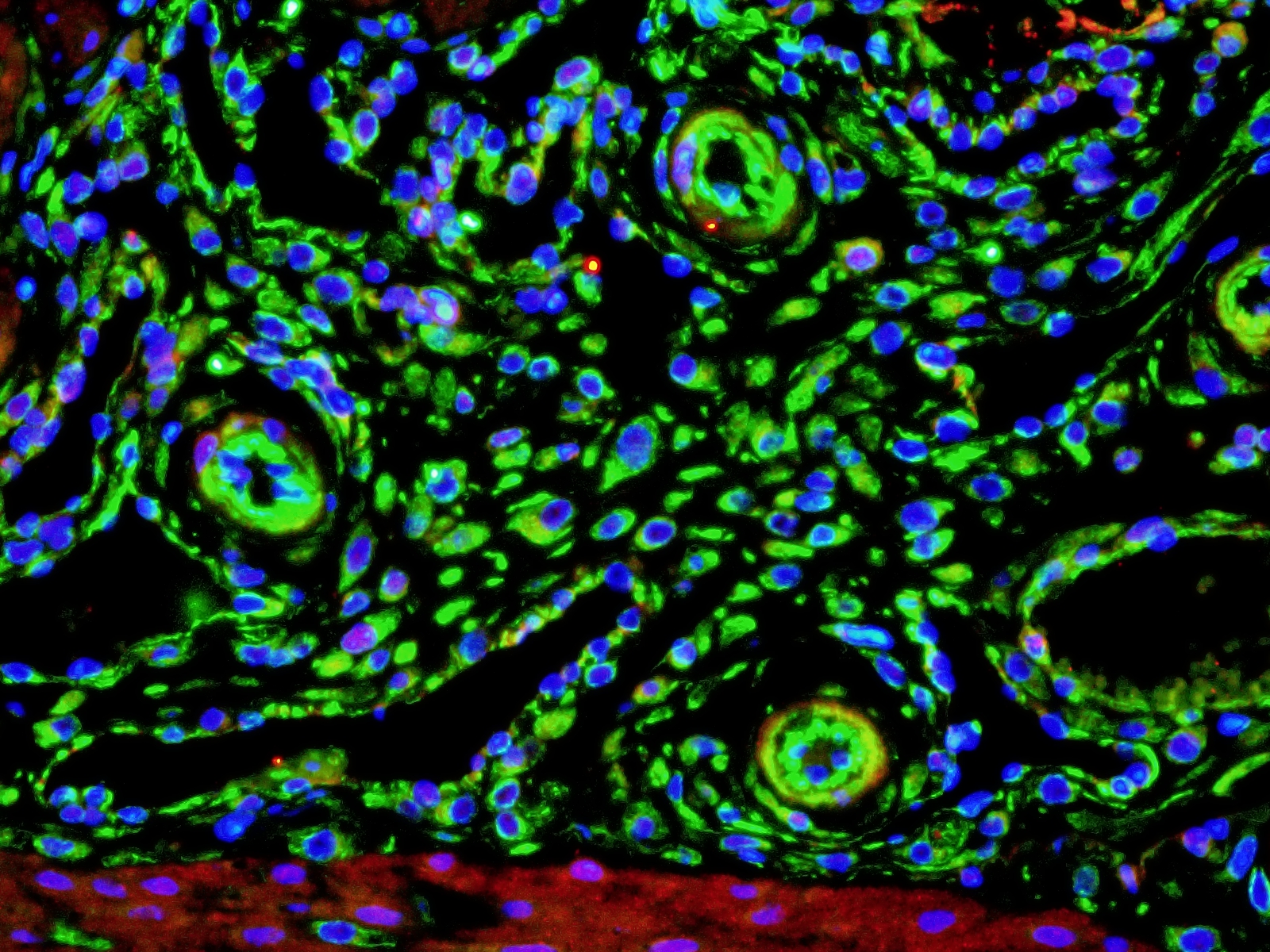

Supplement: Supplementary file 9 — Source data Fig. 7 [file 44318_2024_220_MOESM9_ESM.zip › Figure7/7A/TP53-400-Ctrl (1).jpg]

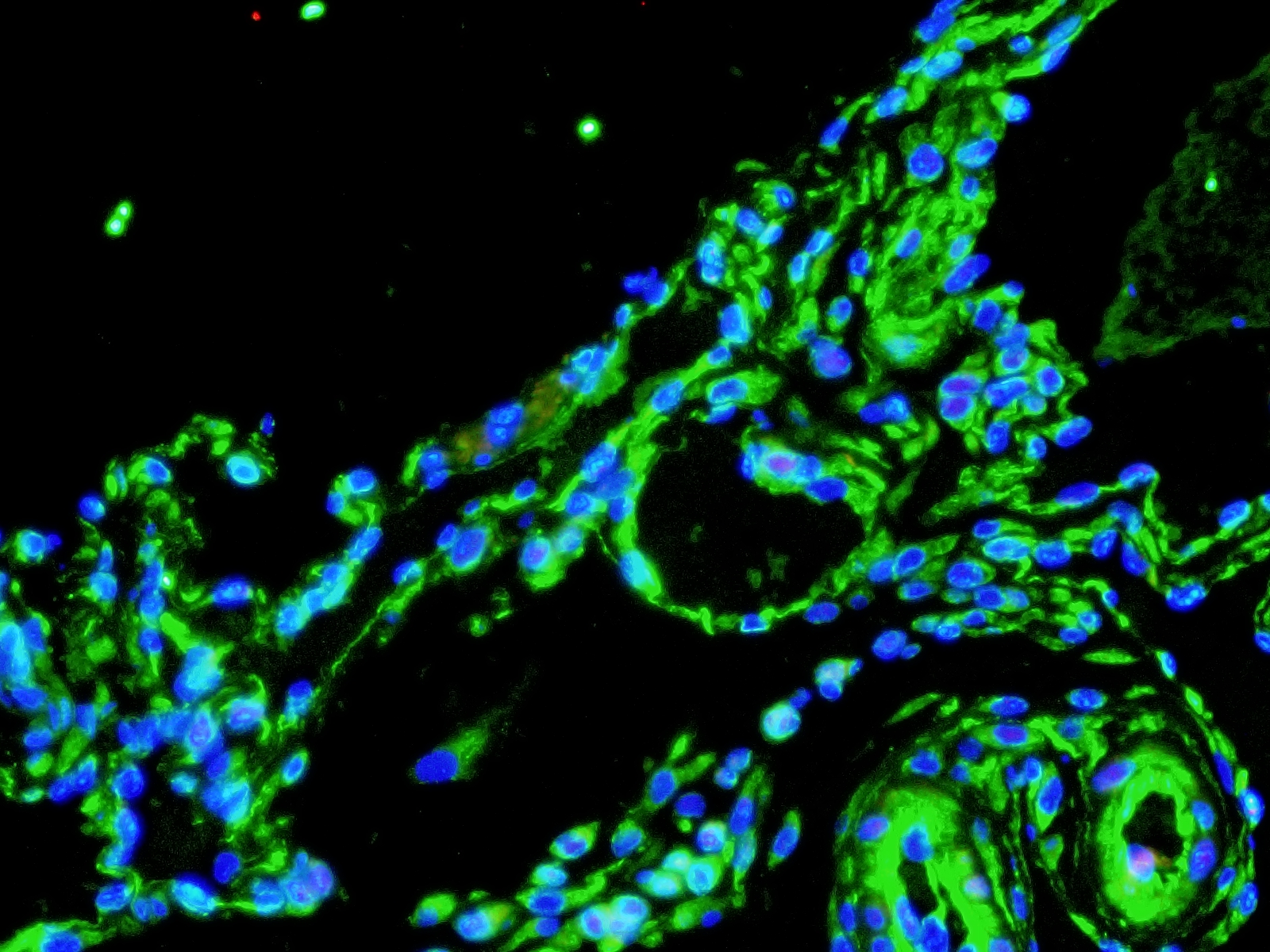

Supplement: Supplementary file 9 — Source data Fig. 7 [file 44318_2024_220_MOESM9_ESM.zip › Figure7/7A/TP53-400-Ctrl (2).jpg]

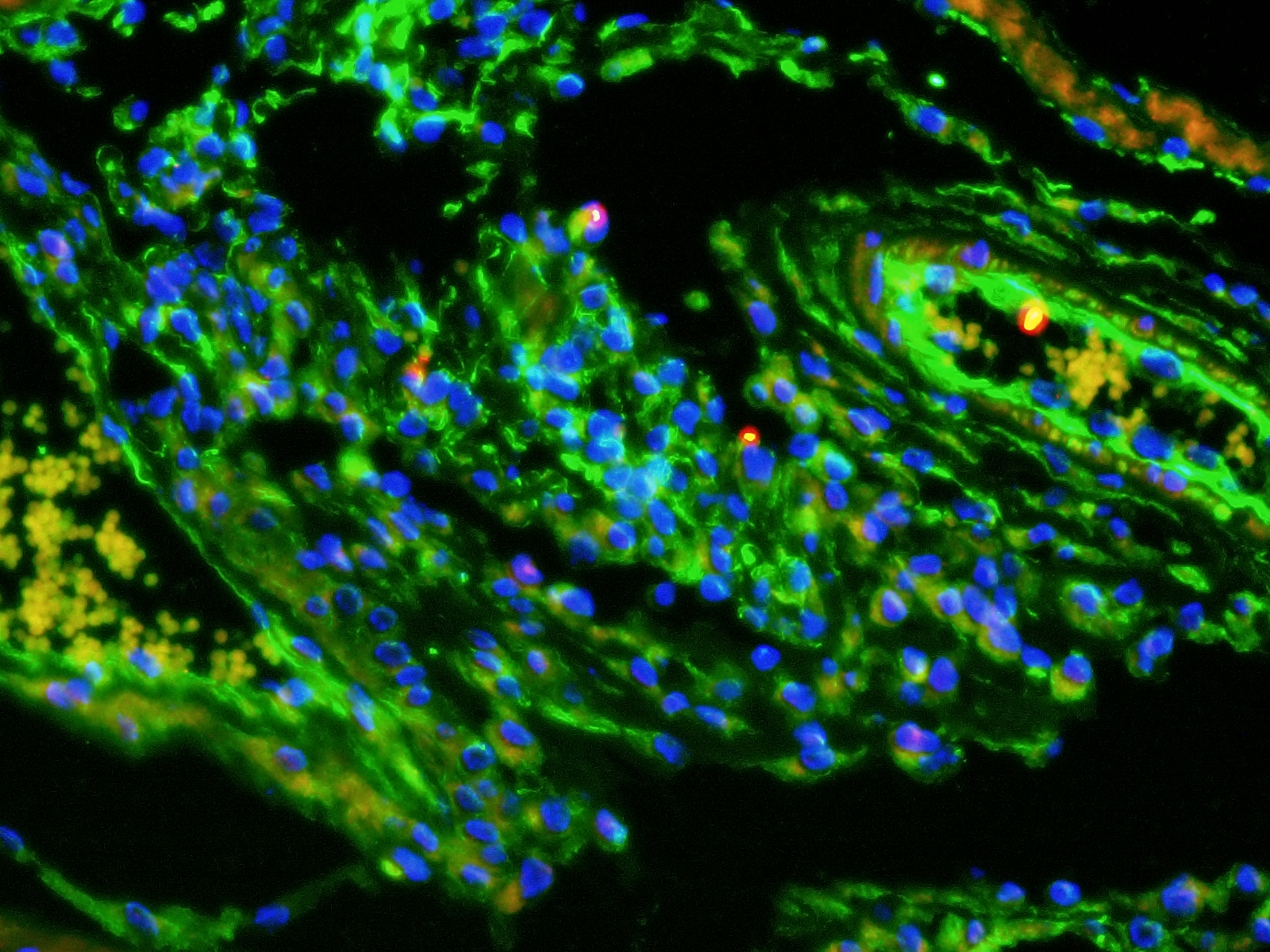

Supplement: Supplementary file 9 — Source data Fig. 7 [file 44318_2024_220_MOESM9_ESM.zip › Figure7/7A/TP53-400-Ctrl (3).jpg]

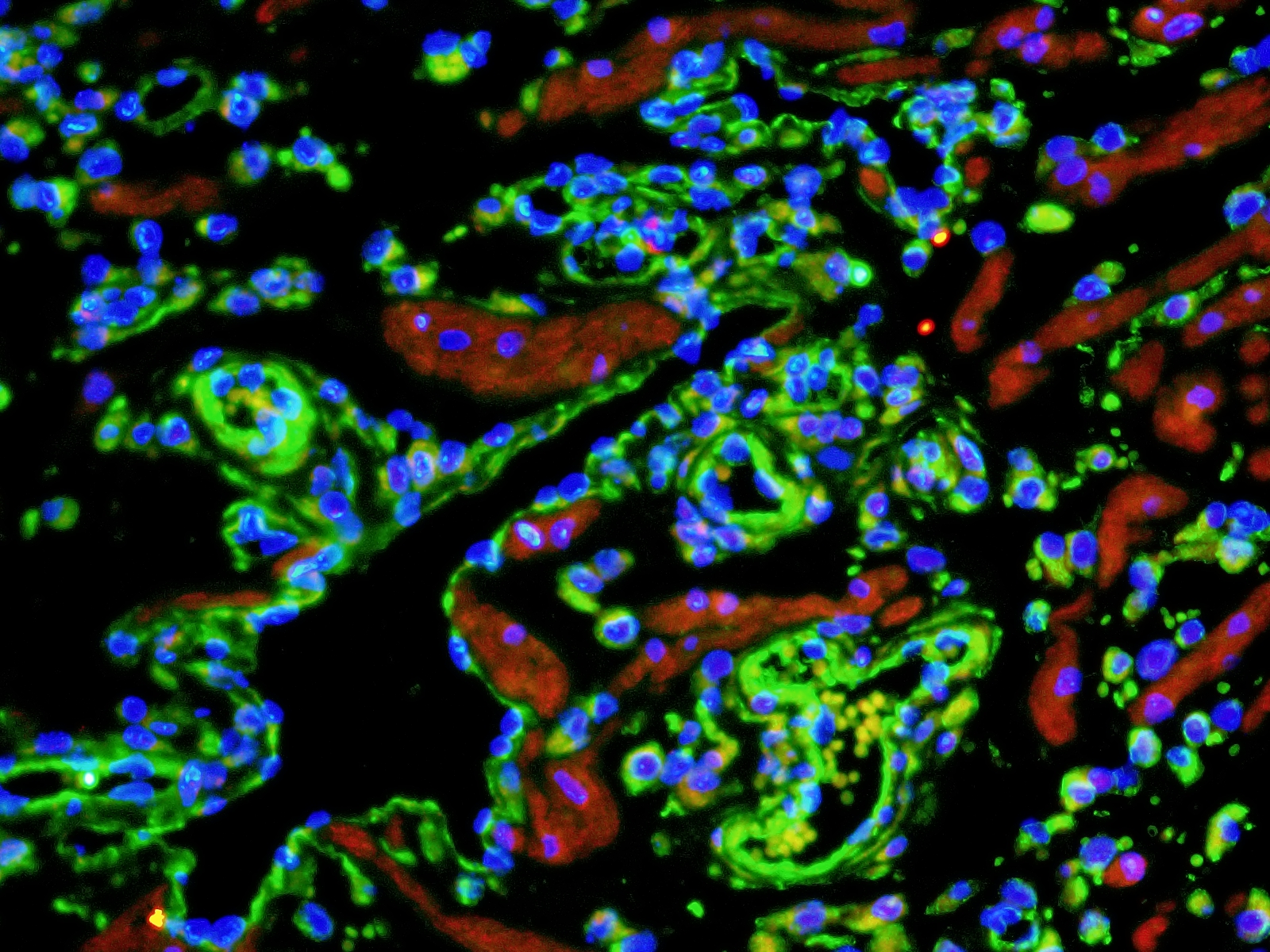

Supplement: Supplementary file 9 — Source data Fig. 7 [file 44318_2024_220_MOESM9_ESM.zip › Figure7/7A/TP53-400-Ctrl (4).jpg]

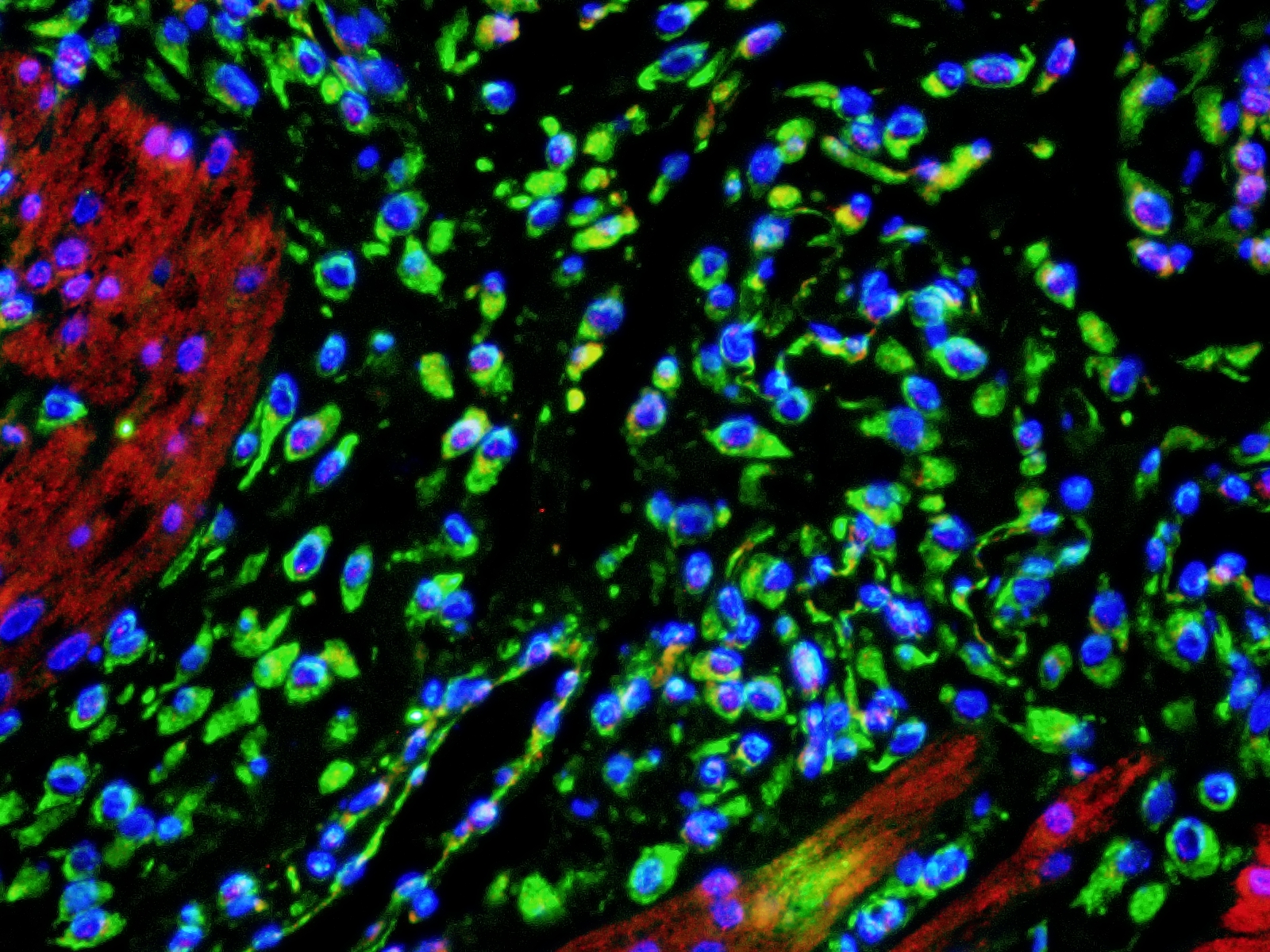

Supplement: Supplementary file 9 — Source data Fig. 7 [file 44318_2024_220_MOESM9_ESM.zip › Figure7/7A/TP53-400-Ctrl (5).jpg]

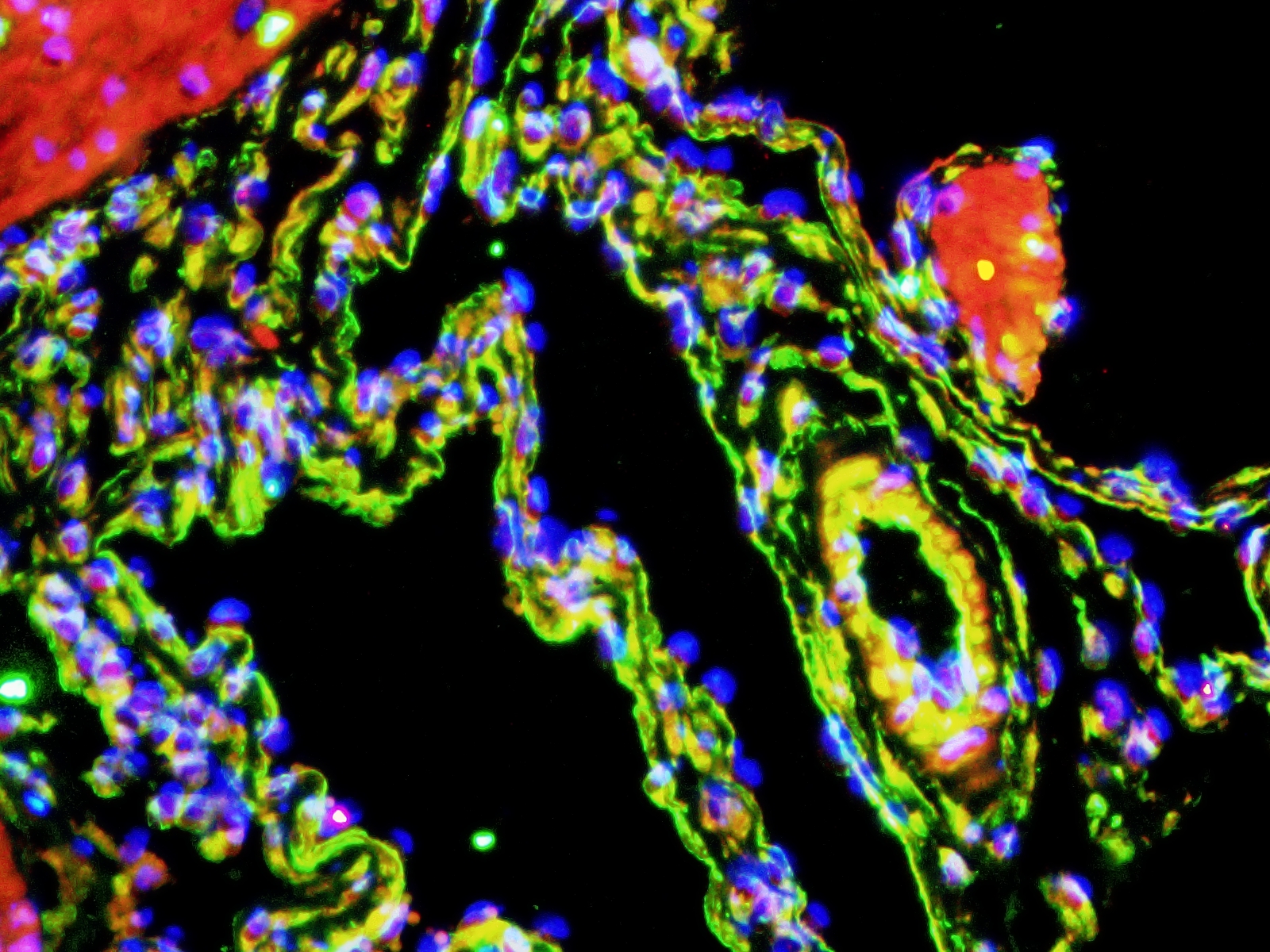

Supplement: Supplementary file 9 — Source data Fig. 7 [file 44318_2024_220_MOESM9_ESM.zip › Figure7/7A/TP53-400-NK1 (1).jpg]

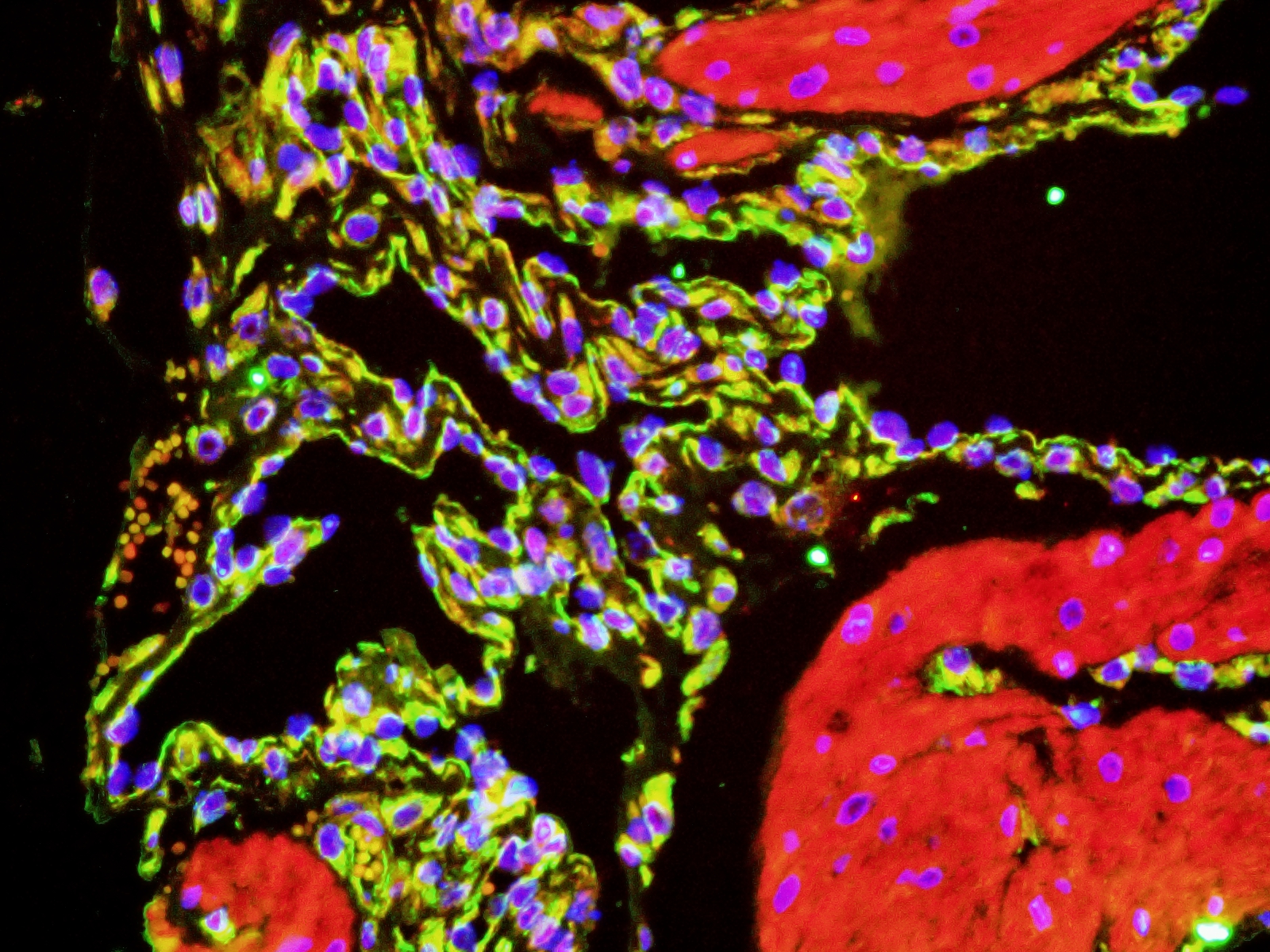

Supplement: Supplementary file 9 — Source data Fig. 7 [file 44318_2024_220_MOESM9_ESM.zip › Figure7/7A/TP53-400-NK1 (2).jpg]

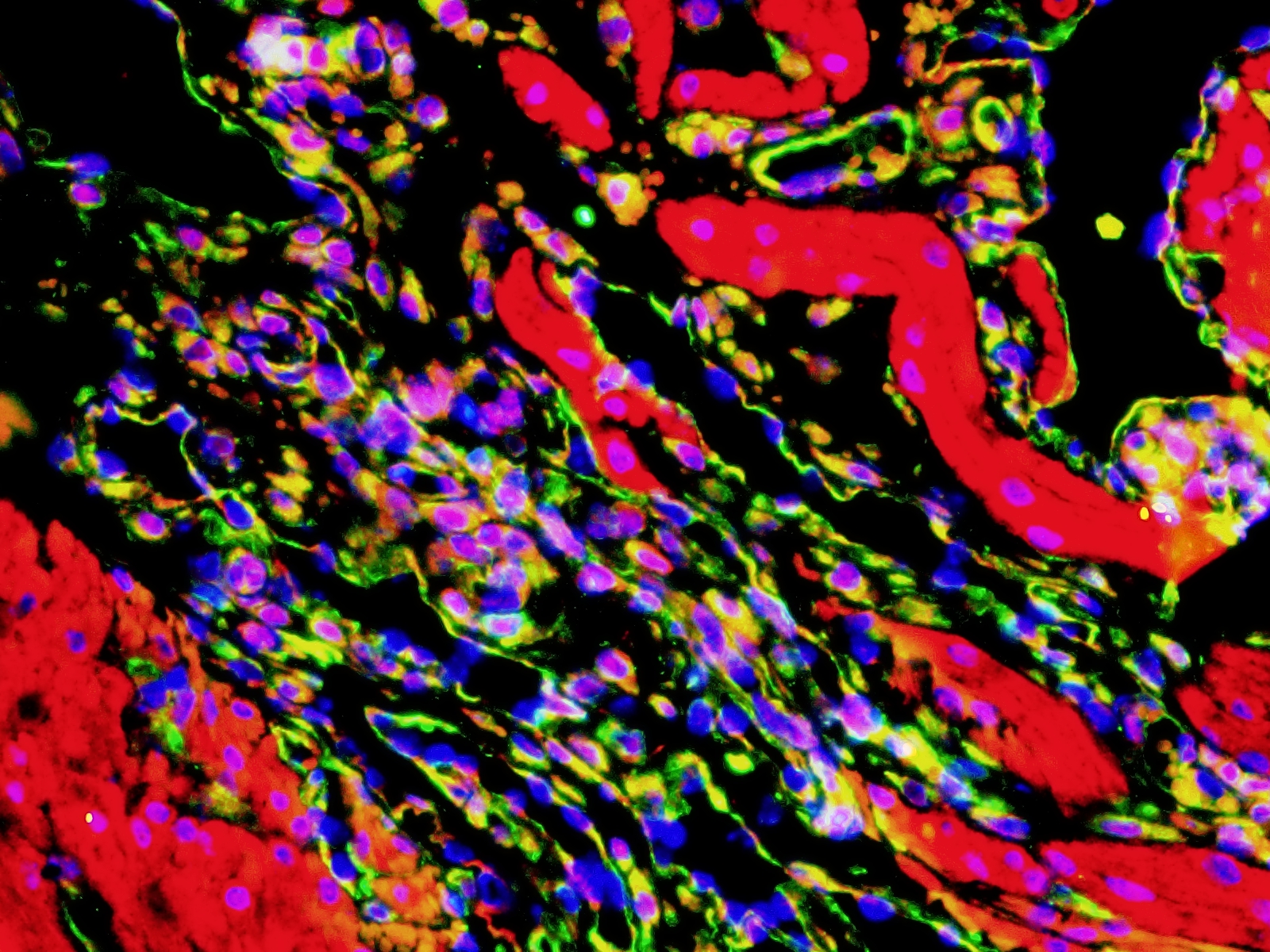

Supplement: Supplementary file 9 — Source data Fig. 7 [file 44318_2024_220_MOESM9_ESM.zip › Figure7/7A/TP53-400-NK1 (3).jpg]

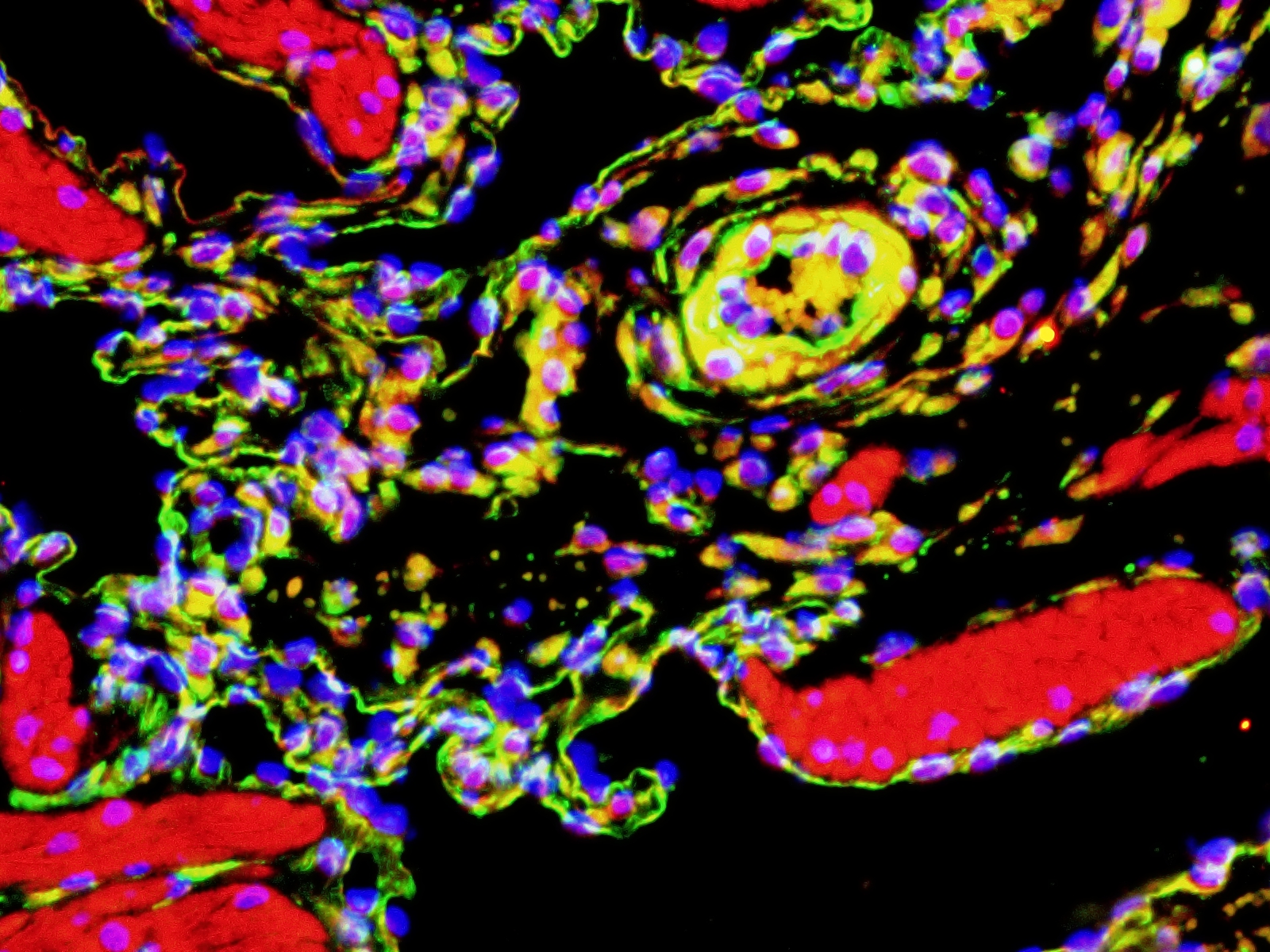

Supplement: Supplementary file 9 — Source data Fig. 7 [file 44318_2024_220_MOESM9_ESM.zip › Figure7/7A/TP53-400-NK1 (4).jpg]

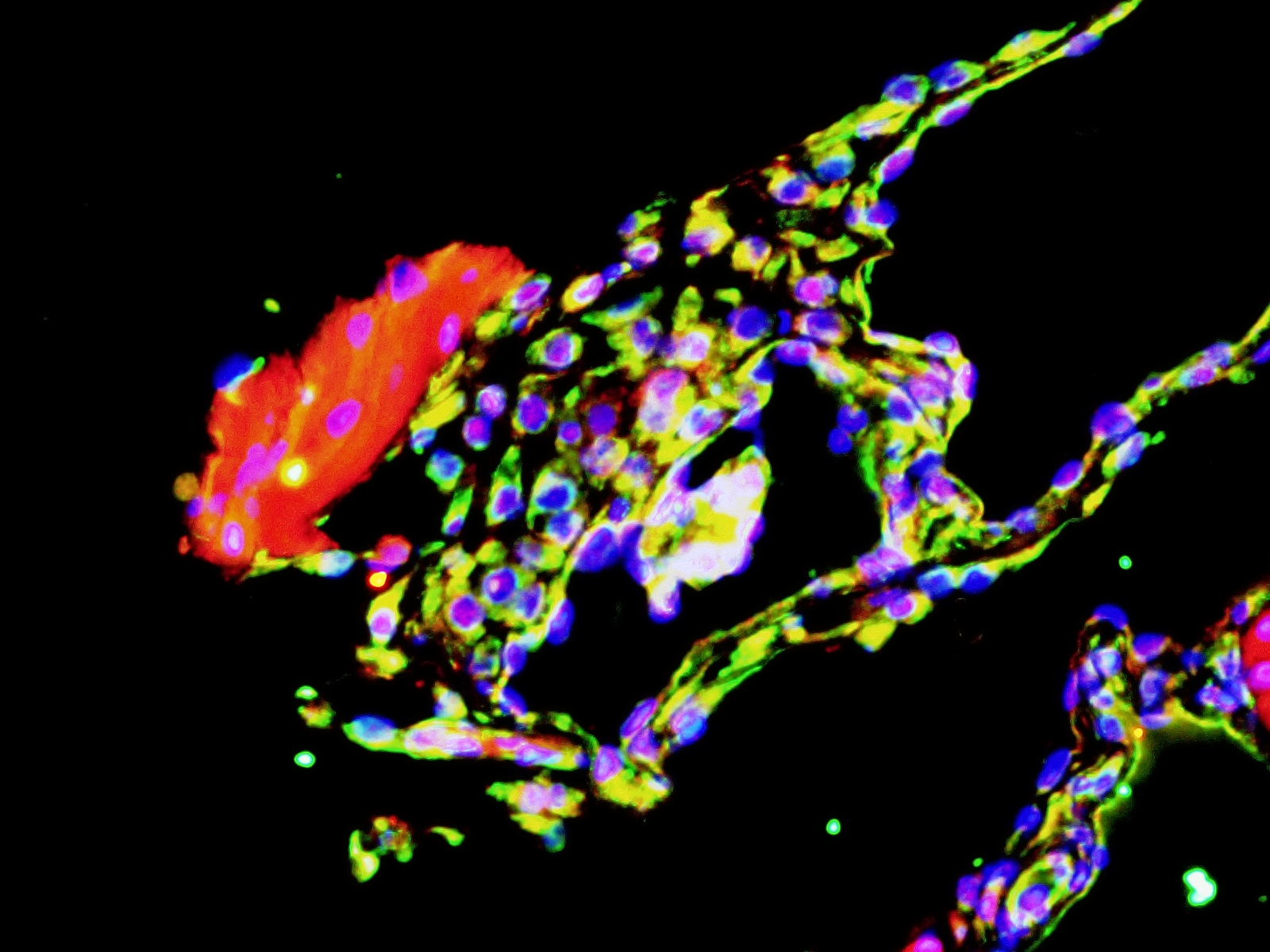

Supplement: Supplementary file 9 — Source data Fig. 7 [file 44318_2024_220_MOESM9_ESM.zip › Figure7/7A/TP53-400-NK1 (5).jpg]

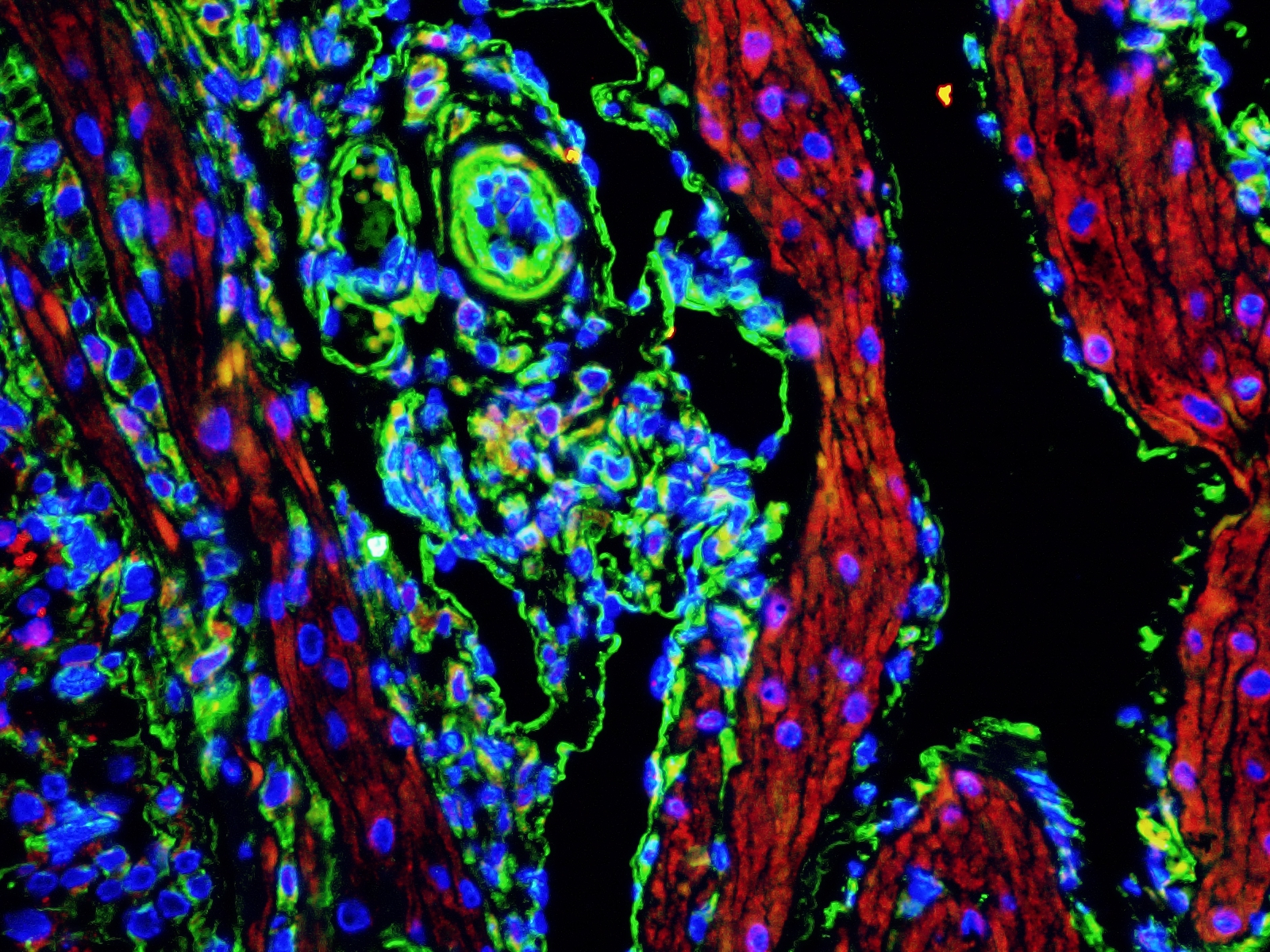

Supplement: Supplementary file 9 — Source data Fig. 7 [file 44318_2024_220_MOESM9_ESM.zip › Figure7/7A/TP53-400-TNFSF14 (1).jpg]

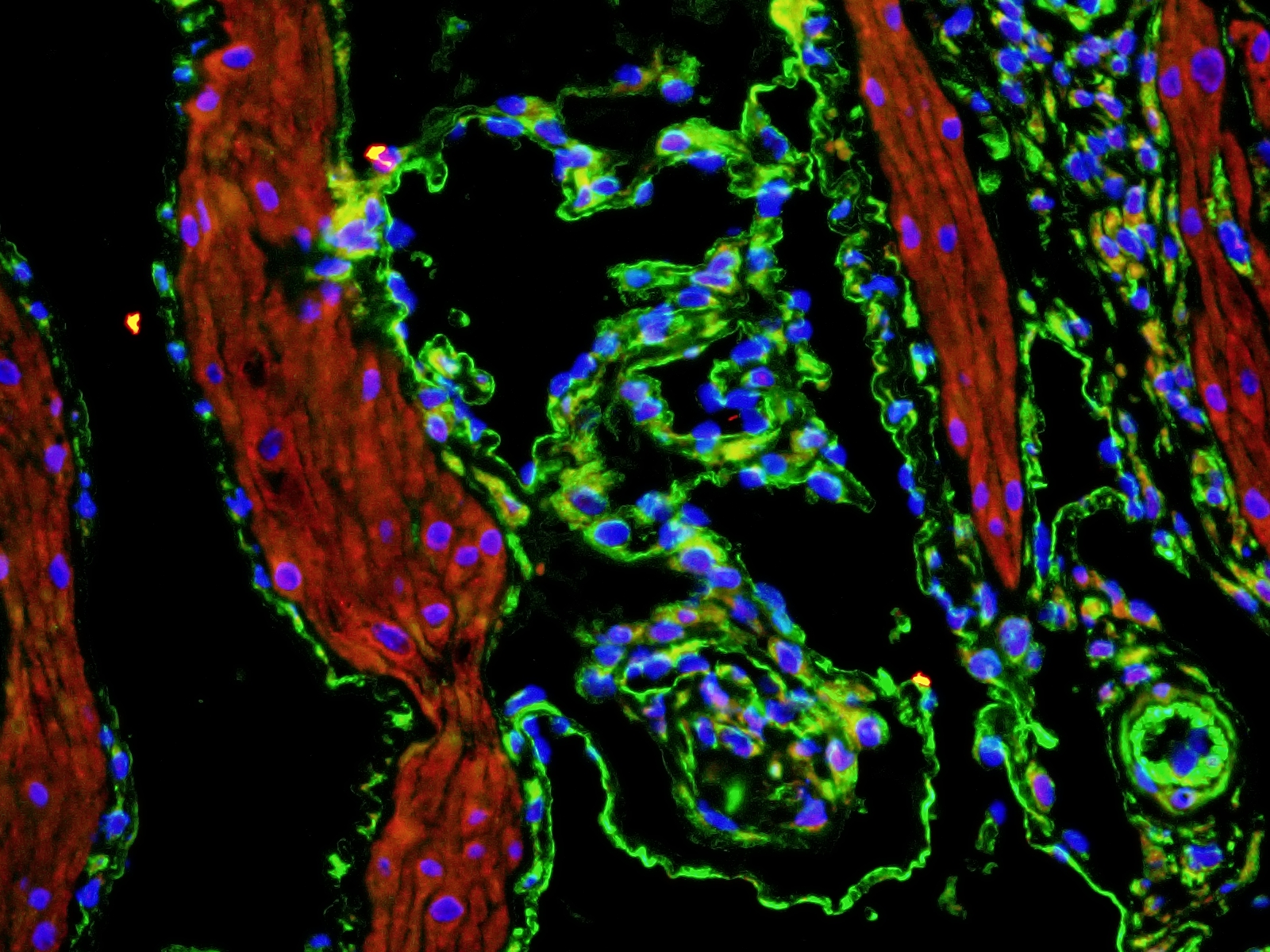

Supplement: Supplementary file 9 — Source data Fig. 7 [file 44318_2024_220_MOESM9_ESM.zip › Figure7/7A/TP53-400-TNFSF14 (2).jpg]

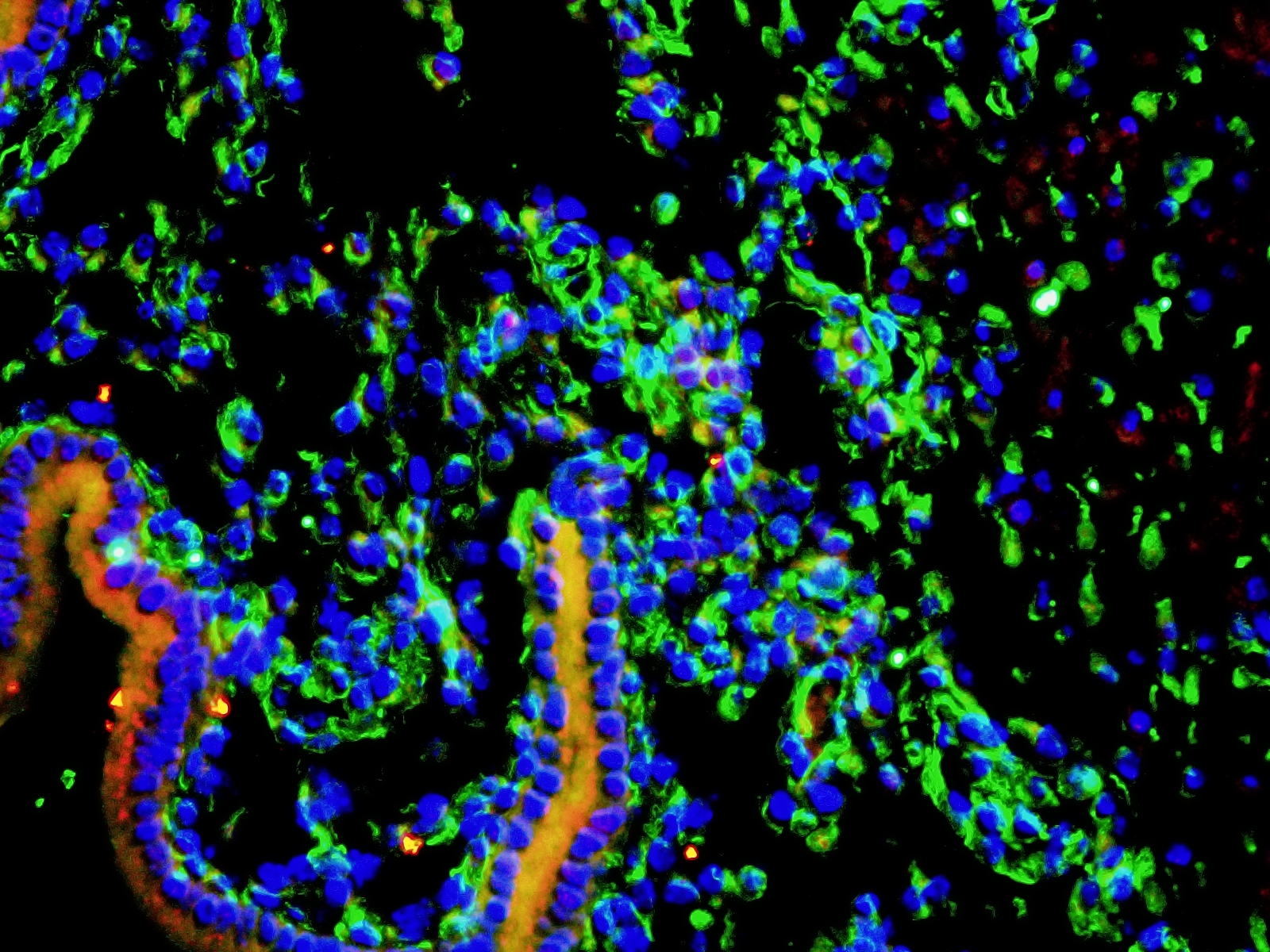

Supplement: Supplementary file 9 — Source data Fig. 7 [file 44318_2024_220_MOESM9_ESM.zip › Figure7/7A/TP53-400-TNFSF14 (3).jpg]

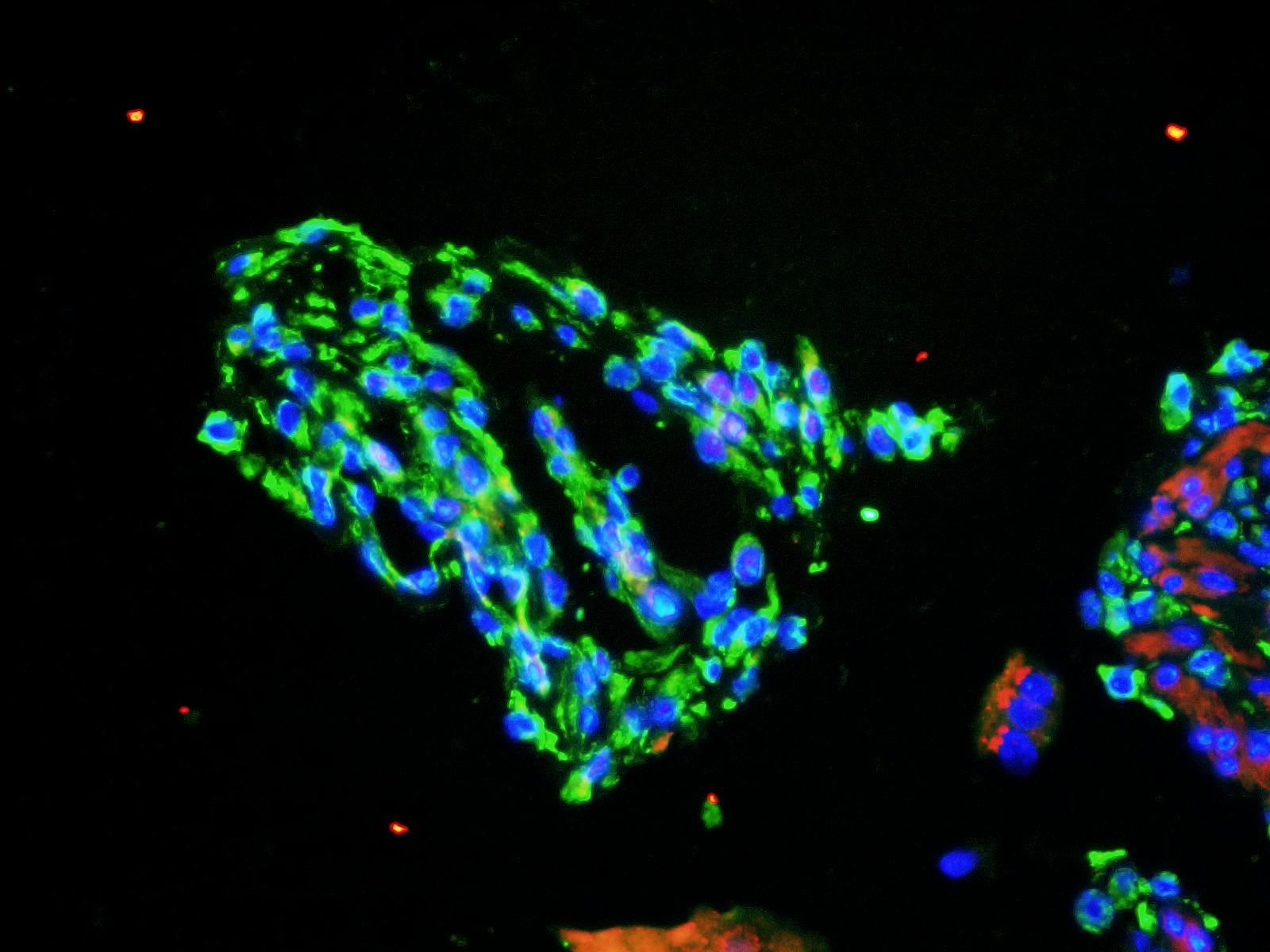

Supplement: Supplementary file 9 — Source data Fig. 7 [file 44318_2024_220_MOESM9_ESM.zip › Figure7/7A/TP53-400-TNFSF14 (4).jpg]

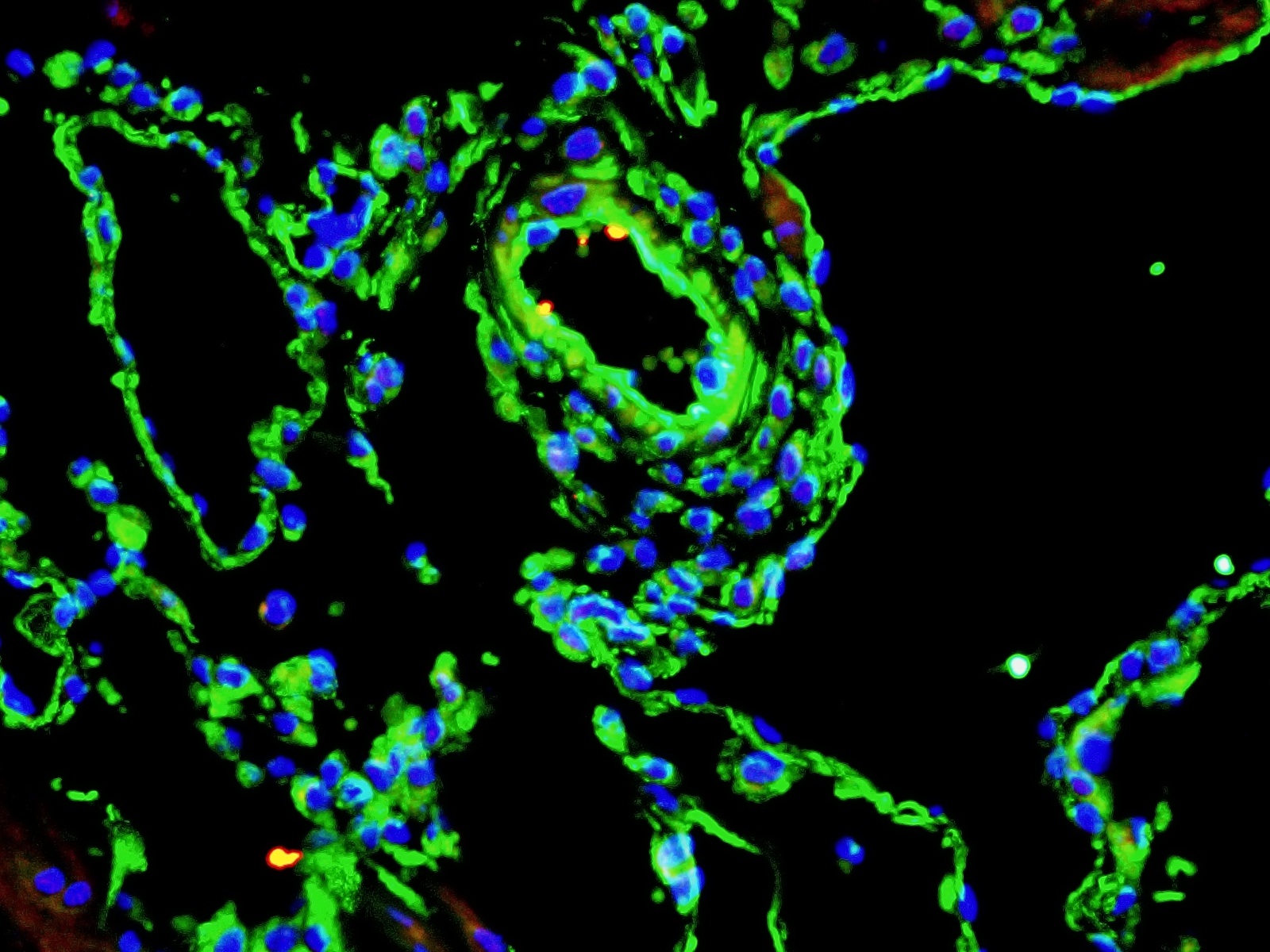

Supplement: Supplementary file 9 — Source data Fig. 7 [file 44318_2024_220_MOESM9_ESM.zip › Figure7/7A/TP53-400-TNFSF14 (5).jpg]

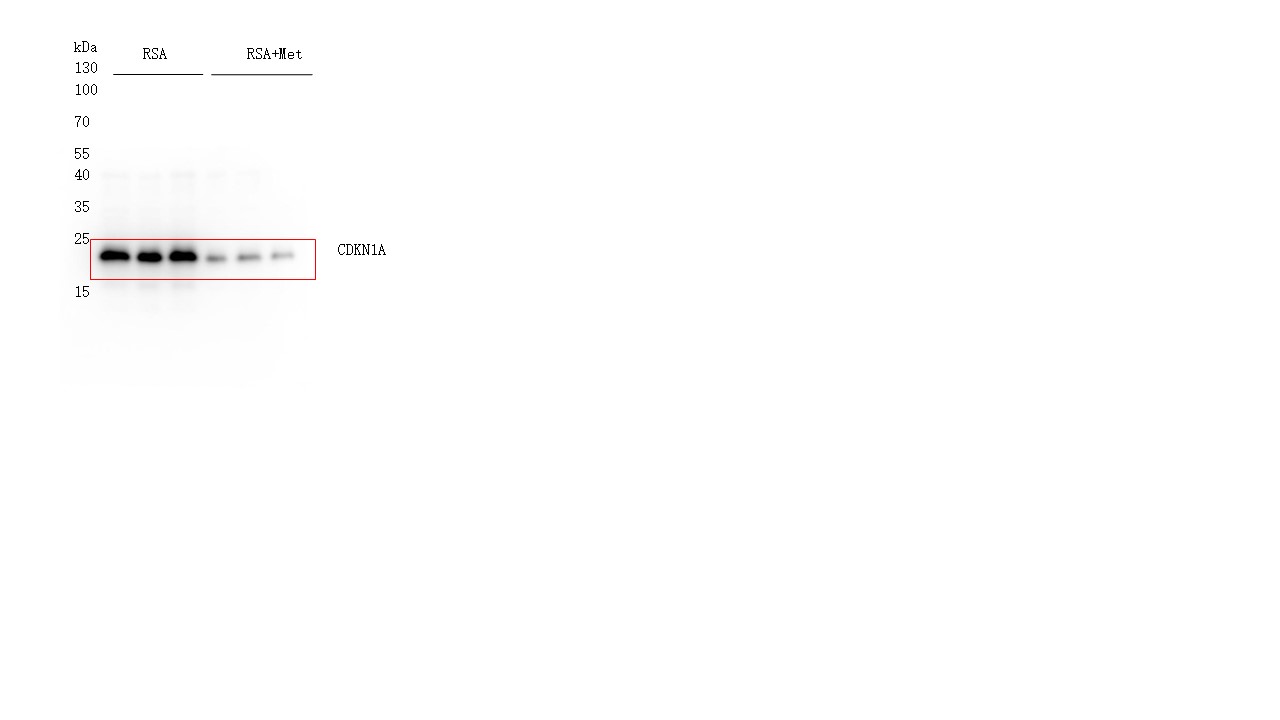

Supplement: Supplementary file 10 — Source data Fig. 8 [file 44318_2024_220_MOESM10_ESM.zip › Figure8/8A/western CDKN1A.jpg]

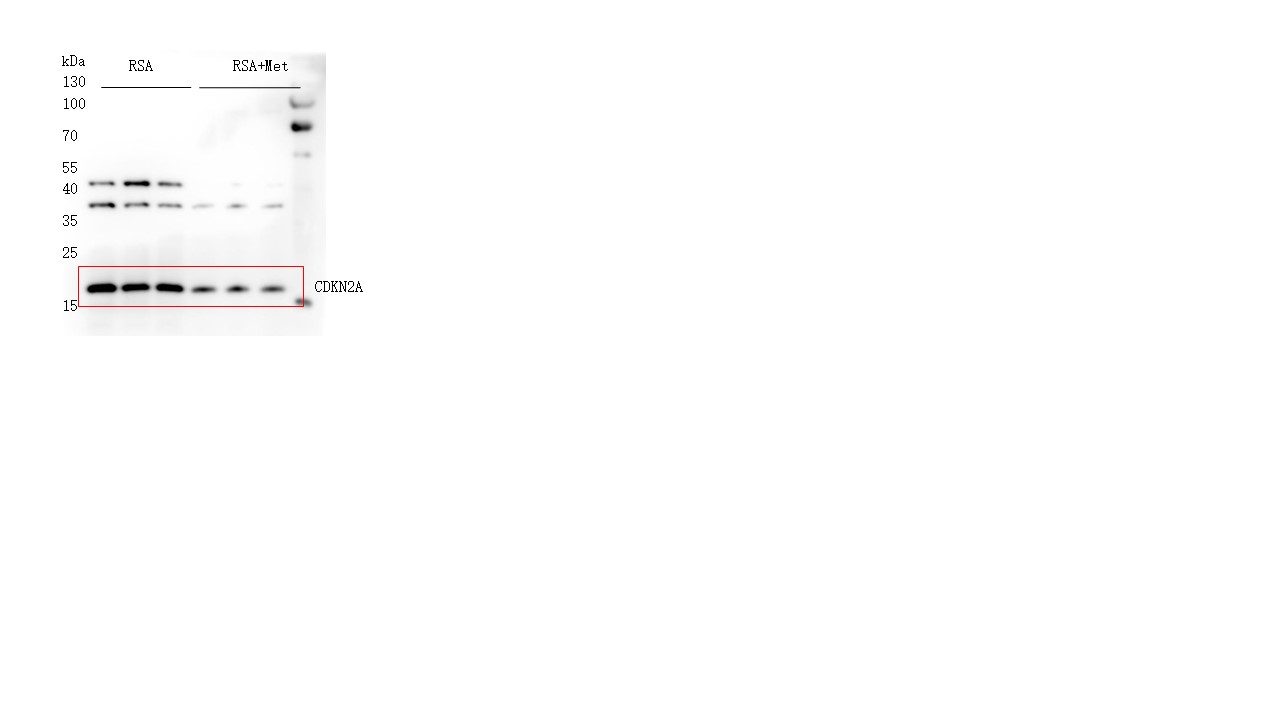

Supplement: Supplementary file 10 — Source data Fig. 8 [file 44318_2024_220_MOESM10_ESM.zip › Figure8/8A/western CDKN2A.jpg]

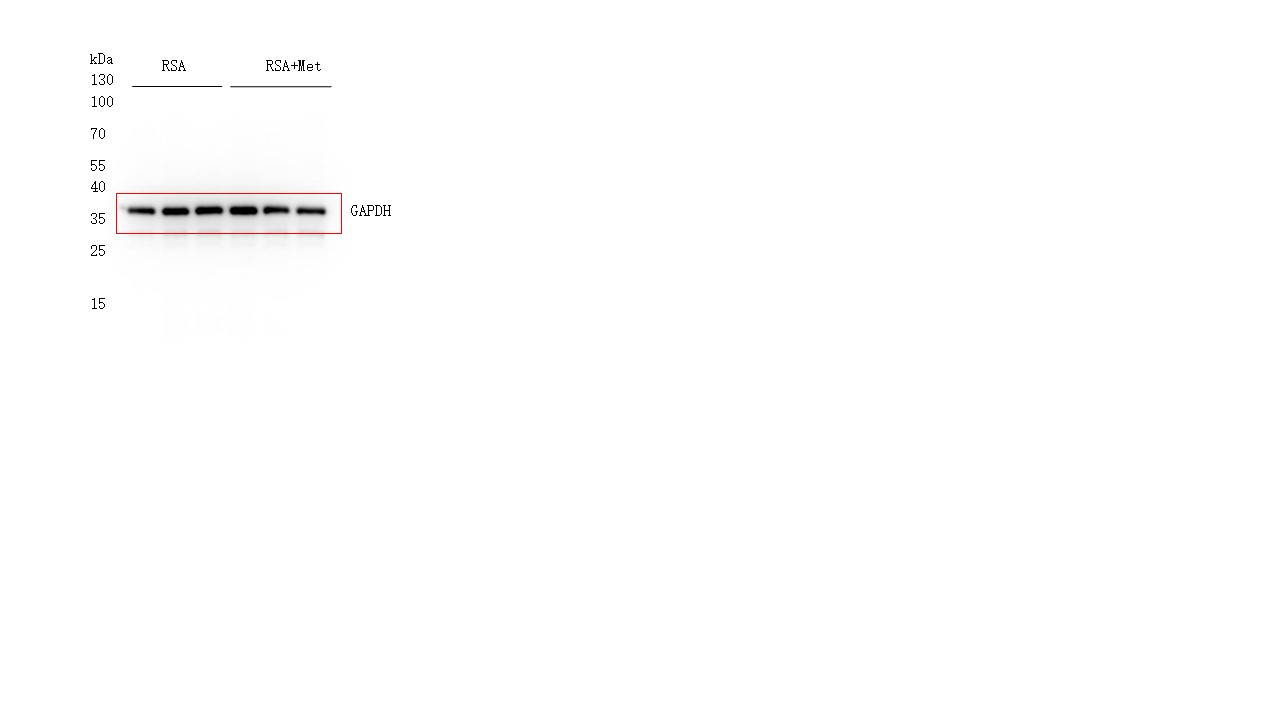

Supplement: Supplementary file 10 — Source data Fig. 8 [file 44318_2024_220_MOESM10_ESM.zip › Figure8/8A/western GAPDH.jpg]

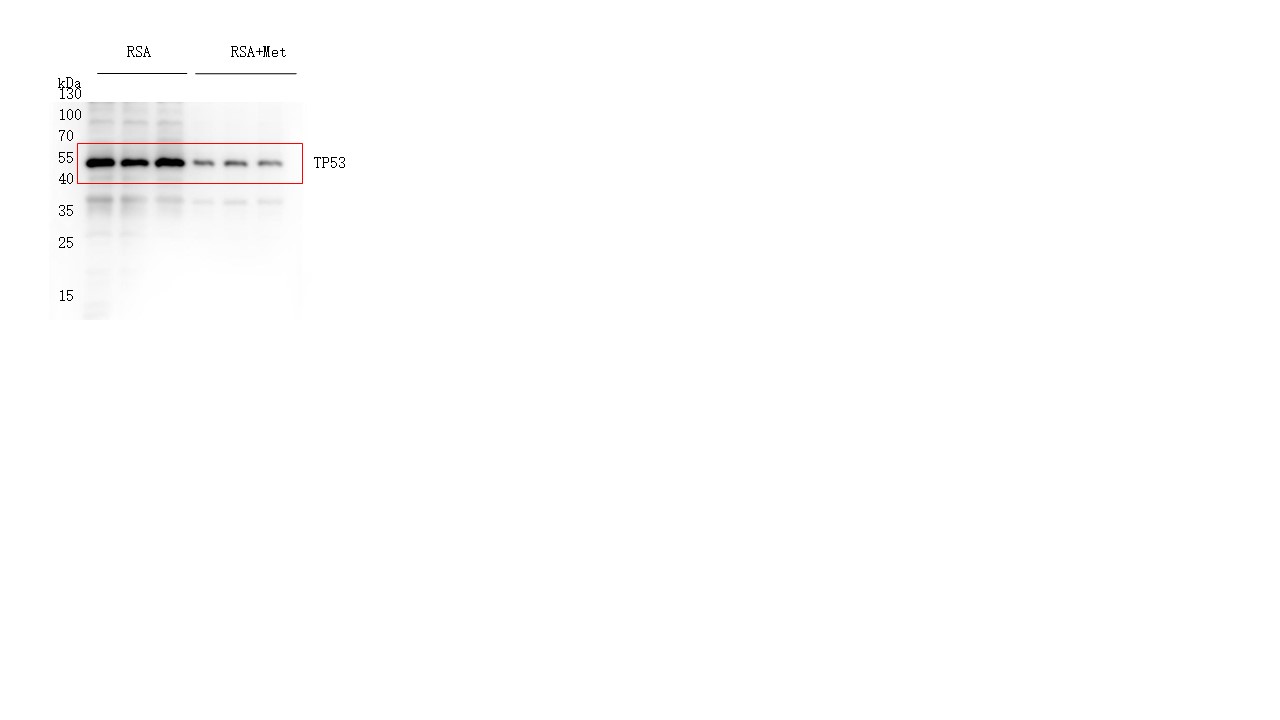

Supplement: Supplementary file 10 — Source data Fig. 8 [file 44318_2024_220_MOESM10_ESM.zip › Figure8/8A/western TP53.jpg]

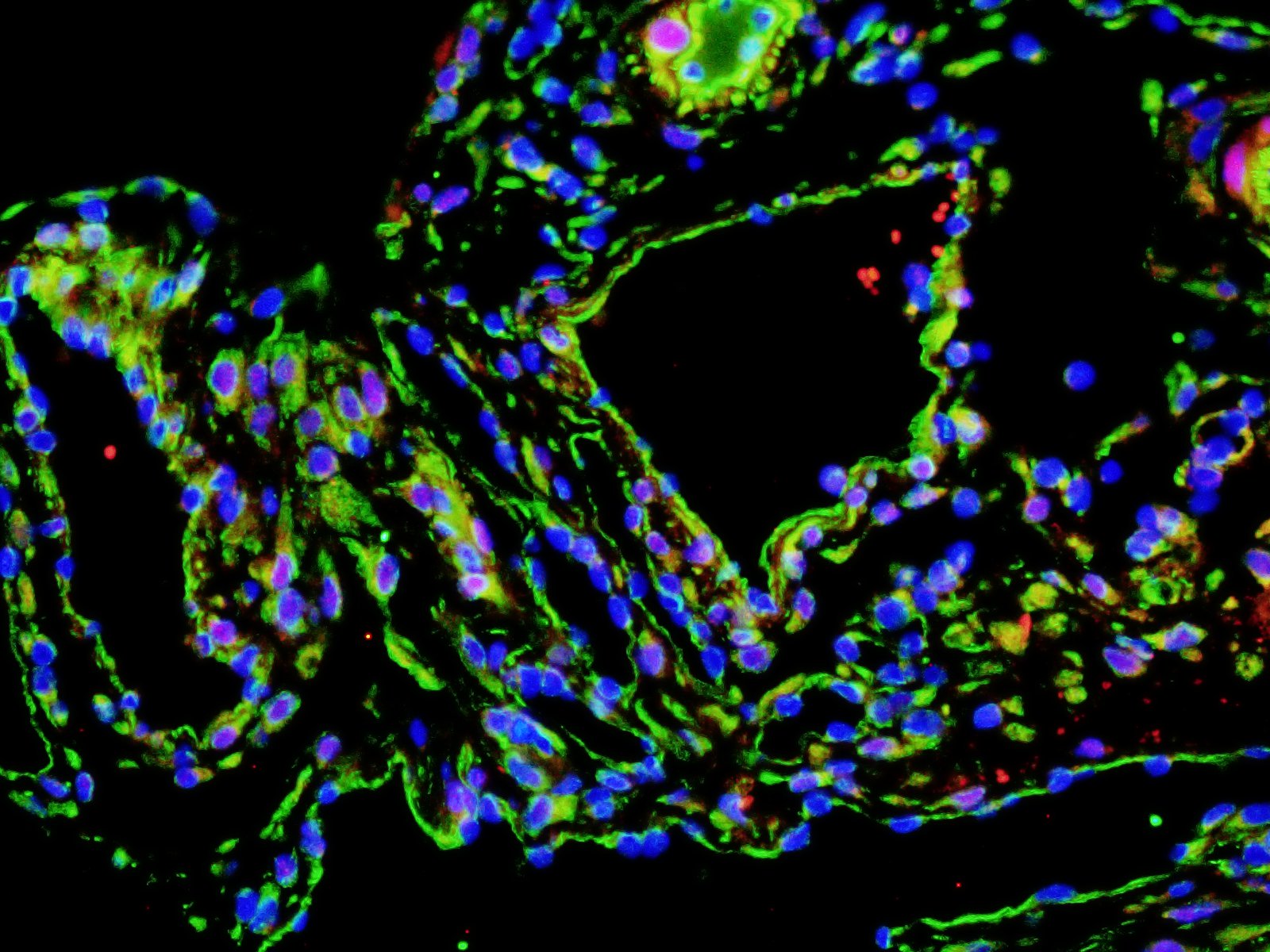

Supplement: Supplementary file 10 — Source data Fig. 8 [file 44318_2024_220_MOESM10_ESM.zip › Figure8/8G/CDKN1A-400-Met (1).jpg]

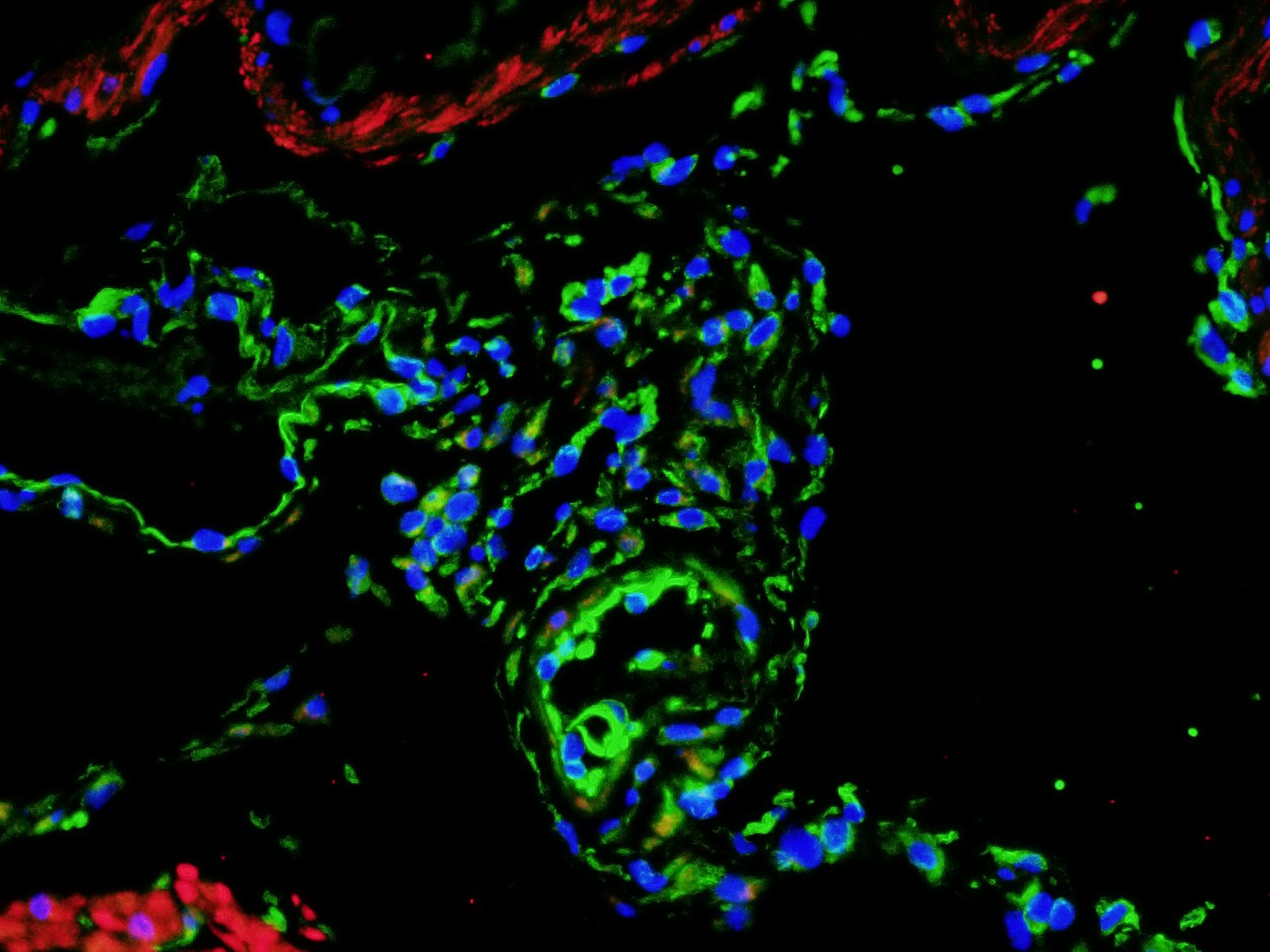

Supplement: Supplementary file 10 — Source data Fig. 8 [file 44318_2024_220_MOESM10_ESM.zip › Figure8/8G/CDKN1A-400-Met (2).jpg]

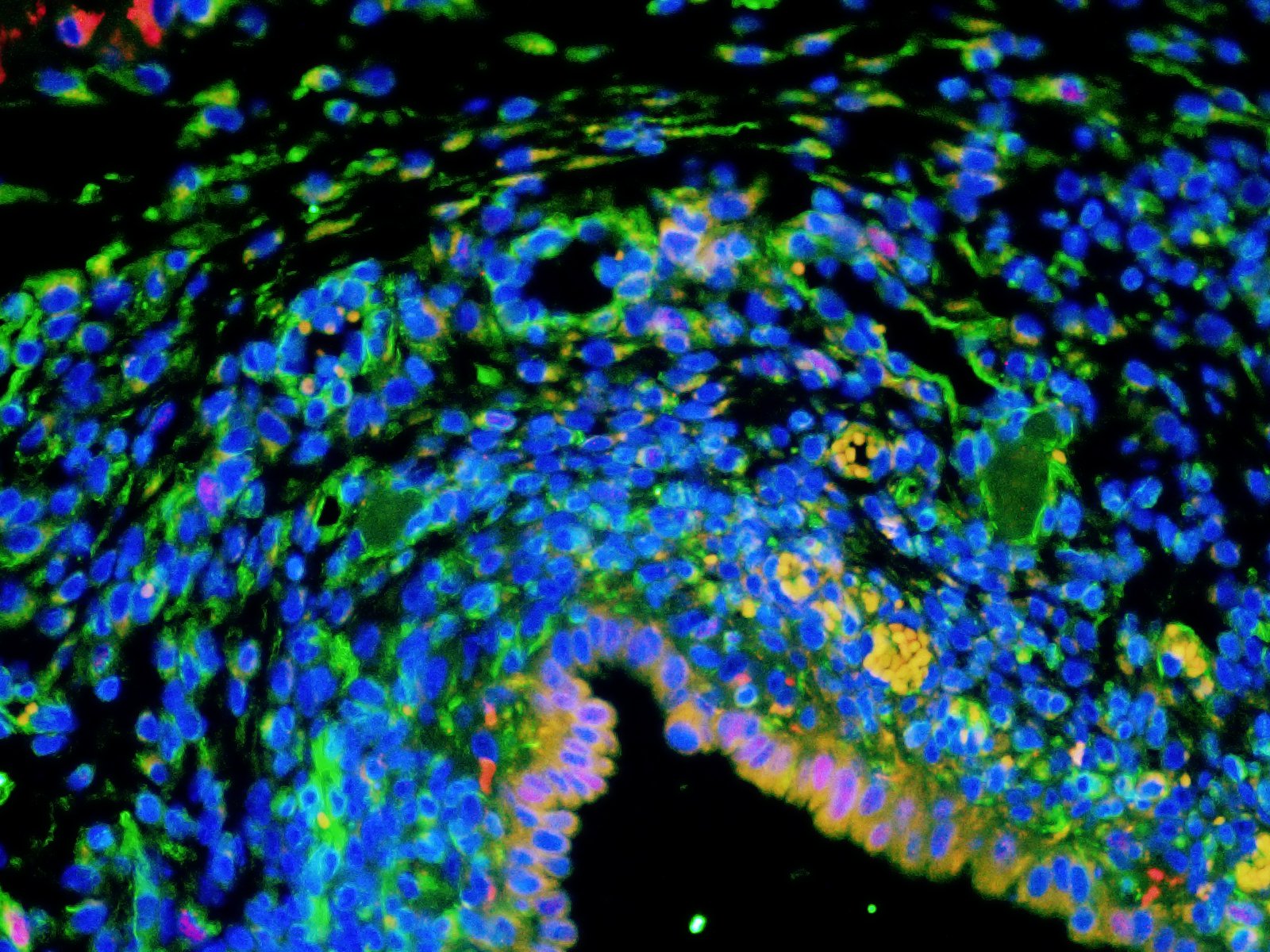

Supplement: Supplementary file 10 — Source data Fig. 8 [file 44318_2024_220_MOESM10_ESM.zip › Figure8/8G/CDKN1A-400-Met (3).jpg]

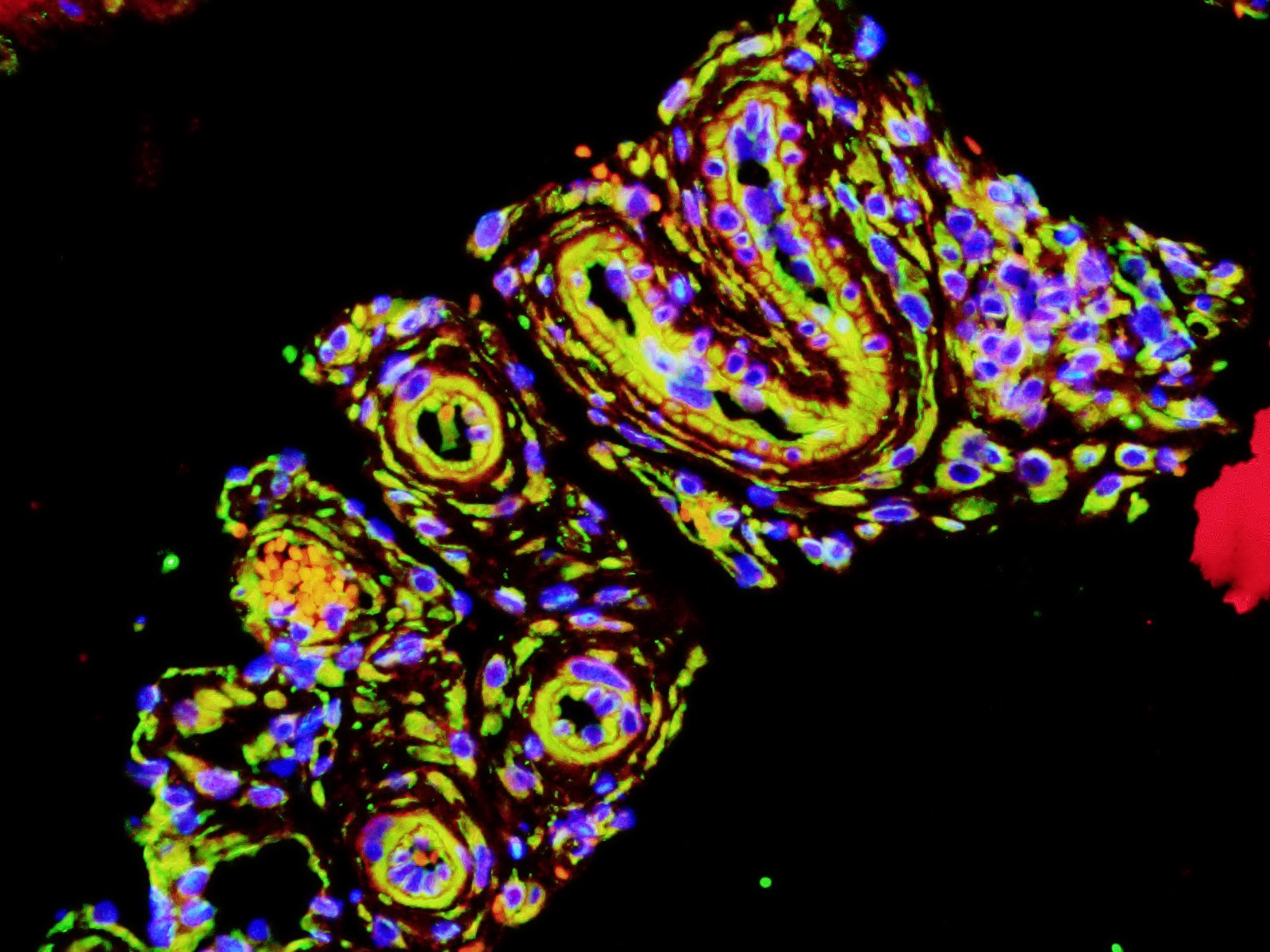

Supplement: Supplementary file 10 — Source data Fig. 8 [file 44318_2024_220_MOESM10_ESM.zip › Figure8/8G/CDKN2A-400-Ctrl (1).jpg]

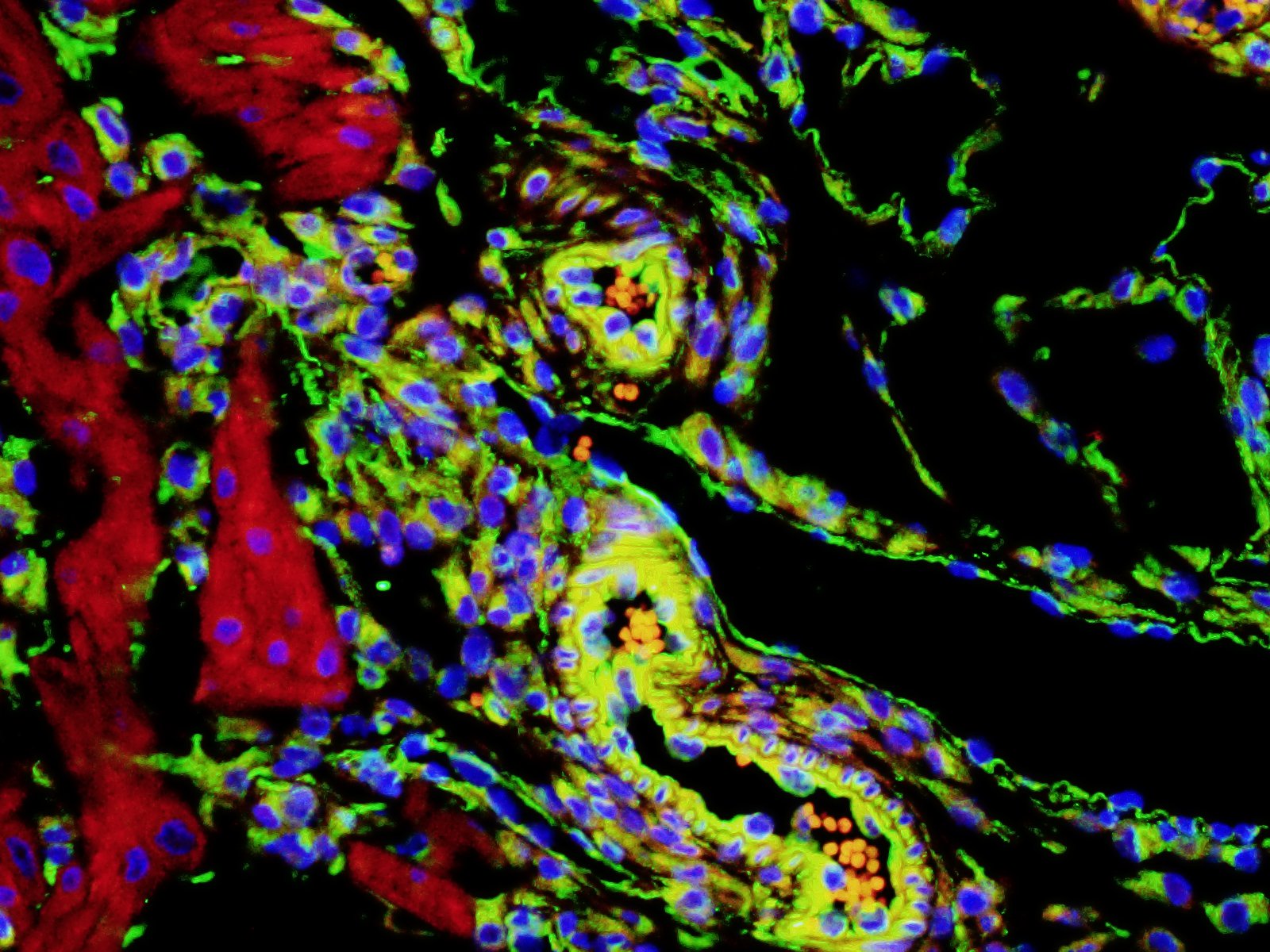

Supplement: Supplementary file 10 — Source data Fig. 8 [file 44318_2024_220_MOESM10_ESM.zip › Figure8/8G/CDKN2A-400-Ctrl (2).jpg]

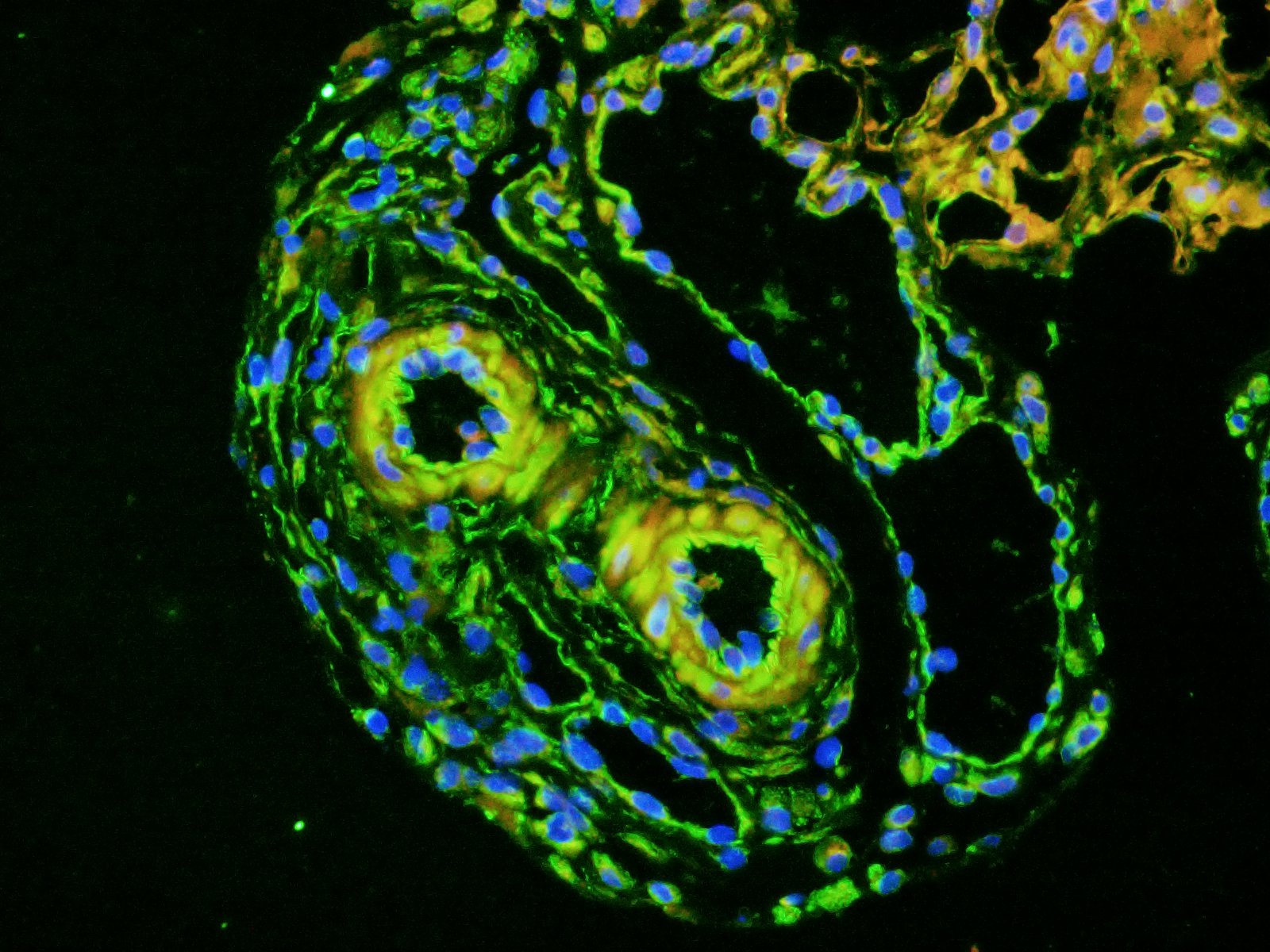

Supplement: Supplementary file 10 — Source data Fig. 8 [file 44318_2024_220_MOESM10_ESM.zip › Figure8/8G/CDKN2A-400-Met (1).jpg]

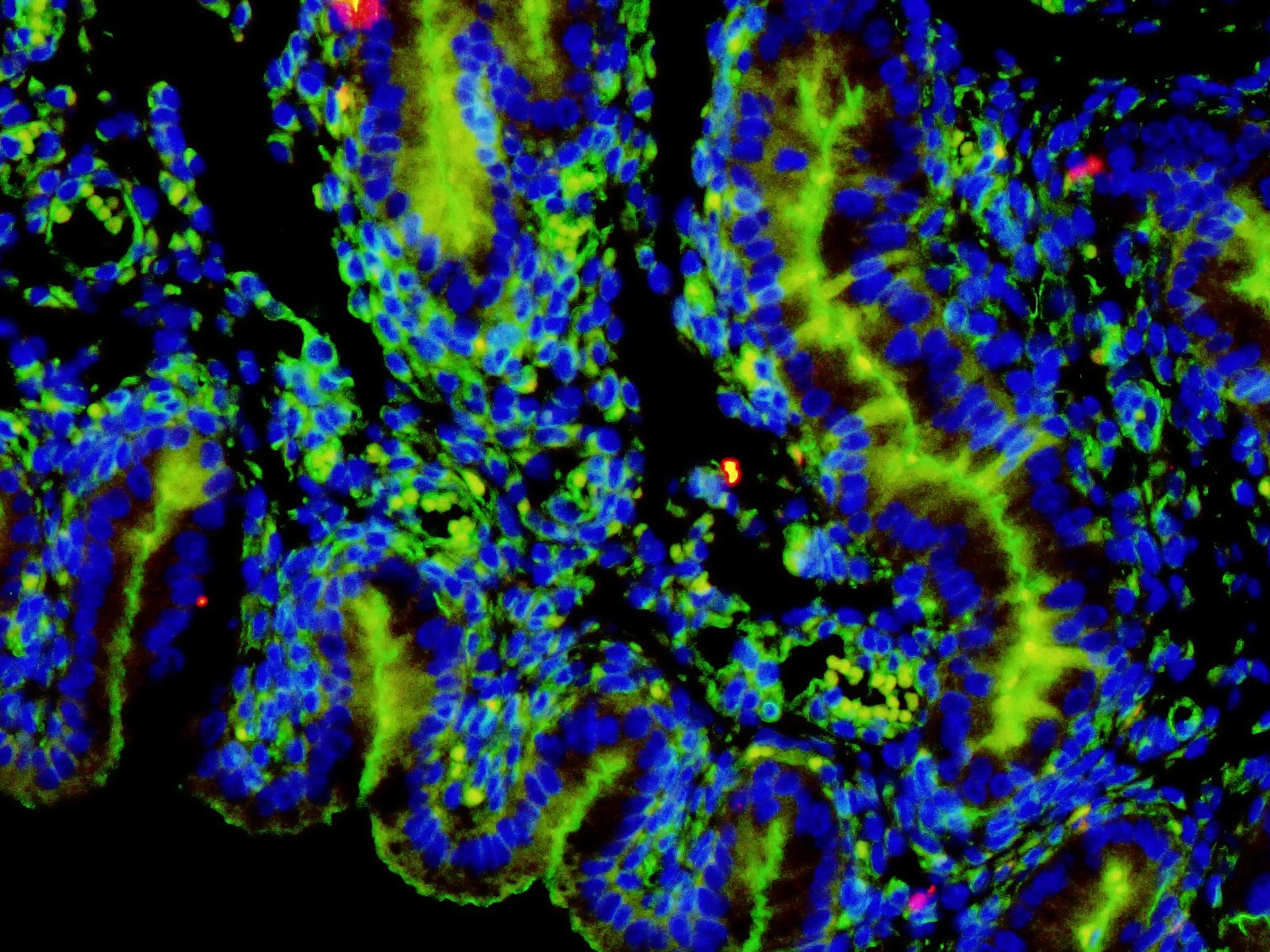

Supplement: Supplementary file 10 — Source data Fig. 8 [file 44318_2024_220_MOESM10_ESM.zip › Figure8/8G/CDKN2A-400-Met (2).jpg]

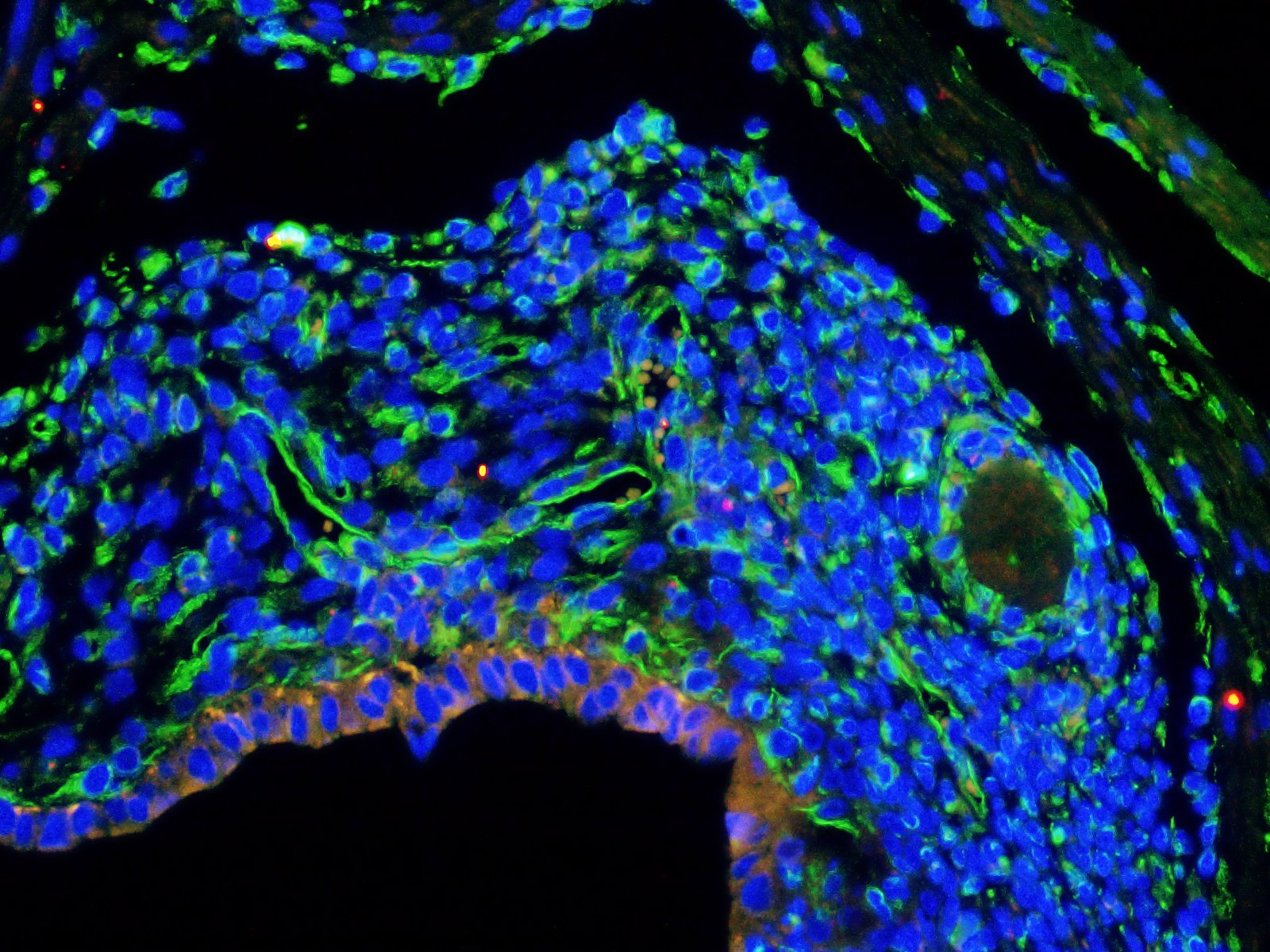

Supplement: Supplementary file 10 — Source data Fig. 8 [file 44318_2024_220_MOESM10_ESM.zip › Figure8/8G/CDKN2A-400-Met (3).jpg]

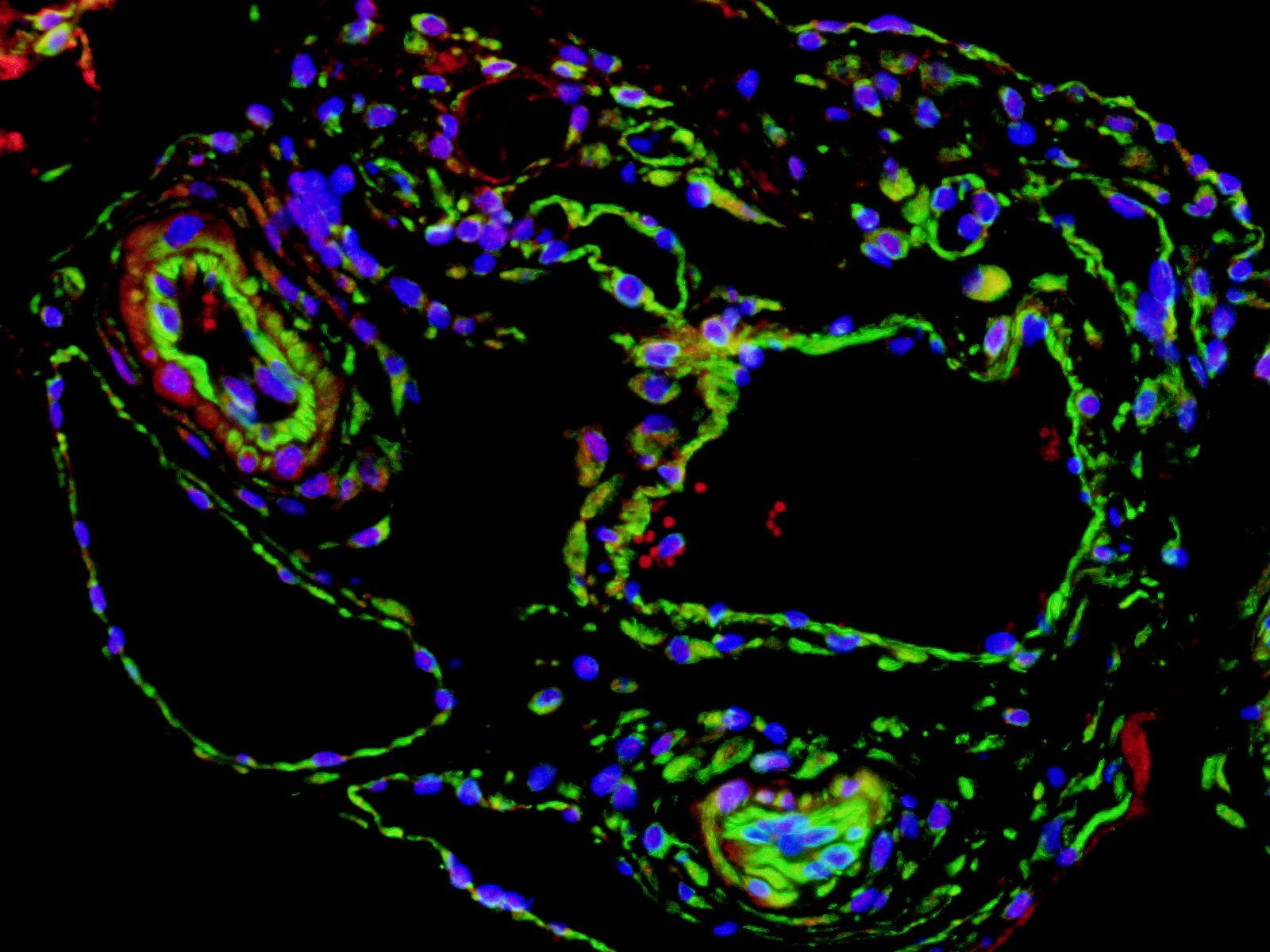

Supplement: Supplementary file 10 — Source data Fig. 8 [file 44318_2024_220_MOESM10_ESM.zip › Figure8/8G/TP53-400-Met (1).jpg]

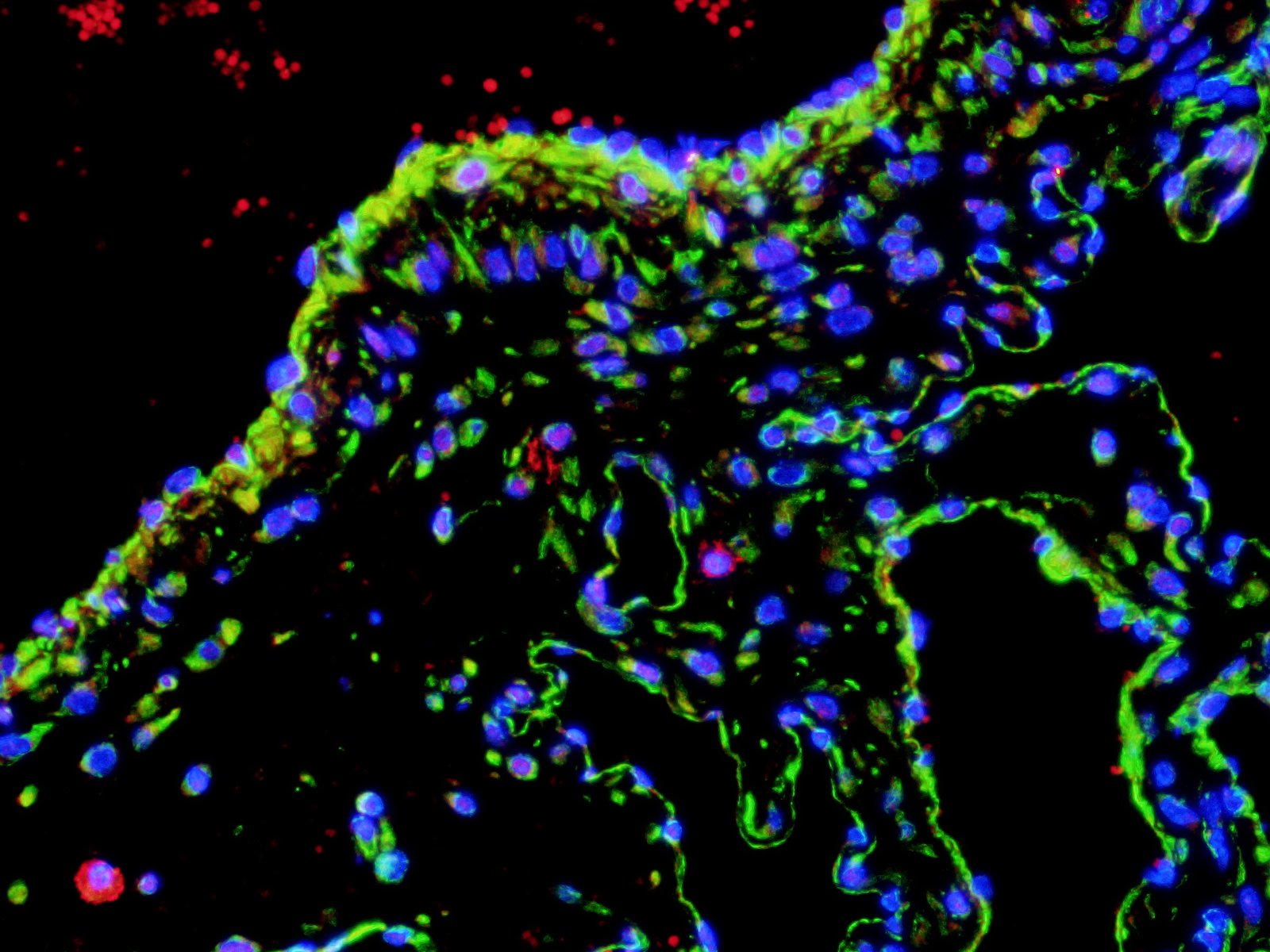

Supplement: Supplementary file 10 — Source data Fig. 8 [file 44318_2024_220_MOESM10_ESM.zip › Figure8/8G/TP53-400-Met (2).jpg]

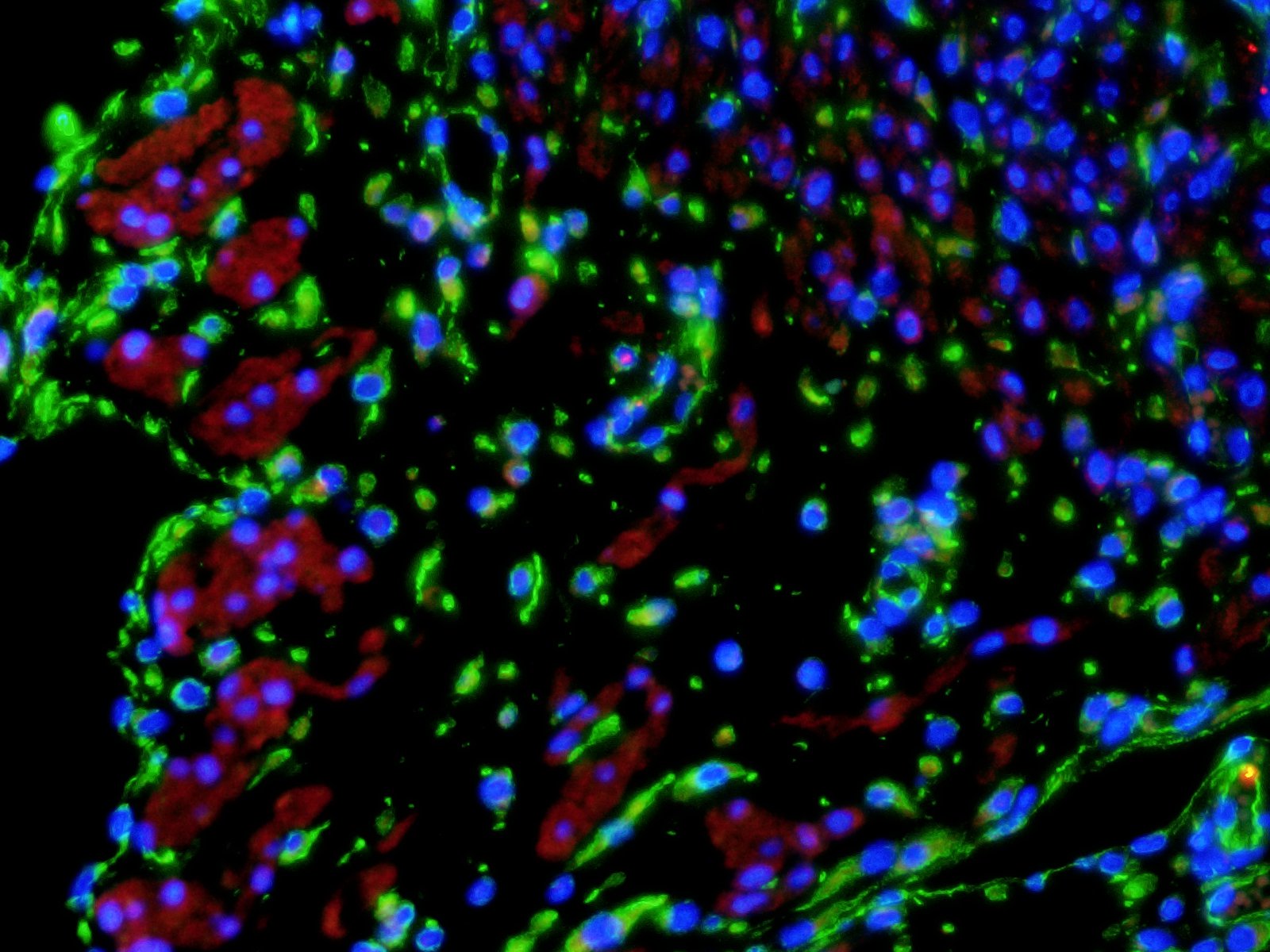

Supplement: Supplementary file 10 — Source data Fig. 8 [file 44318_2024_220_MOESM10_ESM.zip › Figure8/8G/TP53-400-Met (3).jpg]
